# Supplementary material for: What is the quantity, quality and type of systematic review evidence available to inform the optimal prescribing of statins and antihypertensives? A systematic umbrella review and evidence and gap map
Source: BMJ Open. 2024 Feb 24;14(2):e072502. doi: 10.1136/bmjopen-2023-072502 (PMC10895245; doi:10.1136/bmjopen-2023-072502)
Supplement: Supplementary data [file bmjopen-2023-072502supp001.pdf]

APPENDIX A: SEARCH STRATEGY

Bibliographic database searches

Drugs that cause dependency

Database: Cochrane Database of Systematic Reviews
Host: Cochrane Library
Issue: Issue 8 of 12, August 2020
Date Searched: 11/8/2020
Searcher: SB
Hits: 111
Strategy:

#1 (((appropriate* or discontinu* or enhance* or inappropriate* or incorrect* or "in correct*" or optim* or safe or suboptim* or "sub optim*" or tapering or withdrawal) NEAR/4 (drug* or medicine* or medication* or prescri*))):ti,ab,kw

#2 MeSH descriptor: [Inappropriate Prescribing] this term only

#3 MeSH descriptor: [Drug Prescriptions] explode all trees

#4 (((drug* or guideline* or guidance or medicine* or medication* or patient* or prescri*) NEAR/4 (adhere* or compliance or concordance))):ti,ab,kw

#5 MeSH descriptor: [Patient Compliance] explode all trees

#6 ((shared or sharing or informed) near/2 (decision* or choice*)):ti,ab,kw

#7 ((decision near/2 (aid* or support*))):ti,ab,kw

#8 MeSH descriptor: [Decision Making] this term only

#9 MeSH descriptor: [Decision Support Techniques] this term only

#10 (((consumer* or patient*) near/3 (involv* or participat*))):ti,ab,kw

#11 MeSH descriptor: [Patient Participation] this term only

#12 ((patient NEXT cent* near/2 (approach* or care or decision* or intervention* or treatment*))):ti,ab,kw

#13 MeSH descriptor: [Patient-Centered Care] this term only

#14 ((behavi* near/2 chang*)):ti,ab,kw

#15 ((restriction near/2 (policy or policies))):ti,ab,kw

#16 {Wilson, #1-#15}

#17 (benzodiazepine*):ti,ab,kw

#18 ((alprazolam or flunitrazepam or chlordiazepoxide or clobazam or clonazepam or diazepam or lorazepam or midazolam or nitrazepam or oxazepam or prazepam or temazepam)):ti,ab,kw

#19 MeSH descriptor: [Benzodiazepines] explode all trees

Shaw L, *et al. BMJ Open* 2024; 14:e072502. doi: 10.1136/bmjopen-2023-072502

#20 ((antidepres* or "anti depres*")):ti,ab,kw

#21 ((serotonin or norepinephrine or noradrenaline or neurotransmitter* or dopamin* or SSRI* or SNRI* or NARI* or SARI* or NDRI* or tricyclic* or tetracyclic*)):ti,ab,kw

#22 MeSH descriptor: [Antidepressive Agents] explode all trees

#23 ((opioid* or opiate*)):ti,ab,kw

#24 ((morphine or hydromorphone or levorphanol or meperidine or methadone or propoxyphene of codeine or pentazocine or hydrocodone or oxycodone or fentanyl or tramadol)):ti,ab,kw

#25 MeSH descriptor: [Analgesics, Opioid] explode all trees

#26 ("z drug*"):ti,ab,kw

#27 ((zopiclone or zolpidem or zaleplon or eszopiclone)):ti,ab,kw

#28 MeSH descriptor: [Hypnotics and Sedatives] explode all trees

#29 ((gabapentin* or mirogabalin or phenibut or pregabalin)):ti,ab,kw

#30 MeSH descriptor: [Gabapentin] this term only

#31 {Ahmed, #17-#30}

#32 #16 AND #31 in Cochrane Reviews [Drugs that cause dependency search results]

#33 (statin*):ti,ab,kw

#34 (("reductase inhibitors" or "HMGCR inhibitors")):ti,ab,kw

#35 MeSH descriptor: [Hydroxymethylglutaryl-CoA Reductase Inhibitors] explode all trees

#36 ((atorvastatin or Lipitor or Torvast)):ti,ab,kw

#37 ((fluvastatin or Lescol)):ti,ab,kw

#38 ((lovastatin or Mevacor or Altocor or Altoprev)):ti,ab,kw

#39 ((pitavastatin or Livalo or Pitava)):ti,ab,kw

#40 ((pravastatin or Pravachol or Selektine or Lipostat)):ti,ab,kw

#41 ((rosuvastatin or Crestor)):ti,ab,kw

#42 ((simvastatin or Zocor or Lipex)):ti,ab,kw

#43 (antihypertensive*):ti,ab,kw

#44 MeSH descriptor: [Antihypertensive Agents] explode all trees

#45 ((ACE NEXT inhibitor* or antagonist or "angiotensin II receptor" or beta NEXT blocker* or calcium NEXT channel NEXT blocker* or thiazide NEXT diuretic*)):ti,ab,kw

#46 ((acebutolol or adrenomedullin or alprenolol or amlodipine or atenolol or bendroflumethiazide or bepridil or betaxolol or bethanidine or bimatoprost or bisoprolol or bosentan or "bretylium tosylate" or brimonidine tartrate or bupranolol or captopril or carteolol or

carvedilol or celiprolol or chlorisondamine or chlorothiazide or chlorthalidone or cilazapril or clonidine or cromakalim or cyclopenthiazide or debrisoquin or diazoxide or dihydralazine or dihydroalprenolol or diltiazem or doxazosin or enalapril or enalaprilat or eplerenone or epoprostenol or felodipine or fenoldopam or fosinopril or guanabenz or guanethidine or guanfacine or hexamethonium or "hexamethonium compound*" or hydralazine or hydrochlorothiazide or hydroflumethiazide or indapamide or indoramin or irbesartan or isradipine or kallidin or ketanserin or labetalol or latanoprost or lisinopril or losartan or mecamylamine or methyldopa or metipranolol or metolazone or metoprolol or mibefradil or minoxidil or muzolimine or nadolol or nebivolol or nicardipine or nicorandil or nimodipine or nisoldipine or nitrendipine or nitroprusside or olmesartan medoxomil or oxprenolol or pargyline or pempidine or penbutolol or "pentolinium tartrate" or perindopril or phenoxybenzamine or phentolamine or pinacidil or pindolol or piperoxan or polythiazide or prazosin or propranolol or protoveratrines or quinapril or ramipril or reserpine or rilmenidine or telmisartan or teprotide or terlipressin or ticrynafen or timolol or todralazine or tolazoline or torsemide or travoprost or trichlormethiazide or trimethaphan or valsartan or "veratrum alkaloid*" or vincamine or xipamide)):ti,ab,kw

#47 (103-#46)

#48 (cardiovascular):ti,ab,kw

#49 MeSH descriptor: [Cardiovascular Diseases] explode all trees

#50 (((cardiac or coronary or heart) near/3 (arrest* or attack* or disease* or failure*))):ti,ab,kw

#51 ((heart or myocard* or transient) near/3 (infarc* or ischaemi* or ischemi*)):ti,ab,kw

#52 (angina*):ti,ab,kw

#53 MeSH descriptor: [Angina Pectoris] this term only

#54 (stroke*):ti,ab,kw

#55 MeSH descriptor: [Stroke] explode all trees

#56 {Ford, #48-#55}

#57 #16 and #47 and #56 in Cochrane Reviews [Statins search results]

Notes: Date limited January 2010 – to date of search.

Database: CINAHL
Host: EBSCO
Issue: n/a
Date Searched: 11/8/2020
Searcher: SB
Hits: 372
Strategy

1. TI ((appropriate* or discontinu* or enhance* or inappropriate* or incorrect* or "in correct*" or optim* or safe or suboptim* or "sub optim*" or tapering or withdrawal) N3 (drug* or medicine* or medication* or prescri*)) OR AB ((appropriate* or discontinu* or enhance* or inappropriate* or incorrect* or "in correct*" or optim* or safe or suboptim* or

Shaw L, et al. BMJ Open 2024; 14:e072502. doi: 10.1136/bmjopen-2023-072502

"sub optim*" or tapering or withdrawal) N3 (drug* or medicine* or medication* or prescri*))

2. (MH "Inappropriate Prescribing")
3. (MM "Prescriptions, Drug+")
4. TI ((drug* or guideline* or guidance or medicine* or medication* or patient* or prescri*) N3 (adhere* or compliance or concordance)) OR AB ((drug* or guideline* or guidance or medicine* or medication* or patient* or prescri*) N3 (adhere* or compliance or concordance))
5. (MM "Patient Compliance+")
6. TI ((shared or sharing or informed) N1 (decision* or choice*)) OR AB ((shared or sharing or informed) N1 (decision* or choice*))
7. TI (decision N1 (aid* or support*)) OR AB (decision N1 (aid* or support*))
8. (MM "Decision Making")
9. (MM "Decision Support Techniques")
10. TI ((consumer* or patient*) N2 (involv* or participat*)) OR AB ((consumer* or patient*) N2 (involv* or participat*))
11. (MM "Consumer Participation")
12. TI ("patient cent*" N1 (approach* or care or decision* or intervention* or treatment*)) OR AB ("patient cent*" N1 (approach* or care or decision* or intervention* or treatment*))
13. (MH "Patient Centered Care")
14. TI (behavi* N1 chang*) OR AB (behavi* N1 chang*)
15. TI (restriction N1 (policy or policies)) OR AB (restriction N1 (policy or policies))
16. S1 OR S2 OR S3 OR S4 OR S5 OR S6 OR S7 OR S8 OR S9 OR S10 OR S11 OR S12 OR S13 OR S14 OR S15
17. TI benzodiazepine* OR AB benzodiazepine*
18. TI (alprazolam or flunitrazepam or chlordiazepoxide or clobazam or clonazepam or diazepam or lorazepam or midazolam or nitrazepam or oxazepam or prazepam or temazepam) OR AB (alprazolam or flunitrazepam or chlordiazepoxide or clobazam or clonazepam or diazepam or lorazepam or midazolam or nitrazepam or oxazepam or prazepam or temazepam)
19. (MH "Antianxiety Agents, Benzodiazepine+")
20. TI (antidepres* or "anti depres*") OR AB (antidepres* or "anti depres*")
21. TI ((serotonin or norepinephrine or noradrenaline or neurotransmitter* or dopamin* or SSRI* or SNRI* or NARI* or SARI* or NDRI* or tricyclic* or tetracyclic*)) OR AB ((serotonin or norepinephrine or noradrenaline or neurotransmitter* or dopamin* or SSRI* or SNRI* or NARI* or SARI* or NDRI* or tricyclic* or tetracyclic*))

22. (MH "Antidepressive Agents+")
23. TI ((opioid* or opiate*)) OR AB ((opioid* or opiate*))
24. TI ((morphine or hydromorphone or levorphanol or meperidine or methadone or propoxyphene of codeine or pentazocine or hydrocodone or oxycodone or fentanyl or tramadol)) OR AB ((morphine or hydromorphone or levorphanol or meperidine or methadone or propoxyphene of codeine or pentazocine or hydrocodone or oxycodone or fentanyl or tramadol))
25. (MH "Analgesics, Opioid+")
26. TI "z drug*" OR AB "z drug*"
27. TI ((zopiclone or zolpidem or zaleplon or eszopiclone)) OR AB ((zopiclone or zolpidem or zaleplon or eszopiclone))
28. (MH "Hypnotics and Sedatives")
29. TI ((gabapentin* or mirogabalin or phenibut or pregabalin)) OR AB ((gabapentin* or mirogabalin or phenibut or pregabalin))
30. (MH "Gabapentin")
31. S17 OR S18 OR S19 OR S20 OR S21 OR S22 OR S23 OR S24 OR S25 OR S26 OR S27 OR S28 OR S29 OR S30
32. S16 AND S31
33. TI statin* OR AB statin*
34. TI ("HMG-CoA reductase inhibitor*" or "3-hydroxy-3-methyl-glutaryl-CoA reductase inhibitor*" or "3-hydroxy-3-methylglutaryl coenzyme A reductase inhibitor*" or "HMGCR inhibitor*" or "Hydroxymethylglutaryl-CoA Reductase Inhibitor*") OR AB ("HMG-CoA reductase inhibitor*" or "3-hydroxy-3-methyl-glutaryl-CoA reductase inhibitor*" or "3-hydroxy-3-methylglutaryl coenzyme A reductase inhibitor*" or "HMGCR inhibitor*" or "Hydroxymethylglutaryl-CoA Reductase Inhibitor*")
35. (MH "Statins+")
36. TI (atorvastatin or Lipitor or Torvast) OR AB (atorvastatin or Lipitor or Torvast)
37. TI (fluvastatin or Lescol) OR AB (fluvastatin or Lescol)
38. TI (lovastatin or Mevacor or Altocor or Altoprev) OR AB (lovastatin or Mevacor or Altocor or Altoprev)
39. TI (pitavastatin or Livalo or Pitava) OR AB (pitavastatin or Livalo or Pitava)
40. TI (pravastatin or Pravachol or Selektine or Lipostat) OR AB (pravastatin or Pravachol or Selektine or Lipostat)
41. TI (rosuvastatin or Crestor) OR AB (rosuvastatin or Crestor)
42. TI (simvastatin or Zocor or Lipex) OR AB (simvastatin or Zocor or Lipex)
43. TI antihypertensive* OR AB antihypertensive*

Shaw L, *et al. BMJ Open* 2024; 14:e072502. doi: 10.1136/bmjopen-2023-072502

44. (MH "Antihypertensive Agents+")
45. TI ("ACE inhibitor*" or antagonist or "angiotensin II receptor" or "beta blocker*" or "calcium channel blocker*" or "thiazide diuretic*") OR AB ("ACE inhibitor*" or antagonist or "angiotensin II receptor" or "beta blocker*" or "calcium channel blocker*" or "thiazide diuretic*")
46. TI (acebutolol or adrenomedullin or alprenolol or amlodipine or atenolol or bendroflumethiazide or bepridil or betaxolol or bethanidine or bimatoprost or bisoprolol or bosentan or "bretylium tosylate" or brimonidine tartrate or bupranolol or captopril or carteolol or carvedilol or celiprolol or chlorisondamine or chlorothiazide or chlorthalidone or cilazapril or clonidine or cromakalim or cyclopenthiazide or debrisoquin or diazoxide or dihydralazine or dihydroalprenolol or diltiazem or doxazosin or enalapril or enalaprilat or eplerenone or epoprostenol or felodipine or fenoldopam or fosinopril or guanabenz or guanethidine or guanfacine or hexamethonium or "hexamethonium compound*" or hydralazine or hydrochlorothiazide or hydroflumethiazide or indapamide or indoramin or irbesartan or isradipine or kallidin or ketanserin or labetalol or latanoprost or lisinopril or losartan or mecamylamine or methyldopa or metipranolol or metolazone or metoprolol or mibefradil or minoxidil or muzolimine or nadolol or nebivolol or nicardipine or nicorandil or nimodipine or nisoldipine or nitrendipine or nitroprusside or olmesartan medoxomil or oxprenolol or pargyline or pempidine or penbutolol or "pentolinium tartrate" or perindopril or phenoxybenzamine or phentolamine or pinacidil or pindolol or piperoxan or polythiazide or prazosin or propranolol or protoveratrines or quinapril or ramipril or reserpine or rilmenidine or telmisartan or teprotide or terlipressin or ticrynafen or timolol or todralazine or tolazoline or torsemide or travoprost or trichlormethiazide or trimethaphan or valsartan or "veratrum alkaloid*" or vincamine or xipamide) OR AB (acebutolol or adrenomedullin or alprenolol or amlodipine or atenolol or bendroflumethiazide or bepridil or betaxolol or bethanidine or bimatoprost or bisoprolol or bosentan or "bretylium tosylate" or brimonidine tartrate or bupranolol or captopril or carteolol or carvedilol or celiprolol or chlorisondamine or chlorothiazide or chlorthalidone or cilazapril or clonidine or cromakalim or cyclopenthiazide or debrisoquin or diazoxide or dihydralazine or dihydroalprenolol or diltiazem or doxazosin or enalapril or enalaprilat or eplerenone or epoprostenol or felodipine or fenoldopam or fosinopril or guanabenz or guanethidine or guanfacine or hexamethonium or "hexamethonium compound*" or hydralazine or hydrochlorothiazide or hydroflumethiazide or indapamide or indoramin or irbesartan or isradipine or kallidin or ketanserin or labetalol or latanoprost or lisinopril or losartan or mecamylamine or methyldopa or metipranolol or metolazone or metoprolol or mibefradil or minoxidil or muzolimine or nadolol or nebivolol or nicardipine or nicorandil or nimodipine or nisoldipine or nitrendipine or nitroprusside or olmesartan medoxomil or oxprenolol or pargyline or pempidine or penbutolol or "pentolinium tartrate" or perindopril or phenoxybenzamine or phentolamine or pinacidil or pindolol or piperoxan or polythiazide or prazosin or propranolol or protoveratrines or quinapril or ramipril or reserpine or rilmenidine or telmisartan or teprotide or terlipressin or ticrynafen or timolol or todralazine or tolazoline or torsemide or travoprost or trichlormethiazide or trimethaphan or valsartan or "veratrum alkaloid*" or vincamine or xipamide)
47. S33 OR S34 OR S35 OR S36 OR S37 OR S38 OR S39 OR S40 OR S41 OR S42 OR S43 OR S44 OR S45 OR S46
48. TI cardiovascular OR AB cardiovascular

49. (MH "Cardiovascular Diseases+")
50. TI ((cardiac or coronary or heart) N2 (arrest* or attack* or disease* or failure*)) OR AB ((cardiac or coronary or heart) N2 (arrest* or attack* or disease* or failure*))
51. TI ((heart or myocard* or transient) N2 (infarc* or ischaemi* or ischemi*)) OR AB ((heart or myocard* or transient) N2 (infarc* or ischaemi* or ischemi*))
52. TI angina* OR AB angina*
53. (MH "Angina Pectoris")
54. TI stroke* OR AB stroke*
55. (MH "Stroke+")
56. S48 OR S49 OR S50 OR S51 OR S52 OR S53 OR S54 OR S55
57. S16 AND S47 AND S56
58. TI ((effectiveness or implementation or literature or map or mapping or qualitative or rapid or realist or systematic or scoping or "state of the art" or umbrella) N1 (assessment* or overview* or review* or synthes*)) OR AB ((effectiveness or implementation or literature or map or mapping or qualitative or rapid or realist or systematic or scoping or "state of the art" or umbrella) N1 (assessment* or overview* or review* or synthes*))
59. TI ("meta analy*" or metaanaly* or metasynthe* or "meta synthe*") OR AB ("meta analy*" or metaanaly* or metasynthe* or "meta synthe*")
60. TI ((systematic or evidence) N0 assess*) OR AB ((systematic or evidence) N0 assess*)
61. TI (qualitative N1 (evidence or synthes*)) OR AB (qualitative N1 (evidence or synthes*))
62. TI overarching N1 model OR AB overarching N1 model
63. TI "review* of reviews" OR AB "review* of reviews"
64. S58 OR S59 OR S60 OR S61 OR S62 OR S63
65. S32 AND S64 [Drugs that cause dependency search results]
66. S57 AND S64 [Statins search results]

Notes: Date limited 2010 to date of search.

Database: Epistemonikos
Host: https://www.epistemonikos.org/en/
Issue: n/a
Date Searched: 12/8/2020
Searcher: SB
Hits: 56
Strategy

1. title:(prescription* OR prescribing OR prescribe*) OR abstract:(prescription* OR prescribing OR prescribe*)

2. title:(optimi* OR inappropriat* OR enhance*) OR abstract:(optimi* OR inappropriat* OR enhance*)
3. title:(benzodiazepine*) OR abstract:(benzodiazepine*) OR title:(antidepres* OR "anti depres*") OR abstract:(antidepres* OR "anti depres*") OR title:(opioid* OR opiate*) OR abstract:(opioid* OR opiate*) OR title:("z drug*") OR abstract:("z drug*") OR title:(gabapentin*) OR abstract:(gabapentin*)
4. 1 AND 2 AND 3

Notes: Date limited 2010 to 2020.

Database: medRxiv
Host: 12/8/2020
Issue: n/a
Date Searched:
Searcher: SB
Hits: 87
Strategy:

optimi* and prescri* and benzodiazepine *	5 hits
optimi* and prescri* and antidepres*	5 hits
optimi* and prescri* and opioid*	11 hits
optimi* and prescri* and "z drug*	66 hits
optimi* and prescri* and gabapentin*	0 hits

Database: Embase
Host: Ovid
Issue: 1974 to 2020 August 10
Date Searched: 11/8/2020
Searcher: SB
Hits: 1776
Strategy:

1. ((appropriate* or discontinu* or enhance* or inappropriate* or incorrect* or "in correct*" or optim* or safe or suboptim* or "sub optim*" or tapering or withdrawal) adj4 (drug* or medicine* or medication* or prescri*)).tw.
2. exp *inappropriate prescribing/
3. *prescription/
4. ((drug* or guideline* or guidance or medicine* or medication* or patient* or prescri*) adj4 (adhere* or compliance or concordance)).tw.
5. exp *patient compliance/
6. medication compliance/

7. ((shared or sharing or informed) adj2 (decision* or choice*)).tw.
8. (decision adj2 (aid* or support*)).tw.
9. exp *decision making/
10. exp *decision support system/
11. ((consumer* or patient*) adj3 (involv* or participat*)).tw.
12. *patient participation/
13. ("patient cent*" adj2 (approach* or care or decision* or intervention* or treatment*)).tw.
14. *patient care/
15. (behavi* adj2 chang*).tw.
16. *behavior change/
17. (restriction adj2 (policy or policies)).tw.
18. or/1-17
19. benzodiazepine*.tw.
20. (alprazolam or flunitrazepam or chlordiazepoxide or clobazam or clonazepam or diazepam or lorazepam or midazolam or nitrazepam or oxazepam or prazepam or temazepam).tw.
21. exp benzodiazepine derivative/
22. (antidepres* or "anti depres*").tw.
23. (serotonin or norepinephrine or noradrenaline or neurotransmitter* or dopamin* or SSRI* or SNRI* or NARI* or SARI* or NDRI* or tricyclic* or tetracyclic*).tw.
24. exp antidepressant agent/
25. (opioid* or opiate*).tw.
26. (morphine or hydromorphone or levorphanol or meperidine or methadone or propoxyphene of codeine or pentazocine or hydrocodone or oxycodone or fentanyl or tramadol).tw.
27. exp opiate/
28. "z drug*".tw.
29. (zopiclone or zolpidem or zaleplon or eszopiclone).tw.
30. hypnotic agent/
31. sedative agent/
32. (gabapentin* or mirogabalin or phenibut or pregabalin).tw.
33. gabapentin/
34. or/19-33
35. 18 and 34

36. statin*.tw.
37. ("HMG-CoA reductase inhibitor*" or "3-hydroxy-3-methyl-glutaryl-CoA reductase inhibitor*" or "3-hydroxy-3-methylglutaryl coenzyme A reductase inhibitor*" or "HMGCR inhibitor*" or "Hydroxymethylglutaryl-CoA Reductase Inhibitor*").tw.
38. "statin (protein)"/
39. (atorvastatin or Lipitor or Torvast).tw.
40. (fluvastatin or Lescol).tw.
41. (lovastatin or Mevacor or Altocor or Altoprev).tw.
42. (pitavastatin or Livalo or Pitava).tw.
43. (pravastatin or Pravachol or Selektine or Lipostat).tw.
44. (rosuvastatin or Crestor).tw.
45. (simvastatin or Zocor or Lipex).tw.
46. antihypertensive*.tw.
47. exp antihypertensive agent/
48. ("ACE inhibitor*" or antagonist or "angiotensin II receptor" or "beta blocker*" or "calcium channel blocker*" or "thiazide diuretic*").tw.
49. (acebutolol or adrenomedullin or alprenolol or amlodipine or atenolol or bendroflumethiazide or bepridil or betaxolol or bethanidine or bimatoprost or bisoprolol or bosentan or "bretylium tosylate" or brimonidine tartrate or bupranolol or captopril or carteolol or carvedilol or celiprolol or chlorisondamine or chlorothiazide or chlorthalidone or cilazapril or clonidine or cromakalim or cyclopenthiazide or debrisoquin or diazoxide or dihydralazine or dihydroalprenolol or diltiazem or doxazosin or enalapril or enalaprilat or eplerenone or epoprostenol or felodipine or fenoldopam or fosinopril or guanabenz or guanethidine or guanfacine or hexamethonium or "hexamethonium compound*" or hydralazine or hydrochlorothiazide or hydroflumethiazide or indapamide or indoramin or irbesartan or isradipine or kallidin or ketanserin or labetalol or latanoprost or lisinopril or losartan or mecamylamine or methyldopa or metipranolol or metolazone or metoprolol or mibefradil or minoxidil or muzolimine or nadolol or nebivolol or nicardipine or nicorandil or nimodipine or nisoldipine or nitrendipine or nitroprusside or olmesartan medoxomil or oxprenolol or pargyline or pempidine or penbutolol or "pentolinium tartrate" or perindopril or phenoxybenzamine or phentolamine or pinacidil or pindolol or piperoxan or polythiazide or prazosin or propranolol or protoveratrines or quinapril or ramipril or reserpine or rilmenidine or telmisartan or teprotide or terlipressin or ticrynafen or timolol or todralazine or tolazoline or torsemide or travoprost or trichlormethiazide or trimethaphan or valsartan or "veratrum alkaloid*" or vincamine or xipamide).tw.
50. or/36-49
51. cardiovascular.tw.
52. exp cardiovascular disease/

Shaw L, *et al. BMJ Open* 2024; 14:e072502. doi: 10.1136/bmjopen-2023-072502

53. ((cardiac or coronary or heart) adj3 (arrest* or attack* or disease* or failure*)).tw.
54. ((heart or myocard* or transient) adj3 (infarc* or ischaemi* or ischemi*)).tw.
55. angina*.tw.
56. Angina Pectoris/
57. stroke*.tw.
58. exp cerebrovascular accident/
59. or/51-58
60. 18 and 50 and 59
61. ((effectiveness or implementation or literature or map or mapping or qualitative or rapid or realist or systematic or scoping or "state of the art" or umbrella) adj2 (assessment* or overview* or review* or synthes*)).tw.
62. ("meta analy*" or metaanaly* or metasynthe* or "meta synthe*").tw.
63. ((systematic or evidence) adj1 assess*).tw.
64. (qualitative adj2 (evidence or synthes*)).tw.
65. (overarching adj2 model).tw.
66. "review* of reviews".tw.
67. or/61-66
68. 35 and 67 [Drugs that cause dependency search results]
69. 60 and 67 [Statins search results]

Notes: Date limited 2010 to date of search.

Database: Health Management Information Consortium
Host: Ovid
Issue: 1979 to July 2020
Date Searched: 11/8/2020
Searcher: SB
Hits: 10
Strategy: see MEDLINE search strategy

Database: MEDLINE
Host: Ovid
Issue: 1946 to August 10, 2020
Date Searched: 11/8/2020
Searcher: SB
Hits: 875
Strategy:

1. ((appropriate* or discontinu* or enhance* or inappropriate* or incorrect* or "in correct*" or optim* or safe or suboptim* or "sub optim*" or tapering or withdrawal) adj4 (drug* or medicine* or medication* or prescri*)).tw.
2. Inappropriate Prescribing/
3. exp *Drug Prescriptions/
4. ((drug* or guideline* or guidance or medicine* or medication* or patient* or prescri*) adj4 (adhere* or compliance or concordance)).tw.
5. exp *Patient Compliance/
6. ((shared or sharing or informed) adj2 (decision* or choice*)).tw.
7. (decision adj2 (aid* or support*)).tw.
8. *Decision Making/
9. *decision support techniques/
10. ((consumer* or patient*) adj3 (involv* or participat*)).tw.
11. *patient participation/
12. ("patient cent*" adj2 (approach* or care or decision* or intervention* or treatment*)).tw.
13. Patient-Centered Care/
14. (behavi* adj2 chang*).tw.
15. (restriction adj2 (policy or policies)).tw.
16. or/1-15
17. benzodiazepine*.tw.
18. (alprazolam or flunitrazepam or chlordiazepoxide or clobazam or clonazepam or diazepam or lorazepam or midazolam or nitrazepam or oxazepam or prazepam or temazepam).tw.
19. exp Benzodiazepines/
20. (antidepres* or "anti depres*").tw.
21. (serotonin or norepinephrine or noradrenaline or neurotransmitter* or dopamin* or SSRI* or SNRI* or NARI* or SARI* or NDRI* or tricyclic* or tetracyclic*).tw.
22. exp Antidepressive Agents/
23. (opioid* or opiate*).tw.
24. (morphine or hydromorphone or levorphanol or meperidine or methadone or propoxyphene of codeine or pentazocine or hydrocodone or oxycodone or fentanyl or tramadol).tw.
25. exp Analgesics, Opioid/
26. "z drug*".tw.
27. (zopiclone or zolpidem or zaleplon or eszopiclone).tw.

28. exp "hypnotics and sedatives"/
29. (gabapentin* or mirogabalin or phenibut or pregabalin).tw.
30. Gabapentin/
31. or/17-30
32. 16 and 31
33. statin*.tw.
34. ("HMG-CoA reductase inhibitor*" or "3-hydroxy-3-methyl-glutaryl-CoA reductase inhibitor*" or "3-hydroxy-3-methylglutaryl coenzyme A reductase inhibitor*" or "HMGCR inhibitor*" or "Hydroxymethylglutaryl-CoA Reductase Inhibitor*").tw.
35. exp Hydroxymethylglutaryl-CoA Reductase Inhibitors/
36. (atorvastatin or Lipitor or Torvast).tw.
37. (fluvastatin or Lescol).tw.
38. (lovastatin or Mevacor or Altocor or Altoprev).tw.
39. (pitavastatin or Livalo or Pitava).tw.
40. (pravastatin or Pravachol or Selektine or Lipostat).tw.
41. (rosuvastatin or Crestor).tw.
42. (simvastatin or Zocor or Lipex).tw.
43. antihypertensive*.tw.
44. exp Antihypertensive Agents/
45. ("ACE inhibitor*" or antagonist or "angiotensin II receptor" or "beta blocker*" or "calcium channel blocker*" or "thiazide diuretic*").tw.
46. (acebutolol or adrenomedullin or alprenolol or amlodipine or atenolol or bendroflumethiazide or bepridil or betaxolol or bethanidine or bimatoprost or bisoprolol or bosentan or "bretylium tosylate" or brimonidine tartrate or bupranolol or captopril or carteolol or carvedilol or celiprolol or chlorisondamine or chlorothiazide or chlorthalidone or cilazapril or clonidine or cromakalim or cyclopenthiazide or debrisoquin or diazoxide or dihydralazine or dihydroalprenolol or diltiazem or doxazosin or enalapril or enalaprilat or eplerenone or epoprostenol or felodipine or fenoldopam or fosinopril or guanabenz or guanethidine or guanfacine or hexamethonium or "hexamethonium compound*" or hydralazine or hydrochlorothiazide or hydroflumethiazide or indapamide or indoramin or irbesartan or isradipine or kallidin or ketanserin or labetalol or latanoprost or lisinopril or losartan or mecamylamine or methyldopa or metipranolol or metolazone or metoprolol or mibefradil or minoxidil or muzolimine or nadolol or nebivolol or nicardipine or nicorandil or nimodipine or nisoldipine or nitrendipine or nitroprusside or olmesartan medoxomil or oxprenolol or pargyline or pempidine or penbutolol or "pentolinium tartrate" or perindopril or phenoxybenzamine or phentolamine or pinacidil or pindolol or piperoxan or polythiazide or prazosin or propranolol or protoveratrines or quinapril or ramipril or reserpine or rilmenidine or telmisartan or teprotide or terlipressin or ticrynafen or timolol or todralazine

or tolazoline or torsemide or travoprost or trichlormethiazide or trimethaphan or valsartan or "veratrum alkaloid*" or vincamine or xipamide).tw.

47. or/33-46
48. cardiovascular.tw.
49. exp Cardiovascular Diseases/
50. ((cardiac or coronary or heart) adj3 (arrest* or attack* or disease* or failure*)).tw.
51. ((heart or myocard* or transient) adj3 (infarc* or ischaemi* or ischemi*)).tw.
52. angina*.tw.
53. Angina Pectoris/
54. stroke*.tw.
55. exp Stroke/
56. or/48-55
57. 16 and 47 and 56
58. ((effectiveness or implementation or literature or map or mapping or qualitative or rapid or realist or systematic or scoping or "state of the art" or umbrella) adj2 (assessment* or overview* or review* or synthes*)).tw.
59. ("meta analy*" or metaanaly* or metasynthe* or "meta synthe*").tw.
60. ((systematic or evidence) adj1 assess*).tw.
61. (qualitative adj2 (evidence or synthes*)).tw.
62. (overarching adj2 model).tw.
63. "review* of reviews".tw.
64. systematic review.pt.
65. meta-analysis.pt.
66. or/58-65
67. 32 and 66 [Drugs that cause dependency search results]
68. 57 and 66 [Statins search results]

Notes: Date limited 2010 to date of search.

Database: PsycInfo
Host: Ovid
Issue: 1806 to August Week 1 2020
Date Searched: 11/8/2020
Searcher: SB

Hits: 297
Strategy

1. ((appropriate* or discontinu* or enhance* or inappropriate* or incorrect* or "in correct*" or optim* or safe or suboptim* or "sub optim*" or tapering or withdrawal) adj4 (drug* or medicine* or medication* or prescri*)).tw.
2. prescription drug misuse/
3. exp "prescribing (drugs)"/
4. ((drug* or guideline* or guidance or medicine* or medication* or patient* or prescri*) adj4 (adhere* or compliance or concordance)).tw.
5. *treatment compliance/
6. ((shared or sharing or informed) adj2 (decision* or choice*)).tw.
7. (decision adj2 (aid* or support*)).tw.
8. Decision Making/
9. ((consumer* or patient*) adj3 (involv* or participat*)).tw.
10. patient participation/
11. ("patient cent*" adj2 (approach* or care or decision* or intervention* or treatment*)).tw.
12. (behavi* adj2 chang*).ti,ab.
13. exp behavior change/
14. (restriction adj2 (policy or policies)).tw.
15. or/1-14
16. benzodiazepine*.tw.
17. (alprazolam or flunitrazepam or chlordiazepoxide or clobazam or clonazepam or diazepam or lorazepam or midazolam or nitrazepam or oxazepam or prazepam or temazepam).tw.
18. exp Benzodiazepines/
19. (antidepres* or "anti depres*").tw.
20. (serotonin or norepinephrine or noradrenaline or neurotransmitter* or dopamin* or SSRI* or SNRI* or NARI* or SARI* or NDRI* or tricyclic* or tetracyclic*).tw.
21. exp antidepressant drugs/
22. (opioid* or opiate*).tw.
23. (morphine or hydromorphone or levorphanol or meperidine or methadone or propoxyphene of codeine or pentazocine or hydrocodone or oxycodone or fentanyl or tramadol).tw.
24. exp opiates/
25. "z drug*".tw.

Shaw L, *et al. BMJ Open* 2024; 14:e072502. doi: 10.1136/bmjopen-2023-072502

26. (zopiclone or zolpidem or zaleplon or eszopiclone).tw.
27. exp hypnotic drugs/
28. exp sedatives/
29. (gabapentin* or mirogabalin or phenibut or pregabalin).tw.
30. Gabapentin/
31. or/16-30
32. 15 and 31
33. statin*.tw.
34. ("HMG-CoA reductase inhibitor*" or "3-hydroxy-3-methyl-glutaryl-CoA reductase inhibitor*" or "3-hydroxy-3-methylglutaryl coenzyme A reductase inhibitor*" or "HMGCR inhibitor*" or "Hydroxymethylglutaryl-CoA Reductase Inhibitor*").tw.
35. statins/
36. (atorvastatin or Lipitor or Torvast).tw.
37. (fluvastatin or Lescol).tw.
38. (lovastatin or Mevacor or Altocor or Altoprev).tw.
39. (pitavastatin or Livalo or Pitava).tw.
40. (pravastatin or Pravachol or Selektine or Lipostat).tw.
41. (rosuvastatin or Crestor).tw.
42. (simvastatin or Zocor or Lipex).tw.
43. antihypertensive*.tw.
44. exp antihypertensive drugs/
45. ("ACE inhibitor*" or antagonist or "angiotensin II receptor" or "beta blocker*" or "calcium channel blocker*" or "thiazide diuretic*").tw.
46. (acebutolol or adrenomedullin or alprenolol or amlodipine or atenolol or bendroflumethiazide or bepridil or betaxolol or bethanidine or bimatoprost or bisoprolol or bosentan or "bretylium tosylate" or brimonidine tartrate or bupranolol or captopril or carteolol or carvedilol or celiprolol or chlorisondamine or chlorothiazide or chlorthalidone or cilazapril or clonidine or cromakalim or cyclopenthiazide or debrisoquin or diazoxide or dihydralazine or dihydroalprenolol or diltiazem or doxazosin or enalapril or enalaprilat or eplerenone or epoprostenol or felodipine or fenoldopam or fosinopril or guanabenz or guanethidine or guanfacine or hexamethonium or "hexamethonium compound*" or hydralazine or hydrochlorothiazide or hydroflumethiazide or indapamide or indoramin or irbesartan or isradipine or kallidin or ketanserin or labetalol or latanoprost or lisinopril or losartan or mecamylamine or methyldopa or metipranolol or metolazone or metoprolol or mibefradil or minoxidil or muzolimine or nadolol or nebivolol or nicardipine or nicorandil or nimodipine or nisoldipine or nitrendipine or nitroprusside or olmesartan medoxomil or oxprenolol or pargyline or pempidine or penbutolol or "pentolinium tartrate" or perindopril

or phenoxybenzamine or phentolamine or pinacidil or pindolol or piperoxan or polythiazide or prazosin or propranolol or protoveratrines or quinapril or ramipril or reserpine or rilmenidine or telmisartan or teprotide or terlipressin or ticrynafen or timolol or todralazine or tolazoline or torsemide or travoprost or trichlormethiazide or trimethaphan or valsartan or "veratrum alkaloid*" or vincamine or xipamide).tw.

47. or/33-46
48. cardiovascular.tw.
49. exp cardiovascular disorders/
50. ((cardiac or coronary or heart) adj3 (arrest* or attack* or disease* or failure*)).tw.
51. ((heart or myocard* or transient) adj3 (infarc* or ischaemi* or ischemi*)).tw.
52. angina*.tw.
53. Angina Pectoris/
54. stroke*.tw.
55. exp cerebral ischemia/
56. or/48-55
57. 15 and 47 and 56
58. ((effectiveness or implementation or literature or map or mapping or qualitative or rapid or realist or systematic or scoping or "state of the art" or umbrella) adj2 (assessment* or overview* or review* or synthes*)).tw.
59. ("meta analy*" or metaanaly* or metasynthe* or "meta synthe*").tw.
60. ((systematic or evidence) adj1 assess*).tw.
61. (qualitative adj2 (evidence or synthes*)).tw.
62. (overarching adj2 model).tw.
63. "review* of reviews".tw.
64. systematic review.pt.
65. meta-analysis.pt.
66. or/58-65
67. 32 and 66 [Drugs that cause dependency search results]
68. 57 and 66 [Statins search results]

Notes: Date limited 2010 to date of search.

Database: Conference Proceedings Citation Index – Science (CPCI-S); Science Citation Index (SCI)
Host: Web of Science via Clarivate Analytics
Issue: n/a
Date Searched: 11/8/2020

Searcher: SB
Hits: 1030
Strategy:

1. TOPIC =((appropriate* or discontinu* or enhance* or inappropriate* or incorrect* or "in correct*" or optim* or safe or suboptim* or "sub optim*" or tapering or withdrawal) near/3 (drug* or medicine* or medication* or prescri*))
2. TOPIC =((drug* or guideline* or guidance or medicine* or medication* or patient* or prescri*) near/3 (adhere* or compliance or concordance))
3. TOPIC =((shared or sharing or informed) near/1 (decision* or choice*))
4. TOPIC =((consumer* or patient*) near/2 (involv* or participat*))
5. TOPIC =("patient cent*" near/1 (approach* or care or decision* or intervention* or treatment*))
6. TOPIC =(decision near/1 (aid* or support*))
7. TOPIC =(behavi* near/1 chang*)
8. TOPIC =(restriction near/1 (policy or policies))
9. #8 OR #7 OR #6 OR #5 OR #4 OR #3 OR #2 OR #1
10. TOPIC: (benzodiazepine*)
11. TOPIC: ((alprazolam or flunitrazepam or chlordiazepoxide or clobazam or clonazepam or diazepam or lorazepam or midazolam or nitrazepam or oxazepam or prazepam or temazepam))
12. TOPIC: ((antidepres* or "anti depres*"))
13. TOPIC: ((serotonin or norepinephrine or noradrenaline or neurotransmitter* or dopamin* or SSRI* or SNRI* or NARI* or SARI* or NDRI* or tricyclic* or tetracyclic*))
14. TOPIC: ((opioid* or opiate*))
15. TOPIC: ((morphine or hydromorphone or levorphanol or meperidine or methadone or propoxyphene of codeine or pentazocine or hydrocodone or oxycodone or fentanyl or tramadol))
16. TOPIC: ("z drug*")
17. TOPIC: ((zopiclone or zolpidem or zaleplon or eszopiclone))
18. TOPIC: ((gabapentin* or mirogabalin or phenibut or pregabalin))
19. #18 OR #17 OR #16 OR #15 OR #14 OR #13 OR #12 OR #11 OR #10
20. #19 AND #9
21. TOPIC: (statin*)
22. TOPIC: ("HMG-CoA reductase inhibitor*" or "3-hydroxy-3-methyl-glutaryl-CoA reductase inhibitor*" or "3-hydroxy-3-methylglutaryl coenzyme A reductase inhibitor*" or "HMGCR inhibitor*" or "Hydroxymethylglutaryl-CoA Reductase Inhibitor*")

23. TOPIC: (atorvastatin or Lipitor or Torvast)
24. TOPIC: (fluvastatin or Lescol)
25. TOPIC: (lovastatin or Mevacor or Altocor or Altoprev)
26. TOPIC: (pitavastatin or Livalo or Pitava)
27. TOPIC: (pravastatin or Pravachol or Selektine or Lipostat)
28. TOPIC: (rosuvastatin or Crestor)
29. TOPIC: (simvastatin or Zocor or Lipex)
30. TOPIC: (antihypertensive*)
31. TOPIC: ("ACE inhibitor*" or antagonist or "angiotensin II receptor" or "beta blocker*" or "calcium channel blocker*" or "thiazide diuretic*")
32. TOPIC: (acebutolol or adrenomedullin or alprenolol or amlodipine or atenolol or bendroflumethiazide or bepridil or betaxolol or bethanidine or bimatoprost or bisoprolol or bosentan or "bretylium tosylate" or brimonidine tartrate or bupranolol or captopril or carteolol or carvedilol or celiprolol or chlorisondamine or chlorothiazide or chlorthalidone or cilazapril or clonidine or cromakalim or cyclopenthiazide or debrisoquin or diazoxide or dihydralazine or dihydroalprenolol or diltiazem or doxazosin or enalapril or enalaprilat or eplerenone or epoprostenol or felodipine or fenoldopam or fosinopril or guanabenz or guanethidine or guanfacine or hexamethonium or "hexamethonium compound*" or hydralazine or hydrochlorothiazide or hydroflumethiazide or indapamide or indoramin or irbesartan or isradipine or kallidin or ketanserin or labetalol or latanoprost or lisinopril or losartan or mecamylamine or methyldopa or metipranolol or metolazone or metoprolol or mibefradil or minoxidil or muzolimine or nadolol or nebivolol or nicardipine or nicorandil or nimodipine or nisoldipine or nitrendipine or nitroprusside or olmesartan medoxomil or oxprenolol or pargyline or pempidine or penbutolol or "pentolinium tartrate" or perindopril or phenoxybenzamine or phentolamine or pinacidil or pindolol or piperoxan or polythiazide or prazosin or propranolol or protoveratrines or quinapril or ramipril or reserpine or rilmenidine or telmisartan or teprotide or terlipressin or ticrynafen or timolol or todralazine or tolazoline or torsemide or travoprost or trichlormethiazide or trimethaphan or valsartan or "veratrum alkaloid*" or vincamine or xipamide)
33. #32 OR #31 OR #30 OR #29 OR #28 OR #27 OR #26 OR #25 OR #24 OR #23 OR #22 OR #21
34. TOPIC =((cardiac or coronary or heart) near/2 (arrest* or attack* or disease* or failure*))
35. TOPIC: (cardiovascular)
36. TS=((heart or myocard* or transient) near/2 (infarc* or ischaemi* or ischemi*))
37. TOPIC =angina*
38. TOPIC =stroke*
39. #38 OR #37 OR #36 OR #35 OR #34

Shaw L, *et al. BMJ Open* 2024; 14:e072502. doi: 10.1136/bmjopen-2023-072502

40. TOPIC =((effectiveness or implementation or literature or map or mapping or qualitative or rapid or realist or systematic or scoping or "state of the art" or umbrella) near/1 (assessment* or overview* or review* or synthes*))
41. TOPIC =("meta analy*" or metaanaly* or metasynthe* or "meta synthe*")
42. TOPIC =((systematic or evidence) near/0 assess*)
43. TOPIC =(qualitative near/1 (evidence or synthes*))
44. TOPIC =(overarching near/1 model)
45. TOPIC ="review* of reviews"
46. #45 OR #44 OR #43 OR #42 OR #41 OR #40
47. #46 AND #20 [Drugs that cause dependency search results]
48. #46 AND #39 AND #33 AND #9 [Statins search results]

Table 1: Drugs that can cause dependency and antidepressant search results

Database	Hits
**Cochrane Database of Systematic Reviews**	111
**CINAHL**	372
**Epistemonikos**	56
**medRxiv**	87
**Embase**	1776
**HMIC**	10
**MEDLINE**	875
**PsycInfo**	297
**Web of Science (CPCI-S/SCI)**	1030
**TOTAL RECORDS**	**4614**
**DUPLICATE RECORDS**	**1898**
**UNIQUE RECORDS**	**2716**

Statins

Database: Cochrane Database of Systematic Reviews
Host: Cochrane Library
Issue: Issue 8 of 12, August 2020
Date Searched: 10/8/2020
Searcher: SB
Hits: 33
Strategy: see line 57 of Cochrane Database search above.
Notes: date limited 2010 to date of search.

Database: CINAHL
Host: EBSCO
Issue: n/a
Date Searched: 10/8/2020
Searcher: SB

Shaw L, *et al. BMJ Open* 2024; 14:e072502. doi: 10.1136/bmjopen-2023-072502

Hits: 173
Strategy: see line 66 of CINAHL search above.
Notes: Date limited 2010 to date of search.

Database: Epistemonikos
Host: https://www.epistemonikos.org/en/
Issue: n/a
Date Searched: 10/8/2020
Searcher: SB
Hits: 16
Strategy:

1. title:(prescription* OR prescribing OR prescribe*) OR abstract:(prescription* OR prescribing OR prescribe*)
2. title:(optimi* OR inappropriat* OR enhance*) OR abstract:(optimi* OR inappropriat* OR enhance*)
3. title:(statin*) OR abstract:(statin*) OR title:(antihypertensive*) OR abstract:(antihypertensive*)
4. 1 AND 2 AND 3 [Statins search results]

Notes: Date limited 2010 to 2020 and limited to systematic reviews publication type.

Database: medRxiv
Host: https://www.medrxiv.org/
Issue: n/a
Date Searched: 10/8/2020
Searcher: SB
Hits: 8
Strategy:

optimi* and prescri* and statin*	7 hits
optimi* and prescri* and antihypertensive*	1 hit

Notes: Date limited 2010 to date of search.

Database: Embase
Host: Ovid
Issue: 1974 to 2020 August 07
Date Searched: 10/8/2020
Searcher: SB
Hits: 903
Strategy: see line 69 of Embase search above.
Notes: Date limited 2010 to date of search.

Database: HMIC
Host: Ovid
Issue: 1979 to May 2020
Date Searched: 10/8/2020

Searcher: SB
Hits: 0
Strategy: see line 68 of MEDLINE search strategy above.
Notes: Date limited 2010 to date of search.

Database: MEDLINE ALL
Host: Ovid
Issue: 1946 to August 07, 2020
Date Searched: 10/8/2020
Searcher: SB
Hits: 371
Strategy: see line 68 of MEDLINE search strategy above.
Notes: Date limited 2010 to date of search.

Database: PsycInfo
Host: Ovid
Issue: 1806 to August Week 1 2020
Date Searched: 10/8/2020
Searcher: SB
Hits: 17
Strategy: see line 68 of PsycInfo search strategy above.
Notes: Date limited 2010 to date of search.

Database: Conference Proceedings Citation Index – Science (CPCI-S); Science Citation Index (SCI)
Host: Web of Science via Clarivate Analytics
Issue: n/a
Date Searched: 10/8/2020
Searcher: SB
Hits: 638
Strategy: see line 48 of CPCI-S/SCI search strategy above.

Table 2: Statin search results

Database	Hits
**Cochrane Database of Systematic Reviews**	33
**CINAHL**	173
**Epistemonikos**	16
**medRxiv**	8
**EMBASE**	903
**HMIC**	0
**MEDLINE**	371
**PsycInfo**	17
**Web of Science (CPCI-S/SCI)**	638
**TOTAL RECORDS**	**2159**
**DUPLICATE RECORDS**	**714**
**UNIQUE RECORDS**	**1445**

Website searches

DCDs

Website: Royal Pharmaceutical Society Library Catalogue
URL: https://rpsgb.koha-ptfs.co.uk/
Date Searched: 21/10/2020
Searcher: SB
Hits: 73 hits
Strategy:

Advanced search setting.

Keyword: opioid* OR

Keyword: benzodiazepine* OR

Keyword: antidepressant* OR

Keyword: gabapentinoid*

Website: Royal College of Physicians Library Catalogue
URL: https://rcp.soutron.net/Portal/Default/en-GB/Search/SimpleSearch
Date Searched: 21/10/2020
Searcher: SB
Hits: 6
Strategy:

All fields: opioid OR opioids OR benzodiazepine OR benzodiazepines OR antidepressant OR antidepressants OR gabapentinoid OR gabapentinoids

Website: Royal College of Psychiatrists Library Catalogue
URL: https://rcpsych.soutron.net/Portal/Default/en-GB/Search/SimpleSearch
Date Searched: 21/10/2020
Searcher: SB
Hits: 20
Strategy:

opioid* OR benzodiazepine* OR antidepressant* OR gabapentinoid*

Website: Mind
URL: https://www.mind.org.uk/
Date Searched: 21/10/2020
Searcher: SB
Hits: 0
Strategy: Browsed website for relevant studies

Website: Mental Health UK
URL: https://mentalhealth-uk.org/

Date Searched: 21/10/2020
Searcher: SB
Hits: 0
Strategy: Browsed website for relevant studies

Website: Priory Group
URL: https://www.priorygroup.com/
Date Searched: 21/10/2020
Searcher: SB
Hits: 0
Strategy: Browsed website for relevant studies

Statins

Website: Royal Pharmaceutical Society Library Catalogue
URL: https://rpsgb.koha-ptfs.co.uk/
Date Searched: 21/10/2020
Searcher: SB
Hits: 13
Strategy:

Advanced search setting.

Keyword:	statin*	OR
Keyword:	“ace inhibitor*”	OR
Keyword:	antihypertensive*	

Website: Royal College of Physicians Library Catalogue
URL: https://rcp.soutron.net/Portal/Default/en-GB/Search/SimpleSearch
Date Searched: 21/10/2020
Searcher: SB
Hits: 6
Strategy:

All fields: statin OR statins OR "ace inhibitor" OR "ace inhibitors" OR antihypertensive OR antihypertensives

Website: British Cardiovascular Society
URL: https://www.britishcardiovascularsociety.org/
Date Searched: 21/10/2020
Searcher: SB
Hits: 0
Strategy: Browsed website for relevant studies.

Shaw L, *et al. BMJ Open* 2024; 14:e072502. doi: 10.1136/bmjopen-2023-072502

Website: European Society of Cardiology
URL: https://www.escardio.org/
Date Searched: 21/10/2020
Searcher: SB
Hits: 0
Strategy: Browsed website for relevant studies

Website: British and Irish Hypertension Society
URL: https://bihsoc.org/
Date Searched: 21/10/2020
Searcher: SB
Hits: 0
Strategy: Browsed website for relevant studies

Website: British Heart Foundation
URL: https://www.bhf.org.uk/
Date Searched: 21/10/2020
Searcher: SB
Hits: 31 (sum total of each keyword search)
Strategy: Searched for keywords “statin”, “ace inhibitor” or “antihypertensive” (one term per search) and viewed the publications and research tabs.

Website: Heart Research UK
URL: https://heartresearch.org.uk/
Date Searched: 21/10/2020
Searcher: SB
Hits: 0
Strategy: Browsed website for relevant studies

APPENDIX B: REASONS FOR EXCLUSION AT FULL TEXT

Reference	Reason for exclusion
Abstracts from Professional Poster Presentations at AMCP's 2010 Educational Conference. *Journal of Managed Care Pharmacy* 2010;**16**.	Abstract proceedings
The Canadian Association for Population Therapeutics. November 17th - 19th, 2013 Toronto, Ontario. "Healthcare Cost, Quality, and Policy: Driving Stakeholder Innovation in Process and Practice". *Journal of Population Therapeutics and Clinical Pharmacology* 2014;**21**:e116.	Abstract proceedings
Transcatheter Cardiovascular Therapeutics Asia Pacific, 19th Cardiovascular Summit: TCTAP 2014. *Journal of the American College of Cardiology* 2014;**63**.	Abstract proceedings
Abstracts of Papers Presented at the Health Services Research and Pharmacy Practice Conference, HSRPP 2016. *International Journal of Pharmacy Practice* 2016;**24**.	Abstract proceedings
12th Annual Scientific Meeting of Indonesian Society of Hypertension, INASH. *Journal of Hypertension* 2018;**36**.	Abstract proceedings
Selected abstracts from the EGPRN meeting in Tampere, Finland, 9–12 May 2019. *European Journal of General Practice* 2019;**25**:164-75.	Abstract proceedings
46th ESAO Congress 3-7 September 2019 Hannover, Germany Abstracts. *International Journal of Artificial Organs* 2019;**42**:386-474.	Abstract proceedings
AbuDagga A, Resnick HE, Alwan M. Impact of blood pressure telemonitoring on hypertension outcomes: a literature review. *Telemed J E Health* 2010;**16**:830-8. https://doi.org/10.1089/tmj.2010.0015	Not SR
Akeroyd JM, Chan WJ, Kamal AK, Palaniappan L, Virani SS. Adherence to cardiovascular medications in the South Asian population: A systematic review of current evidence and future directions. *World J Cardiol* 2015;**7**:938-47. https://doi.org/10.4330/wjc.v7.i12.938	Not SR
Al AlShaikh S, Quinn T, Dunn W, Dawson J. Interventions to Enhance Adherence to Secondary Preventative Medication After Stroke a Systematic Review and Meta-Analysis. *Stroke* 2016;**47**.	Abstract
Alford DP. Chronic back pain with possible prescription opioid misuse. *JAMA* 2013;**309**:919-25. https://doi.org/10.1001/jama.2013.522	Not SR
AlGhurair SA, Hughes CA, Simpson SH, Guirguis LM. A systematic review of patient self-reported barriers of adherence to antihypertensive medications using the world health organization multidimensional adherence model. *J Clin Hypertens (Greenwich)* 2012;**14**:877-86. https://doi.org/10.1111/j.1751-7176.2012.00699.x	Not SR

Alkhouli M, Noseworthy PA, Rihal CS, Holmes DR, Jr. Stroke Prevention in Nonvalvular Atrial Fibrillation: A Stakeholder Perspective. *J Am Coll Cardiol* 2018;**71**:2790-801. https://doi.org/10.1016/j.jacc.2018.04.013	Not SR
Almeida JT, Esteves AL, Martins F, Palma I. [Approach to Patients with Statin Intolerance: Evidence-Based Review]. *Acta Med Port* 2020;**33**:49-57. https://doi.org/10.20344/amp.10376	Non-English language (Duplicated excluded from update search)
AlShaikh SA, Quinn T, Dunn W, Dawson J. Interventions to enhance adherence to secondary preventative medication after stroke a systematic review and meta-analysis. *Stroke* 2016;**47**.	Duplicate
Anderson SL, Marrs JC. A Review of the Role of the Pharmacist in Heart Failure Transition of Care. *Adv Ther* 2018;**35**:311-23. https://doi.org/10.1007/s12325-018-0671-7	Not SR
Anonymous. Review of: Fuller RH et al. Improving adherence to medicines in people with heart disease: a systematic review. Heart. *Drug Ther Bull* 2018;**56**:114. https://doi.org/10.1136/dtb.2018.10.000021	Not SR
Armstrong SO, Little RA. Cost effectiveness of interventions to improve adherence to statin therapy in ASCVD patients in the United States. *Patient Prefer Adherence* 2019;**13**:1375-89. https://doi.org/10.2147/PPA.S213258	Not SR
Banerjee A, Khandelwal S, Nambiar L, Saxena M, Peck V, Moniruzzaman M*, et al.* Health system barriers and facilitators to medication adherence for the secondary prevention of cardiovascular disease: a systematic review. *Open Heart* 2016;**3**:e000438. https://doi.org/10.1136/openhrt-2016-000438	Quantitative focus
Basaraba JE, Picard M, George-Phillips K, Mysak T. Pharmacists as Care Providers for Stroke Patients: A Systematic Review. *Can J Neurol Sci* 2018;**45**:49-55. https://doi.org/10.1017/cjn.2017.233	Not SR
Bramlage P, Sims H, Minguet J, Ferrero C. The polypill: An effective approach to increasing adherence and reducing cardiovascular event risk. *Eur J Prev Cardiol* 2017;**24**:297-310. https://doi.org/10.1177/2047487316674817	Not SR
Brito JP, Montori VM. Reinitiation of statins after statin-associated musculoskeletal symptoms: a patient-centered approach. *Circ Cardiovasc Qual Outcomes* 2013;**6**:243-7. https://doi.org/10.1161/CIRCOUTCOMES.111.000039	Not SR
Buck HG, McGhee S, Polo RL, Zambroski C. Hypercholesterolemia Management in Older Adults: A Scoping Review of Recent Evidence. *J Gerontol Nurs* 2019;**45**:31-42. https://doi.org/10.3928/00989134-20190211-04	Quantitative focus

Calleja Rivero JL, Zerpa F, Rivera L. Fixed-dose versus separate drug combinations for antihypertensive treatment: literature review. *Medwave* 2016;**16**:e6549. https://doi.org/10.5867/medwave.2016.08.6549	Not SR
Campos K, Sheth S, Coulter SA. Hypertension Treatment ACCORDing to SPRINT. *Tex Heart Inst J* 2016;**43**:324-7. https://doi.org/10.14503/THIJ-16-5908	Not SR
Chan DK, O'Rourke F, Shen Q, Mak JC, Hung WT. Meta-analysis of the cardiovascular benefits of intensive lipid lowering with statins. *Acta Neurol Scand* 2011;**124**:188-95. https://doi.org/10.1111/j.1600-0404.2010.01450.x	Quantitative focus
Chapman RH, Ferrufino CP, Kowal SL, Classi P, Roberts CS. The cost and effectiveness of adherence-improving interventions for antihypertensive and lipid-lowering drugs*. *Int J Clin Pract* 2010;**64**:169-81. https://doi.org/10.1111/j.1742-1241.2009.02196.x	Not SR
Chee YJ, Chan HH, Tan NC. Understanding patients' perspective of statin therapy: can we design a better approach to the management of dyslipidaemia? A literature review. *Singapore Med J* 2014;**55**:416-21. https://doi.org/10.11622/smedj.2014099	Not SR
Choi H-I, Han KH. The ideal management of blood cholesterol: a review of the updated 2013 ACC/AHA cholesterol guideline for reducing cardiovascular risk. *Journal of the Korean Medical Association* 2014;**57**:857-65. https://doi.org/10.5124/jkma.2014.57.10.857	Non-English language
Clarkesmith DE, Pattison HM, Khaing PH, Lane DA. Educational and behavioural interventions for anticoagulant therapy in patients with atrial fibrillation. *Cochrane Database of Systematic Reviews* 2017; 10.1002/14651858.CD008600.pub3. https://doi.org/10.1002/14651858.CD008600.pub3	Drug not relevant
Coleman CI, Roberts MS, Sobieraj DM, Lee S, Alam T, Kaur R. Effect of dosing frequency on chronic cardiovascular disease medication adherence. *Curr Med Res Opin* 2012;**28**:669-80. https://doi.org/10.1185/03007995.2012.677419	Not SR
Conn VS, Ruppar TM, Chase JD. Blood pressure outcomes of medication adherence interventions: systematic review and meta-analysis. *J Behav Med* 2016;**39**:1065-75. https://doi.org/10.1007/s10865-016-9730-1	Quantitative focus
Cooke CE, Xing S, Gale SE, Peters S. Initial non-adherence to antihypertensive medications in the United States: a systematic literature review. Journal of human hypertension 2022;36:3-13. https://doi.org/https://dx.doi.org/10.1038/s41371-021-00549-w	Not SR
Cooke CE, Xing S, Gale SE, Peters S. Initial non-adherence to antihypertensive medications in the United States: a systematic literature review. J Hum Hypertens. 2022 Jan;36(1):3-13. doi: 10.1038/s41371-021-00549-w. Epub 2021 May 14. PMID: 33990698.	Duplicate

Crisafulli S, Luxi N, Coppini R, Capuano A, Scavone C, Zinzi A, et al. Anti-hypertensive drugs deprescribing: an updated systematic review of clinical trials. BMC family practice 2021;22:208. https://doi.org/https://dx.doi.org/10.1186/s12875-021-01557-y	Not SR
Cutrona SL, Choudhry NK, Fischer MA, Servi A, Liberman JN, Brennan TA*, et al.* Modes of delivery for interventions to improve cardiovascular medication adherence. *Am J Manag Care* 2010;**16**:929-42.	Not SR
de Barra M, Scott CL, Scott NW, Johnston M, de Bruin M, Nkansah N*, et al.* Pharmacist services for non-hospitalised patients. *Cochrane Database Syst Rev* 2018;**9**:CD013102. https://doi.org/10.1002/14651858.CD013102	Quantitative focus
Dobremez V, Ruel JH, Hugon A, Gay S, Berlioz J, Pineau-Blondel E. Contraindicated drugs in myasthenia gravis patients: Elaboration of a tool to optimize drug therapy. *International Journal of Clinical Pharmacy* 2015;**37**:236-7. https://doi.org/10.1007/s11096-014-0039-2	Abstract
Dunn BL, Teusink AC, Taber DJ, Hemstreet BA, Uber LA, Weimert NA. Management of hypertension in renal transplant patients: a comprehensive review of nonpharmacologic and pharmacologic treatment strategies. *Ann Pharmacother* 2010;**44**:1259-70. https://doi.org/10.1345/aph.1P004	Not SR
Elley CR, Gupta AK, Webster R, Selak V, Jun M, Patel A*, et al.* The efficacy and tolerability of 'polypills': meta-analysis of randomised controlled trials. *PLoS One* 2012;**7**:e52145. https://doi.org/10.1371/journal.pone.0052145	Quantitative focus
Elnaem MH, Rosley NFF, Alhifany AA, Elrggal ME, Cheema E. Impact of Pharmacist-Led Interventions on Medication Adherence and Clinical Outcomes in Patients with Hypertension and Hyperlipidemia: A Scoping Review of Published Literature. *J Multidiscip Healthc* 2020;**13**:635-45. https://doi.org/10.2147/JMDH.S257273	Not SR
Engel J, Damen NL, van der Wulp I, de Bruijne MC, Wagner C. Adherence to Cardiac Practice Guidelines in the Management of Non-ST-Elevation Acute Coronary Syndromes: A Systematic Literature Review. *Curr Cardiol Rev* 2017;**13**:3-27. https://doi.org/10.2174/1573403x12666160504100025	Quantitative focus
Gale N, Marshall T, Bramley G. Starting and staying on preventive medication for cardiovascular disease. *Curr Opin Cardiol* 2012;**27**:533-41. https://doi.org/10.1097/HCO.0b013e328356dae5	Not SR
Gandapur Y, Kianoush S, Kelli HM, Misra S, Urrea B, Blaha MJ*, et al.* The role of mHealth for improving medication adherence in patients with cardiovascular disease: a systematic review. *Eur Heart J Qual Care Clin Outcomes* 2016;**2**:237-44. https://doi.org/10.1093/ehjqcco/qcw018	Not SR

Gellad WF, Grenard JL, Marcum ZA. A systematic review of barriers to medication adherence in the elderly: looking beyond cost and regimen complexity. *Am J Geriatr Pharmacother* 2011;**9**:11-23. https://doi.org/10.1016/j.amjopharm.2011.02.004	Quantitative focus
Georgiopoulos G, Kollia Z, Katsi V, Oikonomou D, Tsioufis C, Tousoulis D. Nurse's Contribution to Alleviate Non-adherence to Hypertension Treatment. *Curr Hypertens Rep* 2018;**20**:65. https://doi.org/10.1007/s11906-018-0862-2	Not SR
Glynn L, Fahey T. Cardiovascular medication: improving adherence. *BMJ Clin Evid* 2011;**2011**.	Not SR
Golubev S. Compliance measurement-guided medication management programs in hypertension: A health technology assessment. *Journal of Hypertension* 2010;**28**:e269.	Quantitative focus
Golubev S, Fedoseyeva I, Johri M. Compliance measurement-guided medication management programs in hypertension: A systematic review. *Basic and Clinical Pharmacology and Toxicology* 2011;**109**:109-10. http://dx.doi.org/10.1111/j.1742-7843.2011.00722.x	Abstract
Gwadry-Sridhar FH, Manias E, Lal L, Salas M, Hughes DA, Ratzki-Leewing A*, et al.* Impact of interventions on medication adherence and blood pressure control in patients with essential hypertension: a systematic review by the ISPOR medication adherence and persistence special interest group. *Value Health* 2013;**16**:863-71. https://doi.org/10.1016/j.jval.2013.03.1631	Not SR
Hong J, Chak Tiu Y, Leung PYB, Wong MF, Ng WY, Cheung D, Yan Mok H, Lam WY, Li KY, Wong CKH, Interventions that improve adherence to antihypertensive medications in coronary heart disease patients: a systematic review, *Postgraduate Medical Journal*, Volume 98, Issue 1157, March 2022, Pages 219–227	Duplicate
Hopper I, Samuel R, Tonkin A, Krum H. The effects of discontinuing medications in stable systolic heart failure-systematic review and meta-analysis. *Heart Lung and Circulation* 2012;**21**:S95. http://dx.doi.org/10.1016/j.hlc.2012.05.241	Abstract
Imbeault B, Vallee M. Single-Pill Combinations in the Treatment of Hypertension in Adults: Beyond Convenience. *Can J Diabetes* 2018;**42**:205-8. https://doi.org/10.1016/j.jcjd.2018.01.011	Not SR
Iwelunmor J, Airhihenbuwa CO, Cooper R, Tayo B, Plange-Rhule J, Adanu R*, et al.* Prevalence, determinants and systems-thinking approaches to optimal hypertension control in West Africa. *Global Health* 2014;**10**:42. https://doi.org/10.1186/1744-8603-10-42	Not SR
Jamshidnezhad A, Kabootarizadeh L, Hoseini SM. The Effects of Smartphone Applications on Patients Self-care with Hypertension: A Systematic Review Study. *Acta Inform Med* 2019;**27**:263-7. https://doi.org/10.5455/aim.2019.27.263-267	Not SR

Jones LK, Gionfriddo MR, Tilberry S, Frisbie L, Gregor C, Gidding S, *et al.* Implementation strategies to improve statin utilization in individuals with hypercholesterolemia: A systematic review. *JACCP Journal of the American College of Clinical Pharmacy* 2020;**3**:365. http://dx.doi.org/10.1002/jac5.1204	Abstract
Jornten-Karlsson M, Pintat S, Molloy-Bland M, Berg S, Ahlqvist M. Patient-Centered Interventions to Improve Adherence to Statins: A Narrative Synthesis of Systematically Identified Studies. *Drugs* 2016;**76**:1447-65. https://doi.org/10.1007/s40265-016-0640-x	Not SR
Ju A, Hanson CS, Banks E, Korda R, Craig JC, Usherwood T, *et al.* Patient beliefs and attitudes to taking statins: systematic review of qualitative studies. *Br J Gen Pract* 2018;**68**:e408-e19. https://doi.org/10.3399/bjgp18X696365	Not SR
Ko DT, Jackevicius CA. Stopping beta-Blockers After Myocardial Infarction: Not So Fast! *Circ Cardiovasc Qual Outcomes* 2018;**11**:e004678. https://doi.org/10.1161/CIRCOUTCOMES.118.004678	Not SR
Krack G. EXPLANATIONS FOR THE DIFFERENCES BETWEEN EFFECTS ON ADHERENCE OF VALUE-BASED HEALTH INSURANCE DESIGNS: A SYSTEMATIC REVIEW AND META-ANALYSIS. *Value in Health* 2018;**21**:S155. http://dx.doi.org/10.1016/j.jval.2018.09.924	Abstract
Krueger K, Griese-Mammen N, Schubert I, Kieble M, Botermann L, Laufs U, *et al.* In search of a standard when analyzing medication adherence in patients with heart failure using claims data: a systematic review. *Heart Fail Rev* 2018;**23**:63-71. https://doi.org/10.1007/s10741-017-9656-x	Quantitative focus
Kwon A, Kim GH. Single-pill Combination Therapy of Azilsartan Medoxomil/Chlorthalidone for Treatment of Hypertension: A Systematic Review. *Clin Ther* 2020;**42**:1390-403. https://doi.org/10.1016/j.clinthera.2020.05.015	Not SR (Duplicate excluded in update search)
Lane D, Patel P, Khunti K, Gupta P. Objective measures of non-adherence in cardiometabolic diseases: a review focused on urine biochemical screening. *Patient Prefer Adherence* 2019;**13**:537-47. https://doi.org/10.2147/PPA.S162215	Not SR
Lee JL, Maciejewski M, Raju S, Shrank WH, Choudhry NK. Value-based insurance design: quality improvement but no cost savings. *Health Aff (Millwood)* 2013;**32**:1251-7. https://doi.org/10.1377/hlthaff.2012.0902	Not SR
Lennon O, Blake C, Booth J, Pollock A, Lawrence M. Interventions for behaviour change and self-management in stroke secondary prevention: protocol for an overview of reviews. *Syst Rev* 2018;**7**:231. https://doi.org/10.1186/s13643-018-0888-1	Protocol

Liu HL, Chen XY, Li JR, Su SW, Ding T, Shi CX*, et al.* Efficacy and Safety of Pulmonary Arterial Hypertension-specific Therapy in Pulmonary Arterial Hypertension: A Meta-analysis of Randomized Controlled Trials. *Chest* 2016;**150**:353-66. https://doi.org/10.1016/j.chest.2016.03.031	Quantitative focus
Logue J, Al-Ghibiwi H, Alamri AA, Preiss D. Systematic review of studies exploring reasons for statin non-adherence and of randomised controlled trials of interventions to improve adherence. *Atherosclerosis* 2015;**241**:e52.	Abstract
Madhavan MV, Gersh BJ, Alexander KP, Granger CB, Stone GW. Coronary Artery Disease in Patients >/=80 Years of Age. *J Am Coll Cardiol* 2018;**71**:2015-40. https://doi.org/10.1016/j.jacc.2017.12.068	Not SR
Marcum ZA, Handler SM, Boyce R, Gellad W, Hanlon JT. Medication misadventures in the elderly: a year in review. *Am J Geriatr Pharmacother* 2010;**8**:77-83. https://doi.org/10.1016/j.amjopharm.2010.02.002	Not SR
Matthes J, Albus C. Improving adherence with medication: a selective literature review based on the example of hypertension treatment. *Dtsch Arztebl Int* 2014;**111**:41-7. https://doi.org/10.3238/arztebl.2014.0041	Not SR
Mendes Soares M, Oliveira LeÆo e Silva L, Alberto Dias C, Maria Rodrigues S, Jorge Machado C. ADESAO DO IDOSO AO TRATAMENTO DA HIPERTENSAO ARTERIAL SISTEMICA: REVISAO INTEGRATIVA. *Cogitare Enfermagem* 2012;**17**:114-50.	Non-English language
Meza-Contreras A, Wenczenovicz C, Ruiz-Arellanos K, Vesely EAK, Mogollon R, Montori VM. Statin intolerance management: a systematic review. Endocrine. 2023 Mar;79(3):430-436. doi: 10.1007/s12020-022-03263-w. Epub 2022 Dec 2. PMID: 36459335.	Not SR
Molloy GJ, O'Carroll RE, Witham MD, McMurdo ME. Interventions to enhance adherence to medications in patients with heart failure: a systematic review. *Circ Heart Fail* 2012;**5**:126-33. https://doi.org/10.1161/CIRCHEARTFAILURE.111.964569	Not SR
Morgado M, Castanheira L, Verde I, Castelo-Branco M. Pharmacist interventions to enhance patient adherence to antihypertensive medication: A systematic review. *Journal of Clinical Hypertension* 2010;**12**:A112-A3. http://dx.doi.org/10.1111/j.1751-7176.2010.00282.x	Abstract
Morgado MP, Morgado SR, Mendes LC, Pereira LJ, Castelo-Branco M. Pharmacist interventions to enhance blood pressure control and adherence to antihypertensive therapy: Review and meta-analysis. *Am J Health Syst Pharm* 2011;**68**:241-53. https://doi.org/10.2146/ajhp090656	Not SR

Morrissey EC, Durand H, Nieuwlaat R, Navarro T, Haynes RB, Walsh JC*, et al.* Effectiveness and content analysis of interventions to enhance medication adherence in hypertension: a systematic review and meta-analysis protocol. *Syst Rev* 2016;**5**:96. https://doi.org/10.1186/s13643-016-0278-5	Protocol
Na. Selected Abstracts from the National Congress of the Italian Society of Hypertension (SIIA 2010). *High Blood Pressure & Cardiovascular Prevention* 2010;**17**:131-90. https://doi.org/10.2165/11311900-000000000-00000	Quantitative focus
Naderi SH, Bestwick JP, Wald DS. Adherence to drugs that prevent cardiovascular disease: meta-analysis on 376,162 patients. *Am J Med* 2012;**125**:882-7 e1. https://doi.org/10.1016/j.amjmed.2011.12.013	Not SR
Nguyen E, Sobieraj DM. The impact of appointment-based medication synchronization on medication taking behaviour and health outcomes: A systematic review. *J Clin Pharm Ther* 2017;**42**:404-13. https://doi.org/10.1111/jcpt.12554	Not SR
Nguyen T, Gurwitz J. Paper Abstract. *Journal of the American Geriatrics Society* 2016;**64**:S1-S311. https://doi.org/10.1111/jgs.14231	Quantitative focus
Ni Z, Dardas L, Wu B, Shaw R. Cardioprotective medication adherence among patients with coronary heart disease in China: a systematic review. *Heart Asia* 2019;**11**:e011173. https://doi.org/10.1136/heartasia-2018-011173	Not SR
Nielsen JO, Shrestha AD, Neupane D, Kallestrup P. Non-adherence to anti-hypertensive medication in low- and middle-income countries: a systematic review and meta-analysis of 92443 subjects. *J Hum Hypertens* 2017;**31**:14-21. https://doi.org/10.1038/jhh.2016.31	Quantitative focus
Nieuwlaat R, Connolly SJ, MacKay JA, Weise-Kelly L, Navarro T, Wilczynski NL*, et al.* Computerized clinical decision support systems for therapeutic drug monitoring and dosing: A decision maker-researcher partnership systematic review. *Circulation: Cardiovascular Quality and Outcomes* 2011;**4**.	Drug not relevant
Njie GJ, Mukhtar Q, Finnie RKC, Dhakal-Acharya S, Thota AB, Hopkins DP*, et al.* Effectiveness of reducing out-of-pocket costs for medications to treat hypertension and hyperlipidemia: A community guide systematic review. *Journal of the American Society of Hypertension* 2014;**8**:e124-e5. http://dx.doi.org/10.1016/j.jash.2014.03.285	Abstract
Ofori-Asenso R, Zoungas S, Liew D. Reinitiation of Statin Therapy After Discontinuation: A Meta-analysis. *Mayo Clin Proc* 2018;**93**:666-8. https://doi.org/10.1016/j.mayocp.2018.01.011	Quantitative focus
Oosterom-Calo R, van Ballegooijen AJ, Terwee CB, te Velde SJ, Brouwer IA, Jaarsma T*, et al.* Determinants of adherence to heart failure medication: a systematic literature review. *Heart Fail Rev* 2013;**18**:409-27. https://doi.org/10.1007/s10741-012-9321-3	Quantitative focus

Osborn D, Burton A, Walters K, Atkins L, Barnes T, Blackburn R*, et al. Primary care management of cardiovascular risk for people with severe mental illnesses: the Primrose research programme including cluster RCT*. Southampton (UK): NIHR Journals Library; 2019 Apr. http://doi.org/ 10.3310/pgfar07020	Quantitative focus
Pena RCF, Hofmann Bowman MA, Ahmad M, Pham J, Kline-Rogers E, Case MJ, Lee J, Eagle K; Aortic Dissection Collaborative. An assessment of the current medical management of thoracic aortic disease: A patient-centered scoping literature review. Semin Vasc Surg. 2022 Mar;35(1):16-34. doi: 10.1053/j.semvascsurg.2022.02.007. Epub 2022 Feb 18. PMID: 35501038.	Not SR
Perera R, McFadden E, McLellan J, Lung T, Clarke P, Perez T*, et al.* Optimal strategies for monitoring lipid levels in patients at risk or with cardiovascular disease: a systematic review with statistical and cost-effectiveness modelling. *Health Technol Assess* 2015;**19**:1-401, vii-viii. https://doi.org/10.3310/hta191000	Quantitative focus
Phan K, Gomez YH, Elbaz L, Daskalopoulou SS. Statin treatment non-adherence and discontinuation: clinical implications and potential solutions. *Curr Pharm Des* 2014;**20**:6314-24. https://doi.org/10.2174/1381612820666140620162629	Quantitative focus
Pinho S, Cruz M, Ferreira F, Ramalho A, Sampaio R. Improving medication adherence in hypertensive patients: A scoping review. Preventive medicine 2021;146:106467. https://doi.org/https://dx.doi.org/10.1016/j.ypmed.2021.106467	Not SR
Polinski JM, Donohue JM, Kilabuk E, Shrank WH. Medicare Part D's effect on the under- and overuse of medications: a systematic review. *J Am Geriatr Soc* 2011;**59**:1922-33. https://doi.org/10.1111/j.1532-5415.2011.03537.x	Quantitative focus
Powers BJ, Coeytaux RR, Dolor RJ, Hasselblad V, Patel UD, Yancy WS, Jr.*, et al.* Updated report on comparative effectiveness of ACE inhibitors, ARBs, and direct renin inhibitors for patients with essential hypertension: much more data, little new information. *J Gen Intern Med* 2012;**27**:716-29. https://doi.org/10.1007/s11606-011-1938-8	Quantitative focus
Rabien A, Price M. Development of a clinical decision support tool for the primary prevention of cardiovascular disease. *Canadian Family Physician* 2015;**61**:S2.	Abstract
Rahmawati R, Bajorek BV. Self-medication among people living with hypertension: a review. *Fam Pract* 2017;**34**:147-53. https://doi.org/10.1093/fampra/cmw137	Not SR
Reeve E, Jordan V, Thompson W, Sawan M, Todd A, Gammie TM*, et al.* Withdrawal of antihypertensive drugs in older people. *Cochrane Database Syst Rev* 2020;**6**:CD012572. https://doi.org/10.1002/14651858.CD012572.pub2	Quantitative focus

Ruppar TM, Dunbar-Jacob JM, Mehr DR, Lewis L, Conn VS. Medication adherence interventions among hypertensive black adults: a systematic review and meta-analysis. *J Hypertens* 2017;**35**:1145-54. https://doi.org/10.1097/HJH.0000000000001260	Full text not retrieved
Schedlbauer A, Davies P, Fahey T. Interventions to improve adherence to lipid lowering medication. *Cochrane Database Syst Rev* 2010; 10.1002/14651858.CD004371.pub3:CD004371. https://doi.org/10.1002/14651858.CD004371.pub3	Not latest version
Scranton RE, Goldstein I, Stecher VJ. Erectile dysfunction diagnosis and treatment as a means to improve medication adherence and optimize comorbidity management. *J Sex Med* 2013;**10**:551-61. https://doi.org/10.1111/j.1743-6109.2012.02998.x	Not SR
Seedat Y. Fixed drug combination in the treatment of hypertension and hyperlipidemia in the developing world. *Circulation* 2010;**122**:e94. http://dx.doi.org/10.1161/CIRCULATIONAHA.110.192773	Abstract
Selak V, Webster R, Stepien S, Bullen C, Patel A, Thom S*, et al.* Reaching cardiovascular prevention guideline targets with a polypill-based approach: a meta-analysis of randomised clinical trials. *Heart* 2019;**105**:42-8. https://doi.org/10.1136/heartjnl-2018-313108	Not SR
Shahaj O, Denneny D, Schwappach A, Pearce G, Epiphaniou E, Parke HL*, et al.* Supporting self-management for people with hypertension: a meta-review of quantitative and qualitative systematic reviews. *J Hypertens* 2019;**37**:264-79. https://doi.org/10.1097/HJH.0000000000001867	Full text not retrieved
Shaman AM, Smyth B, Arnott C, Palmer SC, Mihailidou AS, Jardine MJ*, et al.* Comparative Efficacy and Safety of BP-Lowering Pharmacotherapy in Patients Undergoing Maintenance Dialysis: A Network Meta-Analysis of Randomized, Controlled Trials. *Clin J Am Soc Nephrol* 2020;**15**:1129-38. https://doi.org/10.2215/CJN.12201019	Full text not retrieved
Sherrill B, Halpern M, Khan S, Zhang J, Panjabi S. Single-pill vs free-equivalent combination therapies for hypertension: a meta-analysis of health care costs and adherence. *J Clin Hypertens (Greenwich)* 2011;**13**:898-909. https://doi.org/10.1111/j.1751-7176.2011.00550.x	Not SR
Sommerauer C, Kaushik N, Woodham A, Renom-Guiteras A, Martinez YV, Reeves D*, et al.* Thiazides in the management of hypertension in older adults - a systematic review. *BMC Geriatr* 2017;**17**:228. https://doi.org/10.1186/s12877-017-0576-3	Quantitative focus

Sparrow RT, Khan AM, Ferreira-Legere LE, Ko DT, Jackevicius CA, Goodman SG, *et al.* Effectiveness of Interventions Aimed at Increasing Statin-Prescribing Rates in Primary Cardiovascular Disease Prevention: A Systematic Review of Randomized Clinical Trials. *JAMA Cardiol* 2019;**4**:1160-9. https://doi.org/10.1001/jamacardio.2019.3066	Full text not retrieved
Stergiou GS, Bliziotis IA. Home blood pressure monitoring in the diagnosis and treatment of hypertension: a systematic review. *Am J Hypertens* 2011;**24**:123-34. https://doi.org/10.1038/ajh.2010.194	Not SR
Sua YS, Jiang Y, Thompson DR, Wang W. Effectiveness of mobile phone-based self-management interventions for medication adherence and change in blood pressure in patients with coronary heart disease: A systematic review and meta-analysis. European journal of cardiovascular nursing 2020;19:192-200. https://doi.org/https://dx.doi.org/10.1177/1474515119895678	Medication NR
Thio SL, Nam J, van Driel ML, Dirven T, Blom JW. Effects of discontinuation of chronic medication in primary care: a systematic review of deprescribing trials. *Br J Gen Pract* 2018;**68**:e663-e72. https://doi.org/10.3399/bjgp18X699041	Quantitative focus
Thomopoulos C, Parati G, Zanchetti A. Effects of blood pressure lowering treatment in hypertension: 8. Outcome reductions vs. discontinuations because of adverse drug events - meta-analyses of randomized trials. *J Hypertens* 2016;**34**:1451-63. https://doi.org/10.1097/HJH.0000000000000972	Full text not retrieved
Thomopoulos C, Parati G, Zanchetti A. Effects of blood-pressure-lowering treatment in hypertension: 9. Discontinuations for adverse events attributed to different classes of antihypertensive drugs: meta-analyses of randomized trials. *J Hypertens* 2016;**34**:1921-32. https://doi.org/10.1097/HJH.0000000000001052	Full text not retrieved
Vassy JL, Chun S, Advani S, Ludin SA, Smith JG, Alligood EC. Impact of SLCO1B1 Pharmacogenetic Testing on Patient and Healthcare Outcomes: A Systematic Review. *Clin Pharmacol Ther* 2019;**106**:360-73. https://doi.org/10.1002/cpt.1223	Not SR
Vogele A, Johansson T, Renom-Guiteras A, Reeves D, Rieckert A, Schlender L, *et al.* Effectiveness and safety of beta blockers in the management of hypertension in older adults: a systematic review to help reduce inappropriate prescribing. *BMC Geriatr* 2017;**17**:224. https://doi.org/10.1186/s12877-017-0575-4	Quantitative focus
Webster R, Patel A, Selak V, Billot L, Bots ML, Brown A, *et al.* Effectiveness of fixed dose combination medication ('polypills') compared with usual care in patients with cardiovascular disease or at high risk: A prospective, individual patient data meta-analysis of 3140 patients in six countries. *Int J Cardiol* 2016;**205**:147-56. https://doi.org/10.1016/j.ijcard.2015.12.015	Not SR

Weeda ER, Coleman CI, McHorney CA, Crivera C, Schein JR, Sobieraj DM. Impact of once- or twice-daily dosing frequency on adherence to chronic cardiovascular disease medications: A meta-regression analysis. *Int J Cardiol* 2016;**216**:104-9. https://doi.org/10.1016/j.ijcard.2016.04.082	Not SR
Weernink MG, Vaanholt MC, Groothuis-Oudshoorn CG, Von Birgelen C, M.J IJ, Van Til JA. Patients' priorities for oral anticoagulation therapy in non-valvular atrial fibrillation. *Value in Health* 2017;**20**:A621. http://dx.doi.org/10.1016/j.jval.2017.08.1355	Abstract
Weisser B, Predel HG, Gillessen A, Hacke C, Vor dem Esche J, Rippin G*, et al.* Single Pill Regimen Leads to Better Adherence and Clinical Outcome in Daily Practice in Patients Suffering from Hypertension and/or Dyslipidemia: Results of a Meta-Analysis. *High Blood Press Cardiovasc Prev* 2020;**27**:157-64. https://doi.org/10.1007/s40292-020-00370-5	Not SR (Duplicate excluded in update search)
Wettermark B, Qvarnstrom M, Hasselstrom J, Kahan T. Controversies on patient persistence in antihypertensive drug treatment: A systematic review. *JRSM Cardiovascular Disease* 2013; http://dx.doi.org/10.1177/2048004013506452:6-7. http://dx.doi.org/10.1177/2048004013506452	Abstract
Xie CX, Chen Q, Hincapié CA, Hofstetter L, Maher CG, Machado GC, Effectiveness of clinical dashboards as audit and feedback or clinical decision support tools on medication use and test ordering: a systematic review of randomized controlled trials, *Journal of the American Medical Informatics Association*, Volume 29, Issue 10, October 2022, Pages 1773–1785, https://doi.org/10.1093/jamia/ocac094	Duplicte
Yan YL, Qiu B, Hu LJ, Jing XD, Liu YJ, Deng SB*, et al.* Efficacy and safety evaluation of intensive statin therapy in older patients with coronry heart disease: a systematic review and meta-analysis. *Eur J Clin Pharmacol* 2013;**69**:2001-9. https://doi.org/10.1007/s00228-013-1570-0	Quantitative focus
Zhao P, Xu P, Wan C, Wang Z. Evening versus morning dosing regimen drug therapy for hypertension. *Cochrane Database Syst Rev* 2011; 10.1002/14651858.CD004184.pub2:CD004184. https://doi.org/10.1002/14651858.CD004184.pub2	Quantitative focus
Zharkova T, Kyomen H. Management Strategies to Prevent Stroke in Elderly with Major Neurocognitive Disorder and Psychosis Treated with Antipsychotic Medication. *The American Journal of Geriatric Psychiatry* 2019;**27**:S135-S6. https://doi.org/10.1016/j.jagp.2019.01.042	Not SR

APPENDIX D: CHARACTERISTICS OF INCLUDED REVIEWS

Key for interpreting tables

Green highlighted text:	High overall quality as appraised by AMSTAR-2
Turquoise highlighted text:	Medium overall quality as appraised by AMSTAR-2
Orange highlighted text:	Low overall quality as appraised by AMSTAR-2
Red highlighted text:	Critically-low quality as appraised by the AMSTAR-2
CEESAT Set 1:	Prioritised for full quality appraisal using AMSTAR-2
CEESAT Set 2:	Scored poorly on 1 out of 4 items on CEESAT
CEESAT Set 3:	Scored poorly on 2-4 items on CEESAT

Reviews were judged to be of ‘High’ relevance if review focused on evaluating intervention/gathering experiences regarding medication of interest within health/social care services relevant to UK service setting, ‘Medium’ relevance: if review aim/inclusion criteria somewhat relevant to aim of systematic mapping review, but also includes other medications not of interest, or quantity of relevant information is limited, ‘Low’ relevance: if quantity of relevant information low and/or intervention being evaluated not relevant to UK health/social care setting

Shaw L, *et al. BMJ Open* 2024; 14:e072502. doi: 10.1136/bmjopen-2023-072502

Table 3: Review overview

Focus/Aim	Study (First author, date)	Type of Review (design of studies included), synthesis methods	Eligible age of participants within studies included in the review	Medications of interest	Medical condition for which medication of interest being taken	Relevance of systematic review to aim of evidence-gap map	CEESAT set	Other comments
Evaluating intervention: enhance adherence	Adler, 2017	SR (Quantitative: RCTs), Narrative	NR<br>*Mean age where reported in included studies: 53.6 years to 64 years*	Statins<br>Antihypertensives	Secondary prevention CVD	Medium	1	
Evaluating intervention: enhance adherence	Al Alshaikh, 2016	SR(Quantitative: RCTs), MA+Narrative	18 years or over	Statins<br>Antihypertensives	Secondary prevention CVD	Medium	3	
Evaluating intervention: enhance adherence	Baumgartner, 2020	SR (Quantitative: RCTs, retrospective/prospective cohort, non-randomized, economic models, systematic reviews, meta-analysis), Narrative	NR<br>*Where reported, participants in included studies 18 years of age or over. Some studies specifically included adults 65 years or older*	Statins<br>Antihypertensives	HBP<br>Primary prevention CVD<br>Secondary prevention CVD	Medium	3	
Evaluating intervention: enhance adherence	Bochkareva, 2019	Scoping review (Quantitative: RCTs, non-RCTs, (cross-sectional, cohort studies), registers), Narrative Matrix/map of studies	18 years or over	Antihypertensives	HBP	Medium	3	Focus on Russia. Boad focus of scoping review, of which interventions to increase adherence are one part
Evaluating intervention: enhance adherence	Checchi, 2014	SR (Quantitative: RCTs, nonRCT, controlled, prospective/retrospective, observational, cohort), Narrative	NR<br>*Adults*	Antihypertensives<br>Statins	HBP<br>Primary prevention CVD	Medium	3	

Shaw L, *et al. BMJ Open* 2024; 14:e072502. doi: 10.1136/bmjopen-2023-072502

Focus/Aim	Study (First author, date)	Type of Review (design of studies included), synthesis methods	Eligible age of participants within studies included in the review	Medications of interest	Medical condition for which medication of interest being taken	Relevance of systematic review to aim of evidence-gap map	CEESAT set	Other comments
Evaluating intervention: enhance adherence Secondary aim	Cheema, 2014	SR (Quantitative: RCTs, systematic reviews of RCTs), Meta-analysis+Narrative	18 years or over	Antihypertensives	HBP	High	2	
Evaluating intervention: optimise prescribing	Chhina, 2013	SR (Quantitative: RCT, observational studies with a control group), Narrative	NR *1 included study focused on children, 3 focused on elderly*	Antihypertensives	HBP	Medium	3	One included study focused on children
Evaluating intervention: enhance adherence	Conn, 2015[70]	SR (Quantitative: NR), Meta-analysis	NR *Median value for mean age accross included studies was 54.4 years*	Statins Antihypertensives	HBP Secondary prevention CVD	Medium	2	Medications of interest inferred from population disease characteristics
Evaluating intervention: enhance adherence	Conn, 2015[71]	SR (Quantitative: treatment-versus-control comparisons, pre-experimental studies), Exploratory analyses, Meta-analysis	NR *Median mean participant age accross included studies was 60.70 years*	Statins Antihypertensives	HBP Primary prevention CVD Secondary prevention CVD Inferred from population disease characteristics	Medium	2	Medications of interest inferred from population disease characteristics
Evaluating intervention: enhance adherence	Conn, 2015[36]	SR (Quantitative: varied research methods were included), Meta-analysis	Other *Adults*	Antihypertensives	HBP	High	3	

Focus/Aim	Study (First author, date)	Type of Review (design of studies included), synthesis methods	Eligible age of participants within studies included in the review	Medications of interest	Medical condition for which medication of interest being taken	Relevance of systematic review to aim of evidence-gap map	CEESAT set	Other comments
Evaluating intervention: enhance adherence	Bond, 2021	SR (Quantitative: RCTs), Narrative	Any age	Statins	Primary and Secondary prevention CVC	High	3	
Evaluating intervention: deprescribing	Chambergo-Michilot, 2023	SR (Quantitative: observational studies, trials, RCT, cohort), Meta-analysis	NR: *In majority of studies, mean/median age was higher than 60 years*	Antihypertensives	HBP	High	3	
Evaluating intervention: enhance adherence	Conn, 2016	SR (Quantitative: RCTs, non-RCTs, Pre-experimental before and after), Narrative+Meta-analysis	NR *Adults. Median mean age accross included studies 53 years*	Statins Antihypertensives	Primary prevention CVD Depression HBP Secondary prevention CVD	Medium	2	Medications of interest inferred from population disease characteristic
Evaluating intervention: enhance adherence/optimise prescribing	Coronado-Vazquez, 2020	SR (Quantitative: RCT, CT), Narrative	NR *mean age of the participants in the reviewed studies ranged from 8 years[32] to 73 years*	Statins Antihypertensives	Primary and secondary prevention CVD	Low	2	
Evaluating intervention: enhance adherence secondary aim	de Cates, 2014	SR (Quantitative: RCTs), Meta-analysis	18 years or over	Statins Antihypertensives	Primary prevention CVD Secondary prevention CVD	Medium	1	Adherence only secondary outcome
Evaluating intervention: enhance adherence	De Simoni, 2013	Quantitative (RCTs), Narrative+Meta-analysis	Other *Adults*	Antihypertensives	HBP Secondary prevention CVD	High	2	

**Focus/Aim**	**Study (First author, date)**	**Type of Review (design of studies included), synthesis methods**	**Eligible age of participants within studies included in the review**	**Medications of interest**	**Medical condition for which medication of interest being taken**	**Relevance of systematic review to aim of evidence-gap map**	**CEESAT set**	**Other comments**
Evaluating intervention: enhance adherence	Deichmann. 2016	SR (Quantitative: RCTs, parallel group or crossover, individual or cluster randomization), Meta-analysis+Narrative	18 years or over	Statins	Primary prevention CVD<br>Secondary prevention CVD	Medium	3	
Evaluating intervention: enhance adherence	Demonceau, 2013	SR (Quantitative: RCT, cross-over and cluster-randomized), Meta-analysis+meta-regression	Other<br>*Average age expressed in years was 47.5 [range (3.0; 73.7)] in the usual care group and 46.8 [range (3.4; 76.2)] in the intervention group*<br>*Adults and children*	Antihypertensives<br>*Assumed*	HBP<br>NR	Medium	3	Included methadone clinic patients (n=1). Included children
Patient views: adherence to medication of interest<br>Only these of interest	Dhar, 2017	SR (Mixed: only quali studies met focus of review. Quantitative, descriptive-co relational study, cross-sectional, focus group interviews, survey, interviews), Narrative	18 years or over	Antihypertensives	HBP	Low	3	Focus on undeveloped countries. Only 5 included qualitative studies

**Focus/Aim**	**Study (First author, date)**	**Type of Review (design of studies included), synthesis methods**	**Eligible age of participants within studies included in the review**	**Medications of interest**	**Medical condition for which medication of interest being taken**	**Relevance of systematic review to aim of evidence-gap map**	**CEESAT set**	**Other comments**
Evaluating intervention: deprescribing	Dills, 2018	SR (Quantitative: Dills-2019-Deprescribing Medications for Chronic.pdf: Page 1: "Randomized Controlled Trials" Narrative	18 years or over	Statins Antihypertensives	HBP Secondary prevention CVD Other Illnesses associated with use of: alendronate, PPI, H2 antagonist	Medium	1	Palliative care studies where life expectancy is > 6 months included
Evaluating intervention: enhance adherence	Du, 2018	SR (Quantitative: parallel comparison studies, retrospective cohort, prospective), Meta-analysis	NR *Mean age of included studies (where reported) ranged from 53-76 years*	Antihypertensives	HBP	High	3	
Evaluating intervention: enhance adherence	Fletcher, 2015	SR (Quantitative: Randomized and quasi-randomized trials), Narrative+Meta-analysis	NR *Where reported, participants mean age ranged from 38 years to 77 accross included studies*	Antihypertensives	HBP	High	1	
Evaluating intervention: enhance adherence	Gupta, 2010	SR (Quantitative: RCT, clinical trial and prospective or retrospective cohort), Narrative+Meta-analysis	Other *18 to 79 years*	Antihypertensives	HBP	High	3	
Evaluating intervention: enhance adherence	Guzman-Tordecilla, 2020	SR (Quantitative: Guzman-Tordecilla-2020-InterventionsToIncreaseThePhar.pdf: Page 5: "Quasi-experimental" "Experimental" Narrative	17 years or over	Antihypertensives	HBP	Low	3	Studies conductedi n Latin America

Focus/Aim	Study (First author, date)	Type of Review (design of studies included), synthesis methods	Eligible age of participants within studies included in the review	Medications of interest	Medical condition for which medication of interest being taken	Relevance of systematic review to aim of evidence-gap map	CEESAT set	Other comments
Evaluating intervention: deprescribing Secondary aim, not actually explicit. Look at interventions to reduce Fall Risk-Inducing Drugs (FRID) in an attempt to understand whether this reduced falls.	Hart, 2020	SR (Quantitative: observational or intervention (eg, RCTs and quasi-experimental design)), Narrative	Other *60 or over*	Antihypertensives	NR	Medium	3	Lots of other drugs. Also approach reduction of drugs of interest in a round-about way, rather than directly wanting to reduce them
Evaluating intervention: enhance adherence	Hong, 2022	SR (Quantitative: RCT, cross-sectional), Narrative	NR *Only adults were recruited*	Antihypertensives	HBP Secondary prevention CVD	High	3	
Evaluating intervention: optimise prescribing	Hukins, 2019	SR (Quantitative: observational, non-randomised evaluation), Narrative	Other *People with a diagnosis of/suspected dementia*	Statins	NR	Medium	1	

**Focus/Aim**	**Study (First author, date)**	**Type of Review (design of studies included), synthesis methods**	**Eligible age of participants within studies included in the review**	**Medications of interest**	**Medical condition for which medication of interest being taken**	**Relevance of systematic review to aim of evidence-gap map**	**CEESAT set**	**Other comments**
Patient views: adherence to medication of interest<br>Patient views: intervention to enhance adherence	Ingersgaard, 2020	Review of SR (Other, all types of systematic reviews), Narrative	NR	Statins<br>Antihypertensives	Primary prevention CVD<br>Secondary prevention CVD	Medium	2	Review of reviews, little relevant qualitative evidence
Evaluating intervention: optimise prescribing	Jeffery, 2015	SR (Quantitative: RCTs, cluster and nexted), Meta-analysis+Narrative	NA<br>T*argeted prescribers*	Statins<br>Antihypertensives	Primary prevention CVD<br>Secondary prevention CVD	High	1	
Evaluating intervention: optimise prescribing	Kang, 2016	SR (Quantitative: RCTs, non-RCT, prospective observational studies), Narrative+Meta-analysis	18 years or over	Statins<br>Antihypertensives	Secondary prevention CVD	High	3	
Evaluating intervention: enhance adherence	Kassavou, 2018	SR (Quantitative: RCTs, randomisation at individual patient or cluster level), Meta-analysis+meta-regression	18 years or over	Statins<br>Antihypertensives	HBP<br>Primary prevention CVD<br>Secondary prevention CVD<br>Other Cardio-metabolic conditions, high cholesterol	High	2	
Evaluating intervention: enhance adherence	Kawalec, 2018	SR (Quantitative: No restrictions were applied as to the study design. SRs, cohort, non-RCT, prospective observational study), Narrative+Meta-analysis	NR<br>*Where reported, all included studies included participants of 18 years or older*	Antihypertensives	HBP	High	3	

**Focus/Aim**	**Study (First author, date)**	**Type of Review (design of studies included), synthesis methods**	**Eligible age of participants within studies included in the review**	**Medications of interest**	**Medical condition for which medication of interest being taken**	**Relevance of systematic review to aim of evidence-gap map**	**CEESAT set**	**Other comments**
Practitioner views: prescribing medication of interest<br>Patient views: adherence to medication of interest	Khatib, 2014	SR (Mixed: Qualitative and quantitative observational studies. Included RCT, cohort, case-control), Framework/Content analysis, Narrative	Other<br>*Any age*	Antihypertensives	HBP	High	2	Methods of qualitative synthesis unclear
Evaluating intervention: optimise prescribing<br>Think this is most appropriate overall, but could justify selecting more than one?<br>Evaluating intervention: enhance adherence	King, 2018	SR (Quantitative: RCT, observational), Narrative	NR	Statins<br>Antihypertensives	HBP<br>Primary prevention CVD	Medium	2	
Patient views: adherence to medication of interest<br>Family/carer views: adherence to medication of interest	Kinnear, 2019	SR (Qualitative: face to face/telephone/online interviews, focus groups) Thematic	NR<br>*264 individuals with FH and 13 family members were involved, aged 8-69 years*	Statins	Primary prevention CVD<br>Secondary prevention CVD	Medium	1	N=7 papers reported findings from samples including individuals under 18 years. N=4 papers reported parental views of having children with FH
Evaluating intervention: deprescribing	Kornholt, 2022	Scoping review (Quantitative: RCT), Narrative/descriptive statistics	65 years or over	Statins<br>Antihypertensives	HBP<br>Primary and Secondary CVD	Medium	2	

**Focus/Aim**	**Study (First author, date)**	**Type of Review (design of studies included), synthesis methods**	**Eligible age of participants within studies included in the review**	**Medications of interest**	**Medical condition for which medication of interest being taken**	**Relevance of systematic review to aim of evidence-gap map**	**CEESAT set**	**Other comments**
Evaluating intervention: enhance adherence	Krack, 2019	SR (Quantitative: RCTs, non-RCTs), Narrative+Meta-analysis	NR	Statins Antihypertensives	HBP Primary prevention CVD Secondary prevention CVD	Low	2	
Evaluating intervention: enhance adherence	Kronish, 2011	SR (Quantitative: observational cohorts), Meta-analysis	18 years or over	Antihypertensives	HBP	High	2	
Evaluating intervention: enhance adherence Evaluating intervention: optimise prescribing Because of interventions directed at HCPs	Laba, 2013	SR (Quantitative: Randomised/quasi-randomised controlled trials), Narrative	Other *Adults*	Statins Antihypertensives	HBP Primary prevention CVD P*ossibly secondary prevention, unclear*	Medium	3	
Evaluating intervention: enhance adherence	Lawrence, 2015	SR (Quantitative: RCTs), Meta-analysis	18 years or over	Statins Antihypertensives	Secondary prevention CVD P*revention of secondary stroke and/or TIA*	Medium	2	Only 3 studies reporting adherence outcomes for each of statins and antihypertensives
Evaluating intervention: optimise prescribing Secondary aim	Legare, 2010	SR (Quantitative: RCTs, well designed quasi-experimental studies (controlled clinical trials, CBA, ITS), Narrative	NR	Statins	NR	Low	1	Possibly only one study (of 5) about statins, the others are not relevant

Focus/Aim	Study (First author, date)	Type of Review (design of studies included), synthesis methods	Eligible age of participants within studies included in the review	Medications of interest	Medical condition for which medication of interest being taken	Relevance of systematic review to aim of evidence-gap map	CEESAT set	Other comments
Evaluating intervention: enhance adherence	Leslie, 2016	SR (Quantitative: RCTs), Narrative	65 years or over *or consisted of Medicare beneficiaries*	Antihypertensives Statins	HBP Primary prevention CVD	Medium	3	Focus on elderly. Medications of interest assumed from population characteristics
Evaluating intervention: optimise prescribing	Loganathan, 2011	SR (Quantitative: RCTs, non-RCTs), Narrative	65 years or over	Antihypertensives	NR	Medium	3	Only a couple of included studies with medications of interest
Evaluating intervention: enhance adherence Secondary aim	Maimaris, 2013	SR (Quantitative: RCTs, controlled trials, cohort, cross-sectional, case-control, cross-sectional, ecological), Narrative	NR	Antihypertensives	HBP	Medium	1	Limited information relating to adherence of hypertensives. Uncertain as to relevance of findings to UK health and social care setting
Evaluating intervention: enhance adherence Seconardy aim	Mallat, 2016	SR (Quantitative: RCTs), Meta-analysis	18 years or over	Antihypertensives	HBP	High	1	
Evaluating intervention: enhance adherence	Mamudu, 2014	SR (Quantitative: RCTs, non-RCTs), Narrative	NR	Statins Antihypertensives	Primary prevention CVD	Medium	3	
Evaluating intervention: enhance adherence	Mann, 2014	SR (Quantitative: RCTs, ITS, CBA), Narrative	Other *Adult patients*	Statins Antihypertensives	HBP Primary prevention CVD Secondary prevention CVD	Low	2	One included study included participants aged 14 and above. Intervention not relevant to UK setting

Focus/Aim	Study (First author, date)	Type of Review (design of studies included), synthesis methods	Eligible age of participants within studies included in the review	Medications of interest	Medical condition for which medication of interest being taken	Relevance of systematic review to aim of evidence-gap map	CEESAT set	Other comments
Patient views: adherence to medication of interest<br>Qualitative: other<br>Understanding and experiences of hypertension and drug adherence	Marshall, 2012	SR (Qualitative: Interviews, focus groups), Narrative/Thematic	NR<br>*Where reported, age of population within included studies ranged from 18-92 years*	Antihypertensives	HBP	High	3	
Evaluating intervention: enhance adherence	Milosavljevic, 2018	SR (Quantitative: RCTs, quasi-controlled trials, cluster-controlled trials, BA, retrospective/prospective cohort, observational, single group pre/post comparisons, statistical predictions), Narrative	NR	Antihypertensives	HBP<br>Depression	Medium	2	
Evaluating intervention: enhance adherence	Morrissey, 2017	SR (Quantitative: RCTs), Narrative Meta-analysis	NR<br>*Where reported, mean age ranged from 51 to 76 accross included studies*	Antihypertensives	HBP	High	1	
Evaluating intervention: optimise prescribing	Nguyen, 2018	SR (Quantitative: RCTs), Meta-analysis	NR	Statins	Secondary prevention CVD<br>*Ischaemic heart disease*	Medium	2	
Evaluating intervention: enhance adherence	Nieuwlaat, 2014	SR (Quantitative: RCTs), Narrative	NR<br>*Included studies characteristics table indicates both adult and child populations included*	Statins<br>Antihypertensives	HBP<br>Primary prevention CVD<br>Secondary prevention CVD	Medium	1	

Shaw L, *et al. BMJ Open* 2024; 14:e072502. doi: 10.1136/bmjopen-2023-072502

Focus/Aim	Study (First author, date)	Type of Review (design of studies included), synthesis methods	Eligible age of participants within studies included in the review	Medications of interest	Medical condition for which medication of interest being taken	Relevance of systematic review to aim of evidence-gap map	CEESAT set	Other comments
Evaluating intervention: enhance adherence	Nili, 2020	SR (Quantitative: RCT), Narrative	Adults	Antihypertensives	HBP	High	1	
Qualitative: other Factors which predict medication adherence. Not a qualitative review but didn't know what else to select What factors are associated with/influence or predict ad- herence of anti-hypertensive medication in older adults with HBP?, How are their causal relationship and their influence on MA in older adults with HBP based on ecological ap- proach?	Oori, 2019	Integrative review (Mixed: quantitative and qualitative designs, including: cross-sectional and descriptive, interventional, cohort and two descriptive qualitative), Content analysis	Older adults	Antihypertensives	HBP	Low	3	A qualitative synthesis of quant/qual data. Included because of qualitative aspect, but only 'descriptive qualitative' studies were included

Focus/Aim	Study (First author, date)	Type of Review (design of studies included), synthesis methods	Eligible age of participants within studies included in the review	Medications of interest	Medical condition for which medication of interest being taken	Relevance of systematic review to aim of evidence-gap map	CEESAT set	Other comments
Evaluating intervention: deprescribing	Page, 2016	SR (Quantitative: RCTs, quasi-randomized controlled studies, non-RCTs, prospective and retrospective cohort, case-control, single arm, BA), Meta-analysis	65 years or over	Statins Antihypertensives	HBP Primary prevention CVD Secondary prevention CVD	Medium	2	
Evaluating intervention: enhance adherence	Palmer, 2018	SR (Quantitative: RCTs of parallel group design that randomised by participant or by cluster), Narrative	18 years or over	Statins Antihypertensives	Primary prevention CVD	Medium	1	
Evaluating intervention: enhance adherence	Palmer, 2020 (update 2018)	SR (Quantitative: RCT), Meta-analysis+Narrative	18 years or over	Statins Antihypertensives	Primary prevention CVD	Medium	1	
Evaluating intervention: optimise prescribing One of a variety of aims	Pantoja, 2019	SR (Quantitative: RCTs, non-RCTs), Narrative	NR	Statins Antihypertensives	HBP Primary prevention CVD *Unclear if statin for primary or secondary prevention*	Medium	1	Some included studies included child participants. Low amount of information about prescribing drugs of interest. intervention is more of a general aid

**Focus/Aim**	**Study (First author, date)**	**Type of Review (design of studies included), synthesis methods**	**Eligible age of participants within studies included in the review**	**Medications of interest**	**Medical condition for which medication of interest being taken**	**Relevance of systematic review to aim of evidence-gap map**	**CEESAT set**	**Other comments**
Evaluating intervention: enhance adherence	Parati, 2021	SR (Quantitative: Retrospective data- base analysis, prospective, open- label, non-randomized, 2 phased, International, randomized, open-label, controlled, 12-wk phase III trial, multi-center, observational study, retrospective database analysis, retrospective cohort study, non-RCT, observational, randomized, prospective, multi-center trial, interventional parallel group, interventional study), Narrative+Meta-analysis	18 years or over	Antihypertensives	HBP	High	2	
Evaluating intervention: enhance adherence	Park, 2017	SR (Quantitative: Original research, comparison group, pre/post comparisons, statistical predictions), Narrative	NR	Antihypertensives Statins	HBP Primary prevention CVD NR	Low	3	Not relevant to UK, medications not of interest included
Evaluating intervention: enhance adherence Note that focus is on 'use' which includes initiating prescribing. Drugs of interest may be currently under- or -ver-used	Polinski, 2011	SR (Quantitative: Polinski-2011-Medicare Part D's Effect on the.pdf: Page 3: "Articles were included if they reported original results regarding Part D drug utilization for specific drugs or drug classes, whether drawn from self-report surveys or prescription drug claims" Narrative	NR	Statins Antihypertensives	HBP Primary prevention CVD NR *Not clearly reported*	Low	3	Low relevance to UK. Other medications not of interest included

**Focus/Aim**	**Study (First author, date)**	**Type of Review (design of studies included), synthesis methods**	**Eligible age of participants within studies included in the review**	**Medications of interest**	**Medical condition for which medication of interest being taken**	**Relevance of systematic review to aim of evidence-gap map**	**CEESAT set**	**Other comments**
Evaluating intervention: enhance adherence Seconary aim: interventions to affect behaviour change that leads to prevention of chronic disease - e.g. taking antihypertensives...	Posadzki, 2016	SR (Quantitative: RCTs, cluster RCTs, quasi-RCTs, ITS, CBA), Narrative+Meta-analysis	NR	Statins Antihypertensives	HBP Primary prevention CVD	Medium	1	Included children. Interventions target general positive behaviour change in terms of health management. However there are specific studies reporting adherence-type interventions. Several non-relevant drugs and outcomes within the review.
Prescriber, patient, and/or family/carer views of issues relating to prescribing and/or adherence to medications of interest	Qadi, 2020	SR(Qualitative) Thematic	NR *Excludes studies initiating medication in older age group*	Statins Antihypertensives	Primary prevention CVD	High	2	
Evaluating intervention: enhance adherence	Rash, 2016	SR (Quantitative: RCTs), Narrative	NR	Statins	Primary prevention CVD Secondary prevention CVD	High	2	
Patient views: adherence to medication of interest	Rashid, 2014	SR (Qualitative: Focus groups, interviews, interviews and questionnaires), Meta-ethnography	Other *Adults*	Statins	Secondary prevention CVD	Medium	3	

Shaw L, *et al. BMJ Open* 2024; 14:e072502. doi: 10.1136/bmjopen-2023-072502

**Focus/Aim**	**Study (First author, date)**	**Type of Review (design of studies included), synthesis methods**	**Eligible age of participants within studies included in the review**	**Medications of interest**	**Medical condition for which medication of interest being taken**	**Relevance of systematic review to aim of evidence-gap map**	**CEESAT set**	**Other comments**
Qualitative: other Rashid 2018.pdf: Page 1: "explored medication-taking experiences in patients, citizens, carers, relatives and clinicians."	Rashid, 2018	SR (Qualitative: All types of qualitative studies, including those linked to observational, experimental, mixed methods papers. Interviews, focus groups or a combination), Thematic	Other	Antihypertensives	HBP	Medium	3	Includes children and adults. Other medications not of interest included
Patient views: intervention to encourage deprescribing Family/carer views: intervention to encourage deprescribing	Reeve, 2013	SR (Mixed: No limits placed on the type of methods used, qualitative, quantitative or mixed), Narrative/Content analysis/Thematic	Other *No limits were placed on the age of the participants*	Antihypertensives	HBP Anxiety NR	Medium	3	Methods of synthesis unclear
Evaluating intervention: enhance adherence	Reeves, 2020	SR (Quantitative: RCTs), Narrative	18 years or over *Adult*	Antihypertensives	HBP	High	2	
Evaluating intervention: enhance adherence	Reston, 2020	SR (Quantitative: SR, RCT, observational), Narrative	18 years or over	Statins	Primary prevention CVD	High	2	
Evaluating intervention: enhance adherence One of multiple aims	Sanders, 2011	SR (Quantitative: comparative clinical studies of any design, including RCTs, non-RCTs, retrospective/prospective cohort, and case-control), Narrative+Meta-analysis	18 years or over	Antihypertensives	HBP *Essential hypertension*	Medium	1	Adherence low on the list of outcomes

**Focus/Aim**	**Study (First author, date)**	**Type of Review (design of studies included), synthesis methods**	**Eligible age of participants within studies included in the review**	**Medications of interest**	**Medical condition for which medication of interest being taken**	**Relevance of systematic review to aim of evidence-gap map**	**CEESAT set**	**Other comments**
Evaluating intervention: optimise prescribing	Shanbhag, 2018	SR (Quantitative: RCTs, cohort (with comparisons), controlled and uncontrolled BA, ITS), Narrative	NR	Antihypertensives	HBP *Associated with heart failure*	High	1	
Evaluating intervention: enhance adherence	Stacey, 2017	SR (Quantitative: RCTs), Meta-analysis	18 years or over	Antihypertensives	HBP Primary prevention CVD Seconday prevention CVD	Medium	1	Study focus: RE: making decisions about screening or treatment options for themselves, a child, or an incapacitated significant other
Evaluating intervention: enhance adherence	Tsioufis, 2020	Rapid review (Quantitative: RCTs, observational studies), Narrative	Other *Adults. Where reported, mean age of populations in included studies ranged from 47 to 71.5 years*	Antihypertensives	HBP	High	3	
Evaluating intervention: enhance adherence	Uhlig, 2013	SR (Quantitative: prospective comparative), Narrative+Meta-analysis	Other *Adults*	Antihypertensives	HBP	High	3	
Evaluating intervention: enhance adherence	van Driel, 2016	SR (Quantitative: RCTs of parallel-group or cross-over design, that used individual or cluster randomisation), Narrative+Meta-analysis	18 years or over	Statins	Primary prevention CVD Seconday prevention CVD	High	1	
Evaluating intervention: enhance adherence	Van Truong, 2021	SR (Quantitative: RCT), Meta-analysis	Other: 60 years or above	Antihypertensives (assumed)	HBP	Medium	2	

Shaw L, *et al. BMJ Open* 2024; 14:e072502. doi: 10.1136/bmjopen-2023-072502

Focus/Aim	Study (First author, date)	Type of Review (design of studies included), synthesis methods	Eligible age of participants within studies included in the review	Medications of interest	Medical condition for which medication of interest being taken	Relevance of systematic review to aim of evidence-gap map	CEESAT set	Other comments
Evaluating intervention: enhance adherence	Verveloet, 2020	SR (Quant: RCT, controlled CT, CT), Narrative	NR	Statins Antihypertensives	HBP	High	2	
Evaluating intervention: enhance adherence and prescription	Xie,2022	SR (Quantitative: RCT), Narrative	NR	Statin Antihypertensives	HBP, Other: atherosclerotic CVD	Low	2	
Evaluating intervention: enhance adherence	Xiong, 2018	SR (Quantitative: RCTs, non-RCTs, before-and-after studies without a control group), Narrative	NR *All studies involved adults aged from 40 to 69*	Antihypertensives	HBP	High	3	
Evaluating intervention: enhance adherence	Xu, 2018	SR (Quantitative: RCTs), Meta-analysis	18 years or over	Antihypertensives	HBP	Medium	3	Focus on Chinese patients
Evaluating intervention: optimise prescribing Secondary aim Evaluating intervention: enhance adherence Secondary aim	Yuan, 2019	SR (Quantitative: RCTs, high quality cohort studies), Narrative+Meta-analysis	NR	Antihypertensives	HBP Primary prevention CVD Seconday prevention CVD *Assumed from content of paper*	Medium	3	

Focus/Aim	Study (First author, date)	Type of Review (design of studies included), synthesis methods	Eligible age of participants within studies included in the review	Medications of interest	Medical condition for which medication of interest being taken	Relevance of systematic review to aim of evidence-gap map	CEESAT set	Other comments
Evaluate intervention: optimise prescribing. Guidelines	Zheng, 2023	SR (Quantitative: RCT, non-randomized observational), meta-analysis	Adults	Antihypertensives	HBP	High	1	

Green highlighted text=High overall quality as appraised by AMSTAR-2, Turquoise highlighted text=Medium overall quality as appraised by AMSTAR-2, Orange highlighted text=Low overall quality as appraised by AMSTAR-2, Red highlighted text=Critically-low quality as appraised by the AMSTAR-2. *CEESAT Set: 1=prioritised for full quality appraisal using AMSTAR-2, CEESAT Set 2=Scored poorly on 1 out of 4 items on CEESAT, CEESAT Set 3=Scored poorly on 2-4 items on CEESAT. BA=Before and After, BPD=Borderline Personality Disorder, CBA=Controlled Before and After, CPG=Clinical Practice Guidelines, CT=Controlled-Trials, CVD=Cardiovascular Disease, FH=Familial Hypercholesterolemia, HPP=High Blood Pressure, ITS=Interrupted Time Series, MA=Meta-analysis, NR=Not Reported, PPI=Proton Pump Inhibitors, PTSD=Post-Traumatic Stress Disorder, RCT=Randomized Controlled Trial, RQ=Research Question, SR=Systematic Review

Table 4: Systematic reviews appraised using AMSTAR-2 which synthesised studies evaluating the effectiveness of an intervention

Focus/ Aim	Study (First author, date)	Medications of interest	Other medications included	Intervention name/s and aim	Outcomes measured	Setting/Context	Relevant part of care pathway
Evaluating intervention: deprescribing	Dills, 2018	Statins<br>Antihypertensives	Diuretics, salmeterol, insulin, sulfonylurea, antipsychotics, anxiolytic, CV drugs, levodopa, nitrate, benzodiazepines<br>Z-drugs<br>Antidepressants	Deprescription: reduce medication burden, and enhance control of chronic medical and mental health conditions commonly managed by primary care physicians, compared with standard care in the non-terminally ill adult population	Primary outcome: successful deprescription, Secondary outcome: AE related to drug or underlying chronic condition as a result of deprescription	Primary/health /social care<br>Hospital (inpatient)<br>Hospital (outpatient)<br>Care home<br>Patient home	Decision to stop treatment: supported
Evaluating intervention: enhance adherence	Stacey, 2017	Antihypertensives	Hepatitis B vaccination, schizophrenia medication, immunotherapy for multiple sclerosis, vaccines (measles, mumps, rubella), radioactive iodine treatment for thyroid cancer, antibiotic use for upper respiratory infections, chemotherapy for cancer, medications for diabetes, hormone therapy, "breast cancer prevention medication, bisphosphonates, thyroid cancer radioactive iodine treatment	Decision aids for people facing health treatment or screening decisions.<br>Aim: help people make specific and deliberated choices among options, by making the decision explicit/by providing information on the options and outcomes relevant to person's health status as well as implicit methods to clarify values	Attributes of choice made: does patient decision aid improve match between chosen option and features that matter most to informed patient (demonstrated by knowledge, accurate risk perceptions, values-choice congruence).<br><br>Attributes of decision-making process: does patient decision aid help patients to recognize decision needs to be made, feel informed about options and their features, be clear about option features that matter most, discuss values with their clinician, and become involved in decision making? Other decision-making process variables: Decisional conflict, Patient-clinician communication, participation in decision making, proportion undecided, satisfaction with the choice/process of decision making/preparation for decision	NR	Information and/or advice<br>Shared decision making<br>Patient adherence

Focus/ Aim	Study (First author, date)	Medications of interest	Other medications included	Intervention name/s and aim	Outcomes measured	Setting/Context	Relevant part of care pathway
					making. Secondary outcomes: Choice (the actual choice implemented; if not reported, the participants preferred option), adherence to chosen option, health status and quality of life (generic and condition-specific), anxiety, depression, emotional distress, regret, confidence, costs, cost-effectiveness, consultation length, litigation rates		

Shaw L, *et al. BMJ Open* 2024; 14:e072502. doi: 10.1136/bmjopen-2023-072502

Focus/ Aim	Study (First author, date)	Medications of interest	Other medications included	Intervention name/s and aim	Outcomes measured	Setting/Context	Relevant part of care pathway
Evaluating intervention: enhance adherence*	Posadzki 2016	Statins Antihypertensives	Asthma medication, prescription drugs taken without prescription or more than prescribed, illicit non-injection drugs, glaucoma medications, inhaled corticosteroids, insulin, methadone maintenance, cholinesterase inhibitor medications, oral hypoglycaemic medications, nicotine replacement therapy, bupropion, and varenicline, antiretroviral treatment, bone active medication, non-hormonal oral agents, opiates antidepressants	Automated telephone communication systems for preventing disease and managing long-term conditions	Health behaviour and clinical outcomes, changes in health-enhancing behaviour, risk-taking behaviour, physiological measures, blood biochemistry. Process outcomes: change in acceptability of service (e.g. consumer accessibility/usability of interventions to apply information/support supplied), satisfaction (e.g. patient/carer) the intervention), cost-effectiveness. Cognitive outcomes: changes in knowledge (i.e. accurate risk knowledge and perception), attitude and intention to change, self-efficacy. Patient-centred outcomes: quality of life, adverse outcomes, unintended AE attributable to intervention	Primary/health/ social care Secondary health/social care (not hospital) Hospital (inpatient) Hospital (outpatient) Patient home	Identifying health or prescribing concern Accessing care Choosing a medication Decision to start agreed treatment plan Patient adherence Maintaining treatment
Evaluating intervention: enhance adherence	Adler, 2017	Statins Antihypertensives	Antiplatelet	Mobile phone text messaging to enhance adherence to recommended medication in patients with established arterial occlusive events	Adherence to treatment, fatal/non-fatal cardiovascular events, combined CVD event, Surrogate outcomes according to the different interventions recommended for secondary prevention including: low-density lipoprotein-cholesterol, blood pressure, heart rate, urinary 11-dehydrothromboxane B2, AEs	Primary/health /social care Hospital (outpatient)	Patient adherence

Focus/ Aim	Study (First author, date)	Medications of interest	Other medications included	Intervention name/s and aim	Outcomes measured	Setting/Context	Relevant part of care pathway
Evaluating intervention: enhance adherence	Fletcher, 2015	Antihypertensives	NA	Self-monitoring of blood pressure: improve medication adherence, medication persistence, and lifestyle factors in people with hypertension	Primary outcomes: antihypertensive medication adherence and persistence, dietary outcomes, alcohol consumption and physical activity. Secondary outcomes: BP, BP control, and adherence to SMBP component of interventions	Primary/health/ social care<br>Hospital (outpatient)	Decision to start agreed treatment plan<br>Patient adherence<br>Progress review
Evaluating intervention: enhance adherence	Morrissey, 2017	Antihypertensives	NA	Interventions to enhance medication adherence and blood pressure control in hypertension	Change in BP, medication adherence	Primary/health/ social care<br>Hospital (inpatient)<br>Hospital (outpatient)<br>Care home<br>Patient home	Patient adherence
Evaluating intervention: enhance adherence	Nieuwlaat, 2014	Antidepressants<br>Statins<br>Antihypertensives	Medications to treat following conditions: HIV/AIDS, psychiatric disorders, chronic obstructive pulmonary disease, CVD/CV risk, diabetes, antibiotics, arthritis, complex chronic care, dyspepsia, glaucoma, oral anticoagulation, osteoporosis, tuberculosis, acne, cancer, hepatitis, iron supplementation during pregnancy, liver transplant, malaria, oral contraceptives,	Interventions to affect adherence with prescribed, self-administered medications	Medication adherence, clinical outcomes	Primary/health/ social care<br>Hospital (inpatient)<br>Hospital (outpatient)<br>Care home<br>Patient home	Information and/or advice<br>Patient adherence<br>Progress review

Focus/ Aim	Study (First author, date)	Medications of interest	Other medications included	Intervention name/s and aim	Outcomes measured	Setting/Context	Relevant part of care pathway
			antidepressants tonsillectomy/adenoidectomy, ulcerative colitis				
Evaluating intervention: enhance adherence	Palmer, 2018	Statins<br>Antihypertensives	Antiplatelet drugs (low-dose aspirin, non-aspirin antiplatelet drugs)	Mobile phone-based interventions to improve adherence to medication prescribed for the primary prevention of cardiovascular disease in adults	Objective measures of adherence to treatment, combined CVD events, AE, indirect measures of adherence to treatment, health-related quality of life, cognitive outcomes (satisfaction with treatment, medication-taking self-efficacy, autonomy related to medication, attitudes (e.g. concerns about medicine AEs)), costs, process measures: extent of intervention received and acceptability of intervention	Primary/health/ social care<br>Secondary health/social care (not hospital)<br>Hospital (outpatient)<br>Patient home	Progress review<br>Patient adherence
Evaluating intervention: enhance adherence	Palmer, 2020	See above	See above	See above	See above	See above	See above

Focus/ Aim	Study (First author, date)	Medications of interest	Other medications included	Intervention name/s and aim	Outcomes measured	Setting/Context	Relevant part of care pathway
Evaluating intervention: enhance adherence	van Driel, 2016	Statins	Any lipid-lowering medication	Interventions to improve adherence to lipid-lowering drugs: 1. Simplification of drug regimen; 2. Patient education and information; 3. Intensified patient care; 4. Complex behavioural approaches; 5. Decision support systems; 6. Administrative improvements; and 7. Pharmacy-led interventions	Indirect measures of adherence, subjective measures of adherence, direct measures of adherence, physiological indicators, health outcome indications (e.g. quality of life, morbidity, mortality), AEs, implications for costs (impact of intervention on economic outcomes, economic evaluation)	Primary/health /social care<br>Secondary health/social care (not hospital)	Information and/or advice<br>Patient adherence<br>Progress review
Evaluating intervention: enhance adherence*	Sanders, 2011	Antihypertensives	NA	ACEIs to treat essential hypertension	Blood pressure control, mortality, morbidity, safety, specific adverse events, persistence/adherence, rate of use of a single antihypertensive medication for blood pressure control, lipid levels, rates of progression to type 2 diabetes, carbohydrate metabolism/diabetes control, left ventricular mass/function, kidney disease	Primary/health/ social care<br>Secondary health/social care (not hospital)<br>Hospital (outpatient)<br>Other<br>*Setting of studies not restricted*	Choosing a medication<br>Progress review<br>Patient adherence
Evaluating intervention: enhance adherence*	Nili, 2020	Antihypertensives	NA	Describe randomized clinical trial (RCT) interventions using health behavioral models/theories to improve medication adherence among adults with hypertension	Adherence, MMAS, discontinuation rate, MAES, MMAS, TAQPH, Self-report of number of missed pills, re view of prescription renewal patterns Steinberg's Hypertension Self-Efficacy Scale	Primary/health/soci al care<br>Hospital (outpatient)<br>Care home<br>Patient home	Information and/or advice<br>Patient adherence<br>Progress review<br>Maintaining treatmenT

Focus/ Aim	Study (First author, date)	Medications of interest	Other medications included	Intervention name/s and aim	Outcomes measured	Setting/Context	Relevant part of care pathway
Evaluating intervention: enhance adherence*	Mallat, 2016	Antihypertensives	NA	Free versus fixed drug combination. Aim: to increase adherence, increase overall BP-lowering efficacy	Mortality, morbidity (cardiovascular outcomes such as coronary events, stroke, progression of peripheral vascular disease and kidney disease), mean systolic BP, AE, adherence to treatment	NR	Choosing a medication<br>Patient adherence<br>Progress review
Evaluating intervention: enhance adherence*	de Cates, 2014	Statins<br>Antihypertensives	NA	A fixed-dose combination therapy, a combination of several active components into a single pill, including at least one statin and one antihypertensive agent. Aim: to optimise CVD risk and reduce CVD fatal and non-fatal events	Mortality, non-fatal CVD endpoints, AEs (overall rates of discontinuation, proportion of participants experiencing specific symptoms or results and rates of discontinuation by specific symptoms). Secondary outcomes: Systolic and diastolic blood pressure, total and LDL cholesterol, adherence, health-related QOL, costs of fixed-dose combination therapy	Primary/health/ social care	Choosing a medication<br>Progress Review<br>Patient adherence

Focus/ Aim	Study (First author, date)	Medications of interest	Other medications included	Intervention name/s and aim	Outcomes measured	Setting/Context	Relevant part of care pathway
Evaluating intervention: enhance adherence*	Maimaris, 2013	Antihypertensives	NA	National or regional health system arrangements on HT awareness, treatment, control, and antihypertensive medication adherence	HT awareness (persons with clinically measured HT diagnosed by a health care professional as hypertensive), HT treatment (use of at least one antihypertensive medication in an individual with known HT), antihypertensive medication adherence, HT control	Primary/health/ social care<br>Secondary health/social care (not hospital)<br>Hospital (inpatient)<br>Hospital (outpatient)	Identifying health or prescribing concern<br>Accessing care<br>Decision to start agreed treatment plan<br>Patient adherence<br>Other *health systems, infrastructure. System level interventions*
Evaluating intervention: optimise prescribing	Hukins, 2019	Statins	Anticholinergic medications, anxiolytics, oestrogens, NSAID, antipsychotics, antiplatelets, proton pump inhibitors, benzodiazepines Hypnotics/Z-drugs	Any tool (dementia-specific or non-disease-specific) to identify potentially inappropriate prescribing	Prevalence of polypharmacy, prevalence of PIP, most common prescribed PIP medications	Primary/health/ social care<br>Secondary health/social care (not hospital)<br>Hospital (inpatient)<br>Hospital (outpatient)<br>Care home<br>Patient home	Identifying health or prescribing concern<br>Progress review<br>Decision to stop treatment: supported
Evaluating intervention: optimise prescribing*	Pantoja, 2019	Benzodiazepines<br>Hypnotics/Z-drugs<br>Statins<br>Antihypertensives	Anticonvulsants, antibiotic therapy, NSAID, nicotine gum, ezetimibe, thromboprophylaxis	Manual paper reminders to improve compliance with preventive guidelines and disease management guidelines	Patient-important endpoints: death, development of a pulmonary embolism , surrogate or intermediate endpoints: achievement of target blood pressure or serum cholesterol level, markers of disease/health status, adverse effects, resource use, changes in professional practice, patient outcomes	Primary/health /social care<br>Hospital (inpatient)<br>Hospital (outpatient)	Progress review<br>Identifying health or prescribing concern<br>Initial assessment<br>Prescribing guidelines

Shaw L, *et al. BMJ Open* 2024; 14:e072502. doi: 10.1136/bmjopen-2023-072502

Focus/ Aim	Study (First author, date)	Medications of interest	Other medications included	Intervention name/s and aim	Outcomes measured	Setting/Context	Relevant part of care pathway
Evaluating intervention: optimise prescribing*	Legare, 2010	Statins	Not clear	Interventions for improving the adoption of shared decision making by healthcare professionals	Healthcare professionals' adoption of SDM	Primary/health/ social care<br>Secondary health/social care (not hospital)	Initial assessment<br>Information and/or advice<br>Choosing a medication<br>Shared decision making<br>Decision to start agreed treatment plan
Evaluating intervention: optimise prescribing	Jeffery, 2015	Statins<br>Antihypertensives	NA	Interventions to improve adherence to CVD guidelines and patient outcomes: education, audit and feedback, academic detailing, comprehensive interventions that included education, audit and feedback and an academic detailing component	Guideline adherence, clinical outcomes	Primary/health/ social care<br>Secondary health/social care (not hospital)<br>Hospital (inpatient)Hospital (outpatient)	Identifying health or prescribing concern<br>Initial assessment<br>Information and/or advice<br>Prescribing guidelines<br>Progress review
Evaluating intervention: optimise prescribing	Shanbhag, 2018	Antihypertensives	NA	Implementation interventions classified at the provider, organisational or health system levels. Aim: increasing physician adherence to the specified HF guideline recommendations	Patient-level outcomes. Primary outcomes: process indicators (assessing guideline-consistent activities undertaken by a provider, proportion of patients who were: prescribed a guideline-recommended pharmacological treatment, referred for implantable cardioverter defibrillator, CRT consideration, provided self-care education at discharge, LVEF. Secondary outcomes: HF	Hospital (inpatient)<br>Hospital (outpatient)	Prescribing guidelines<br>Decision to start agreed treatment plan<br>Other

Focus/ Aim	Study (First author, date)	Medications of interest	Other medications included	Intervention name/s and aim	Outcomes measured	Setting/Context	Relevant part of care pathway
					specific/all-cause clinical outcomes		
Evaluating intervention: optimise prescribing	Zheng, 2020	Antihypertensives	NA	Nonphysician provider-led interventions: GDMT initiation and target dosage optimization	Primary outcomes: proportion of patients newly initiated into GDMT and the proportion of patients up titrated to target dosages of GDMT, stratified by therapeutic class. Secondary outcomes included clinical endpoints: all-cause mortality and hospitalizations due to HF. GDMT was defined as angiotensin-converting enzyme inhibitor/angiotensin II receptor blocker/angiotensin receptor-neprilysin inhibitor (ACEI/ARB/ARNI or RASI), BB, MRA, and SGLT2I. Outcomes related to GDMT management were collected only for therapeutic classes detailed in study-specific titration protocol	Secondary health/social care (not hospital) Hospital (inpatient) Hospital (outpatient)	Pre-treatment/Initiation Maintaining treatment Prescribing guidelines

*A secondary aim or one of multiple aims of review. Green highlighted text=High Overall Quality AMSTAR-2, Turquoise highlighted text=Medium Overall Quality AMSTAR-2, Orange highlighted text=Low Overall Quality AMSTAR-2, Red highlighted text=Critically-low Overall Quality AMSTAR-2. ACEI= Angiotensin-Converting Enzyme Inhibitors, AE=Adverse Events, AIDS= Acquired Immunodeficiency Syndrome, BEZRA=Benzodiazepines/Zdrugs, BP=Blood Pressure, CBT=Cognitive Behavioural Therapy, CPM=Continuous Passive Motion, CRT= Cardiac Resynchronisation Therapy, CV=Cardiovascular, CVD=Cardiovascular Disease, DBI=Drug Burden Index, ED=Emergency Department, GDR=Gradual Dose Reduction, GP=General Practitioner, HF=Heart Failure, HIV= Human Immunodeficiency Virus, HT=Hypertension, LDL= Low-Density Lipoprotein, LOS=Hospital Length of Stay, NA=Not Applicable, NR=Not Reported, QOL=Quality Of Life, LVEF= Left Ventricular Ejection Fraction, MBCBT=Mindfulness Based Cognitive Behavioural Therapy, NSAID=Non-Steroidal Anti-Inflammatories, PIP=Potentially Inappropriately Prescribed, SAE=Serious Adverse Events, SDM=Shared Decision Making, SMBP=Self-Monitoring of Blood Pressure, TKA=Total Knee Arthroplasty

Table 5: Systematic reviews scoring negatively on one CEESAT item which synthesised studies evaluating the effectiveness of an intervention

Focus/ Aim	Study (First author, date)	Medications of interest	Other medications included	Intervention name/s and aim	Outcomes measured	Setting/Context	Relevant part of care pathway
**Evaluating intervention: deprescribing**	Kornhold, 2022	Statins<br>Antihypertensives	Treatment for: dementia, Parkinson's disease, orthostatic hypotension, dyskinesia, osteoporosis, Pain, LUTS, COPD, Insomnia (Lorazepam), Depression (Sertraline)	Discontinuation	Dropout rate (proportion of patients who did not complete the entire study per protocol in the discontin-uation and continuation groups); Disease recurrence (proportion of patients with disease appearance for preventive treatments (eg, fractures after discontinuation of bisphosphonate treatment) or clinical worsening of the treated dis-ease only for primarily symptomatic treatments (eg, pain after opioid treatment withdrawal) in the discontinuation and continuation group), Adverse drug withdrawal events	Primary/health/social care<br>Hospital (inpatient)<br>Care home	Decision to stop treatment: supported Decision/ability to adhere to deprescribing plan

Shaw L, *et al. BMJ Open* 2024; 14:e072502. doi: 10.1136/bmjopen-2023-072502

Focus/ Aim	Study (First author, date)	Medications of interest	Other medications included	Intervention name/s and aim	Outcomes measured	Setting/Context	Relevant part of care pathway
Evaluating intervention: deprescribing	Page, 2016	Statins Antihypertensives	Clopidogrel, Cilostazol, pentoxifylline, diuretics, nitrates, Glucosamine, bisphosphonates, calcium, vitamin D, Calcitriol, conjugated equine estrogens Premarin combined with medroxyprogesterone acetate, Carbamazepine, Levodopa, lithium, anticholinestera, prednisolone, corticosteroids, d beta-2 receptor agonist, antipsychotics, benzodiazepines antidepressants	Deprescribing by a health care professional of one or more regular prescription medications:	Mortality, adverse drug withdrawal events, physical health, cognitive function and psychological health, quality of life	Hospital (inpatient) Care home Primary/health/ social care Hospital (outpatient) Patient home	Progress review Decision to stop treatment: supported

Focus/ Aim	Study (First author, date)	Medications of interest	Other medications included	Intervention name/s and aim	Outcomes measured	Setting/Context	Relevant part of care pathway
Evaluating intervention: enhance adherence	Park, 2017	Antihypertensives Statins	Antidementia medication, pneumonia-related ambulatory antibiotics, antilpemics, insulin, biologics, oral diabetes drugs, antiasthmatics, pain reliever, phosphate binders, cinacalce, teriparatide, antidepressants	Medicare Part D: increase drug utilization and lower Medicare and Medicaid participants out- of-pocket prescription drug costs, thereby improving access	Out-of-pocket costs, drug utilization	Primary/health/social care Hospital (inpatient) Hospital (outpatient)	Choosing a medication Patient adherence Progress review
Evaluating intervention: enhance adherence	Parati, 2021	Antihypertensives	Na	Single Pill Combination Therapy: determine whether SPC therapy leads to improved adherence, patient persistence, and better BP control compared with FEC therapy in patients with hypertension	adherence, BP	NR	Choosing a medication Patient adherence Maintaining treatmen
Evaluating intervention: enhance adherence	Conn, 2015[70]	Statins Antihypertensives	Yes, in relation to following medical conditions: organ transplant, epilepsy, gastrointestinal, infections, chronic diseases, malaria, STD, neurological, HIV	Packaging interventions to improve adherence: blister packs, unit-packaging, unit-of-use systems, unit-of-dose packaging, monitored dosage systems	Adherence, health outcomes	Primary/health/social care Hospital (inpatient) Patient home	Patient adherence

Focus/ Aim	Study (First author, date)	Medications of interest	Other medications included	Intervention name/s and aim	Outcomes measured	Setting/Context	Relevant part of care pathway
**Evaluating intervention: enhance adherence**	Conn, 2015[71]	Statins *Inferred from population disease characteristics* Antihypertensives *Inferred from population disease characteristics*	Yes, in relation to following medical conditions: diabetes, HIV, renal disease, asthma, lung disease. stroke, gastrointestinal diseases, acute illness, allergies, autoimmune disease, blood disorders, cancer, eye problems, gynaecological disorders, liver disease, malaria, nervous system problems, osteoarthritis, osteoporosis, rheumatoid arthritis, seizures, skin diseases, organ transplant, tuberculosis, and vascular disease	Health care provider targeted interventions to improve medication adherence: Research staff trained providers, integration of care across providers, increased providers, communication skills, monitor patients adherence behaviour, Provider- patient medication concordance, Increased provider time with patients, reduce the geographical distance between providers and patients, increase continuity of care, activating provider	Adherence behaviour	Primary/health/social care	Accessing care Working with other health or social care services/ charities Shared decision making Decision to start agreed treatment plan Patient adherence Progress review

Focus/ Aim	Study (First author, date)	Medications of interest	Other medications included	Intervention name/s and aim	Outcomes measured	Setting/Context	Relevant part of care pathway
Evaluating intervention: enhance adherence	Conn, 2016	Statins<br>Antihypertensives<br>*All Inferred from participant sample characteristics*	Yes, in relation to following medical conditions: gallbladder disease, unspecified chronic illnesses, osteoarthritis, asthma, IBD, glaucoma, kidney transplant, anti-coagulant therapy, osteoarthritis stroke, diabetes, HIV, antidepressants	Medication Adherence Interventions: Intervention content could include: prompts/cues to administer medications, self-monitoring of medication administration, self-monitoring of disease symptoms, written instructions, rewards for increased adherence, increased communication between providers and patients, providing feedback to participants about their adherence, goal setting about adherence, habit assessment/modification, and problem solving about adherence challenges	Adherence	Primary/health/social care<br>Secondary health/social care (not hospital)<br>Patient home	Decision to start agreed treatment plan<br>Patient adherence<br>Progress review
Evaluating intervention: enhance adherence	Coronado-Vazquez, 2020	Statins<br>Antihypertensives	Antidepressants, medications to treat cancer screening and type 2 diabetes	Shared decision making: to balance the patients' right of autonomy with the practitioners' responsibility to protect patients' safety.[2] SDM is a process within the physician–patient relationship applicable to any clinical action, whether diagnostic, therapeutic, or preventive in nature	Adherence to the treatment, knowledge and awareness of the illness, satisfaction of both professionals and patients with the intervention, and decisional conflict	Primary health/social care	Information and/or advice<br>Shared decision making<br>Patient adherence

Focus/ Aim	Study (First author, date)	Medications of interest	Other medications included	Intervention name/s and aim	Outcomes measured	Setting/Context	Relevant part of care pathway
Evaluating intervention: enhance adherence	De Simoni, 2013	Antihypertensives	NA	Interventions to improve adherence to antihypertensive medications: education, lifestyle, verbal information/advice on disease and secondary-prevention drug treatment, goal setting, supply of printed information/advice material, screening for depression, personalized instructions, and integrated care	Adherence and blood pressure control	Primary/health/social care<br>Hospital (outpatient)<br>Patient home	Information and/or advice<br>Decision to start agreed treatment plan<br>Patient adherence<br>Progress review
Evaluating intervention: enhance adherence	Kassavou, 2018	Statins<br>Antihypertensives	Yes, NR	Automated telecommunication systems, including voice messaging and text messaging: promote adherence to cardio-metabolic medications	Adherence	Primary/health/social care<br>Secondary health/social care (not hospital)<br>Hospital (inpatient)<br>Hospital (outpatient)<br>Patient home	Patient adherence
Evaluating intervention: enhance adherence	Krack, 2019	Statins<br>Antihypertensives	Calcium channel blockers, Clopidogrel, insulin oral antidiabetics, diabetes drugs, inhaled steroids, metformin, thiazides, Inhaled corticosteroid	Value-based health insurance designs including: VBID vs usual medication coverage VBID+education vs usual medication coverage, VBID+education vs usual medication coverage+education<br><br>Aim: increase patients understanding of importance of high-value medications, increase number of adherent patients without increasing total health care spending	Adherence	Primary/health/social care	Information and/or advice<br>Patient adherence

Focus/ Aim	Study (First author, date)	Medications of interest	Other medications included	Intervention name/s and aim	Outcomes measured	Setting/Context	Relevant part of care pathway
Evaluating interventio n: enhance adherence	Kronish, 2011	Antihypertensives	NA	Drug Class: improve adherence to antihypertensive medication	Adherence	NR	Choosing a medication Patient adherence
Evaluating Intervention: enhance adherence	Lawrence, 2015	Statins Antihypertensives	Antithrombotic medication	Multimodal behavioural interventions for secondary stroke prevention: addresses: 1) medication education and/or medication compliance education, 2) education or active information provision e.g. about stroke, stroke (lifestyle) risk factors, and 3) one or more of four specified lifestyle behaviours i.e. smoking, diet, physical inactivity, and alcohol consumption, and/or behaviours associated with amelioration of lifestyle risk factors i.e. medication compliance and management of perceived psychosocial stress	Primary outcomes of interest: physiological outcomes e.g. blood pressure, blood lipids, and lifestyle behaviour change. Secondary outcomes: psychosocial outcomes e.g. anxiety, learning e.g. knowledge of lifestyle risk factors for stroke, incidence of vascular events and mortality	Secondary health/social care (not hospital) Patient home	Information and/or advice Patient adherence Progress review
Evaluating intervention: enhance adherence	Mann, 2014	Statins Antihypertensives	NA	Drug Insurance Cost Sharing Strategies: lower expenditures for prescription drug insurance plans	Adherence, clinical events (myocardial infarction, stroke, death), QOL, healthcare utilization, or cost	NR	Accessing care Decision to start agreed treatment plan Patient adherence

Focus/ Aim	Study (First author, date)	Medications of interest	Other medications included	Intervention name/s and aim	Outcomes measured	Setting/Context	Relevant part of care pathway
Evaluating intervention: enhance adherence	Milosavljevic, 2018	Antihypertensives	Yes, in relation to following conditions: Asthma, diabetes, dyslipidemia, COPD, 'older people's health' aldosterone-inhibiting diuretics (for heart failure), intravenous vitamin D analogs, calcimimetic, oral hypoglycemics, antipsychotics, other central nervous system drugs, beta blockers, analgesics, antidepressants	Community pharmacist interventions: improve adherence, improve clinical outcomes.	Behavioural outcome: adherence, self-efficacy. Clinical outcome: clinical biomarkers (e.g. blood pressure, glycosylated haemoglobin, LDL), hospitalisation rates, mortality, emergency room visits, markers of disease progress. Economic outcome: cost effectiveness analysis, or other relevant analysis. Humanistic outcome: patient quality of life, knowledge, or satisfaction	Primary/health/social care Patient home	Information and/or advice Choosing a medication Decision to start agreed treatment plan Progress review Patient adherence

Focus/ Aim	Study (First author, date)	Medications of interest	Other medications included	Intervention name/s and aim	Outcomes measured	Setting/Context	Relevant part of care pathway
Evaluating intervention: enhance adherence	Rash, 2016	Statins	NA	Interventions targeting medication adherence:<br>Prescription cost coverage, simplification of drug regimen, reminders, education-based, multi-faceted interventions (including one or more of following: education, counselling, behavioural interventions, follow-up, medication review, risk-factor and adherence education, personalized health reports, bi-monthly mailings, collaborative care)	Patient adherence	Primary/health/social care<br>Secondary health/social care (not hospital)<br>Hospital (inpatient)<br>Hospital (outpatient)	Information and/or advice<br>Working with other health/social care services<br>Choosing a medication<br>Shared decision making<br>Patient adherence<br>Progress review
Evaluating intervention: enhance adherence	Reeves, 2020	Antihypertensives	NA	Pharmacist-led interventions to improve medication adherence and BP control	BP control, medication adherence	Primary/health/social care<br>Hospital (inpatient)<br>Hospital (outpatient)	Information and/or advice<br>Patient adherence<br>Progress review
Evaluating intervention: enhance adherence	Vervloet, 2020	Statin<br>Antihypertensives	Anticoagulants	Mix: improve adherence	Adherence	NR<br>Primary care	Information and/or advice<br>Patient adherence<br>Progress review<br>Maintaining treatment
Evaluating intervention: enhance adherence and prescribing	Xie, 2022	Statin<br>Antihypertensive	Antibiotics	Clinical dashboards: change clinician or patient behaviour	1) medication use, including the rate of medication prescribed/administered and medication intake adherence; (2) test ordering, such as the rate of imaging referrals and the count of routine laboratory test orders	Primary health/social care	Initial assessment<br>Prescribing guidelines<br>Patient adherence<br>Maintaining treatment

Shaw L, *et al. BMJ Open* 2024; 14:e072502. doi: 10.1136/bmjopen-2023-072502

Focus/ Aim	Study (First author, date)	Medications of interest	Other medications included	Intervention name/s and aim	Outcomes measured	Setting/Context	Relevant part of care pathway
Evaluating intervention: enhance adherence*	Cheema, 2014	Antihypertensives	NA	Interventions to improve management of BP: Pharmacological: education on drug treatment of BP, advice to patients to improve medication adherence, identifying drug adverse effects and drug prescribing issues, liaising with prescribers about concerns of drug treatment. Nonpharmacological: education about hypertension. education about lifestyle (advice to patients on diet, weight management, alcohol consumption, smoking cessation)	Systolic and diastolic BP, adherence, identification and management of drug-related problems, cardiovascular risk factors, i.e. smoking, alcohol consumption	Primary/health/social care	Information and/or advice<br>Working with other health/social care services<br>Identifying health or prescribing concern<br>Patient adherence<br>Progress review
Evaluating intervention: optimise prescribing	Nguyen, 2018	Statins	Aspirin, antiplatelet agents	Organisational or professional interventions to enhance prescribing of guideline-recommended medications. Interventions included: Distribution of educational materials, educational outreach visits, audit and feedback and reminders, continuity of care, communication and case discussions between distant healthcare professionals, distribution of published/printed recommendations for clinical care, including clinical practice guidelines	Proportion of patients receiving guideline-recommended medications. Secondary outcomes: proportion of patients achieving target blood pressure and target LDL, C/cholesterol level, mortality rate	Primary/health/social care<br>Hospital (outpatient)	Identifying health or prescribing concern<br>Initial assessment<br>Information and/or advice<br>Choosing a medication<br>Prescribing guidelines

Focus/ Aim	Study (First author, date)	Medications of interest	Other medications included	Intervention name/s and aim	Outcomes measured	Setting/Context	Relevant part of care pathway
Evaluating intervention: optimise prescribing<br>Evaluating intervention: enhance adherence	King, 2018	Statins<br>Antihypertensives	Diabetic medication, Digoxin, mixed medications, antidepressants	Longer-duration (2–4 months) versus shorter-duration (28-day)prescriptions to improve patient outcomes	Health outcomes, adverse events, medication adherence, medication wastage, professional administration time, pharmacists time and/or costs, patient experience, and patient out-of-pocket costs	Primary/health/social care<br>Secondary health/social care (not hospital)<br>Hospital (inpatient)	Initial assessment<br>Choosing a medication<br>Decision to start agreed treatment plan<br>Patient adherence<br>Progress review

*Secondary aim or outcome within the review. AE=Adverse Events, BI=Behavioural Interventions, BP=Blood Pressure, BZ=Benzodiazepine, CBT=Cognitive Behavioural Therapy, COPD=Coronary Obstructive Pulmonary Disease, DBT=Dialectical Behavioural Therapy, ED=Emergency Department, GP=General Practitioner, HIV= Human Immunodeficiency Virus, IBD=Irritable Bowel Disease, LDL= Low-Density Lipoprotein, LTOT=Long-Term Opioid Therapy, MDD=Major Depressive Disorder, MI=Motivational Interviewing, NA=Not Applicable, NR=Not Reported, QOL=Quality of Life, VBID=Value Based Insurance Design

Table 6: Systematic reviews scoring negatively on 2-4 CEESAT items which synthesised studies evaluating the effectiveness of an intervention

Focus/ Aim	Study (First author, date)	Medications of interest	Other medications included	Intervention name/s and aim	Outcomes measured	Setting/Context	Relevant part of care pathway
Evaluating intervention: deprescribing*.	Hart, 2020	Antihypertensives	Antipsychotics, Antihistamines, Antivertigo, Anti-Parkinson, Urinary spasmolytics, Vasodilators, Digoxin, Diuretics, Beta Blockers, Hypoglycemics, Beta blocker eye drops, Alpha adrenergic antagonists, Alpha blockers, Benzodiazepines Opiates Hypnotics/Z-drugs Antidepressants	Interventions to reduce falls or use of FRIDs: Face-to-face medication consultations with community pharmacy resident, fall-related assessment performed by research physician, geriatrician assessment of falls risk and systematic medication review, consideration of potential FRIDs for withdrawal+prescribing physician contact	Change in FRID use	Hospital (inpatient) Hospital (outpatient)	Initial assessment Choosing a medication Progress review Decision to stop treatment: supported Other *Medication review in acute care (e.g. after hip fracture)*
Evaluating interventions: deprescribing	Chambergo-Michilot, 2023	Antihypertensives	No	NR	Discontinuation	Hospital (inpatient)	Discontinuing treatment

Focus/ Aim	Study (First author, date)	Medications of interest	Other medications included	Intervention name/s and aim	Outcomes measured	Setting/Context	Relevant part of care pathway
Evaluating intervention: enhance adherence	Al Alshaikh, 2016	Statins Antihypertensives	Aspirin, Dipyridamole, antithrombotics, antiplatelets, anticoagulants, diabetic medications	Multimodal Interventions to improve adherence: categories included educational+motivational, simplification of drug regimen, environmental cues or reminders+reducing concerns and misbeliefs regarding medications, combined interventions to enhance risk factor management after stroke, prescribed medications+educated about them	Medication adherence	Primary/health/social care<br>Hospital (inpatient)<br>Hospital (outpatient)<br>Patient home	Information and/or advice<br>Patient adherence
Evaluating intervention: enhance adherence	Baumgartner, 2020	Statins Antihypertensives	Yes, in relation to following medical conditions:<br>HIV, diabetes, tuberculosis<br><br>Specific medications include: metformin/sulfonylurea, various oral antidiabetic drugs, pioglitazone/ thiazolidinedione+ sulfonurea, FDC: rosiglitazone/glimepiride, glyburide/metformin, thiazolidinedione, Î±-blocker/antimuscarinic	Polypill to improve medication adherence	Adherence	Primary/health/social care<br>Hospital (inpatient)<br>Hospital (outpatient)	Choosing a medication<br>Decision to start agreed treatment plan<br>Patient adherence<br>Progress review

Focus/ Aim	Study (First author, date)	Medications of interest	Other medications included	Intervention name/s and aim	Outcomes measured	Setting/Context	Relevant part of care pathway
Evaluating intervention: enhance adherence	Bochkareva, 2019	Antihypertensives	NA	Medical Assistance Scheme to increase adherence to treatment: Examples include special packaging of medications, amount of prescribed medications, interventions to improve communication with patients (more frequent visits, motivational interviewing, patient education, home BP monitoring, and provision of written instructions), duration and number of sessions/consultations	Adherence	Primary/health/social care Secondary health/social care (not hospital) Hospital (outpatient)	Information and/or advice Patient adherence
Evaluating intervention: enhance adherence	Bond, 2021	Statins	No	Mobile health: improve adherence	Adherence	Hospital (outpatient) *Assumed* Patient home	Information and/or advice Patient adherenc

Shaw L, *et al. BMJ Open* 2024; 14:e072502. doi: 10.1136/bmjopen-2023-072502

Focus/ Aim	Study (First author, date)	Medications of interest	Other medications included	Intervention name/s and aim	Outcomes measured	Setting/Context	Relevant part of care pathway
Evaluating intervention: enhance adherence	Checchi, 2014	Antihypertensives<br>Statins	Various HAARTs, luticasone propionate, travoprost, pilocarpine, antidiabetic medications, ipratropium bromide, lithium, Clopidogrel, bupropion-SR, antipsychotics, fluticasone, sulfonylureas, immuno-suppressants	Electronic medication packaging devices to promote medication adherence	Adherence, health outcomes, cost effectiveness results, and satisfaction	Primary/health/social care<br>Patient home	Patient adherence<br>Information and/or advice
Evaluating intervention: enhance adherence	Conn 2015[36]	Antihypertensives	NA	Interventions to increase adherence among adults with hypertension	Adherence	Primary/health/social care<br>NR	Information and/or advice<br>Patient adherence<br>Progress review<br>Referral to specialist care

Focus/ Aim	Study (First author, date)	Medications of interest	Other medications included	Intervention name/s and aim	Outcomes measured	Setting/Context	Relevant part of care pathway
Evaluating intervention: enhance adherence	Deichmann, 2016	Statins	Fibrates, Anion-exchange resins or bile acid sequestrants, Niacin or nicotinic acid, combined medication regimen	Interventions to increase adherence to self-administered lipid-lowering medication: Drug regimen simplification, patient education and information, intensified patient care with reminders via mail, telephone, and hand-held pill devices, complex behavioural approaches, group sessions, decision support systems, administrative improvements, pharmacy-led automated telephone intervention	Adherence to lipid-lowering medical therapy. Secondary outcomes: physiologic indicators (e.g., total cholesterol), health outcome indications (e.g., QOL, morbidity, mortality), adverse effects, implications for costs	Primary/health/social care<br>Patient home	Information and/or advice<br>Choosing a medication<br>Patient adherence<br>Progress review
Evaluating intervention: enhance adherence	Du, 2018	Antihypertensives	NA	Fixed-dose combination versus free-equivalent combination therapies on adherence to medication for hypertension	Medication adherence/persistence	NR	Choosing a medication<br>Patient adherence<br>Progress review
Evaluating intervention: enhance adherence	Gupta, 2010	Antihypertensives	NA	Fixed-Dose Combinations of Antihypertensive agents. Aim: to achieve better BP control by improving compliance	Compliance (or adherence), persistence, BP-lowering efficacy, adverse effects	NR	Choosing a medication<br>Patient adherence<br>Progress review

Shaw L, *et al. BMJ Open* 2024; 14:e072502. doi: 10.1136/bmjopen-2023-072502

Focus/ Aim	Study (First author, date)	Medications of interest	Other medications included	Intervention name/s and aim	Outcomes measured	Setting/Context	Relevant part of care pathway
Evaluating intervention: enhance adherence	Guzman-Tordecilla, 2020	Antihypertensives	NA	Interventions to enhance adherence and control of arterial hypertension: community fund organization, reminders, educational material and calendar, sessions for the optimization of the use of medicines, advice with experienced pharmacists, group educational meetings, obtention of medical records, identification of problems with medications and intervention on them, follow-up with the pharmacist and education	Adherence, control of HBP	Primary/health/social care<br>Patient home	Information and/or advice<br>Working with other health or social care services/charities<br>Patient adherence<br>Progress review
Evaluating intervention: enhance adherence	Hong, 2022	Antihypertensives	No	Mix: influence decisions of the patients with CHD on their adherence to antihypertensive medications	Adherence	Primary/health/ social care<br>Hospital (inpatient)<br>Hospital (outpatient)	

Focus/ Aim	Study (First author, date)	Medications of interest	Other medications included	Intervention name/s and aim	Outcomes measured	Setting/Context	Relevant part of care pathway
Evaluating interventio n: enhance adherence	Kawalec, 2018	Antihypertensives	NA	Fixed-dose combination therapy. Aim: blood pressure lowering, increasing patients adherence	Medication adherence, blood pressure lowering, persistence, health outcomes and adverse events	Primary/health/soci al care<br>Patient home	Choosing a medication<br>Patient adherence<br>Progress review
-Evaluating intervention: enhance adherence	Leslie, 2016	Antihypertensives<br>Statins (assumed)	Oral diabetes medications	Interventions aimed to modify medication adherence behaviour to chronic medications administered orally to treat diabetes, hypertension, or hyperlipidaemia: behavioural counselling, education and Informational materials, medication packaging that increased convenience by combining pills in a blister pack	Adherence to medications	Primary/health/soci al care<br>Care home<br>Patient home	Information and/or advice<br>Patient adherence<br>Progress review
Evaluating intervention: enhance adherence	Mamudu, 2014	Statins<br>Antihypertensives	Diabetic medications, Aspirin	Coronary artery calcium screening: enable risk stratification, diagnosis of CAD and directs the treatment and management of CAD among asymptomatic individuals	Behavioural modification, risk perception, and medication adherence, lifestyle change	Primary/health/soci al care	Identifying health or prescribing concern<br>Initial assessment<br>Patient adherence
Evaluating interventio n: enhance adherence	Tsioufis, 2020	Antihypertensives	NA	Single-pill combination therapy to treat hypertension. Aim: improve clinical outcomes and medication adherence	Adherence and/or persistent, BP, cardiovascular outcomes, mortality or morbidity	Primary/health/soci al care	Choosing a medication<br>Patient adherence<br>Progress review

Focus/ Aim	Study (First author, date)	Medications of interest	Other medications included	Intervention name/s and aim	Outcomes measured	Setting/Context	Relevant part of care pathway
Evaluating intervention: enhance adherence	Uhlig, 2013	Antihypertensives	NA	Interventions to enhance management of hypertension: self-measured blood pressure with or without additional support	Clinical outcomes (e.g. death and cardiovascular events), patient-reported outcomes (e.g. patient satisfaction or QOL), surrogate outcomes (e.g. measures of left ventricular hypertrophy), intermediate outcomes (e.g. BP; number, dose, or changes of antihypertensive medications; adherence to antihypertensive medication), health care utilization (including visits, calls, and e-mails	Primary/health/soci al care<br>Patient home	Patient adherence<br>Progress review
Evaluating intervention: enhance adherence	Xiong, 2018	Antihypertensives	Immunosuppressant's (n=1), lipid-lowering medication (n=1)	Mobile health technology to enhance medication adherence	Medication adherence	Primary/health/soci al care<br>Secondary health/social care (not hospital)<br>Hospital (outpatient)<br>Patient home	Patient adherence
Evaluating intervention : enhance adherence	Xu, 2018	Antihypertensives	NA	Medication education, self-monitoring of BP, reminders and regular follow-up visits to improve medication adherence	Adherence, BP, BP control	Primary/health/soci al care<br>Hospital (outpatient) *might be inpatient*<br>Patient home	Patient adherence<br>Information and/or advice<br>Progress review

Shaw L, *et al. BMJ Open* 2024; 14:e072502. doi: 10.1136/bmjopen-2023-072502

Focus/ Aim	Study (First author, date)	Medications of interest	Other medications included	Intervention name/s and aim	Outcomes measured	Setting/Context	Relevant part of care pathway
Evaluating intervention: enhance adherence	Demonceau, 2013	Antihypertensives *Assumed*	Medications for following medications:: HIV, asthma, osteoporosis Specific medications: sulfonylurea drugs, oral nitrates	Interventions to affect adherence to self-administered prescribed medications in short-term and in long-term therapy: treatment simplification, cognitive-educational interventions, behavioural counselling, social-psycho-affective interventions, electronically monitored adherence feedback, technical reminder systems, monitoring the disease being managed, rewards, integrating depression treatment with hypertension management or type 2 diabetes care, home-based physician-directed, nurse-guided drug therapy, drug regimen	Health outcomes' e.g. reductions in blood pressure, lower levels of glycosylated haemoglobin, medication adherence	Primary/health/social care<br>Hospital (inpatient)<br>Hospital (outpatient)<br>Patient home	Information and/or advice<br>Working with other health/social care services<br>Patient adherence<br>Referral to specialist care
Evaluating intervention: enhance adherence<br>Evaluating intervention: optimise prescribing	Laba, 2013	Statins<br>Antihypertensives	Yes<br>*Not all medicines reported, but all for CVD* e.g. spironolactone, loop diuretics, digoxin	Strategies to improve patient adherence to cardiovascular medications:<br>education, persuasion, incentivisation, enablement, training, coercion, restriction, environmental restructuring, modelling, policies, communication/marketing, guidelines, fiscal, regulation, legislation, environmental/social planning, service provision	Adherence, Physiological: BP control, mean DBP/SBP, lipids, glucose/HbA1c, INR, echocardiogram, health outcomes, health service utilisation, mortality, QOL, other lifestyle, smoking/alcohol, nutrition, physical activity, stress, adverse events, cost comparison, cost effectiveness	Primary/health/social care<br>Patient home<br>Hospital (outpatient)	Information and/or advice<br>Shared decision making<br>Patient adherence<br>Progress review<br>Prescribing guidelines

Shaw L, *et al. BMJ Open* 2024; 14:e072502. doi: 10.1136/bmjopen-2023-072502

Focus/ Aim	Study (First author, date)	Medications of interest	Other medications included	Intervention name/s and aim	Outcomes measured	Setting/Context	Relevant part of care pathway
**Evaluating intervention: enhance adherence**	Polinski, 2011	Statins<br>Antihypertensives	HEDIS, high risk drugs, anti-convulsants, antihistamines, propoxyphene, digoxin, NSAIDs, doxazosin, muscle relaxants/anti-spasmodics, others, warfarin, oral anti-diabetics, antiretroviral medications, warfarin, clopidogrel, antipsychotics, proton pump inhibitors, antibiotics, benzodiazepines antidepressants, hypnotics/Z-drugs (sedatives)	Medicare Part D cost-sharing provisions and drug coverage rules. Aim: improve access to essential medications through reduced cost-sharing with focus on under and over-use of specific drugs and classes	Reduced out-of-pocket costs, health	Primary/health/social care<br>Hospital (outpatient)	Accessing care<br>Choosing a medication<br>Decision to start agreed treatment plan<br>Patient adherence<br>Decision to stop treatment: unsupported

Focus/ Aim	Study (First author, date)	Medications of interest	Other medications included	Intervention name/s and aim	Outcomes measured	Setting/Context	Relevant part of care pathway
Evaluating intervention: optimise prescribing	Kang, 2016	Statins<br>Antihypertensives	Aspirin	Pharmacist-involved care (patient- targeted services) and healthcare professional-targeted services: PTS-counselling, providing educational materials, medical reminders, post-discharge follow- up services and supporting rehabilitation transition care. HTS-medication reconciliation, communication with health-care providers<br>Aim: improve CVD outcomes	All-cause mortality, all-cause hospitalization, cardiac-related hospitalization, key medication prescription rates, health-related QOL, medication adherence, medication utilization	Primary/health/social care<br>Hospital (inpatient)<br>Hospital (outpatient)<br>Patient home	Information and/or advice<br>Working alongside health/social care services<br>Choosing a medication<br>Patient adherence<br>Progress review
Evaluating intervention: optimise prescribing	Chhina, 2013	Antihypertensives	Propoxyphene, cerebral & peripheral vasodilators, cephalexin, amoxycillin with or without clavulinic acid, cephalexin, doxycycline, erythromycin, penicillin, rrimethoprim, cefaclor, roxithromycin, NSAIDS, metronidazole, omeprazole, inhaled steroids, antipsychotic, benzodiazepines antidepressants	Academic Detailing to modify drug prescription behaviour of Family Physicians in primary care settings	Prescription rate	Primary/health/social care	Choosing a medication<br>Progress review<br>Other<br>*Reducing repeat prescription*

Focus/ Aim	Study (First author, date)	Medications of interest	Other medications included	Intervention name/s and aim	Outcomes measured	Setting/Context	Relevant part of care pathway
Evaluating intervention: optimise prescribing* Evaluating intervention: enhance adherence*	Yuan, 2019	Antihypertensives	Yes, associated with following medical conditions: diabetes, chronic obstructive pulmonary disease, osteoporosis, obesity, antiphospholipid syndrome, drug related high risk of disease, outpatients taking multiple prescription drugs, bronchial asthma	Community Pharmacist interventions to improve outcomes for people with hypertension: medication review, patient education, adherence assessment, health/lifestyle advice, physical assessment (e.g., BP), monitoring, prescribing, or adjusting and administering therapy	Clinical outcomes of pharmaceutical services e.g. common end- points of systolic blood pressure, diastolic blood pressure and glycosylated haemoglobin after providing CPS	Primary/health/social care<br>Patient home<br>Hospital (inpatient)	Initial assessment<br>Information and/or advice<br>Decision to start agreed treatment plan<br>Patient adherence<br>Progress review

*Secondary aim or one of multiple aims; [a]Note that focus is on 'use' which includes initiating prescribing. Drugs of interest may be currently under- or over-used, [b]One aspect of broad aim, [c]One aspect of interventions. BP=Blood Pressure, CAD=Coronary Artery Disease, CPS=Community Pharmacist Services, CVD=Cardiovascular Disease, COX-2=Cyclooxygenase-2, DBP-Diastolic Blood Pressure, FDC=Fixed-Dose Combination, FRID=Fall Risk Increasing Drugs, HAART= Highly Active Antiretroviral Therapy, HBP=High Blood Pressure, HIV= Human Immunodeficiency Virus, HRT=Hormonal Replacement Therapy, HTS=Healthcare professional Targeted Services, INR=International Normalised Ratio, NA=Not Applicable, NR=Not Reported, NSAID=Non-Steroidal Anti-Inflammatories,PTS=Patient Targeted Services, SBP=Systolic Blood Pressure, SH=Sedative Hypnotics, SR=Sustained Release, QOL=Quality of Life,

Shaw L, *et al. BMJ Open* 2024; 14:e072502. doi: 10.1136/bmjopen-2023-072502

Table 7: Systematic reviews synthesising qualitative evidence

Study (First author, date)	Medications of interest	Other medications included	Perspectives obtained	Phenomenon of Interest	Setting/Context	Relevant part of care pathway
Ingersgaard, 2020	Statins<br>Antihypertensives	Lipid-lowering agents, Î² blockers, angiotensin, renin system blockers, aspirin	Patient	Experiences/views of an intervention (adherence)<br>Reasons for adherence/non-adherence	Primary/health/social care<br>NR	Patient adherence
Rashid, 2014	Statins	Clopidogrel, antiplatelet medication, aspirin	Patient<br>Family member	Reasons for adherence/non-adherence	Primary/health/social care<br>Hospital (outpatient)<br>Secondary health/social care (not hospital)<br>Patient home	Patient adherence<br>Decision to stop treatment: unsupported

Study (First author, date)	Medications of interest	Other medications included	Perspectives obtained	Phenomenon of Interest	Setting/Context	Relevant part of care pathway
Kinnear, 2019	Statins	NA	Patient<br>Carer<br>Family member	Experiences/views of an intervention (adherence)<br>Reasons for adherence/non-adherence	Primary/health/social care<br>Secondary health/social care (not hospital)	Initial assessment<br>Information and/or advice<br>Choosing a medication<br>Shared decision making<br>Patient adherence<br>Progress review<br>Decision to stop treatment: unsupported
Reeve, 2013	Antihypertensives	Any long-term medication, methadone (substitution program), antiepileptic's, HRT, PPIs, donepezil, benzodiazepines antidepressants	Patient<br>Carer	Experiences/views of an intervention (deprescribing)	NR	Decision to stop treatment: supported
Dhar, 2017	Antihypertensives	NA	Patient	Reasons for adherence/non-adherence	Primary/health/social care<br>Secondary health/social care (not hospital)<br>Hospital (outpatient)<br>Patient home	Patient adherence<br>Decision to stop treatment: unsupported

Study (First author, date)	Medications of interest	Other medications included	Perspectives obtained	Phenomenon of Interest	Setting/Context	Relevant part of care pathway
Marshall, 2012	Antihypertensives	NA	Patient	Reasons for adherence/non-adherence	Primary/health/social care<br>Hospital (outpatient)<br>Patient home	Choosing a medication<br>Decision to start agreed treatment plan<br>Patient adherence<br>Decision to stop treatment: supported<br>Decision to stop treatment: unsupported
Khatib, 2014	Antihypertensives	NA	Practitioner/Prescriber<br>Patient	Prescribing<br>Of an intervention (adherence)<br>Reasons for adherence/non-adherence	Primary/health/social care<br>Hospital (outpatient)<br>Patient home	Identifying health or prescribing concern<br>Accessing care<br>Initial assessment<br>Information and/or advice<br>Choosing a medication<br>Shared decision making<br>Prescribing guidelines<br>Decision to start agreed Patient adherence<br>Progress review

Study (First author, date)	Medications of interest	Other medications included	Perspectives obtained	Phenomenon of Interest	Setting/Context	Relevant part of care pathway
Oori, 2019	Antihypertensives	NA	Patient	Reasons for adherence/non-adherence	Care home<br>Primary/health/social care-<br>Secondary health/social care (not hospital)<br>Hospital (outpatient)<br>Patient home	Patient adherence

Shaw L, et al. BMJ Open 2024; 14:e072502. doi: 10.1136/bmjopen-2023-072502

Study (First author, date)	Medications of interest	Other medications included	Perspectives obtained	Phenomenon of Interest	Setting/Context	Relevant part of care pathway
Rashid, 2018	Antihypertensives	Yes, relevant to the following conditions: asthma, renal transplant, proton pump inhibitors, schizophrenia, migraine, antiretroviral for HIV, osteoporosis, anxiety, malaria prophylaxis, bipolar, back pain, ADHD, dysphagia, IBD, RA, breast cancer, anticoagulants, borderline personality disorder,<br>Specific drugs mentioned: clozapine, sumatriptan, SSRI, HAART, malaria prophylaxis, antibiotics, phosphate binding, insulin, clopidogrel, PMTCT, chemotherapy, Aripiprazole, Antidepressants	Practitioner/Prescriber<br>Patient<br>Carer<br>Family member	Experiences/views of an intervention (adherence)<br>Taking a medication	Primary/health/social care<br>Secondary health/social care (not hospital)<br>Hospital (inpatient)<br>Hospital (outpatient)	Accessing care<br>Initial assessment<br>Information and/or advice<br>Patient adherence<br>Progress review

Shaw L, *et al. BMJ Open* 2024; 14:e072502. doi: 10.1136/bmjopen-2023-072502

Study (First author, date)	Medications of interest	Other medications included	Perspectives obtained	Phenomenon of Interest	Setting/Context	Relevant part of care pathway
Qadi, 2020	Statins<br>Antihypertensives	No	Practitioner/Prescriber<br>Patient	Prescribing medication<br>Taking medication	Primary health/social care	Initial assessment<br>Decision to start agreed treatment plan

* Data too thin for synthesis. Green highlighted text=High Overall Quality as appraised by AMSTAR-2, Turquoise highlighted text=Moderate Overall Quality as appraised by AMSTAR-2.
ADHD=Attention-Deficit Hyperactivity Disorder, BZ=Benzodiazepine, HAART= Highly Active Antiretroviral Therapy, HIV= Human Immunodeficiency Virus, IBD=Irritable Bowel Disease, NA=Not Applicable, NR=Not Reported, PMTCT=Prevention of Mother-to-Child Transmission, RA=Rheumatoid Arthritis, SSRI=Selective Serotonin Reuptake Inhibitor

Shaw L, *et al. BMJ Open* 2024; 14:e072502. doi: 10.1136/bmjopen-2023-072502

APPENDIX E: QUALITY APPRAISAL OF INCLUDED STUDIES

Table 8: Scores on CEESAT items included studies

First Author, Date	Search strategy: Is approach to searching clearly defined, systematic and transparent?	Is search comprehensive?	Does the review critically appraise each study?	During critical appraisal was an effort made to minimise subjectivity?	Total Score
Dills, 2018	Green	Green	Green	Green	0
Hukins, 2019	Green	Green	Green	Green	0
Pantoja, 2019	Green	Green	Green	Green	0
Posadzki, 2016	Green	Green	Green	Green	0
Stacey, 2017	Green	Green	Green	Green	0
Adler, 2017	Green	Green	Green	Green	0
de Cates, 2014	Green	Green	Green	Green	0
Fletcher, 2015	Green	Green	Green	Green	0
Jeffery, 2015	Green	Green	Green	Green	0
Kinnear, 2019	Green	Green	Green	Green	0
Legare, 2010	Green	Green	Green	Green	0
Maimaris, 2013	Green	Green	Green	Green	0
Mallat, 2016	Green	Green	Green	Green	0
Morrissey, 2017	Green	Green	Green	Green	0
Nieuwlaat, 2014	Green	Green	Green	Green	0
Nili, 2020	Green	Green	Green	Green	0
Palmer, 2018;2020	Green	Green	Green	Green	0
Sanders, 2011	Green	Green	Green	Green	0
Shanbhag, 2018	Green	Green	Green	Green	0
van Driel, 2016	Green	Green	Green	Green	0
Zheng, 2023	Green	Green	Green	Green	0
King, 2018	Green	Green	Amber	Green	1
Milosavljevic, 2018	Green	Green	Amber	Green	1
Page, 2016	Green	Green	Amber	Green	1
Park, 2017	Green	Green	Amber	Green	1

Shaw L, *et al. BMJ Open* 2024; 14:e072502. doi: 10.1136/bmjopen-2023-072502

Cheema, 2014	Green	Green	Amber	Green	1
Conn, 2015[38]	Amber	Green	Green	Green	1
Conn, 2015[40]	Amber	Green	Green	Green	1
Conn, 2016	Amber	Green	Green	Green	1
Coronado- Vázquez, 2020	Green	Green	Amber	Green	1
de Simoni, 2013	Green	Amber	Green	Green	1
Ingersgaard, 2020	Green	Amber	Green	Green	1
Kornholt 2022	Green	Amber	Green	Green	1
Kassavou, 2018	Green	Green	Green	Red	1
Krack, 2019	Green	Green	Green	Red	1
Kronish, 2011	Green	Green	Amber	Green	1
Lawrence, 2015	Green	Green	Green	Red	1
Mann, 2014	Green	Green	Green	Red	1
Nguyen, 2018	Green	Amber	Green	Green	1
Parati, 2021	Green	Green	Green	Amber	1
Qadi, 2020	Green	Green	Amber	Green	1
Rash, 2016	Green	Green	Green	Red	1
Reeve,s 2020	Amber	Green	Green	Green	1
Reston, 2020	Green	Green	Amber	Green	1
Van Truong	Green	Amber	Green	Green	1
Verveloet, 2020	Green	Amber	Green	Green	2
Xie, 2022	Green	Green	Green	Amber	1
Loganathan, 2011	Green	Amber	Amber	Green	2
Bond, 2021	Green	Green	Amber	Ambeo	2
Mamudu, 2014	Green	Green	Red	Red	2
Oori, 2019	Amber	Green	Amber	Green	2
Xu, 2018	Green	Amber	Amber	Green	2
Checchi, 2014	Green	Amber	Green	Red	2
Demonceau, 2013	Green	Amber	Amber	Green	2
Rashid, 2018	Green	Amber	Amber	Green	2
Reeve, 2013	Green	Green	Amber	Red	2
Baumgartner, 2020	Green	Amber	Green	Red	2
Bochkareva, 2019	Green	Green	Amber	Red	2
Deichmann, 2016	Amber	Green	Amber	Green	2

Hong 2020	Green	Amber	Green	Amber	2
Khatib, 2014	Green	Amber	Green	Red	2
Laba, 2013	Green	Green	Amber	Red	2
Rashid, 2014	Green	Amber	Amber	Green	2
Tsioufis, 2020	Green	Amber	Green	Red	2
Uhlig, 2013	Green	Amber	Amber	Green	2
Yuan, 2019	Amber	Amber	Green	Green	2
Hart, 2020	Amber	Amber	Amber	Green	3
Polinski, 2011	Amber	Amber	Amber	Green	3
Al Alshaikh, 2016	Amber	Amber	Amber	Green	3
Conn, 2015	Amber	Green	Amber	Red	3
Dhar, 2017	Amber	Green	Amber	Red	3
Gupta, 2010	Amber	Amber	Amber	Green	3
Guzman-Tordecilla, 2020	Amber	Amber	Green	Red	3
Kang, 2016	Amber	Amber	Amber	Green	3
Marshall, 2012	Green	Amber	Amber	Red	3
Xiong, 2018	Green	Amber	Amber	Red	3
Du,2019	Amber	Amber	Amber	Red	4
Chhina, 2013.	Amber	Amber	Amber	Red	4
Kawalec, 2018	Amber	Amber	Amber	Red	4
Leslie, 2016[7]	Amber	Amber	Amber	Red	4

BMJ Publishing Group Limited (BMJ) disclaims all liability and responsibility arising from any reliance placed on this supplemental material which has been supplied by the author(s)

Table 9: Scores on AMSTAR-2 tool for reviews included in map with CEESAT score of 0

Study (First author, date)	1. PICO components	2. Protocol	3. Study design explanation	4. Comprehensive search strategy	5. Duplicate study selection	6. Duplicate data extraction	7. Details of excluded studies	8. Description of included studies	9b. RoB assessment (NRSIs)	10. Funding sources	9a. Risk of Bias (RoB) assessment (RCTs)	11a. RCTs Meta-analysis	11b. NRSIs Meta-analysis (MA)	12. MA: RoB in individual studies	13. Qualitative reviews: Is synthesis methodology /theoretical framework clearly described /justified?	14. Qualitative reviews: was description /rationale for sampling described?	15. Qualitative reviews: was process of data extraction clearly described?	16. Qualitative reviews: was process for coding data clearly described?	17. Qualitative reviews: was process of developing themes clearly described?	18. Qualitative reviews: Do authors reflect on how their theoretical /ideological perspectives may influence their findings?	19 Qualitative reviews: Was use of any software clearly described?	20. RoB: discussion of results	21. Heterogeneity	22. Publication bias	23. Reports conflicts of interest	Overall rating
Adler, 2017	Y	Y	N	Y	Y	Y	Y	Y	NA	Y	Y	N	NA	NA	NA	NA	NA	NA	NA	NA	NA	Y	Y	NA	Y	H
de Cates, 2014	Y	PY	N	Y	Y	Y	Y	Y	NA	N	Y	Y	NA	Y	NA	NA	NA	NA	NA	NA	NA	Y	Y	Y	Y	H
Dills 2018,	Y	N	N	Y	Y	Y	N	Y	NA	Y	Y	N	NA	NA	NA	NA	NA	NA	NA	NA	NA	Y	Y	NA	Y	L
Fletcher, 2015	Y	Y	Y	Y	Y	Y	N	PY	N	N	Y	Y	N	Y	NA	NA	NA	NA	NA	NA	NA	Y	Y	N	Y	CL
Hukins, 2019	Y	N	N	Y	Y	Y	N	Y	Y	N	NA	NA	N	NA	NA	NA	NA	NA	NA	NA	NA	Y	Y	NA	Y	L
Jeffery, 2015	Y	Y	N	Y	Y	Y	N	Y	Y	N	Y	Y	Y	Y	NA	NA	NA	NA	NA	NA	NA	Y	Y	N	Y	M
Kinnear, 2019	Y	Y	NA	Y	Y	Y	N	Y	Y	Y	NA	NA	N	NA	PY	NA	Y	Y	Y	N	Y	Y	Y	NA	Y	H
Legare, 2010	Y	Y	N	Y	Y	Y	Y	Y	NA	N	Y	N	NA	NA	NA	NA	NA	NA	NA	NA	NA	Y	Y	NA	Y	H
Maimaris, 2013	Y	Y	Y	Y	Y	Y	N	PY	PY	N	Y	N	N	NA	NA	NA	NA	NA	NA	NA	NA	Y	Y	NA	Y	M
Mallat, 2016	Y	Y	N	Y	Y	Y	N	Y	NA	Y	Y	Y	NA	Y	NA	NA	NA	NA	NA	NA	NA	Y	Y	Y	Y	H
Morrissey, 2017	Y	Y	N	Y	Y	Y	N	Y	NA	N	Y	N	NA	Y	NA	NA	NA	NA	NA	NA	NA	Y	Y	Y	Y	L

Shaw L, et al. BMJ Open 2024; 14:e072502. doi: 10.1136/bmjopen-2023-072502

Study (First author, date)	1. PICO components	2. Protocol	3. Study design explanation	4. Comprehensive search strategy	5. Duplicate study selection	6. Duplicate data extraction	7. Details of excluded studies	8. Description of included studies	9b. RoB assessment (NRSIs)	10. Funding sources	9a. Risk of Bias (RoB) assessment (RCTs)	11a. RCTs Meta-analysis	11b. NRSIs Meta-analysis (MA)	12. MA: RoB in individual studies	13. Qualitative reviews: Is synthesis methodology /theoretical framework clearly described /justified?	14. Qualitative reviews: was description /rationale for sampling described?	15. Qualitative reviews: was process of data extraction clearly described?	16. Qualitative reviews: was process for coding data clearly described?	17. Qualitative reviews: was process of developing themes clearly described?	18. Qualitative reviews: Do authors reflect on how their theoretical /ideological perspectives may influence their findings?	19 Qualitative reviews: Was use of any software clearly described?	20. RoB: discussion of results	21. Heterogeneity	22. Publication bias	23. Reports conflicts of interest	Overall rating
Nieuwlaat, 2014	Y	Y	N	Y	Y	Y	Y	Y	NA	N	Y	N	NA	NA	NA	NA	NA	NA	NA	NA	NA	Y	Y	NA	Y	M
Nili, 2020	Y	PY	N	PY	Y	N	N	PY	NA	Y	Y	NA	NA	NA	NA	NA	NA	NA	NA	NA	NA	N	N	NA	Y	CL
Palmer, 2018	Y	Y	Y	Y	Y	Y	Y	Y	NA	Y	Y	N	NA	NA	NA	NA	NA	NA	NA	NA	NA	Y	Y	NA	Y	H
Palmer	Y	Y	Y	Y	Y	Y	Y	Y	Y	Y	Y	Y	NA	Y	NA	NA	NA	NA	NA	NA	NA	Y	Y	Y	Y	H
Pantoja, 2019	Y	Y	N	Y	Y	Y	Y	Y	Y	Y	Y	N	N	NA	NA	NA	NA	NA	NA	NA	NA	Y	Y	NA	Y	H
Posadzki, 2016	Y	Y	Y	Y	Y	Y	Y	Y	Y	Y	Y	Y	Y	Y	NA	NA	NA	NA	NA	NA	NA	Y	Y	N	Y	H
Sanders, 2011	Y	N	N	Y	Y	Y	Y	Y	PY	Y	PY	Y	Y	N	NA	NA	NA	NA	NA	NA	NA	N	Y	N	Y	CL
Shanbhag, 2018	Y	Y	N	Y	Y	Y	N	PY	Y	N	Y	N	N	NA	NA	NA	NA	NA	NA	NA	NA	N	Y	NA	Y	L
Stacey, 2017	Y	Y	N	Y	Y	Y	Y	Y	NA	N	Y	Y	NA	Y	NA	NA	NA	NA	NA	NA	NA	Y	Y	Y	Y	M
van Driel, 2016	Y	Y	N	Y	Y	Y	Y	Y	NA	Y	Y	Y	NA	Y	NA	NA	NA	NA	NA	NA	NA	Y	Y	Y	Y	H
Zheng, 2023	Y	Y	N	Y	Y	Y	Y	Y	Y	N	Y	Y	NA	Y	NA	NA	NA	NA	NA	NA	NA	Y	Y	Y	N	H

CL=Critically Low overall quality; H=High overall quality; Low overall quality; M=Moderate overall quality; N=No; NA=Not Applicable; NRSI=Non-Randomised Studies of healthcare Interventions; PICO=Population, Intervention, Comparator, Outcome; PY=Partial Yes; RCT=Randomised Controlled Trial; RoB=Risk of Bias; Y=Yes

Shaw L, *et al. BMJ Open* 2024; 14:e072502. doi: 10.1136/bmjopen-2023-072502
