# Supplementary material for: What is the quantity, quality and type of systematic review evidence available to inform the optimal prescribing of statins and antihypertensives? A systematic umbrella review and evidence and gap map
Source: BMJ Open. 2024 Feb 24;14(2):e072502. doi: 10.1136/bmjopen-2023-072502 (PMC10895245; doi:10.1136/bmjopen-2023-072502)
Supplement: Author's manuscript [file bmjopen-2023-072502.draft_revisions.pdf]

BMJ Open is committed to open peer review. As part of this commitment we make the peer review history of every article we publish publicly available.

When an article is published we post the peer reviewers' comments and the authors' responses online. We also post the versions of the paper that were used during peer review. These are the versions that the peer review comments apply to.

The versions of the paper that follow are the versions that were submitted during the peer review process. They are not the versions of record or the final published versions. They should not be cited or distributed as the published version of this manuscript.

BMJ Open is an open access journal and the full, final, typeset and author-corrected version of record of the manuscript is available on our site with no access controls, subscription charges or pay-per-view fees (http://bmjopen.bmj.com).

If you have any questions on BMJ Open's open peer review process please email info.bmjopen@bmj.com

BMJ Open

What is the quantity, quality and type of systematic review evidence available to inform the optimal prescribing of statins and antihypertensives? A systematic umbrella review and evidence and gap map

Journal:	*BMJ Open*
Manuscript ID	bmjopen-2023-072502
Article Type:	Original research
Date Submitted by the Author:	10-Feb-2023

Complete List of Authors:	Shaw, Liz; University of Exeter, Exeter Policy Research Programme Evidence Review Facility<br>Briscoe, Simon; University of Exeter, Exeter Policy Research Programme Evidence Review Facility<br>Nunns, Michael; University of Exeter, Exeter Policy Research Programme Evidence Review Facility<br>Lawal, Hassanat; University of Exeter, Exeter Policy Research Programme Evidence Review Facility<br>Melendez-Torres, G.J; University of Exeter, Exeter Policy Research Programme Evidence Review Facility<br>Turner, Malcolm; University of Exeter, Exeter Policy Research Programme Evidence Review Facility; University of Exeter, NIHR ARC South West Peninsula Patient and Public Engagement group<br>Garside, Ruth ; University of Exeter, Exeter Policy Research Programme Evidence Review Facility; University of Exeter, European Centre for Environment and Health<br>Thompson Coon, Jo; University of Exeter, Exeter Policy Research Programme Evidence Review Facility

Keywords:	Systematic Review, Hypertension < CARDIOLOGY, STROKE MEDICINE

SCHOLARONE™
Manuscripts

I, the Submitting Author has the right to grant and does grant on behalf of all authors of the Work (as defined in the below author licence), an exclusive licence and/or a non-exclusive licence for contributions from authors who are: i) UK Crown employees; ii) where BMJ has agreed a CC-BY licence shall apply, and/or iii) in accordance with the terms applicable for US Federal Government officers or employees acting as part of their official duties; on a worldwide, perpetual, irrevocable, royalty-free basis to BMJ Publishing Group Ltd ("BMJ") its licensees and where the relevant Journal is co-owned by BMJ to the co-owners of the Journal, to publish the Work in this journal and any other BMJ products and to exploit all rights, as set out in our licence.

The Submitting Author accepts and understands that any supply made under these terms is made by BMJ to the Submitting Author unless you are acting as an employee on behalf of your employer or a postgraduate student of an affiliated institution which is paying any applicable article publishing charge ("APC") for Open Access articles. Where the Submitting Author wishes to make the Work available on an Open Access basis (and intends to pay the relevant APC), the terms of reuse of such Open Access shall be governed by a Creative Commons licence – details of these licences and which Creative Commons licence will apply to this Work are set out in our licence referred to above.

Other than as permitted in any relevant BMJ Author's Self Archiving Policies, I confirm this Work has not been accepted for publication elsewhere, is not being considered for publication elsewhere and does not duplicate material already published. I confirm all authors consent to publication of this Work and authorise the granting of this licence.

For peer review only

What is the quantity, quality and type of systematic review evidence available to inform the optimal prescribing of statins and antihypertensives? A systematic umbrella review and evidence and gap map

Liz Shaw[1*], Simon Briscoe[1], Michael Nunns[1], Hassanat Lawal[1], G.J. Melendez-Torres[1], Malcolm Turner[1,2], Ruth Garside[1,3], Jo Thompson Coon[1],

[1]Exeter Policy Research Programme Evidence Review Facility, Faculty of Health and Life Sciences, St Lukes Campus, University of Exeter, Exeter, Devon, EX1 2LU, UK

[2] NIHR ARC South West Peninsula Patient and Public Engagement group, Faculty of Health and Life Sciences, St Lukes Campus, University of Exeter, Exeter, Devon, EX1 2LU

[3] European Centre for Environment and Health, Faculty of Health and Life Sciences, Knowledge Spa, Truro, TR1 3HD

*Corresponding author: E.H.Shaw@exeter.ac.uk, 01392 72 2396

Keywords: Statins, antihypertensives, mapping review

Word count (Excluding abstract, strengths/limitations, tables, figures, reference and appendices): 3254

Number of Figures: 3

Number of Tables: 2

For peer review only

ABSTRACT

Objectives: This systematic umbrella review aimed to map the systematic review evidence available to inform the optimal prescribing of medication to treat cardiovascular disease.

Design: Systematic umbrella review and evidence and gap map.

Methods: Eight bibliographic databases (Cochrane Database of Systematic Reviews, CINAHL, EMBASE, Health Management Information Consortium, MEDLINE ALL, PsychInfo, Conference Proceedings Citation Index – Science and Science Citation Index) were searched from 2010 to 11th August 2020. Update searches conducted in MEDLINE ALL 2nd August 2022. We searched relevant websites and conducted backwards citation chasing. Title and abstract and full text screening and data extraction were conducted independently by two reviewers. Quality appraisal was completed by one reviewer and checked by a second. Review characteristics were tabulated and incorporated into an evidence and gap map based on a patient care pathway. Patients with experiences of being prescribed statins or antihypertensives provided feedback on our research questions and evidence and gap map.

Results: Sixty-eight reviews were included within the evidence and gap map, with the highest quantity of evidence focusing on evaluating interventions to promote patient adherence to antihypertensive medication. Key gaps included a lack of reviews synthesising evidence on experiences of specific interventions to promote patient adherence or improve prescribing practice. The evidence was predominantly of low quality, limiting the confidence which can be placed in the findings from individual reviews.

Conclusions: This evidence and gap map provides an accessible format for policy developers, service commissioners and clinicians to view the systematic review evidence available relevant to optimising the prescribing of statin and antihypertensive medication. Its interactive format means that map users can tailor the map's contents to suit their individual needs. To address the paucity of high-quality research, future systematic reviews should be conducted and reported according to existing guidelines and address the evidence gaps identified above.

For peer review only - http://bmjopen.bmj.com/site/about/guidelines.xhtml

STRENGTHS AND LIMITATIONS

- This evidence and gap map will be a useful resource for decision makers who are seeking systematic review evidence to answer questions relating to the optimal prescribing of statins or antihypertensive medication. The map displays key characteristics of the evidence, including an indication of systematic review quality, and allows map users to access an evidence summary and links to the full text as suited to their needs and interests.
- The evidence and gap map includes only systematic review evidence and thus there may be primary research relevant to the topic which may sit within the identified gaps.
- Our search strategy did not capture systematic reviews which may have met our eligibility criteria that were used to inform NICE guidelines relevant to the review topic. These reviews were not retrieved by our bibliographic database searches, highlighting issues regarding the visibility of the reviews contributing to NICE guidelines.

For peer review only

INTRODUCTION

With an estimated 17.9 million deaths during 2019, cardiovascular disease (CVD) is the leading cause of death worldwide.[1] Within the UK, CVD causes one in four premature deaths and costs the NHS seven billion pounds per year,[2] with individuals from black, Asian and other minority ethnic backgrounds, low socio-economic status or those living with diabetes or severe mental illness being more at risk of the disease.[2, 3] Along with earlier detection of risk factors for CVD and promotion of lifestyle changes, the NHS Long Term Plan aims to increase initiation of preventative treatment for CVD.[4] High intensity statins are recommended over the use of low intensity statins as an effective treatment to prevent CVD, although the prescribing of this type of medication has not yet reached optimal levels.[5]

Hypertension is another risk factor in the development of CVD,[6] and can be controlled through use of antihypertensive medication, sometimes in combination with statins, to reduce the occurrence of CVD.[7] Whilst, the proportion of untreated hypertension in England has decreased between 2003 and 2018,[8] there are early signs that awareness and treatment of hypertension within the UK has decreased within the last five years.[9, 10] Patient non-adherence is also an issue of concern, with adherence decreasing with higher numbers of antihypertensive medications prescribed per patient.[11]

To achieve optimal prescribing of statins and antihypertensive medication, it is essential that the factors influencing both their prescription and patient adherence are fully understood. There is already a diverse range of existing systematic review evidence in this area which could be utilised by policy makers, commissioners and clinicians to design and deliver services or research to support the needs of those living with, or at risk of, CVD. To fully understand and make use of this existing evidence base, this paper focuses on the creation of an evidence and gap map to display the quantity, quality and type of quantitative and qualitative systematic review evidence available to inform the optimal prescribing of statins and antihypertensives.

We were interested in systematic reviews which synthesised the following:

- Evidence regarding the effectiveness or experiences of interventions intended to improve prescribing practices or patient adherence;
- Evidence on the effectiveness or experiences of interventions intended to improve implementation of interventions intended to improve prescribing practices or patient adherence;
- Evidence focusing on practitioner views or perceptions of making prescribing decisions;
- Guidelines intended to inform prescribing practise.

For peer review only - http://bmjopen.bmj.com/site/about/guidelines.xhtml

This paper is part of a broader systematic mapping review commissioned by NHS England and Improvement relating to the optimising of the prescribing of three different groups of medication: medications to treat cardiovascular disease, DCD (including opioids, benzodiazepines, gabapentinoids and non-benzodiazepine hypnotics) and antidepressants.[12]

For peer review only

For peer review only - http://bmjopen.bmj.com/site/about/guidelines.xhtml

METHODS

The methods used for the searching, screening, data extraction and quality appraisal processes of the evidence included in this evidence and gap map were in accordance with the University of York's Centre for Reviews and Dissemination guidelines.[13] We also consulted guidance for the development of evidence and gap maps.[14] Our methods were outlined in our pre-registered review protocol and are summarised below according to PRISMA reporting guidance.[15, 16]

Search strategy

Our bibliographic database search strategy combined terms for optimising prescribing with terms for statins and antihypertensives and CVD. The search strategy was developed using MEDLINE (via Ovid) using a combination of free-text terms and controlled headings. Search terms were derived from pre-identified relevant papers and inspected by stakeholders with expertise on optimising prescribing. Searches were run on 11th August 2020 in the following bibliographic databases: Cochrane Database of Systematic Reviews (via Cochrane Library), CINAHL (via EBSCO), EMBASE (via Ovid), Health Management Information Consortium (via Ovid), MEDLINE ALL (via Ovid), PsychInfo (via Ovid), Conference Proceedings Citation Index – Science and Science Citation Index (both via Web of Science, Clarivate Analytics). We also searched systematic review database Epistemonikos and preprint server medRxiv. An historical date limit of 2010 was applied to all search results. We carried out backwards citation chasing for all reviews which met our inclusion criteria, searched topically relevant websites and pursued full texts for conference abstracts and review protocols identified through our searches. We also contacted review authors where we could not access full-texts. Update searches were conducted in MEDLINE ALL (via OVID) on 2nd August 2022. We limited our update search to MEDLINE since all the studies identified through our original search were indexed in this database. Full details of database search strategies are reproduced in Appendix A (which also includes search terms for drugs that cause dependency, which was a second topic that our client asked us to investigate in our mapping review. The findings associated with this second topic are available elsewhere).

Study selection

A sample of 100 title and abstracts were double screened by two reviewers (LS, MN), before the revised inclusion criteria (Table 1), were applied independently by two reviewers to each citation (LS, MN), with disagreements resolved through discussion or referral to a third reviewer (SB). Eligibility of full texts were assessed using the same method. Screening decisions were recorded in Endnote X8

For peer review only - http://bmjopen.bmj.com/site/about/guidelines.xhtml

software (Clarivate Analytics, Philadephia, PA, USA) and the study selection process detailed within a PRISMA-style flowchart.

Table 1: Review inclusion criteria

PICO/PICo criteria	Inclusion criteria
Population	Mean age ≥ 16<br>Receiving/being considered for a prescription for statins and/or antihypertensives.
Intervention/Phenomenon of interest	For systematic reviews of quantitative evidence, interventions which aimed to improve one or more of the following:<br>- Adherence to prescribed medication of interest;<br>- Prescriber adherence to clinical guidance for prescribing;<br>- Prescriber practices;<br>- Implementation of an intervention to enhance patient adherence or prescriber practices.<br><br>Interventions could be conducted at the level of system or that of the patient/prescriber.<br><br>For systematic reviews of qualitative evidence, review foci could include the views or experiences of patients, carers or clinicians for:<br>- Healthcare consultations to discuss initiation, reviewing or discontinuing a prescription;<br>- Interventions to improve adherence/prescribing practice;<br>- Reasons for adherence or non-adherence to prescribed medication;<br>- Making prescribing decisions.
Comparator/Context	Any
Outcome	Relevant to the aims of our review and stated clearly in the study abstract.
Study design	Systematic reviews which met the following criteria:<br>- Clearly stated research question;<br>- Indicate which sources were searched;<br>- Reproducible search strategy and search date;<br>- Clearly defined inclusion and exclusion criteria and study selection methods;<br>- Critically appraise and report the quality of included studies;<br>- Reproducible data synthesis strategy.<br><br>Encompasses: systematic reviews of quantitative and qualitative literature, systematic reviews of reviews, systematic reviews of guidelines, scoping and rapid reviews.

For convenience, we use the term “patient adherence” throughout this report to encapsulate this continual decision-making process in which the patient can choose, and/or be enabled, to play an active role when taking their medication as prescribed.

For peer review only - http://bmjopen.bmj.com/site/about/guidelines.xhtml

Data extraction and quality appraisal

Following a pilot of five reviews by two reviewers, the data extraction and quality appraisal form was applied to all included systematic reviews by one reviewer (MN, LS) and checked by a second (SB, LS, MN), supported by EPPI-Reviewer (v 4.11.5.2) software. Disagreements were resolved through discussion or referral to a third person.

Due to the high number of studies eligible for inclusion and the variation in the rigour and/or transparency of reporting of searching and critical appraisal methods across the included systematic reviews, we deviated from our protocol and conducted quality appraisal in two stages. Reviews were first appraised using four modified criteria from the Collaboration for Environmental Evidence Synthesis Assessment Tool (CEESAT):[17]

1. Search strategy: Is the approach to searching clearly defined, systematic and transparent?
2. Is the search comprehensive?
3. Does the review critically appraise each study?
4. During critical appraisal was an effort made to minimise subjectivity?

Only systematic reviews which met all four criteria were prioritised for full quality appraisal using the AMSTAR-2,[18] modified to incorporate reporting standards for qualitative evidence synthesis.[19] Reviews appraised using only the CEESAT criteria were given an overall quality rating of 'Critically-Low". Full details of the data extracted from each review, including CEESAT and AMSTAR-2 criteria, are provided elsewhere.[12]

Data analysis and presentation

Details of the systematic reviews included within the evidence and gap map were tabulated within a study interview table and described narratively. We used EPPI-Mapper software to structure the evidence and gap map according to the medication of interest and the part of the patient care pathway it may inform.[20]

The initial care pathway was based upon NICE guidelines for statins and antihypertensives, optimising prescribing and shared decision making.[5, 21-23] To ensure the pathways reflected both recommended practice and implementation within 'real-life' settings, we also drew upon a systematic review exploring patient experiences of medication taking and consulted with patients and carers with experience of seeking, being prescribed, taking and/or discontinuing statins and/or antihypertensives.[24] We also asked clinicians if the pathway we developed reflected their clinical

For peer review only - http://bmjopen.bmj.com/site/about/guidelines.xhtml

experience working within the area. Finally, we produced a condensed version of the map which focused on pre-treatment/initiation, maintenance, discontinuation and development of guidelines.

The map presents the systematic review evidence according to the 'Overall Quality' rating provided by the AMSTAR-2. The colour and size of the bubble indicate the quality rating and number of systematic reviews for that medication type at that part of the care pathway respectively. Systematic reviews relevant to more than one type of medication or position on the care pathway are represented more than once within the map. Filters also allow the map user to alter the type of evidence shown in the map. Policy, clinical and patient stakeholders were asked to comment on the presentation and content of the final evidence and gap map to ensure findings were accessible to our intended audiences.

Studies identified through update searches were only included in the map if they provided evidence for parts of the care pathway where there was insufficient evidence.

Patient and public involvement

We worked alongside a group of stakeholders with a variety of expertise in the topic area throughout the production of this evidence and gap map. Stakeholders included representatives from NHSE-I, NICE, clinicians and people with experience of taking one or more of the medications of interest. Key stages at which all stakeholders, including patients and members of the public, contributed during the project included; development of the protocol, including providing feedback on the appropriateness of review questions, supporting development of the bibliographic database search strategies and list of relevant topic websites to be searched. Clinical stakeholders and patients/members of the public also provided insight on the list of included studies, including resolving queries regarding eligibility for inclusion, supported the development of the patient care-pathway and also provided feedback on the presentation of the evidence and gap map and gaps in systematic review evidence within the evidence and gap map and how findings should be disseminated.

For peer review only - http://bmjopen.bmj.com/site/about/guidelines.xhtml

RESULTS

Following deduplication, 1623 unique records were found through our bibliographic database and supplementary searches. 1429 of these were excluded during title and abstract screening, with a further 108 excluded after the reviewing of full-texts. Fourteen systematic reviews were identified through the search strategy intended to identify other medication groups as part of a wider piece of work (ref withheld for peer review). In total, 80 systematic reviews were eligible for inclusion in the review. The 68 studies identified via our original searches are presented within our evidence and gap map (access here, also shown in Figure 1). The 12 studies identified from our update searches related to parts of the care pathway where there was sufficient evidence and are therefore not included in the map but summarised in Appendix B. Figure 2 summarises the study selection process and reasons for exclusion at full-text, with a list of full reasons for exclusion provided in Appendix C.

[Insert Figure 1]

[Insert Figure 2]

Medications of interest within the included reviews were: statins (n=9),[25-32, 93, 101] antihypertensives (n=37),[33-65, 95, 96, 99, 102] and statins and antihypertensives (n=32).[66-92, 94, 98, 100, 103, 104] Nine of the reviews synthesised qualitative evidence,[27, 31, 39, 46, 51, 54, 55, 56,75, 100] four of which also included quantitative evidence in a mixed evidence synthesis.[39, 46, 54, 56] Of the reviews synthesising quantitative evidence evaluating interventions, interventions focused on: enhancing patient adherence to medication as prescribed (n=57),[25, 30, 32-34, 36- 38, 40-43, 45, 47, 49, 50, 52, 53, 57, 58, 60-73, 78-86, 88, 90-99, 101-103] deprescribing a prescribed medication (n=3),[44, 74, 87] or optimising the prescribing of medication to meet the needs of the patient (n=13).[26, 28, 29, 35, 48, 59, 65, 76, 77, 79, 81, 89, 104] Three of these reviews were judged by reviewers to have dual aims.[65, 79, 81] An overview of the characteristics of all reviews included in the evidence and gap map is included in Appendix D.

Nineteen of the included systematic reviews were fully appraised using the modified AMSTAR-2 tool Eight were 'High' quality,[27, 28, 50, 66, 73, 88, 89, 92] five were of 'Moderate' quality,[32, 49, 60, 76, 86] four of 'Low' quality,[26, 53, 59, 74] and two 'Critically-Low' quality.[41,58] The remaining 49 reviews scored poorly on one or more of the CEESAT criteria during the first stage of quality appraisal, and were thus awarded a 'Critically-Low' overall quality rating. The full quality appraisal ratings for each review can be found in Appendix E.

Table 2 provides a summary of the systematic review evidence included in the evidence and gap map.

For peer review only - http://bmjopen.bmj.com/site/about/guidelines.xhtml

Table 2: Summary of included reviews

Topic/type of evaluation	Type of evidence	Number of reviews*	Appraised using AMSTAR-2 Y/N: N (Overall Quality rating)	Medication of interest
Prescriber, patient, and/or family/carer views of issues relating to prescribing and/or adherence to medications of interest	Qualitative	9*[1]	Y: 1<br>(High: 1)	Statins: 1
			N: 8	AH:6<br>Statins: 1<br>Statins+AH: 1
Evaluating intervention to optimise prescribing	Quantitative	12	Y: 5<br>(High 2:, Moderate:1, Low: 2)	AH: 1<br>Statins: 2<br>Statins+AH: 2
			N: 7	AH:3<br>Statins: 1<br>Statins+AH: 3
Evaluating intervention to deprescribe medication	Quantitative	3	Y: 1<br>(Critically low: 1)	Statins+AH: 1
			N: 2	AH: 1<br>Statins+AH: 1
Evaluating intervention to optimise patient adherence to a medication	Quantitative	47	Y: 12<br>(High: 6, Moderate: 3, Low: 1, Critically low: 2)	AH: 7<br>Statins: 1<br>Statins+AH: 4
			N: 35	AH: 17<br>Statins: 2<br>Statins+AH: 16

*n>68 as some reviews were coded as having multiple aims; *[1]Four reviews also included quantitative evidence; AH=Antihypertensives; N=No; Y=Yes

Summary of evidence on the patient care pathway

Figure 3 presents a summary of the evidence provided within each segment of the evidence and gap map based on the foci and/or aims of interest of this evidence and gap map. Further detail is summarised below.

[Insert Figure 3]

Summary of evidence and gaps

Figure 3 illustrates a large cluster of evidence relating to the evaluation of interventions to promote maintaining adherence to medication, particularly to treat hypertension. The next biggest cluster of evidence is relevant to the 'Pre-treatment' or 'Initiation' stage of the pathway with antihypertensive medication. However, overall the quality of the evidence at these segments of the care pathway was predominantly of critically-low quality.

The quantity of qualitative evidence available is low and, whilst the evidence examines the perceptions of patients, carers and/or clinicians of issues affecting medication prescription or

For peer review only

adherence, none of the systematic reviews explore experiences of specific interventions intended to optimise prescribing in some way. Only one of the systematic reviews of qualitative evidence was of 'High' quality.

Pre-treatment/Initiation

Four reviews synthesised qualitative research.[27, 31,46, 51] One high quality review of qualitative evidence explored patient and family members views of the barriers and enablers to treatment adherence for heterozygous familial hypercholesterolaemia.[27] Three reviews of 'Critically Low' quality review synthesised qualitative evidence relevant to the prescribing of antihypertensives.[46, 31, 51] Two reviews included a range of different perspectives,[31, 46] whilst one focused on patient perspectives.[51]

Twenty-nine systematic reviews synthesised quantitative evidence to promote medication adherence. Statins were the medication of interest for nine reviews, two of 'High' overall quality,[73, 92] and seven of 'Critically Low' overall quality.[68, 79, 82, 84, 85, 90, 91] Antihypertensives were the medication of interest in twenty systematic reviews; three were of 'High' quality,[50, 73, 92] two of 'Moderate' overall quality,[49, 60] and fifteen were 'Critically Low' overall quality.[34, 40, 42, 45, 47, 52, 61, 65, 68, 79, 82, 84, 85, 90, 91] Eight reviews sat across both statin and antihypertensive care pathways.[68, 73, 79, 82, 84, 85, 91, 92]

Six systematic reviews synthesised quantitative evidence evaluating interventions to optimise prescribing, where the medication of interest was statins; 'High' quality (n=2),[28, 89] 'Moderate' quality (n=1),[76] and 'Critically Low' quality (n=3).[29, 77, 79] One 'Critically Low' quality review synthesised quantitative evidence evaluating interventions to reduce the prescribing of fall risk increasing drugs in older adults.[44]

Maintaining treatment

One high quality,27 and three 'Critically-low' quality,[31,46, 51] reviews of qualitative evidence, included in the 'Pre-treatment/initiation' part of pathway, were also relevant here. In addition, one 'Critically-Low' quality review explored reasons associated with adherence or non-adherence to statin medication,[56] and a further three 'Critically-Low' quality reviews combined quantitative and qualitative data to explore factors relating to adherence/nonadherence where antihypertensives and/or statins were the medication of interest.[16, 24, 48]

Twenty-four systematic reviews synthesised quantitative evidence where statins were the medication of interest. Five were of 'High' overall quality,[2, 13, 50, 54, 65] one of 'Moderate' overall quality,[47] and 18 of 'Critically Low' overall quality synthesised quantitative evidence to promote

For peer review only - http://bmjopen.bmj.com/site/about/guidelines.xhtml

adherence. Forty-four systematic reviews synthesised quantitative evidence evaluating interventions to promote adherence to medications, which included antihypertensives.[25, 30, 67--72, 78-85, 90, 91]

Five were of ‘High’ overall quality,[50, 66, 73, 88, 92] three were ‘Moderate’ quality,[49, 60, 86] three were of ‘Critically low’ quality,[41, 53, 58] and thirty-three 33 were of critically-low quality.[33, 34, 36-38, 40, 42, 43, 45, 47, 52, 57, 61-65, 67-69, 71-73, 78-85, 90, 91]

Three ‘Critically Low’ quality reviews synthesised quantitative evidence to optimise the prescribing of medications, including statins and antihypertensives.[77, 79, 81] Two further ‘Critically Low’ quality reviews synthesised evidence evaluating interventions to optimise the prescribing of antihypertensives alone.[35, 65]

Discontinuing treatment

Three reviews, one of ‘Low’ quality,74 two of ‘Critically Low’ quality,[44, 87] synthesised quantitative evidence evaluating interventions promote deprescribing. All of these reviews had a broad remit, focusing on deprescribing medications for chronic diseases,[74] and older adults.[44, 87]

One systematic review of quantitative evidence evaluated impact of Medicare Part D on use of under and overuse of medication. Statins and antihypertensives were only two types of medication included in this review.[91]

One review of ‘Low’ quality focused on identifying potentially inappropriate prescribing in older adults with dementia.[26]

Two ‘Critically-Low’ quality reviews of qualitative evidence; one lay perspectives on hypertension and drug adherence,[51] one reported on patient and/or carer views barriers to and enablers of deprescribing where antihypertensives were the medication of interest.56

Two ‘Critically Low’ quality reviews synthesised quantitative evidence to optimise the prescribing of antihypertensives.[35, 48]

Prescribing guidelines

One ‘Moderate’ quality review, relevant to both the prescribing of statins and antihypertensives, synthesised quantitative evidence evaluating interventions to improve adherence to CVD guidelines.[76]

For peer review only - http://bmjopen.bmj.com/site/about/guidelines.xhtml

DISCUSSION

We aimed to provide a resource to enable policy makers, commissioners, clinicians and other decision makers to find and view existing systematic review evidence relevant to optimising the prescribing of statin and antihypertensive medication. The evidence and gap map displays systematic review evidence synthesising quantitative evidence evaluating interventions to improve or optimise medication adherence or prescribing and also qualitative evidence examining the perspectives of patients, carers and clinicians of issues which can affect optimal prescribing. This evidence is mapped onto a patient care pathway, according to the type of medication of interest, enabling map users to find and view the evidence most suited to their needs.

The map highlights a cluster of systematic review evidence evaluating interventions to promote adherence to medication, particularly antihypertensive, relevant to the “Pre-treatment” and “Maintaining treatment” sections of the patient care pathway. An identified gap within the pathway is that of qualitative evidence which seeks to explore the views and/or experiences of these types of intervention.

Strengths and limitations

This evidence and gap map will be a useful resource for decision makers who are seeking systematic review evidence to answer questions relating to the optimal prescribing of statins or antihypertensive medication. The maps functionality means that the key characteristics of the evidence are easily displayed and allow for map users to access a summary or link to full texts as required. The map also identifies both clusters of existing systematic review evidence and gaps, highlighting where further systematic review evidence may or may not be required. However, this map includes only systematic review evidence and thus there may be primary research relevant to the topic which may sit within the identified gaps. In addition, the majority of evidence in this map was of critically-low quality. These systematic reviews may be of more limited use to decision makers and mask gaps where further evidence syntheses or commissioning of primary research is required.

Our search strategy did not capture systematic reviews used to inform NICE guidelines relevant to the review topic. These reviews were not retrieved by our bibliographic database searches, highlighting issues regarding the visibility of the reviews contributing to NICE guidelines. It was beyond our resources to search through individual guidelines to locate relevant reviews.

For peer review only - http://bmjopen.bmj.com/site/about/guidelines.xhtml

Implications of our findings

For research commissioners, the map highlights where further research may be useful, including the following specific areas:

- Further synthesis of existing systematic reviews of experiences of taking or adhering to statin and/or antihypertensive medication;
- Searching for primary studies where gaps in high quality systematic review evidence have been identified, for example, qualitative evidence regarding patient and/or prescriber experiences of specific interventions to increase medication adherence or practitioner experiences of prescribing statin medication.

The clusters of existing research may also be useful in informing government policy, however caution is required as many of the systematic reviews are of poor quality which may limit the confidence that can be placed in their findings.

For clinicians, the qualitative evidence within the map highlights factors which could influence patient's seeking out or accessing health services for CVD and their willingness or ability to take any medication which is prescribed to them. This may be helpful in informing clinical practice. The map also includes systematic review evidence evaluating interventions to optimise medication adherence, which may also be useful to inform clinical practice.

CONCLUSIONS

This evidence and gap map presents a snapshot of the systematic review evidence available to inform the optimal prescribing of statin and antihypertensive medication, displayed to indicate the quality and quantity of evidence available to inform decision making at key points in the patient care pathway. The evidence included within the map may support policy makers and service commissioners or aid in the commissioning of further research. The map may also help inform the prescribing decisions of clinicians. Whilst some caution is required when interpreting the content of the map given the limitations of the included evidence and methodology used within this mapping review as indicated above, this map provides a useful resource for a variety of stakeholders to access recent systematic review evidence in this topic area.

ACKNOWLEDGEMENTS

Sue Whiffin and Jenny Lowe for administrative support. Morwenna Rodgers for support developing the protocol and search strategy. Kirstin Liabo for support developing the protocol and facilitating involvement of patients and members of the public with the review. The PenARC Patient Engagement Group for their insight throughout the review process. Dr Phil Evans for his support

For peer review only - http://bmjopen.bmj.com/site/about/guidelines.xhtml

developing the patient care pathways and feedback on the draft evidence and gap map. Melissa Bond from the EPPI centre for continued support in developing the evidence and gap map.

CONTRIBUTION OF AUTHORS

Liz Shaw: Lead author. Led scoping process and contributed to the development of the protocol and conduct of screening, data extraction, quality appraisal. Led development of the evidence-and-gap map, patient and public involvement and write up of paper. Supported liaison with government and clinical stakeholders.

Simon Briscoe: Information specialist and reviewer. Supported scoping and led to the development of the search strategy to inform the protocol. Contributed to screening, data extraction and quality appraisal and was responsible for data management throughout the project. Developed PRISMA diagram and contributed to all stakeholder involvement and write up of paper.

Michael Nunns: Reviewer. Supported development of the protocol and contributed to screening, data extraction, quality appraisal and write up of paper.

Hassanat Lawal: Reviewer. Supported screening, data extraction and quality appraisal and reviewed final paper.

G.J. Melendez-Torres: Supported conceptualisation of the project and development of the protocol. Supported development of the evidence and gap map and government stakeholder involvement. Read and reviewed final paper.

Malcolm Turner: Patient and Public Involvement representative and lead. Supported development of the protocol and provided feedback on main findings. Read and reviewed final paper.

Ruth Garside: Supported conceptualisation of the project and development of the protocol. Supported development of the evidence and gap map and government stakeholder involvement. Read and reviewed final paper.

Jo Thompson-Coon: Supported conceptualisation of the project and development of the protocol. Supported development of the evidence and gap map and led government stakeholder involvement. Read and reviewed final paper. Guarantor of work.

Conflicts of interest: None to declare.

Funding: This paper represents part of a larger piece of work commissioned by the National Institute of Health Research Policy Research Programme by the Exeter PRP Evidence Review Facility

For peer review only - http://bmjopen.bmj.com/site/about/guidelines.xhtml

(NIHR200695) following a request to the Department of Health and Social Care (DHSC) Research and Development Committee by NHS England and Improvement (NHSE-I).

Data sharing: For access to documentation supporting this review, please contact L. Shaw.

Ethics approval: This review is secondary research and did not require ethical approval.

Patient consent statement: This review is secondary research and did not require collection of patient data.

REFERENCES

1. WHO 2021 https://www.who.int/news-room/fact-sheets/detail/cardiovascular-diseases-(cvds) Accessed 15.9.21
2. NHSE 2019 https://www.england.nhs.uk/blog/the-long-term-plan-is-a-game-changer/ Accessed 15.9.21
3. NHSE 2021 https://www.england.nhs.uk/ourwork/clinical-policy/cvd/ Accessed 15.9.21
4. NHS. *The NHS Long Term Plan*: NHS; 2019.
5. National Institute for Health and Care Excellence. *Cardiovascular disease: risk assessment and reduction, including lipid modification [CG181]. Last updated: 27 September 2016*: NICE; 2014.
6. Kjeldsen SE. Hypertension and cardiovascular risk: General aspects. Pharmacol Res 2018;129:95-9. https://doi.org/10.1016/j.phrs.2017.11.003
7. Gupta A, Mackay J, Whitehouse A, Godec T, Collier T, Pocock S*, et al.* Long-term mortality after blood pressure-lowering and lipid-lowering treatment in patients with hypertension in the Anglo-Scandinavian Cardiac Outcomes Trial (ASCOT) Legacy study: 16-year follow-up results of a randomised factorial trial. *The Lancet* 2018;**392**:1127-37. https://doi.org/https://doi.org/10.1016/S0140-6736(18)31776-8
8. Scholes S, Gebert S, NHS Digital. *Health Survey for England 2018: Adults' Health*. Health and Social Care Information Centre; 2019. URL: https://files.digital.nhs.uk/E4/DAA235/HSE18-Adult-Health-rep.pdf (accessed 1 March, 2021).
9. NCD Risk Factor Collaboration. Long-term and recent trends in hypertension awareness, treatment, and control in 12 high-income countries: an analysis of 123 nationally representative surveys. *Lancet* 2019;**394**:639-51. https://doi.org/10.1016/s0140-6736(19)31145-6
10. Suissa S, Brophy J, Azoulay L, Schuster T. *Trends in Prescription of Antihypertensive Medications in UK Primary Care Practices, 1988-2018*. Clinical Practice Research Datalink; 2019. URL: https://cprd.com/protocol/trends-prescription-antihypertensive-medications-uk-primary-care-practices-1988-2018 (accessed 1 March, 2021).
11. Gupta P, Patel P, Štrauch B, Lai FY, Akbarov A, Marešová V*, et al.* Risk Factors for Nonadherence to Antihypertensive Treatment. *Hypertension* 2017;**69**:1113-20. https://doi.org/10.1161/hypertensionaha.116.08729
12. Reference full report. Withheld for peer review.
13. Centre for Reviews and Dissemination. *Systematic Reviews: CRD's guidance for undertaking reviews in health care*: University of York; 2008.

For peer review only - http://bmjopen.bmj.com/site/about/guidelines.xhtml

14. White H, Albers B, Gaarder M, Kornør H, Littell J, Marshall Z*, et al.* Guidance for producing a Campbell evidence and gap map. *Campbell Systematic Reviews* 2020;**16**:e1125. https://doi.org/10.1002/cl2.1125
15. Review protocol. Reference withheld for peer review
16. Moher D, Liberati A, Tetzlaff J, Altman DG. Preferred reporting items for systematic reviews and meta-analyses: the PRISMA statement. *BMJ* 2009;**339**:b2535. https://doi.org/10.1136/bmj.b2535
17. Collaboration for Environmental Evidence. *The Collaboration for Environmental Evidence Synthesis Appraisal Tool (CEESAT). Version 2.1. (Last updated 27 October 2020).* CEE; 2018. URL: https://environmentalevidence.shinyapps.io/CEEDER/ (accessed 13 Jan, 2021).
18. Shea BJ, Reeves BC, Wells G, Thuku M, Hamel C, Moran J*, et al.* AMSTAR 2: a critical appraisal tool for systematic reviews that include randomised or non-randomised studies of healthcare interventions, or both. *BMJ* 2017;**358**:j4008. https://doi.org/10.1136/bmj.j4008
19. Tong A, Flemming K, McInnes E, Oliver S, Craig J. Enhancing transparency in reporting the synthesis of qualitative research: ENTREQ. *BMC Med Res Methodol* 2012;**12**:181. https://doi.org/10.1186/1471-2288-12-181
20. Digital Solution Foundry, EPPI-Centre. *EPPI Mapper, Version 1.2.5*: EPPI-Centre, UCL Social Research Institute, University College London; 2020.
21. National Institute for Health and Care Excellence. *Hypertension in adults: diagnosis and management NICE guideline [NG136]* London: NICE; 2019.
22. National Institute for Health and Care Excellence. *Medicines optimisation: the safe and effective use of medicines to enable the best possible outcomes* London: NICE; 2015.
23. National Institute for Health and Care Excellence. *Shared decision-making about medicines*. NICE Pathways; 2021. URL: https://pathways.nice.org.uk/pathways/medicines-optimisation#path=view%3A/pathways/medicines-optimisation/shared-decision-making-about-medicines.xml&content=view-index (accessed 3 March, 2021).
24. Pound P, Britten N, Morgan M, Yardley L, Pope C, Daker-White G*, et al.* Resisting medicines: a synthesis of qualitative studies of medicine taking. *Soc Sci Med* 2005;**61**:133-55. https://doi.org/10.1016/j.socscimed.2004.11.063
25. Deichmann RE, Morledge MD, Ulep R, Shaffer JP, Davies P, van Driel ML. A Metaanalysis of Interventions to Improve Adherence to Lipid-Lowering Medication. *Ochsner J* 2016;**16**:230-7.
26. Hukins D, Macleod U, Boland JW. Identifying potentially inappropriate prescribing in older people with dementia: a systematic review. *Eur J Clin Pharmacol* 2019;**75**:467-81. https://doi.org/10.1007/s00228-018-02612-x
27. Kinnear FJ, Wainwright E, Perry R, Lithander FE, Bayly G, Huntley A*, et al.* Enablers and barriers to treatment adherence in heterozygous familial hypercholesterolaemia: a qualitative evidence synthesis. *BMJ Open* 2019;**9**:e030290. https://doi.org/10.1136/bmjopen-2019-030290
28. Legare F, Ratte S, Stacey D, Kryworuchko J, Gravel K, Graham ID*, et al.* Interventions for improving the adoption of shared decision making by healthcare professionals. *Cochrane Database Syst Rev* 2010; 10.1002/14651858.CD006732.pub2:CD006732. https://doi.org/10.1002/14651858.CD006732.pub2
29. Nguyen T, Nguyen HQ, Widyakusuma NN, Nguyen TH, Pham TT, Taxis K. Enhancing prescribing of guideline-recommended medications for ischaemic heart diseases: a systematic review and meta-analysis of interventions targeted at healthcare professionals. *BMJ Open* 2018;**8**:e018271. https://doi.org/10.1136/bmjopen-2017-018271

For peer review only - http://bmjopen.bmj.com/site/about/guidelines.xhtml

30. Rash JA, Campbell DJ, Tonelli M, Campbell TS. A systematic review of interventions to improve adherence to statin medication: What do we know about what works? *Prev Med* 2016;**90**:155-69. https://doi.org/10.1016/j.ypmed.2016.07.006
31. Rashid MA, Edwards D, Walter FM, Mant J. Medication taking in coronary artery disease: a systematic review and qualitative synthesis. *Ann Fam Med* 2014;**12**:224-32. https://doi.org/10.1370/afm.1620
32. van Driel ML, Morledge MD, Ulep R, Shaffer JP, Davies P, Deichmann R. Interventions to improve adherence to lipid-lowering medication. *Cochrane Database of Systematic Reviews* 2016; 10.1002/14651858.CD004371.pub4. https://doi.org/10.1002/14651858.CD004371.pub4
33. Bochkareva EV, Butina EK, Kim IV, Kontsevaya AV, Drapkina OM, Leon D*, et al.* Adherence to antihypertensive medication in Russia: a scoping review of studies on levels, determinants and intervention strategies published between 2000 and 2017. *Arch Public Health* 2019;**77**:43. https://doi.org/10.1186/s13690-019-0366-9
34. Cheema E, Sutcliffe P, Singer DR. The impact of interventions by pharmacists in community pharmacies on control of hypertension: a systematic review and meta-analysis of randomized controlled trials. *Br J Clin Pharmacol* 2014;**78**:1238-47. https://doi.org/10.1111/bcp.12452
35. Chhina HK, Bhole VM, Goldsmith C, Hall W, Kaczorowski J, Lacaille D. Effectiveness of academic detailing to optimize medication prescribing behaviour of family physicians. *J Pharm Pharm Sci* 2013;**16**:511-29. https://doi.org/10.18433/j3kk6c
36. Conn VS, Ruppar TM, Chase JA, Enriquez M, Cooper PS. Interventions to Improve Medication Adherence in Hypertensive Patients: Systematic Review and Meta-analysis. *Curr Hypertens Rep* 2015;**17**:94. https://doi.org/10.1007/s11906-015-0606-5
37. De Simoni A, Hardeman W, Mant J, Farmer AJ, Kinmonth AL. Trials to improve blood pressure through adherence to antihypertensives in stroke/TIA: systematic review and meta-analysis. *J Am Heart Assoc* 2013;**2**:e000251. https://doi.org/10.1161/JAHA.113.000251
38. Demonceau J, Ruppar T, Kristanto P, Hughes DA, Fargher E, Kardas P*, et al.* Identification and assessment of adherence-enhancing interventions in studies assessing medication adherence through electronically compiled drug dosing histories: a systematic literature review and meta-analysis. *Drugs* 2013;**73**:545-62. https://doi.org/10.1007/s40265-013-0041-3
39. Dhar L, Dantas J, Ali M. A Systematic Review of Factors Influencing Medication Adherence to Hypertension Treatment in Developing Countries. *Open Journal of Epidemiology* 2017;**07**:211-50. https://doi.org/10.4236/ojepi.2017.73018
40. Du LP, Cheng ZW, Zhang YX, Li Y, Mei D. The impact of fixed-dose combination versus free-equivalent combination therapies on adherence for hypertension: a meta-analysis. *J Clin Hypertens (Greenwich)* 2018;**20**:902-7. https://doi.org/10.1111/jch.13272
41. Fletcher BR, Hartmann-Boyce J, Hinton L, McManus RJ. The Effect of Self-Monitoring of Blood Pressure on Medication Adherence and Lifestyle Factors: A Systematic Review and Meta-Analysis. *Am J Hypertens* 2015;**28**:1209-21. https://doi.org/10.1093/ajh/hpv008
42. Gupta AK, Arshad S, Poulter NR. Compliance, safety, and effectiveness of fixed-dose combinations of antihypertensive agents: a meta-analysis. *Hypertension* 2010;**55**:399-407. https://doi.org/10.1161/HYPERTENSIONAHA.109.139816
43. Guzman-Tordecilla DN, Bernal Garcia A, Rodriguez I. Interventions to increase the pharmacological adherence on arterial hypertension in Latin America: a systematic review. *Int J Public Health* 2020;**65**:55-64. https://doi.org/10.1007/s00038-019-01317-x

For peer review only - http://bmjopen.bmj.com/site/about/guidelines.xhtml

44. Hart LA, Phelan EA, Yi JY, Marcum ZA, Gray SL. Use of Fall Risk-Increasing Drugs Around a Fall-Related Injury in Older Adults: A Systematic Review. *J Am Geriatr Soc* 2020;**68**:1334-43. https://doi.org/10.1111/jgs.16369
45. Kawalec P, Holko P, Gawin M, Pilc A. Effectiveness of fixed-dose combination therapy in hypertension: systematic review and meta-analysis. *Arch Med Sci* 2018;**14**:1125-36. https://doi.org/10.5114/aoms.2018.77561
46. Khatib R, Schwalm JD, Yusuf S, Haynes RB, McKee M, Khan M*, et al.* Patient and healthcare provider barriers to hypertension awareness, treatment and follow up: a systematic review and meta-analysis of qualitative and quantitative studies. *PLoS One* 2014;**9**:e84238. https://doi.org/10.1371/journal.pone.0084238
47. Kronish IM, Woodward M, Sergie Z, Ogedegbe G, Falzon L, Mann DM. Meta-analysis: impact of drug class on adherence to antihypertensives. *Circulation* 2011;**123**:1611-21. https://doi.org/10.1161/CIRCULATIONAHA.110.983874
48. Loganathan M, Singh S, Franklin BD, Bottle A, Majeed A. Interventions to optimise prescribing in care homes: systematic review. *Age Ageing* 2011;**40**:150-62. https://doi.org/10.1093/ageing/afq161
49. Maimaris W, Paty J, Perel P, Legido-Quigley H, Balabanova D, Nieuwlaat R*, et al.* The influence of health systems on hypertension awareness, treatment, and control: a systematic literature review. *PLoS Med* 2013;**10**:e1001490. https://doi.org/10.1371/journal.pmed.1001490
50. Mallat SG, Tanios BY, Itani HS, Lotfi T, Akl EA. Free versus Fixed Combination Antihypertensive Therapy for Essential Arterial Hypertension: A Systematic Review and Meta-Analysis. *PLoS One* 2016;**11**:e0161285. https://doi.org/10.1371/journal.pone.0161285
51. Marshall IJ, Wolfe CD, McKevitt C. Lay perspectives on hypertension and drug adherence: systematic review of qualitative research. *BMJ* 2012;**345**:e3953. https://doi.org/10.1136/bmj.e3953
52. Milosavljevic A, Aspden T, Harrison J. Community pharmacist-led interventions and their impact on patients' medication adherence and other health outcomes: a systematic review. *Int J Pharm Pract* 2018;**26**:387-97. https://doi.org/10.1111/ijpp.12462
53. Morrissey EC, Durand H, Nieuwlaat R, Navarro T, Haynes RB, Walsh JC*, et al.* Effectiveness and content analysis of interventions to enhance medication adherence and blood pressure control in hypertension: A systematic review and meta-analysis. *Psychol Health* 2017;**32**:1195-232. https://doi.org/10.1080/08870446.2016.1273356
54. Oori MJ, Mohammadi F, Norouzi K, Fallahi-Khoshknab M, Ebadi A. Conceptual Model of Medication Adherence in Older Adults with High Blood Pressure-An Integrative Review of the Literature. *Curr Hypertens Rev* 2019;**15**:85-92. https://doi.org/10.2174/1573402114666181022152313
55. Rashid MA, Llanwarne N, Heyns N, Walter F, Mant J. What are the implications for practice that arise from studies of medication taking? A systematic review of qualitative research. *PLoS One* 2018;**13**:e0195076. https://doi.org/10.1371/journal.pone.0195076
56. Reeve E, To J, Hendrix I, Shakib S, Roberts MS, Wiese MD. Patient barriers to and enablers of deprescribing: a systematic review. *Drugs Aging* 2013;**30**:793-807. https://doi.org/10.1007/s40266-013-0106-8
57. Reeves L, Robinson K, McClelland T, Adedoyin CA, Broeseker A, Adunlin G. Pharmacist Interventions in the Management of Blood Pressure Control and Adherence to Antihypertensive Medications: A Systematic Review of Randomized Controlled Trials. *J Pharm Pract* 2020; 10.1177/0897190020903573:897190020903573. https://doi.org/10.1177/0897190020903573

For peer review only - http://bmjopen.bmj.com/site/about/guidelines.xhtml

58. Sanders GD, Coeytaux R, Dolor RJ, Hasselblad V, Patel UD, Powers B*, et al.* AHRQ Comparative Effectiveness Reviews. In: *Angiotensin-Converting Enzyme Inhibitors (ACEIs), Angiotensin II Receptor Antagonists (ARBs), and Direct Renin Inhibitors for Treating Essential Hypertension: An Update*Rockville (MD): Agency for Healthcare Research and Quality (US); 2011.
59. Shanbhag D, Graham ID, Harlos K, Haynes RB, Gabizon I, Connolly SJ*, et al.* Effectiveness of implementation interventions in improving physician adherence to guideline recommendations in heart failure: a systematic review. *BMJ Open* 2018;**8**:e017765. https://doi.org/10.1136/bmjopen-2017-017765
60. Stacey D, Legare F, Lewis K, Barry MJ, Bennett CL, Eden KB*, et al.* Decision aids for people facing health treatment or screening decisions. *Cochrane Database Syst Rev* 2017;**4**:CD001431. https://doi.org/10.1002/14651858.CD001431.pub5
61. Tsioufis K, Kreutz R, Sykara G, van Vugt J, Hassan T. Impact of single-pill combination therapy on adherence, blood pressure control, and clinical outcomes: a rapid evidence assessment of recent literature. *Journal of Hypertension* 2020;**38**:1016-28. https://doi.org/10.1097/Hjh.0000000000002381
62. Uhlig K, Patel K, Ip S, Kitsios GD, Balk EM. Self-measured blood pressure monitoring in the management of hypertension: a systematic review and meta-analysis. *Ann Intern Med* 2013;**159**:185-94. https://doi.org/10.7326/0003-4819-159-3-201308060-00008
63. Xiong S, Berkhouse H, Schooler M, Pu W, Sun A, Gong E*, et al.* Effectiveness of mHealth Interventions in Improving Medication Adherence Among People with Hypertension: a Systematic Review. *Curr Hypertens Rep* 2018;**20**:86. https://doi.org/10.1007/s11906-018-0886-7
64. Xu R, Xie X, Li S, Chen X, Wang S, Hu C*, et al.* Interventions to improve medication adherence among Chinese patients with hypertension: a systematic review and meta-analysis of randomized controlled trails. *Int J Pharm Pract* 2018;**26**:291-301. https://doi.org/10.1111/ijpp.12452
65. Yuan C, Ding Y, Zhou K, Huang Y, Xi X. Clinical outcomes of community pharmacy services: A systematic review and meta-analysis. *Health Soc Care Community* 2019;**27**:e567-e87. https://doi.org/10.1111/hsc.12794
66. Adler AJ, Martin N, Mariani J, Tajer CD, Owolabi OO, Free C*, et al.* Mobile phone text messaging to improve medication adherence in secondary prevention of cardiovascular disease. *Cochrane Database Syst Rev* 2017;**4**:CD011851. https://doi.org/10.1002/14651858.CD011851.pub2
67. Al AlShaikh S, Quinn T, Dunn W, Walters M, Dawson J. Multimodal Interventions to Enhance Adherence to Secondary Preventive Medication after Stroke: A Systematic Review and Meta-Analyses. Cardiovasc Ther 2016;34:85-93. https://doi.org/10.1111/1755-5922.12176
68. Baumgartner A, Drame K, Geutjens S, Airaksinen M. Does the Polypill Improve Patient Adherence Compared to Its Individual Formulations? A Systematic Review. *Pharmaceutics* 2020;**12**:190. https://doi.org/10.3390/pharmaceutics12020190
69. Checchi KD, Huybrechts KF, Avorn J, Kesselheim AS. Electronic medication packaging devices and medication adherence: a systematic review. *JAMA* 2014;**312**:1237-47. https://doi.org/10.1001/jama.2014.10059
70. Conn VS, Ruppar TM, Chan KC, Dunbar-Jacob J, Pepper GA, De Geest S. Packaging interventions to increase medication adherence: systematic review and meta-analysis. *Curr Med Res Opin* 2015;**31**:145-60. https://doi.org/10.1185/03007995.2014.978939

For peer review only - http://bmjopen.bmj.com/site/about/guidelines.xhtml

71. Conn VS, Ruppar TM, Enriquez M, Cooper PS, Chan KC. Healthcare provider targeted interventions to improve medication adherence: systematic review and meta-analysis. *Int J Clin Pract* 2015;**69**:889-99. https://doi.org/10.1111/ijcp.12632
72. Conn VS, Ruppar TM, Enriquez M, Cooper P. Medication adherence interventions that target subjects with adherence problems: Systematic review and meta-analysis. *Res Social Adm Pharm* 2016;**12**:218-46. https://doi.org/10.1016/j.sapharm.2015.06.001
73. de Cates AN, Farr MR, Wright N, Jarvis MC, Rees K, Ebrahim S*, et al.* Fixed-dose combination therapy for the prevention of cardiovascular disease. *Cochrane Database Syst Rev* 2014; 10.1002/14651858.CD009868.pub2:CD009868. https://doi.org/10.1002/14651858.CD009868.pub2
74. Dills H, Shah K, Messinger-Rapport B, Bradford K, Syed Q. Deprescribing Medications for Chronic Diseases Management in Primary Care Settings: A Systematic Review of Randomized Controlled Trials. *J Am Med Dir Assoc* 2018;**19**:923-35 e2.
75. Ingersgaard MV, Helms Andersen T, Norgaard O, Grabowski D, Olesen K. Reasons for Nonadherence to Statins - A Systematic Review of Reviews. *Patient Prefer Adherence* 2020;**14**:675-91. https://doi.org/10.2147/PPA.S245365
76. Jeffery RA, To MJ, Hayduk-Costa G, Cameron A, Taylor C, Van Zoost C*, et al.* Interventions to improve adherence to cardiovascular disease guidelines: a systematic review. *BMC Fam Pract* 2015;**16**:147. https://doi.org/10.1186/s12875-015-0341-7
77. Kang JE, Han NY, Oh JM, Jin HK, Kim HA, Son IJ*, et al.* Pharmacist-involved care for patients with heart failure and acute coronary syndrome: a systematic review with qualitative and quantitative meta-analysis. *J Clin Pharm Ther* 2016;**41**:145-57. https://doi.org/10.1111/jcpt.12367
78. Kassavou A, Sutton S. Automated telecommunication interventions to promote adherence to cardio-metabolic medications: meta-analysis of effectiveness and meta-regression of behaviour change techniques. *Health Psychol Rev* 2018;**12**:25-42. https://doi.org/10.1080/17437199.2017.1365617
79. King S, Miani C, Exley J, Larkin J, Kirtley A, Payne RA. Impact of issuing longer- versus shorter-duration prescriptions: a systematic review. *Br J Gen Pract* 2018;**68**:e286-e92. https://doi.org/10.3399/bjgp18X695501
80. Krack G. How to make value-based health insurance designs more effective? A systematic review and meta-analysis. *Eur J Health Econ* 2019;**20**:841-56. https://doi.org/10.1007/s10198-019-01046-1
81. Laba TL, Bleasel J, Brien JA, Cass A, Howard K, Peiris D*, et al.* Strategies to improve adherence to medications for cardiovascular diseases in socioeconomically disadvantaged populations: a systematic review. *Int J Cardiol* 2013;**167**:2430-40. https://doi.org/10.1016/j.ijcard.2013.01.049
82. Lawrence M, Pringle J, Kerr S, Booth J, Govan L, Roberts NJ. Multimodal secondary prevention behavioral interventions for TIA and stroke: a systematic review and meta-analysis. *PLoS One* 2015;**10**:e0120902. https://doi.org/10.1371/journal.pone.0120902
83. Leslie RS. *Adherence to medications to treat diabetes, hypertension, and hypercholesterolemia among Medicare Part D beneficiaries* [PhD]. San Diego: University of California; 2016.
84. Mamudu HM, Paul TK, Veeranki SP, Budoff M. The effects of coronary artery calcium screening on behavioral modification, risk perception, and medication adherence among asymptomatic adults: a systematic review. *Atherosclerosis* 2014;**236**:338-50. https://doi.org/10.1016/j.atherosclerosis.2014.07.022

For peer review only - http://bmjopen.bmj.com/site/about/guidelines.xhtml

85. Mann BS, Barnieh L, Tang K, Campbell DJ, Clement F, Hemmelgarn B, *et al.* Association between drug insurance cost sharing strategies and outcomes in patients with chronic diseases: a systematic review. *PLoS One* 2014;**9**:e89168. https://doi.org/10.1371/journal.pone.0089168
86. Nieuwlaat R, Wilczynski N, Navarro T, Hobson N, Jeffery R, Keepanasseril A, *et al.* Interventions for enhancing medication adherence. *Cochrane Database Syst Rev* 2014;**2014**:CD000011. https://doi.org/10.1002/14651858.CD000011.pub4
87. Page AT, Clifford RM, Potter K, Schwartz D, Etherton-Beer CD. The feasibility and effect of deprescribing in older adults on mortality and health: a systematic review and meta-analysis. *Br J Clin Pharmacol* 2016;**82**:583-623. https://doi.org/10.1111/bcp.12975
88. Palmer MJ, Barnard S, Perel P, Free C. Mobile phone-based interventions for improving adherence to medication prescribed for the primary prevention of cardiovascular disease in adults. *Cochrane Database Syst Rev* 2018;**6**:CD012675. https://doi.org/10.1002/14651858.CD012675.pub2
89. Pantoja T, Grimshaw JM, Colomer N, Castanon C, Leniz Martelli J. Manually-generated reminders delivered on paper: effects on professional practice and patient outcomes. *Cochrane Database Syst Rev* 2019;**12**:CD001174. https://doi.org/10.1002/14651858.CD001174.pub4
90. Park YJ, Martin EG. Medicare Part D's Effects on Drug Utilization and Out-of-Pocket Costs: A Systematic Review. *Health Serv Res* 2017;**52**:1685-728. https://doi.org/10.1111/1475-6773.12534
91. Polinski JM, Donohue JM, Kilabuk E, Shrank WH. Medicare Part D's effect on the under- and overuse of medications: a systematic review. *J Am Geriatr Soc* 2011;**59**:1922-33. https://doi.org/10.1111/j.1532-5415.2011.03537.x
92. Posadzki P, Mastellos N, Ryan R, Gunn LH, Felix LM, Pappas Y, *et al.* Automated telephone communication systems for preventive healthcare and management of long-term conditions. *Cochrane Database Syst Rev* 2016;**12**:CD009921. https://doi.org/10.1002/14651858.CD009921.pub2
93. Bond, Z.; Scanlon, T.; Judah, G. Systematic Review of RCTs Assessing the Effectiveness of mHealth Interventions to Improve Statin Medication Adherence: Using the Behaviour-Change Technique Taxonomy to Identify the Techniques That Improve Adherence. Healthcare 2021, 9, 1282. https://doi.org/ 10.3390/healthcare9101282
94. Coronado-Vázquez V, Canet-Fajas C, Delgado-Marroquín MT, Magallón-Botaya R, Romero-Martín M, Gómez-Salgado J. Interventions to facilitate shared decision-making using decision aids with patients in Primary Health Care: A systematic review. Medicine (Baltimore). 2020 Aug 7;99(32):e21389. doi: 10.1097/MD.0000000000021389
95. Guzman-Tordecilla DN, Bernal García A, Rodríguez I. Interventions to increase the pharmacological adherence on arterial hypertension in Latin America: a systematic review. Int J Public Health. 2020 Jan;65(1):55-64. doi: 10.1007/s00038-019-01317-x
96. Hong J, Tiu YC, Leung PYB, Wong MF, Ng WY, Cheung D, Mok HY, Lam WY, Li KY, Wong CK. Interventions that improve adherence to antihypertensive medications in coronary heart disease patients: a systematic review. Postgrad Med J. 2022 Mar;98(1157):219-227. doi: 10.1136/postgradmedj-2020-139116
97. Nili M, Mohamed R, Kelly KM. A systematic review of interventions using health behavioral theories to improve medication adherence among patients with hypertension. Transl Behav Med. 2020 Oct 12;10(5):1177-1186. doi: 10.1093/tbm/ibaa020. PMID: 33044533
98. Palmer MJ, Machiyama K, Woodd S, Gubijev A, Barnard S, Russell S, Perel P, Free C. Mobile phone-based interventions for improving adherence to medication prescribed for the

For peer review only - http://bmjopen.bmj.com/site/about/guidelines.xhtml

primary prevention of cardiovascular disease in adults. Cochrane Database of Systematic Reviews 2021, Issue 3. Art. No.: CD012675. DOI: 10.1002/14651858.CD012675.pub3

99. Parati G, Kjeldsen S, Coca A, Cushman WC, Wang J. Adherence to Single-Pill Versus Free-Equivalent Combination Therapy in Hypertension: A Systematic Review and Meta-Analysis. Hypertension. 2021 Feb;77(2):692-705. doi: 10.1161/HYPERTENSIONAHA.120.15781

100. Qadi O, Lufumpa N, Adderley N, Bem D, Marshall T, Kokab F. Patients' and health professionals' attitudes and perceptions towards the initiation of preventive drugs for primary prevention of cardiovascular disease: a systematic review of qualitative studies. BJGP Open. 2020 Dec 15;4(5):bjgpopen20X101087. doi: 10.3399/bjgpopen20X101087

101. Reston JT, Buelt A, Donahue MP, Neubauer B, Vagichev E, McShea K. Interventions to Improve Statin Tolerance and Adherence in Patients at Risk for Cardiovascular Disease : A Systematic Review for the 2020 U.S. Department of Veterans Affairs and U.S. Department of Defense Guidelines for Management of Dyslipidemia. Ann Intern Med. 2020 Nov 17;173(10):806-812. doi: 10.7326/M20-4680

102. Van Truong P, Wulan Apriliyasari R, Lin MY, Chiu HY, Tsai PS. Effects of self-management programs on blood pressure, self-efficacy, medication adherence and body mass index in older adults with hypertension: Meta-analysis of randomized controlled trials. Int J Nurs Pract. 2021 Apr;27(2):e12920. doi: 10.1111/ijn.12920

103. Vervloet M, Korevaar JC, Leemrijse CJ, Paget J, Zullig LL, van Dijk L. Interventions to Improve Adherence to Cardiovascular Medication: What About Gender Differences? A Systematic Literature Review. Patient Prefer Adherence. 2020 Oct 29;14:2055-2070. doi: 10.2147/PPA.S260562

104. Xie CX, Chen Q, Hincapié CA, Hofstetter L, Maher CG, Machado GC. Effectiveness of clinical dashboards as audit and feedback or clinical decision support tools on medication use and test ordering: a systematic review of randomized controlled trials. J Am Med Inform Assoc. 2022 Sep 12;29(10):1773-1785. doi: 10.1093/jamia/ocac094

For peer review only

For peer review only - http://bmjopen.bmj.com/site/about/guidelines.xhtml

FIGURES

Figure 1: Evidence and gap map

Figure 2: PRISMA diagram

Figure 3: Focus/aims of systematic review evidence included in the map

For peer review only

For peer review only - http://bmjopen.bmj.com/site/about/guidelines.xhtml

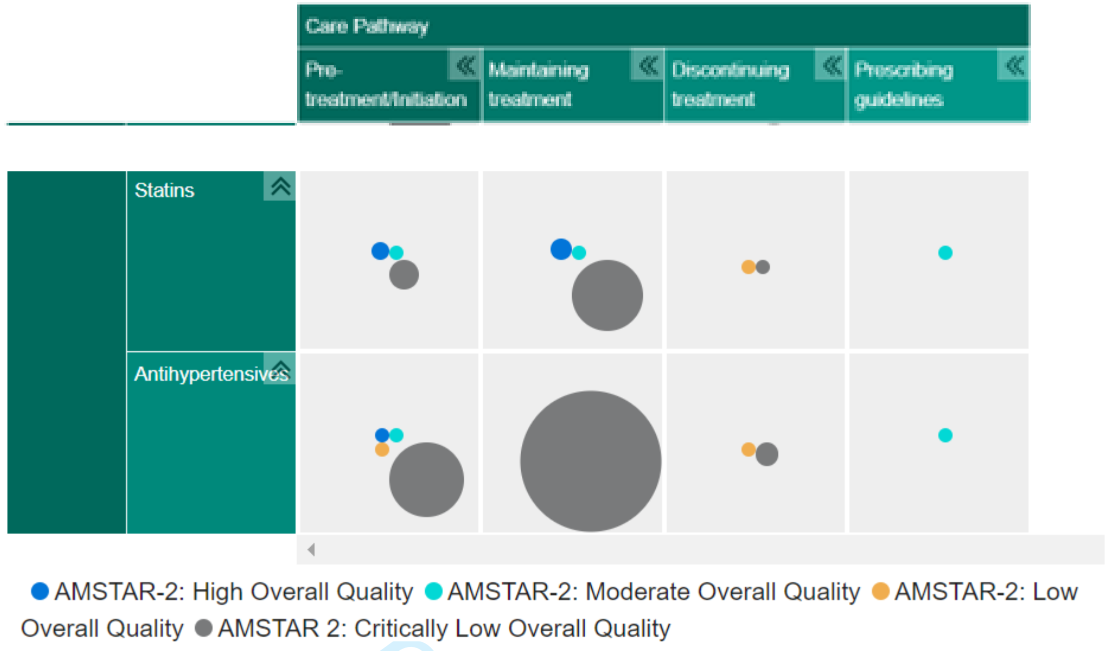

Figure 1: Evidence and gap map

peer review only

PRISMA 2020 flow diagram for new systematic reviews which included searches of databases, registers and other sources

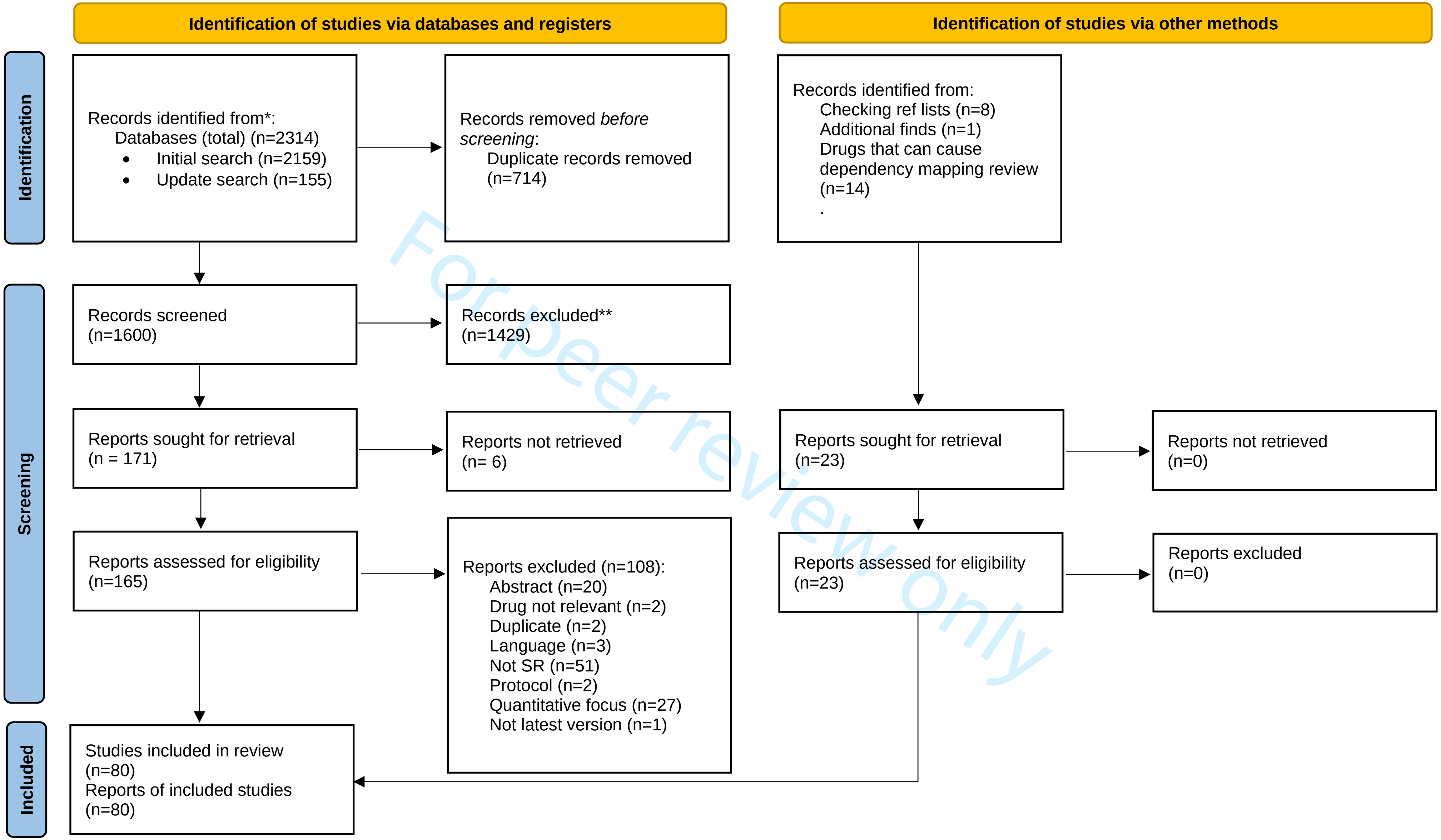

*Consider, if feasible to do so, reporting the number of records identified from each database or register searched (rather than the total number across all databases/registers).
**If automation tools were used, indicate how many records were excluded by a human and how many were excluded by automation tools.

From: Page MJ, McKenzie JE, Bossuyt PM, Boutron I, Hoffmann TC, Mulrow CD, et al. The PRISMA 2020 statement: an updated guideline for reporting systematic reviews. BMJ 2021;372:n71. doi: 10.1136/bmj.n71. For more information, visit: http://www.prisma-statement.org/

For peer review only - http://bmjopen.bmj.com/site/about/guidelines.xhtml

PRISMA 2020 flow diagram for new systematic reviews which included searches of databases, registers and other sources

For peer review only

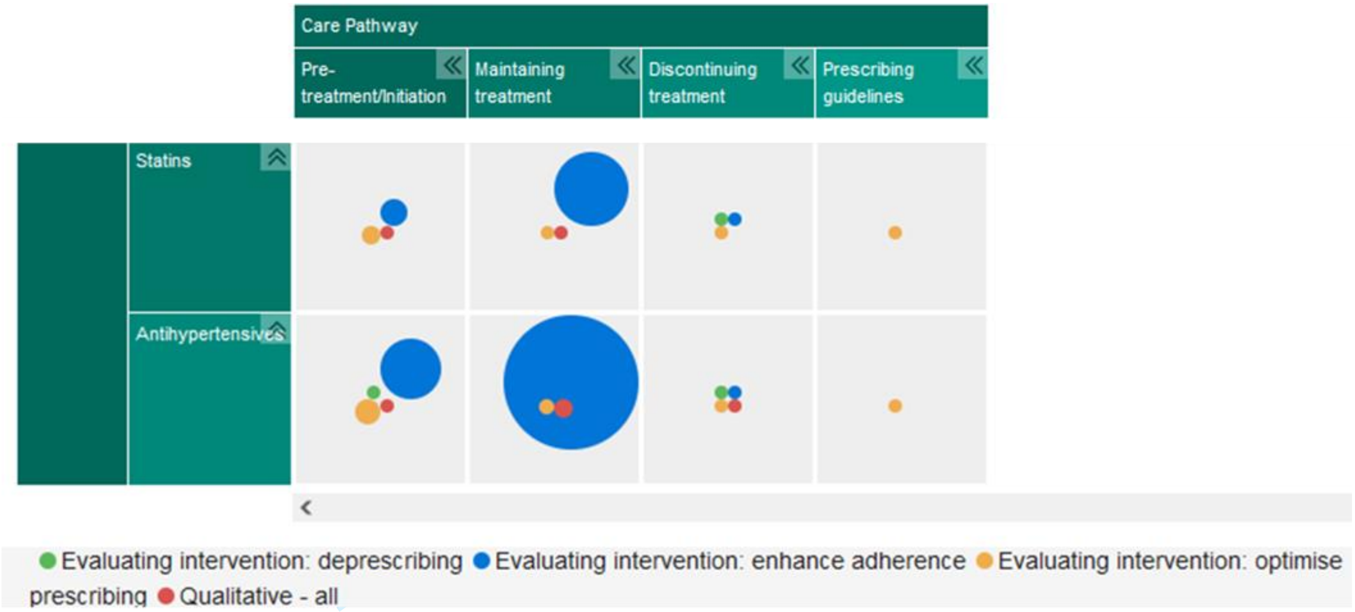

Figure 3: Focus/aims of systematic review evidence included in the map

For peer review only

APPENDIX A: SEARCH STRATEGY

Bibliographic database searches

Drugs that cause dependency

Database: Cochrane Database of Systematic Reviews
Host: Cochrane Library
Issue: Issue 8 of 12, August 2020
Date Searched: 11/8/2020
Searcher: SB
Hits: 111
Strategy:

#1 (((appropriate* or discontinu* or enhance* or inappropriate* or incorrect* or "in correct*" or optim* or safe or suboptim* or "sub optim*" or tapering or withdrawal) NEAR/4 (drug* or medicine* or medication* or prescri*))):ti,ab,kw

#2 MeSH descriptor: [Inappropriate Prescribing] this term only

#3 MeSH descriptor: [Drug Prescriptions] explode all trees

#4 (((drug* or guideline* or guidance or medicine* or medication* or patient* or prescri*) NEAR/4 (adhere* or compliance or concordance))):ti,ab,kw

#5 MeSH descriptor: [Patient Compliance] explode all trees

#6 ((shared or sharing or informed) near/2 (decision* or choice*)):ti,ab,kw

#7 ((decision near/2 (aid* or support*))):ti,ab,kw

#8 MeSH descriptor: [Decision Making] this term only

#9 MeSH descriptor: [Decision Support Techniques] this term only

#10 (((consumer* or patient*) near/3 (involv* or participat*))):ti,ab,kw

#11 MeSH descriptor: [Patient Participation] this term only

#12 ((patient NEXT cent* near/2 (approach* or care or decision* or intervention* or treatment*))):ti,ab,kw

#13 MeSH descriptor: [Patient-Centered Care] this term only

#14 ((behavi* near/2 chang*)):ti,ab,kw

#15 ((restriction near/2 (policy or policies))):ti,ab,kw

#16 {Wilson, #1-#15}

#17 (benzodiazepine*):ti,ab,kw

#18 ((alprazolam or flunitrazepam or chlordiazepoxide or clobazam or clonazepam or diazepam or lorazepam or midazolam or nitrazepam or oxazepam or prazepam or temazepam)):ti,ab,kw

#19 MeSH descriptor: [Benzodiazepines] explode all trees

For peer review only - http://bmjopen.bmj.com/site/about/guidelines.xhtml

#20 ((antidepres* or "anti depres*")):ti,ab,kw

#21 ((serotonin or norepinephrine or noradrenaline or neurotransmitter* or dopamin* or SSRI* or SNRI* or NARI* or SARI* or NDRI* or tricyclic* or tetracyclic*)):ti,ab,kw

#22 MeSH descriptor: [Antidepressive Agents] explode all trees

#23 ((opioid* or opiate*)):ti,ab,kw

#24 ((morphine or hydromorphone or levorphanol or meperidine or methadone or propoxyphene of codeine or pentazocine or hydrocodone or oxycodone or fentanyl or tramadol)):ti,ab,kw

#25 MeSH descriptor: [Analgesics, Opioid] explode all trees

#26 ("z drug*"):ti,ab,kw

#27 ((zopiclone or zolpidem or zaleplon or eszopiclone)):ti,ab,kw

#28 MeSH descriptor: [Hypnotics and Sedatives] explode all trees

#29 ((gabapentin* or mirogabalin or phenibut or pregabalin)):ti,ab,kw

#30 MeSH descriptor: [Gabapentin] this term only

#31 {Ahmed, #17-#30}

#32 #16 AND #31 in Cochrane Reviews [Drugs that cause dependency search results]

#33 (statin*):ti,ab,kw

#34 (("reductase inhibitors" or "HMGCR inhibitors")):ti,ab,kw

#35 MeSH descriptor: [Hydroxymethylglutaryl-CoA Reductase Inhibitors] explode all trees

#36 ((atorvastatin or Lipitor or Torvast)):ti,ab,kw

#37 ((fluvastatin or Lescol)):ti,ab,kw

#38 ((lovastatin or Mevacor or Altocor or Altoprev)):ti,ab,kw

#39 ((pitavastatin or Livalo or Pitava)):ti,ab,kw

#40 ((pravastatin or Pravachol or Selektine or Lipostat)):ti,ab,kw

#41 ((rosuvastatin or Crestor)):ti,ab,kw

#42 ((simvastatin or Zocor or Lipex)):ti,ab,kw

#43 (antihypertensive*):ti,ab,kw

#44 MeSH descriptor: [Antihypertensive Agents] explode all trees

#45 ((ACE NEXT inhibitor* or antagonist or "angiotensin II receptor" or beta NEXT blocker* or calcium NEXT channel NEXT blocker* or thiazide NEXT diuretic*)):ti,ab,kw

#46 ((acebutolol or adrenomedullin or alprenolol or amlodipine or atenolol or bendroflumethiazide or bepridil or betaxolol or bethanidine or bimatoprost or bisoprolol or bosentan or "bretylium tosylate" or brimonidine tartrate or bupranolol or captopril or carteolol or

For peer review only - http://bmjopen.bmj.com/site/about/guidelines.xhtml

carvedilol or celiprolol or chlorisondamine or chlorothiazide or chlorthalidone or cilazapril or clonidine or cromakalim or cyclopenthiazide or debrisoquin or diazoxide or dihydralazine or dihydroalprenolol or diltiazem or doxazosin or enalapril or enalaprilat or eplerenone or epoprostenol or felodipine or fenoldopam or fosinopril or guanabenz or guanethidine or guanfacine or hexamethonium or "hexamethonium compound*" or hydralazine or hydrochlorothiazide or hydroflumethiazide or indapamide or indoramin or irbesartan or isradipine or kallidin or ketanserin or labetalol or latanoprost or lisinopril or losartan or mecamylamine or methyldopa or metipranolol or metolazone or metoprolol or mibefradil or minoxidil or muzolimine or nadolol or nebivolol or nicardipine or nicorandil or nimodipine or nisoldipine or nitrendipine or nitroprusside or olmesartan medoxomil or oxprenolol or pargyline or pempidine or penbutolol or "pentolinium tartrate" or perindopril or phenoxybenzamine or phentolamine or pinacidil or pindolol or piperoxan or polythiazide or prazosin or propranolol or protoveratrines or quinapril or ramipril or reserpine or rilmenidine or telmisartan or teprotide or terlipressin or ticrynafen or timolol or todralazine or tolazoline or torsemide or travoprost or trichlormethiazide or trimethaphan or valsartan or "veratrum alkaloid*" or vincamine or xipamide)):ti,ab,kw

#47 (103-#46)

#48 (cardiovascular):ti,ab,kw

#49 MeSH descriptor: [Cardiovascular Diseases] explode all trees

#50 (((cardiac or coronary or heart) near/3 (arrest* or attack* or disease* or failure*))):ti,ab,kw

#51 ((heart or myocard* or transient) near/3 (infarc* or ischaemi* or ischemi*)):ti,ab,kw

#52 (angina*):ti,ab,kw

#53 MeSH descriptor: [Angina Pectoris] this term only

#54 (stroke*):ti,ab,kw

#55 MeSH descriptor: [Stroke] explode all trees

#56 {Ford, #48-#55}

#57 #16 and #47 and #56 in Cochrane Reviews [Statins search results]

Notes: Date limited January 2010 – to date of search.

Database: CINAHL
Host: EBSCO
Issue: n/a
Date Searched: 11/8/2020
Searcher: SB
Hits: 372
Strategy

1. TI ((appropriate* or discontinu* or enhance* or inappropriate* or incorrect* or "in correct*" or optim* or safe or suboptim* or "sub optim*" or tapering or withdrawal) N3 (drug* or medicine* or medication* or prescri*)) OR AB ((appropriate* or discontinu* or enhance* or inappropriate* or incorrect* or "in correct*" or optim* or safe or suboptim* or

For peer review only

For peer review only - http://bmjopen.bmj.com/site/about/guidelines.xhtml

"sub optim*" or tapering or withdrawal) N3 (drug* or medicine* or medication* or prescri*))

2. (MH "Inappropriate Prescribing")
3. (MM "Prescriptions, Drug+")
4. TI ((drug* or guideline* or guidance or medicine* or medication* or patient* or prescri*) N3 (adhere* or compliance or concordance)) OR AB ((drug* or guideline* or guidance or medicine* or medication* or patient* or prescri*) N3 (adhere* or compliance or concordance))
5. (MM "Patient Compliance+")
6. TI ((shared or sharing or informed) N1 (decision* or choice*)) OR AB ((shared or sharing or informed) N1 (decision* or choice*))
7. TI (decision N1 (aid* or support*)) OR AB (decision N1 (aid* or support*))
8. (MM "Decision Making")
9. (MM "Decision Support Techniques")
10. TI ((consumer* or patient*) N2 (involv* or participat*)) OR AB ((consumer* or patient*) N2 (involv* or participat*))
11. (MM "Consumer Participation")
12. TI ("patient cent*" N1 (approach* or care or decision* or intervention* or treatment*)) OR AB ("patient cent*" N1 (approach* or care or decision* or intervention* or treatment*))
13. (MH "Patient Centered Care")
14. TI (behavi* N1 chang*) OR AB (behavi* N1 chang*)
15. TI (restriction N1 (policy or policies)) OR AB (restriction N1 (policy or policies))
16. S1 OR S2 OR S3 OR S4 OR S5 OR S6 OR S7 OR S8 OR S9 OR S10 OR S11 OR S12 OR S13 OR S14 OR S15
17. TI benzodiazepine* OR AB benzodiazepine*
18. TI (alprazolam or flunitrazepam or chlordiazepoxide or clobazam or clonazepam or diazepam or lorazepam or midazolam or nitrazepam or oxazepam or prazepam or temazepam) OR AB (alprazolam or flunitrazepam or chlordiazepoxide or clobazam or clonazepam or diazepam or lorazepam or midazolam or nitrazepam or oxazepam or prazepam or temazepam)
19. (MH "Antianxiety Agents, Benzodiazepine+")
20. TI (antidepres* or "anti depres*") OR AB (antidepres* or "anti depres*")
21. TI ((serotonin or norepinephrine or noradrenaline or neurotransmitter* or dopamin* or SSRI* or SNRI* or NARI* or SARI* or NDRI* or tricyclic* or tetracyclic*)) OR AB ((serotonin or norepinephrine or noradrenaline or neurotransmitter* or dopamin* or SSRI* or SNRI* or NARI* or SARI* or NDRI* or tricyclic* or tetracyclic*))

For peer review only - http://bmjopen.bmj.com/site/about/guidelines.xhtml

22. (MH "Antidepressive Agents+")
23. TI ((opioid* or opiate*)) OR AB ((opioid* or opiate*))
24. TI ((morphine or hydromorphone or levorphanol or meperidine or methadone or propoxyphene of codeine or pentazocine or hydrocodone or oxycodone or fentanyl or tramadol)) OR AB ((morphine or hydromorphone or levorphanol or meperidine or methadone or propoxyphene of codeine or pentazocine or hydrocodone or oxycodone or fentanyl or tramadol))
25. (MH "Analgesics, Opioid+")
26. TI "z drug*" OR AB "z drug*"
27. TI ((zopiclone or zolpidem or zaleplon or eszopiclone)) OR AB ((zopiclone or zolpidem or zaleplon or eszopiclone))
28. (MH "Hypnotics and Sedatives")
29. TI ((gabapentin* or mirogabalin or phenibut or pregabalin)) OR AB ((gabapentin* or mirogabalin or phenibut or pregabalin))
30. (MH "Gabapentin")
31. S17 OR S18 OR S19 OR S20 OR S21 OR S22 OR S23 OR S24 OR S25 OR S26 OR S27 OR S28 OR S29 OR S30
32. S16 AND S31
33. TI statin* OR AB statin*
34. TI ("HMG-CoA reductase inhibitor*" or "3-hydroxy-3-methyl-glutaryl-CoA reductase inhibitor*" or "3-hydroxy-3-methylglutaryl coenzyme A reductase inhibitor*" or "HMGCR inhibitor*" or "Hydroxymethylglutaryl-CoA Reductase Inhibitor*") OR AB ("HMG-CoA reductase inhibitor*" or "3-hydroxy-3-methyl-glutaryl-CoA reductase inhibitor*" or "3-hydroxy-3-methylglutaryl coenzyme A reductase inhibitor*" or "HMGCR inhibitor*" or "Hydroxymethylglutaryl-CoA Reductase Inhibitor*")
35. (MH "Statins+")
36. TI (atorvastatin or Lipitor or Torvast) OR AB (atorvastatin or Lipitor or Torvast)
37. TI (fluvastatin or Lescol) OR AB (fluvastatin or Lescol)
38. TI (lovastatin or Mevacor or Altocor or Altoprev) OR AB (lovastatin or Mevacor or Altocor or Altoprev)
39. TI (pitavastatin or Livalo or Pitava) OR AB (pitavastatin or Livalo or Pitava)
40. TI (pravastatin or Pravachol or Selektine or Lipostat) OR AB (pravastatin or Pravachol or Selektine or Lipostat)
41. TI (rosuvastatin or Crestor) OR AB (rosuvastatin or Crestor)
42. TI (simvastatin or Zocor or Lipex) OR AB (simvastatin or Zocor or Lipex)
43. TI antihypertensive* OR AB antihypertensive*

For peer review only

44. (MH "Antihypertensive Agents+")

45. TI ("ACE inhibitor*" or antagonist or "angiotensin II receptor" or "beta blocker*" or "calcium channel blocker*" or "thiazide diuretic*") OR AB ("ACE inhibitor*" or antagonist or "angiotensin II receptor" or "beta blocker*" or "calcium channel blocker*" or "thiazide diuretic*")

46. TI (acebutolol or adrenomedullin or alprenolol or amlodipine or atenolol or bendroflumethiazide or bepridil or betaxolol or bethanidine or bimatoprost or bisoprolol or bosentan or "bretylium tosylate" or brimonidine tartrate or bupranolol or captopril or carteolol or carvedilol or celiprolol or chlorisondamine or chlorothiazide or chlorthalidone or cilazapril or clonidine or cromakalim or cyclopenthiazide or debrisoquin or diazoxide or dihydralazine or dihydroalprenolol or diltiazem or doxazosin or enalapril or enalaprilat or eplerenone or epoprostenol or felodipine or fenoldopam or fosinopril or guanabenz or guanethidine or guanfacine or hexamethonium or "hexamethonium compound*" or hydralazine or hydrochlorothiazide or hydroflumethiazide or indapamide or indoramin or irbesartan or isradipine or kallidin or ketanserin or labetalol or latanoprost or lisinopril or losartan or mecamylamine or methyldopa or metipranolol or metolazone or metoprolol or mibefradil or minoxidil or muzolimine or nadolol or nebivolol or nicardipine or nicorandil or nimodipine or nisoldipine or nitrendipine or nitroprusside or olmesartan medoxomil or oxprenolol or pargyline or pempidine or penbutolol or "pentolinium tartrate" or perindopril or phenoxybenzamine or phentolamine or pinacidil or pindolol or piperoxan or polythiazide or prazosin or propranolol or protoveratrines or quinapril or ramipril or reserpine or rilmenidine or telmisartan or teprotide or terlipressin or ticrynafen or timolol or todralazine or tolazoline or torsemide or travoprost or trichlormethiazide or trimethaphan or valsartan or "veratrum alkaloid*" or vincamine or xipamide) OR AB (acebutolol or adrenomedullin or alprenolol or amlodipine or atenolol or bendroflumethiazide or bepridil or betaxolol or bethanidine or bimatoprost or bisoprolol or bosentan or "bretylium tosylate" or brimonidine tartrate or bupranolol or captopril or carteolol or carvedilol or celiprolol or chlorisondamine or chlorothiazide or chlorthalidone or cilazapril or clonidine or cromakalim or cyclopenthiazide or debrisoquin or diazoxide or dihydralazine or dihydroalprenolol or diltiazem or doxazosin or enalapril or enalaprilat or eplerenone or epoprostenol or felodipine or fenoldopam or fosinopril or guanabenz or guanethidine or guanfacine or hexamethonium or "hexamethonium compound*" or hydralazine or hydrochlorothiazide or hydroflumethiazide or indapamide or indoramin or irbesartan or isradipine or kallidin or ketanserin or labetalol or latanoprost or lisinopril or losartan or mecamylamine or methyldopa or metipranolol or metolazone or metoprolol or mibefradil or minoxidil or muzolimine or nadolol or nebivolol or nicardipine or nicorandil or nimodipine or nisoldipine or nitrendipine or nitroprusside or olmesartan medoxomil or oxprenolol or pargyline or pempidine or penbutolol or "pentolinium tartrate" or perindopril or phenoxybenzamine or phentolamine or pinacidil or pindolol or piperoxan or polythiazide or prazosin or propranolol or protoveratrines or quinapril or ramipril or reserpine or rilmenidine or telmisartan or teprotide or terlipressin or ticrynafen or timolol or todralazine or tolazoline or torsemide or travoprost or trichlormethiazide or trimethaphan or valsartan or "veratrum alkaloid*" or vincamine or xipamide)

47. S33 OR S34 OR S35 OR S36 OR S37 OR S38 OR S39 OR S40 OR S41 OR S42 OR S43 OR S44 OR S45 OR S46

48. TI cardiovascular OR AB cardiovascular

For peer review only - http://bmjopen.bmj.com/site/about/guidelines.xhtml

For peer review only

49. (MH "Cardiovascular Diseases+")
50. TI ((cardiac or coronary or heart) N2 (arrest* or attack* or disease* or failure*)) OR AB ((cardiac or coronary or heart) N2 (arrest* or attack* or disease* or failure*))
51. TI ((heart or myocard* or transient) N2 (infarc* or ischaemi* or ischemi*)) OR AB ((heart or myocard* or transient) N2 (infarc* or ischaemi* or ischemi*))
52. TI angina* OR AB angina*
53. (MH "Angina Pectoris")
54. TI stroke* OR AB stroke*
55. (MH "Stroke+")
56. S48 OR S49 OR S50 OR S51 OR S52 OR S53 OR S54 OR S55
57. S16 AND S47 AND S56
58. TI ((effectiveness or implementation or literature or map or mapping or qualitative or rapid or realist or systematic or scoping or "state of the art" or umbrella) N1 (assessment* or overview* or review* or synthes*)) OR AB ((effectiveness or implementation or literature or map or mapping or qualitative or rapid or realist or systematic or scoping or "state of the art" or umbrella) N1 (assessment* or overview* or review* or synthes*))
59. TI ("meta analy*" or metaanaly* or metasynthe* or "meta synthe*") OR AB ("meta analy*" or metaanaly* or metasynthe* or "meta synthe*")
60. TI ((systematic or evidence) N0 assess*) OR AB ((systematic or evidence) N0 assess*)
61. TI (qualitative N1 (evidence or synthes*)) OR AB (qualitative N1 (evidence or synthes*))
62. TI overarching N1 model OR AB overarching N1 model
63. TI "review* of reviews" OR AB "review* of reviews"
64. S58 OR S59 OR S60 OR S61 OR S62 OR S63
65. S32 AND S64 [Drugs that cause dependency search results]
66. S57 AND S64 [Statins search results]

Notes: Date limited 2010 to date of search.

Database: Epistemonikos
Host: https://www.epistemonikos.org/en/
Issue: n/a
Date Searched: 12/8/2020
Searcher: SB
Hits: 56
Strategy

1. title:(prescription* OR prescribing OR prescribe*) OR abstract:(prescription* OR prescribing OR prescribe*)

For peer review only - http://bmjopen.bmj.com/site/about/guidelines.xhtml

2. title:(optimi* OR inappropriat* OR enhance*) OR abstract:(optimi* OR inappropriat* OR enhance*)

3. title:(benzodiazepine*) OR abstract:(benzodiazepine*) OR title:(antidepres* OR "anti depres*") OR abstract:(antidepres* OR "anti depres*") OR title:(opioid* OR opiate*) OR abstract:(opioid* OR opiate*) OR title:("z drug*") OR abstract:("z drug*") OR title:(gabapentin*) OR abstract:(gabapentin*)

4. 1 AND 2 AND 3

Notes: Date limited 2010 to 2020.

Database: medRxiv
Host: 12/8/2020
Issue: n/a
Date Searched:
Searcher: SB
Hits: 87
Strategy:

optimi* and prescri* and benzodiazepine *	5 hits
optimi* and prescri* and antidepres*	5 hits
optimi* and prescri* and opioid*	11 hits
optimi* and prescri* and "z drug*	66 hits
optimi* and prescri* and gabapentin*	0 hits

Database: Embase
Host: Ovid
Issue: 1974 to 2020 August 10
Date Searched: 11/8/2020
Searcher: SB
Hits: 1776
Strategy:

1. ((appropriate* or discontinu* or enhance* or inappropriate* or incorrect* or "in correct*" or optim* or safe or suboptim* or "sub optim*" or tapering or withdrawal) adj4 (drug* or medicine* or medication* or prescri*)).tw.

2. exp *inappropriate prescribing/

3. *prescription/

4. ((drug* or guideline* or guidance or medicine* or medication* or patient* or prescri*) adj4 (adhere* or compliance or concordance)).tw.

5. exp *patient compliance/

6. medication compliance/

For peer review only - http://bmjopen.bmj.com/site/about/guidelines.xhtml

7. ((shared or sharing or informed) adj2 (decision* or choice*)).tw.
8. (decision adj2 (aid* or support*)).tw.
9. exp *decision making/
10. exp *decision support system/
11. ((consumer* or patient*) adj3 (involv* or participat*)).tw.
12. *patient participation/
13. ("patient cent*" adj2 (approach* or care or decision* or intervention* or treatment*)).tw.
14. *patient care/
15. (behavi* adj2 chang*).tw.
16. *behavior change/
17. (restriction adj2 (policy or policies)).tw.
18. or/1-17
19. benzodiazepine*.tw.
20. (alprazolam or flunitrazepam or chlordiazepoxide or clobazam or clonazepam or diazepam or lorazepam or midazolam or nitrazepam or oxazepam or prazepam or temazepam).tw.
21. exp benzodiazepine derivative/
22. (antidepres* or "anti depres*").tw.
23. (serotonin or norepinephrine or noradrenaline or neurotransmitter* or dopamin* or SSRI* or SNRI* or NARI* or SARI* or NDRI* or tricyclic* or tetracyclic*).tw.
24. exp antidepressant agent/
25. (opioid* or opiate*).tw.
26. (morphine or hydromorphone or levorphanol or meperidine or methadone or propoxyphene of codeine or pentazocine or hydrocodone or oxycodone or fentanyl or tramadol).tw.
27. exp opiate/
28. "z drug*".tw.
29. (zopiclone or zolpidem or zaleplon or eszopiclone).tw.
30. hypnotic agent/
31. sedative agent/
32. (gabapentin* or mirogabalin or phenibut or pregabalin).tw.
33. gabapentin/
34. or/19-33
35. 18 and 34

For peer review only - http://bmjopen.bmj.com/site/about/guidelines.xhtml

For peer review only

36. statin*.tw.

37. ("HMG-CoA reductase inhibitor*" or "3-hydroxy-3-methyl-glutaryl-CoA reductase inhibitor*" or "3-hydroxy-3-methylglutaryl coenzyme A reductase inhibitor*" or "HMGCR inhibitor*" or "Hydroxymethylglutaryl-CoA Reductase Inhibitor*").tw.

38. "statin (protein)"/

39. (atorvastatin or Lipitor or Torvast).tw.

40. (fluvastatin or Lescol).tw.

41. (lovastatin or Mevacor or Altocor or Altoprev).tw.

42. (pitavastatin or Livalo or Pitava).tw.

43. (pravastatin or Pravachol or Selektine or Lipostat).tw.

44. (rosuvastatin or Crestor).tw.

45. (simvastatin or Zocor or Lipex).tw.

46. antihypertensive*.tw.

47. exp antihypertensive agent/

48. ("ACE inhibitor*" or antagonist or "angiotensin II receptor" or "beta blocker*" or "calcium channel blocker*" or "thiazide diuretic*").tw.

49. (acebutolol or adrenomedullin or alprenolol or amlodipine or atenolol or bendroflumethiazide or bepridil or betaxolol or bethanidine or bimatoprost or bisoprolol or bosentan or "bretylium tosylate" or brimonidine tartrate or bupranolol or captopril or carteolol or carvedilol or celiprolol or chlorisondamine or chlorothiazide or chlorthalidone or cilazapril or clonidine or cromakalim or cyclopenthiazide or debrisoquin or diazoxide or dihydralazine or dihydroalprenolol or diltiazem or doxazosin or enalapril or enalaprilat or eplerenone or epoprostenol or felodipine or fenoldopam or fosinopril or guanabenz or guanethidine or guanfacine or hexamethonium or "hexamethonium compound*" or hydralazine or hydrochlorothiazide or hydroflumethiazide or indapamide or indoramin or irbesartan or isradipine or kallidin or ketanserin or labetalol or latanoprost or lisinopril or losartan or mecamylamine or methyldopa or metipranolol or metolazone or metoprolol or mibefradil or minoxidil or muzolimine or nadolol or nebivolol or nicardipine or nicorandil or nimodipine or nisoldipine or nitrendipine or nitroprusside or olmesartan medoxomil or oxprenolol or pargyline or pempidine or penbutolol or "pentolinium tartrate" or perindopril or phenoxybenzamine or phentolamine or pinacidil or pindolol or piperoxan or polythiazide or prazosin or propranolol or protoveratrines or quinapril or ramipril or reserpine or rilmenidine or telmisartan or teprotide or terlipressin or ticrynafen or timolol or todralazine or tolazoline or torsemide or travoprost or trichlormethiazide or trimethaphan or valsartan or "veratrum alkaloid*" or vincamine or xipamide).tw.

50. or/36-49

51. cardiovascular.tw.

52. exp cardiovascular disease/

For peer review only - http://bmjopen.bmj.com/site/about/guidelines.xhtml

53. ((cardiac or coronary or heart) adj3 (arrest* or attack* or disease* or failure*)).tw.
54. ((heart or myocard* or transient) adj3 (infarc* or ischaemi* or ischemi*)).tw.
55. angina*.tw.
56. Angina Pectoris/
57. stroke*.tw.
58. exp cerebrovascular accident/
59. or/51-58
60. 18 and 50 and 59
61. ((effectiveness or implementation or literature or map or mapping or qualitative or rapid or realist or systematic or scoping or "state of the art" or umbrella) adj2 (assessment* or overview* or review* or synthes*)).tw.
62. ("meta analy*" or metaanaly* or metasynthe* or "meta synthe*").tw.
63. ((systematic or evidence) adj1 assess*).tw.
64. (qualitative adj2 (evidence or synthes*)).tw.
65. (overarching adj2 model).tw.
66. "review* of reviews".tw.
67. or/61-66
68. 35 and 67 [Drugs that cause dependency search results]
69. 60 and 67 [Statins search results]

Notes: Date limited 2010 to date of search.

Database: Health Management Information Consortium
Host: Ovid
Issue: 1979 to July 2020
Date Searched: 11/8/2020
Searcher: SB
Hits: 10
Strategy: see MEDLINE search strategy

Database: MEDLINE
Host: Ovid
Issue: 1946 to August 10, 2020
Date Searched: 11/8/2020
Searcher: SB
Hits: 875
Strategy:

For peer review only - http://bmjopen.bmj.com/site/about/guidelines.xhtml

1. ((appropriate* or discontinu* or enhance* or inappropriate* or incorrect* or "in correct*" or optim* or safe or suboptim* or "sub optim*" or tapering or withdrawal) adj4 (drug* or medicine* or medication* or prescri*)).tw.
2. Inappropriate Prescribing/
3. exp *Drug Prescriptions/
4. ((drug* or guideline* or guidance or medicine* or medication* or patient* or prescri*) adj4 (adhere* or compliance or concordance)).tw.
5. exp *Patient Compliance/
6. ((shared or sharing or informed) adj2 (decision* or choice*)).tw.
7. (decision adj2 (aid* or support*)).tw.
8. *Decision Making/
9. *decision support techniques/
10. ((consumer* or patient*) adj3 (involv* or participat*)).tw.
11. *patient participation/
12. ("patient cent*" adj2 (approach* or care or decision* or intervention* or treatment*)).tw.
13. Patient-Centered Care/
14. (behavi* adj2 chang*).tw.
15. (restriction adj2 (policy or policies)).tw.
16. or/1-15
17. benzodiazepine*.tw.
18. (alprazolam or flunitrazepam or chlordiazepoxide or clobazam or clonazepam or diazepam or lorazepam or midazolam or nitrazepam or oxazepam or prazepam or temazepam).tw.
19. exp Benzodiazepines/
20. (antidepres* or "anti depres*").tw.
21. (serotonin or norepinephrine or noradrenaline or neurotransmitter* or dopamin* or SSRI* or SNRI* or NARI* or SARI* or NDRI* or tricyclic* or tetracyclic*).tw.
22. exp Antidepressive Agents/
23. (opioid* or opiate*).tw.
24. (morphine or hydromorphone or levorphanol or meperidine or methadone or propoxyphene of codeine or pentazocine or hydrocodone or oxycodone or fentanyl or tramadol).tw.
25. exp Analgesics, Opioid/
26. "z drug*".tw.
27. (zopiclone or zolpidem or zaleplon or eszopiclone).tw.

For peer review only

For peer review only - http://bmjopen.bmj.com/site/about/guidelines.xhtml

28. exp "hypnotics and sedatives"/

29. (gabapentin* or mirogabalin or phenibut or pregabalin).tw.

30. Gabapentin/

31. or/17-30

32. 16 and 31

33. statin*.tw.

34. ("HMG-CoA reductase inhibitor*" or "3-hydroxy-3-methyl-glutaryl-CoA reductase inhibitor*" or "3-hydroxy-3-methylglutaryl coenzyme A reductase inhibitor*" or "HMGCR inhibitor*" or "Hydroxymethylglutaryl-CoA Reductase Inhibitor*").tw.

35. exp Hydroxymethylglutaryl-CoA Reductase Inhibitors/

36. (atorvastatin or Lipitor or Torvast).tw.

37. (fluvastatin or Lescol).tw.

38. (lovastatin or Mevacor or Altocor or Altoprev).tw.

39. (pitavastatin or Livalo or Pitava).tw.

40. (pravastatin or Pravachol or Selektine or Lipostat).tw.

41. (rosuvastatin or Crestor).tw.

42. (simvastatin or Zocor or Lipex).tw.

43. antihypertensive*.tw.

44. exp Antihypertensive Agents/

45. ("ACE inhibitor*" or antagonist or "angiotensin II receptor" or "beta blocker*" or "calcium channel blocker*" or "thiazide diuretic*").tw.

46. (acebutolol or adrenomedullin or alprenolol or amlodipine or atenolol or bendroflumethiazide or bepridil or betaxolol or bethanidine or bimatoprost or bisoprolol or bosentan or "bretylium tosylate" or brimonidine tartrate or bupranolol or captopril or carteolol or carvedilol or celiprolol or chlorisondamine or chlorothiazide or chlorthalidone or cilazapril or clonidine or cromakalim or cyclopenthiazide or debrisoquin or diazoxide or dihydralazine or dihydroalprenolol or diltiazem or doxazosin or enalapril or enalaprilat or eplerenone or epoprostenol or felodipine or fenoldopam or fosinopril or guanabenz or guanethidine or guanfacine or hexamethonium or "hexamethonium compound*" or hydralazine or hydrochlorothiazide or hydroflumethiazide or indapamide or indoramin or irbesartan or isradipine or kallidin or ketanserin or labetalol or latanoprost or lisinopril or losartan or mecamylamine or methyldopa or metipranolol or metolazone or metoprolol or mibefradil or minoxidil or muzolimine or nadolol or nebivolol or nicardipine or nicorandil or nimodipine or nisoldipine or nitrendipine or nitroprusside or olmesartan medoxomil or oxprenolol or pargyline or pempidine or penbutolol or "pentolinium tartrate" or perindopril or phenoxybenzamine or phentolamine or pinacidil or pindolol or piperoxan or polythiazide or prazosin or propranolol or protoveratrines or quinapril or ramipril or reserpine or rilmenidine or telmisartan or teprotide or terlipressin or ticrynafen or timolol or todralazine

For peer review only - http://bmjopen.bmj.com/site/about/guidelines.xhtml

or tolazoline or torsemide or travoprost or trichlormethiazide or trimethaphan or valsartan or "veratrum alkaloid*" or vincamine or xipamide).tw.

47. or/33-46
48. cardiovascular.tw.
49. exp Cardiovascular Diseases/
50. ((cardiac or coronary or heart) adj3 (arrest* or attack* or disease* or failure*)).tw.
51. ((heart or myocard* or transient) adj3 (infarc* or ischaemi* or ischemi*)).tw.
52. angina*.tw.
53. Angina Pectoris/
54. stroke*.tw.
55. exp Stroke/
56. or/48-55
57. 16 and 47 and 56
58. ((effectiveness or implementation or literature or map or mapping or qualitative or rapid or realist or systematic or scoping or "state of the art" or umbrella) adj2 (assessment* or overview* or review* or synthes*)).tw.
59. ("meta analy*" or metaanaly* or metasynthe* or "meta synthe*").tw.
60. ((systematic or evidence) adj1 assess*).tw.
61. (qualitative adj2 (evidence or synthes*)).tw.
62. (overarching adj2 model).tw.
63. "review* of reviews".tw.
64. systematic review.pt.
65. meta-analysis.pt.
66. or/58-65
67. 32 and 66 [Drugs that cause dependency search results]
68. 57 and 66 [Statins search results]

Notes: Date limited 2010 to date of search.

Database: PsycInfo
Host: Ovid
Issue: 1806 to August Week 1 2020
Date Searched: 11/8/2020
Searcher: SB

For peer review only - http://bmjopen.bmj.com/site/about/guidelines.xhtml

For peer review only

Hits: 297
Strategy

1. ((appropriate* or discontinu* or enhance* or inappropriate* or incorrect* or "in correct*" or optim* or safe or suboptim* or "sub optim*" or tapering or withdrawal) adj4 (drug* or medicine* or medication* or prescri*)).tw.
2. prescription drug misuse/
3. exp "prescribing (drugs)"/
4. ((drug* or guideline* or guidance or medicine* or medication* or patient* or prescri*) adj4 (adhere* or compliance or concordance)).tw.
5. *treatment compliance/
6. ((shared or sharing or informed) adj2 (decision* or choice*)).tw.
7. (decision adj2 (aid* or support*)).tw.
8. Decision Making/
9. ((consumer* or patient*) adj3 (involv* or participat*)).tw.
10. patient participation/
11. ("patient cent*" adj2 (approach* or care or decision* or intervention* or treatment*)).tw.
12. (behavi* adj2 chang*).ti,ab.
13. exp behavior change/
14. (restriction adj2 (policy or policies)).tw.
15. or/1-14
16. benzodiazepine*.tw.
17. (alprazolam or flunitrazepam or chlordiazepoxide or clobazam or clonazepam or diazepam or lorazepam or midazolam or nitrazepam or oxazepam or prazepam or temazepam).tw.
18. exp Benzodiazepines/
19. (antidepres* or "anti depres*").tw.
20. (serotonin or norepinephrine or noradrenaline or neurotransmitter* or dopamin* or SSRI* or SNRI* or NARI* or SARI* or NDRI* or tricyclic* or tetracyclic*).tw.
21. exp antidepressant drugs/
22. (opioid* or opiate*).tw.
23. (morphine or hydromorphone or levorphanol or meperidine or methadone or propoxyphene of codeine or pentazocine or hydrocodone or oxycodone or fentanyl or tramadol).tw.
24. exp opiates/
25. "z drug*".tw.

For peer review only - http://bmjopen.bmj.com/site/about/guidelines.xhtml

26. (zopiclone or zolpidem or zaleplon or eszopiclone).tw.
27. exp hypnotic drugs/
28. exp sedatives/
29. (gabapentin* or mirogabalin or phenibut or pregabalin).tw.
30. Gabapentin/
31. or/16-30
32. 15 and 31
33. statin*.tw.
34. ("HMG-CoA reductase inhibitor*" or "3-hydroxy-3-methyl-glutaryl-CoA reductase inhibitor*" or "3-hydroxy-3-methylglutaryl coenzyme A reductase inhibitor*" or "HMGCR inhibitor*" or "Hydroxymethylglutaryl-CoA Reductase Inhibitor*").tw.
35. statins/
36. (atorvastatin or Lipitor or Torvast).tw.
37. (fluvastatin or Lescol).tw.
38. (lovastatin or Mevacor or Altocor or Altoprev).tw.
39. (pitavastatin or Livalo or Pitava).tw.
40. (pravastatin or Pravachol or Selektine or Lipostat).tw.
41. (rosuvastatin or Crestor).tw.
42. (simvastatin or Zocor or Lipex).tw.
43. antihypertensive*.tw.
44. exp antihypertensive drugs/
45. ("ACE inhibitor*" or antagonist or "angiotensin II receptor" or "beta blocker*" or "calcium channel blocker*" or "thiazide diuretic*").tw.
46. (acebutolol or adrenomedullin or alprenolol or amlodipine or atenolol or bendroflumethiazide or bepridil or betaxolol or bethanidine or bimatoprost or bisoprolol or bosentan or "bretylium tosylate" or brimonidine tartrate or bupranolol or captopril or carteolol or carvedilol or celiprolol or chlorisondamine or chlorothiazide or chlorthalidone or cilazapril or clonidine or cromakalim or cyclopenthiazide or debrisoquin or diazoxide or dihydralazine or dihydroalprenolol or diltiazem or doxazosin or enalapril or enalaprilat or eplerenone or epoprostenol or felodipine or fenoldopam or fosinopril or guanabenz or guanethidine or guanfacine or hexamethonium or "hexamethonium compound*" or hydralazine or hydrochlorothiazide or hydroflumethiazide or indapamide or indoramin or irbesartan or isradipine or kallidin or ketanserin or labetalol or latanoprost or lisinopril or losartan or mecamylamine or methyldopa or metipranolol or metolazone or metoprolol or mibefradil or minoxidil or muzolimine or nadolol or nebivolol or nicardipine or nicorandil or nimodipine or nisoldipine or nitrendipine or nitroprusside or olmesartan medoxomil or oxprenolol or pargyline or pempidine or penbutolol or "pentolinium tartrate" or perindopril

For peer review only - http://bmjopen.bmj.com/site/about/guidelines.xhtml

or phenoxybenzamine or phentolamine or pinacidil or pindolol or piperoxan or polythiazide or prazosin or propranolol or protoveratrines or quinapril or ramipril or reserpine or rilmenidine or telmisartan or teprotide or terlipressin or ticrynafen or timolol or todralazine or tolazoline or torsemide or travoprost or trichlormethiazide or trimethaphan or valsartan or "veratrum alkaloid*" or vincamine or xipamide).tw.

47. or/33-46
48. cardiovascular.tw.
49. exp cardiovascular disorders/
50. ((cardiac or coronary or heart) adj3 (arrest* or attack* or disease* or failure*)).tw.
51. ((heart or myocard* or transient) adj3 (infarc* or ischaemi* or ischemi*)).tw.
52. angina*.tw.
53. Angina Pectoris/
54. stroke*.tw.
55. exp cerebral ischemia/
56. or/48-55
57. 15 and 47 and 56
58. ((effectiveness or implementation or literature or map or mapping or qualitative or rapid or realist or systematic or scoping or "state of the art" or umbrella) adj2 (assessment* or overview* or review* or synthes*)).tw.
59. ("meta analy*" or metaanaly* or metasynthe* or "meta synthe*").tw.
60. ((systematic or evidence) adj1 assess*).tw.
61. (qualitative adj2 (evidence or synthes*)).tw.
62. (overarching adj2 model).tw.
63. "review* of reviews".tw.
64. systematic review.pt.
65. meta-analysis.pt.
66. or/58-65
67. 32 and 66 [Drugs that cause dependency search results]
68. 57 and 66 [Statins search results]

Notes: Date limited 2010 to date of search.

Database: Conference Proceedings Citation Index – Science (CPCI-S); Science Citation Index (SCI)
Host: Web of Science via Clarivate Analytics
Issue: n/a
Date Searched: 11/8/2020

For peer review only - http://bmjopen.bmj.com/site/about/guidelines.xhtml
For peer review only

Searcher: SB
Hits: 1030
Strategy:

1. TOPIC =((appropriate* or discontinu* or enhance* or inappropriate* or incorrect* or "in correct*" or optim* or safe or suboptim* or "sub optim*" or tapering or withdrawal) near/3 (drug* or medicine* or medication* or prescri*))
2. TOPIC =((drug* or guideline* or guidance or medicine* or medication* or patient* or prescri*) near/3 (adhere* or compliance or concordance))
3. TOPIC =((shared or sharing or informed) near/1 (decision* or choice*))
4. TOPIC =((consumer* or patient*) near/2 (involv* or participat*))
5. TOPIC =("patient cent*" near/1 (approach* or care or decision* or intervention* or treatment*))
6. TOPIC =(decision near/1 (aid* or support*))
7. TOPIC =(behavi* near/1 chang*)
8. TOPIC =(restriction near/1 (policy or policies))
9. #8 OR #7 OR #6 OR #5 OR #4 OR #3 OR #2 OR #1
10. TOPIC: (benzodiazepine*)
11. TOPIC: ((alprazolam or flunitrazepam or chlordiazepoxide or clobazam or clonazepam or diazepam or lorazepam or midazolam or nitrazepam or oxazepam or prazepam or temazepam))
12. TOPIC: ((antidepres* or "anti depres*"))
13. TOPIC: ((serotonin or norepinephrine or noradrenaline or neurotransmitter* or dopamin* or SSRI* or SNRI* or NARI* or SARI* or NDRI* or tricyclic* or tetracyclic*))
14. TOPIC: ((opioid* or opiate*))
15. TOPIC: ((morphine or hydromorphone or levorphanol or meperidine or methadone or propoxyphene of codeine or pentazocine or hydrocodone or oxycodone or fentanyl or tramadol))
16. TOPIC: ("z drug*")
17. TOPIC: ((zopiclone or zolpidem or zaleplon or eszopiclone))
18. TOPIC: ((gabapentin* or mirogabalin or phenibut or pregabalin))
19. #18 OR #17 OR #16 OR #15 OR #14 OR #13 OR #12 OR #11 OR #10
20. #19 AND #9
21. TOPIC: (statin*)
22. TOPIC: ("HMG-CoA reductase inhibitor*" or "3-hydroxy-3-methyl-glutaryl-CoA reductase inhibitor*" or "3-hydroxy-3-methylglutaryl coenzyme A reductase inhibitor*" or "HMGCR inhibitor*" or "Hydroxymethylglutaryl-CoA Reductase Inhibitor*")

For peer review only - http://bmjopen.bmj.com/site/about/guidelines.xhtml

23. TOPIC: (atorvastatin or Lipitor or Torvast)
24. TOPIC: (fluvastatin or Lescol)
25. TOPIC: (lovastatin or Mevacor or Altocor or Altoprev)
26. TOPIC: (pitavastatin or Livalo or Pitava)
27. TOPIC: (pravastatin or Pravachol or Selektine or Lipostat)
28. TOPIC: (rosuvastatin or Crestor)
29. TOPIC: (simvastatin or Zocor or Lipex)
30. TOPIC: (antihypertensive*)
31. TOPIC: ("ACE inhibitor*" or antagonist or "angiotensin II receptor" or "beta blocker*" or "calcium channel blocker*" or "thiazide diuretic*")
32. TOPIC: (acebutolol or adrenomedullin or alprenolol or amlodipine or atenolol or bendroflumethiazide or bepridil or betaxolol or bethanidine or bimatoprost or bisoprolol or bosentan or "bretylium tosylate" or brimonidine tartrate or bupranolol or captopril or carteolol or carvedilol or celiprolol or chlorisondamine or chlorothiazide or chlorthalidone or cilazapril or clonidine or cromakalim or cyclopenthiazide or debrisoquin or diazoxide or dihydralazine or dihydroalprenolol or diltiazem or doxazosin or enalapril or enalaprilat or eplerenone or epoprostenol or felodipine or fenoldopam or fosinopril or guanabenz or guanethidine or guanfacine or hexamethonium or "hexamethonium compound*" or hydralazine or hydrochlorothiazide or hydroflumethiazide or indapamide or indoramin or irbesartan or isradipine or kallidin or ketanserin or labetalol or latanoprost or lisinopril or losartan or mecamylamine or methyldopa or metipranolol or metolazone or metoprolol or mibefradil or minoxidil or muzolimine or nadolol or nebivolol or nicardipine or nicorandil or nimodipine or nisoldipine or nitrendipine or nitroprusside or olmesartan medoxomil or oxprenolol or pargyline or pempidine or penbutolol or "pentolinium tartrate" or perindopril or phenoxybenzamine or phentolamine or pinacidil or pindolol or piperoxan or polythiazide or prazosin or propranolol or protoveratrines or quinapril or ramipril or reserpine or rilmenidine or telmisartan or teprotide or terlipressin or ticrynafen or timolol or todralazine or tolazoline or torsemide or travoprost or trichlormethiazide or trimethaphan or valsartan or "veratrum alkaloid*" or vincamine or xipamide)
33. #32 OR #31 OR #30 OR #29 OR #28 OR #27 OR #26 OR #25 OR #24 OR #23 OR #22 OR #21
34. TOPIC =((cardiac or coronary or heart) near/2 (arrest* or attack* or disease* or failure*))
35. TOPIC: (cardiovascular)
36. TS=((heart or myocard* or transient) near/2 (infarc* or ischaemi* or ischemi*))
37. TOPIC =angina*
38. TOPIC =stroke*
39. #38 OR #37 OR #36 OR #35 OR #34

For peer review only - http://bmjopen.bmj.com/site/about/guidelines.xhtml

40. TOPIC =((effectiveness or implementation or literature or map or mapping or qualitative or rapid or realist or systematic or scoping or "state of the art" or umbrella) near/1 (assessment* or overview* or review* or synthes*))
41. TOPIC =("meta analy*" or metaanaly* or metasynthe* or "meta synthe*")
42. TOPIC =((systematic or evidence) near/0 assess*)
43. TOPIC =(qualitative near/1 (evidence or synthes*))
44. TOPIC =(overarching near/1 model)
45. TOPIC ="review* of reviews"
46. #45 OR #44 OR #43 OR #42 OR #41 OR #40
47. #46 AND #20 [Drugs that cause dependency search results]
48. #46 AND #39 AND #33 AND #9 [Statins search results]

Table 1: Drugs that can cause dependency and antidepressant search results

Database	Hits
**Cochrane Database of Systematic Reviews**	111
**CINAHL**	372
**Epistemonikos**	56
**medRxiv**	87
**Embase**	1776
**HMIC**	10
**MEDLINE**	875
**PsycInfo**	297
**Web of Science (CPCI-S/SCI)**	1030
**TOTAL RECORDS**	**4614**
**DUPLICATE RECORDS**	**1898**
**UNIQUE RECORDS**	**2716**

Statins

Database: Cochrane Database of Systematic Reviews
Host: Cochrane Library
Issue: Issue 8 of 12, August 2020
Date Searched: 10/8/2020
Searcher: SB
Hits: 33
Strategy: see line 57 of Cochrane Database search above.
Notes: date limited 2010 to date of search.

Database: CINAHL
Host: EBSCO
Issue: n/a
Date Searched: 10/8/2020
Searcher: SB

For peer review only - http://bmjopen.bmj.com/site/about/guidelines.xhtml

Hits: 173
Strategy: see line 66 of CINAHL search above.
Notes: Date limited 2010 to date of search.

Database: Epistemonikos
Host: https://www.epistemonikos.org/en/
Issue: n/a
Date Searched: 10/8/2020
Searcher: SB
Hits: 16
Strategy:

1. title:(prescription* OR prescribing OR prescribe*) OR abstract:(prescription* OR prescribing OR prescribe*)
2. title:(optimi* OR inappropriat* OR enhance*) OR abstract:(optimi* OR inappropriat* OR enhance*)
3. title:(statin*) OR abstract:(statin*) OR title:(antihypertensive*) OR abstract:(antihypertensive*)
4. 1 AND 2 AND 3 [Statins search results]

Notes: Date limited 2010 to 2020 and limited to systematic reviews publication type.

Database: medRxiv
Host: https://www.medrxiv.org/
Issue: n/a
Date Searched: 10/8/2020
Searcher: SB
Hits: 8
Strategy:

optimi* and prescri* and statin*	7 hits
optimi* and prescri* and antihypertensive*	1 hit

Notes: Date limited 2010 to date of search.

Database: Embase
Host: Ovid
Issue: 1974 to 2020 August 07
Date Searched: 10/8/2020
Searcher: SB
Hits: 903
Strategy: see line 69 of Embase search above.
Notes: Date limited 2010 to date of search.

Database: HMIC
Host: Ovid
Issue: 1979 to May 2020
Date Searched: 10/8/2020

For peer review only - http://bmjopen.bmj.com/site/about/guidelines.xhtml

Searcher: SB
Hits: 0
Strategy: see line 68 of MEDLINE search strategy above.
Notes: Date limited 2010 to date of search.

Database: MEDLINE ALL
Host: Ovid
Issue: 1946 to August 07, 2020
Date Searched: 10/8/2020
Searcher: SB
Hits: 371
Strategy: see line 68 of MEDLINE search strategy above.
Notes: Date limited 2010 to date of search.

Database: PsycInfo
Host: Ovid
Issue: 1806 to August Week 1 2020
Date Searched: 10/8/2020
Searcher: SB
Hits: 17
Strategy: see line 68 of PsycInfo search strategy above.
Notes: Date limited 2010 to date of search.

Database: Conference Proceedings Citation Index – Science (CPCI-S); Science Citation Index (SCI)
Host: Web of Science via Clarivate Analytics
Issue: n/a
Date Searched: 10/8/2020
Searcher: SB
Hits: 638
Strategy: see line 48 of CPCI-S/SCI search strategy above.

Table 2: Statin search results

Database	Hits
**Cochrane Database of Systematic Reviews**	33
**CINAHL**	173
**Epistemonikos**	16
**medRxiv**	8
**EMBASE**	903
**HMIC**	0
**MEDLINE**	371
**PsycInfo**	17
**Web of Science (CPCI-S/SCI)**	638
**TOTAL RECORDS**	**2159**
**DUPLICATE RECORDS**	**714**
**UNIQUE RECORDS**	**1445**

Website searches

For peer review only - http://bmjopen.bmj.com/site/about/guidelines.xhtml

DCDs

Website: Royal Pharmaceutical Society Library Catalogue
URL: https://rpsgb.koha-ptfs.co.uk/
Date Searched: 21/10/2020
Searcher: SB
Hits: 73 hits
Strategy:

Advanced search setting.

Keyword: opioid* OR

Keyword: benzodiazepine* OR

Keyword: antidepressant* OR

Keyword: gabapentinoid*

Website: Royal College of Physicians Library Catalogue
URL: https://rcp.soutron.net/Portal/Default/en-GB/Search/SimpleSearch
Date Searched: 21/10/2020
Searcher: SB
Hits: 6
Strategy:

All fields: opioid OR opioids OR benzodiazepine OR benzodiazepines OR antidepressant OR antidepressants OR gabapentinoid OR gabapentinoids

Website: Royal College of Psychiatrists Library Catalogue
URL: https://rcpsych.soutron.net/Portal/Default/en-GB/Search/SimpleSearch
Date Searched: 21/10/2020
Searcher: SB
Hits: 20
Strategy:

opioid* OR benzodiazepine* OR antidepressant* OR gabapentinoid*

Website: Mind
URL: https://www.mind.org.uk/
Date Searched: 21/10/2020
Searcher: SB
Hits: 0
Strategy: Browsed website for relevant studies

Website: Mental Health UK
URL: https://mentalhealth-uk.org/

For peer review only - http://bmjopen.bmj.com/site/about/guidelines.xhtml

For peer review only

Date Searched: 21/10/2020
Searcher: SB
Hits: 0
Strategy: Browsed website for relevant studies

Website: Priory Group
URL: https://www.priorygroup.com/
Date Searched: 21/10/2020
Searcher: SB
Hits: 0
Strategy: Browsed website for relevant studies

Statins

Website: Royal Pharmaceutical Society Library Catalogue
URL: https://rpsgb.koha-ptfs.co.uk/
Date Searched: 21/10/2020
Searcher: SB
Hits: 13
Strategy:

Advanced search setting.

Keyword:	statin*	OR
Keyword:	“ace inhibitor*”	OR
Keyword:	antihypertensive*	

Website: Royal College of Physicians Library Catalogue
URL: https://rcp.soutron.net/Portal/Default/en-GB/Search/SimpleSearch
Date Searched: 21/10/2020
Searcher: SB
Hits: 6
Strategy:

All fields: statin OR statins OR "ace inhibitor" OR "ace inhibitors" OR antihypertensive OR antihypertensives

Website: British Cardiovascular Society
URL: https://www.britishcardiovascularsociety.org/
Date Searched: 21/10/2020
Searcher: SB
Hits: 0
Strategy: Browsed website for relevant studies.

For peer review only - http://bmjopen.bmj.com/site/about/guidelines.xhtml

Website: European Society of Cardiology
URL: https://www.escardio.org/
Date Searched: 21/10/2020
Searcher: SB
Hits: 0
Strategy: Browsed website for relevant studies

Website: British and Irish Hypertension Society
URL: https://bihsoc.org/
Date Searched: 21/10/2020
Searcher: SB
Hits: 0
Strategy: Browsed website for relevant studies

Website: British Heart Foundation
URL: https://www.bhf.org.uk/
Date Searched: 21/10/2020
Searcher: SB
Hits: 31 (sum total of each keyword search)
Strategy: Searched for keywords “statin”, “ace inhibitor” or “antihypertensive” (one term per search) and viewed the publications and research tabs.

Website: Heart Research UK
URL: https://heartresearch.org.uk/
Date Searched: 21/10/2020
Searcher: SB
Hits: 0
Strategy: Browsed website for relevant studies

For peer review only

APPENDIX B: RESULTS FROM UPDATE SEARCHES

Summary of systematic reviews (n=12) identified for inclusion following update searches conducted 2nd August 2022.

Author	Year	CEESAT rating (n positive of 4 items)	Topic [Type of evidence]	Medication of interest	Care pathway
Bond	2021	2	Evaluating intervention to optimise patient adherence to a medication [Quantitative]	Statins	Maintaining treatment
Coronado-Vázquez	2020	3	Evaluating intervention to optimise patient adherence to a medication [Quantitative]	Statins, Antihypertensives	Maintaining treatment
Guzman-Tordecilla*	2020	1	Evaluating intervention to optimise patient adherence to a medication [Quantitative]	Antihypertensives	Maintaining treatment
Hong	2022	2	Evaluating intervention to optimise patient adherence to a medication [Quantitative]	Antihypertensives	Maintaining treatment
Nili	2020	4	Evaluating intervention to optimise patient adherence to a medication [Quantitative]	Antihypertensives	Maintaining treatment
Palmer*	2021	4	Evaluating intervention to optimise patient adherence to a medication [Quantitative]	Statins, Antihypertensives	Maintaining treatment

For peer review only - http://bmjopen.bmj.com/site/about/guidelines.xhtml

Parati	2021	3	Evaluating intervention to optimise patient adherence to a medication [Quantitative]	Antihypertensives	Maintaining treatment
Qadi	2020	3	Prescriber, patient, and/or family/carer views of issues relating to prescribing and/or adherence to medications of interest [Qualitative]	Statins, Antihypertensives	Pre-treatment/ initiation,
Reston	2020	3	Evaluating intervention to optimise patient adherence to a medication [Quantitative]	Statin	Maintaining treatment
Van Truong	2021	3	Evaluating intervention to optimise patient adherence to a medication [Quantitative]	Antihypertensives	Maintaining treatment
Vervloet	2020	3	Evaluating intervention to optimise patient adherence to a medication [Quantitative]	Statin Antihypertensives	Maintaining treatment
Xie	2022	3	Evaluating intervention to optimise prescribing [Quantitative]	Statin, Antihypertensives	Pre-treatment/ initiation, Maintaining treatment

*Updated of review identified by original searches, CEESAT=Collaboration for Environmental Evidence Synthesis Assessment Tool, N=Number

For peer review only - http://bmjopen.bmj.com/site/about/guidelines.xhtml

APPENDIX C: REASONS FOR EXCLUSION AT FULL TEXT

Reference	Reason for exclusion
Abstracts from Professional Poster Presentations at AMCP's 2010 Educational Conference. *Journal of Managed Care Pharmacy* 2010;**16**.	Abstract proceedings
The Canadian Association for Population Therapeutics. November 17th - 19th, 2013 Toronto, Ontario. "Healthcare Cost, Quality, and Policy: Driving Stakeholder Innovation in Process and Practice". *Journal of Population Therapeutics and Clinical Pharmacology* 2014;**21**:e116.	Abstract proceedings
Transcatheter Cardiovascular Therapeutics Asia Pacific, 19th Cardiovascular Summit: TCTAP 2014. *Journal of the American College of Cardiology* 2014;**63**.	Abstract proceedings
Abstracts of Papers Presented at the Health Services Research and Pharmacy Practice Conference, HSRPP 2016. *International Journal of Pharmacy Practice* 2016;**24**.	Abstract proceedings
12th Annual Scientific Meeting of Indonesian Society of Hypertension, INASH. *Journal of Hypertension* 2018;**36**.	Abstract proceedings
Selected abstracts from the EGPRN meeting in Tampere, Finland, 9–12 May 2019. *European Journal of General Practice* 2019;**25**:164-75.	Abstract proceedings
46th ESAO Congress 3-7 September 2019 Hannover, Germany Abstracts. *International Journal of Artificial Organs* 2019;**42**:386-474.	Abstract proceedings
AbuDagga A, Resnick HE, Alwan M. Impact of blood pressure telemonitoring on hypertension outcomes: a literature review. *Telemed J E Health* 2010;**16**:830-8. https://doi.org/10.1089/tmj.2010.0015	Not SR
Akeroyd JM, Chan WJ, Kamal AK, Palaniappan L, Virani SS. Adherence to cardiovascular medications in the South Asian population: A systematic review of current evidence and future directions. *World J Cardiol* 2015;**7**:938-47. https://doi.org/10.4330/wjc.v7.i12.938	Not SR
Al AlShaikh S, Quinn T, Dunn W, Dawson J. Interventions to Enhance Adherence to Secondary Preventative Medication After Stroke a Systematic Review and Meta-Analysis. *Stroke* 2016;**47**.	Abstract
Alford DP. Chronic back pain with possible prescription opioid misuse. *JAMA* 2013;**309**:919-25. https://doi.org/10.1001/jama.2013.522	Not SR
AlGhurair SA, Hughes CA, Simpson SH, Guirguis LM. A systematic review of patient self-reported barriers of adherence to antihypertensive medications using the world health organization multidimensional adherence model. *J Clin Hypertens (Greenwich)* 2012;**14**:877-86. https://doi.org/10.1111/j.1751-7176.2012.00699.x	Not SR

For peer review only - http://bmjopen.bmj.com/site/about/guidelines.xhtml

Alkhouli M, Noseworthy PA, Rihal CS, Holmes DR, Jr. Stroke Prevention in Nonvalvular Atrial Fibrillation: A Stakeholder Perspective. *J Am Coll Cardiol* 2018;**71**:2790-801. https://doi.org/10.1016/j.jacc.2018.04.013	Not SR
Almeida JT, Esteves AL, Martins F, Palma I. [Approach to Patients with Statin Intolerance: Evidence-Based Review]. *Acta Med Port* 2020;**33**:49-57. https://doi.org/10.20344/amp.10376	Non-English language (Duplicated excluded from update search)
AlShaikh SA, Quinn T, Dunn W, Dawson J. Interventions to enhance adherence to secondary preventative medication after stroke a systematic review and meta-analysis. *Stroke* 2016;**47**.	Duplicate
Anderson SL, Marrs JC. A Review of the Role of the Pharmacist in Heart Failure Transition of Care. *Adv Ther* 2018;**35**:311-23. https://doi.org/10.1007/s12325-018-0671-7	Not SR
Anonymous. Review of: Fuller RH et al. Improving adherence to medicines in people with heart disease: a systematic review. Heart. *Drug Ther Bull* 2018;**56**:114. https://doi.org/10.1136/dtb.2018.10.000021	Not SR
Armstrong SO, Little RA. Cost effectiveness of interventions to improve adherence to statin therapy in ASCVD patients in the United States. *Patient Prefer Adherence* 2019;**13**:1375-89. https://doi.org/10.2147/PPA.S213258	Not SR
Banerjee A, Khandelwal S, Nambiar L, Saxena M, Peck V, Moniruzzaman M*, et al.* Health system barriers and facilitators to medication adherence for the secondary prevention of cardiovascular disease: a systematic review. *Open Heart* 2016;**3**:e000438. https://doi.org/10.1136/openhrt-2016-000438	Quantitative focus
Basaraba JE, Picard M, George-Phillips K, Mysak T. Pharmacists as Care Providers for Stroke Patients: A Systematic Review. *Can J Neurol Sci* 2018;**45**:49-55. https://doi.org/10.1017/cjn.2017.233	Not SR
Bramlage P, Sims H, Minguet J, Ferrero C. The polypill: An effective approach to increasing adherence and reducing cardiovascular event risk. *Eur J Prev Cardiol* 2017;**24**:297-310. https://doi.org/10.1177/2047487316674817	Not SR
Brito JP, Montori VM. Reinitiation of statins after statin-associated musculoskeletal symptoms: a patient-centered approach. *Circ Cardiovasc Qual Outcomes* 2013;**6**:243-7. https://doi.org/10.1161/CIRCOUTCOMES.111.000039	Not SR
Buck HG, McGhee S, Polo RL, Zambroski C. Hypercholesterolemia Management in Older Adults: A Scoping Review of Recent Evidence. *J Gerontol Nurs* 2019;**45**:31-42. https://doi.org/10.3928/00989134-20190211-04	Quantitative focus
Calleja Rivero JL, Zerpa F, Rivera L. Fixed-dose versus separate drug combinations for antihypertensive treatment: literature review. *Medwave* 2016;**16**:e6549. https://doi.org/10.5867/medwave.2016.08.6549	Not SR

For peer review only - http://bmjopen.bmj.com/site/about/guidelines.xhtml

Campos K, Sheth S, Coulter SA. Hypertension Treatment ACCORDing to SPRINT. *Tex Heart Inst J* 2016;**43**:324-7. https://doi.org/10.14503/THIJ-16-5908	Not SR
Chan DK, O'Rourke F, Shen Q, Mak JC, Hung WT. Meta-analysis of the cardiovascular benefits of intensive lipid lowering with statins. *Acta Neurol Scand* 2011;**124**:188-95. https://doi.org/10.1111/j.1600-0404.2010.01450.x	Quantitative focus
Chapman RH, Ferrufino CP, Kowal SL, Classi P, Roberts CS. The cost and effectiveness of adherence-improving interventions for antihypertensive and lipid-lowering drugs*. *Int J Clin Pract* 2010;**64**:169-81. https://doi.org/10.1111/j.1742-1241.2009.02196.x	Not SR
Chee YJ, Chan HH, Tan NC. Understanding patients' perspective of statin therapy: can we design a better approach to the management of dyslipidaemia? A literature review. *Singapore Med J* 2014;**55**:416-21. https://doi.org/10.11622/smedj.2014099	Not SR
Choi H-I, Han KH. The ideal management of blood cholesterol: a review of the updated 2013 ACC/AHA cholesterol guideline for reducing cardiovascular risk. *Journal of the Korean Medical Association* 2014;**57**:857-65. https://doi.org/10.5124/jkma.2014.57.10.857	Non-English language
Clarkesmith DE, Pattison HM, Khaing PH, Lane DA. Educational and behavioural interventions for anticoagulant therapy in patients with atrial fibrillation. *Cochrane Database of Systematic Reviews* 2017; 10.1002/14651858.CD008600.pub3. https://doi.org/10.1002/14651858.CD008600.pub3	Drug not relevant
Coleman CI, Roberts MS, Sobieraj DM, Lee S, Alam T, Kaur R. Effect of dosing frequency on chronic cardiovascular disease medication adherence. *Curr Med Res Opin* 2012;**28**:669-80. https://doi.org/10.1185/03007995.2012.677419	Not SR
Conn VS, Ruppar TM, Chase JD. Blood pressure outcomes of medication adherence interventions: systematic review and meta-analysis. *J Behav Med* 2016;**39**:1065-75. https://doi.org/10.1007/s10865-016-9730-1	Quantitative focus
Cooke CE, Xing S, Gale SE, Peters S. Initial non-adherence to antihypertensive medications in the United States: a systematic literature review. Journal of human hypertension 2022;36:3-13. https://doi.org/https://dx.doi.org/10.1038/s41371-021-00549-w	Not SR
Crisafulli S, Luxi N, Coppini R, Capuano A, Scavone C, Zinzi A, et al. Anti-hypertensive drugs deprescribing: an updated systematic review of clinical trials. BMC family practice 2021;22:208. https://doi.org/https://dx.doi.org/10.1186/s12875-021-01557-y	Not SR
Cutrona SL, Choudhry NK, Fischer MA, Servi A, Liberman JN, Brennan TA*, et al.* Modes of delivery for interventions to improve cardiovascular medication adherence. *Am J Manag Care* 2010;**16**:929-42.	Not SR

For peer review only

For peer review only - http://bmjopen.bmj.com/site/about/guidelines.xhtml

de Barra M, Scott CL, Scott NW, Johnston M, de Bruin M, Nkansah N*, et al.* Pharmacist services for non-hospitalised patients. *Cochrane Database Syst Rev* 2018;**9**:CD013102. https://doi.org/10.1002/14651858.CD013102	Quantitative focus
Dobremez V, Ruel JH, Hugon A, Gay S, Berlioz J, Pineau-Blondel E. Contraindicated drugs in myasthenia gravis patients: Elaboration of a tool to optimize drug therapy. *International Journal of Clinical Pharmacy* 2015;**37**:236-7. https://doi.org/10.1007/s11096-014-0039-2	Abstract
Dunn BL, Teusink AC, Taber DJ, Hemstreet BA, Uber LA, Weimert NA. Management of hypertension in renal transplant patients: a comprehensive review of nonpharmacologic and pharmacologic treatment strategies. *Ann Pharmacother* 2010;**44**:1259-70. https://doi.org/10.1345/aph.1P004	Not SR
Elley CR, Gupta AK, Webster R, Selak V, Jun M, Patel A*, et al.* The efficacy and tolerability of 'polypills': meta-analysis of randomised controlled trials. *PLoS One* 2012;**7**:e52145. https://doi.org/10.1371/journal.pone.0052145	Quantitative focus
Elnaem MH, Rosley NFF, Alhifany AA, Elrggal ME, Cheema E. Impact of Pharmacist-Led Interventions on Medication Adherence and Clinical Outcomes in Patients with Hypertension and Hyperlipidemia: A Scoping Review of Published Literature. *J Multidiscip Healthc* 2020;**13**:635-45. https://doi.org/10.2147/JMDH.S257273	Not SR
Engel J, Damen NL, van der Wulp I, de Bruijne MC, Wagner C. Adherence to Cardiac Practice Guidelines in the Management of Non-ST-Elevation Acute Coronary Syndromes: A Systematic Literature Review. *Curr Cardiol Rev* 2017;**13**:3-27. https://doi.org/10.2174/1573403x12666160504100025	Quantitative focus
Gale N, Marshall T, Bramley G. Starting and staying on preventive medication for cardiovascular disease. *Curr Opin Cardiol* 2012;**27**:533-41. https://doi.org/10.1097/HCO.0b013e328356dae5	Not SR
Gandapur Y, Kianoush S, Kelli HM, Misra S, Urrea B, Blaha MJ*, et al.* The role of mHealth for improving medication adherence in patients with cardiovascular disease: a systematic review. *Eur Heart J Qual Care Clin Outcomes* 2016;**2**:237-44. https://doi.org/10.1093/ehjqcco/qcw018	Not SR
Gellad WF, Grenard JL, Marcum ZA. A systematic review of barriers to medication adherence in the elderly: looking beyond cost and regimen complexity. *Am J Geriatr Pharmacother* 2011;**9**:11-23. https://doi.org/10.1016/j.amjopharm.2011.02.004	Quantitative focus
Georgiopoulos G, Kollia Z, Katsi V, Oikonomou D, Tsioufis C, Tousoulis D. Nurse's Contribution to Alleviate Non-adherence to Hypertension Treatment. *Curr Hypertens Rep* 2018;**20**:65. https://doi.org/10.1007/s11906-018-0862-2	Not SR

For peer review only - http://bmjopen.bmj.com/site/about/guidelines.xhtml

Glynn L, Fahey T. Cardiovascular medication: improving adherence. *BMJ Clin Evid* 2011;**2011**.	Not SR
Golubev S. Compliance measurement-guided medication management programs in hypertension: A health technology assessment. *Journal of Hypertension* 2010;**28**:e269.	Quantitative focus
Golubev S, Fedoseyeva I, Johri M. Compliance measurement-guided medication management programs in hypertension: A systematic review. *Basic and Clinical Pharmacology and Toxicology* 2011;**109**:109-10. http://dx.doi.org/10.1111/j.1742-7843.2011.00722.x	Abstract
Gwadry-Sridhar FH, Manias E, Lal L, Salas M, Hughes DA, Ratzki-Leewing A*, et al.* Impact of interventions on medication adherence and blood pressure control in patients with essential hypertension: a systematic review by the ISPOR medication adherence and persistence special interest group. *Value Health* 2013;**16**:863-71. https://doi.org/10.1016/j.jval.2013.03.1631	Not SR
Hopper I, Samuel R, Tonkin A, Krum H. The effects of discontinuing medications in stable systolic heart failure-systematic review and meta-analysis. *Heart Lung and Circulation* 2012;**21**:S95. http://dx.doi.org/10.1016/j.hlc.2012.05.241	Abstract
Imbeault B, Vallee M. Single-Pill Combinations in the Treatment of Hypertension in Adults: Beyond Convenience. *Can J Diabetes* 2018;**42**:205-8. https://doi.org/10.1016/j.jcjd.2018.01.011	Not SR
Iwelunmor J, Airhihenbuwa CO, Cooper R, Tayo B, Plange-Rhule J, Adanu R*, et al.* Prevalence, determinants and systems-thinking approaches to optimal hypertension control in West Africa. *Global Health* 2014;**10**:42. https://doi.org/10.1186/1744-8603-10-42	Not SR
Jamshidnezhad A, Kabootarizadeh L, Hoseini SM. The Effects of Smartphone Applications on Patients Self-care with Hypertension: A Systematic Review Study. *Acta Inform Med* 2019;**27**:263-7. https://doi.org/10.5455/aim.2019.27.263-267	Not SR
Jones LK, Gionfriddo MR, Tilberry S, Frisbie L, Gregor C, Gidding S*, et al.* Implementation strategies to improve statin utilization in individuals with hypercholesterolemia: A systematic review. *JACCP Journal of the American College of Clinical Pharmacy* 2020;**3**:365. http://dx.doi.org/10.1002/jac5.1204	Abstract
Jornten-Karlsson M, Pintat S, Molloy-Bland M, Berg S, Ahlqvist M. Patient-Centered Interventions to Improve Adherence to Statins: A Narrative Synthesis of Systematically Identified Studies. *Drugs* 2016;**76**:1447-65. https://doi.org/10.1007/s40265-016-0640-x	Not SR
Ju A, Hanson CS, Banks E, Korda R, Craig JC, Usherwood T*, et al.* Patient beliefs and attitudes to taking statins: systematic review of qualitative studies. *Br J Gen Pract* 2018;**68**:e408-e19. https://doi.org/10.3399/bjgp18X696365	Not SR

For peer review only

For peer review only - http://bmjopen.bmj.com/site/about/guidelines.xhtml

Ko DT, Jackevicius CA. Stopping beta-Blockers After Myocardial Infarction: Not So Fast! *Circ Cardiovasc Qual Outcomes* 2018;**11**:e004678. https://doi.org/10.1161/CIRCOUTCOMES.118.004678	Not SR	
Krack G. EXPLANATIONS FOR THE DIFFERENCES BETWEEN EFFECTS ON ADHERENCE OF VALUE-BASED HEALTH INSURANCE DESIGNS: A SYSTEMATIC REVIEW AND META-ANALYSIS. *Value in Health* 2018;**21**:S155. http://dx.doi.org/10.1016/j.jval.2018.09.924	Abstract	
Krueger K, Griese-Mammen N, Schubert I, Kieble M, Botermann L, Laufs U*, et al.* In search of a standard when analyzing medication adherence in patients with heart failure using claims data: a systematic review. *Heart Fail Rev* 2018;**23**:63-71. https://doi.org/10.1007/s10741-017-9656-x	Quantitative focus	
Kwon A, Kim GH. Single-pill Combination Therapy of Azilsartan Medoxomil/Chlorthalidone for Treatment of Hypertension: A Systematic Review. *Clin Ther* 2020;**42**:1390-403. https://doi.org/10.1016/j.clinthera.2020.05.015	Not SR (Duplicate excluded in update search)	
Lane D, Patel P, Khunti K, Gupta P. Objective measures of non-adherence in cardiometabolic diseases: a review focused on urine biochemical screening. *Patient Prefer Adherence* 2019;**13**:537-47. https://doi.org/10.2147/PPA.S162215	Not SR	
Lee JL, Maciejewski M, Raju S, Shrank WH, Choudhry NK. Value-based insurance design: quality improvement but no cost savings. *Health Aff (Millwood)* 2013;**32**:1251-7. https://doi.org/10.1377/hlthaff.2012.0902	Not SR	
Lennon O, Blake C, Booth J, Pollock A, Lawrence M. Interventions for behaviour change and self-management in stroke secondary prevention: protocol for an overview of reviews. *Syst Rev* 2018;**7**:231. https://doi.org/10.1186/s13643-018-0888-1	Protocol	
Liu HL, Chen XY, Li JR, Su SW, Ding T, Shi CX*, et al.* Efficacy and Safety of Pulmonary Arterial Hypertension-specific Therapy in Pulmonary Arterial Hypertension: A Meta-analysis of Randomized Controlled Trials. *Chest* 2016;**150**:353-66. https://doi.org/10.1016/j.chest.2016.03.031	Quantitative focus	
Logue J, Al-Ghibiwi H, Alamri AA, Preiss D. Systematic review of studies exploring reasons for statin non-adherence and of randomised controlled trials of interventions to improve adherence. *Atherosclerosis* 2015;**241**:e52.	Abstract	
Madhavan MV, Gersh BJ, Alexander KP, Granger CB, Stone GW. Coronary Artery Disease in Patients >/=80 Years of Age. *J Am Coll Cardiol* 2018;**71**:2015-40. https://doi.org/10.1016/j.jacc.2017.12.068	Not SR	
Marcum ZA, Handler SM, Boyce R, Gellad W, Hanlon JT. Medication misadventures in the elderly: a year in review. *Am J Geriatr Pharmacother* 2010;**8**:77-83. https://doi.org/10.1016/j.amjopharm.2010.02.002	Not SR	

For peer review only - http://bmjopen.bmj.com/site/about/guidelines.xhtml

Matthes J, Albus C. Improving adherence with medication: a selective literature review based on the example of hypertension treatment. *Dtsch Arztebl Int* 2014;**111**:41-7. https://doi.org/10.3238/arztebl.2014.0041	Not SR	
Mendes Soares M, Oliveira LeÆo e Silva L, Alberto Dias C, Maria Rodrigues S, Jorge Machado C. ADESAO DO IDOSO AO TRATAMENTO DA HIPERTENSAO ARTERIAL SISTEMICA: REVISAO INTEGRATIVA. *Cogitare Enfermagem* 2012;**17**:114-50.	Non-English language	
Molloy GJ, O'Carroll RE, Witham MD, McMurdo ME. Interventions to enhance adherence to medications in patients with heart failure: a systematic review. *Circ Heart Fail* 2012;**5**:126-33. https://doi.org/10.1161/CIRCHEARTFAILURE.111.964569	Not SR	
Morgado M, Castanheira L, Verde I, Castelo-Branco M. Pharmacist interventions to enhance patient adherence to antihypertensive medication: A systematic review. *Journal of Clinical Hypertension* 2010;**12**:A112-A3. http://dx.doi.org/10.1111/j.1751-7176.2010.00282.x	Abstract	
Morgado MP, Morgado SR, Mendes LC, Pereira LJ, Castelo-Branco M. Pharmacist interventions to enhance blood pressure control and adherence to antihypertensive therapy: Review and meta-analysis. *Am J Health Syst Pharm* 2011;**68**:241-53. https://doi.org/10.2146/ajhp090656	Not SR	
Morrissey EC, Durand H, Nieuwlaat R, Navarro T, Haynes RB, Walsh JC*, et al.* Effectiveness and content analysis of interventions to enhance medication adherence in hypertension: a systematic review and meta-analysis protocol. *Syst Rev* 2016;**5**:96. https://doi.org/10.1186/s13643-016-0278-5	Protocol	
Na. Selected Abstracts from the National Congress of the Italian Society of Hypertension (SIIA 2010). *High Blood Pressure & Cardiovascular Prevention* 2010;**17**:131-90. https://doi.org/10.2165/11311900-000000000-00000	Quantitative focus	
Naderi SH, Bestwick JP, Wald DS. Adherence to drugs that prevent cardiovascular disease: meta-analysis on 376,162 patients. *Am J Med* 2012;**125**:882-7 e1. https://doi.org/10.1016/j.amjmed.2011.12.013	Not SR	
Nguyen E, Sobieraj DM. The impact of appointment-based medication synchronization on medication taking behaviour and health outcomes: A systematic review. *J Clin Pharm Ther* 2017;**42**:404-13. https://doi.org/10.1111/jcpt.12554	Not SR	
Nguyen T, Gurwitz J. Paper Abstract. *Journal of the American Geriatrics Society* 2016;**64**:S1-S311. https://doi.org/10.1111/jgs.14231	Quantitative focus	
Ni Z, Dardas L, Wu B, Shaw R. Cardioprotective medication adherence among patients with coronary heart disease in China: a systematic review. *Heart Asia* 2019;**11**:e011173. https://doi.org/10.1136/heartasia-2018-011173	Not SR	

For peer review only

For peer review only - http://bmjopen.bmj.com/site/about/guidelines.xhtml

Nielsen JO, Shrestha AD, Neupane D, Kallestrup P. Non-adherence to anti-hypertensive medication in low- and middle-income countries: a systematic review and meta-analysis of 92443 subjects. *J Hum Hypertens* 2017;**31**:14-21. https://doi.org/10.1038/jhh.2016.31	Quantitative focus	
Nieuwlaat R, Connolly SJ, MacKay JA, Weise-Kelly L, Navarro T, Wilczynski NL*, et al.* Computerized clinical decision support systems for therapeutic drug monitoring and dosing: A decision maker-researcher partnership systematic review. *Circulation: Cardiovascular Quality and Outcomes* 2011;**4**.	Drug not relevant	
Njie GJ, Mukhtar Q, Finnie RKC, Dhakal-Acharya S, Thota AB, Hopkins DP*, et al.* Effectiveness of reducing out-of-pocket costs for medications to treat hypertension and hyperlipidemia: A community guide systematic review. *Journal of the American Society of Hypertension* 2014;**8**:e124-e5. http://dx.doi.org/10.1016/j.jash.2014.03.285	Abstract	
Ofori-Asenso R, Zoungas S, Liew D. Reinitiation of Statin Therapy After Discontinuation: A Meta-analysis. *Mayo Clin Proc* 2018;**93**:666-8. https://doi.org/10.1016/j.mayocp.2018.01.011	Quantitative focus	
Oosterom-Calo R, van Ballegooijen AJ, Terwee CB, te Velde SJ, Brouwer IA, Jaarsma T*, et al.* Determinants of adherence to heart failure medication: a systematic literature review. *Heart Fail Rev* 2013;**18**:409-27. https://doi.org/10.1007/s10741-012-9321-3	Quantitative focus	
Osborn D, Burton A, Walters K, Atkins L, Barnes T, Blackburn R*, et al. Primary care management of cardiovascular risk for people with severe mental illnesses: the Primrose research programme including cluster RCT*. Southampton (UK): NIHR Journals Library; 2019 Apr. http://doi.org/ 10.3310/pgfar07020	Quantitative focus	
Perera R, McFadden E, McLellan J, Lung T, Clarke P, Perez T*, et al.* Optimal strategies for monitoring lipid levels in patients at risk or with cardiovascular disease: a systematic review with statistical and cost-effectiveness modelling. *Health Technol Assess* 2015;**19**:1-401, vii-viii. https://doi.org/10.3310/hta191000	Quantitative focus	
Phan K, Gomez YH, Elbaz L, Daskalopoulou SS. Statin treatment non-adherence and discontinuation: clinical implications and potential solutions. *Curr Pharm Des* 2014;**20**:6314-24. https://doi.org/10.2174/1381612820666140620162629	Quantitative focus	
Pinho S, Cruz M, Ferreira F, Ramalho A, Sampaio R. Improving medication adherence in hypertensive patients: A scoping review. Preventive medicine 2021;146:106467. https://doi.org/https://dx.doi.org/10.1016/j.ypmed.2021.106467	Not SR	
Polinski JM, Donohue JM, Kilabuk E, Shrank WH. Medicare Part D's effect on the under- and overuse of medications: a systematic review. *J Am Geriatr Soc* 2011;**59**:1922-33. https://doi.org/10.1111/j.1532-5415.2011.03537.x	Quantitative focus	

For peer review only - http://bmjopen.bmj.com/site/about/guidelines.xhtml

Powers BJ, Coeytaux RR, Dolor RJ, Hasselblad V, Patel UD, Yancy WS, Jr., *et al.* Updated report on comparative effectiveness of ACE inhibitors, ARBs, and direct renin inhibitors for patients with essential hypertension: much more data, little new information. *J Gen Intern Med* 2012;**27**:716-29. https://doi.org/10.1007/s11606-011-1938-8	Quantitative focus	
Rabien A, Price M. Development of a clinical decision support tool for the primary prevention of cardiovascular disease. *Canadian Family Physician* 2015;**61**:S2.	Abstract	
Rahmawati R, Bajorek BV. Self-medication among people living with hypertension: a review. *Fam Pract* 2017;**34**:147-53. https://doi.org/10.1093/fampra/cmw137	Not SR	
Reeve E, Jordan V, Thompson W, Sawan M, Todd A, Gammie TM, *et al.* Withdrawal of antihypertensive drugs in older people. *Cochrane Database Syst Rev* 2020;**6**:CD012572. https://doi.org/10.1002/14651858.CD012572.pub2	Quantitative focus	
Ruppar TM, Dunbar-Jacob JM, Mehr DR, Lewis L, Conn VS. Medication adherence interventions among hypertensive black adults: a systematic review and meta-analysis. *J Hypertens* 2017;**35**:1145-54. https://doi.org/10.1097/HJH.0000000000001260	Full text not retrieved	
Schedlbauer A, Davies P, Fahey T. Interventions to improve adherence to lipid lowering medication. *Cochrane Database Syst Rev* 2010; 10.1002/14651858.CD004371.pub3:CD004371. https://doi.org/10.1002/14651858.CD004371.pub3	Not latest version	
Scranton RE, Goldstein I, Stecher VJ. Erectile dysfunction diagnosis and treatment as a means to improve medication adherence and optimize comorbidity management. *J Sex Med* 2013;**10**:551-61. https://doi.org/10.1111/j.1743-6109.2012.02998.x	Not SR	
Seedat Y. Fixed drug combination in the treatment of hypertension and hyperlipidemia in the developing world. *Circulation* 2010;**122**:e94. http://dx.doi.org/10.1161/CIRCULATIONAHA.110.192773	Abstract	
Selak V, Webster R, Stepien S, Bullen C, Patel A, Thom S, *et al.* Reaching cardiovascular prevention guideline targets with a polypill-based approach: a meta-analysis of randomised clinical trials. *Heart* 2019;**105**:42-8. https://doi.org/10.1136/heartjnl-2018-313108	Not SR	
Shahaj O, Denneny D, Schwappach A, Pearce G, Epiphaniou E, Parke HL, *et al.* Supporting self-management for people with hypertension: a meta-review of quantitative and qualitative systematic reviews. *J Hypertens* 2019;**37**:264-79. https://doi.org/10.1097/HJH.0000000000001867	Full text not retrieved	

For peer review only - http://bmjopen.bmj.com/site/about/guidelines.xhtml

Shaman AM, Smyth B, Arnott C, Palmer SC, Mihailidou AS, Jardine MJ*, et al.* Comparative Efficacy and Safety of BP-Lowering Pharmacotherapy in Patients Undergoing Maintenance Dialysis: A Network Meta-Analysis of Randomized, Controlled Trials. *Clin J Am Soc Nephrol* 2020;**15**:1129-38. https://doi.org/10.2215/CJN.12201019	Full text not retrieved	
Sherrill B, Halpern M, Khan S, Zhang J, Panjabi S. Single-pill vs free-equivalent combination therapies for hypertension: a meta-analysis of health care costs and adherence. *J Clin Hypertens (Greenwich)* 2011;**13**:898-909. https://doi.org/10.1111/j.1751-7176.2011.00550.x	Not SR	
Sommerauer C, Kaushik N, Woodham A, Renom-Guiteras A, Martinez YV, Reeves D*, et al.* Thiazides in the management of hypertension in older adults - a systematic review. *BMC Geriatr* 2017;**17**:228. https://doi.org/10.1186/s12877-017-0576-3	Quantitative focus	
Sparrow RT, Khan AM, Ferreira-Legere LE, Ko DT, Jackevicius CA, Goodman SG*, et al.* Effectiveness of Interventions Aimed at Increasing Statin-Prescribing Rates in Primary Cardiovascular Disease Prevention: A Systematic Review of Randomized Clinical Trials. *JAMA Cardiol* 2019;**4**:1160-9. https://doi.org/10.1001/jamacardio.2019.3066	Full text not retrieved	
Stergiou GS, Bliziotis IA. Home blood pressure monitoring in the diagnosis and treatment of hypertension: a systematic review. *Am J Hypertens* 2011;**24**:123-34. https://doi.org/10.1038/ajh.2010.194	Not SR	
Sua YS, Jiang Y, Thompson DR, Wang W. Effectiveness of mobile phone-based self-management interventions for medication adherence and change in blood pressure in patients with coronary heart disease: A systematic review and meta-analysis. European journal of cardiovascular nursing 2020;19:192-200. https://doi.org/https://dx.doi.org/10.1177/1474515119895678	Medication NR	
Thio SL, Nam J, van Driel ML, Dirven T, Blom JW. Effects of discontinuation of chronic medication in primary care: a systematic review of deprescribing trials. *Br J Gen Pract* 2018;**68**:e663-e72. https://doi.org/10.3399/bjgp18X699041	Quantitative focus	
Thomopoulos C, Parati G, Zanchetti A. Effects of blood pressure lowering treatment in hypertension: 8. Outcome reductions vs. discontinuations because of adverse drug events - meta-analyses of randomized trials. *J Hypertens* 2016;**34**:1451-63. https://doi.org/10.1097/HJH.0000000000000972	Full text not retrieved	
Thomopoulos C, Parati G, Zanchetti A. Effects of blood-pressure-lowering treatment in hypertension: 9. Discontinuations for adverse events attributed to different classes of antihypertensive drugs: meta-analyses of randomized trials. *J Hypertens* 2016;**34**:1921-32. https://doi.org/10.1097/HJH.0000000000001052	Full text not retrieved	

For peer review only - http://bmjopen.bmj.com/site/about/guidelines.xhtml

Vassy JL, Chun S, Advani S, Ludin SA, Smith JG, Alligood EC. Impact of SLCO1B1 Pharmacogenetic Testing on Patient and Healthcare Outcomes: A Systematic Review. *Clin Pharmacol Ther* 2019;**106**:360-73. https://doi.org/10.1002/cpt.1223	Not SR	
Vogele A, Johansson T, Renom-Guiteras A, Reeves D, Rieckert A, Schlender L*, et al.* Effectiveness and safety of beta blockers in the management of hypertension in older adults: a systematic review to help reduce inappropriate prescribing. *BMC Geriatr* 2017;**17**:224. https://doi.org/10.1186/s12877-017-0575-4	Quantitative focus	
Webster R, Patel A, Selak V, Billot L, Bots ML, Brown A*, et al.* Effectiveness of fixed dose combination medication ('polypills') compared with usual care in patients with cardiovascular disease or at high risk: A prospective, individual patient data meta-analysis of 3140 patients in six countries. *Int J Cardiol* 2016;**205**:147-56. https://doi.org/10.1016/j.ijcard.2015.12.015	Not SR	
Weeda ER, Coleman CI, McHorney CA, Crivera C, Schein JR, Sobieraj DM. Impact of once- or twice-daily dosing frequency on adherence to chronic cardiovascular disease medications: A meta-regression analysis. *Int J Cardiol* 2016;**216**:104-9. https://doi.org/10.1016/j.ijcard.2016.04.082	Not SR	
Weernink MG, Vaanholt MC, Groothuis-Oudshoorn CG, Von Birgelen C, M.J IJ, Van Til JA. Patients' priorities for oral anticoagulation therapy in non-valvular atrial fibrillation. *Value in Health* 2017;**20**:A621. http://dx.doi.org/10.1016/j.jval.2017.08.1355	Abstract	
Weisser B, Predel HG, Gillessen A, Hacke C, Vor dem Esche J, Rippin G*, et al.* Single Pill Regimen Leads to Better Adherence and Clinical Outcome in Daily Practice in Patients Suffering from Hypertension and/or Dyslipidemia: Results of a Meta-Analysis. *High Blood Press Cardiovasc Prev* 2020;**27**:157-64. https://doi.org/10.1007/s40292-020-00370-5	Not SR (Duplicate excluded in update search)	
Wettermark B, Qvarnstrom M, Hasselstrom J, Kahan T. Controversies on patient persistence in antihypertensive drug treatment: A systematic review. *JRSM Cardiovascular Disease* 2013; http://dx.doi.org/10.1177/2048004013506452:6-7. http://dx.doi.org/10.1177/2048004013506452	Abstract	
Yan YL, Qiu B, Hu LJ, Jing XD, Liu YJ, Deng SB*, et al.* Efficacy and safety evaluation of intensive statin therapy in older patients with coronary heart disease: a systematic review and meta-analysis. *Eur J Clin Pharmacol* 2013;**69**:2001-9. https://doi.org/10.1007/s00228-013-1570-0	Quantitative focus	
Zhao P, Xu P, Wan C, Wang Z. Evening versus morning dosing regimen drug therapy for hypertension. *Cochrane Database Syst Rev* 2011; 10.1002/14651858.CD004184.pub2:CD004184. https://doi.org/10.1002/14651858.CD004184.pub2	Quantitative focus	

For peer review only - http://bmjopen.bmj.com/site/about/guidelines.xhtml

Zharkova T, Kyomen H. Management Strategies to Prevent Stroke in Elderly with Major Neurocognitive Disorder and Psychosis Treated with Antipsychotic Medication. *The American Journal of Geriatric Psychiatry* 2019;**27**:S135-S6. https://doi.org/10.1016/j.jagp.2019.01.042	Not SR	

For peer review only

For peer review only - http://bmjopen.bmj.com/site/about/guidelines.xhtml

APPENDIX D: CHARACTERISTICS OF INCLUDED REVIEWS

Key for interpreting tables

Green highlighted text:	High overall quality as appraised by AMSTAR-2
Turquoise highlighted text:	Medium overall quality as appraised by AMSTAR-2
Orange highlighted text:	Low overall quality as appraised by AMSTAR-2
Red highlighted text:	Critically-low quality as appraised by the AMSTAR-2
CEESAT Set 1:	Prioritised for full quality appraisal using AMSTAR-2
CEESAT Set 2:	Scored poorly on 1 out of 4 items on CEESAT
CEESAT Set 3:	Scored poorly on 2-4 items on CEESAT

Reviews were judged to be of ‘High’ relevance if review focused on evaluating intervention/gathering experiences regarding medication of interest within health/social care services relevant to UK service setting, ‘Medium’ relevance: if review aim/inclusion criteria somewhat relevant to aim of systematic mapping review, but also includes other medications not of interest, or quantity of relevant information is limited, ‘Low’ relevance: if quantity of relevant information low and/or intervention being evaluated not relevant to UK health/social care setting

For peer review only - http://bmjopen.bmj.com/site/about/guidelines.xhtml

Table 3: Review overview

Focus/Aim	Study (First author, date)	Type of Review (design of studies included), synthesis methods	Eligible age of participants within studies included in the review	Medications of interest	Medical condition for which medication of interest being taken	Relevance of systematic review to aim of evidence-gap map	CEESAT set	Other comments
Evaluating intervention: enhance adherence	Adler, 2017	SR (Quantitative: RCTs), Narrative	NR<br>*Mean age where reported in included studies: 53.6 years to 64 years*	Statins<br>Antihypertensives	Secondary prevention CVD	Medium	1	
Evaluating intervention: enhance adherence	Al Alshaikh, 2016	SR(Quantitative: RCTs), MA+Narrative	18 years or over	Statins<br>Antihypertensives	Secondary prevention CVD	Medium	3	
Evaluating intervention: enhance adherence	Baumgartner, 2020	SR (Quantitative: RCTs, retrospective/prospective cohort, non-randomized, economic models, systematic reviews, meta-analysis), Narrative	NR<br>*Where reported, participants in included studies 18 years of age or over. Some studies specifically included adults 65 years or older*	Statins<br>Antihypertensives	HBP<br>Primary prevention CVD<br>Secondary prevention CVD	Medium	3	
Evaluating intervention: enhance adherence	Bochkareva, 2019	Scoping review (Quantitative: RCTs, non-RCTs, (cross-sectional, cohort studies), registers), Narrative Matrix/map of studies	18 years or over	Antihypertensives	HBP	Medium	3	Focus on Russia. Boad focus of scoping review, of which interventions to increase adherence are one part
Evaluating intervention: enhance adherence	Checchi, 2014	SR (Quantitative: RCTs, nonRCT, controlled, prospective/retrospective, observational, cohort), Narrative	NR<br>*Adults*	Antihypertensives<br>Statins	HBP<br>Primary prevention CVD	Medium	3	

For peer review only - http://bmjopen.bmj.com/site/about/guidelines.xhtml

Focus/Aim	Study (First author, date)	Type of Review (design of studies included), synthesis methods	Eligible age of participants within studies included in the review	Medications of interest	Medical condition for which medication of interest being taken	Relevance of systematic review to aim of evidence-gap map	CEESAT set	Other comments
Evaluating intervention: enhance adherence Secondary aim	Cheema, 2014	SR (Quantitative: RCTs, systematic reviews of RCTs), Meta-analysis+Narrative	18 years or over	Antihypertensives	HBP	High	2	
Evaluating intervention: optimise prescribing	Chhina, 2013	SR (Quantitative: RCT, observational studies with a control group), Narrative	NR *1 included study focused on children, 3 focused on elderly*	Antihypertensives	HBP	Medium	3	One included study focused on children
Evaluating intervention: enhance adherence	Conn, 2015[70]	SR (Quantitative: NR), Meta-analysis	NR *Median value for mean age accross included studies was 54.4 years*	Statins Antihypertensives	HBP Secondary prevention CVD	Medium	2	Medications of interest inferred from population disease characteristics
Evaluating intervention: enhance adherence	Conn, 2015[71]	SR (Quantitative: treatment-versus-control comparisons, pre-experimental studies), Exploratory analyses, Meta-analysis	NR *Median mean participant age accross included studies was 60.70 years*	Statins Antihypertensives	HBP Primary prevention CVD Secondary prevention CVD Inferred from population disease characteristics	Medium	2	Medications of interest inferred from population disease characteristics
Evaluating intervention: enhance adherence	Conn, 2015[36]	SR (Quantitative: varied research methods were included), Meta-analysis	Other *Adults*	Antihypertensives	HBP	High	3	

For peer review only - http://bmjopen.bmj.com/site/about/guidelines.xhtml

Focus/Aim	Study (First author, date)	Type of Review (design of studies included), synthesis methods	Eligible age of participants within studies included in the review	Medications of interest	Medical condition for which medication of interest being taken	Relevance of systematic review to aim of evidence-gap map	CEESAT set	Other comments
Evaluating intervention: enhance adherence	Conn, 2016	SR (Quantitative: RCTs, non-RCTs, Pre-experimental before and after), Narrative+Meta-analysis	NR<br>*Adults. Median mean age accross included studies 53 years*	Statins<br>Antihypertensives	Primary prevention CVD<br>Depression<br>HBP<br>Secondary prevention CVD	Medium	2	Medications of interest inferred from population disease characteristic
Evaluating intervention: enhance adherence secondary aim	de Cates, 2014	SR (Quantitative: RCTs), Meta-analysis	18 years or over	Statins<br>Antihypertensives	Primary prevention CVD<br>Secondary prevention CVD	Medium	1	Adherence only secondary outcome
Evaluating intervention: enhance adherence	De Simoni, 2013	Quantitative (RCTs), Narrative+Meta-analysis	Other<br>*Adults*	Antihypertensives	HBP<br>Secondary prevention CVD	High	2	
Evaluating intervention: enhance adherence	Deichmann. 2016	SR (Quantitative: RCTs, parallel group or crossover, individual or cluster randomization), Meta-analysis+Narrative	18 years or over	Statins	Primary prevention CVD<br>Secondary prevention CVD	Medium	3	

For peer review only - http://bmjopen.bmj.com/site/about/guidelines.xhtml

Focus/Aim	Study (First author, date)	Type of Review (design of studies included), synthesis methods	Eligible age of participants within studies included in the review	Medications of interest	Medical condition for which medication of interest being taken	Relevance of systematic review to aim of evidence-gap map	CEESAT set	Other comments
Evaluating intervention: enhance adherence	Demonceau, 2013	SR (Quantitative: RCT, cross-over and cluster-randomized), Meta-analysis+meta-regression	Other<br>*Average age expressed in years was 47.5 [range (3.0; 73.7)] in the usual care group and 46.8 [range (3.4; 76.2)] in the intervention group*<br>*Adults and children*	Antihypertensives<br>*Assumed*	HBP<br>NR	Medium	3	Included methadone clinic patients (n=1). Included children
Patient views: adherence to medication of interest<br>Only these of interest	Dhar, 2017	SR (Mixed: only quali studies met focus of review. Quantitative, descriptive-co relational study, cross-sectional, focus group interviews, survey, interviews), Narrative	18 years or over	Antihypertensives	HBP	Low	3	Focus on undeveloped countries. Only 5 included qualitative studies
Evaluating intervention: deprescribing	Dills, 2018	SR (Quantitative: Dills-2019-Deprescribing Medications for Chronic.pdf: Page 1: "Randomized Controlled Trials" Narrative	18 years or over	Statins<br>Antihypertensives	HBP<br>Secondary prevention CVD<br>Other<br>Illnesses associated with use of: alendronate, PPI, H2 antagonist	Medium	1	Palliative care studies where life expectancy is > 6 months included

For peer review only - http://bmjopen.bmj.com/site/about/guidelines.xhtml

Focus/Aim	Study (First author, date)	Type of Review (design of studies included), synthesis methods	Eligible age of participants within studies included in the review	Medications of interest	Medical condition for which medication of interest being taken	Relevance of systematic review to aim of evidence-gap map	CEESAT set	Other comments
Evaluating intervention: enhance adherence	Du, 2018	SR (Quantitative: parallel comparison studies, retrospective cohort, prospective), Meta-analysis	NR *Mean age of included studies (where reported) ranged from 53-76 years*	Antihypertensives	HBP	High	3	
Evaluating intervention: enhance adherence	Fletcher, 2015	SR (Quantitative: Randomized and quasi-randomized trials), Narrative+Meta-analysis	NR *Where reported, participants mean age ranged from 38 years to 77 accross included studies*	Antihypertensives	HBP	High	1	
Evaluating intervention: enhance adherence	Gupta, 2010	SR (Quantitative: RCT, clinical trial and prospective or retrospective cohort), Narrative+Meta-analysis	Other *18 to 79 years*	Antihypertensives	HBP	High	3	
Evaluating intervention: enhance adherence	Guzman-Tordecilla, 2020	SR (Quantitative: Guzman-Tordecilla-2020-InterventionsToIncreaseThePhar.pdf: Page 5: "Quasi-experimental" "Experimental" Narrative	17 years or over	Antihypertensives	HBP	Low	3	Studies conductedi n Latin America

For peer review only - http://bmjopen.bmj.com/site/about/guidelines.xhtml

Focus/Aim	Study (First author, date)	Type of Review (design of studies included), synthesis methods	Eligible age of participants within studies included in the review	Medications of interest	Medical condition for which medication of interest being taken	Relevance of systematic review to aim of evidence-gap map	CEESAT set	Other comments
Evaluating intervention: deprescribing Secondary aim, not actually explicit. Look at interventions to reduce Fall Risk-Inducing Drugs (FRID) in an attempt to understand whether this reduced falls.	Hart, 2020	SR (Quantitative: observational or intervention (eg, RCTs and quasi-experimental design)), Narrative	Other *60 or over*	Antihypertensives	NR	Medium	3	Lots of other drugs. Also approach reduction of drugs of interest in a round-about way, rather than directly wanting to reduce them
Evaluating intervention: optimise prescribing	Hukins, 2019	SR (Quantitative: observational, non-randomised evaluation), Narrative	Other *People with a diagnosis of/suspected dementia*	Statins	NR	Medium	1	
Patient views: adherence to medication of interest Patient views: intervention to enhance adherence	Ingersgaard, 2020	Review of SR (Other, all types of systematic reviews), Narrative	NR	Statins Antihypertensives	Primary prevention CVD Secondary prevention CVD	Medium	2	Review of reviews, little relevant qualitative evidence
Evaluating intervention: optimise prescribing	Jeffery, 2015	SR (Quantitative: RCTs, cluster and nexted), Meta-analysis+Narrative	NA *Targeted prescribers*	Statins Antihypertensives	Primary prevention CVD Secondary prevention CVD	High	1	
Evaluating intervention: optimise prescribing	Kang, 2016	SR (Quantitative: RCTs, non-RCT, prospective observational studies), Narrative+Meta-analysis	18 years or over	Statins Antihypertensives	Secondary prevention CVD	High	3	

For peer review only - http://bmjopen.bmj.com/site/about/guidelines.xhtml

Focus/Aim	Study (First author, date)	Type of Review (design of studies included), synthesis methods	Eligible age of participants within studies included in the review	Medications of interest	Medical condition for which medication of interest being taken	Relevance of systematic review to aim of evidence-gap map	CEESAT set	Other comments
Evaluating intervention: enhance adherence	Kassavou, 2018	SR (Quantitative: RCTs, randomisation at individual patient or cluster level), Meta-analysis+meta-regression	18 years or over	Statins<br>Antihypertensives	HBP<br>Primary prevention CVD<br>Secondary prevention CVD<br>Other Cardio-metabolic conditions, high cholesterol	High	2	
Evaluating intervention: enhance adherence	Kawalec, 2018	SR (Quantitative: No restrictions were applied as to the study design. SRs, cohort, non-RCT, prospective observational study), Narrative+Meta-analysis	NR<br>*Where reported, all included studies included participants of 18 years or older*	Antihypertensives	HBP	High	3	
Practitioner views: prescribing medication of interest<br>Patient views: adherence to medication of interest	Khatib, 2014	SR (Mixed: Qualitative and quantitative observational studies. Included RCT, cohort, case-control), Framework/Content analysis, Narrative	Other<br>*Any age*	Antihypertensives	HBP	High	2	Methods of qualitative synthesis unclear

For peer review only - http://bmjopen.bmj.com/site/about/guidelines.xhtml

Focus/Aim	Study (First author, date)	Type of Review (design of studies included), synthesis methods	Eligible age of participants within studies included in the review	Medications of interest	Medical condition for which medication of interest being taken	Relevance of systematic review to aim of evidence-gap map	CEESAT set	Other comments
Evaluating intervention: optimise prescribing<br>Think this is most appropriate overall, but could justify selecting more than one?<br>Evaluating intervention: enhance adherence	King, 2018	SR (Quantitative: RCT, observational), Narrative	NR	Statins<br>Antihypertensives	HBP<br>Primary prevention CVD	Medium	2	
Patient views: adherence to medication of interest<br>Family/carer views: adherence to medication of interest	Kinnear, 2019	SR (Qualitative: face to face/telephone/online interviews, focus groups) Thematic	NR<br>*264 individuals with FH and 13 family members were involved, aged 8-69 years*	Statins	Primary prevention CVD<br>Secondary prevention CVD	Medium	1	N=7 papers reported findings from samples including individuals under 18 years. N=4 papers reported parental views of having children with FH
Evaluating intervention: enhance adherence	Krack, 2019	SR (Quantitative: RCTs, non-RCTs), Narrative+Meta-analysis	NR	Statins<br>Antihypertensives	HBP<br>Primary prevention CVD<br>Secondary prevention CVD	Low	2	
Evaluating intervention: enhance adherence	Kronish, 2011	SR (Quantitative: observational cohorts), Meta-analysis	18 years or over	Antihypertensives	HBP	High	2	

For peer review only - http://bmjopen.bmj.com/site/about/guidelines.xhtml

Focus/Aim	Study (First author, date)	Type of Review (design of studies included), synthesis methods	Eligible age of participants within studies included in the review	Medications of interest	Medical condition for which medication of interest being taken	Relevance of systematic review to aim of evidence-gap map	CEESAT set	Other comments
Evaluating intervention: enhance adherence<br>Evaluating intervention: optimise prescribing<br>Because of interventions directed at HCPs	Laba, 2013	SR (Quantitative: Randomised/quasi-randomised controlled trials), Narrative	Other<br>*Adults*	Statins<br>Antihypertensives	HBP<br>Primary prevention CVD<br>*Possibly secondary prevention, unclear*	Medium	3	
Evaluating intervention: enhance adherence	Lawrence, 2015	SR (Quantitative: RCTs), Meta-analysis	18 years or over	Statins<br>Antihypertensives	Secondary prevention CVD<br>*Prevention of secondary stroke and/or TIA*	Medium	2	Only 3 studies reporting adherence outcomes for each of statins and antihypertensives
Evaluating intervention: optimise prescribing<br>Secondary aim	Legare, 2010	SR (Quantitative: RCTs, well designed quasi-experimental studies (controlled clinical trials, CBA, ITS), Narrative	NR	Statins	NR	Low	1	Possibly only one study (of 5) about statins, the others are not relevant
Evaluating intervention: enhance adherence	Leslie, 2016	SR (Quantitative: RCTs), Narrative	65 years or over<br>*or consisted of Medicare beneficiaries*	Antihypertensives<br>Statins	HBP<br>Primary prevention CVD	Medium	3	Focus on elderly. Medications of interest assumed from population characteristics
Evaluating intervention: optimise prescribing	Loganathan, 2011	SR (Quantitative: RCTs, non-RCTs), Narrative	65 years or over	Antihypertensives	NR	Medium	3	Only a couple of included studies with medications of interest

For peer review only - http://bmjopen.bmj.com/site/about/guidelines.xhtml

Focus/Aim	Study (First author, date)	Type of Review (design of studies included), synthesis methods	Eligible age of participants within studies included in the review	Medications of interest	Medical condition for which medication of interest being taken	Relevance of systematic review to aim of evidence-gap map	CEESAT set	Other comments
Evaluating intervention: enhance adherence Secondary aim	Maimaris, 2013	SR (Quantitative: RCTs, controlled trials, cohort, cross-sectional, case-control, cross-sectional, ecological), Narrative	NR	Antihypertensives	HBP	Medium	1	Limited information relating to adherence of hypertensives. Uncertain as to relevance of findings to UK health and social care setting
Evaluating intervention: enhance adherence Seconardy aim	Mallat, 2016	SR (Quantitative: RCTs), Meta-analysis	18 years or over	Antihypertensives	HBP	High	1	
Evaluating intervention: enhance adherence	Mamudu, 2014	SR (Quantitative: RCTs, non-RCTs), Narrative	NR	Statins Antihypertensives	Primary prevention CVD	Medium	3	
Evaluating intervention: enhance adherence	Mann, 2014	SR (Quantitative: RCTs, ITS, CBA), Narrative	Other *Adult patients*	Statins Antihypertensives	HBP Primary prevention CVD Secondary prevention CVD	Low	2	One included study included participants aged 14 and above. Intervention not relevant to UK setting
Patient views: adherence to medication of interest Qualitative: other Understanding and experiences of hypertension and drug adherence	Marshall, 2012	SR (Qualitative: Interviews, focus groups), Narrative/Thematic	NR *Where reported, age of population within included studies ranged from 18-92 years*	Antihypertensives	HBP	High	3	

For peer review only - http://bmjopen.bmj.com/site/about/guidelines.xhtml

Focus/Aim	Study (First author, date)	Type of Review (design of studies included), synthesis methods	Eligible age of participants within studies included in the review	Medications of interest	Medical condition for which medication of interest being taken	Relevance of systematic review to aim of evidence-gap map	CEESAT set	Other comments
Evaluating intervention: enhance adherence	Milosavljevic, 2018	SR (Quantitative: RCTs, quasi-controlled trials, cluster-controlled trials, BA, retrospective/prospective cohort, observational, single group pre/post comparisons, statistical predictions), Narrative	NR	Antihypertensives	HBP<br>Depression	Medium	2	
Evaluating intervention: enhance adherence	Morrissey, 2017	SR (Quantitative: RCTs), Narrative Meta-analysis	NR<br>*Where reported, mean age ranged from 51 to 76 across included studies*	Antihypertensives	HBP	High	1	
Evaluating intervention: optimise prescribing	Nguyen, 2018	SR (Quantitative: RCTs), Meta-analysis	NR	Statins	Secondary prevention CVD<br>*Ischaemic heart disease*	Medium	2	
Evaluating intervention: enhance adherence	Nieuwlaat, 2014	SR (Quantitative: RCTs), Narrative	NR<br>*Included studies characteristics table indicates both adult and child populations included*	Statins<br>Antihypertensives	HBP<br>Primary prevention CVD<br>Secondary prevention CVD	Medium	1	

For peer review only - http://bmjopen.bmj.com/site/about/guidelines.xhtml

Focus/Aim	Study (First author, date)	Type of Review (design of studies included), synthesis methods	Eligible age of participants within studies included in the review	Medications of interest	Medical condition for which medication of interest being taken	Relevance of systematic review to aim of evidence-gap map	CEESAT set	Other comments
Qualitative: other Factors which predict medication adherence. Not a qualitative review but didn't know what else to select What factors are associated with/influence or predict ad- herence of anti-hypertensive medication in older adults with HBP?, How are their causal relationship and their influence on MA in older adults with HBP based on ecological ap- proach?	Oori, 2019	Integrative review (Mixed: quantitative and qualitative designs, including: cross-sectional and descriptive, interventional, cohort and two descriptive qualitative), Content analysis	Older adults	Antihypertensives	HBP	Low	3	A qualitative synthesis of quant/qual data. Included because of qualitative aspect, but only 'descriptive qualitative' studies were included
Evaluating intervention: deprescribing	Page, 2016	SR (Quantitative: RCTs, quasi-randomized controlled studies, non-RCTs, prospective and retrospective cohort, case-control, single arm, BA), Meta-analysis	65 years or over	Statins Antihypertensives	HBP Primary prevention CVD Secondary prevention CVD	Medium	2	
Evaluating intervention: enhance adherence	Palmer, 2018	SR (Quantitative: RCTs of parallel group design that randomised by participant or by cluster), Narrative	18 years or over	Statins Antihypertensives	Primary prevention CVD	Medium	1	

For peer review only - http://bmjopen.bmj.com/site/about/guidelines.xhtml

Focus/Aim	Study (First author, date)	Type of Review (design of studies included), synthesis methods	Eligible age of participants within studies included in the review	Medications of interest	Medical condition for which medication of interest being taken	Relevance of systematic review to aim of evidence-gap map	CEESAT set	Other comments
Evaluating intervention: optimise prescribing<br>One of a variety of aims	Pantoja, 2019	SR (Quantitative: RCTs, non-RCTs), Narrative	NR	Statins<br>Antihypertensives	HBP<br>Primary prevention<br>CVD *Unclear if statin for primary or secondary prevention*	Medium	1	Some included studies included child participants. Low amount of information about prescribing drugs of interest. intervention is more of a general aid
Evaluating intervention: enhance adherence	Park, 2017	SR (Quantitative: Original research, comparison group, pre/post comparisons, statistical predictions), Narrative	NR	Antihypertensives<br>Statins	HBP<br>Primary prevention<br>CVD<br>NR	Low	3	Not relevant to UK, medications not of interest included
Evaluating intervention: enhance adherence<br>Note that focus is on 'use' which includes initiating prescribing. Drugs of interest may be currently under- or -ver-used	Polinski, 2011	SR (Quantitative: Polinski-2011-Medicare Part D's Effect on the.pdf: Page 3: "Articles were included if they reported original results regarding Part D drug utilization for specific drugs or drug classes, whether drawn from self-report surveys or prescription drug claims"<br>Narrative	NR	Statins<br>Antihypertensives	HBP<br>Primary prevention<br>CVD<br>NR<br>*Not clearly reported*	Low	3	Low relevance to UK. Other medications not of interest included

For peer review only - http://bmjopen.bmj.com/site/about/guidelines.xhtml

Focus/Aim	Study (First author, date)	Type of Review (design of studies included), synthesis methods	Eligible age of participants within studies included in the review	Medications of interest	Medical condition for which medication of interest being taken	Relevance of systematic review to aim of evidence-gap map	CEESAT set	Other comments
Evaluating intervention: enhance adherence Seconary aim: interventions to affect behaviour change that leads to prevention of chronic disease - e.g. taking antihypertensives...	Posadzki, 2016	SR (Quantitative: RCTs, cluster RCTs, quasi-RCTs, ITS, CBA), Narrative+Meta-analysis	NR	Statins Antihypertensives	HBP Primary prevention CVD	Medium	1	Included children. Interventions target general positive behaviour change in terms of health management. However there are specific studies reporting adherence-type interventions. Several non-relevant drugs and outcomes within the review.
Evaluating intervention: enhance adherence	Rash, 2016	SR (Quantitative: RCTs), Narrative	NR	Statins	Primary prevention CVD Secondary prevention CVD	High	2	
Patient views: adherence to medication of interest	Rashid, 2014	SR (Qualitative: Focus groups, interviews, interviews and questionnaires), Meta-ethnography	Other *Adults*	Statins	Secondary prevention CVD	Medium	3	
Qualitative: other Rashid 2018.pdf: Page 1: "explored medication-taking experiences in patients, citizens, carers, relatives and clinicians."	Rashid, 2018	SR (Qualitative: All types of qualitative studies, including those linked to observational, experimental, mixed methods papers. Interviews, focus groups or a combination), Thematic	Other	Antihypertensives	HBP	Medium	3	Includes children and adults. Other medications not of interest included

For peer review only - http://bmjopen.bmj.com/site/about/guidelines.xhtml

Focus/Aim	Study (First author, date)	Type of Review (design of studies included), synthesis methods	Eligible age of participants within studies included in the review	Medications of interest	Medical condition for which medication of interest being taken	Relevance of systematic review to aim of evidence-gap map	CEESAT set	Other comments
Patient views: intervention to encourage deprescribing Family/carer views: intervention to encourage deprescribing	Reeve, 2013	SR (Mixed: No limits placed on the type of methods used, qualitative, quantitative or mixed), Narrative/Content analysis/Thematic	Other *No limits were placed on the age of the participants*	Antihypertensives	HBP Anxiety NR	Medium	3	Methods of synthesis unclear
Evaluating intervention: enhance adherence	Reeves, 2020	SR (Quantitative: RCTs), Narrative	18 years or over *Adult*	Antihypertensives	HBP	High	2	
Evaluating intervention: enhance adherence One of multiple aims	Sanders, 2011	SR (Quantitative: comparative clinical studies of any design, including RCTs, non-RCTs, retrospective/prospective cohort, and case-control), Narrative+Meta-analysis	18 years or over	Antihypertensives	HBP *Essential hypertension*	Medium	1	Adherence low on the list of outcomes
Evaluating intervention: optimise prescribing	Shanbhag, 2018	SR (Quantitative: RCTs, cohort (with comparisons), controlled and uncontrolled BA, ITS), Narrative	NR	Antihypertensives	HBP *Associated with heart failure*	High	1	
Evaluating intervention: enhance adherence	Stacey, 2017	SR (Quantitative: RCTs), Meta-analysis	18 years or over	Antihypertensives	HBP Primary prevention CVD Seconday prevention CVD	Medium	1	Study focus: RE: making decisions about screening or treatment options for themselves, a child, or an incapacitated significant other

For peer review only

For peer review only - http://bmjopen.bmj.com/site/about/guidelines.xhtml

Focus/Aim	Study (First author, date)	Type of Review (design of studies included), synthesis methods	Eligible age of participants within studies included in the review	Medications of interest	Medical condition for which medication of interest being taken	Relevance of systematic review to aim of evidence-gap map	CEESAT set	Other comments
Evaluating intervention: enhance adherence	Tsioufis, 2020	Rapid review (Quantitative: RCTs, observational studies), Narrative	Other *Adults. Where reported, mean age of populations in included studies ranged from 47 to 71.5 years*	Antihypertensives	HBP	High	3	
Evaluating intervention: enhance adherence	Uhlig, 2013	SR (Quantitative: prospective comparative), Narrative+Meta-analysis	Other *Adults*	Antihypertensives	HBP	High	3	
Evaluating intervention: enhance adherence	van Driel, 2016	SR (Quantitative: RCTs of parallel-group or cross-over design, that used individual or cluster randomisation), Narrative+Meta-analysis	18 years or over	Statins	Primary prevention CVD Seconday prevention CVD	High	1	
Evaluating intervention: enhance adherence	Xiong, 2018	SR (Quantitative: RCTs, non-RCTs, before-and-after studies without a control group), Narrative	NR *All studies involved adults aged from 40 to 69*	Antihypertensives	HBP	High	3	
Evaluating intervention: enhance adherence	Xu, 2018	SR (Quantitative: RCTs), Meta-analysis	18 years or over	Antihypertensives	HBP	Medium	3	Focus on Chinese patients

For peer review only

Focus/Aim	Study (First author, date)	Type of Review (design of studies included), synthesis methods	Eligible age of participants within studies included in the review	Medications of interest	Medical condition for which medication of interest being taken	Relevance of systematic review to aim of evidence-gap map	CEESAT set	Other comments
Evaluating intervention: optimise prescribing Secondary aim Evaluating intervention: enhance adherence Secondary aim	Yuan, 2019	SR (Quantitative: RCTs, high quality cohort studies), Narrative+Meta-analysis	NR	Antihypertensives	HBP Primary prevention CVD Seconday prevention CVD *Assumed from content of paper*	Medium	3	

Green highlighted text=High overall quality as appraised by AMSTAR-2, Turquoise highlighted text=Medium overall quality as appraised by AMSTAR-2, Orange highlighted text=Low overall quality as appraised by AMSTAR-2, Red highlighted text=Critically-low quality as appraised by the AMSTAR-2. *CEESAT Set: 1=prioritised for full quality appraisal using AMSTAR-2, CEESAT Set 2=Scored poorly on 1 out of 4 items on CEESAT, CEESAT Set 3=Scored poorly on 2-4 items on CEESAT. BA=Before and After, BPD=Borderline Personality Disorder, CBA=Controlled Before and After, CPG=Clinical Practice Guidelines, CT=Controlled-Trials, CVD=Cardiovascular Disease, FH=Familial Hypercholesterolemia, HPP=High Blood Pressure, ITS=Interrupted Time Series, MA=Meta-analysis, NR=Not Reported, PPI=Proton Pump Inhibitors, PTSD=Post-Traumatic Stress Disorder, RCT=Randomized Controlled Trial, RQ=Research Question, SR=Systematic Review

For peer review only - http://bmjopen.bmj.com/site/about/guidelines.xhtml

Table 4: Systematic reviews appraised using AMSTAR-2 which synthesised studies evaluating the effectiveness of an intervention

Focus/ Aim	Study (First author, date)	Medications of interest	Other medications included	Intervention name/s and aim	Outcomes measured	Setting/Context	Relevant part of care pathway
Evaluating intervention: deprescribing	Dills, 2018	Statins Antihypertensives	Diuretics, salmeterol, insulin, sulfonylurea, antipsychotics, anxiolytic, CV drugs, levodopa, nitrate, benzodiazepines Z-drugs Antidepressants	Deprescription: reduce medication burden, and enhance control of chronic medical and mental health conditions commonly managed by primary care physicians, compared with standard care in the non-terminally ill adult population	Primary outcome: successful deprescription, Secondary outcome: AE related to drug or underlying chronic condition as a result of deprescription	Primary/health /social care Hospital (inpatient) Hospital (outpatient) Care home Patient home	Decision to stop treatment: supported
Evaluating intervention: enhance adherence	Stacey, 2017	Antihypertensives	Hepatitis B vaccination, schizophrenia medication, immunotherapy for multiple sclerosis, vaccines (measles, mumps, rubella), radioactive iodine treatment for thyroid cancer, antibiotic use for upper respiratory infections, chemotherapy for cancer, medications for diabetes, hormone therapy, "breast cancer prevention medication, bisphosphonates, thyroid cancer radioactive iodine treatment	Decision aids for people facing health treatment or screening decisions.<br>Aim: help people make specific and deliberated choices among options, by making the decision explicit/by providing information on the options and outcomes relevant to person's health status as well as implicit methods to clarify values	Attributes of choice made: does patient decision aid improve match between chosen option and features that matter most to informed patient (demonstrated by knowledge, accurate risk perceptions, values-choice congruence).<br><br>Attributes of decision-making process: does patient decision aid help patients to recognize decision needs to be made, feel informed about options and their features, be clear about option features that matter most, discuss values with their clinician, and become involved in decision making? Other decision-making process variables: Decisional conflict, Patient-clinician communication, participation in decision making, proportion undecided, satisfaction with the choice/process of decision making/preparation for decision	NR	Information and/or advice Shared decision making Patient adherence

Focus/ Aim	Study (First author, date)	Medications of interest	Other medications included	Intervention name/s and aim	Outcomes measured	Setting/Context	Relevant part of care pathway
					making. Secondary outcomes: Choice (the actual choice implemented; if not reported, the participants preferred option), adherence to chosen option, health status and quality of life (generic and condition-specific), anxiety, depression, emotional distress, regret, confidence, costs, cost-effectiveness, consultation length, litigation rates		

For peer review only - http://bmjopen.bmj.com/site/about/guidelines.xhtml

Focus/ Aim	Study (First author, date)	Medications of interest	Other medications included	Intervention name/s and aim	Outcomes measured	Setting/Context	Relevant part of care pathway
Evaluating intervention: enhance adherence*	Posadzki 2016	Statins Antihypertensives	Asthma medication, prescription drugs taken without prescription or more than prescribed, illicit non-injection drugs, glaucoma medications, inhaled corticosteroids, insulin, methadone maintenance, cholinesterase inhibitor medications, oral hypoglycaemic medications, nicotine replacement therapy, bupropion, and varenicline, antiretroviral treatment, bone active medication, non-hormonal oral agents, opiates antidepressants	Automated telephone communication systems for preventing disease and managing long-term conditions	Health behaviour and clinical outcomes, changes in health-enhancing behaviour, risk-taking behaviour, physiological measures, blood biochemistry. Process outcomes: change in acceptability of service (e.g. consumer accessibility/usability of interventions to apply information/support supplied), satisfaction (e.g. patient/carer) the intervention), cost-effectiveness. Cognitive outcomes: changes in knowledge (i.e. accurate risk knowledge and perception), attitude and intention to change, self-efficacy. Patient-centred outcomes: quality of life, adverse outcomes, unintended AE attributable to intervention	Primary/health/ social care Secondary health/social care (not hospital) Hospital (inpatient) Hospital (outpatient) Patient home	Identifying health or prescribing concern Accessing care Choosing a medication Decision to start agreed treatment plan Patient adherence Maintaining treatment
Evaluating intervention: enhance adherence	Adler, 2017	Statins Antihypertensives	Antiplatelet	Mobile phone text messaging to enhance adherence to recommended medication in patients with established arterial occlusive events	Adherence to treatment, fatal/non-fatal cardiovascular events, combined CVD event, Surrogate outcomes according to the different interventions recommended for secondary prevention including: low-density lipoprotein-cholesterol, blood pressure, heart rate, urinary 11-dehydrothromboxane B2, AEs	Primary/health /social care Hospital (outpatient)	Patient adherence

For peer review only - http://bmjopen.bmj.com/site/about/guidelines.xhtml

Focus/ Aim	Study (First author, date)	Medications of interest	Other medications included	Intervention name/s and aim	Outcomes measured	Setting/Context	Relevant part of care pathway
Evaluating intervention: enhance adherence	Fletcher, 2015	Antihypertensives	NA	Self-monitoring of blood pressure: improve medication adherence, medication persistence, and lifestyle factors in people with hypertension	Primary outcomes: antihypertensive medication adherence and persistence, dietary outcomes, alcohol consumption and physical activity. Secondary outcomes: BP, BP control, and adherence to SMBP component of interventions	Primary/health/ social care<br>Hospital (outpatient)	Decision to start agreed treatment plan<br>Patient adherence<br>Progress review
Evaluating intervention: enhance adherence	Morrissey, 2017	Antihypertensives	NA	Interventions to enhance medication adherence and blood pressure control in hypertension	Change in BP, medication adherence	Primary/health/ social care<br>Hospital (inpatient)<br>Hospital (outpatient)<br>Care home<br>Patient home	Patient adherence
Evaluating intervention: enhance adherence	Nieuwlaat, 2014	Antidepressants<br>Statins<br>Antihypertensives	Medications to treat following conditions: HIV/AIDS, psychiatric disorders, chronic obstructive pulmonary disease, CVD/CV risk, diabetes, antibiotics, arthritis, complex chronic care, dyspepsia, glaucoma, oral anticoagulation, osteoporosis, tuberculosis, acne, cancer, hepatitis, iron supplementation during pregnancy, liver transplant, malaria, oral contraceptives,	Interventions to affect adherence with prescribed, self-administered medications	Medication adherence, clinical outcomes	Primary/health/ social care<br>Hospital (inpatient)<br>Hospital (outpatient)<br>Care home<br>Patient home	Information and/or advice<br>Patient adherence<br>Progress review

For peer review only

Focus/ Aim	Study (First author, date)	Medications of interest	Other medications included	Intervention name/s and aim	Outcomes measured	Setting/Context	Relevant part of care pathway
			antidepressants tonsillectomy/adenoidectomy, ulcerative colitis				
Evaluating intervention: enhance adherence	Palmer, 2018	Statins Antihypertensives	Antiplatelet drugs (low-dose aspirin, non-aspirin antiplatelet drugs)	Mobile phone-based interventions to improve adherence to medication prescribed for the primary prevention of cardiovascular disease in adults	Objective measures of adherence to treatment, combined CVD events, AE, indirect measures of adherence to treatment, health-related quality of life, cognitive outcomes (satisfaction with treatment, medication-taking self-efficacy, autonomy related to medication, attitudes (e.g. concerns about medicine AEs)), costs, process measures: extent of intervention received and acceptability of intervention	Primary/health/ social care Secondary health/social care (not hospital) Hospital (outpatient) Patient home	Progress review Patient adherence
Evaluating intervention: enhance adherence	van Driel, 2016	Statins	Any lipid-lowering medication	Interventions to improve adherence to lipid-lowering drugs: 1. Simplification of drug regimen; 2. Patient education and information; 3. Intensified patient care; 4. Complex behavioural approaches; 5. Decision support systems; 6. Administrative improvements; and 7. Pharmacy-led interventions	Indirect measures of adherence, subjective measures of adherence, direct measures of adherence, physiological indicators, health outcome indications (e.g. quality of life, morbidity, mortality), AEs, implications for costs (impact of intervention on economic outcomes, economic evaluation)	Primary/health /social care Secondary health/social care (not hospital)	Information and/or advice Patient adherence Progress review

For peer review only - http://bmjopen.bmj.com/site/about/guidelines.xhtml

Focus/ Aim	Study (First author, date)	Medications of interest	Other medications included	Intervention name/s and aim	Outcomes measured	Setting/Context	Relevant part of care pathway
Evaluating intervention: enhance adherence*	Sanders, 2011	Antihypertensives	NA	ACEIs to treat essential hypertension	Blood pressure control, mortality, morbidity, safety, specific adverse events, persistence/adherence, rate of use of a single antihypertensive medication for blood pressure control, lipid levels, rates of progression to type 2 diabetes, carbohydrate metabolism/diabetes control, left ventricular mass/function, kidney disease	Primary/health/ social care<br>Secondary health/social care (not hospital)<br>Hospital (outpatient)<br>Other<br>*Setting of studies not restricted*	Choosing a medication<br>Progress review<br>Patient adherence
Evaluating intervention: enhance adherence*	Mallat, 2016	Antihypertensives	NA	Free versus fixed drug combination. Aim: to increase adherence, increase overall BP-lowering efficacy	Mortality, morbidity (cardiovascular outcomes such as coronary events, stroke, progression of peripheral vascular disease and kidney disease), mean systolic BP, AE, adherence to treatment	NR	Choosing a medication<br>Patient adherence<br>Progress review

For peer review only - http://bmjopen.bmj.com/site/about/guidelines.xhtml

Focus/ Aim	Study (First author, date)	Medications of interest	Other medications included	Intervention name/s and aim	Outcomes measured	Setting/Context	Relevant part of care pathway
Evaluating intervention: enhance adherence*	de Cates, 2014	Statins Antihypertensives	NA	A fixed-dose combination therapy, a combination of several active components into a single pill, including at least one statin and one antihypertensive agent. Aim: to optimise CVD risk and reduce CVD fatal and non-fatal events	Mortality, non-fatal CVD endpoints, AEs (overall rates of discontinuation, proportion of participants experiencing specific symptoms or results and rates of discontinuation by specific symptoms). Secondary outcomes: Systolic and diastolic blood pressure, total and LDL cholesterol, adherence, health-related QOL, costs of fixed-dose combination therapy	Primary/health/ social care	Choosing a medication<br>Progress Review<br>Patient adherence
Evaluating intervention: enhance adherence*	Maimaris, 2013	Antihypertensives	NA	National or regional health system arrangements on HT awareness, treatment, control, and antihypertensive medication adherence	HT awareness (persons with clinically measured HT diagnosed by a health care professional as hypertensive), HT treatment (use of at least one antihypertensive medication in an individual with known HT), antihypertensive medication adherence, HT control	Primary/health/ social care<br>Secondary health/social care (not hospital)<br>Hospital (inpatient)<br>Hospital (outpatient)	Identifying health or prescribing concern<br>Accessing care<br>Decision to start agreed treatment plan<br>Patient adherence<br>Other<br>*health systems, infrastructure. System level interventions*

For peer review only - http://bmjopen.bmj.com/site/about/guidelines.xhtml

Focus/ Aim	Study (First author, date)	Medications of interest	Other medications included	Intervention name/s and aim	Outcomes measured	Setting/Context	Relevant part of care pathway
Evaluating intervention: optimise prescribing	Gillaizeau, 2013	Antidepressants	Insulin, aminoglycoside antibiotics, anticoagulants, theophylline, anti-rejection drugs, anaesthetic agents, gonadotropins	Computerized advice on drug dosage to improve prescribing practice computerized advice on drug dosage: optimize effects and minimize dangers	Proportion of participants/time: plasma drug concentrations within therapeutic range, studied physiological parameter within the target range. "Time to achieve therapeutic control, proportion of participants: with toxic drug levels, with clinical improvement, with adverse effects of drug therapy. Proportion of deaths, LOS, total cost/participant	Primary/health /social care<br>Secondary health/social care (not hospital)<br>Hospital (inpatient)<br>Hospital (outpatient)	Choosing a medication<br>Prescribing guidelines<br>Progress review
Evaluating intervention: optimise prescribing	Hukins, 2019	Statins<br>Hypnotics/Z-drugs	Anticholinergic medications, anxiolytics, oestrogens, NSAID, antipsychotics, antiplatelets, proton pump inhibitors, benzodiazepines	Any tool (dementia-specific or non-disease-specific) to identify potentially inappropriate prescribing	Prevalence of polypharmacy, prevalence of PIP, most common prescribed PIP medications	Primary/health/ social care<br>Secondary health/social care (not hospital)<br>Hospital (inpatient)<br>Hospital (outpatient)<br>Care home<br>Patient home	Identifying health or prescribing concern<br>Progress review<br>Decision to stop treatment: supported
Evaluating intervention: optimise prescribing*	Pantoja, 2019	Benzodiazepines<br>Hypnotics/Z-drugs<br>Statins<br>Antihypertensives	Anticonvulsants, antibiotic therapy, NSAID, nicotine gum, ezetimibe, thromboprophylaxis	Manual paper reminders to improve compliance with preventive guidelines and disease management guidelines	Patient-important endpoints: death, development of a pulmonary embolism , surrogate or intermediate endpoints: achievement of target blood pressure or serum cholesterol level, markers of disease/health status, adverse effects, resource use, changes in professional practice, patient outcomes	Primary/health /social care<br>Hospital (inpatient)<br>Hospital (outpatient)	Progress review<br>Identifying health or prescribing concern<br>Initial assessment<br>Prescribing guidelines

Focus/ Aim	Study (First author, date)	Medications of interest	Other medications included	Intervention name/s and aim	Outcomes measured	Setting/Context	Relevant part of care pathway
Evaluating intervention: optimise prescribing*	Legare, 2010	Statins	Not clear	Interventions for improving the adoption of shared decision making by healthcare professionals	Healthcare professionals' adoption of SDM	Primary/health/ social care Secondary health/social care (not hospital)	Initial assessment Information and/or advice Choosing a medication Shared decision making Decision to start agreed treatment plan
Evaluating intervention: optimise prescribing	Jeffery, 2015	Statins Antihypertensives	NA	Interventions to improve adherence to CVD guidelines and patient outcomes: education, audit and feedback, academic detailing, comprehensive interventions that included education, audit and feedback and an academic detailing component	Guideline adherence, clinical outcomes	Primary/health/ social care Secondary health/social care (not hospital) Hospital (inpatient)Hospital (outpatient)	Identifying health or prescribing concern Initial assessment Information and/or advice Prescribing guidelines Progress review
Evaluating intervention: optimise prescribing	Shanbhag, 2018	Antihypertensives	NA	Implementation interventions classified at the provider, organisational or health system levels. Aim: increasing physician adherence to the specified HF guideline recommendations	Patient-level outcomes. Primary outcomes: process indicators (assessing guideline-consistent activities undertaken by a provider, proportion of patients who were: prescribed a guideline-recommended pharmacological treatment, referred for implantable cardioverter defibrillator, CRT consideration, provided self-care education at discharge, LVEF. Secondary outcomes: HF	Hospital (inpatient) Hospital (outpatient)	Prescribing guidelines Decision to start agreed treatment plan Other

For peer review only - http://bmjopen.bmj.com/site/about/guidelines.xhtml

Focus/ Aim	Study (First author, date)	Medications of interest	Other medications included	Intervention name/s and aim	Outcomes measured	Setting/Context	Relevant part of care pathway
					specific/all-cause clinical outcomes		

*A secondary aim or one of multiple aims of review. Green highlighted text=High Overall Quality AMSTAR-2, Turquoise highlighted text=Medium Overall Quality AMSTAR-2, Orange highlighted text=Low Overall Quality AMSTAR-2, Red highlighted text=Critically-low Overall Quality AMSTAR-2. ACEI= Angiotensin-Converting Enzyme Inhibitors, AE=Adverse Events, AIDS= Acquired Immunodeficiency Syndrome, BEZRA=Benzodiazepines/Zdrugs, BP=Blood Pressure, CBT=Cognitive Behavioural Therapy, CPM=Continuous Passive Motion, CRT= Cardiac Resynchronisation Therapy, CV=Cardiovascular, CVD=Cardiovascular Disease, DBI=Drug Burden Index, ED=Emergency Department, GDR=Gradual Dose Reduction, GP=General Practitioner, HF=Heart Failure, HIV= Human Immunodeficiency Virus, HT=Hypertension, LDL= Low-Density Lipoprotein, LOS=Hospital Length of Stay, NA=Not Applicable, NR=Not Reported, QOL=Quality Of Life, LVEF= Left Ventricular Ejection Fraction, MBCBT=Mindfulness Based Cognitive Behavioural Therapy, NSAID=Non-Steroidal Anti-Inflammatories, PIP=Potentially Inappropriately Prescribed, SAE=Serious Adverse Events, SDM=Shared Decision Making, SMBP=Self-Monitoring of Blood Pressure, TKA=Total Knee Arthroplasty

For peer review only

Table 5: Systmatic reviews scoring negatively on one CEESAT item which syntehsised studies evaluating the effectiveness of an intervention

Focus/ Aim	Study (First author, date)	Medications of interest	Other medications included	Intervention name/s and aim	Outcomes measured	Setting/Context	Relevant part of care pathway
Evaluating intervention: deprescribing	Page, 2016	Statins Antihypertensives	Clopidogrel, Cilostazol, pentoxifylline, diuretics, nitrates, Glucosamine, bisphosphonates, calcium, vitamin D, Calcitriol, conjugated equine estrogens Premarin combined with medroxyprogesterone acetate, Carbamazepine, Levodopa, lithium, anticholinestera, prednisolone, corticosteroids, d beta-2 receptor agonist, antipsychotics, benzodiazepines antidepressants	Deprescribing by a health care professional of one or more regular prescription medications:	Mortality, adverse drug withdrawal events, physical health, cognitive function and psychological health, quality of life	Hospital (inpatient) Care home Primary/health/ social care Hospital (outpatient) Patient home	Progress review Decision to stop treatment: supported

Focus/ Aim	Study (First author, date)	Medications of interest	Other medications included	Intervention name/s and aim	Outcomes measured	Setting/Context	Relevant part of care pathway
Evaluating intervention: enhance adherence	Park, 2017	Antihypertensives<br>Statins	Antidementia medication, pneumonia-related ambulatory antibiotics, antilpemics, insulin, biologics, oral diabetes drugs, antiasthmatics, pain reliever, phosphate binders, cinacalce, teriparatide, antidepressants	Medicare Part D: increase drug utilization and lower Medicare and Medicaid participants out- of-pocket prescription drug costs, thereby improving access	Out-of-pocket costs, drug utilization	Primary/health/social care<br>Hospital (inpatient)<br>Hospital (outpatient)	Choosing a medication<br>Patient adherence<br>Progress review
Evaluating intervention: enhance adherence	Conn, 2015[70]	Statins<br>Antihypertensives	Yes, in relation to following medical conditions: organ transplant, epilepsy, gastrointestinal, infections, chronic diseases, malaria, STD, neurological, HIV	Packaging interventions to improve adherence: blister packs, unit-packaging, unit-of-use systems, unit-of-dose packaging,<br>monitored dosage systems	Adherence, health outcomes	Primary/health/social care<br>Hospital (inpatient)<br>Patient home	Patient adherence

For peer review only - http://bmjopen.bmj.com/site/about/guidelines.xhtml

Focus/ Aim	Study (First author, date)	Medications of interest	Other medications included	Intervention name/s and aim	Outcomes measured	Setting/Context	Relevant part of care pathway
Evaluating intervention: enhance adherence	Conn, 2015[71]	Statins *Inferred from population disease characteristics* Antihypertensives *Inferred from population disease characteristics*	Yes, in relation to following medical conditions: diabetes, HIV, renal disease, asthma, lung disease. stroke, gastrointestinal diseases, acute illness, allergies, autoimmune disease, blood disorders, cancer, eye problems, gynaecological disorders, liver disease, malaria, nervous system problems, osteoarthritis, osteoporosis, rheumatoid arthritis, seizures, skin diseases, organ transplant, tuberculosis, and vascular disease	Health care provider targeted interventions to improve medication adherence: Research staff trained providers, integration of care across providers, increased providers, communication skills, monitor patients adherence behaviour, Provider- patient medication concordance, Increased provider time with patients, reduce the geographical distance between providers and patients, increase continuity of care, activating provider	Adherence behaviour	Primary/health/social care	Accessing care Working with other health or social care services/ charities Shared decision making Decision to start agreed treatment plan Patient adherence Progress review

Focus/ Aim	Study (First author, date)	Medications of interest	Other medications included	Intervention name/s and aim	Outcomes measured	Setting/Context	Relevant part of care pathway
Evaluating intervention: enhance adherence	Conn, 2016	Statins<br>Antihypertensives<br>*All Inferred from participant sample characteristics*	Yes, in relation to following medical conditions: gallbladder disease, unspecified chronic illnesses, osteoarthritis, asthma, IBD, glaucoma, kidney transplant, anti-coagulant therapy, osteoarthritis stroke, diabetes, HIV, antidepressants	Medication Adherence Interventions: Intervention content could include: prompts/cues to administer medications, self-monitoring of medication administration, self-monitoring of disease symptoms, written instructions, rewards for increased adherence, increased communication between providers and patients, providing feedback to participants about their adherence, goal setting about adherence, habit assessment/modification, and problem solving about adherence challenges	Adherence	Primary/health/social care<br>Secondary health/social care (not hospital)<br>Patient home	Decision to start agreed treatment plan<br>Patient adherence<br>Progress review
Evaluating intervention: enhance adherence	De Simoni, 2013	Antihypertensives	NA	Interventions to improve adherence to antihypertensive medications: education, lifestyle, verbal information/advice on disease and secondary-prevention drug treatment, goal setting, supply of printed information/advice material, screening for depression, personalized instructions, and integrated care	Adherence and blood pressure control	Primary/health/social care<br>Hospital (outpatient)<br>Patient home	Information and/or advice<br>Decision to start agreed treatment plan<br>Patient adherence<br>Progress review

For peer review only - http://bmjopen.bmj.com/site/about/guidelines.xhtml

Focus/ Aim	Study (First author, date)	Medications of interest	Other medications included	Intervention name/s and aim	Outcomes measured	Setting/Context	Relevant part of care pathway
Evaluating intervention: enhance adherence	Kassavou, 2018	Statins Antihypertensives	Yes, NR	Automated telecommunication systems, including voice messaging and text messaging: promote adherence to cardio-metabolic medications	Adherence	Primary/health/social care Secondary health/social care (not hospital) Hospital (inpatient) Hospital (outpatient) Patient home	Patient adherence
Evaluating intervention: enhance adherence	Krack, 2019	Statins Antihypertensives	Calcium channel blockers, Clopidogrel, insulin oral antidiabetics, diabetes drugs, inhaled steroids, metformin, thiazides, Inhaled corticosteroid	Value-based health insurance designs including: VBID vs usual medication coverage VBID+education vs usual medication coverage, VBID+education vs usual medication coverage+education<br><br>Aim: increase patients understanding of importance of high-value medications, increase number of adherent patients without increasing total health care spending	Adherence	Primary/health/social care	Information and/or advice Patient adherence
Evaluating interventio n: enhance	Kronish, 2011	Antihypertensives	NA	Drug Class: improve adherence to antihypertensive medication	Adherence	NR	Choosing a medication Patient adherence

For peer review only - http://bmjopen.bmj.com/site/about/guidelines.xhtml

Focus/ Aim	Study (First author, date)	Medications of interest	Other medications included	Intervention name/s and aim	Outcomes measured	Setting/Context	Relevant part of care pathway
Evaluating Intervention: enhance adherence	Lawrence, 2015	Statins Antihypertensives	Antithrombotic medication	Multimodal behavioural interventions for secondary stroke prevention: addresses: 1) medication education and/or medication compliance education, 2) education or active information provision e.g. about stroke, stroke (lifestyle) risk factors, and 3) one or more of four specified lifestyle behaviours i.e. smoking, diet, physical inactivity, and alcohol consumption, and/or behaviours associated with amelioration of lifestyle risk factors i.e. medication compliance and management of perceived psychosocial stress	Primary outcomes of interest: physiological outcomes e.g. blood pressure, blood lipids, and lifestyle behaviour change. Secondary outcomes: psychosocial outcomes e.g. anxiety, learning e.g. knowledge of lifestyle risk factors for stroke, incidence of vascular events and mortality	Secondary health/social care (not hospital) Patient home	Information and/or advice Patient adherence Progress review
Evaluating intervention: enhance	Mann, 2014	Statins Antihypertensives	NA	Drug Insurance Cost Sharing Strategies: lower expenditures for prescription drug insurance plans	Adherence, clinical events (myocardial infarction, stroke, death), QOL, healthcare utilization, or cost	NR	Accessing care Decision to start agreed treatment plan Patient adherence

For peer review only - http://bmjopen.bmj.com/site/about/guidelines.xhtml

Focus/ Aim	Study (First author, date)	Medications of interest	Other medications included	Intervention name/s and aim	Outcomes measured	Setting/Context	Relevant part of care pathway
Evaluating intervention: enhance adherence	Milosavljevic, 2018	Antihypertensives	Yes, in relation to following conditions: Asthma, diabetes, dyslipidemia, COPD, 'older people's health' aldosterone-inhibiting diuretics (for heart failure), intravenous vitamin D analogs, calcimimetic, oral hypoglycemics, antipsychotics, other central nervous system drugs, beta blockers, analgesics, antidepressants	Community pharmacist interventions: improve adherence, improve clinical outcomes.	Behavioural outcome: adherence, self-efficacy. Clinical outcome: clinical biomarkers (e.g. blood pressure, glycosylated haemoglobin, LDL), hospitalisation rates, mortality, emergency room visits, markers of disease progress. Economic outcome: cost effectiveness analysis, or other relevant analysis. Humanistic outcome: patient quality of life, knowledge, or satisfaction	Primary/health/social care Patient home	Information and/or advice Choosing a medication Decision to start agreed treatment plan Progress review Patient adherence

For peer review only - http://bmjopen.bmj.com/site/about/guidelines.xhtml

Focus/ Aim	Study (First author, date)	Medications of interest	Other medications included	Intervention name/s and aim	Outcomes measured	Setting/Context	Relevant part of care pathway
Evaluating intervention: enhance adherence	Rash, 2016	Statins	NA	Interventions targeting medication adherence:<br>Prescription cost coverage, simplification of drug regimen, reminders, education-based, multi-faceted interventions (including one or more of following: education, counselling, behavioural interventions, follow-up, medication review, risk-factor and adherence education, personalized health reports, bi-monthly mailings, collaborative care)	Patient adherence	Primary/health/social care<br>Secondary health/social care (not hospital)<br>Hospital (inpatient)<br>Hospital (outpatient)	Information and/or advice<br>Working with other health/social care services<br>Choosing a medication<br>Shared decision making<br>Patient adherence<br>Progress review
Evaluating interventio n: enhance	Reeves, 2020	Antihypertensives	NA	Pharmacist-led interventions to improve medication adherence and BP control	BP control, medication adherence	Primary/health/social care<br>Hospital (inpatient)<br>Hospital (outpatient)	Information and/or advice<br>Patient adherence<br>Progress review
Evaluating intervention: enhance adherence*	Cheema, 2014	Antihypertensives	NA	Interventions to improve management of BP: Pharmacological: education on drug treatment of BP, advice to patients to improve medication adherence, identifying drug adverse effects and drug prescribing issues, liaising with prescribers about concerns of drug treatment.<br>Nonpharmacological: education about hypertension. education about lifestyle (advice to patients on diet, weight management, alcohol consumption, smoking cessation)	Systolic and diastolic BP, adherence, identification and management of drug-related problems, cardiovascular risk factors, i.e. smoking, alcohol consumption	Primary/health/social care	Information and/or advice<br>Working with other health/social care services<br>Identifying health or prescribing concern<br>Patient adherence<br>Progress review

For peer review only - http://bmjopen.bmj.com/site/about/guidelines.xhtml

Focus/ Aim	Study (First author, date)	Medications of interest	Other medications included	Intervention name/s and aim	Outcomes measured	Setting/Context	Relevant part of care pathway
Evaluating intervention: optimise prescribing	Nguyen, 2018	Statins	Aspirin, antiplatelet agents	Organisational or professional interventions to enhance prescribing of guideline-recommended medications. Interventions included: Distribution of educational materials, educational outreach visits, audit and feedback and reminders, continuity of care, communication and case discussions between distant healthcare professionals, distribution of published/printed recommendations for clinical care, including clinical practice guidelines	Proportion of patients receiving guideline-recommended medications. Secondary outcomes: proportion of patients achieving target blood pressure and target LDL, C/cholesterol level, mortality rate	Primary/health/social care<br>Hospital (outpatient)	Identifying health or prescribing concern<br>Initial assessment<br>Information and/or advice<br>Choosing a medication<br>Prescribing guidelines
Evaluating intervention: optimise prescribing<br>Evaluating intervention: enhance	King, 2018	Statins<br>Antihypertensives	Diabetic medication, Digoxin, mixed medications, antidepressants	Longer-duration (2–4 months) versus shorter-duration (28-day)prescriptions to improve patient outcomes	Health outcomes, adverse events, medication adherence, medication wastage, professional administration time, pharmacists time and/or costs, patient experience, and patient out-of-pocket costs	Primary/health/social care<br>Secondary health/social care (not hospital)<br>Hospital (inpatient)	Initial assessment<br>Choosing a medication<br>Decision to start agreed treatment plan<br>Patient adherence<br>Progress review

*Secondary aim or outcome within the review. AE=Adverse Events, BI=Behavioural Interventions, BP=Blood Pressure, BZ=Benzodiazepine, CBT=Cognitive Behavioural Therapy, COPD=Coronary Obstructive Pulmonary Disease, DBT=Dialectical Behavioural Therapy, ED=Emergency Department, GP=General Practitioner, HIV= Human Immunodeficiency Virus, IBD=Irritable Bowel Disease, LDL= Low-Density Lipoprotein, LTOT=Long-Term Opioid Therapy, MDD=Major Depressive Disorder, MI=Motivational Interviewing, NA=Not Applicable, NR=Not Reported, QOL=Quality of Life, VBID=Value Based Insurance Design

For peer review only - http://bmjopen.bmj.com/site/about/guidelines.xhtml

Table 6: Systematic reviews scoring negatively on 2-4 CEESAT items which synthesised studies evaluating the effectiveness of an intervention

Focus/ Aim	Study (First author, date)	Medications of interest	Other medications included	Intervention name/s and aim	Outcomes measured	Setting/Context	Relevant part of care pathway
Evaluating intervention: deprescribing*.	Hart, 2020	Antihypertensives	Antipsychotics, Antihistamines, Antivertigo, Anti-Parkinson, Urinary spasmolytics, Vasodilators, Digoxin, Diuretics, Beta Blockers, Hypoglycemics, Beta blocker eye drops, Alpha adrenergic antagonists, Alpha blockers, Benzodiazepines Opiates Hypnotics/Z-drugs Antidepressants	Interventions to reduce falls or use of FRIDs: Face-to-face medication consultations with community pharmacy resident, fall-related assessment performed by research physician, geriatrician assessment of falls risk and systematic medication review, consideration of potential FRIDs for withdrawal+prescribing physician contact	Change in FRID use	Hospital (inpatient) Hospital (outpatient)	Initial assessment Choosing a medication Progress review Decision to stop treatment: supported Other *Medication review in acute care (e.g. after hip fracture)*
Evaluating intervention: enhance adherence	Al Alshaikh, 2016	Statins Antihypertensives	Aspirin, Dipyridamole, antithrombotics, antiplatelets, anticoagulants, diabetic medications	Multimodal Interventions to improve adherence: categories included educational+motivational, simplification of drug regimen, environmental cues or reminders+reducing concerns and misbeliefs regarding medications, combined interventions to enhance risk factor management after stroke, prescribed medications+educated about them	Medication adherence	Primary/health/social care Hospital (inpatient) Hospital (outpatient) Patient home	Information and/or advice Patient adherence

For peer review only - http://bmjopen.bmj.com/site/about/guidelines.xhtml

Focus/ Aim	Study (First author, date)	Medications of interest	Other medications included	Intervention name/s and aim	Outcomes measured	Setting/Context	Relevant part of care pathway
Evaluating intervention: enhance adherence	Baumgartner, 2020	Statins<br>Antihypertensives	Yes, in relation to following medical conditions:<br>HIV, diabetes, tuberculosis<br><br>Specific medications include:<br>metformin/sulfonylurea, various oral antidiabetic drugs, pioglitazone/ thiazolidinedione+ sulfonurea, FDC: rosiglitazone/glimepiride, glyburide/metformin, thiazolidinedione, Î±-blocker/antimuscarinic	Polypill to improve medication adherence	Adherence	Primary/health/soci al care<br>Hospital (inpatient)<br>Hospital (outpatient)	Choosing a medication<br>Decision to start agreed treatment plan<br>Patient adherence<br>Progress review
Evaluating intervention: enhance adherence	Bochkareva, 2019	Antihypertensives	NA	Medical Assistance Scheme to increase adherence to treatment: Examples include special packaging of medications, amount of prescribed medications, interventions to improve communication with patients (more frequent visits, motivational interviewing, patient education, home BP monitoring, and provision of written instructions), duration and number of sessions/consultations	Adherence	Primary/health/soci al care<br>Secondary health/social care (not hospital)<br>Hospital (outpatient)	Information and/or advice<br>Patient adherence

For peer review only - http://bmjopen.bmj.com/site/about/guidelines.xhtml

Focus/ Aim	Study (First author, date)	Medications of interest	Other medications included	Intervention name/s and aim	Outcomes measured	Setting/Context	Relevant part of care pathway
Evaluating intervention: enhance adherence	Checchi, 2014	Antihypertensives Statins	Various HAARTs, luticasone propionate, travoprost, pilocarpine, antidiabetic medications, ipratropium bromide, lithium, Clopidogrel, bupropion-SR, antipsychotics, fluticasone, sulfonylureas, immuno-suppressants	Electronic medication packaging devices to promote medication adherence	Adherence, health outcomes, cost effectiveness results, and satisfaction	Primary/health/social care<br>Patient home	Patient adherence<br>Information and/or advice
Evaluating intervention: enhance adherence	Conn 2015[36]	Antihypertensives	NA	Interventions to increase adherence among adults with hypertension	Adherence	Primary/health/social care<br>NR	Information and/or advice<br>Patient adherence<br>Progress review<br>Referral to specialist care

Focus/ Aim	Study (First author, date)	Medications of interest	Other medications included	Intervention name/s and aim	Outcomes measured	Setting/Context	Relevant part of care pathway
Evaluating intervention: enhance adherence	Deichmann, 2016	Statins	Fibrates, Anion-exchange resins or bile acid sequestrants, Niacin or nicotinic acid, combined medication regimen	Interventions to increase adherence to self-administered lipid-lowering medication: Drug regimen simplification, patient education and information, intensified patient care with reminders via mail, telephone, and hand-held pill devices, complex behavioural approaches, group sessions, decision support systems, administrative improvements, pharmacy-led automated telephone intervention	Adherence to lipid-lowering medical therapy. Secondary outcomes: physiologic indicators (e.g., total cholesterol), health outcome indications (e.g., QOL, morbidity, mortality), adverse effects, implications for costs	Primary/health/social care<br>Patient home	Information and/or advice<br>Choosing a medication<br>Patient adherence<br>Progress review
Evaluating intervention: enhance adherence	Du, 2018	Antihypertensives	NA	Fixed-dose combination versus free-equivalent combination therapies on adherence to medication for hypertension	Medication adherence/persistence	NR	Choosing a medication<br>Patient adherence<br>Progress review
Evaluating intervention: enhance adherence	Gupta, 2010	Antihypertensives	NA	Fixed-Dose Combinations of Antihypertensive agents. Aim: to achieve better BP control by improving compliance	Compliance (or adherence), persistence, BP-lowering efficacy, adverse effects	NR	Choosing a medication<br>Patient adherence<br>Progress review

Focus/ Aim	Study (First author, date)	Medications of interest	Other medications included	Intervention name/s and aim	Outcomes measured	Setting/Context	Relevant part of care pathway
Evaluating intervention: enhance adherence	Guzman-Tordecilla, 2020	Antihypertensives	NA	Interventions to enhance adherence and control of arterial hypertension: community fund organization, reminders, educational material and calendar, sessions for the optimization of the use of medicines, advice with experienced pharmacists, group educational meetings, obtention of medical records, identification of problems with medications and intervention on them, follow-up with the pharmacist and education	Adherence, control of HBP	Primary/health/social care<br>Patient home	Information and/or advice<br>Working with other health or social care services/charities<br>Patient adherence<br>Progress review
Evaluating intervention: enhance adherence	Kawalec, 2018	Antihypertensives	NA	Fixed-dose combination therapy. Aim: blood pressure lowering, increasing patients adherence	Medication adherence, blood pressure lowering, persistence, health outcomes and adverse events	Primary/health/social care<br>Patient home	Choosing a medication<br>Patient adherence<br>Progress review
-Evaluating intervention: enhance adherence	Leslie, 2016	Antihypertensives<br>Statins (assumed)	Oral diabetes medications	Interventions aimed to modify medication adherence behaviour to chronic medications administered orally to treat diabetes, hypertension, or hyperlipidaemia: behavioural counselling, education and Informational materials, medication packaging that increased convenience by combining pills in a blister pack	Adherence to medications	Primary/health/social care<br>Care home<br>Patient home	Information and/or advice<br>Patient adherence<br>Progress review

Focus/ Aim	Study (First author, date)	Medications of interest	Other medications included	Intervention name/s and aim	Outcomes measured	Setting/Context	Relevant part of care pathway
Evaluating intervention: enhance adherence	Mamudu, 2014	Statins Antihypertensives	Diabetic medications, Aspirin	Coronary artery calcium screening: enable risk stratification, diagnosis of CAD and directs the treatment and management of CAD among asymptomatic individuals	Behavioural modification, risk perception, and medication adherence, lifestyle change	Primary/health/social care	Identifying health or prescribing concern Initial assessment Patient adherence
Evaluating intervention: enhance adherence	Tsioufis, 2020	Antihypertensives	NA	Single-pill combination therapy to treat hypertension. Aim: improve clinical outcomes and medication adherence	Adherence and/or persistent, BP, cardiovascular outcomes, mortality or morbidity	Primary/health/social care	Choosing a medication Patient adherence Progress review
Evaluating intervention: enhance adherence	Uhlig, 2013	Antihypertensives	NA	Interventions to enhance management of hypertension: self-measured blood pressure with or without additional support	Clinical outcomes (e.g. death and cardiovascular events), patient-reported outcomes (e.g. patient satisfaction or QOL), surrogate outcomes (e.g. measures of left ventricular hypertrophy), intermediate outcomes (e.g. BP; number, dose, or changes of antihypertensive medications; adherence to antihypertensive medication), health care utilization (including visits, calls, and e-mails	Primary/health/social care Patient home	Patient adherence Progress review

For peer review only - http://bmjopen.bmj.com/site/about/guidelines.xhtml

Focus/ Aim	Study (First author, date)	Medications of interest	Other medications included	Intervention name/s and aim	Outcomes measured	Setting/Context	Relevant part of care pathway
Evaluating intervention: enhance adherence	Xiong, 2018	Antihypertensives	Immunosuppressant's (n=1), lipid-lowering medication (n=1)	Mobile health technology to enhance medication adherence	Medication adherence	Primary/health/social care<br>Secondary health/social care (not hospital)<br>Hospital (outpatient)<br>Patient home	Patient adherence
Evaluating intervention : enhance adherence	Xu, 2018	Antihypertensives	NA	Medication education, self-monitoring of BP, reminders and regular follow-up visits to improve medication adherence	Adherence, BP, BP control	Primary/health/social care<br>Hospital (outpatient) *might be inpatient*<br>Patient home	Patient adherence<br>Information and/or advice<br>Progress review
Evaluating intervention: enhance adherence	Demonceau, 2013	Antihypertensives *Assumed*	Medications for following medications:: HIV, asthma, osteoporosis<br>Specific medications: sulfonylurea drugs, oral nitrates	Interventions to affect adherence to self-administered prescribed medications in short-term and in long-term therapy: treatment simplification, cognitive-educational interventions, behavioural counselling, social-psycho-affective interventions, electronically monitored adherence feedback, technical reminder systems, monitoring the disease being managed, rewards, integrating depression treatment with hypertension management or type 2 diabetes care, home-based physician-directed, nurse-guided drug therapy, drug regimen	Health outcomes' e.g. reductions in blood pressure, lower levels of glycosylated haemoglobin, medication adherence	Primary/health/social care<br>Hospital (inpatient)<br>Hospital (outpatient)<br>Patient home	Information and/or advice<br>Working with other health/social care services<br>Patient adherence<br>Referral to specialist care

For peer review only - http://bmjopen.bmj.com/site/about/guidelines.xhtml

Focus/ Aim	Study (First author, date)	Medications of interest	Other medications included	Intervention name/s and aim	Outcomes measured	Setting/Context	Relevant part of care pathway
Evaluating intervention: enhance adherence<br>Evaluating intervention: optimise prescribing	Laba, 2013	Statins<br>Antihypertensives	Yes<br>*Not all medicines reported, but all for CVD* e.g. spironolactone, loop diuretics, digoxin	Strategies to improve patient adherence to cardiovascular medications:<br>education, persuasion, incentivisation, enablement, training, coercion, restriction, environmental restructuring, modelling, policies, communication/marketing, guidelines, fiscal, regulation, legislation, environmental/social planning, service provision	Adherence, Physiological: BP control, mean DBP/SBP, lipids, glucose/HbA1c, INR, echocardiogram, health outcomes, health service utilisation, mortality, QOL, other lifestyle, smoking/alcohol, nutrition, physical activity, stress, adverse events, cost comparison, cost effectiveness	Primary/health/social care<br>Patient home<br>Hospital (outpatient)	Information and/or advice<br>Shared decision making<br>Patient adherence<br>Progress review<br>Prescribing guidelines
Evaluating intervention: enhance adherence	Polinski, 2011	Statins<br>Antihypertensives	HEDIS, high risk drugs, anti-convulsants, antihistamines, propoxyphene, digoxin, NSAIDs, doxazosin, muscle relaxants/anti-spasmodics, others, warfarin, oral anti-diabetics, antiretroviral medications, warfarin, clopidogrel, antipsychotics, proton pump inhibitors, antibiotics, benzodiazepines antidepressants, hypnotics/Z-drugs (sedatives)	Medicare Part D cost-sharing provisions and drug coverage rules. Aim: improve access to essential medications through reduced cost-sharing with focus on under and over-use of specific drugs and classes	Reduced out-of-pocket costs, health	Primary/health/social care<br>Hospital (outpatient)	Accessing care<br>Choosing a medication<br>Decision to start agreed treatment plan<br>Patient adherence<br>Decision to stop treatment: unsupported

For peer review only - http://bmjopen.bmj.com/site/about/guidelines.xhtml

Focus/ Aim	Study (First author, date)	Medications of interest	Other medications included	Intervention name/s and aim	Outcomes measured	Setting/Context	Relevant part of care pathway
Evaluating intervention: optimise prescribing	Kang, 2016	Statins<br>Antihypertensives	Aspirin	Pharmacist-involved care (patient- targeted services) and healthcare professional-targeted services: PTS-counselling, providing educational materials, medical reminders, post-discharge follow- up services and supporting rehabilitation transition care. HTS-medication reconciliation, communication with health-care providers<br>Aim: improve CVD outcomes	All-cause mortality, all-cause hospitalization, cardiac-related hospitalization, key medication prescription rates, health-related QOL, medication adherence, medication utilization	Primary/health/social care<br>Hospital (inpatient)<br>Hospital (outpatient)<br>Patient home	Information and/or advice<br>Working alongside health/social care services<br>Choosing a medication<br>Patient adherence<br>Progress review
Evaluating intervention: optimise prescribing	Chhina, 2013	Antihypertensives	Propoxyphene, cerebral & peripheral vasodilators, cephalexin, amoxycillin with or without clavulinic acid, cephalexin, doxycycline, erythromycin, penicillin, rrimethoprim, cefaclor, roxithromycin, NSAIDS, metronidazole, omeprazole, inhaled steroids, antipsychotic, benzodiazepines antidepressants	Academic Detailing to modify drug prescription behaviour of Family Physicians in primary care settings	Prescription rate	Primary/health/social care	Choosing a medication<br>Progress review<br>Other<br>*Reducing repeat prescription*

Focus/ Aim	Study (First author, date)	Medications of interest	Other medications included	Intervention name/s and aim	Outcomes measured	Setting/Context	Relevant part of care pathway
Evaluating intervention: optimise prescribing* Evaluating intervention: enhance adherence*	Yuan, 2019	Antihypertensives	Yes, associated with following medical conditions: diabetes, chronic obstructive pulmonary disease, osteoporosis, obesity, antiphospholipid syndrome, drug related high risk of disease, outpatients taking multiple prescription drugs, bronchial asthma	Community Pharmacist interventions to improve outcomes for people with hypertension: medication review, patient education, adherence assessment, health/lifestyle advice, physical assessment (e.g., BP), monitoring, prescribing, or adjusting and administering therapy	Clinical outcomes of pharmaceutical services e.g. common end- points of systolic blood pressure, diastolic blood pressure and glycosylated haemoglobin after providing CPS	Primary/health/social care Patient home Hospital (inpatient)	Initial assessment Information and/or advice Decision to start agreed treatment plan Patient adherence Progress review

*Secondary aim or one of multiple aims; [a]Note that focus is on 'use' which includes initiating prescribing. Drugs of interest may be currently under- or over-used, [b]One aspect of broad aim, [c]One aspect of interventions. BP=Blood Pressure, CAD=Coronary Artery Disease, CPS=Community Pharmacist Services, CVD=Cardiovascular Disease, COX-2=Cyclooxygenase-2, DBP-Diastolic Blood Pressure, FDC=Fixed-Dose Combination, FRID=Fall Risk Increasing Drugs, HAART= Highly Active Antiretroviral Therapy, HBP=High Blood Pressure, HIV= Human Immunodeficiency Virus, HRT=Hormonal Replacement Therapy, HTS=Healthcare professional Targeted Services, INR=International Normalised Ratio, NA=Not Applicable, NR=Not Reported, NSAID=Non-Steroidal Anti-Inflammatories,PTS=Patient Targeted Services, SBP=Systolic Blood Pressure, SH=Sedative Hypnotics, SR=Sustained Release, QOL=Quality of Life,

For peer review only - http://bmjopen.bmj.com/site/about/guidelines.xhtml

Table 7: Systematic reviews synthesising qualitative evidence

Study (First author, date)	Medications of interest	Other medications included	Perspectives obtained	Phenomenon of Interest	Setting/Context	Relevant part of care pathway
Ingersgaard, 2020	Statins<br>Antihypertensives	Lipid-lowering agents, Î² blockers, angiotensin, renin system blockers, aspirin	Patient	Experiences/views of an intervention (adherence)<br>Reasons for adherence/non-adherence	Primary/health/social care<br>NR	Patient adherence
Rashid, 2014	Statins	Clopidogrel, antiplatelet medication, aspirin	Patient<br>Family member	Reasons for adherence/non-adherence	Primary/health/social care<br>Hospital (outpatient)<br>Secondary health/social care (not hospital)<br>Patient home	Patient adherence<br>Decision to stop treatment: unsupported

For peer review only - http://bmjopen.bmj.com/site/about/guidelines.xhtml

Study (First author, date)	Medications of interest	Other medications included	Perspectives obtained	Phenomenon of Interest	Setting/Context	Relevant part of care pathway
Kinnear, 2019	Statins	NA	Patient<br>Carer<br>Family member	Experiences/views of an intervention (adherence)<br>Reasons for adherence/non-adherence	Primary/health/social care<br>Secondary health/social care (not hospital)	Initial assessment<br>Information and/or advice<br>Choosing a medication<br>Shared decision making<br>Patient adherence<br>Progress review<br>Decision to stop treatment: unsupported
Reeve, 2013	Antihypertensives	Any long-term medication, methadone (substitution program), antiepileptic's, HRT, PPIs, donepezil, benzodiazepines antidepressants	Patient<br>Carer	Experiences/views of an intervention (deprescribing)	NR	Decision to stop treatment: supported
Dhar, 2017	Antihypertensives	NA	Patient	Reasons for adherence/non-adherence	Primary/health/social care<br>Secondary health/social care (not hospital)<br>Hospital (outpatient)<br>Patient home	Patient adherence<br>Decision to stop treatment: unsupported

For peer review only - http://bmjopen.bmj.com/site/about/guidelines.xhtml

Study (First author, date)	Medications of interest	Other medications included	Perspectives obtained	Phenomenon of Interest	Setting/Context	Relevant part of care pathway
Marshall, 2012	Antihypertensives	NA	Patient	Reasons for adherence/non-adherence	Primary/health/social care<br>Hospital (outpatient)<br>Patient home	Choosing a medication<br>Decision to start agreed treatment plan<br>Patient adherence<br>Decision to stop treatment: supported<br>Decision to stop treatment: unsupported
Khatib, 2014	Antihypertensives	NA	Practitioner/Prescriber<br>Patient	Prescribing<br>Of an intervention (adherence)<br>Reasons for adherence/non-adherence	Primary/health/social care<br>Hospital (outpatient)<br>Patient home	Identifying health or prescribing concern<br>Accessing care<br>Initial assessment<br>Information and/or advice<br>Choosing a medication<br>Shared decision making<br>Prescribing guidelines<br>Decision to start agreed Patient adherence<br>Progress review

For peer review only - http://bmjopen.bmj.com/site/about/guidelines.xhtml

Study (First author, date)	Medications of interest	Other medications included	Perspectives obtained	Phenomenon of Interest	Setting/Context	Relevant part of care pathway
Oori, 2019	Antihypertensives	NA	Patient	Reasons for adherence/non-adherence	Care home<br>Primary/health/social care-<br>Secondary health/social care (not hospital)<br>Hospital (outpatient)<br>Patient home	Patient adherence

For peer review only - http://bmjopen.bmj.com/site/about/guidelines.xhtml

Study (First author, date)	Medications of interest	Other medications included	Perspectives obtained	Phenomenon of Interest	Setting/Context	Relevant part of care pathway
Rashid, 2018	Antihypertensives	Yes, relevant to the following conditions: asthma, renal transplant, proton pump inhibitors, schizophrenia, migraine, antiretroviral for HIV, osteoporosis, anxiety, malaria prophylaxis, bipolar, back pain, ADHD, dysphagia, IBD, RA, breast cancer, anticoagulants, borderline personality disorder,<br>Specific drugs mentioned: clozapine, sumatriptan, SSRI, HAART, malaria prophylaxis, antibiotics, phosphate binding, insulin, clopidogrel, PMTCT, chemotherapy, Aripiprazole, Antidepressants	Practitioner/Prescriber<br>Patient<br>Carer<br>Family member	Experiences/views of an intervention (adherence)<br>Taking a medication	Primary/health/social care<br>Secondary health/social care (not hospital)<br>Hospital (inpatient)<br>Hospital (outpatient)	Accessing care<br>Initial assessment<br>Information and/or advice<br>Patient adherence<br>Progress review

* Data too thin for synthesis. Green highlighted text=High Overall Quality as appraised by AMSTAR-2, Turquoise highlighted text=Moderate Overall Quality as appraised by AMSTAR-2.
ADHD=Attention-Deficit Hyperactivity Disorder, BZ=Benzodiazepine, HAART= Highly Active Antiretroviral Therapy, HIV= Human Immunodeficiency Virus, IBD=Irritable Bowel Disease, NA=Not Applicable, NR=Not Reported, PMTCT=Prevention of Mother-to-Child Transmission, RA=Rheumatoid Arthritis, SSRI=Selective Serotonin Reuptake Inhibitor

For peer review only - http://bmjopen.bmj.com/site/about/guidelines.xhtml

APPENDIX E: QUALITY APPRAISAL OF INCLUDED STUDIES

Table 8: Scores on CEESAT items included studies - original search

First Author, Date	Search strategy: Is approach to searching clearly defined, systematic and transparent?	Is search comprehensive?	Does the review critically appraise each study?	During critical appraisal was an effort made to minimise subjectivity?	Total Score
Dills, 2018	Green	Green	Green	Green	0
Hukins, 2019	Green	Green	Green	Green	0
Pantoja, 2019	Green	Green	Green	Green	0
Posadzki, 2016	Green	Green	Green	Green	0
Stacey, 2017	Green	Green	Green	Green	0
Adler, 2017	Green	Green	Green	Green	0
de Cates, 2014	Green	Green	Green	Green	0
Fletcher, 2015	Green	Green	Green	Green	0
Jeffery, 2015	Green	Green	Green	Green	0
Kinnear, 2019	Green	Green	Green	Green	0
Legare, 2010	Green	Green	Green	Green	0
Maimaris, 2013	Green	Green	Green	Green	0
Mallat, 2016	Green	Green	Green	Green	0
Morrissey, 2017	Green	Green	Green	Green	0
Nieuwlaat, 2014	Green	Green	Green	Green	0
Palmer, 2018	Green	Green	Green	Green	0
Sanders, 2011	Green	Green	Green	Green	0
Shanbhag, 2018	Green	Green	Green	Green	0
van Driel, 2016	Green	Green	Green	Green	0
King, 2018	Green	Green	Amber	Green	1
Milosavljevic, 2018	Green	Green	Amber	Green	1
Page, 2016	Green	Green	Amber	Green	1
Park, 2017	Green	Green	Amber	Green	1
Cheema, 2014	Green	Green	Amber	Green	1

For peer review only - http://bmjopen.bmj.com/site/about/guidelines.xhtml

Conn, 2015[38]	Amber	Green	Green	Green	1
Conn, 2015[40]	Amber	Green	Green	Green	1
Conn, 2016	Amber	Green	Green	Green	1
de Simoni, 2013	Green	Amber	Green	Green	1
Ingersgaard, 2020	Green	Amber	Green	Green	1
Kassavou, 2018	Green	Green	Green	Red	1
Krack, 2019	Green	Green	Green	Red	1
Kronish, 2011	Green	Green	Amber	Green	1
Lawrence, 2015	Green	Green	Green	Red	1
Mann, 2014	Green	Green	Green	Red	1
Nguyen, 2018	Green	Amber	Green	Green	1
Rash, 2016	Green	Green	Green	Red	1
Reeve,s 2020	Amber	Green	Green	Green	1
Loganathan, 2011	Green	Amber	Amber	Green	2
Mamudu, 2014	Green	Green	Red	Red	2
Oori, 2019	Amber	Green	Amber	Green	2
Xu, 2018	Green	Amber	Amber	Green	2
Checchi, 2014	Green	Amber	Green	Red	2
Demonceau, 2013	Green	Amber	Amber	Green	2
Rashid, 2018	Green	Amber	Amber	Green	2
Reeve, 2013	Green	Green	Amber	Red	2
Baumgartner, 2020	Green	Amber	Green	Red	2
Bochkareva, 2019	Green	Green	Amber	Red	2
Deichmann, 2016	Amber	Green	Amber	Green	2
Khatib, 2014	Green	Amber	Green	Red	2
Laba, 2013	Green	Green	Amber	Red	2
Rashid, 2014	Green	Amber	Amber	Green	2
Tsioufis, 2020	Green	Amber	Green	Red	2
Uhlig, 2013	Green	Amber	Amber	Green	2
Yuan, 2019	Amber	Amber	Green	Green	2
Hart, 2020	Amber	Amber	Amber	Green	3
Polinski, 2011	Amber	Amber	Amber	Green	3
Al Alshaikh, 2016	Amber	Amber	Amber	Green	3
Conn, 2015	Amber	Green	Amber	Red	3
Dhar, 2017	Amber	Green	Amber	Red	3
Gupta, 2010	Amber	Amber	Amber	Green	3

Guzman-Tordecilla, 2020	Amber	Amber	Green	Red	3
Kang, 2016	Amber	Amber	Amber	Green	3
Marshall, 2012	Green	Amber	Amber	Red	3
Xiong, 2018	Green	Amber	Amber	Red	3
Du,2019	Amber	Amber	Amber	Red	4
Chhina, 2013.	Amber	Amber	Amber	Red	4
Kawalec, 2018	Amber	Amber	Amber	Red	4
Leslie, 2016[7]	Amber	Amber	Amber	Red	4

For peer review only

Table 9: Scores on AMSTAR-2 tool for reviews included in map with CEESAT score of 0

Study (First author, date)	1. PICO components	2. Protocol	3. Study design explanation	4. Comprehensive search strategy	5. Duplicate study selection	6. Duplicate data extraction	7. Details of excluded studies	8. Description of included studies	9b. RoB assessment (NRSIs)	10. Funding sources	9a. Risk of Bias (RoB) assessment (RCTs)	11a. RCTs Meta-analysis	11b. NRSIs Meta-analysis (MA)	12. MA: RoB in individual studies	13. Qualitative reviews: Is synthesis methodology /theoretical framework clearly described /justified?	14. Qualitative reviews: was description /rationale for sampling described?	15. Qualitative reviews: was process of data extraction clearly described?	16. Qualitative reviews: was process for coding data clearly described?	17. Qualitative reviews: was process of developing themes clearly described?	18. Qualitative reviews: Do authors reflect on how their theoretical /ideological perspectives may influence their findings?	19 Qualitative reviews: Was use of any software clearly described?	20. RoB: discussion of results	21. Heterogeneity	22. Publication bias	23. Reports conflicts of interest	Overall rating
Adler, 2017	Y	Y	N	Y	Y	Y	Y	Y	NA	Y	Y	N	NA	NA	NA	NA	NA	NA	NA	NA	NA	Y	Y	NA	Y	H
de Cates, 2014	Y	PY	N	Y	Y	Y	Y	Y	NA	N	Y	Y	NA	Y	NA	NA	NA	NA	NA	NA	NA	Y	Y	Y	Y	H
Dills 2018,	Y	N	N	Y	Y	Y	N	Y	NA	Y	Y	N	NA	NA	NA	NA	NA	NA	NA	NA	NA	Y	Y	NA	Y	L
Fletcher, 2015	Y	Y	Y	Y	Y	Y	N	PY	N	N	Y	Y	N	Y	NA	NA	NA	NA	NA	NA	NA	Y	Y	N	Y	CL
Hukins, 2019	Y	N	N	Y	Y	Y	N	Y	Y	N	NA	NA	N	NA	NA	NA	NA	NA	NA	NA	NA	Y	Y	NA	Y	L
Jeffery, 2015	Y	Y	N	Y	Y	Y	N	Y	Y	N	Y	Y	Y	Y	NA	NA	NA	NA	NA	NA	NA	Y	Y	N	Y	M
Kinnear, 2019	Y	Y	NA	Y	Y	Y	N	Y	Y	Y	NA	NA	N	NA	PY	NA	Y	Y	Y	N	Y	Y	Y	NA	Y	H
Legare, 2010	Y	Y	N	Y	Y	Y	Y	Y	NA	N	Y	N	NA	NA	NA	NA	NA	NA	NA	NA	NA	Y	Y	NA	Y	H
Maimaris, 2013	Y	Y	Y	Y	Y	Y	N	PY	PY	N	Y	N	N	NA	NA	NA	NA	NA	NA	NA	NA	Y	Y	NA	Y	M
Mallat, 2016	Y	Y	N	Y	Y	Y	N	Y	NA	Y	Y	Y	NA	Y	NA	NA	NA	NA	NA	NA	NA	Y	Y	Y	Y	H
Morrissey, 2017	Y	Y	N	Y	Y	Y	N	Y	NA	N	Y	N	NA	Y	NA	NA	NA	NA	NA	NA	NA	Y	Y	Y	Y	L

For peer review only - http://bmjopen.bmj.com/site/about/guidelines.xhtml

Study (First author, date)	1. PICO components	2. Protocol	3. Study design explanation	4. Comprehensive search strategy	5. Duplicate study selection	6. Duplicate data extraction	7. Details of excluded studies	8. Description of included studies	9b. RoB assessment (NRSIs)	10. Funding sources	9a. Risk of Bias (RoB) assessment (RCTs)	11a. RCTs Meta-analysis	11b. NRSIs Meta-analysis (MA)	12. MA: RoB in individual studies	13. Qualitative reviews: Is synthesis methodology /theoretical framework clearly described /justified?	14. Qualitative reviews: was description /rationale for sampling described?	15. Qualitative reviews: was process of data extraction clearly described?	16. Qualitative reviews: was process for coding data clearly described?	17. Qualitative reviews: was process of developing themes clearly described?	18. Qualitative reviews: Do authors reflect on how their theoretical /ideological perspectives may influence their findings?	19 Qualitative reviews: Was use of any software clearly described?	20. RoB: discussion of results	21. Heterogeneity	22. Publication bias	23. Reports conflicts of interest	Overall rating
Nieuwlaat, 2014	Y	Y	N	Y	Y	Y	Y	Y	NA	N	Y	N	NA	NA	NA	NA	NA	NA	NA	NA	NA	Y	Y	NA	Y	M
Palmer, 2018	Y	Y	Y	Y	Y	Y	Y	Y	NA	Y	Y	N	NA	NA	NA	NA	NA	NA	NA	NA	NA	Y	Y	NA	Y	H
Pantoja, 2019	Y	Y	N	Y	Y	Y	Y	Y	Y	Y	Y	N	N	NA	NA	NA	NA	NA	NA	NA	NA	Y	Y	NA	Y	H
Posadzki, 2016	Y	Y	Y	Y	Y	Y	Y	Y	Y	Y	Y	Y	Y	Y	NA	NA	NA	NA	NA	NA	NA	Y	Y	N	Y	H
Sanders, 2011	Y	N	N	Y	Y	Y	Y	Y	PY	Y	PY	Y	Y	N	NA	NA	NA	NA	NA	NA	NA	N	Y	N	Y	CL
Shanbhag, 2018	Y	Y	N	Y	Y	Y	N	PY	Y	N	Y	N	N	NA	NA	NA	NA	NA	NA	NA	NA	N	Y	NA	Y	L
Stacey, 2017	Y	Y	N	Y	Y	Y	Y	Y	NA	N	Y	Y	NA	Y	NA	NA	NA	NA	NA	NA	NA	Y	Y	Y	Y	M
van Driel, 2016	Y	Y	N	Y	Y	Y	Y	Y	NA	Y	Y	Y	NA	Y	NA	NA	NA	NA	NA	NA	NA	Y	Y	Y	Y	H

CL=Critically Low overall quality; H=High overall quality; Low overall quality; M=Moderate overall quality; N=No; NA=Not Applicable; NRSI=Non-Randomised Studies of healthcare Interventions; PICO=Population, Intervention, Comparator, Outcome; PY=Partial Yes; RCT=Randomised Controlled Trial; RoB=Risk of Bias; Y=Yes

For peer review only

For peer review only - http://bmjopen.bmj.com/site/about/guidelines.xhtml

PRISMA 2020 Checklist

Section and Topic	Item #	Checklist item	Location where item is reported
**TITLE**			
Title	1	Identify the report as a systematic review.	1
**ABSTRACT**			
Abstract	2	See the PRISMA 2020 for Abstracts checklist.	2
**INTRODUCTION**			
Rationale	3	Describe the rationale for the review in the context of existing knowledge.	4
Objectives	4	Provide an explicit statement of the objective(s) or question(s) the review addresses.	4
**METHODS**			
Eligibility criteria	5	Specify the inclusion and exclusion criteria for the review and how studies were grouped for the syntheses.	7-8
Information sources	6	Specify all databases, registers, websites, organisations, reference lists and other sources searched or consulted to identify studies. Specify the date when each source was last searched or consulted.	6
Search strategy	7	Present the full search strategies for all databases, registers and websites, including any filters and limits used.	Supplementary Materials
Selection process	8	Specify the methods used to decide whether a study met the inclusion criteria of the review, including how many reviewers screened each record and each report retrieved, whether they worked independently, and if applicable, details of automation tools used in the process.	6-7
Data collection process	9	Specify the methods used to collect data from reports, including how many reviewers collected data from each report, whether they worked independently, any processes for obtaining or confirming data from study investigators, and if applicable, details of automation tools used in the process.	7-8
Data items	10a	List and define all outcomes for which data were sought. Specify whether all results that were compatible with each outcome domain in each study were sought (e.g. for all measures, time points, analyses), and if not, the methods used to decide which results to collect.	8
	10b	List and define all other variables for which data were sought (e.g. participant and intervention characteristics, funding sources). Describe any assumptions made about any missing or unclear information.	8
Study risk of bias assessment	11	Specify the methods used to assess risk of bias in the included studies, including details of the tool(s) used, how many reviewers assessed each study and whether they worked independently, and if applicable, details of automation tools used in the process.	7-8
Effect measures	12	Specify for each outcome the effect measure(s) (e.g. risk ratio, mean difference) used in the synthesis or presentation of results.	8
Synthesis methods	13a	Describe the processes used to decide which studies were eligible for each synthesis (e.g. tabulating the study intervention characteristics and comparing against the planned groups for each synthesis (item #5)).	8-9
	13b	Describe any methods required to prepare the data for presentation or synthesis, such as handling of missing summary statistics, or data conversions.	na
	13c	Describe any methods used to tabulate or visually display results of individual studies and syntheses.	8
	13d	Describe any methods used to synthesize results and provide a rationale for the choice(s). If meta-analysis was performed, describe the model(s), method(s) to identify the presence and extent of statistical heterogeneity, and software package(s) used.	na
	13e	Describe any methods used to explore possible causes of heterogeneity among study results (e.g. subgroup analysis, meta-regression).	na
	13f	Describe any sensitivity analyses conducted to assess robustness of the synthesized results.	na
Reporting bias assessment	14	Describe any methods used to assess risk of bias due to missing results in a synthesis (arising from reporting biases).	na

For peer review only

PRISMA 2020 Checklist

Section and Topic	Item #	Checklist item	Location where item is reported
Certainty assessment	15	Describe any methods used to assess certainty (or confidence) in the body of evidence for an outcome.	na
**RESULTS**			
Study selection	16a	Describe the results of the search and selection process, from the number of records identified in the search to the number of studies included in the review, ideally using a flow diagram.	10
	16b	Cite studies that might appear to meet the inclusion criteria, but which were excluded, and explain why they were excluded.	Supplementary Materials
Study characteristics	17	Cite each included study and present its characteristics.	10-13
Risk of bias in studies	18	Present assessments of risk of bias for each included study.	10, Supplementary Materials
Results of individual studies	19	For all outcomes, present, for each study: (a) summary statistics for each group (where appropriate) and (b) an effect estimate and its precision (e.g. confidence/credible interval), ideally using structured tables or plots.	NA
Results of syntheses	20a	For each synthesis, briefly summarise the characteristics and risk of bias among contributing studies.	Supplementary Materials, 10-13
	20b	Present results of all statistical syntheses conducted. If meta-analysis was done, present for each the summary estimate and its precision (e.g. confidence/credible interval) and measures of statistical heterogeneity. If comparing groups, describe the direction of the effect.	na
	20c	Present results of all investigations of possible causes of heterogeneity among study results.	na
	20d	Present results of all sensitivity analyses conducted to assess the robustness of the synthesized results.	na
Reporting biases	21	Present assessments of risk of bias due to missing results (arising from reporting biases) for each synthesis assessed.	na
Certainty of evidence	22	Present assessments of certainty (or confidence) in the body of evidence for each outcome assessed.	na
**DISCUSSION**			
Discussion	23a	Provide a general interpretation of the results in the context of other evidence.	na
	23b	Discuss any limitations of the evidence included in the review.	14
	23c	Discuss any limitations of the review processes used.	14
	23d	Discuss implications of the results for practice, policy, and future research.	14-15
**OTHER INFORMATION**			
Registration and protocol	24a	Provide registration information for the review, including register name and registration number, or state that the review was not registered.	4
	24b	Indicate where the review protocol can be accessed, or state that a protocol was not prepared.	4
	24c	Describe and explain any amendments to information provided at registration or in the protocol.	8
Support	25	Describe sources of financial or non-financial support for the review, and the role of the funders or sponsors in the review.	1
Competing interests	26	Declare any competing interests of review authors.	1

For peer review only - http://bmjopen.bmj.com/site/about/guidelines.xhtml

PRISMA 2020 Checklist

Section and Topic	Item #	Checklist item	Location where item is reported
Availability of data, code and other materials	27	Report which of the following are publicly available and where they can be found: template data collection forms; data extracted from included studies; data used for all analyses; analytic code; any other materials used in the review.	1

From: Page MJ, McKenzie JE, Bossuyt PM, Boutron I, Hoffmann TC, Mulrow CD, et al. The PRISMA 2020 statement: an updated guideline for reporting systematic reviews. BMJ 2021;372:n71. doi: 10.1136/bmj.n71

For more information, visit: http://www.prisma-statement.org/

For peer review only

BMJ Open

What is the quantity, quality and type of systematic review evidence available to inform the optimal prescribing of statins and antihypertensives? A systematic umbrella review and evidence and gap map

Journal:	*BMJ Open*
Manuscript ID	bmjopen-2023-072502.R1
Article Type:	Original research
Date Submitted by the Author:	04-Jul-2023
Complete List of Authors:	Shaw, Liz; University of Exeter, Exeter Policy Research Programme Evidence Review Facility<br>Briscoe, Simon; University of Exeter, Exeter Policy Research Programme Evidence Review Facility<br>Nunns, Michael; University of Exeter, Exeter Policy Research Programme Evidence Review Facility<br>Lawal, Hassanat; University of Exeter, Exeter Policy Research Programme Evidence Review Facility<br>Melendez-Torres, G.J; University of Exeter, Exeter Policy Research Programme Evidence Review Facility<br>Turner, Malcolm; University of Exeter, Exeter Policy Research Programme Evidence Review Facility; University of Exeter, NIHR ARC South West Peninsula Patient and Public Engagement group<br>Garside, Ruth ; University of Exeter, Exeter Policy Research Programme Evidence Review Facility; University of Exeter, European Centre for Environment and Health<br>Thompson Coon, Jo; University of Exeter, Exeter Policy Research Programme Evidence Review Facility
**Primary Subject Heading**:	Cardiovascular medicine
Secondary Subject Heading:	Cardiovascular medicine, Evidence based practice, Global health, Pharmacology and therapeutics
Keywords:	Systematic Review, Hypertension < CARDIOLOGY, STROKE MEDICINE

SCHOLARONE™
Manuscripts

I, the Submitting Author has the right to grant and does grant on behalf of all authors of the Work (as defined in the below author licence), an exclusive licence and/or a non-exclusive licence for contributions from authors who are: i) UK Crown employees; ii) where BMJ has agreed a CC-BY licence shall apply, and/or iii) in accordance with the terms applicable for US Federal Government officers or employees acting as part of their official duties; on a worldwide, perpetual, irrevocable, royalty-free basis to BMJ Publishing Group Ltd ("BMJ") its licensees and where the relevant Journal is co-owned by BMJ to the co-owners of the Journal, to publish the Work in this journal and any other BMJ products and to exploit all rights, as set out in our licence.

The Submitting Author accepts and understands that any supply made under these terms is made by BMJ to the Submitting Author unless you are acting as an employee on behalf of your employer or a postgraduate student of an affiliated institution which is paying any applicable article publishing charge ("APC") for Open Access articles. Where the Submitting Author wishes to make the Work available on an Open Access basis (and intends to pay the relevant APC), the terms of reuse of such Open Access shall be governed by a Creative Commons licence – details of these licences and which Creative Commons licence will apply to this Work are set out in our licence referred to above.

Other than as permitted in any relevant BMJ Author's Self Archiving Policies, I confirm this Work has not been accepted for publication elsewhere, is not being considered for publication elsewhere and does not duplicate material already published. I confirm all authors consent to publication of this Work and authorise the granting of this licence.

For peer review only

What is the quantity, quality and type of systematic review evidence available to inform the optimal prescribing of statins and antihypertensives? A systematic umbrella review and evidence and gap map

Liz Shaw[1*], Simon Briscoe[1], Michael Nunns[1], Hassanat Lawal[1], G.J. Melendez-Torres[1], Malcolm Turner[1,2], Ruth Garside[1,3], Jo Thompson Coon[1],

[1]Exeter Policy Research Programme Evidence Review Facility, Faculty of Health and Life Sciences, St Lukes Campus, University of Exeter, Exeter, Devon, EX1 2LU, UK

[2] NIHR ARC South West Peninsula Patient and Public Engagement group, Faculty of Health and Life Sciences, St Lukes Campus, University of Exeter, Exeter, Devon, EX1 2LU

[3] European Centre for Environment and Health, Faculty of Health and Life Sciences, Knowledge Spa, Truro, TR1 3HD

*Corresponding author: E.H.Shaw@exeter.ac.uk, 01392 72 2396

Keywords: Statins, antihypertensives, mapping review

Word count (Excluding abstract, strengths/limitations, tables, figures, reference and appendices): 3254

Number of Figures: 3

Number of Tables: 2

For peer review only

ABSTRACT

Objectives: We aimed to map the systematic review evidence available to inform the optimal prescribing of statins and antihypertensive medication.

Design: Systematic umbrella review and evidence and gap map (EGM).

Data sources: Eight bibliographic databases (Cochrane Database of Systematic Reviews, CINAHL, EMBASE, Health Management Information Consortium, MEDLINE ALL, PsychInfo, Conference Proceedings Citation Index – Science and Science Citation Index) were searched from 2010 to 11th August 2020. Update searches conducted in MEDLINE ALL 2nd August 2022. We searched relevant websites and conducted backwards citation chasing.

Eligibility criteria for selecting studies: We sought systematic reviews of quantitative or qualitative research where adults 16 years+ were currently receiving, or being considered for, a prescription of statin or anti-hypertensive medication. Eligibility criteria were applied to the title and abstract and full text of each article independently by two reviewers.

Data extraction and synthesis: Quality appraisal was completed by one reviewer and checked by a second. Review characteristics were tabulated and incorporated into an EGM based on a patient care pathway. Patients with lived experience provided feedback on our research questions and evidence and gap map.

Results: Sixty-eight reviews were included within the EGM. The highest quantity of evidence focused on evaluating interventions to promote patient adherence to antihypertensive medication. Key gaps included a lack of reviews synthesising evidence on experiences of specific interventions to promote patient adherence or improve prescribing practice. The evidence was predominantly of low quality, limiting confidence in the findings from individual reviews.

Conclusions: This EGM provides an interactive, accessible format for policy developers, service commissioners and clinicians to view the systematic review evidence available relevant to optimising the prescribing of statin and antihypertensive medication. To address the paucity of high-quality research, future reviews should be conducted and reported according to existing guidelines and address the evidence gaps identified above.

For peer review only - http://bmjopen.bmj.com/site/about/guidelines.xhtml

STRENGTHS AND LIMITATIONS

- Our evidence and gap map (EGM) represents the results of a comprehensive, systematic search for systematic review evidence in this field.
- The evidence and gap map includes only systematic review evidence and thus there may be primary research relevant to the topic which may sit within the identified gaps.
- Our search strategy did not capture systematic reviews which may have met our eligibility criteria that were used to inform NICE guidelines relevant to the review topic. These reviews were not retrieved by our bibliographic database searches, thus highlighting issues regarding the lack of visibility of the reviews contributing to NICE guidelines.

For peer review only

For peer review only - http://bmjopen.bmj.com/site/about/guidelines.xhtml

INTRODUCTION

With an estimated 17.9 million deaths during 2019, cardiovascular disease (CVD) is the leading cause of death worldwide.[1] One study estimates the total costs of heart failure for 98.7% of the world's population to be $108 billion dollars.,[2] with individuals from black, Asian and other minority ethnic backgrounds, low socio-economic status or those living with diabetes or severe mental illness being more at risk of the disease.[3] Along with earlier detection of risk factors for CVD and promotion of lifestyle changes, high intensity statins are recommended over the use of low intensity statins as an effective treatment to prevent CVD.[4] Whilst a key target set by the World Health Organisation that a minimum 50% of eligible individuals will be in receipt of statins has been met in high-income countries,[5] the prescribing of this type of medication in the UK has not yet reached optimal levels.[4]

Hypertension is another risk factor in the development of CVD,[6] and can be controlled through use of antihypertensive medication, sometimes in combination with statins, to reduce the occurrence of CVD.[7] Whilst, the rate of hypertension remained largely unchanged between 1990 and 2019, the burden of illness shifted from high to middle- and low-income countries.[8] There are early signs that awareness and treatment of hypertension within some high-income countries, such as the UK, has decreased within the last five years.[9, 10] Patient non-adherence is also an issue of concern, with adherence decreasing with higher numbers of antihypertensive medications prescribed per patient.[11]

To achieve optimal prescribing of statins and antihypertensive medication, it is essential that the factors influencing both their prescription and patient adherence are fully understood. There is already a diverse range of existing systematic review evidence in this area which could be utilised by policy makers, commissioners and clinicians to design and deliver services or research to support the needs of those living with, or at risk of, CVD. To fully understand and make use of this existing evidence base, this paper focuses on the creation of an evidence and gap map to display the quantity, quality and type of quantitative and qualitative systematic review evidence available to inform the optimal prescribing of statins and antihypertensives.

We were interested in systematic reviews which synthesised the following:

- Evidence regarding the effectiveness or experiences of interventions intended to improve prescribing practices or patient adherence;
- Evidence on the effectiveness or experiences of interventions intended to improve implementation of interventions intended to improve prescribing practices or patient adherence;

For peer review only - http://bmjopen.bmj.com/site/about/guidelines.xhtml

- Evidence focusing on practitioner views or perceptions of making prescribing decisions;
- Guidelines intended to inform prescribing practise.

This paper is part of a broader systematic mapping review commissioned by NHS England and Improvement relating to the optimising of the prescribing of three different groups of medication: medications to treat cardiovascular disease, DCD (including opioids, benzodiazepines, gabapentinoids and non-benzodiazepine hypnotics) and antidepressants.[12]

For peer review only

For peer review only - http://bmjopen.bmj.com/site/about/guidelines.xhtml

METHODS

The methods used for the searching, screening, data extraction and quality appraisal processes of the evidence included in this evidence and gap map were in accordance with the University of York's Centre for Reviews and Dissemination guidelines.[13] We also consulted guidance for the development of evidence and gap maps.[14] Our methods were outlined in our pre-registered review protocol and are summarised below according to PRISMA reporting guidance.[15, 16]

Search strategy

The first stage of our systematic umbrella review involved conducting searches of bibliographic databases and websites for relevant literature. Our bibliographic database search strategy combined terms for optimising prescribing with terms for statins and antihypertensives and CVD. The search strategy was developed using MEDLINE (via Ovid) using a combination of free-text terms and controlled headings. Search terms were derived from pre-identified relevant papers and inspected by stakeholders with expertise on optimising prescribing. Searches were run on 11th August 2020 in the following bibliographic databases: Cochrane Database of Systematic Reviews (via Cochrane Library), CINAHL (via EBSCO), EMBASE (via Ovid), Health Management Information Consortium (via Ovid), MEDLINE ALL (via Ovid), PsychInfo (via Ovid), Conference Proceedings Citation Index – Science and Science Citation Index (both via Web of Science, Clarivate Analytics). We also searched systematic review database Epistemonikos and preprint server medRxiv. An historical date limit of 2010 was applied to all search results. We carried out backwards citation chasing for all reviews which met our inclusion criteria, searched topically relevant websites and pursued full texts for conference abstracts and review protocols identified through our searches. We also contacted review authors where we could not access full-texts. Update searches were conducted in MEDLINE ALL (via OVID) on 2nd August 2022. We limited our update search to MEDLINE since all the studies identified through our original search were indexed in this database. Full details of database search strategies are reproduced in Appendix A (which also includes search terms for drugs that cause dependency, which was a second topic that our client asked us to investigate in our mapping review. The findings associated with this second topic are available elsewhere).

Study selection

After running our search strategy, the second stage of our umbrella review involved two reviewers (LS, MN)screening a sample of 100 title and abstracts to ascertain the utility of our inclusion criteria for this review. The revised inclusion criteria (Table 1), were then applied independently by two reviewers to each citation retrieved via our search strategy (LS, MN), with disagreements resolved through discussion or referral to a third reviewer (SB). Eligibility of full texts were assessed using the

For peer review only - http://bmjopen.bmj.com/site/about/guidelines.xhtml

same method. Screening decisions were recorded in Endnote X8 software (Clarivate Analytics, Philadephia, PA, USA) and the study selection process detailed within a PRISMA-style flowchart.

Table 1: Review inclusion criteria

PICO/PICo criteria	Inclusion criteria
Population	Mean age ≥ 16<br>Receiving/being considered for a prescription for statins and/or antihypertensives.
Intervention/Phenomenon of interest	For systematic reviews of quantitative evidence, interventions which aimed to improve one or more of the following:<br>- Adherence to prescribed medication of interest;<br>- Prescriber adherence to clinical guidance for prescribing;<br>- Prescriber practices;<br>- Implementation of an intervention to enhance patient adherence or prescriber practices.<br><br>Interventions could be conducted at the level of system or that of the patient/prescriber.<br><br>For systematic reviews of qualitative evidence, review foci could include the views or experiences of patients, carers or clinicians for:<br>- Healthcare consultations to discuss initiation, reviewing or discontinuing a prescription;<br>- Interventions to improve adherence*/prescribing practice;<br>- Reasons for adherence or non-adherence to prescribed medication;<br>- Making prescribing decisions.
Comparator/Context	Any
Outcome	Relevant to the aims of our review and stated clearly in the study abstract.<br><br>For reviews of quantitative evidence, examples may include:<br>- Measures of patient adherence;<br>- Measures of prescriber adherence to prescribing guidelines;<br>- Measures of success of implementing interventions to promote adherence or prescribing.<br><br>For reviews of qualitative evidence, please see phenomenon of interest section above.
Study design	Systematic reviews which met the following criteria:<br>- Clearly stated research question;<br>- Indicate which sources were searched;<br>- Reproducible search strategy and search date;<br>- Clearly defined inclusion and exclusion criteria and study selection methods;<br>- Critically appraise and report the quality of included studies;<br>- Reproducible data synthesis strategy.

For peer review only - http://bmjopen.bmj.com/site/about/guidelines.xhtml

	Encompasses: systematic reviews of quantitative and qualitative literature, systematic reviews of reviews, systematic reviews of guidelines, scoping and rapid reviews.
Date limit	Systematic reviews published after 2010 were excluded. This was to prioritise the most recent systematic reviews, evaluating interventions and experiences most relevant to current medical settings, for inclusion in the EGM

*For convenience, we use the term "patient adherence" throughout this report to encapsulate this continual decision-making process in which the patient can choose, and/or be enabled, to play an active role when taking their medication as prescribed.

Data extraction and quality appraisal

After title and abstract and full-text screening of the articles identified via our search strategy, two reviewers carried out a pilot of our data extraction form on five reviews. The finalised data extraction and quality appraisal form was applied to all included systematic reviews by one reviewer (MN, LS) and checked by a second (SB, LS, MN), supported by EPPI-Reviewer (v 4.11.5.2) software. Disagreements were resolved through discussion or referral to a third person.

We THENconducted quality appraisal in two stages. Reviews were first appraised using four modified criteria from the Collaboration for Environmental Evidence Synthesis Assessment Tool (CEESAT):[17]

1. Search strategy: Is the approach to searching clearly defined, systematic and transparent?
2. Is the search comprehensive?
3. Does the review critically appraise each study?
4. During critical appraisal was an effort made to minimise subjectivity?

Only systematic reviews which met all four criteria were prioritised for full quality appraisal using the AMSTAR-2,[18] modified to incorporate reporting standards for qualitative evidence synthesis.[19] Reviews appraised using only the CEESAT criteria were given an overall quality rating of 'Critically-Low". This process of quality appraisal represents a deviation from our protocol and was necessary due to the high number of studies eligible for inclusion in the EGM. Thus, we decided to utilise the process described above to priories the inclusion of the most robust evidence for inclusion in the EGM.

Full details of the data extracted from each review, including CEESAT and AMSTAR-2 criteria, are provided elsewhere.[12]

For peer review only - http://bmjopen.bmj.com/site/about/guidelines.xhtml

Data analysis and presentation

In the final stage of our umbrella review, details of the systematic reviews prioritised for inclusion in the evidence and gap map were summarised in a table and described narratively. We used EPPI-Mapper software to structure the evidence and gap map according to medications of interest and patient care pathway.[20]

The initial care pathway was based upon NICE guidelines for statins and antihypertensives, optimising prescribing and shared decision making.[5, 21-23] To ensure the pathways reflected both recommended practice and implementation within 'real-life' settings, we also drew upon a systematic review exploring patient experiences of medication taking and consulted with patients and carers with experience of seeking, being prescribed, taking and/or discontinuing statins and/or antihypertensives.[24] We also asked clinicians if the pathway we developed reflected their clinical experience working within the area. Finally, we produced a condensed version of the map which focused on pre-treatment/initiation, maintenance, discontinuation and development of guidelines.

The map presents the systematic review evidence according to the 'Overall Quality' rating provided by the AMSTAR-2. The colour and size of the bubble indicate the quality rating and number of systematic reviews for that medication type at that part of the care pathway respectively. Systematic reviews relevant to more than one type of medication or position on the care pathway are represented more than once within the map. Filters also allow the map user to alter the type of evidence shown in the map. Policy, clinical and patient stakeholders were asked to comment on the presentation and content of the final evidence and gap map to ensure findings were accessible to our intended audiences.

Studies identified through update searches were only included in the map if they provided evidence for parts of the care pathway where there was insufficient evidence.

Patient and public involvement

We worked alongside a group of stakeholders with a variety of expertise in the topic area throughout the production of this evidence and gap map. Stakeholders included representatives from NHSE-I, NICE, clinicians and people with experience of taking one or more of the medications of interest. Key stages at which all stakeholders, including patients and members of the public, contributed during the project included; development of the protocol, including providing feedback on the appropriateness of review questions, supporting development of the bibliographic database search strategies and list of relevant topic websites to be searched. Clinical stakeholders and patients/members of the public also provided insight on the list of included studies, including

For peer review only - http://bmjopen.bmj.com/site/about/guidelines.xhtml

resolving queries regarding eligibility for inclusion, supported the development of the patient care-pathway and also provided feedback on the presentation of the evidence and gap map and gaps in systematic review evidence within the evidence and gap map and how findings should be disseminated.

For peer review only

For peer review only - http://bmjopen.bmj.com/site/about/guidelines.xhtml

RESULTS

Following deduplication, 1623 unique records were found through our bibliographic database and supplementary searches. 1429 of these were excluded during title and abstract screening, with a further 108 excluded after the reviewing of full-texts. Fourteen systematic reviews were identified through the search strategy intended to identify other medication groups as part of a wider piece of work (ref withheld for peer review). In total, 80 systematic reviews were eligible for inclusion in the review. The 68 studies identified via our original searches are presented within our evidence and gap map (access here, also shown in Figure 1). The 12 studies identified from our update searches related to parts of the care pathway where there was sufficient evidence and are therefore not included in the map but summarised in Appendix B. Figure 2 summarises the study selection process and reasons for exclusion at full-text, with a list of full reasons for exclusion provided in Appendix C.

[Insert Figure 1]

[Insert Figure 2]

Characteristics of evidence included within umbrella review

Medications of interest within the 80 reviews included in our umbrella review were: statins (n=9),[25-32, 33, 34] antihypertensives (n=37),[35-69, , 70,71] and statins and antihypertensives (n=32).[272-98, 99, 100, 101, 102, 103, 104] Nine of the reviews synthesised qualitative evidence,[27, 31, 41, 48, 53, 56, 57, 58, 81, 102] four of which also included quantitative evidence in a mixed evidence synthesis.[43, 48, 56, 58] Of the reviews synthesising quantitative evidence evaluating interventions, interventions focused on: enhancing patient adherence to medication as prescribed (n=57),[25, 30, 32, 33, 35-40, , 42-45, 47, 49, 51, 52, 54, 55, 59, 60, 62-70, 72-79, 84-92, 94, 96-98, 99-101, 34, 71,103] deprescribing a prescribed medication (n=3),[46, 80, 93] or optimising the prescribing of medication to meet the needs of the patient (n=13).[26, 28, 29, 37, 50, 61, 67, 82, 83, 85, 87, 95, 104] Three of these reviews were judged by reviewers to have dual aims.[67, 85, 87] An overview of the characteristics of all reviews included in the evidence and gap map is included in Appendix D.

Characteristics of evidence included in evidence and gap map

Nineteen of the 80 systematic reviews included in our umbrella review were fully appraised using the modified AMSTAR-2 tool Eight were ‘High’ quality,[27, 28, 52, 72, 79, 94, 95, 98] five were of ‘Moderate’ quality,[32, 51, 62, 82, 92] four of ‘Low’ quality,[26, 55, 61, 80] and two ‘Critically-Low’ quality.[43, 60] The remaining 49 reviews scored poorly on one or more of the CEESAT criteria during

For peer review only - http://bmjopen.bmj.com/site/about/guidelines.xhtml

the first stage of quality appraisal, and were thus awarded a ‘Critically-Low’ overall quality rating. The full quality appraisal ratings for each review can be found in Appendix E.

Table 2 provides a summary of the systematic review evidence included in the evidence and gap map.

Table 2: Summary of included reviews

**Topic/type of evaluation**	**Type of evidence**	**Number of reviews***	**Appraised using AMSTAR-2 Y/N: N (Overall Quality rating)**	**Medication of interest**
Prescriber, patient, and/or family/carer views of issues relating to prescribing and/or adherence to medications of interest	Qualitative	9*[1]	Y: 1<br>(High: 1)	Statins: 1
			N: 8	AH:6<br>Statins: 1<br>Statins+AH: 1
Evaluating intervention to optimise prescribing	Quantitative	12	Y: 5<br>(High 2:, Moderate:1, Low: 2)	AH: 1<br>Statins: 2<br>Statins+AH: 2
			N: 7	AH:3<br>Statins: 1<br>Statins+AH: 3
Evaluating intervention to deprescribe medication	Quantitative	3	Y: 1<br>(Critically low: 1)	Statins+AH: 1
			N: 2	AH: 1<br>Statins+AH: 1
Evaluating intervention to optimise patient adherence to a medication	Quantitative	47	Y: 12<br>(High: 6, Moderate: 3, Low: 1, Critically low: 2)	AH: 7<br>Statins: 1<br>Statins+AH: 4
			N: 35	AH: 17<br>Statins: 2<br>Statins+AH: 16

*n>68 as some reviews were coded as having multiple aims; *[1]Four reviews also included quantitative evidence; AH=Antihypertensives; N=No; Y=Yes

Summary of evidence on the patient care pathway

Figure 3 presents a visual summary of the 68 reviews included in the evidence and gap map, presented according to the volume of evidence available which corresponds to the different aims of interest to this umbrella review. Further detail is summarised below.

[Insert Figure 3]

Summary of evidence and gaps

Figure 3 illustrates a large cluster of evidence relating to the evaluation of interventions to promote maintaining adherence to medication, particularly to treat hypertension. The next biggest cluster of evidence is relevant to the ‘Pre-treatment’ or ‘Initiation’ stage of the pathway with antihypertensive

For peer review only

medication. However, overall the quality of the evidence at these segments of the care pathway was predominantly of critically-low quality.

The quantity of qualitative evidence available is low and, whilst the evidence examines the perceptions of patients, carers and/or clinicians of issues affecting medication prescription or adherence, none of the systematic reviews explore experiences of specific interventions intended to optimise prescribing in some way. Only one of the systematic reviews of qualitative evidence was of 'High' quality.

Pre-treatment/Initiation

Four reviews synthesised qualitative research.[27, 31,48, 53] One high quality review of qualitative evidence explored patient and family members views of the barriers and enablers to treatment adherence for heterozygous familial hypercholesterolaemia.[27] Three reviews of 'Critically Low' quality review synthesised qualitative evidence relevant to the prescribing of antihypertensives.[31, 48, 53] Two reviews included a range of different perspectives,[31, 48] whilst one focused on patient perspectives.[53]

Twenty-nine systematic reviews synthesised quantitative evidence to promote medication adherence. Statins were the medication of interest for nine reviews, two of 'High' overall quality,[79, 98] and seven of 'Critically Low' overall quality.[74, 85, 88, 90, 91, 96, 97] Antihypertensives were the medication of interest in twenty systematic reviews; three were of 'High' quality,[52, 79, 98] two of 'Moderate' overall quality,[51, 62] and fifteen were 'Critically Low' overall quality.[36, 42, 44, 47, 49, 54, 63, 67, 74, 85, 88, 91, 92, 96, 97] Eight reviews sat across both statin and antihypertensive care pathways.[74, 79, 85, 88, 90, 91, 97, 98]

Six systematic reviews synthesised quantitative evidence evaluating interventions to optimise prescribing, where the medication of interest was statins; 'High' quality (n=2),[28, 95] 'Moderate' quality (n=1),[82] and 'Critically Low' quality (n=3).[29, 83, 85] One 'Critically Low' quality review synthesised quantitative evidence evaluating interventions to reduce the prescribing of fall risk increasing drugs in older adults.[46]

Maintaining treatment

One high quality,27 and three 'Critically-low' quality,[31,48, 53] reviews of qualitative evidence, included in the 'Pre-treatment/initiation' part of pathway, were also relevant here. In addition, one 'Critically-Low' quality review explored reasons associated with adherence or non-adherence to statin medication,[58] and a further three 'Critically-Low' quality reviews combined quantitative and

For peer review only - http://bmjopen.bmj.com/site/about/guidelines.xhtml

qualitative data to explore factors relating to adherence/nonadherence where antihypertensives and/or statins were the medication of interest.[16, 24, 50]

Twenty-four systematic reviews synthesised quantitative evidence where statins were the medication of interest. Five were of 'High' overall quality,[2, 13, 52, 56, 67] one of 'Moderate' overall quality,[49] and 18 of 'Critically Low' overall quality synthesised quantitative evidence to promote adherence. Forty-four systematic reviews synthesised quantitative evidence evaluating interventions to promote adherence to medications, which included antihypertensives.[25, 30, —73-78, 84-91, 96, 97]

Five were of 'High' overall quality,[52, 72, 79, 94, 98] three were 'Moderate' quality,[51, 62, 92] three were of 'Critically low' quality,[43, 55, 60] and thirty-three 33 were of critically-low quality.[35, 36, 38-40, 42, 44, 45, 47, 49, 54, 59, 63-67, 73-75, 77-79, 84-91, 96, 97]

Three 'Critically Low' quality reviews synthesised quantitative evidence to optimise the prescribing of medications, including statins and antihypertensives.[83, 85, 87] Two further 'Critically Low' quality reviews synthesised evidence evaluating interventions to optimise the prescribing of antihypertensives alone.[37, 67]

Discontinuing treatment

Three reviews, one of 'Low' quality,[80] two of 'Critically Low' quality,[46, 93] synthesised quantitative evidence evaluating interventions promote deprescribing. All of these reviews had a broad remit, focusing on deprescribing medications for chronic diseases,[80] and older adults.[46, 94]

One systematic review of quantitative evidence evaluated impact of Medicare Part D on use of under and overuse of medication. Statins and antihypertensives were only two types of medication included in this review.[97]

One review of 'Low' quality focused on identifying potentially inappropriate prescribing in older adults with dementia.[26]

Two 'Critically-Low' quality reviews of qualitative evidence; one lay perspectives on hypertension and drug adherence,[53] one reported on patient and/or carer views barriers to and enablers of deprescribing where antihypertensives were the medication of interest.[58]

Two 'Critically Low' quality reviews synthesised quantitative evidence to optimise the prescribing of antihypertensives.[37, 50]

For peer review only - http://bmjopen.bmj.com/site/about/guidelines.xhtml

For peer review only

Prescribing guidelines

One 'Moderate' quality review, relevant to both the prescribing of statins and antihypertensives, synthesised quantitative evidence evaluating interventions to improve adherence to CVD guidelines.[82]

DISCUSSION

We aimed to provide a resource to enable policy makers, commissioners, clinicians and other decision makers to find and view existing systematic review evidence relevant to optimising the prescribing of statin and antihypertensive medication. The evidence and gap map displays systematic review evidence synthesising quantitative evidence evaluating interventions to improve or optimise medication adherence or prescribing and also qualitative evidence examining the perspectives of patients, carers and clinicians of issues which can affect optimal prescribing. This evidence is mapped onto a patient care pathway, according to the type of medication of interest, enabling map users to find and view the evidence most suited to their needs.

The map highlights a cluster of systematic review evidence evaluating interventions to promote adherence to medication, particularly antihypertensive, relevant to the "Pre-treatment" and "Maintaining treatment" sections of the patient care pathway. An identified gap within the pathway is that of qualitative evidence which seeks to explore the views and/or experiences of these types of intervention.

Strengths and limitations

This evidence and gap map will be a useful resource for decision makers who are seeking systematic review evidence to answer questions relating to the optimal prescribing of statins or antihypertensive medication. The maps functionality means that the key characteristics of the evidence are easily displayed and allow for map users to access a summary or link to full texts as required. The map also identifies both clusters of existing systematic review evidence and gaps, highlighting where further systematic review evidence may or may not be required. However, this map includes only systematic review evidence and thus there may be primary research relevant to the topic which may sit within the identified gaps. In addition, the majority of evidence in this map was of critically-low quality. These systematic reviews may be of more limited use to decision makers and mask gaps where further evidence syntheses or commissioning of primary research is required.

Our search strategy did not capture systematic reviews used to inform NICE guidelines relevant to the review topic. These reviews were not retrieved by our bibliographic database searches,

For peer review only - http://bmjopen.bmj.com/site/about/guidelines.xhtml

highlighting issues regarding the visibility of the reviews contributing to NICE guidelines. It was beyond our resources to search through individual guidelines to locate relevant reviews.

Implications of our findings

For research commissioners, the map highlights where further research may be useful, including the following specific areas:

- Further synthesis of existing systematic reviews of experiences of taking or adhering to statin and/or antihypertensive medication;
- Searching for primary studies where gaps in high quality systematic review evidence have been identified, for example, qualitative evidence regarding patient and/or prescriber experiences of specific interventions to increase medication adherence or practitioner experiences of prescribing statin medication.

The clusters of existing research may also be useful in informing government policy, however caution is required as many of the systematic reviews are of poor quality which may limit the confidence that can be placed in their findings.

For clinicians, the qualitative evidence within the map highlights factors which could influence patient's seeking out or accessing health services for CVD and their willingness or ability to take any medication which is prescribed to them. This may be helpful in informing clinical practice. The map also includes systematic review evidence evaluating interventions to optimise medication adherence, which may also be useful to inform clinical practice.

CONCLUSIONS

This evidence and gap map presents a snapshot of the systematic review evidence available to inform the optimal prescribing of statin and antihypertensive medication, displayed to indicate the quality and quantity of evidence available to inform decision making at key points in the patient care pathway. The evidence included within the map may support policy makers and service commissioners or aid in the commissioning of further research. The map may also help inform the prescribing decisions of clinicians. Whilst some caution is required when interpreting the content of the map given the limitations of the included evidence and methodology used within this mapping review as indicated above, this map provides a useful resource for a variety of stakeholders to access recent systematic review evidence in this topic area.

For peer review only

For peer review only - http://bmjopen.bmj.com/site/about/guidelines.xhtml

ACKNOWLEDGEMENTS

Sue Whiffin and Jenny Lowe for administrative support. Morwenna Rodgers for support developing the protocol and search strategy. Kirstin Liabo for support developing the protocol and facilitating involvement of patients and members of the public with the review. The PenARC Patient Engagement Group for their insight throughout the review process. Dr Phil Evans for his support developing the patient care pathways and feedback on the draft evidence and gap map. Melissa Bond from the EPPI centre for continued support in developing the evidence and gap map.

CONTRIBUTION OF AUTHORS

Liz Shaw: Lead author. Led scoping process and contributed to the development of the protocol and conduct of screening, data extraction, quality appraisal. Led development of the evidence-and-gap map, patient and public involvement and write up of paper. Supported liaison with government and clinical stakeholders.

Simon Briscoe: Information specialist and reviewer. Supported scoping and led to the development of the search strategy to inform the protocol. Contributed to screening, data extraction and quality appraisal and was responsible for data management throughout the project. Developed PRISMA diagram and contributed to all stakeholder involvement and write up of paper.

Michael Nunns: Reviewer. Supported development of the protocol and contributed to screening, data extraction, quality appraisal and write up of paper.

Hassanat Lawal: Reviewer. Supported screening, data extraction and quality appraisal and reviewed final paper.

G.J. Melendez-Torres: Supported conceptualisation of the project and development of the protocol. Supported development of the evidence and gap map and government stakeholder involvement. Read and reviewed final paper.

Malcolm Turner: Patient and Public Involvement representative and lead. Supported development of the protocol and provided feedback on main findings. Read and reviewed final paper.

Ruth Garside: Supported conceptualisation of the project and development of the protocol. Supported development of the evidence and gap map and government stakeholder involvement. Read and reviewed final paper.

Jo Thompson-Coon: Supported conceptualisation of the project and development of the protocol. Supported development of the evidence and gap map and led government stakeholder involvement. Read and reviewed final paper. Guarantor of work.

For peer review only - http://bmjopen.bmj.com/site/about/guidelines.xhtml

Conflicts of interest: None to declare.

Funding: This paper represents part of a larger piece of work commissioned by the National Institute of Health Research Policy Research Programme by the Exeter PRP Evidence Review Facility (NIHR200695) following a request to the Department of Health and Social Care (DHSC) Research and Development Committee by NHS England and Improvement (NHSE-I).

This paper is independent research funded by the National Institute for Health and Care Research Policy Research Programme (NIHR200605) and supported by the National Institute for Health and Care Research Applied Research Collaboration South West Peninsula. The views expressed in this publication are those of the author(s) and not necessarily those of the National Institute for Health and Care Research, the Department of Health and Social Care or NHS England and Improvement.

Data sharing: For access to documentation supporting this review, please contact L. Shaw.

Ethics approval: This review is secondary research and did not require ethical approval.

Patient consent statement: This review is secondary research and did not require collection of patient data.

REFERENCES

1. WHO 2021 https://www.who.int/news-room/fact-sheets/detail/cardiovascular-diseases-(cvds) Accessed 15.9.21
2. Ekinci G. Economic Impacts of Cardiovascular Diseases: An Econometric Evaluation in Turkey. Iran J Public Health. 2023 Jan;52(1):118-127. doi: 10.18502/ijph.v52i1.11673. PMID: 36824237; PMCID: PMC9941436
3. NHSE 2021 https://www.england.nhs.uk/ourwork/clinical-policy/cvd/ Accessed 15.9.21

4. National Institute for Health and Care Excellence. *Cardiovascular disease: risk assessment and reduction, including lipid modification [CG181]. Last updated: 27 September 2016*: NICE; 2014.
5. Marcus ME, Manne-Goehler J, Theilmann M, Farzadfar F, Moghaddam SS, Keykhaei M, Hajebi A, Tschida S, Lemp JM, Aryal KK, Dunn M, Houehanou C, Bahendeka S, Rohloff P, Atun R, Bärnighausen TW, Geldsetzer P, Ramirez-Zea M, Chopra V, Heisler M, Davies JI, Huffman MD, Vollmer S, Flood D. Use of statins for the prevention of cardiovascular disease in 41 low-income and middle-income countries: a cross-sectional study of nationally representative, individual-level data. Lancet Glob Health. 2022 Mar;10(3):e369-e379. doi: 10.1016/S2214-109X(21)00551-9.
6. Kjeldsen SE. Hypertension and cardiovascular risk: General aspects. Pharmacol Res 2018;129:95-9. https://doi.org/10.1016/j.phrs.2017.11.003
7. Gupta A, Mackay J, Whitehouse A, Godec T, Collier T, Pocock S*, et al.* Long-term mortality after blood pressure-lowering and lipid-lowering treatment in patients with hypertension in the Anglo-Scandinavian Cardiac Outcomes Trial (ASCOT) Legacy study: 16-year follow-up results of a randomised factorial trial. *The Lancet* 2018;**392**:1127-37. https://doi.org/https://doi.org/10.1016/S0140-6736(18)31776-8

For peer review only - http://bmjopen.bmj.com/site/about/guidelines.xhtml

8. NCD Risk Factor Collaboration (NCD-RisC). Worldwide trends in hypertension prevalence and progress in treatment and control from 1990 to 2019: a pooled analysis of 1201 population-representative studies with 104 million participants. Lancet. 2021 Sep 11;398(10304):957-980. doi: 10.1016/S0140-6736(21)01330-1. Epub 2021 Aug 24. Erratum in: Lancet. 2022 Feb 5;399(10324):520. PMID: 34450083; PMCID: PMC8446938
9. NCD Risk Factor Collaboration. Long-term and recent trends in hypertension awareness, treatment, and control in 12 high-income countries: an analysis of 123 nationally representative surveys. *Lancet* 2019;**394**:639-51. https://doi.org/10.1016/s0140-6736(19)31145-6
10. Suissa S, Brophy J, Azoulay L, Schuster T. *Trends in Prescription of Antihypertensive Medications in UK Primary Care Practices, 1988-2018*. Clinical Practice Research Datalink; 2019. URL: https://cprd.com/protocol/trends-prescription-antihypertensive-medications-uk-primary-care-practices-1988-2018 (accessed 1 March, 2021).
11. Gupta P, Patel P, Štrauch B, Lai FY, Akbarov A, Marešová V*, et al.* Risk Factors for Nonadherence to Antihypertensive Treatment. *Hypertension* 2017;**69**:1113-20. https://doi.org/10.1161/hypertensionaha.116.08729
12. Reference full report. Withheld for peer review.
13. Centre for Reviews and Dissemination. *Systematic Reviews: CRD's guidance for undertaking reviews in health care*: University of York; 2008.
14. White H, Albers B, Gaarder M, Kornør H, Littell J, Marshall Z*, et al.* Guidance for producing a Campbell evidence and gap map. *Campbell Systematic Reviews* 2020;**16**:e1125. https://doi.org/10.1002/cl2.1125
15. Review protocol. Reference withheld for peer review
16. Moher D, Liberati A, Tetzlaff J, Altman DG. Preferred reporting items for systematic reviews and meta-analyses: the PRISMA statement. *BMJ* 2009;**339**:b2535. https://doi.org/10.1136/bmj.b2535
17. Collaboration for Environmental Evidence. *The Collaboration for Environmental Evidence Synthesis Appraisal Tool (CEESAT). Version 2.1. (Last updated 27 October 2020).* CEE; 2018. URL: https://environmentalevidence.shinyapps.io/CEEDER/ (accessed 13 Jan, 2021).
18. Shea BJ, Reeves BC, Wells G, Thuku M, Hamel C, Moran J*, et al.* AMSTAR 2: a critical appraisal tool for systematic reviews that include randomised or non-randomised studies of healthcare interventions, or both. *BMJ* 2017;**358**:j4008. https://doi.org/10.1136/bmj.j4008
19. Tong A, Flemming K, McInnes E, Oliver S, Craig J. Enhancing transparency in reporting the synthesis of qualitative research: ENTREQ. *BMC Med Res Methodol* 2012;**12**:181. https://doi.org/10.1186/1471-2288-12-181
20. Digital Solution Foundry, EPPI-Centre. *EPPI Mapper, Version 1.2.5*: EPPI-Centre, UCL Social Research Institute, University College London; 2020.
21. National Institute for Health and Care Excellence. *Hypertension in adults: diagnosis and management NICE guideline [NG136]* London: NICE; 2019.
22. National Institute for Health and Care Excellence. *Medicines optimisation: the safe and effective use of medicines to enable the best possible outcomes* London: NICE; 2015.
23. National Institute for Health and Care Excellence. *Shared decision-making about medicines*. NICE Pathways; 2021. URL: https://pathways.nice.org.uk/pathways/medicines-optimisation#path=view%3A/pathways/medicines-optimisation/shared-decision-making-about-medicines.xml&content=view-index (accessed 3 March, 2021).
24. Pound P, Britten N, Morgan M, Yardley L, Pope C, Daker-White G*, et al.* Resisting medicines: a synthesis of qualitative studies of medicine taking. *Soc Sci Med* 2005;**61**:133-55. https://doi.org/10.1016/j.socscimed.2004.11.063

For peer review only - http://bmjopen.bmj.com/site/about/guidelines.xhtml

25. Deichmann RE, Morledge MD, Ulep R, Shaffer JP, Davies P, van Driel ML. A Metaanalysis of Interventions to Improve Adherence to Lipid-Lowering Medication. *Ochsner J* 2016;**16**:230-7.
26. Hukins D, Macleod U, Boland JW. Identifying potentially inappropriate prescribing in older people with dementia: a systematic review. *Eur J Clin Pharmacol* 2019;**75**:467-81. https://doi.org/10.1007/s00228-018-02612-x
27. Kinnear FJ, Wainwright E, Perry R, Lithander FE, Bayly G, Huntley A*, et al.* Enablers and barriers to treatment adherence in heterozygous familial hypercholesterolaemia: a qualitative evidence synthesis. *BMJ Open* 2019;**9**:e030290. https://doi.org/10.1136/bmjopen-2019-030290
28. Legare F, Ratte S, Stacey D, Kryworuchko J, Gravel K, Graham ID*, et al.* Interventions for improving the adoption of shared decision making by healthcare professionals. *Cochrane Database Syst Rev* 2010; 10.1002/14651858.CD006732.pub2:CD006732. https://doi.org/10.1002/14651858.CD006732.pub2
29. Nguyen T, Nguyen HQ, Widyakusuma NN, Nguyen TH, Pham TT, Taxis K. Enhancing prescribing of guideline-recommended medications for ischaemic heart diseases: a systematic review and meta-analysis of interventions targeted at healthcare professionals. *BMJ Open* 2018;**8**:e018271. https://doi.org/10.1136/bmjopen-2017-018271
30. Rash JA, Campbell DJ, Tonelli M, Campbell TS. A systematic review of interventions to improve adherence to statin medication: What do we know about what works? *Prev Med* 2016;**90**:155-69. https://doi.org/10.1016/j.ypmed.2016.07.006
31. Rashid MA, Edwards D, Walter FM, Mant J. Medication taking in coronary artery disease: a systematic review and qualitative synthesis. *Ann Fam Med* 2014;**12**:224-32. https://doi.org/10.1370/afm.1620
32. van Driel ML, Morledge MD, Ulep R, Shaffer JP, Davies P, Deichmann R. Interventions to improve adherence to lipid-lowering medication. *Cochrane Database of Systematic Reviews* 2016; 10.1002/14651858.CD004371.pub4. https://doi.org/10.1002/14651858.CD004371.pub4
33. Bond, Z.; Scanlon, T.; Judah, G. Systematic Review of RCTs Assessing the Effectiveness of mHealth Interventions to Improve Statin Medication Adherence: Using the Behaviour-Change Technique Taxonomy to Identify the Techniques That Improve Adherence. Healthcare 2021, 9, 1282. https://doi.org/ 10.3390/healthcare9101282
34. Reston JT, Buelt A, Donahue MP, Neubauer B, Vagichev E, McShea K. Interventions to Improve Statin Tolerance and Adherence in Patients at Risk for Cardiovascular Disease : A Systematic Review for the 2020 U.S. Department of Veterans Affairs and U.S. Department of Defense Guidelines for Management of Dyslipidemia. Ann Intern Med. 2020 Nov 17;173(10):806-812. doi: 10.7326/M20-4680
35. Bochkareva EV, Butina EK, Kim IV, Kontsevaya AV, Drapkina OM, Leon D, et al. Adherence to antihypertensive medication in Russia: a scoping review of studies on levels, determinants and intervention strategies published between 2000 and 2017. Arch Public Health 2019;77:43. https://doi.org/10.1186/s13690-019-0366-9
36. Cheema E, Sutcliffe P, Singer DR. The impact of interventions by pharmacists in community pharmacies on control of hypertension: a systematic review and meta-analysis of randomized controlled trials. Br J Clin Pharmacol 2014;78:1238-47. https://doi.org/10.1111/bcp.12452
37. Chhina HK, Bhole VM, Goldsmith C, Hall W, Kaczorowski J, Lacaille D. Effectiveness of academic detailing to optimize medication prescribing behaviour of family physicians. J Pharm Pharm Sci 2013;16:511-29. https://doi.org/10.18433/j3kk6c

For peer review only - http://bmjopen.bmj.com/site/about/guidelines.xhtml

38. Conn VS, Ruppar TM, Chase JA, Enriquez M, Cooper PS. Interventions to Improve Medication Adherence in Hypertensive Patients: Systematic Review and Meta-analysis. Curr Hypertens Rep 2015;17:94. https://doi.org/10.1007/s11906-015-0606-5
39. De Simoni A, Hardeman W, Mant J, Farmer AJ, Kinmonth AL. Trials to improve blood pressure through adherence to antihypertensives in stroke/TIA: systematic review and meta-analysis. J Am Heart Assoc 2013;2:e000251. https://doi.org/10.1161/JAHA.113.000251
40. Demonceau J, Ruppar T, Kristanto P, Hughes DA, Fargher E, Kardas P, et al. Identification and assessment of adherence-enhancing interventions in studies assessing medication adherence through electronically compiled drug dosing histories: a systematic literature review and meta-analysis. Drugs 2013;73:545-62. https://doi.org/10.1007/s40265-013-0041-3
41. Dhar L, Dantas J, Ali M. A Systematic Review of Factors Influencing Medication Adherence to Hypertension Treatment in Developing Countries. Open Journal of Epidemiology 2017;07:211-50. https://doi.org/10.4236/ojepi.2017.73018
42. Du LP, Cheng ZW, Zhang YX, Li Y, Mei D. The impact of fixed-dose combination versus free-equivalent combination therapies on adherence for hypertension: a meta-analysis. J Clin Hypertens (Greenwich) 2018;20:902-7. https://doi.org/10.1111/jch.13272
43. Fletcher BR, Hartmann-Boyce J, Hinton L, McManus RJ. The Effect of Self-Monitoring of Blood Pressure on Medication Adherence and Lifestyle Factors: A Systematic Review and Meta-Analysis. Am J Hypertens 2015;28:1209-21. https://doi.org/10.1093/ajh/hpv008
44. Gupta AK, Arshad S, Poulter NR. Compliance, safety, and effectiveness of fixed-dose combinations of antihypertensive agents: a meta-analysis. Hypertension 2010;55:399-407. https://doi.org/10.1161/HYPERTENSIONAHA.109.139816
45. Guzman-Tordecilla DN, Bernal Garcia A, Rodriguez I. Interventions to increase the pharmacological adherence on arterial hypertension in Latin America: a systematic review. Int J Public Health 2020;65:55-64. https://doi.org/10.1007/s00038-019-01317-x
46. Hart LA, Phelan EA, Yi JY, Marcum ZA, Gray SL. Use of Fall Risk-Increasing Drugs Around a Fall-Related Injury in Older Adults: A Systematic Review. J Am Geriatr Soc 2020;68:1334-43. https://doi.org/10.1111/jgs.16369
47. Kawalec P, Holko P, Gawin M, Pilc A. Effectiveness of fixed-dose combination therapy in hypertension: systematic review and meta-analysis. Arch Med Sci 2018;14:1125-36. https://doi.org/10.5114/aoms.2018.77561
48. Khatib R, Schwalm JD, Yusuf S, Haynes RB, McKee M, Khan M, et al. Patient and healthcare provider barriers to hypertension awareness, treatment and follow up: a systematic review and meta-analysis of qualitative and quantitative studies. PLoS One 2014;9:e84238. https://doi.org/10.1371/journal.pone.0084238
49. Kronish IM, Woodward M, Sergie Z, Ogedegbe G, Falzon L, Mann DM. Meta-analysis: impact of drug class on adherence to antihypertensives. Circulation 2011;123:1611-21. https://doi.org/10.1161/CIRCULATIONAHA.110.983874
50. Loganathan M, Singh S, Franklin BD, Bottle A, Majeed A. Interventions to optimise prescribing in care homes: systematic review. Age Ageing 2011;40:150-62. https://doi.org/10.1093/ageing/afq161
51. Maimaris W, Paty J, Perel P, Legido-Quigley H, Balabanova D, Nieuwlaat R, et al. The influence of health systems on hypertension awareness, treatment, and control: a systematic literature review. PLoS Med 2013;10:e1001490. https://doi.org/10.1371/journal.pmed.1001490

For peer review only - http://bmjopen.bmj.com/site/about/guidelines.xhtml

52. Mallat SG, Tanios BY, Itani HS, Lotfi T, Akl EA. Free versus Fixed Combination Antihypertensive Therapy for Essential Arterial Hypertension: A Systematic Review and Meta-Analysis. PLoS One 2016;11:e0161285. https://doi.org/10.1371/journal.pone.0161285
53. Marshall IJ, Wolfe CD, McKevitt C. Lay perspectives on hypertension and drug adherence: systematic review of qualitative research. BMJ 2012;345:e3953. https://doi.org/10.1136/bmj.e3953
54. Milosavljevic A, Aspden T, Harrison J. Community pharmacist-led interventions and their impact on patients' medication adherence and other health outcomes: a systematic review. Int J Pharm Pract 2018;26:387-97. https://doi.org/10.1111/ijpp.12462
55. Morrissey EC, Durand H, Nieuwlaat R, Navarro T, Haynes RB, Walsh JC, et al. Effectiveness and content analysis of interventions to enhance medication adherence and blood pressure control in hypertension: A systematic review and meta-analysis. Psychol Health 2017;32:1195-232. https://doi.org/10.1080/08870446.2016.1273356
56. Oori MJ, Mohammadi F, Norouzi K, Fallahi-Khoshknab M, Ebadi A. Conceptual Model of Medication Adherence in Older Adults with High Blood Pressure-An Integrative Review of the Literature. Curr Hypertens Rev 2019;15:85-92. https://doi.org/10.2174/1573402114666181022152313
57. Rashid MA, Llanwarne N, Heyns N, Walter F, Mant J. What are the implications for practice that arise from studies of medication taking? A systematic review of qualitative research. PLoS One 2018;13:e0195076. https://doi.org/10.1371/journal.pone.0195076
58. Reeve E, To J, Hendrix I, Shakib S, Roberts MS, Wiese MD. Patient barriers to and enablers of deprescribing: a systematic review. Drugs Aging 2013;30:793-807. https://doi.org/10.1007/s40266-013-0106-8
59. Reeves L, Robinson K, McClelland T, Adedoyin CA, Broeseker A, Adunlin G. Pharmacist Interventions in the Management of Blood Pressure Control and Adherence to Antihypertensive Medications: A Systematic Review of Randomized Controlled Trials. J Pharm Pract 2020; 10.1177/0897190020903573:897190020903573. https://doi.org/10.1177/0897190020903573
60. Sanders GD, Coeytaux R, Dolor RJ, Hasselblad V, Patel UD, Powers B, et al. AHRQ Comparative Effectiveness Reviews. In: Angiotensin-Converting Enzyme Inhibitors (ACEIs), Angiotensin II Receptor Antagonists (ARBs), and Direct Renin Inhibitors for Treating Essential Hypertension: An UpdateRockville (MD): Agency for Healthcare Research and Quality (US); 2011.
61. Shanbhag D, Graham ID, Harlos K, Haynes RB, Gabizon I, Connolly SJ, et al. Effectiveness of implementation interventions in improving physician adherence to guideline recommendations in heart failure: a systematic review. BMJ Open 2018;8:e017765. https://doi.org/10.1136/bmjopen-2017-017765
62. Stacey D, Legare F, Lewis K, Barry MJ, Bennett CL, Eden KB, et al. Decision aids for people facing health treatment or screening decisions. Cochrane Database Syst Rev 2017;4:CD001431. https://doi.org/10.1002/14651858.CD001431.pub5
63. Tsioufis K, Kreutz R, Sykara G, van Vugt J, Hassan T. Impact of single-pill combination therapy on adherence, blood pressure control, and clinical outcomes: a rapid evidence assessment of recent literature. Journal of Hypertension 2020;38:1016-28. https://doi.org/10.1097/Hjh.0000000000002381
64. Uhlig K, Patel K, Ip S, Kitsios GD, Balk EM. Self-measured blood pressure monitoring in the management of hypertension: a systematic review and meta-analysis. Ann Intern Med 2013;159:185-94. https://doi.org/10.7326/0003-4819-159-3-201308060-00008

For peer review only - http://bmjopen.bmj.com/site/about/guidelines.xhtml

65. Xiong S, Berkhouse H, Schooler M, Pu W, Sun A, Gong E, et al. Effectiveness of mHealth Interventions in Improving Medication Adherence Among People with Hypertension: a Systematic Review. Curr Hypertens Rep 2018;20:86. https://doi.org/10.1007/s11906-018-0886-7
66. Xu R, Xie X, Li S, Chen X, Wang S, Hu C, et al. Interventions to improve medication adherence among Chinese patients with hypertension: a systematic review and meta-analysis of randomized controlled trails. Int J Pharm Pract 2018;26:291-301. https://doi.org/10.1111/ijpp.12452
67. Yuan C, Ding Y, Zhou K, Huang Y, Xi X. Clinical outcomes of community pharmacy services: A systematic review and meta-analysis. Health Soc Care Community 2019;27:e567-e87. https://doi.org/10.1111/hsc.12794
68. Guzman-Tordecilla DN, Bernal García A, Rodríguez I. Interventions to increase the pharmacological adherence on arterial hypertension in Latin America: a systematic review. Int J Public Health. 2020 Jan;65(1):55-64. doi: 10.1007/s00038-019-01317-x
69. Hong J, Tiu YC, Leung PYB, Wong MF, Ng WY, Cheung D, Mok HY, Lam WY, Li KY, Wong CK. Interventions that improve adherence to antihypertensive medications in coronary heart disease patients: a systematic review. Postgrad Med J. 2022 Mar;98(1157):219-227. doi: 10.1136/postgradmedj-2020-139116
70. Parati G, Kjeldsen S, Coca A, Cushman WC, Wang J. Adherence to Single-Pill Versus Free-Equivalent Combination Therapy in Hypertension: A Systematic Review and Meta-Analysis. Hypertension. 2021 Feb;77(2):692-705. doi: 10.1161/HYPERTENSIONAHA.120.15781
71. Van Truong P, Wulan Apriliyasari R, Lin MY, Chiu HY, Tsai PS. Effects of self-management programs on blood pressure, self-efficacy, medication adherence and body mass index in older adults with hypertension: Meta-analysis of randomized controlled trials. Int J Nurs Pract. 2021 Apr;27(2):e12920. doi: 10.1111/ijn.12920
72. Adler AJ, Martin N, Mariani J, Tajer CD, Owolabi OO, Free C, et al. Mobile phone text messaging to improve medication adherence in secondary prevention of cardiovascular disease. Cochrane Database Syst Rev 2017;4:CD011851. https://doi.org/10.1002/14651858.CD011851.pub2
73. Al AlShaikh S, Quinn T, Dunn W, Walters M, Dawson J. Multimodal Interventions to Enhance Adherence to Secondary Preventive Medication after Stroke: A Systematic Review and Meta-Analyses. Cardiovasc Ther 2016;34:85-93. https://doi.org/10.1111/1755-5922.12176
74. Baumgartner A, Drame K, Geutjens S, Airaksinen M. Does the Polypill Improve Patient Adherence Compared to Its Individual Formulations? A Systematic Review. Pharmaceutics 2020;12:190. https://doi.org/10.3390/pharmaceutics12020190
75. Checchi KD, Huybrechts KF, Avorn J, Kesselheim AS. Electronic medication packaging devices and medication adherence: a systematic review. JAMA 2014;312:1237-47. https://doi.org/10.1001/jama.2014.10059
76. Conn VS, Ruppar TM, Chan KC, Dunbar-Jacob J, Pepper GA, De Geest S. Packaging interventions to increase medication adherence: systematic review and meta-analysis. Curr Med Res Opin 2015;31:145-60. https://doi.org/10.1185/03007995.2014.978939
77. Conn VS, Ruppar TM, Enriquez M, Cooper PS, Chan KC. Healthcare provider targeted interventions to improve medication adherence: systematic review and meta-analysis. Int J Clin Pract 2015;69:889-99. https://doi.org/10.1111/ijcp.12632
78. Conn VS, Ruppar TM, Enriquez M, Cooper P. Medication adherence interventions that target subjects with adherence problems: Systematic review and meta-analysis. Res Social Adm Pharm 2016;12:218-46. https://doi.org/10.1016/j.sapharm.2015.06.001

For peer review only - http://bmjopen.bmj.com/site/about/guidelines.xhtml

79. de Cates AN, Farr MR, Wright N, Jarvis MC, Rees K, Ebrahim S, et al. Fixed-dose combination therapy for the prevention of cardiovascular disease. Cochrane Database Syst Rev 2014; 10.1002/14651858.CD009868.pub2:CD009868. https://doi.org/10.1002/14651858.CD009868.pub2
80. Dills H, Shah K, Messinger-Rapport B, Bradford K, Syed Q. Deprescribing Medications for Chronic Diseases Management in Primary Care Settings: A Systematic Review of Randomized Controlled Trials. J Am Med Dir Assoc 2018;19:923-35 e2.
81. Ingersgaard MV, Helms Andersen T, Norgaard O, Grabowski D, Olesen K. Reasons for Nonadherence to Statins - A Systematic Review of Reviews. Patient Prefer Adherence 2020;14:675-91. https://doi.org/10.2147/PPA.S245365
82. Jeffery RA, To MJ, Hayduk-Costa G, Cameron A, Taylor C, Van Zoost C, et al. Interventions to improve adherence to cardiovascular disease guidelines: a systematic review. BMC Fam Pract 2015;16:147. https://doi.org/10.1186/s12875-015-0341-7
83. Kang JE, Han NY, Oh JM, Jin HK, Kim HA, Son IJ, et al. Pharmacist-involved care for patients with heart failure and acute coronary syndrome: a systematic review with qualitative and quantitative meta-analysis. J Clin Pharm Ther 2016;41:145-57. https://doi.org/10.1111/jcpt.12367
84. Kassavou A, Sutton S. Automated telecommunication interventions to promote adherence to cardio-metabolic medications: meta-analysis of effectiveness and meta-regression of behaviour change techniques. Health Psychol Rev 2018;12:25-42. https://doi.org/10.1080/17437199.2017.1365617
85. King S, Miani C, Exley J, Larkin J, Kirtley A, Payne RA. Impact of issuing longer- versus shorter-duration prescriptions: a systematic review. Br J Gen Pract 2018;68:e286-e92. https://doi.org/10.3399/bjgp18X695501
86. Krack G. How to make value-based health insurance designs more effective? A systematic review and meta-analysis. Eur J Health Econ 2019;20:841-56. https://doi.org/10.1007/s10198-019-01046-1
87. Laba TL, Bleasel J, Brien JA, Cass A, Howard K, Peiris D, et al. Strategies to improve adherence to medications for cardiovascular diseases in socioeconomically disadvantaged populations: a systematic review. Int J Cardiol 2013;167:2430-40. https://doi.org/10.1016/j.ijcard.2013.01.049
88. Lawrence M, Pringle J, Kerr S, Booth J, Govan L, Roberts NJ. Multimodal secondary prevention behavioral interventions for TIA and stroke: a systematic review and meta-analysis. PLoS One 2015;10:e0120902. https://doi.org/10.1371/journal.pone.0120902
89. Leslie RS. Adherence to medications to treat diabetes, hypertension, and hypercholesterolemia among Medicare Part D beneficiaries [PhD]. San Diego: University of California; 2016.
90. Mamudu HM, Paul TK, Veeranki SP, Budoff M. The effects of coronary artery calcium screening on behavioral modification, risk perception, and medication adherence among asymptomatic adults: a systematic review. Atherosclerosis 2014;236:338-50. https://doi.org/10.1016/j.atherosclerosis.2014.07.022
91. Mann BS, Barnieh L, Tang K, Campbell DJ, Clement F, Hemmelgarn B, et al. Association between drug insurance cost sharing strategies and outcomes in patients with chronic diseases: a systematic review. PLoS One 2014;9:e89168. https://doi.org/10.1371/journal.pone.0089168
92. Nieuwlaat R, Wilczynski N, Navarro T, Hobson N, Jeffery R, Keepanasseril A, et al. Interventions for enhancing medication adherence. Cochrane Database Syst Rev 2014;2014:CD000011. https://doi.org/10.1002/14651858.CD000011.pub4

For peer review only - http://bmjopen.bmj.com/site/about/guidelines.xhtml

93. Page AT, Clifford RM, Potter K, Schwartz D, Etherton-Beer CD. The feasibility and effect of deprescribing in older adults on mortality and health: a systematic review and meta-analysis. Br J Clin Pharmacol 2016;82:583-623. https://doi.org/10.1111/bcp.12975
94. Palmer MJ, Barnard S, Perel P, Free C. Mobile phone-based interventions for improving adherence to medication prescribed for the primary prevention of cardiovascular disease in adults. Cochrane Database Syst Rev 2018;6:CD012675. https://doi.org/10.1002/14651858.CD012675.pub2
95. Pantoja T, Grimshaw JM, Colomer N, Castanon C, Leniz Martelli J. Manually-generated reminders delivered on paper: effects on professional practice and patient outcomes. Cochrane Database Syst Rev 2019;12:CD001174. https://doi.org/10.1002/14651858.CD001174.pub4
96. Park YJ, Martin EG. Medicare Part D's Effects on Drug Utilization and Out-of-Pocket Costs: A Systematic Review. Health Serv Res 2017;52:1685-728. https://doi.org/10.1111/1475-6773.12534
97. Polinski JM, Donohue JM, Kilabuk E, Shrank WH. Medicare Part D's effect on the under- and overuse of medications: a systematic review. J Am Geriatr Soc 2011;59:1922-33. https://doi.org/10.1111/j.1532-5415.2011.03537.x
98. Posadzki P, Mastellos N, Ryan R, Gunn LH, Felix LM, Pappas Y, et al. Automated telephone communication systems for preventive healthcare and management of long-term conditions. Cochrane Database Syst Rev 2016;12:CD009921. https://doi.org/10.1002/14651858.CD009921.pub2
99. Coronado-Vázquez V, Canet-Fajas C, Delgado-Marroquín MT, Magallón-Botaya R, Romero-Martín M, Gómez-Salgado J. Interventions to facilitate shared decision-making using decision aids with patients in Primary Health Care: A systematic review. Medicine (Baltimore). 2020 Aug 7;99(32):e21389. doi: 10.1097/MD.0000000000021389
100. Nili M, Mohamed R, Kelly KM. A systematic review of interventions using health behavioral theories to improve medication adherence among patients with hypertension. Transl Behav Med. 2020 Oct 12;10(5):1177-1186. doi: 10.1093/tbm/ibaa020. PMID: 33044533
101. Palmer MJ, Machiyama K, Woodd S, Gubijev A, Barnard S, Russell S, Perel P, Free C. Mobile phone-based interventions for improving adherence to medication prescribed for the primary prevention of cardiovascular disease in adults. Cochrane Database of Systematic Reviews 2021, Issue 3. Art. No.: CD012675. DOI: 10.1002/14651858.CD012675.pub3
102. Qadi O, Lufumpa N, Adderley N, Bem D, Marshall T, Kokab F. Patients' and health professionals' attitudes and perceptions towards the initiation of preventive drugs for primary prevention of cardiovascular disease: a systematic review of qualitative studies. BJGP Open. 2020 Dec 15;4(5):bjgpopen20X101087. doi: 10.3399/bjgpopen20X101087
103. Vervloet M, Korevaar JC, Leemrijse CJ, Paget J, Zullig LL, van Dijk L. Interventions to Improve Adherence to Cardiovascular Medication: What About Gender Differences? A Systematic Literature Review. Patient Prefer Adherence. 2020 Oct 29;14:2055-2070. doi: 10.2147/PPA.S260562
104. Xie CX, Chen Q, Hincapié CA, Hofstetter L, Maher CG, Machado GC. Effectiveness of clinical dashboards as audit and feedback or clinical decision support tools on medication use and test ordering: a systematic review of randomized controlled trials. J Am Med Inform Assoc. 2022 Sep 12;29(10):1773-1785. doi: 10.1093/jamia/ocac094

For peer review only - http://bmjopen.bmj.com/site/about/guidelines.xhtml

FIGURES

Figure 1: Evidence and gap map

Figure 2: PRISMA diagram

Figure 3: Focus/aims of systematic review evidence included in the map

For peer review only

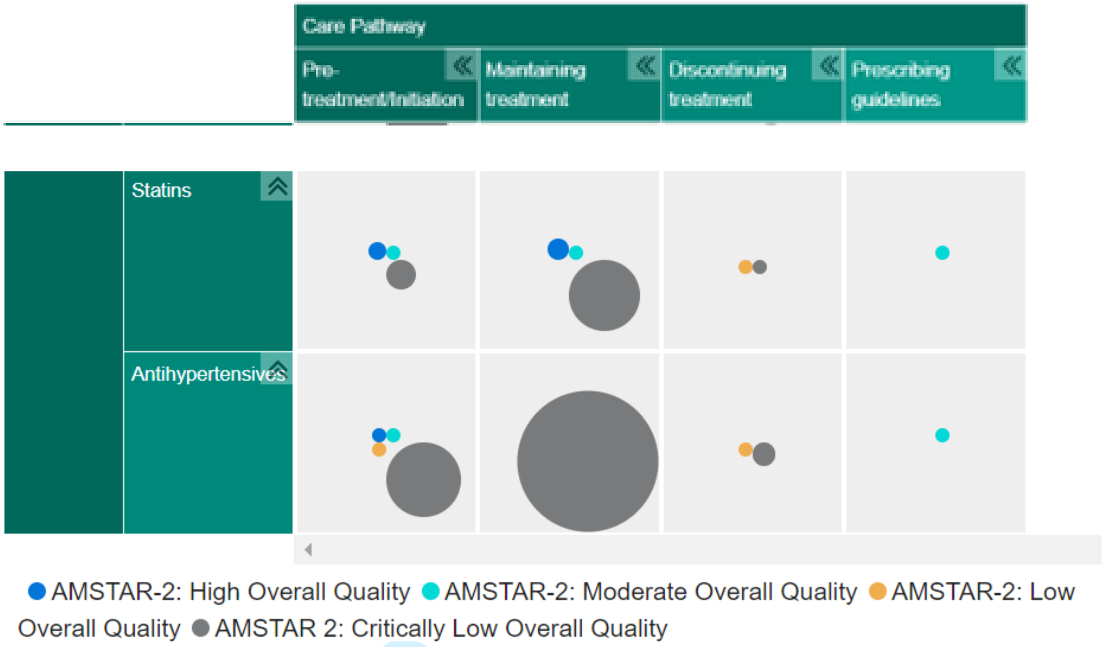

Figure 1: Evidence and gap map

For peer review only

PRISMA 2020 flow diagram for new systematic reviews which included searches of databases, registers and other sources

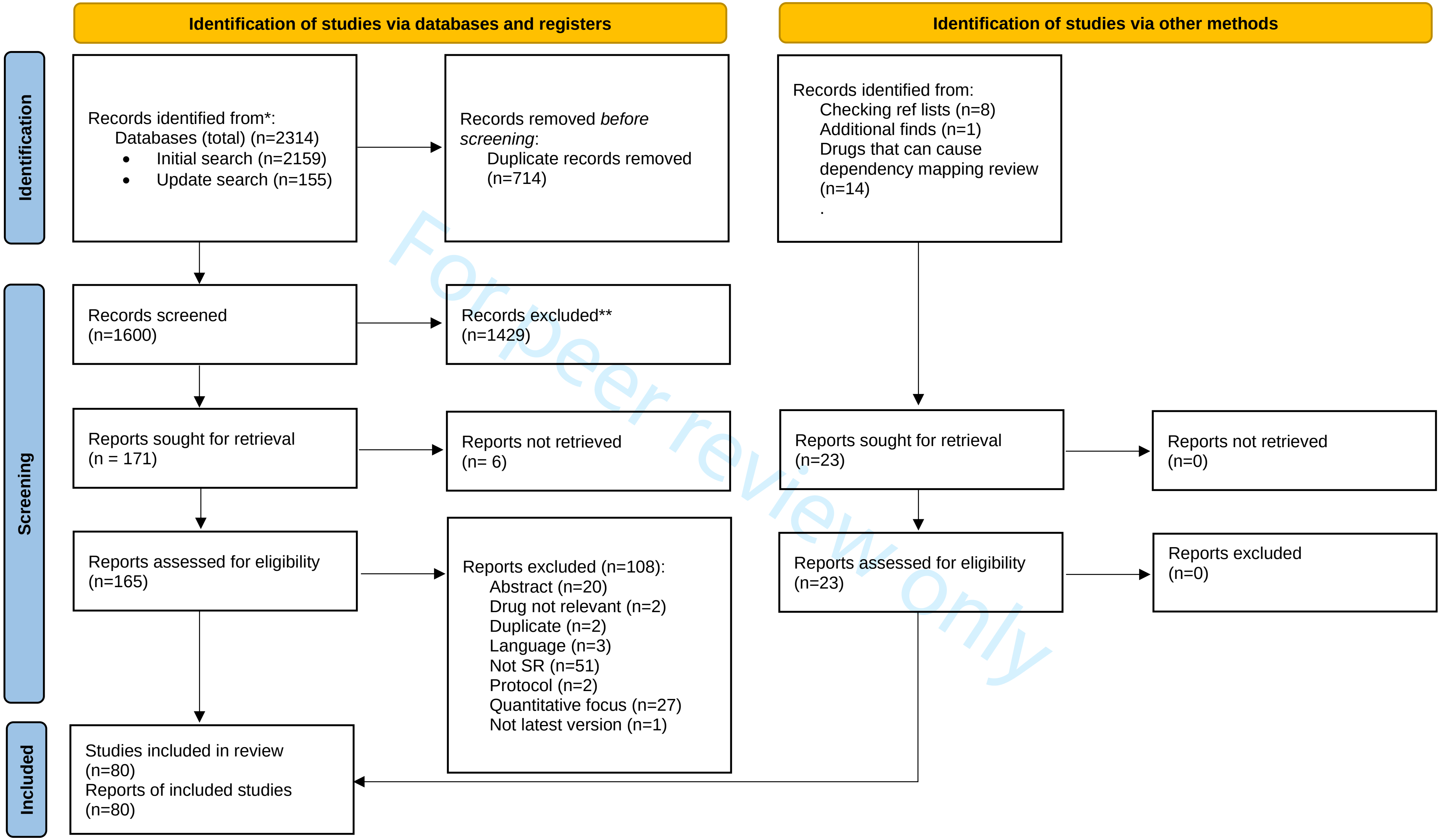

*Consider, if feasible to do so, reporting the number of records identified from each database or register searched (rather than the total number across all databases/registers).
**If automation tools were used, indicate how many records were excluded by a human and how many were excluded by automation tools.

From: Page MJ, McKenzie JE, Bossuyt PM, Boutron I, Hoffmann TC, Mulrow CD, et al. The PRISMA 2020 statement: an updated guideline for reporting systematic reviews. BMJ 2021;372:n71. doi: 10.1136/bmj.n71. For more information, visit: http://www.prisma-statement.org/

PRISMA 2020 flow diagram for new systematic reviews which included searches of databases, registers and other sources

For peer review only

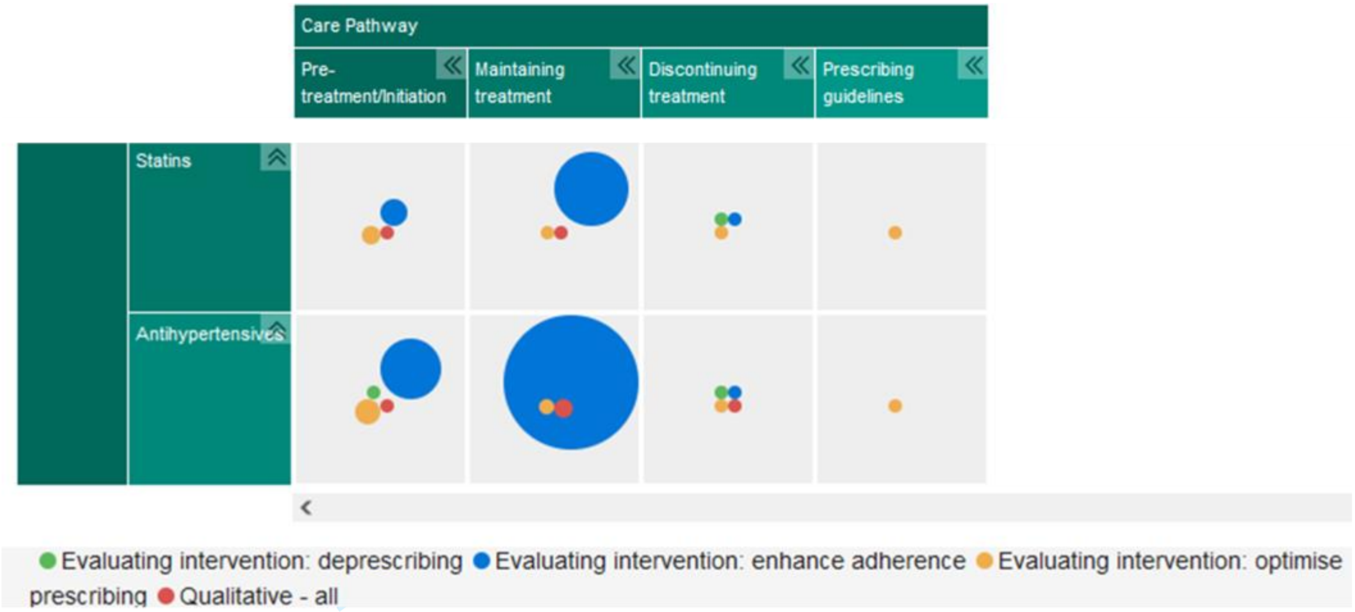

Figure 3: Focus/aims of systematic review evidence included in the map

APPENDIX A: SEARCH STRATEGY

Bibliographic database searches

Drugs that cause dependency

Database: Cochrane Database of Systematic Reviews
Host: Cochrane Library
Issue: Issue 8 of 12, August 2020
Date Searched: 11/8/2020
Searcher: SB
Hits: 111
Strategy:

#1 (((appropriate* or discontinu* or enhance* or inappropriate* or incorrect* or "in correct*" or optim* or safe or suboptim* or "sub optim*" or tapering or withdrawal) NEAR/4 (drug* or medicine* or medication* or prescri*))):ti,ab,kw

#2 MeSH descriptor: [Inappropriate Prescribing] this term only

#3 MeSH descriptor: [Drug Prescriptions] explode all trees

#4 (((drug* or guideline* or guidance or medicine* or medication* or patient* or prescri*) NEAR/4 (adhere* or compliance or concordance))):ti,ab,kw

#5 MeSH descriptor: [Patient Compliance] explode all trees

#6 ((shared or sharing or informed) near/2 (decision* or choice*)):ti,ab,kw

#7 ((decision near/2 (aid* or support*))):ti,ab,kw

#8 MeSH descriptor: [Decision Making] this term only

#9 MeSH descriptor: [Decision Support Techniques] this term only

#10 (((consumer* or patient*) near/3 (involv* or participat*))):ti,ab,kw

#11 MeSH descriptor: [Patient Participation] this term only

#12 ((patient NEXT cent* near/2 (approach* or care or decision* or intervention* or treatment*))):ti,ab,kw

#13 MeSH descriptor: [Patient-Centered Care] this term only

#14 ((behavi* near/2 chang*)):ti,ab,kw

#15 ((restriction near/2 (policy or policies))):ti,ab,kw

#16 {Wilson, #1-#15}

#17 (benzodiazepine*):ti,ab,kw

#18 ((alprazolam or flunitrazepam or chlordiazepoxide or clobazam or clonazepam or diazepam or lorazepam or midazolam or nitrazepam or oxazepam or prazepam or temazepam)):ti,ab,kw

#19 MeSH descriptor: [Benzodiazepines] explode all trees

For peer review only - http://bmjopen.bmj.com/site/about/guidelines.xhtml

#20 ((antidepres* or "anti depres*")):ti,ab,kw

#21 ((serotonin or norepinephrine or noradrenaline or neurotransmitter* or dopamin* or SSRI* or SNRI* or NARI* or SARI* or NDRI* or tricyclic* or tetracyclic*)):ti,ab,kw

#22 MeSH descriptor: [Antidepressive Agents] explode all trees

#23 ((opioid* or opiate*)):ti,ab,kw

#24 ((morphine or hydromorphone or levorphanol or meperidine or methadone or propoxyphene of codeine or pentazocine or hydrocodone or oxycodone or fentanyl or tramadol)):ti,ab,kw

#25 MeSH descriptor: [Analgesics, Opioid] explode all trees

#26 ("z drug*"):ti,ab,kw

#27 ((zopiclone or zolpidem or zaleplon or eszopiclone)):ti,ab,kw

#28 MeSH descriptor: [Hypnotics and Sedatives] explode all trees

#29 ((gabapentin* or mirogabalin or phenibut or pregabalin)):ti,ab,kw

#30 MeSH descriptor: [Gabapentin] this term only

#31 {Ahmed, #17-#30}

#32 #16 AND #31 in Cochrane Reviews [Drugs that cause dependency search results]

#33 (statin*):ti,ab,kw

#34 (("reductase inhibitors" or "HMGCR inhibitors")):ti,ab,kw

#35 MeSH descriptor: [Hydroxymethylglutaryl-CoA Reductase Inhibitors] explode all trees

#36 ((atorvastatin or Lipitor or Torvast)):ti,ab,kw

#37 ((fluvastatin or Lescol)):ti,ab,kw

#38 ((lovastatin or Mevacor or Altocor or Altoprev)):ti,ab,kw

#39 ((pitavastatin or Livalo or Pitava)):ti,ab,kw

#40 ((pravastatin or Pravachol or Selektine or Lipostat)):ti,ab,kw

#41 ((rosuvastatin or Crestor)):ti,ab,kw

#42 ((simvastatin or Zocor or Lipex)):ti,ab,kw

#43 (antihypertensive*):ti,ab,kw

#44 MeSH descriptor: [Antihypertensive Agents] explode all trees

#45 ((ACE NEXT inhibitor* or antagonist or "angiotensin II receptor" or beta NEXT blocker* or calcium NEXT channel NEXT blocker* or thiazide NEXT diuretic*)):ti,ab,kw

#46 ((acebutolol or adrenomedullin or alprenolol or amlodipine or atenolol or bendroflumethiazide or bepridil or betaxolol or bethanidine or bimatoprost or bisoprolol or bosentan or "bretylium tosylate" or brimonidine tartrate or bupranolol or captopril or carteolol or

For peer review only - http://bmjopen.bmj.com/site/about/guidelines.xhtml

carvedilol or celiprolol or chlorisondamine or chlorothiazide or chlorthalidone or cilazapril or clonidine or cromakalim or cyclopenthiazide or debrisoquin or diazoxide or dihydralazine or dihydroalprenolol or diltiazem or doxazosin or enalapril or enalaprilat or eplerenone or epoprostenol or felodipine or fenoldopam or fosinopril or guanabenz or guanethidine or guanfacine or hexamethonium or "hexamethonium compound*" or hydralazine or hydrochlorothiazide or hydroflumethiazide or indapamide or indoramin or irbesartan or isradipine or kallidin or ketanserin or labetalol or latanoprost or lisinopril or losartan or mecamylamine or methyldopa or metipranolol or metolazone or metoprolol or mibefradil or minoxidil or muzolimine or nadolol or nebivolol or nicardipine or nicorandil or nimodipine or nisoldipine or nitrendipine or nitroprusside or olmesartan medoxomil or oxprenolol or pargyline or pempidine or penbutolol or "pentolinium tartrate" or perindopril or phenoxybenzamine or phentolamine or pinacidil or pindolol or piperoxan or polythiazide or prazosin or propranolol or protoveratrines or quinapril or ramipril or reserpine or rilmenidine or telmisartan or teprotide or terlipressin or ticrynafen or timolol or todralazine or tolazoline or torsemide or travoprost or trichlormethiazide or trimethaphan or valsartan or "veratrum alkaloid*" or vincamine or xipamide)):ti,ab,kw

#47 (103-#46)

#48 (cardiovascular):ti,ab,kw

#49 MeSH descriptor: [Cardiovascular Diseases] explode all trees

#50 (((cardiac or coronary or heart) near/3 (arrest* or attack* or disease* or failure*))):ti,ab,kw

#51 ((heart or myocard* or transient) near/3 (infarc* or ischaemi* or ischemi*)):ti,ab,kw

#52 (angina*):ti,ab,kw

#53 MeSH descriptor: [Angina Pectoris] this term only

#54 (stroke*):ti,ab,kw

#55 MeSH descriptor: [Stroke] explode all trees

#56 {Ford, #48-#55}

#57 #16 and #47 and #56 in Cochrane Reviews [Statins search results]

Notes: Date limited January 2010 – to date of search.

Database: CINAHL
Host: EBSCO
Issue: n/a
Date Searched: 11/8/2020
Searcher: SB
Hits: 372
Strategy

1. TI ((appropriate* or discontinu* or enhance* or inappropriate* or incorrect* or "in correct*" or optim* or safe or suboptim* or "sub optim*" or tapering or withdrawal) N3 (drug* or medicine* or medication* or prescri*)) OR AB ((appropriate* or discontinu* or enhance* or inappropriate* or incorrect* or "in correct*" or optim* or safe or suboptim* or

For peer review only - http://bmjopen.bmj.com/site/about/guidelines.xhtml

For peer review only

"sub optim*" or tapering or withdrawal) N3 (drug* or medicine* or medication* or prescri*))

2. (MH "Inappropriate Prescribing")
3. (MM "Prescriptions, Drug+")
4. TI ((drug* or guideline* or guidance or medicine* or medication* or patient* or prescri*) N3 (adhere* or compliance or concordance)) OR AB ((drug* or guideline* or guidance or medicine* or medication* or patient* or prescri*) N3 (adhere* or compliance or concordance))
5. (MM "Patient Compliance+")
6. TI ((shared or sharing or informed) N1 (decision* or choice*)) OR AB ((shared or sharing or informed) N1 (decision* or choice*))
7. TI (decision N1 (aid* or support*)) OR AB (decision N1 (aid* or support*))
8. (MM "Decision Making")
9. (MM "Decision Support Techniques")
10. TI ((consumer* or patient*) N2 (involv* or participat*)) OR AB ((consumer* or patient*) N2 (involv* or participat*))
11. (MM "Consumer Participation")
12. TI ("patient cent*" N1 (approach* or care or decision* or intervention* or treatment*)) OR AB ("patient cent*" N1 (approach* or care or decision* or intervention* or treatment*))
13. (MH "Patient Centered Care")
14. TI (behavi* N1 chang*) OR AB (behavi* N1 chang*)
15. TI (restriction N1 (policy or policies)) OR AB (restriction N1 (policy or policies))
16. S1 OR S2 OR S3 OR S4 OR S5 OR S6 OR S7 OR S8 OR S9 OR S10 OR S11 OR S12 OR S13 OR S14 OR S15
17. TI benzodiazepine* OR AB benzodiazepine*
18. TI (alprazolam or flunitrazepam or chlordiazepoxide or clobazam or clonazepam or diazepam or lorazepam or midazolam or nitrazepam or oxazepam or prazepam or temazepam) OR AB (alprazolam or flunitrazepam or chlordiazepoxide or clobazam or clonazepam or diazepam or lorazepam or midazolam or nitrazepam or oxazepam or prazepam or temazepam)
19. (MH "Antianxiety Agents, Benzodiazepine+")
20. TI (antidepres* or "anti depres*") OR AB (antidepres* or "anti depres*")
21. TI ((serotonin or norepinephrine or noradrenaline or neurotransmitter* or dopamin* or SSRI* or SNRI* or NARI* or SARI* or NDRI* or tricyclic* or tetracyclic*)) OR AB ((serotonin or norepinephrine or noradrenaline or neurotransmitter* or dopamin* or SSRI* or SNRI* or NARI* or SARI* or NDRI* or tricyclic* or tetracyclic*))

For peer review only - http://bmjopen.bmj.com/site/about/guidelines.xhtml

22. (MH "Antidepressive Agents+")
23. TI ((opioid* or opiate*)) OR AB ((opioid* or opiate*))
24. TI ((morphine or hydromorphone or levorphanol or meperidine or methadone or propoxyphene of codeine or pentazocine or hydrocodone or oxycodone or fentanyl or tramadol)) OR AB ((morphine or hydromorphone or levorphanol or meperidine or methadone or propoxyphene of codeine or pentazocine or hydrocodone or oxycodone or fentanyl or tramadol))
25. (MH "Analgesics, Opioid+")
26. TI "z drug*" OR AB "z drug*"
27. TI ((zopiclone or zolpidem or zaleplon or eszopiclone)) OR AB ((zopiclone or zolpidem or zaleplon or eszopiclone))
28. (MH "Hypnotics and Sedatives")
29. TI ((gabapentin* or mirogabalin or phenibut or pregabalin)) OR AB ((gabapentin* or mirogabalin or phenibut or pregabalin))
30. (MH "Gabapentin")
31. S17 OR S18 OR S19 OR S20 OR S21 OR S22 OR S23 OR S24 OR S25 OR S26 OR S27 OR S28 OR S29 OR S30
32. S16 AND S31
33. TI statin* OR AB statin*
34. TI ("HMG-CoA reductase inhibitor*" or "3-hydroxy-3-methyl-glutaryl-CoA reductase inhibitor*" or "3-hydroxy-3-methylglutaryl coenzyme A reductase inhibitor*" or "HMGCR inhibitor*" or "Hydroxymethylglutaryl-CoA Reductase Inhibitor*") OR AB ("HMG-CoA reductase inhibitor*" or "3-hydroxy-3-methyl-glutaryl-CoA reductase inhibitor*" or "3-hydroxy-3-methylglutaryl coenzyme A reductase inhibitor*" or "HMGCR inhibitor*" or "Hydroxymethylglutaryl-CoA Reductase Inhibitor*")
35. (MH "Statins+")
36. TI (atorvastatin or Lipitor or Torvast) OR AB (atorvastatin or Lipitor or Torvast)
37. TI (fluvastatin or Lescol) OR AB (fluvastatin or Lescol)
38. TI (lovastatin or Mevacor or Altocor or Altoprev) OR AB (lovastatin or Mevacor or Altocor or Altoprev)
39. TI (pitavastatin or Livalo or Pitava) OR AB (pitavastatin or Livalo or Pitava)
40. TI (pravastatin or Pravachol or Selektine or Lipostat) OR AB (pravastatin or Pravachol or Selektine or Lipostat)
41. TI (rosuvastatin or Crestor) OR AB (rosuvastatin or Crestor)
42. TI (simvastatin or Zocor or Lipex) OR AB (simvastatin or Zocor or Lipex)
43. TI antihypertensive* OR AB antihypertensive*

For peer review only - http://bmjopen.bmj.com/site/about/guidelines.xhtml
For peer review only

44. (MH "Antihypertensive Agents+")

45. TI ("ACE inhibitor*" or antagonist or "angiotensin II receptor" or "beta blocker*" or "calcium channel blocker*" or "thiazide diuretic*") OR AB ("ACE inhibitor*" or antagonist or "angiotensin II receptor" or "beta blocker*" or "calcium channel blocker*" or "thiazide diuretic*")

46. TI (acebutolol or adrenomedullin or alprenolol or amlodipine or atenolol or bendroflumethiazide or bepridil or betaxolol or bethanidine or bimatoprost or bisoprolol or bosentan or "bretylium tosylate" or brimonidine tartrate or bupranolol or captopril or carteolol or carvedilol or celiprolol or chlorisondamine or chlorothiazide or chlorthalidone or cilazapril or clonidine or cromakalim or cyclopenthiazide or debrisoquin or diazoxide or dihydralazine or dihydroalprenolol or diltiazem or doxazosin or enalapril or enalaprilat or eplerenone or epoprostenol or felodipine or fenoldopam or fosinopril or guanabenz or guanethidine or guanfacine or hexamethonium or "hexamethonium compound*" or hydralazine or hydrochlorothiazide or hydroflumethiazide or indapamide or indoramin or irbesartan or isradipine or kallidin or ketanserin or labetalol or latanoprost or lisinopril or losartan or mecamylamine or methyldopa or metipranolol or metolazone or metoprolol or mibefradil or minoxidil or muzolimine or nadolol or nebivolol or nicardipine or nicorandil or nimodipine or nisoldipine or nitrendipine or nitroprusside or olmesartan medoxomil or oxprenolol or pargyline or pempidine or penbutolol or "pentolinium tartrate" or perindopril or phenoxybenzamine or phentolamine or pinacidil or pindolol or piperoxan or polythiazide or prazosin or propranolol or protoveratrines or quinapril or ramipril or reserpine or rilmenidine or telmisartan or teprotide or terlipressin or ticrynafen or timolol or todralazine or tolazoline or torsemide or travoprost or trichlormethiazide or trimethaphan or valsartan or "veratrum alkaloid*" or vincamine or xipamide) OR AB (acebutolol or adrenomedullin or alprenolol or amlodipine or atenolol or bendroflumethiazide or bepridil or betaxolol or bethanidine or bimatoprost or bisoprolol or bosentan or "bretylium tosylate" or brimonidine tartrate or bupranolol or captopril or carteolol or carvedilol or celiprolol or chlorisondamine or chlorothiazide or chlorthalidone or cilazapril or clonidine or cromakalim or cyclopenthiazide or debrisoquin or diazoxide or dihydralazine or dihydroalprenolol or diltiazem or doxazosin or enalapril or enalaprilat or eplerenone or epoprostenol or felodipine or fenoldopam or fosinopril or guanabenz or guanethidine or guanfacine or hexamethonium or "hexamethonium compound*" or hydralazine or hydrochlorothiazide or hydroflumethiazide or indapamide or indoramin or irbesartan or isradipine or kallidin or ketanserin or labetalol or latanoprost or lisinopril or losartan or mecamylamine or methyldopa or metipranolol or metolazone or metoprolol or mibefradil or minoxidil or muzolimine or nadolol or nebivolol or nicardipine or nicorandil or nimodipine or nisoldipine or nitrendipine or nitroprusside or olmesartan medoxomil or oxprenolol or pargyline or pempidine or penbutolol or "pentolinium tartrate" or perindopril or phenoxybenzamine or phentolamine or pinacidil or pindolol or piperoxan or polythiazide or prazosin or propranolol or protoveratrines or quinapril or ramipril or reserpine or rilmenidine or telmisartan or teprotide or terlipressin or ticrynafen or timolol or todralazine or tolazoline or torsemide or travoprost or trichlormethiazide or trimethaphan or valsartan or "veratrum alkaloid*" or vincamine or xipamide)

47. S33 OR S34 OR S35 OR S36 OR S37 OR S38 OR S39 OR S40 OR S41 OR S42 OR S43 OR S44 OR S45 OR S46

48. TI cardiovascular OR AB cardiovascular

For peer review only - http://bmjopen.bmj.com/site/about/guidelines.xhtml

49. (MH "Cardiovascular Diseases+")
50. TI ((cardiac or coronary or heart) N2 (arrest* or attack* or disease* or failure*)) OR AB ((cardiac or coronary or heart) N2 (arrest* or attack* or disease* or failure*))
51. TI ((heart or myocard* or transient) N2 (infarc* or ischaemi* or ischemi*)) OR AB ((heart or myocard* or transient) N2 (infarc* or ischaemi* or ischemi*))
52. TI angina* OR AB angina*
53. (MH "Angina Pectoris")
54. TI stroke* OR AB stroke*
55. (MH "Stroke+")
56. S48 OR S49 OR S50 OR S51 OR S52 OR S53 OR S54 OR S55
57. S16 AND S47 AND S56
58. TI ((effectiveness or implementation or literature or map or mapping or qualitative or rapid or realist or systematic or scoping or "state of the art" or umbrella) N1 (assessment* or overview* or review* or synthes*)) OR AB ((effectiveness or implementation or literature or map or mapping or qualitative or rapid or realist or systematic or scoping or "state of the art" or umbrella) N1 (assessment* or overview* or review* or synthes*))
59. TI ("meta analy*" or metaanaly* or metasynthe* or "meta synthe*") OR AB ("meta analy*" or metaanaly* or metasynthe* or "meta synthe*")
60. TI ((systematic or evidence) N0 assess*) OR AB ((systematic or evidence) N0 assess*)
61. TI (qualitative N1 (evidence or synthes*)) OR AB (qualitative N1 (evidence or synthes*))
62. TI overarching N1 model OR AB overarching N1 model
63. TI "review* of reviews" OR AB "review* of reviews"
64. S58 OR S59 OR S60 OR S61 OR S62 OR S63
65. S32 AND S64 [Drugs that cause dependency search results]
66. S57 AND S64 [Statins search results]

Notes: Date limited 2010 to date of search.

Database: Epistemonikos
Host: https://www.epistemonikos.org/en/
Issue: n/a
Date Searched: 12/8/2020
Searcher: SB
Hits: 56
Strategy

1. title:(prescription* OR prescribing OR prescribe*) OR abstract:(prescription* OR prescribing OR prescribe*)

For peer review only - http://bmjopen.bmj.com/site/about/guidelines.xhtml

2. title:(optimi* OR inappropriat* OR enhance*) OR abstract:(optimi* OR inappropriat* OR enhance*)
3. title:(benzodiazepine*) OR abstract:(benzodiazepine*) OR title:(antidepres* OR "anti depres*") OR abstract:(antidepres* OR "anti depres*") OR title:(opioid* OR opiate*) OR abstract:(opioid* OR opiate*) OR title:("z drug*") OR abstract:("z drug*") OR title:(gabapentin*) OR abstract:(gabapentin*)
4. 1 AND 2 AND 3

Notes: Date limited 2010 to 2020.

Database: medRxiv
Host: 12/8/2020
Issue: n/a
Date Searched:
Searcher: SB
Hits: 87
Strategy:

optimi* and prescri* and benzodiazepine *	5 hits
optimi* and prescri* and antidepres*	5 hits
optimi* and prescri* and opioid*	11 hits
optimi* and prescri* and "z drug*	66 hits
optimi* and prescri* and gabapentin*	0 hits

Database: Embase
Host: Ovid
Issue: 1974 to 2020 August 10
Date Searched: 11/8/2020
Searcher: SB
Hits: 1776
Strategy:

1. ((appropriate* or discontinu* or enhance* or inappropriate* or incorrect* or "in correct*" or optim* or safe or suboptim* or "sub optim*" or tapering or withdrawal) adj4 (drug* or medicine* or medication* or prescri*)).tw.
2. exp *inappropriate prescribing/
3. *prescription/
4. ((drug* or guideline* or guidance or medicine* or medication* or patient* or prescri*) adj4 (adhere* or compliance or concordance)).tw.
5. exp *patient compliance/
6. medication compliance/

For peer review only - http://bmjopen.bmj.com/site/about/guidelines.xhtml

7. ((shared or sharing or informed) adj2 (decision* or choice*)).tw.
8. (decision adj2 (aid* or support*)).tw.
9. exp *decision making/
10. exp *decision support system/
11. ((consumer* or patient*) adj3 (involv* or participat*)).tw.
12. *patient participation/
13. ("patient cent*" adj2 (approach* or care or decision* or intervention* or treatment*)).tw.
14. *patient care/
15. (behavi* adj2 chang*).tw.
16. *behavior change/
17. (restriction adj2 (policy or policies)).tw.
18. or/1-17
19. benzodiazepine*.tw.
20. (alprazolam or flunitrazepam or chlordiazepoxide or clobazam or clonazepam or diazepam or lorazepam or midazolam or nitrazepam or oxazepam or prazepam or temazepam).tw.
21. exp benzodiazepine derivative/
22. (antidepres* or "anti depres*").tw.
23. (serotonin or norepinephrine or noradrenaline or neurotransmitter* or dopamin* or SSRI* or SNRI* or NARI* or SARI* or NDRI* or tricyclic* or tetracyclic*).tw.
24. exp antidepressant agent/
25. (opioid* or opiate*).tw.
26. (morphine or hydromorphone or levorphanol or meperidine or methadone or propoxyphene of codeine or pentazocine or hydrocodone or oxycodone or fentanyl or tramadol).tw.
27. exp opiate/
28. "z drug*".tw.
29. (zopiclone or zolpidem or zaleplon or eszopiclone).tw.
30. hypnotic agent/
31. sedative agent/
32. (gabapentin* or mirogabalin or phenibut or pregabalin).tw.
33. gabapentin/
34. or/19-33
35. 18 and 34

For peer review only - http://bmjopen.bmj.com/site/about/guidelines.xhtml

For peer review only

36. statin*.tw.
37. ("HMG-CoA reductase inhibitor*" or "3-hydroxy-3-methyl-glutaryl-CoA reductase inhibitor*" or "3-hydroxy-3-methylglutaryl coenzyme A reductase inhibitor*" or "HMGCR inhibitor*" or "Hydroxymethylglutaryl-CoA Reductase Inhibitor*").tw.
38. "statin (protein)"/
39. (atorvastatin or Lipitor or Torvast).tw.
40. (fluvastatin or Lescol).tw.
41. (lovastatin or Mevacor or Altocor or Altoprev).tw.
42. (pitavastatin or Livalo or Pitava).tw.
43. (pravastatin or Pravachol or Selektine or Lipostat).tw.
44. (rosuvastatin or Crestor).tw.
45. (simvastatin or Zocor or Lipex).tw.
46. antihypertensive*.tw.
47. exp antihypertensive agent/
48. ("ACE inhibitor*" or antagonist or "angiotensin II receptor" or "beta blocker*" or "calcium channel blocker*" or "thiazide diuretic*").tw.
49. (acebutolol or adrenomedullin or alprenolol or amlodipine or atenolol or bendroflumethiazide or bepridil or betaxolol or bethanidine or bimatoprost or bisoprolol or bosentan or "bretylium tosylate" or brimonidine tartrate or bupranolol or captopril or carteolol or carvedilol or celiprolol or chlorisondamine or chlorothiazide or chlorthalidone or cilazapril or clonidine or cromakalim or cyclopenthiazide or debrisoquin or diazoxide or dihydralazine or dihydroalprenolol or diltiazem or doxazosin or enalapril or enalaprilat or eplerenone or epoprostenol or felodipine or fenoldopam or fosinopril or guanabenz or guanethidine or guanfacine or hexamethonium or "hexamethonium compound*" or hydralazine or hydrochlorothiazide or hydroflumethiazide or indapamide or indoramin or irbesartan or isradipine or kallidin or ketanserin or labetalol or latanoprost or lisinopril or losartan or mecamylamine or methyldopa or metipranolol or metolazone or metoprolol or mibefradil or minoxidil or muzolimine or nadolol or nebivolol or nicardipine or nicorandil or nimodipine or nisoldipine or nitrendipine or nitroprusside or olmesartan medoxomil or oxprenolol or pargyline or pempidine or penbutolol or "pentolinium tartrate" or perindopril or phenoxybenzamine or phentolamine or pinacidil or pindolol or piperoxan or polythiazide or prazosin or propranolol or protoveratrines or quinapril or ramipril or reserpine or rilmenidine or telmisartan or teprotide or terlipressin or ticrynafen or timolol or todralazine or tolazoline or torsemide or travoprost or trichlormethiazide or trimethaphan or valsartan or "veratrum alkaloid*" or vincamine or xipamide).tw.
50. or/36-49
51. cardiovascular.tw.
52. exp cardiovascular disease/

For peer review only - http://bmjopen.bmj.com/site/about/guidelines.xhtml

53. ((cardiac or coronary or heart) adj3 (arrest* or attack* or disease* or failure*)).tw.
54. ((heart or myocard* or transient) adj3 (infarc* or ischaemi* or ischemi*)).tw.
55. angina*.tw.
56. Angina Pectoris/
57. stroke*.tw.
58. exp cerebrovascular accident/
59. or/51-58
60. 18 and 50 and 59
61. ((effectiveness or implementation or literature or map or mapping or qualitative or rapid or realist or systematic or scoping or "state of the art" or umbrella) adj2 (assessment* or overview* or review* or synthes*)).tw.
62. ("meta analy*" or metaanaly* or metasynthe* or "meta synthe*").tw.
63. ((systematic or evidence) adj1 assess*).tw.
64. (qualitative adj2 (evidence or synthes*)).tw.
65. (overarching adj2 model).tw.
66. "review* of reviews".tw.
67. or/61-66
68. 35 and 67 [Drugs that cause dependency search results]
69. 60 and 67 [Statins search results]

Notes: Date limited 2010 to date of search.

Database: Health Management Information Consortium
Host: Ovid
Issue: 1979 to July 2020
Date Searched: 11/8/2020
Searcher: SB
Hits: 10
Strategy: see MEDLINE search strategy

Database: MEDLINE
Host: Ovid
Issue: 1946 to August 10, 2020
Date Searched: 11/8/2020
Searcher: SB
Hits: 875
Strategy:

For peer review only - http://bmjopen.bmj.com/site/about/guidelines.xhtml

1. ((appropriate* or discontinu* or enhance* or inappropriate* or incorrect* or "in correct*" or optim* or safe or suboptim* or "sub optim*" or tapering or withdrawal) adj4 (drug* or medicine* or medication* or prescri*)).tw.
2. Inappropriate Prescribing/
3. exp *Drug Prescriptions/
4. ((drug* or guideline* or guidance or medicine* or medication* or patient* or prescri*) adj4 (adhere* or compliance or concordance)).tw.
5. exp *Patient Compliance/
6. ((shared or sharing or informed) adj2 (decision* or choice*)).tw.
7. (decision adj2 (aid* or support*)).tw.
8. *Decision Making/
9. *decision support techniques/
10. ((consumer* or patient*) adj3 (involv* or participat*)).tw.
11. *patient participation/
12. ("patient cent*" adj2 (approach* or care or decision* or intervention* or treatment*)).tw.
13. Patient-Centered Care/
14. (behavi* adj2 chang*).tw.
15. (restriction adj2 (policy or policies)).tw.
16. or/1-15
17. benzodiazepine*.tw.
18. (alprazolam or flunitrazepam or chlordiazepoxide or clobazam or clonazepam or diazepam or lorazepam or midazolam or nitrazepam or oxazepam or prazepam or temazepam).tw.
19. exp Benzodiazepines/
20. (antidepres* or "anti depres*").tw.
21. (serotonin or norepinephrine or noradrenaline or neurotransmitter* or dopamin* or SSRI* or SNRI* or NARI* or SARI* or NDRI* or tricyclic* or tetracyclic*).tw.
22. exp Antidepressive Agents/
23. (opioid* or opiate*).tw.
24. (morphine or hydromorphone or levorphanol or meperidine or methadone or propoxyphene of codeine or pentazocine or hydrocodone or oxycodone or fentanyl or tramadol).tw.
25. exp Analgesics, Opioid/
26. "z drug*".tw.
27. (zopiclone or zolpidem or zaleplon or eszopiclone).tw.

For peer review only

For peer review only - http://bmjopen.bmj.com/site/about/guidelines.xhtml

28. exp "hypnotics and sedatives"/
29. (gabapentin* or mirogabalin or phenibut or pregabalin).tw.
30. Gabapentin/
31. or/17-30
32. 16 and 31
33. statin*.tw.
34. ("HMG-CoA reductase inhibitor*" or "3-hydroxy-3-methyl-glutaryl-CoA reductase inhibitor*" or "3-hydroxy-3-methylglutaryl coenzyme A reductase inhibitor*" or "HMGCR inhibitor*" or "Hydroxymethylglutaryl-CoA Reductase Inhibitor*").tw.
35. exp Hydroxymethylglutaryl-CoA Reductase Inhibitors/
36. (atorvastatin or Lipitor or Torvast).tw.
37. (fluvastatin or Lescol).tw.
38. (lovastatin or Mevacor or Altocor or Altoprev).tw.
39. (pitavastatin or Livalo or Pitava).tw.
40. (pravastatin or Pravachol or Selektine or Lipostat).tw.
41. (rosuvastatin or Crestor).tw.
42. (simvastatin or Zocor or Lipex).tw.
43. antihypertensive*.tw.
44. exp Antihypertensive Agents/
45. ("ACE inhibitor*" or antagonist or "angiotensin II receptor" or "beta blocker*" or "calcium channel blocker*" or "thiazide diuretic*").tw.
46. (acebutolol or adrenomedullin or alprenolol or amlodipine or atenolol or bendroflumethiazide or bepridil or betaxolol or bethanidine or bimatoprost or bisoprolol or bosentan or "bretylium tosylate" or brimonidine tartrate or bupranolol or captopril or carteolol or carvedilol or celiprolol or chlorisondamine or chlorothiazide or chlorthalidone or cilazapril or clonidine or cromakalim or cyclopenthiazide or debrisoquin or diazoxide or dihydralazine or dihydroalprenolol or diltiazem or doxazosin or enalapril or enalaprilat or eplerenone or epoprostenol or felodipine or fenoldopam or fosinopril or guanabenz or guanethidine or guanfacine or hexamethonium or "hexamethonium compound*" or hydralazine or hydrochlorothiazide or hydroflumethiazide or indapamide or indoramin or irbesartan or isradipine or kallidin or ketanserin or labetalol or latanoprost or lisinopril or losartan or mecamylamine or methyldopa or metipranolol or metolazone or metoprolol or mibefradil or minoxidil or muzolimine or nadolol or nebivolol or nicardipine or nicorandil or nimodipine or nisoldipine or nitrendipine or nitroprusside or olmesartan medoxomil or oxprenolol or pargyline or pempidine or penbutolol or "pentolinium tartrate" or perindopril or phenoxybenzamine or phentolamine or pinacidil or pindolol or piperoxan or polythiazide or prazosin or propranolol or protoveratrines or quinapril or ramipril or reserpine or rilmenidine or telmisartan or teprotide or terlipressin or ticrynafen or timolol or todralazine

For peer review only - http://bmjopen.bmj.com/site/about/guidelines.xhtml

or tolazoline or torsemide or travoprost or trichlormethiazide or trimethaphan or valsartan or "veratrum alkaloid*" or vincamine or xipamide).tw.

47. or/33-46
48. cardiovascular.tw.
49. exp Cardiovascular Diseases/
50. ((cardiac or coronary or heart) adj3 (arrest* or attack* or disease* or failure*)).tw.
51. ((heart or myocard* or transient) adj3 (infarc* or ischaemi* or ischemi*)).tw.
52. angina*.tw.
53. Angina Pectoris/
54. stroke*.tw.
55. exp Stroke/
56. or/48-55
57. 16 and 47 and 56
58. ((effectiveness or implementation or literature or map or mapping or qualitative or rapid or realist or systematic or scoping or "state of the art" or umbrella) adj2 (assessment* or overview* or review* or synthes*)).tw.
59. ("meta analy*" or metaanaly* or metasynthe* or "meta synthe*").tw.
60. ((systematic or evidence) adj1 assess*).tw.
61. (qualitative adj2 (evidence or synthes*)).tw.
62. (overarching adj2 model).tw.
63. "review* of reviews".tw.
64. systematic review.pt.
65. meta-analysis.pt.
66. or/58-65
67. 32 and 66 [Drugs that cause dependency search results]
68. 57 and 66 [Statins search results]

Notes: Date limited 2010 to date of search.

Database: PsycInfo
Host: Ovid
Issue: 1806 to August Week 1 2020
Date Searched: 11/8/2020
Searcher: SB

For peer review only

Hits: 297
Strategy

1. ((appropriate* or discontinu* or enhance* or inappropriate* or incorrect* or "in correct*" or optim* or safe or suboptim* or "sub optim*" or tapering or withdrawal) adj4 (drug* or medicine* or medication* or prescri*)).tw.
2. prescription drug misuse/
3. exp "prescribing (drugs)"/
4. ((drug* or guideline* or guidance or medicine* or medication* or patient* or prescri*) adj4 (adhere* or compliance or concordance)).tw.
5. *treatment compliance/
6. ((shared or sharing or informed) adj2 (decision* or choice*)).tw.
7. (decision adj2 (aid* or support*)).tw.
8. Decision Making/
9. ((consumer* or patient*) adj3 (involv* or participat*)).tw.
10. patient participation/
11. ("patient cent*" adj2 (approach* or care or decision* or intervention* or treatment*)).tw.
12. (behavi* adj2 chang*).ti,ab.
13. exp behavior change/
14. (restriction adj2 (policy or policies)).tw.
15. or/1-14
16. benzodiazepine*.tw.
17. (alprazolam or flunitrazepam or chlordiazepoxide or clobazam or clonazepam or diazepam or lorazepam or midazolam or nitrazepam or oxazepam or prazepam or temazepam).tw.
18. exp Benzodiazepines/
19. (antidepres* or "anti depres*").tw.
20. (serotonin or norepinephrine or noradrenaline or neurotransmitter* or dopamin* or SSRI* or SNRI* or NARI* or SARI* or NDRI* or tricyclic* or tetracyclic*).tw.
21. exp antidepressant drugs/
22. (opioid* or opiate*).tw.
23. (morphine or hydromorphone or levorphanol or meperidine or methadone or propoxyphene of codeine or pentazocine or hydrocodone or oxycodone or fentanyl or tramadol).tw.
24. exp opiates/
25. "z drug*".tw.

For peer review only - http://bmjopen.bmj.com/site/about/guidelines.xhtml

26. (zopiclone or zolpidem or zaleplon or eszopiclone).tw.
27. exp hypnotic drugs/
28. exp sedatives/
29. (gabapentin* or mirogabalin or phenibut or pregabalin).tw.
30. Gabapentin/
31. or/16-30
32. 15 and 31
33. statin*.tw.
34. ("HMG-CoA reductase inhibitor*" or "3-hydroxy-3-methyl-glutaryl-CoA reductase inhibitor*" or "3-hydroxy-3-methylglutaryl coenzyme A reductase inhibitor*" or "HMGCR inhibitor*" or "Hydroxymethylglutaryl-CoA Reductase Inhibitor*").tw.
35. statins/
36. (atorvastatin or Lipitor or Torvast).tw.
37. (fluvastatin or Lescol).tw.
38. (lovastatin or Mevacor or Altocor or Altoprev).tw.
39. (pitavastatin or Livalo or Pitava).tw.
40. (pravastatin or Pravachol or Selektine or Lipostat).tw.
41. (rosuvastatin or Crestor).tw.
42. (simvastatin or Zocor or Lipex).tw.
43. antihypertensive*.tw.
44. exp antihypertensive drugs/
45. ("ACE inhibitor*" or antagonist or "angiotensin II receptor" or "beta blocker*" or "calcium channel blocker*" or "thiazide diuretic*").tw.
46. (acebutolol or adrenomedullin or alprenolol or amlodipine or atenolol or bendroflumethiazide or bepridil or betaxolol or bethanidine or bimatoprost or bisoprolol or bosentan or "bretylium tosylate" or brimonidine tartrate or bupranolol or captopril or carteolol or carvedilol or celiprolol or chlorisondamine or chlorothiazide or chlorthalidone or cilazapril or clonidine or cromakalim or cyclopenthiazide or debrisoquin or diazoxide or dihydralazine or dihydroalprenolol or diltiazem or doxazosin or enalapril or enalaprilat or eplerenone or epoprostenol or felodipine or fenoldopam or fosinopril or guanabenz or guanethidine or guanfacine or hexamethonium or "hexamethonium compound*" or hydralazine or hydrochlorothiazide or hydroflumethiazide or indapamide or indoramin or irbesartan or isradipine or kallidin or ketanserin or labetalol or latanoprost or lisinopril or losartan or mecamylamine or methyldopa or metipranolol or metolazone or metoprolol or mibefradil or minoxidil or muzolimine or nadolol or nebivolol or nicardipine or nicorandil or nimodipine or nisoldipine or nitrendipine or nitroprusside or olmesartan medoxomil or oxprenolol or pargyline or pempidine or penbutolol or "pentolinium tartrate" or perindopril

For peer review only - http://bmjopen.bmj.com/site/about/guidelines.xhtml
For peer review only

or phenoxybenzamine or phentolamine or pinacidil or pindolol or piperoxan or polythiazide or prazosin or propranolol or protoveratrines or quinapril or ramipril or reserpine or rilmenidine or telmisartan or teprotide or terlipressin or ticrynafen or timolol or todralazine or tolazoline or torsemide or travoprost or trichlormethiazide or trimethaphan or valsartan or "veratrum alkaloid*" or vincamine or xipamide).tw.

47. or/33-46
48. cardiovascular.tw.
49. exp cardiovascular disorders/
50. ((cardiac or coronary or heart) adj3 (arrest* or attack* or disease* or failure*)).tw.
51. ((heart or myocard* or transient) adj3 (infarc* or ischaemi* or ischemi*)).tw.
52. angina*.tw.
53. Angina Pectoris/
54. stroke*.tw.
55. exp cerebral ischemia/
56. or/48-55
57. 15 and 47 and 56
58. ((effectiveness or implementation or literature or map or mapping or qualitative or rapid or realist or systematic or scoping or "state of the art" or umbrella) adj2 (assessment* or overview* or review* or synthes*)).tw.
59. ("meta analy*" or metaanaly* or metasynthe* or "meta synthe*").tw.
60. ((systematic or evidence) adj1 assess*).tw.
61. (qualitative adj2 (evidence or synthes*)).tw.
62. (overarching adj2 model).tw.
63. "review* of reviews".tw.
64. systematic review.pt.
65. meta-analysis.pt.
66. or/58-65
67. 32 and 66 [Drugs that cause dependency search results]
68. 57 and 66 [Statins search results]

Notes: Date limited 2010 to date of search.

Database: Conference Proceedings Citation Index – Science (CPCI-S); Science Citation Index (SCI)
Host: Web of Science via Clarivate Analytics
Issue: n/a
Date Searched: 11/8/2020

For peer review only - http://bmjopen.bmj.com/site/about/guidelines.xhtml

Searcher: SB
Hits: 1030
Strategy:

1. TOPIC =((appropriate* or discontinu* or enhance* or inappropriate* or incorrect* or "in correct*" or optim* or safe or suboptim* or "sub optim*" or tapering or withdrawal) near/3 (drug* or medicine* or medication* or prescri*))
2. TOPIC =((drug* or guideline* or guidance or medicine* or medication* or patient* or prescri*) near/3 (adhere* or compliance or concordance))
3. TOPIC =((shared or sharing or informed) near/1 (decision* or choice*))
4. TOPIC =((consumer* or patient*) near/2 (involv* or participat*))
5. TOPIC =("patient cent*" near/1 (approach* or care or decision* or intervention* or treatment*))
6. TOPIC =(decision near/1 (aid* or support*))
7. TOPIC =(behavi* near/1 chang*)
8. TOPIC =(restriction near/1 (policy or policies))
9. #8 OR #7 OR #6 OR #5 OR #4 OR #3 OR #2 OR #1
10. TOPIC: (benzodiazepine*)
11. TOPIC: ((alprazolam or flunitrazepam or chlordiazepoxide or clobazam or clonazepam or diazepam or lorazepam or midazolam or nitrazepam or oxazepam or prazepam or temazepam))
12. TOPIC: ((antidepres* or "anti depres*"))
13. TOPIC: ((serotonin or norepinephrine or noradrenaline or neurotransmitter* or dopamin* or SSRI* or SNRI* or NARI* or SARI* or NDRI* or tricyclic* or tetracyclic*))
14. TOPIC: ((opioid* or opiate*))
15. TOPIC: ((morphine or hydromorphone or levorphanol or meperidine or methadone or propoxyphene of codeine or pentazocine or hydrocodone or oxycodone or fentanyl or tramadol))
16. TOPIC: ("z drug*")
17. TOPIC: ((zopiclone or zolpidem or zaleplon or eszopiclone))
18. TOPIC: ((gabapentin* or mirogabalin or phenibut or pregabalin))
19. #18 OR #17 OR #16 OR #15 OR #14 OR #13 OR #12 OR #11 OR #10
20. #19 AND #9
21. TOPIC: (statin*)
22. TOPIC: ("HMG-CoA reductase inhibitor*" or "3-hydroxy-3-methyl-glutaryl-CoA reductase inhibitor*" or "3-hydroxy-3-methylglutaryl coenzyme A reductase inhibitor*" or "HMGCR inhibitor*" or "Hydroxymethylglutaryl-CoA Reductase Inhibitor*")

For peer review only - http://bmjopen.bmj.com/site/about/guidelines.xhtml

23. TOPIC: (atorvastatin or Lipitor or Torvast)
24. TOPIC: (fluvastatin or Lescol)
25. TOPIC: (lovastatin or Mevacor or Altocor or Altoprev)
26. TOPIC: (pitavastatin or Livalo or Pitava)
27. TOPIC: (pravastatin or Pravachol or Selektine or Lipostat)
28. TOPIC: (rosuvastatin or Crestor)
29. TOPIC: (simvastatin or Zocor or Lipex)
30. TOPIC: (antihypertensive*)
31. TOPIC: ("ACE inhibitor*" or antagonist or "angiotensin II receptor" or "beta blocker*" or "calcium channel blocker*" or "thiazide diuretic*")
32. TOPIC: (acebutolol or adrenomedullin or alprenolol or amlodipine or atenolol or bendroflumethiazide or bepridil or betaxolol or bethanidine or bimatoprost or bisoprolol or bosentan or "bretylium tosylate" or brimonidine tartrate or bupranolol or captopril or carteolol or carvedilol or celiprolol or chlorisondamine or chlorothiazide or chlorthalidone or cilazapril or clonidine or cromakalim or cyclopenthiazide or debrisoquin or diazoxide or dihydralazine or dihydroalprenolol or diltiazem or doxazosin or enalapril or enalaprilat or eplerenone or epoprostenol or felodipine or fenoldopam or fosinopril or guanabenz or guanethidine or guanfacine or hexamethonium or "hexamethonium compound*" or hydralazine or hydrochlorothiazide or hydroflumethiazide or indapamide or indoramin or irbesartan or isradipine or kallidin or ketanserin or labetalol or latanoprost or lisinopril or losartan or mecamylamine or methyldopa or metipranolol or metolazone or metoprolol or mibefradil or minoxidil or muzolimine or nadolol or nebivolol or nicardipine or nicorandil or nimodipine or nisoldipine or nitrendipine or nitroprusside or olmesartan medoxomil or oxprenolol or pargyline or pempidine or penbutolol or "pentolinium tartrate" or perindopril or phenoxybenzamine or phentolamine or pinacidil or pindolol or piperoxan or polythiazide or prazosin or propranolol or protoveratrines or quinapril or ramipril or reserpine or rilmenidine or telmisartan or teprotide or terlipressin or ticrynafen or timolol or todralazine or tolazoline or torsemide or travoprost or trichlormethiazide or trimethaphan or valsartan or "veratrum alkaloid*" or vincamine or xipamide)
33. #32 OR #31 OR #30 OR #29 OR #28 OR #27 OR #26 OR #25 OR #24 OR #23 OR #22 OR #21
34. TOPIC =((cardiac or coronary or heart) near/2 (arrest* or attack* or disease* or failure*))
35. TOPIC: (cardiovascular)
36. TS=((heart or myocard* or transient) near/2 (infarc* or ischaemi* or ischemi*))
37. TOPIC =angina*
38. TOPIC =stroke*
39. #38 OR #37 OR #36 OR #35 OR #34

For peer review only

For peer review only - http://bmjopen.bmj.com/site/about/guidelines.xhtml

40. TOPIC =((effectiveness or implementation or literature or map or mapping or qualitative or rapid or realist or systematic or scoping or "state of the art" or umbrella) near/1 (assessment* or overview* or review* or synthes*))
41. TOPIC =("meta analy*" or metaanaly* or metasynthe* or "meta synthe*")
42. TOPIC =((systematic or evidence) near/0 assess*)
43. TOPIC =(qualitative near/1 (evidence or synthes*))
44. TOPIC =(overarching near/1 model)
45. TOPIC ="review* of reviews"
46. #45 OR #44 OR #43 OR #42 OR #41 OR #40
47. #46 AND #20 [Drugs that cause dependency search results]
48. #46 AND #39 AND #33 AND #9 [Statins search results]

Table 1: Drugs that can cause dependency and antidepressant search results

Database	Hits
**Cochrane Database of Systematic Reviews**	111
**CINAHL**	372
**Epistemonikos**	56
**medRxiv**	87
**Embase**	1776
**HMIC**	10
**MEDLINE**	875
**PsycInfo**	297
**Web of Science (CPCI-S/SCI)**	1030
**TOTAL RECORDS**	**4614**
**DUPLICATE RECORDS**	**1898**
**UNIQUE RECORDS**	**2716**

Statins

Database: Cochrane Database of Systematic Reviews
Host: Cochrane Library
Issue: Issue 8 of 12, August 2020
Date Searched: 10/8/2020
Searcher: SB
Hits: 33
Strategy: see line 57 of Cochrane Database search above.
Notes: date limited 2010 to date of search.

Database: CINAHL
Host: EBSCO
Issue: n/a
Date Searched: 10/8/2020
Searcher: SB

For peer review only - http://bmjopen.bmj.com/site/about/guidelines.xhtml

Hits: 173
Strategy: see line 66 of CINAHL search above.
Notes: Date limited 2010 to date of search.

Database: Epistemonikos
Host: https://www.epistemonikos.org/en/
Issue: n/a
Date Searched: 10/8/2020
Searcher: SB
Hits: 16
Strategy:

1. title:(prescription* OR prescribing OR prescribe*) OR abstract:(prescription* OR prescribing OR prescribe*)
2. title:(optimi* OR inappropriat* OR enhance*) OR abstract:(optimi* OR inappropriat* OR enhance*)
3. title:(statin*) OR abstract:(statin*) OR title:(antihypertensive*) OR abstract:(antihypertensive*)
4. 1 AND 2 AND 3 [Statins search results]

Notes: Date limited 2010 to 2020 and limited to systematic reviews publication type.

Database: medRxiv
Host: https://www.medrxiv.org/
Issue: n/a
Date Searched: 10/8/2020
Searcher: SB
Hits: 8
Strategy:

optimi* and prescri* and statin*	7 hits
optimi* and prescri* and antihypertensive*	1 hit

Notes: Date limited 2010 to date of search.

Database: Embase
Host: Ovid
Issue: 1974 to 2020 August 07
Date Searched: 10/8/2020
Searcher: SB
Hits: 903
Strategy: see line 69 of Embase search above.
Notes: Date limited 2010 to date of search.

Database: HMIC
Host: Ovid
Issue: 1979 to May 2020
Date Searched: 10/8/2020

For peer review only - http://bmjopen.bmj.com/site/about/guidelines.xhtml

Searcher: SB
Hits: 0
Strategy: see line 68 of MEDLINE search strategy above.
Notes: Date limited 2010 to date of search.

Database: MEDLINE ALL
Host: Ovid
Issue: 1946 to August 07, 2020
Date Searched: 10/8/2020
Searcher: SB
Hits: 371
Strategy: see line 68 of MEDLINE search strategy above.
Notes: Date limited 2010 to date of search.

Database: PsycInfo
Host: Ovid
Issue: 1806 to August Week 1 2020
Date Searched: 10/8/2020
Searcher: SB
Hits: 17
Strategy: see line 68 of PsycInfo search strategy above.
Notes: Date limited 2010 to date of search.

Database: Conference Proceedings Citation Index – Science (CPCI-S); Science Citation Index (SCI)
Host: Web of Science via Clarivate Analytics
Issue: n/a
Date Searched: 10/8/2020
Searcher: SB
Hits: 638
Strategy: see line 48 of CPCI-S/SCI search strategy above.

Table 2: Statin search results

Database	Hits
**Cochrane Database of Systematic Reviews**	33
**CINAHL**	173
**Epistemonikos**	16
**medRxiv**	8
**EMBASE**	903
**HMIC**	0
**MEDLINE**	371
**PsycInfo**	17
**Web of Science (CPCI-S/SCI)**	638
**TOTAL RECORDS**	**2159**
**DUPLICATE RECORDS**	**714**
**UNIQUE RECORDS**	**1445**

<u>Website searches</u>

For peer review only - http://bmjopen.bmj.com/site/about/guidelines.xhtml

For peer review only

DCDs

Website: Royal Pharmaceutical Society Library Catalogue
URL: https://rpsgb.koha-ptfs.co.uk/
Date Searched: 21/10/2020
Searcher: SB
Hits: 73 hits
Strategy:

Advanced search setting.

Keyword: opioid* OR

Keyword: benzodiazepine* OR

Keyword: antidepressant* OR

Keyword: gabapentinoid*

Website: Royal College of Physicians Library Catalogue
URL: https://rcp.soutron.net/Portal/Default/en-GB/Search/SimpleSearch
Date Searched: 21/10/2020
Searcher: SB
Hits: 6
Strategy:

All fields: opioid OR opioids OR benzodiazepine OR benzodiazepines OR antidepressant OR antidepressants OR gabapentinoid OR gabapentinoids

Website: Royal College of Psychiatrists Library Catalogue
URL: https://rcpsych.soutron.net/Portal/Default/en-GB/Search/SimpleSearch
Date Searched: 21/10/2020
Searcher: SB
Hits: 20
Strategy:

opioid* OR benzodiazepine* OR antidepressant* OR gabapentinoid*

Website: Mind
URL: https://www.mind.org.uk/
Date Searched: 21/10/2020
Searcher: SB
Hits: 0
Strategy: Browsed website for relevant studies

Website: Mental Health UK
URL: https://mentalhealth-uk.org/

For peer review only - http://bmjopen.bmj.com/site/about/guidelines.xhtml

For peer review only

Date Searched: 21/10/2020
Searcher: SB
Hits: 0
Strategy: Browsed website for relevant studies

Website: Priory Group
URL: https://www.priorygroup.com/
Date Searched: 21/10/2020
Searcher: SB
Hits: 0
Strategy: Browsed website for relevant studies

Statins

Website: Royal Pharmaceutical Society Library Catalogue
URL: https://rpsgb.koha-ptfs.co.uk/
Date Searched: 21/10/2020
Searcher: SB
Hits: 13
Strategy:

Advanced search setting.

Keyword:	statin*	OR
Keyword:	"ace inhibitor*"	OR
Keyword:	antihypertensive*	

Website: Royal College of Physicians Library Catalogue
URL: https://rcp.soutron.net/Portal/Default/en-GB/Search/SimpleSearch
Date Searched: 21/10/2020
Searcher: SB
Hits: 6
Strategy:

All fields: statin OR statins OR "ace inhibitor" OR "ace inhibitors" OR antihypertensive OR antihypertensives

Website: British Cardiovascular Society
URL: https://www.britishcardiovascularsociety.org/
Date Searched: 21/10/2020
Searcher: SB
Hits: 0
Strategy: Browsed website for relevant studies.

For peer review only - http://bmjopen.bmj.com/site/about/guidelines.xhtml
For peer review only

Website: European Society of Cardiology
URL: https://www.escardio.org/
Date Searched: 21/10/2020
Searcher: SB
Hits: 0
Strategy: Browsed website for relevant studies

Website: British and Irish Hypertension Society
URL: https://bihsoc.org/
Date Searched: 21/10/2020
Searcher: SB
Hits: 0
Strategy: Browsed website for relevant studies

Website: British Heart Foundation
URL: https://www.bhf.org.uk/
Date Searched: 21/10/2020
Searcher: SB
Hits: 31 (sum total of each keyword search)
Strategy: Searched for keywords “statin”, “ace inhibitor” or “antihypertensive” (one term per search) and viewed the publications and research tabs.

Website: Heart Research UK
URL: https://heartresearch.org.uk/
Date Searched: 21/10/2020
Searcher: SB
Hits: 0
Strategy: Browsed website for relevant studies

For peer review only

APPENDIX B: RESULTS FROM UPDATE SEARCHES

Summary of systematic reviews (n=12) identified for inclusion following update searches conducted 2nd August 2022.

Author	Year	CEESAT rating (n positive of 4 items)	Topic [Type of evidence]	Medication of interest	Care pathway
Bond	2021	2	Evaluating intervention to optimise patient adherence to a medication [Quantitative]	Statins	Maintaining treatment
Coronado-Vázquez	2020	3	Evaluating intervention to optimise patient adherence to a medication [Quantitative]	Statins, Antihypertensives	Maintaining treatment
Guzman-Tordecilla*	2020	1	Evaluating intervention to optimise patient adherence to a medication [Quantitative]	Antihypertensives	Maintaining treatment
Hong	2022	2	Evaluating intervention to optimise patient adherence to a medication [Quantitative]	Antihypertensives	Maintaining treatment
Nili	2020	4	Evaluating intervention to optimise patient adherence to a medication [Quantitative]	Antihypertensives	Maintaining treatment
Palmer*	2021	4	Evaluating intervention to optimise patient adherence to a medication [Quantitative]	Statins, Antihypertensives	Maintaining treatment

For peer review only

Parati	2021	3	Evaluating intervention to optimise patient adherence to a medication [Quantitative]	Antihypertensives	Maintaining treatment
Qadi	2020	3	Prescriber, patient, and/or family/carer views of issues relating to prescribing and/or adherence to medications of interest [Qualitative]	Statins, Antihypertensives	Pre-treatment/ initiation,
Reston	2020	3	Evaluating intervention to optimise patient adherence to a medication [Quantitative]	Statin	Maintaining treatment
Van Truong	2021	3	Evaluating intervention to optimise patient adherence to a medication [Quantitative]	Antihypertensives	Maintaining treatment
Vervloet	2020	3	Evaluating intervention to optimise patient adherence to a medication [Quantitative]	Statin Antihypertensives	Maintaining treatment
Xie	2022	3	Evaluating intervention to optimise prescribing [Quantitative]	Statin, Antihypertensives	Pre-treatment/ initiation, Maintaining treatment

*Updated of review identified by original searches, CEESAT=Collaboration for Environmental Evidence Synthesis Assessment Tool, N=Number

For peer review only - http://bmjopen.bmj.com/site/about/guidelines.xhtml

APPENDIX C: REASONS FOR EXCLUSION AT FULL TEXT

Reference	Reason for exclusion
Abstracts from Professional Poster Presentations at AMCP's 2010 Educational Conference. *Journal of Managed Care Pharmacy* 2010;**16**.	Abstract proceedings
The Canadian Association for Population Therapeutics. November 17th - 19th, 2013 Toronto, Ontario. "Healthcare Cost, Quality, and Policy: Driving Stakeholder Innovation in Process and Practice". *Journal of Population Therapeutics and Clinical Pharmacology* 2014;**21**:e116.	Abstract proceedings
Transcatheter Cardiovascular Therapeutics Asia Pacific, 19th Cardiovascular Summit: TCTAP 2014. *Journal of the American College of Cardiology* 2014;**63**.	Abstract proceedings
Abstracts of Papers Presented at the Health Services Research and Pharmacy Practice Conference, HSRPP 2016. *International Journal of Pharmacy Practice* 2016;**24**.	Abstract proceedings
12th Annual Scientific Meeting of Indonesian Society of Hypertension, INASH. *Journal of Hypertension* 2018;**36**.	Abstract proceedings
Selected abstracts from the EGPRN meeting in Tampere, Finland, 9–12 May 2019. *European Journal of General Practice* 2019;**25**:164-75.	Abstract proceedings
46th ESAO Congress 3-7 September 2019 Hannover, Germany Abstracts. *International Journal of Artificial Organs* 2019;**42**:386-474.	Abstract proceedings
AbuDagga A, Resnick HE, Alwan M. Impact of blood pressure telemonitoring on hypertension outcomes: a literature review. *Telemed J E Health* 2010;**16**:830-8. https://doi.org/10.1089/tmj.2010.0015	Not SR
Akeroyd JM, Chan WJ, Kamal AK, Palaniappan L, Virani SS. Adherence to cardiovascular medications in the South Asian population: A systematic review of current evidence and future directions. *World J Cardiol* 2015;**7**:938-47. https://doi.org/10.4330/wjc.v7.i12.938	Not SR
Al AlShaikh S, Quinn T, Dunn W, Dawson J. Interventions to Enhance Adherence to Secondary Preventative Medication After Stroke a Systematic Review and Meta-Analysis. *Stroke* 2016;**47**.	Abstract
Alford DP. Chronic back pain with possible prescription opioid misuse. *JAMA* 2013;**309**:919-25. https://doi.org/10.1001/jama.2013.522	Not SR
AlGhurair SA, Hughes CA, Simpson SH, Guirguis LM. A systematic review of patient self-reported barriers of adherence to antihypertensive medications using the world health organization multidimensional adherence model. *J Clin Hypertens (Greenwich)* 2012;**14**:877-86. https://doi.org/10.1111/j.1751-7176.2012.00699.x	Not SR

For peer review only

Alkhouli M, Noseworthy PA, Rihal CS, Holmes DR, Jr. Stroke Prevention in Nonvalvular Atrial Fibrillation: A Stakeholder Perspective. *J Am Coll Cardiol* 2018;**71**:2790-801. https://doi.org/10.1016/j.jacc.2018.04.013	Not SR
Almeida JT, Esteves AL, Martins F, Palma I. [Approach to Patients with Statin Intolerance: Evidence-Based Review]. *Acta Med Port* 2020;**33**:49-57. https://doi.org/10.20344/amp.10376	Non-English language (Duplicated excluded from update search)
AlShaikh SA, Quinn T, Dunn W, Dawson J. Interventions to enhance adherence to secondary preventative medication after stroke a systematic review and meta-analysis. *Stroke* 2016;**47**.	Duplicate
Anderson SL, Marrs JC. A Review of the Role of the Pharmacist in Heart Failure Transition of Care. *Adv Ther* 2018;**35**:311-23. https://doi.org/10.1007/s12325-018-0671-7	Not SR
Anonymous. Review of: Fuller RH et al. Improving adherence to medicines in people with heart disease: a systematic review. Heart. *Drug Ther Bull* 2018;**56**:114. https://doi.org/10.1136/dtb.2018.10.000021	Not SR
Armstrong SO, Little RA. Cost effectiveness of interventions to improve adherence to statin therapy in ASCVD patients in the United States. *Patient Prefer Adherence* 2019;**13**:1375-89. https://doi.org/10.2147/PPA.S213258	Not SR
Banerjee A, Khandelwal S, Nambiar L, Saxena M, Peck V, Moniruzzaman M*, et al.* Health system barriers and facilitators to medication adherence for the secondary prevention of cardiovascular disease: a systematic review. *Open Heart* 2016;**3**:e000438. https://doi.org/10.1136/openhrt-2016-000438	Quantitative focus
Basaraba JE, Picard M, George-Phillips K, Mysak T. Pharmacists as Care Providers for Stroke Patients: A Systematic Review. *Can J Neurol Sci* 2018;**45**:49-55. https://doi.org/10.1017/cjn.2017.233	Not SR
Bramlage P, Sims H, Minguet J, Ferrero C. The polypill: An effective approach to increasing adherence and reducing cardiovascular event risk. *Eur J Prev Cardiol* 2017;**24**:297-310. https://doi.org/10.1177/2047487316674817	Not SR
Brito JP, Montori VM. Reinitiation of statins after statin-associated musculoskeletal symptoms: a patient-centered approach. *Circ Cardiovasc Qual Outcomes* 2013;**6**:243-7. https://doi.org/10.1161/CIRCOUTCOMES.111.000039	Not SR
Buck HG, McGhee S, Polo RL, Zambroski C. Hypercholesterolemia Management in Older Adults: A Scoping Review of Recent Evidence. *J Gerontol Nurs* 2019;**45**:31-42. https://doi.org/10.3928/00989134-20190211-04	Quantitative focus
Calleja Rivero JL, Zerpa F, Rivera L. Fixed-dose versus separate drug combinations for antihypertensive treatment: literature review. *Medwave* 2016;**16**:e6549. https://doi.org/10.5867/medwave.2016.08.6549	Not SR

For peer review only - http://bmjopen.bmj.com/site/about/guidelines.xhtml

Campos K, Sheth S, Coulter SA. Hypertension Treatment ACCORDing to SPRINT. *Tex Heart Inst J* 2016;**43**:324-7. https://doi.org/10.14503/THIJ-16-5908	Not SR
Chan DK, O'Rourke F, Shen Q, Mak JC, Hung WT. Meta-analysis of the cardiovascular benefits of intensive lipid lowering with statins. *Acta Neurol Scand* 2011;**124**:188-95. https://doi.org/10.1111/j.1600-0404.2010.01450.x	Quantitative focus
Chapman RH, Ferrufino CP, Kowal SL, Classi P, Roberts CS. The cost and effectiveness of adherence-improving interventions for antihypertensive and lipid-lowering drugs*. *Int J Clin Pract* 2010;**64**:169-81. https://doi.org/10.1111/j.1742-1241.2009.02196.x	Not SR
Chee YJ, Chan HH, Tan NC. Understanding patients' perspective of statin therapy: can we design a better approach to the management of dyslipidaemia? A literature review. *Singapore Med J* 2014;**55**:416-21. https://doi.org/10.11622/smedj.2014099	Not SR
Choi H-I, Han KH. The ideal management of blood cholesterol: a review of the updated 2013 ACC/AHA cholesterol guideline for reducing cardiovascular risk. *Journal of the Korean Medical Association* 2014;**57**:857-65. https://doi.org/10.5124/jkma.2014.57.10.857	Non-English language
Clarkesmith DE, Pattison HM, Khaing PH, Lane DA. Educational and behavioural interventions for anticoagulant therapy in patients with atrial fibrillation. *Cochrane Database of Systematic Reviews* 2017; 10.1002/14651858.CD008600.pub3. https://doi.org/10.1002/14651858.CD008600.pub3	Drug not relevant
Coleman CI, Roberts MS, Sobieraj DM, Lee S, Alam T, Kaur R. Effect of dosing frequency on chronic cardiovascular disease medication adherence. *Curr Med Res Opin* 2012;**28**:669-80. https://doi.org/10.1185/03007995.2012.677419	Not SR
Conn VS, Ruppar TM, Chase JD. Blood pressure outcomes of medication adherence interventions: systematic review and meta-analysis. *J Behav Med* 2016;**39**:1065-75. https://doi.org/10.1007/s10865-016-9730-1	Quantitative focus
Cooke CE, Xing S, Gale SE, Peters S. Initial non-adherence to antihypertensive medications in the United States: a systematic literature review. Journal of human hypertension 2022;36:3-13. https://doi.org/https://dx.doi.org/10.1038/s41371-021-00549-w	Not SR
Crisafulli S, Luxi N, Coppini R, Capuano A, Scavone C, Zinzi A, et al. Anti-hypertensive drugs deprescribing: an updated systematic review of clinical trials. BMC family practice 2021;22:208. https://doi.org/https://dx.doi.org/10.1186/s12875-021-01557-y	Not SR
Cutrona SL, Choudhry NK, Fischer MA, Servi A, Liberman JN, Brennan TA*, et al.* Modes of delivery for interventions to improve cardiovascular medication adherence. *Am J Manag Care* 2010;**16**:929-42.	Not SR

For peer review only

de Barra M, Scott CL, Scott NW, Johnston M, de Bruin M, Nkansah N*, et al.* Pharmacist services for non-hospitalised patients. *Cochrane Database Syst Rev* 2018;**9**:CD013102. https://doi.org/10.1002/14651858.CD013102	Quantitative focus
Dobremez V, Ruel JH, Hugon A, Gay S, Berlioz J, Pineau-Blondel E. Contraindicated drugs in myasthenia gravis patients: Elaboration of a tool to optimize drug therapy. *International Journal of Clinical Pharmacy* 2015;**37**:236-7. https://doi.org/10.1007/s11096-014-0039-2	Abstract
Dunn BL, Teusink AC, Taber DJ, Hemstreet BA, Uber LA, Weimert NA. Management of hypertension in renal transplant patients: a comprehensive review of nonpharmacologic and pharmacologic treatment strategies. *Ann Pharmacother* 2010;**44**:1259-70. https://doi.org/10.1345/aph.1P004	Not SR
Elley CR, Gupta AK, Webster R, Selak V, Jun M, Patel A*, et al.* The efficacy and tolerability of 'polypills': meta-analysis of randomised controlled trials. *PLoS One* 2012;**7**:e52145. https://doi.org/10.1371/journal.pone.0052145	Quantitative focus
Elnaem MH, Rosley NFF, Alhifany AA, Elrggal ME, Cheema E. Impact of Pharmacist-Led Interventions on Medication Adherence and Clinical Outcomes in Patients with Hypertension and Hyperlipidemia: A Scoping Review of Published Literature. *J Multidiscip Healthc* 2020;**13**:635-45. https://doi.org/10.2147/JMDH.S257273	Not SR
Engel J, Damen NL, van der Wulp I, de Bruijne MC, Wagner C. Adherence to Cardiac Practice Guidelines in the Management of Non-ST-Elevation Acute Coronary Syndromes: A Systematic Literature Review. *Curr Cardiol Rev* 2017;**13**:3-27. https://doi.org/10.2174/1573403x12666160504100025	Quantitative focus
Gale N, Marshall T, Bramley G. Starting and staying on preventive medication for cardiovascular disease. *Curr Opin Cardiol* 2012;**27**:533-41. https://doi.org/10.1097/HCO.0b013e328356dae5	Not SR
Gandapur Y, Kianoush S, Kelli HM, Misra S, Urrea B, Blaha MJ*, et al.* The role of mHealth for improving medication adherence in patients with cardiovascular disease: a systematic review. *Eur Heart J Qual Care Clin Outcomes* 2016;**2**:237-44. https://doi.org/10.1093/ehjqcco/qcw018	Not SR
Gellad WF, Grenard JL, Marcum ZA. A systematic review of barriers to medication adherence in the elderly: looking beyond cost and regimen complexity. *Am J Geriatr Pharmacother* 2011;**9**:11-23. https://doi.org/10.1016/j.amjopharm.2011.02.004	Quantitative focus
Georgiopoulos G, Kollia Z, Katsi V, Oikonomou D, Tsioufis C, Tousoulis D. Nurse's Contribution to Alleviate Non-adherence to Hypertension Treatment. *Curr Hypertens Rep* 2018;**20**:65. https://doi.org/10.1007/s11906-018-0862-2	Not SR

For peer review only - http://bmjopen.bmj.com/site/about/guidelines.xhtml

Glynn L, Fahey T. Cardiovascular medication: improving adherence. *BMJ Clin Evid* 2011;**2011**.	Not SR
Golubev S. Compliance measurement-guided medication management programs in hypertension: A health technology assessment. *Journal of Hypertension* 2010;**28**:e269.	Quantitative focus
Golubev S, Fedoseyeva I, Johri M. Compliance measurement-guided medication management programs in hypertension: A systematic review. *Basic and Clinical Pharmacology and Toxicology* 2011;**109**:109-10. http://dx.doi.org/10.1111/j.1742-7843.2011.00722.x	Abstract
Gwadry-Sridhar FH, Manias E, Lal L, Salas M, Hughes DA, Ratzki-Leewing A*, et al.* Impact of interventions on medication adherence and blood pressure control in patients with essential hypertension: a systematic review by the ISPOR medication adherence and persistence special interest group. *Value Health* 2013;**16**:863-71. https://doi.org/10.1016/j.jval.2013.03.1631	Not SR
Hopper I, Samuel R, Tonkin A, Krum H. The effects of discontinuing medications in stable systolic heart failure-systematic review and meta-analysis. *Heart Lung and Circulation* 2012;**21**:S95. http://dx.doi.org/10.1016/j.hlc.2012.05.241	Abstract
Imbeault B, Vallee M. Single-Pill Combinations in the Treatment of Hypertension in Adults: Beyond Convenience. *Can J Diabetes* 2018;**42**:205-8. https://doi.org/10.1016/j.jcjd.2018.01.011	Not SR
Iwelunmor J, Airhihenbuwa CO, Cooper R, Tayo B, Plange-Rhule J, Adanu R*, et al.* Prevalence, determinants and systems-thinking approaches to optimal hypertension control in West Africa. *Global Health* 2014;**10**:42. https://doi.org/10.1186/1744-8603-10-42	Not SR
Jamshidnezhad A, Kabootarizadeh L, Hoseini SM. The Effects of Smartphone Applications on Patients Self-care with Hypertension: A Systematic Review Study. *Acta Inform Med* 2019;**27**:263-7. https://doi.org/10.5455/aim.2019.27.263-267	Not SR
Jones LK, Gionfriddo MR, Tilberry S, Frisbie L, Gregor C, Gidding S*, et al.* Implementation strategies to improve statin utilization in individuals with hypercholesterolemia: A systematic review. *JACCP Journal of the American College of Clinical Pharmacy* 2020;**3**:365. http://dx.doi.org/10.1002/jac5.1204	Abstract
Jornten-Karlsson M, Pintat S, Molloy-Bland M, Berg S, Ahlqvist M. Patient-Centered Interventions to Improve Adherence to Statins: A Narrative Synthesis of Systematically Identified Studies. *Drugs* 2016;**76**:1447-65. https://doi.org/10.1007/s40265-016-0640-x	Not SR
Ju A, Hanson CS, Banks E, Korda R, Craig JC, Usherwood T*, et al.* Patient beliefs and attitudes to taking statins: systematic review of qualitative studies. *Br J Gen Pract* 2018;**68**:e408-e19. https://doi.org/10.3399/bjgp18X696365	Not SR

For peer review only - http://bmjopen.bmj.com/site/about/guidelines.xhtml

Ko DT, Jackevicius CA. Stopping beta-Blockers After Myocardial Infarction: Not So Fast! *Circ Cardiovasc Qual Outcomes* 2018;**11**:e004678. https://doi.org/10.1161/CIRCOUTCOMES.118.004678	Not SR	
Krack G. EXPLANATIONS FOR THE DIFFERENCES BETWEEN EFFECTS ON ADHERENCE OF VALUE-BASED HEALTH INSURANCE DESIGNS: A SYSTEMATIC REVIEW AND META-ANALYSIS. *Value in Health* 2018;**21**:S155. http://dx.doi.org/10.1016/j.jval.2018.09.924	Abstract	
Krueger K, Griese-Mammen N, Schubert I, Kieble M, Botermann L, Laufs U*, et al.* In search of a standard when analyzing medication adherence in patients with heart failure using claims data: a systematic review. *Heart Fail Rev* 2018;**23**:63-71. https://doi.org/10.1007/s10741-017-9656-x	Quantitative focus	
Kwon A, Kim GH. Single-pill Combination Therapy of Azilsartan Medoxomil/Chlorthalidone for Treatment of Hypertension: A Systematic Review. *Clin Ther* 2020;**42**:1390-403. https://doi.org/10.1016/j.clinthera.2020.05.015	Not SR (Duplicate excluded in update search)	
Lane D, Patel P, Khunti K, Gupta P. Objective measures of non-adherence in cardiometabolic diseases: a review focused on urine biochemical screening. *Patient Prefer Adherence* 2019;**13**:537-47. https://doi.org/10.2147/PPA.S162215	Not SR	
Lee JL, Maciejewski M, Raju S, Shrank WH, Choudhry NK. Value-based insurance design: quality improvement but no cost savings. *Health Aff (Millwood)* 2013;**32**:1251-7. https://doi.org/10.1377/hlthaff.2012.0902	Not SR	
Lennon O, Blake C, Booth J, Pollock A, Lawrence M. Interventions for behaviour change and self-management in stroke secondary prevention: protocol for an overview of reviews. *Syst Rev* 2018;**7**:231. https://doi.org/10.1186/s13643-018-0888-1	Protocol	
Liu HL, Chen XY, Li JR, Su SW, Ding T, Shi CX*, et al.* Efficacy and Safety of Pulmonary Arterial Hypertension-specific Therapy in Pulmonary Arterial Hypertension: A Meta-analysis of Randomized Controlled Trials. *Chest* 2016;**150**:353-66. https://doi.org/10.1016/j.chest.2016.03.031	Quantitative focus	
Logue J, Al-Ghibiwi H, Alamri AA, Preiss D. Systematic review of studies exploring reasons for statin non-adherence and of randomised controlled trials of interventions to improve adherence. *Atherosclerosis* 2015;**241**:e52.	Abstract	
Madhavan MV, Gersh BJ, Alexander KP, Granger CB, Stone GW. Coronary Artery Disease in Patients >/=80 Years of Age. *J Am Coll Cardiol* 2018;**71**:2015-40. https://doi.org/10.1016/j.jacc.2017.12.068	Not SR	
Marcum ZA, Handler SM, Boyce R, Gellad W, Hanlon JT. Medication misadventures in the elderly: a year in review. *Am J Geriatr Pharmacother* 2010;**8**:77-83. https://doi.org/10.1016/j.amjopharm.2010.02.002	Not SR	

For peer review only

For peer review only - http://bmjopen.bmj.com/site/about/guidelines.xhtml

Matthes J, Albus C. Improving adherence with medication: a selective literature review based on the example of hypertension treatment. *Dtsch Arztebl Int* 2014;**111**:41-7. https://doi.org/10.3238/arztebl.2014.0041	Not SR	
Mendes Soares M, Oliveira LeÆo e Silva L, Alberto Dias C, Maria Rodrigues S, Jorge Machado C. ADESAO DO IDOSO AO TRATAMENTO DA HIPERTENSAO ARTERIAL SISTEMICA: REVISAO INTEGRATIVA. *Cogitare Enfermagem* 2012;**17**:114-50.	Non-English language	
Molloy GJ, O'Carroll RE, Witham MD, McMurdo ME. Interventions to enhance adherence to medications in patients with heart failure: a systematic review. *Circ Heart Fail* 2012;**5**:126-33. https://doi.org/10.1161/CIRCHEARTFAILURE.111.964569	Not SR	
Morgado M, Castanheira L, Verde I, Castelo-Branco M. Pharmacist interventions to enhance patient adherence to antihypertensive medication: A systematic review. *Journal of Clinical Hypertension* 2010;**12**:A112-A3. http://dx.doi.org/10.1111/j.1751-7176.2010.00282.x	Abstract	
Morgado MP, Morgado SR, Mendes LC, Pereira LJ, Castelo-Branco M. Pharmacist interventions to enhance blood pressure control and adherence to antihypertensive therapy: Review and meta-analysis. *Am J Health Syst Pharm* 2011;**68**:241-53. https://doi.org/10.2146/ajhp090656	Not SR	
Morrissey EC, Durand H, Nieuwlaat R, Navarro T, Haynes RB, Walsh JC*, et al.* Effectiveness and content analysis of interventions to enhance medication adherence in hypertension: a systematic review and meta-analysis protocol. *Syst Rev* 2016;**5**:96. https://doi.org/10.1186/s13643-016-0278-5	Protocol	
Na. Selected Abstracts from the National Congress of the Italian Society of Hypertension (SIIA 2010). *High Blood Pressure & Cardiovascular Prevention* 2010;**17**:131-90. https://doi.org/10.2165/11311900-000000000-00000	Quantitative focus	
Naderi SH, Bestwick JP, Wald DS. Adherence to drugs that prevent cardiovascular disease: meta-analysis on 376,162 patients. *Am J Med* 2012;**125**:882-7 e1. https://doi.org/10.1016/j.amjmed.2011.12.013	Not SR	
Nguyen E, Sobieraj DM. The impact of appointment-based medication synchronization on medication taking behaviour and health outcomes: A systematic review. *J Clin Pharm Ther* 2017;**42**:404-13. https://doi.org/10.1111/jcpt.12554	Not SR	
Nguyen T, Gurwitz J. Paper Abstract. *Journal of the American Geriatrics Society* 2016;**64**:S1-S311. https://doi.org/10.1111/jgs.14231	Quantitative focus	
Ni Z, Dardas L, Wu B, Shaw R. Cardioprotective medication adherence among patients with coronary heart disease in China: a systematic review. *Heart Asia* 2019;**11**:e011173. https://doi.org/10.1136/heartasia-2018-011173	Not SR	

For peer review only - http://bmjopen.bmj.com/site/about/guidelines.xhtml

Nielsen JO, Shrestha AD, Neupane D, Kallestrup P. Non-adherence to anti-hypertensive medication in low- and middle-income countries: a systematic review and meta-analysis of 92443 subjects. *J Hum Hypertens* 2017;**31**:14-21. https://doi.org/10.1038/jhh.2016.31	Quantitative focus	
Nieuwlaat R, Connolly SJ, MacKay JA, Weise-Kelly L, Navarro T, Wilczynski NL*, et al.* Computerized clinical decision support systems for therapeutic drug monitoring and dosing: A decision maker-researcher partnership systematic review. *Circulation: Cardiovascular Quality and Outcomes* 2011;**4**.	Drug not relevant	
Njie GJ, Mukhtar Q, Finnie RKC, Dhakal-Acharya S, Thota AB, Hopkins DP*, et al.* Effectiveness of reducing out-of-pocket costs for medications to treat hypertension and hyperlipidemia: A community guide systematic review. *Journal of the American Society of Hypertension* 2014;**8**:e124-e5. http://dx.doi.org/10.1016/j.jash.2014.03.285	Abstract	
Ofori-Asenso R, Zoungas S, Liew D. Reinitiation of Statin Therapy After Discontinuation: A Meta-analysis. *Mayo Clin Proc* 2018;**93**:666-8. https://doi.org/10.1016/j.mayocp.2018.01.011	Quantitative focus	
Oosterom-Calo R, van Ballegooijen AJ, Terwee CB, te Velde SJ, Brouwer IA, Jaarsma T*, et al.* Determinants of adherence to heart failure medication: a systematic literature review. *Heart Fail Rev* 2013;**18**:409-27. https://doi.org/10.1007/s10741-012-9321-3	Quantitative focus	
Osborn D, Burton A, Walters K, Atkins L, Barnes T, Blackburn R*, et al. Primary care management of cardiovascular risk for people with severe mental illnesses: the Primrose research programme including cluster RCT*. Southampton (UK): NIHR Journals Library; 2019 Apr. http://doi.org/ 10.3310/pgfar07020	Quantitative focus	
Perera R, McFadden E, McLellan J, Lung T, Clarke P, Perez T*, et al.* Optimal strategies for monitoring lipid levels in patients at risk or with cardiovascular disease: a systematic review with statistical and cost-effectiveness modelling. *Health Technol Assess* 2015;**19**:1-401, vii-viii. https://doi.org/10.3310/hta191000	Quantitative focus	
Phan K, Gomez YH, Elbaz L, Daskalopoulou SS. Statin treatment non-adherence and discontinuation: clinical implications and potential solutions. *Curr Pharm Des* 2014;**20**:6314-24. https://doi.org/10.2174/1381612820666140620162629	Quantitative focus	
Pinho S, Cruz M, Ferreira F, Ramalho A, Sampaio R. Improving medication adherence in hypertensive patients: A scoping review. Preventive medicine 2021;146:106467. https://doi.org/https://dx.doi.org/10.1016/j.ypmed.2021.106467	Not SR	
Polinski JM, Donohue JM, Kilabuk E, Shrank WH. Medicare Part D's effect on the under- and overuse of medications: a systematic review. *J Am Geriatr Soc* 2011;**59**:1922-33. https://doi.org/10.1111/j.1532-5415.2011.03537.x	Quantitative focus	

For peer review only - http://bmjopen.bmj.com/site/about/guidelines.xhtml

Powers BJ, Coeytaux RR, Dolor RJ, Hasselblad V, Patel UD, Yancy WS, Jr., *et al.* Updated report on comparative effectiveness of ACE inhibitors, ARBs, and direct renin inhibitors for patients with essential hypertension: much more data, little new information. *J Gen Intern Med* 2012;**27**:716-29. https://doi.org/10.1007/s11606-011-1938-8	Quantitative focus	
Rabien A, Price M. Development of a clinical decision support tool for the primary prevention of cardiovascular disease. *Canadian Family Physician* 2015;**61**:S2.	Abstract	
Rahmawati R, Bajorek BV. Self-medication among people living with hypertension: a review. *Fam Pract* 2017;**34**:147-53. https://doi.org/10.1093/fampra/cmw137	Not SR	
Reeve E, Jordan V, Thompson W, Sawan M, Todd A, Gammie TM, *et al.* Withdrawal of antihypertensive drugs in older people. *Cochrane Database Syst Rev* 2020;**6**:CD012572. https://doi.org/10.1002/14651858.CD012572.pub2	Quantitative focus	
Ruppar TM, Dunbar-Jacob JM, Mehr DR, Lewis L, Conn VS. Medication adherence interventions among hypertensive black adults: a systematic review and meta-analysis. *J Hypertens* 2017;**35**:1145-54. https://doi.org/10.1097/HJH.0000000000001260	Full text not retrieved	
Schedlbauer A, Davies P, Fahey T. Interventions to improve adherence to lipid lowering medication. *Cochrane Database Syst Rev* 2010; 10.1002/14651858.CD004371.pub3:CD004371. https://doi.org/10.1002/14651858.CD004371.pub3	Not latest version	
Scranton RE, Goldstein I, Stecher VJ. Erectile dysfunction diagnosis and treatment as a means to improve medication adherence and optimize comorbidity management. *J Sex Med* 2013;**10**:551-61. https://doi.org/10.1111/j.1743-6109.2012.02998.x	Not SR	
Seedat Y. Fixed drug combination in the treatment of hypertension and hyperlipidemia in the developing world. *Circulation* 2010;**122**:e94. http://dx.doi.org/10.1161/CIRCULATIONAHA.110.192773	Abstract	
Selak V, Webster R, Stepien S, Bullen C, Patel A, Thom S, *et al.* Reaching cardiovascular prevention guideline targets with a polypill-based approach: a meta-analysis of randomised clinical trials. *Heart* 2019;**105**:42-8. https://doi.org/10.1136/heartjnl-2018-313108	Not SR	
Shahaj O, Denneny D, Schwappach A, Pearce G, Epiphaniou E, Parke HL, *et al.* Supporting self-management for people with hypertension: a meta-review of quantitative and qualitative systematic reviews. *J Hypertens* 2019;**37**:264-79. https://doi.org/10.1097/HJH.0000000000001867	Full text not retrieved	

For peer review only - http://bmjopen.bmj.com/site/about/guidelines.xhtml

Shaman AM, Smyth B, Arnott C, Palmer SC, Mihailidou AS, Jardine MJ*, et al.* Comparative Efficacy and Safety of BP-Lowering Pharmacotherapy in Patients Undergoing Maintenance Dialysis: A Network Meta-Analysis of Randomized, Controlled Trials. *Clin J Am Soc Nephrol* 2020;**15**:1129-38. https://doi.org/10.2215/CJN.12201019	Full text not retrieved	
Sherrill B, Halpern M, Khan S, Zhang J, Panjabi S. Single-pill vs free-equivalent combination therapies for hypertension: a meta-analysis of health care costs and adherence. *J Clin Hypertens (Greenwich)* 2011;**13**:898-909. https://doi.org/10.1111/j.1751-7176.2011.00550.x	Not SR	
Sommerauer C, Kaushik N, Woodham A, Renom-Guiteras A, Martinez YV, Reeves D*, et al.* Thiazides in the management of hypertension in older adults - a systematic review. *BMC Geriatr* 2017;**17**:228. https://doi.org/10.1186/s12877-017-0576-3	Quantitative focus	
Sparrow RT, Khan AM, Ferreira-Legere LE, Ko DT, Jackevicius CA, Goodman SG*, et al.* Effectiveness of Interventions Aimed at Increasing Statin-Prescribing Rates in Primary Cardiovascular Disease Prevention: A Systematic Review of Randomized Clinical Trials. *JAMA Cardiol* 2019;**4**:1160-9. https://doi.org/10.1001/jamacardio.2019.3066	Full text not retrieved	
Stergiou GS, Bliziotis IA. Home blood pressure monitoring in the diagnosis and treatment of hypertension: a systematic review. *Am J Hypertens* 2011;**24**:123-34. https://doi.org/10.1038/ajh.2010.194	Not SR	
Sua YS, Jiang Y, Thompson DR, Wang W. Effectiveness of mobile phone-based self-management interventions for medication adherence and change in blood pressure in patients with coronary heart disease: A systematic review and meta-analysis. European journal of cardiovascular nursing 2020;19:192-200. https://doi.org/https://dx.doi.org/10.1177/1474515119895678	Medication NR	
Thio SL, Nam J, van Driel ML, Dirven T, Blom JW. Effects of discontinuation of chronic medication in primary care: a systematic review of deprescribing trials. *Br J Gen Pract* 2018;**68**:e663-e72. https://doi.org/10.3399/bjgp18X699041	Quantitative focus	
Thomopoulos C, Parati G, Zanchetti A. Effects of blood pressure lowering treatment in hypertension: 8. Outcome reductions vs. discontinuations because of adverse drug events - meta-analyses of randomized trials. *J Hypertens* 2016;**34**:1451-63. https://doi.org/10.1097/HJH.0000000000000972	Full text not retrieved	
Thomopoulos C, Parati G, Zanchetti A. Effects of blood-pressure-lowering treatment in hypertension: 9. Discontinuations for adverse events attributed to different classes of antihypertensive drugs: meta-analyses of randomized trials. *J Hypertens* 2016;**34**:1921-32. https://doi.org/10.1097/HJH.0000000000001052	Full text not retrieved	

For peer review only - http://bmjopen.bmj.com/site/about/guidelines.xhtml

Vassy JL, Chun S, Advani S, Ludin SA, Smith JG, Alligood EC. Impact of SLCO1B1 Pharmacogenetic Testing on Patient and Healthcare Outcomes: A Systematic Review. *Clin Pharmacol Ther* 2019;**106**:360-73. https://doi.org/10.1002/cpt.1223	Not SR	
Vogele A, Johansson T, Renom-Guiteras A, Reeves D, Rieckert A, Schlender L*, et al.* Effectiveness and safety of beta blockers in the management of hypertension in older adults: a systematic review to help reduce inappropriate prescribing. *BMC Geriatr* 2017;**17**:224. https://doi.org/10.1186/s12877-017-0575-4	Quantitative focus	
Webster R, Patel A, Selak V, Billot L, Bots ML, Brown A*, et al.* Effectiveness of fixed dose combination medication ('polypills') compared with usual care in patients with cardiovascular disease or at high risk: A prospective, individual patient data meta-analysis of 3140 patients in six countries. *Int J Cardiol* 2016;**205**:147-56. https://doi.org/10.1016/j.ijcard.2015.12.015	Not SR	
Weeda ER, Coleman CI, McHorney CA, Crivera C, Schein JR, Sobieraj DM. Impact of once- or twice-daily dosing frequency on adherence to chronic cardiovascular disease medications: A meta-regression analysis. *Int J Cardiol* 2016;**216**:104-9. https://doi.org/10.1016/j.ijcard.2016.04.082	Not SR	
Weernink MG, Vaanholt MC, Groothuis-Oudshoorn CG, Von Birgelen C, M.J IJ, Van Til JA. Patients' priorities for oral anticoagulation therapy in non-valvular atrial fibrillation. *Value in Health* 2017;**20**:A621. http://dx.doi.org/10.1016/j.jval.2017.08.1355	Abstract	
Weisser B, Predel HG, Gillessen A, Hacke C, Vor dem Esche J, Rippin G*, et al.* Single Pill Regimen Leads to Better Adherence and Clinical Outcome in Daily Practice in Patients Suffering from Hypertension and/or Dyslipidemia: Results of a Meta-Analysis. *High Blood Press Cardiovasc Prev* 2020;**27**:157-64. https://doi.org/10.1007/s40292-020-00370-5	Not SR (Duplicate excluded in update search)	
Wettermark B, Qvarnstrom M, Hasselstrom J, Kahan T. Controversies on patient persistence in antihypertensive drug treatment: A systematic review. *JRSM Cardiovascular Disease* 2013; http://dx.doi.org/10.1177/2048004013506452:6-7. http://dx.doi.org/10.1177/2048004013506452	Abstract	
Yan YL, Qiu B, Hu LJ, Jing XD, Liu YJ, Deng SB*, et al.* Efficacy and safety evaluation of intensive statin therapy in older patients with coronary heart disease: a systematic review and meta-analysis. *Eur J Clin Pharmacol* 2013;**69**:2001-9. https://doi.org/10.1007/s00228-013-1570-0	Quantitative focus	
Zhao P, Xu P, Wan C, Wang Z. Evening versus morning dosing regimen drug therapy for hypertension. *Cochrane Database Syst Rev* 2011; 10.1002/14651858.CD004184.pub2:CD004184. https://doi.org/10.1002/14651858.CD004184.pub2	Quantitative focus	

For peer review only

For peer review only - http://bmjopen.bmj.com/site/about/guidelines.xhtml

Zharkova T, Kyomen H. Management Strategies to Prevent Stroke in Elderly with Major Neurocognitive Disorder and Psychosis Treated with Antipsychotic Medication. *The American Journal of Geriatric Psychiatry* 2019;**27**:S135-S6. https://doi.org/10.1016/j.jagp.2019.01.042	Not SR	

For peer review only

For peer review only - http://bmjopen.bmj.com/site/about/guidelines.xhtml

APPENDIX D: CHARACTERISTICS OF INCLUDED REVIEWS

Key for interpreting tables

Green highlighted text:	High overall quality as appraised by AMSTAR-2
Turquoise highlighted text:	Medium overall quality as appraised by AMSTAR-2
Orange highlighted text:	Low overall quality as appraised by AMSTAR-2
Red highlighted text:	Critically-low quality as appraised by the AMSTAR-2
CEESAT Set 1:	Prioritised for full quality appraisal using AMSTAR-2
CEESAT Set 2:	Scored poorly on 1 out of 4 items on CEESAT
CEESAT Set 3:	Scored poorly on 2-4 items on CEESAT

Reviews were judged to be of 'High' relevance if review focused on evaluating intervention/gathering experiences regarding medication of interest within health/social care services relevant to UK service setting, 'Medium' relevance: if review aim/inclusion criteria somewhat relevant to aim of systematic mapping review, but also includes other medications not of interest, or quantity of relevant information is limited, 'Low' relevance: if quantity of relevant information low and/or intervention being evaluated not relevant to UK health/social care setting

For peer review only

For peer review only - http://bmjopen.bmj.com/site/about/guidelines.xhtml

Table 3: Review overview

Focus/Aim	Study (First author, date)	Type of Review (design of studies included), synthesis methods	Eligible age of participants within studies included in the review	Medications of interest	Medical condition for which medication of interest being taken	Relevance of systematic review to aim of evidence-gap map	CEESAT set	Other comments
Evaluating intervention: enhance adherence	Adler, 2017	SR (Quantitative: RCTs), Narrative	NR<br>*Mean age where reported in included studies: 53.6 years to 64 years*	Statins<br>Antihypertensives	Secondary prevention CVD	Medium	1	
Evaluating intervention: enhance adherence	Al Alshaikh, 2016	SR(Quantitative: RCTs), MA+Narrative	18 years or over	Statins<br>Antihypertensives	Secondary prevention CVD	Medium	3	
Evaluating intervention: enhance adherence	Baumgartner, 2020	SR (Quantitative: RCTs, retrospective/prospective cohort, non-randomized, economic models, systematic reviews, meta-analysis), Narrative	NR<br>*Where reported, participants in included studies 18 years of age or over. Some studies specifically included adults 65 years or older*	Statins<br>Antihypertensives	HBP<br>Primary prevention CVD<br>Secondary prevention CVD	Medium	3	
Evaluating intervention: enhance adherence	Bochkareva, 2019	Scoping review (Quantitative: RCTs, non-RCTs, (cross-sectional, cohort studies), registers), Narrative Matrix/map of studies	18 years or over	Antihypertensives	HBP	Medium	3	Focus on Russia. Boad focus of scoping review, of which interventions to increase adherence are one part
Evaluating intervention: enhance adherence	Checchi, 2014	SR (Quantitative: RCTs, nonRCT, controlled, prospective/retrospective, observational, cohort), Narrative	NR<br>*Adults*	Antihypertensives<br>Statins	HBP<br>Primary prevention CVD	Medium	3	

For peer review only - http://bmjopen.bmj.com/site/about/guidelines.xhtml

Focus/Aim	Study (First author, date)	Type of Review (design of studies included), synthesis methods	Eligible age of participants within studies included in the review	Medications of interest	Medical condition for which medication of interest being taken	Relevance of systematic review to aim of evidence-gap map	CEESAT set	Other comments
Evaluating intervention: enhance adherence Secondary aim	Cheema, 2014	SR (Quantitative: RCTs, systematic reviews of RCTs), Meta-analysis+Narrative	18 years or over	Antihypertensives	HBP	High	2	
Evaluating intervention: optimise prescribing	Chhina, 2013	SR (Quantitative: RCT, observational studies with a control group), Narrative	NR *1 included study focused on children, 3 focused on elderly*	Antihypertensives	HBP	Medium	3	One included study focused on children
Evaluating intervention: enhance adherence	Conn, 2015[70]	SR (Quantitative: NR), Meta-analysis	NR *Median value for mean age accross included studies was 54.4 years*	Statins Antihypertensives	HBP Secondary prevention CVD	Medium	2	Medications of interest inferred from population disease characteristics
Evaluating intervention: enhance adherence	Conn, 2015[71]	SR (Quantitative: treatment-versus-control comparisons, pre-experimental studies), Exploratory analyses, Meta-analysis	NR *Median mean participant age accross included studies was 60.70 years*	Statins Antihypertensives	HBP Primary prevention CVD Secondary prevention CVD Inferred from population disease characteristics	Medium	2	Medications of interest inferred from population disease characteristics
Evaluating intervention: enhance adherence	Conn, 2015[36]	SR (Quantitative: varied research methods were included), Meta-analysis	Other *Adults*	Antihypertensives	HBP	High	3	

For peer review only - http://bmjopen.bmj.com/site/about/guidelines.xhtml

Focus/Aim	Study (First author, date)	Type of Review (design of studies included), synthesis methods	Eligible age of participants within studies included in the review	Medications of interest	Medical condition for which medication of interest being taken	Relevance of systematic review to aim of evidence-gap map	CEESAT set	Other comments
Evaluating intervention: enhance adherence	Conn, 2016	SR (Quantitative: RCTs, non-RCTs, Pre-experimental before and after), Narrative+Meta-analysis	NR<br>*Adults. Median mean age accross included studies 53 years*	Statins<br>Antihypertensives	Primary prevention CVD<br>Depression<br>HBP<br>Secondary prevention CVD	Medium	2	Medications of interest inferred from population disease characteristic
Evaluating intervention: enhance adherence secondary aim	de Cates, 2014	SR (Quantitative: RCTs), Meta-analysis	18 years or over	Statins<br>Antihypertensives	Primary prevention CVD<br>Secondary prevention CVD	Medium	1	Adherence only secondary outcome
Evaluating intervention: enhance adherence	De Simoni, 2013	Quantitative (RCTs), Narrative+Meta-analysis	Other<br>*Adults*	Antihypertensives	HBP<br>Secondary prevention CVD	High	2	
Evaluating intervention: enhance adherence	Deichmann. 2016	SR (Quantitative: RCTs, parallel group or crossover, individual or cluster randomization), Meta-analysis+Narrative	18 years or over	Statins	Primary prevention CVD<br>Secondary prevention CVD	Medium	3	

For peer review only - http://bmjopen.bmj.com/site/about/guidelines.xhtml

Focus/Aim	Study (First author, date)	Type of Review (design of studies included), synthesis methods	Eligible age of participants within studies included in the review	Medications of interest	Medical condition for which medication of interest being taken	Relevance of systematic review to aim of evidence-gap map	CEESAT set	Other comments
Evaluating intervention: enhance adherence	Demonceau, 2013	SR (Quantitative: RCT, cross-over and cluster-randomized), Meta-analysis+meta-regression	Other *Average age expressed in years was 47.5 [range (3.0; 73.7)] in the usual care group and 46.8 [range (3.4; 76.2)] in the intervention group* *Adults and children*	Antihypertensives *Assumed*	HBP NR	Medium	3	Included methadone clinic patients (n=1). Included children
Patient views: adherence to medication of interest Only these of interest	Dhar, 2017	SR (Mixed: only quali studies met focus of review. Quantitative, descriptive-co relational study, cross-sectional, focus group interviews, survey, interviews), Narrative	18 years or over	Antihypertensives	HBP	Low	3	Focus on undeveloped countries. Only 5 included qualitative studies
Evaluating intervention: deprescribing	Dills, 2018	SR (Quantitative: Dills-2019-Deprescribing Medications for Chronic.pdf: Page 1: "Randomized Controlled Trials" Narrative	18 years or over	Statins Antihypertensives	HBP Secondary prevention CVD Other Illnesses associated with use of: alendronate, PPI, H2 antagonist	Medium	1	Palliative care studies where life expectancy is > 6 months included

For peer review only - http://bmjopen.bmj.com/site/about/guidelines.xhtml

Focus/Aim	Study (First author, date)	Type of Review (design of studies included), synthesis methods	Eligible age of participants within studies included in the review	Medications of interest	Medical condition for which medication of interest being taken	Relevance of systematic review to aim of evidence-gap map	CEESAT set	Other comments
Evaluating intervention: enhance adherence	Du, 2018	SR (Quantitative: parallel comparison studies, retrospective cohort, prospective), Meta-analysis	NR *Mean age of included studies (where reported) ranged from 53-76 years*	Antihypertensives	HBP	High	3	
Evaluating intervention: enhance adherence	Fletcher, 2015	SR (Quantitative: Randomized and quasi-randomized trials), Narrative+Meta-analysis	NR *Where reported, participants mean age ranged from 38 years to 77 accross included studies*	Antihypertensives	HBP	High	1	
Evaluating intervention: enhance adherence	Gupta, 2010	SR (Quantitative: RCT, clinical trial and prospective or retrospective cohort), Narrative+Meta-analysis	Other *18 to 79 years*	Antihypertensives	HBP	High	3	
Evaluating intervention: enhance adherence	Guzman-Tordecilla, 2020	SR (Quantitative: Guzman-Tordecilla-2020-InterventionsToIncreaseThePhar.pdf: Page 5: "Quasi-experimental" "Experimental" Narrative	17 years or over	Antihypertensives	HBP	Low	3	Studies conductedi n Latin America

For peer review only - http://bmjopen.bmj.com/site/about/guidelines.xhtml

Focus/Aim	Study (First author, date)	Type of Review (design of studies included), synthesis methods	Eligible age of participants within studies included in the review	Medications of interest	Medical condition for which medication of interest being taken	Relevance of systematic review to aim of evidence-gap map	CEESAT set	Other comments
Evaluating intervention: deprescribing Secondary aim, not actually explicit. Look at interventions to reduce Fall Risk-Inducing Drugs (FRID) in an attempt to understand whether this reduced falls.	Hart, 2020	SR (Quantitative: observational or intervention (eg, RCTs and quasi-experimental design)), Narrative	Other *60 or over*	Antihypertensives	NR	Medium	3	Lots of other drugs. Also approach reduction of drugs of interest in a round-about way, rather than directly wanting to reduce them
Evaluating intervention: optimise prescribing	Hukins, 2019	SR (Quantitative: observational, non-randomised evaluation), Narrative	Other *People with a diagnosis of/suspected dementia*	Statins	NR	Medium	1	
Patient views: adherence to medication of interest Patient views: intervention to enhance adherence	Ingersgaard, 2020	Review of SR (Other, all types of systematic reviews), Narrative	NR	Statins Antihypertensives	Primary prevention CVD Secondary prevention CVD	Medium	2	Review of reviews, little relevant qualitative evidence
Evaluating intervention: optimise prescribing	Jeffery, 2015	SR (Quantitative: RCTs, cluster and nexted), Meta-analysis+Narrative	NA *Targeted prescribers*	Statins Antihypertensives	Primary prevention CVD Secondary prevention CVD	High	1	
Evaluating intervention: optimise prescribing	Kang, 2016	SR (Quantitative: RCTs, non-RCT, prospective observational studies), Narrative+Meta-analysis	18 years or over	Statins Antihypertensives	Secondary prevention CVD	High	3	

For peer review only - http://bmjopen.bmj.com/site/about/guidelines.xhtml

Focus/Aim	Study (First author, date)	Type of Review (design of studies included), synthesis methods	Eligible age of participants within studies included in the review	Medications of interest	Medical condition for which medication of interest being taken	Relevance of systematic review to aim of evidence-gap map	CEESAT set	Other comments
Evaluating intervention: enhance adherence	Kassavou, 2018	SR (Quantitative: RCTs, randomisation at individual patient or cluster level), Meta-analysis+meta-regression	18 years or over	Statins<br>Antihypertensives	HBP<br>Primary prevention CVD<br>Secondary prevention CVD<br>Other Cardio-metabolic conditions, high cholesterol	High	2	
Evaluating intervention: enhance adherence	Kawalec, 2018	SR (Quantitative: No restrictions were applied as to the study design. SRs, cohort, non-RCT, prospective observational study), Narrative+Meta-analysis	NR<br>*Where reported, all included studies included participants of 18 years or older*	Antihypertensives	HBP	High	3	
Practitioner views: prescribing medication of interest<br>Patient views: adherence to medication of interest	Khatib, 2014	SR (Mixed: Qualitative and quantitative observational studies. Included RCT, cohort, case-control), Framework/Content analysis, Narrative	Other<br>*Any age*	Antihypertensives	HBP	High	2	Methods of qualitative synthesis unclear

For peer review only - http://bmjopen.bmj.com/site/about/guidelines.xhtml

Focus/Aim	Study (First author, date)	Type of Review (design of studies included), synthesis methods	Eligible age of participants within studies included in the review	Medications of interest	Medical condition for which medication of interest being taken	Relevance of systematic review to aim of evidence-gap map	CEESAT set	Other comments
Evaluating intervention: optimise prescribing<br>Think this is most appropriate overall, but could justify selecting more than one?<br>Evaluating intervention: enhance adherence	King, 2018	SR (Quantitative: RCT, observational), Narrative	NR	Statins<br>Antihypertensives	HBP<br>Primary prevention CVD	Medium	2	
Patient views: adherence to medication of interest<br>Family/carer views: adherence to medication of interest	Kinnear, 2019	SR (Qualitative: face to face/telephone/online interviews, focus groups) Thematic	NR<br>*264 individuals with FH and 13 family members were involved, aged 8-69 years*	Statins	Primary prevention CVD<br>Secondary prevention CVD	Medium	1	N=7 papers reported findings from samples including individuals under 18 years. N=4 papers reported parental views of having children with FH
Evaluating intervention: enhance adherence	Krack, 2019	SR (Quantitative: RCTs, non-RCTs), Narrative+Meta-analysis	NR	Statins<br>Antihypertensives	HBP<br>Primary prevention CVD<br>Secondary prevention CVD	Low	2	
Evaluating intervention: enhance adherence	Kronish, 2011	SR (Quantitative: observational cohorts), Meta-analysis	18 years or over	Antihypertensives	HBP	High	2	

For peer review only - http://bmjopen.bmj.com/site/about/guidelines.xhtml

Focus/Aim	Study (First author, date)	Type of Review (design of studies included), synthesis methods	Eligible age of participants within studies included in the review	Medications of interest	Medical condition for which medication of interest being taken	Relevance of systematic review to aim of evidence-gap map	CEESAT set	Other comments
Evaluating intervention: enhance adherence<br>Evaluating intervention: optimise prescribing<br>Because of interventions directed at HCPs	Laba, 2013	SR (Quantitative: Randomised/quasi-randomised controlled trials), Narrative	Other<br>*Adults*	Statins<br>Antihypertensives	HBP<br>Primary prevention CVD<br>*Possibly secondary prevention, unclear*	Medium	3	
Evaluating intervention: enhance adherence	Lawrence, 2015	SR (Quantitative: RCTs), Meta-analysis	18 years or over	Statins<br>Antihypertensives	Secondary prevention CVD<br>*Prevention of secondary stroke and/or TIA*	Medium	2	Only 3 studies reporting adherence outcomes for each of statins and antihypertensives
Evaluating intervention: optimise prescribing<br>Secondary aim	Legare, 2010	SR (Quantitative: RCTs, well designed quasi-experimental studies (controlled clinical trials, CBA, ITS), Narrative	NR	Statins	NR	Low	1	Possibly only one study (of 5) about statins, the others are not relevant
Evaluating intervention: enhance adherence	Leslie, 2016	SR (Quantitative: RCTs), Narrative	65 years or over<br>*or consisted of Medicare beneficiaries*	Antihypertensives<br>Statins	HBP<br>Primary prevention CVD	Medium	3	Focus on elderly. Medications of interest assumed from population characteristics
Evaluating intervention: optimise prescribing	Loganathan, 2011	SR (Quantitative: RCTs, non-RCTs), Narrative	65 years or over	Antihypertensives	NR	Medium	3	Only a couple of included studies with medications of interest

For peer review only - http://bmjopen.bmj.com/site/about/guidelines.xhtml

Focus/Aim	Study (First author, date)	Type of Review (design of studies included), synthesis methods	Eligible age of participants within studies included in the review	Medications of interest	Medical condition for which medication of interest being taken	Relevance of systematic review to aim of evidence-gap map	CEESAT set	Other comments
Evaluating intervention: enhance adherence Secondary aim	Maimaris, 2013	SR (Quantitative: RCTs, controlled trials, cohort, cross-sectional, case-control, cross-sectional, ecological), Narrative	NR	Antihypertensives	HBP	Medium	1	Limited information relating to adherence of hypertensives. Uncertain as to relevance of findings to UK health and social care setting
Evaluating intervention: enhance adherence Seconardy aim	Mallat, 2016	SR (Quantitative: RCTs), Meta-analysis	18 years or over	Antihypertensives	HBP	High	1	
Evaluating intervention: enhance adherence	Mamudu, 2014	SR (Quantitative: RCTs, non-RCTs), Narrative	NR	Statins Antihypertensives	Primary prevention CVD	Medium	3	
Evaluating intervention: enhance adherence	Mann, 2014	SR (Quantitative: RCTs, ITS, CBA), Narrative	Other *Adult patients*	Statins Antihypertensives	HBP Primary prevention CVD Secondary prevention CVD	Low	2	One included study included participants aged 14 and above. Intervention not relevant to UK setting
Patient views: adherence to medication of interest Qualitative: other Understanding and experiences of hypertension and drug adherence	Marshall, 2012	SR (Qualitative: Interviews, focus groups), Narrative/Thematic	NR *Where reported, age of population within included studies ranged from 18-92 years*	Antihypertensives	HBP	High	3	

For peer review only - http://bmjopen.bmj.com/site/about/guidelines.xhtml

Focus/Aim	Study (First author, date)	Type of Review (design of studies included), synthesis methods	Eligible age of participants within studies included in the review	Medications of interest	Medical condition for which medication of interest being taken	Relevance of systematic review to aim of evidence-gap map	CEESAT set	Other comments
Evaluating intervention: enhance adherence	Milosavljevic, 2018	SR (Quantitative: RCTs, quasi-controlled trials, cluster-controlled trials, BA, retrospective/prospective cohort, observational, single group pre/post comparisons, statistical predictions), Narrative	NR	Antihypertensives	HBP<br>Depression	Medium	2	
Evaluating intervention: enhance adherence	Morrissey, 2017	SR (Quantitative: RCTs), Narrative Meta-analysis	NR<br>*Where reported, mean age ranged from 51 to 76 across included studies*	Antihypertensives	HBP	High	1	
Evaluating intervention: optimise prescribing	Nguyen, 2018	SR (Quantitative: RCTs), Meta-analysis	NR	Statins	Secondary prevention CVD<br>*Ischaemic heart disease*	Medium	2	
Evaluating intervention: enhance adherence	Nieuwlaat, 2014	SR (Quantitative: RCTs), Narrative	NR<br>*Included studies characteristics table indicates both adult and child populations included*	Statins<br>Antihypertensives	HBP<br>Primary prevention CVD<br>Secondary prevention CVD	Medium	1	

For peer review only - http://bmjopen.bmj.com/site/about/guidelines.xhtml

Focus/Aim	Study (First author, date)	Type of Review (design of studies included), synthesis methods	Eligible age of participants within studies included in the review	Medications of interest	Medical condition for which medication of interest being taken	Relevance of systematic review to aim of evidence-gap map	CEESAT set	Other comments
Qualitative: other Factors which predict medication adherence. Not a qualitative review but didn't know what else to select What factors are associated with/influence or predict ad- herence of anti-hypertensive medication in older adults with HBP?, How are their causal relationship and their influence on MA in older adults with HBP based on ecological ap-proach?	Oori, 2019	Integrative review (Mixed: quantitative and qualitative designs, including: cross-sectional and descriptive, interventional, cohort and two descriptive qualitative), Content analysis	Older adults	Antihypertensives	HBP	Low	3	A qualitative synthesis of quant/qual data. Included because of qualitative aspect, but only 'descriptive qualitative' studies were included
Evaluating intervention: deprescribing	Page, 2016	SR (Quantitative: RCTs, quasi-randomized controlled studies, non-RCTs, prospective and retrospective cohort, case-control, single arm, BA), Meta-analysis	65 years or over	Statins Antihypertensives	HBP Primary prevention CVD Secondary prevention CVD	Medium	2	
Evaluating intervention: enhance adherence	Palmer, 2018	SR (Quantitative: RCTs of parallel group design that randomised by participant or by cluster), Narrative	18 years or over	Statins Antihypertensives	Primary prevention CVD	Medium	1	

For peer review only - http://bmjopen.bmj.com/site/about/guidelines.xhtml

Focus/Aim	Study (First author, date)	Type of Review (design of studies included), synthesis methods	Eligible age of participants within studies included in the review	Medications of interest	Medical condition for which medication of interest being taken	Relevance of systematic review to aim of evidence-gap map	CEESAT set	Other comments
Evaluating intervention: optimise prescribing<br>One of a variety of aims	Pantoja, 2019	SR (Quantitative: RCTs, non-RCTs), Narrative	NR	Statins<br>Antihypertensives	HBP<br>Primary prevention<br>CVD *Unclear if statin for primary or secondary prevention*	Medium	1	Some included studies included child participants. Low amount of information about prescribing drugs of interest. intervention is more of a general aid
Evaluating intervention: enhance adherence	Park, 2017	SR (Quantitative: Original research, comparison group, pre/post comparisons, statistical predictions), Narrative	NR	Antihypertensives<br>Statins	HBP<br>Primary prevention<br>CVD<br>NR	Low	3	Not relevant to UK, medications not of interest included
Evaluating intervention: enhance adherence Note that focus is on 'use' which includes initiating prescribing. Drugs of interest may be currently under- or -ver-used	Polinski, 2011	SR (Quantitative: Polinski-2011-Medicare Part D's Effect on the.pdf: Page 3: "Articles were included if they reported original results regarding Part D drug utilization for specific drugs or drug classes, whether drawn from self-report surveys or prescription drug claims"<br>Narrative	NR	Statins<br>Antihypertensives	HBP<br>Primary prevention<br>CVD<br>NR<br>*Not clearly reported*	Low	3	Low relevance to UK. Other medications not of interest included

For peer review only - http://bmjopen.bmj.com/site/about/guidelines.xhtml

Focus/Aim	Study (First author, date)	Type of Review (design of studies included), synthesis methods	Eligible age of participants within studies included in the review	Medications of interest	Medical condition for which medication of interest being taken	Relevance of systematic review to aim of evidence-gap map	CEESAT set	Other comments
Evaluating intervention: enhance adherence Seconary aim: interventions to affect behaviour change that leads to prevention of chronic disease - e.g. taking antihypertensives...	Posadzki, 2016	SR (Quantitative: RCTs, cluster RCTs, quasi-RCTs, ITS, CBA), Narrative+Meta-analysis	NR	Statins Antihypertensives	HBP Primary prevention CVD	Medium	1	Included children. Interventions target general positive behaviour change in terms of health management. However there are specific studies reporting adherence-type interventions. Several non-relevant drugs and outcomes within the review.
Evaluating intervention: enhance adherence	Rash, 2016	SR (Quantitative: RCTs), Narrative	NR	Statins	Primary prevention CVD Secondary prevention CVD	High	2	
Patient views: adherence to medication of interest	Rashid, 2014	SR (Qualitative: Focus groups, interviews, interviews and questionnaires), Meta-ethnography	Other *Adults*	Statins	Secondary prevention CVD	Medium	3	
Qualitative: other Rashid 2018.pdf: Page 1: "explored medication-taking experiences in patients, citizens, carers, relatives and clinicians."	Rashid, 2018	SR (Qualitative: All types of qualitative studies, including those linked to observational, experimental, mixed methods papers. Interviews, focus groups or a combination), Thematic	Other	Antihypertensives	HBP	Medium	3	Includes children and adults. Other medications not of interest included

For peer review only - http://bmjopen.bmj.com/site/about/guidelines.xhtml

Focus/Aim	Study (First author, date)	Type of Review (design of studies included), synthesis methods	Eligible age of participants within studies included in the review	Medications of interest	Medical condition for which medication of interest being taken	Relevance of systematic review to aim of evidence-gap map	CEESAT set	Other comments
Patient views: intervention to encourage deprescribing Family/carer views: intervention to encourage deprescribing	Reeve, 2013	SR (Mixed: No limits placed on the type of methods used, qualitative, quantitative or mixed), Narrative/Content analysis/Thematic	Other *No limits were placed on the age of the participants*	Antihypertensives	HBP Anxiety NR	Medium	3	Methods of synthesis unclear
Evaluating intervention: enhance adherence	Reeves, 2020	SR (Quantitative: RCTs), Narrative	18 years or over *Adult*	Antihypertensives	HBP	High	2	
Evaluating intervention: enhance adherence One of multiple aims	Sanders, 2011	SR (Quantitative: comparative clinical studies of any design, including RCTs, non-RCTs, retrospective/prospective cohort, and case-control), Narrative+Meta-analysis	18 years or over	Antihypertensives	HBP *Essential hypertension*	Medium	1	Adherence low on the list of outcomes
Evaluating intervention: optimise prescribing	Shanbhag, 2018	SR (Quantitative: RCTs, cohort (with comparisons), controlled and uncontrolled BA, ITS), Narrative	NR	Antihypertensives	HBP *Associated with heart failure*	High	1	
Evaluating intervention: enhance adherence	Stacey, 2017	SR (Quantitative: RCTs), Meta-analysis	18 years or over	Antihypertensives	HBP Primary prevention CVD Seconday prevention CVD	Medium	1	Study focus: RE: making decisions about screening or treatment options for themselves, a child, or an incapacitated significant other

For peer review only - http://bmjopen.bmj.com/site/about/guidelines.xhtml

Focus/Aim	Study (First author, date)	Type of Review (design of studies included), synthesis methods	Eligible age of participants within studies included in the review	Medications of interest	Medical condition for which medication of interest being taken	Relevance of systematic review to aim of evidence-gap map	CEESAT set	Other comments
Evaluating intervention: enhance adherence	Tsioufis, 2020	Rapid review (Quantitative: RCTs, observational studies), Narrative	Other<br>*Adults. Where reported, mean age of populations in included studies ranged from 47 to 71.5 years*	Antihypertensives	HBP	High	3	
Evaluating intervention: enhance adherence	Uhlig, 2013	SR (Quantitative: prospective comparative), Narrative+Meta-analysis	Other<br>*Adults*	Antihypertensives	HBP	High	3	
Evaluating intervention: enhance adherence	van Driel, 2016	SR (Quantitative: RCTs of parallel-group or cross-over design, that used individual or cluster randomisation), Narrative+Meta-analysis	18 years or over	Statins	Primary prevention CVD<br>Seconday prevention CVD	High	1	
Evaluating intervention: enhance adherence	Xiong, 2018	SR (Quantitative: RCTs, non-RCTs, before-and-after studies without a control group), Narrative	NR<br>*All studies involved adults aged from 40 to 69*	Antihypertensives	HBP	High	3	
Evaluating intervention: enhance adherence	Xu, 2018	SR (Quantitative: RCTs), Meta-analysis	18 years or over	Antihypertensives	HBP	Medium	3	Focus on Chinese patients

For peer review only - http://bmjopen.bmj.com/site/about/guidelines.xhtml

Focus/Aim	Study (First author, date)	Type of Review (design of studies included), synthesis methods	Eligible age of participants within studies included in the review	Medications of interest	Medical condition for which medication of interest being taken	Relevance of systematic review to aim of evidence-gap map	CEESAT set	Other comments
Evaluating intervention: optimise prescribing<br>Secondary aim Evaluating intervention: enhance adherence<br>Secondary aim	Yuan, 2019	SR (Quantitative: RCTs, high quality cohort studies), Narrative+Meta-analysis	NR	Antihypertensives	HBP<br>Primary prevention CVD<br>Seconday prevention CVD<br>*Assumed from content of paper*	Medium	3	

Green highlighted text=High overall quality as appraised by AMSTAR-2, Turquoise highlighted text=Medium overall quality as appraised by AMSTAR-2, Orange highlighted text=Low overall quality as appraised by AMSTAR-2, Red highlighted text=Critically-low quality as appraised by the AMSTAR-2. *CEESAT Set: 1=prioritised for full quality appraisal using AMSTAR-2, CEESAT Set 2=Scored poorly on 1 out of 4 items on CEESAT, CEESAT Set 3=Scored poorly on 2-4 items on CEESAT. BA=Before and After, BPD=Borderline Personality Disorder, CBA=Controlled Before and After, CPG=Clinical Practice Guidelines, CT=Controlled-Trials, CVD=Cardiovascular Disease, FH=Familial Hypercholesterolemia, HPP=High Blood Pressure, ITS=Interrupted Time Series, MA=Meta-analysis, NR=Not Reported, PPI=Proton Pump Inhibitors, PTSD=Post-Traumatic Stress Disorder, RCT=Randomized Controlled Trial, RQ=Research Question, SR=Systematic Review

For peer review only

Table 4: Systematic reviews appraised using AMSTAR-2 which synthesised studies evaluating the effectiveness of an intervention

Focus/ Aim	Study (First author, date)	Medications of interest	Other medications included	Intervention name/s and aim	Outcomes measured	Setting/Context	Relevant part of care pathway
Evaluating intervention: deprescribing	Dills, 2018	Statins<br>Antihypertensives	Diuretics, salmeterol, insulin, sulfonylurea, antipsychotics, anxiolytic, CV drugs, levodopa, nitrate, benzodiazepines<br>Z-drugs<br>Antidepressants	Deprescription: reduce medication burden, and enhance control of chronic medical and mental health conditions commonly managed by primary care physicians, compared with standard care in the non-terminally ill adult population	Primary outcome: successful deprescription, Secondary outcome: AE related to drug or underlying chronic condition as a result of deprescription	Primary/health /social care<br>Hospital (inpatient)<br>Hospital (outpatient)<br>Care home<br>Patient home	Decision to stop treatment: supported
Evaluating intervention: enhance adherence	Stacey, 2017	Antihypertensives	Hepatitis B vaccination, schizophrenia medication, immunotherapy for multiple sclerosis, vaccines (measles, mumps, rubella), radioactive iodine treatment for thyroid cancer, antibiotic use for upper respiratory infections, chemotherapy for cancer, medications for diabetes, hormone therapy, "breast cancer prevention medication, bisphosphonates, thyroid cancer radioactive iodine treatment	Decision aids for people facing health treatment or screening decisions.<br>Aim: help people make specific and deliberated choices among options, by making the decision explicit/by providing information on the options and outcomes relevant to person's health status as well as implicit methods to clarify values	Attributes of choice made: does patient decision aid improve match between chosen option and features that matter most to informed patient (demonstrated by knowledge, accurate risk perceptions, values-choice congruence).<br><br>Attributes of decision-making process: does patient decision aid help patients to recognize decision needs to be made, feel informed about options and their features, be clear about option features that matter most, discuss values with their clinician, and become involved in decision making? Other decision-making process variables: Decisional conflict, Patient-clinician communication, participation in decision making, proportion undecided, satisfaction with the choice/process of decision making/preparation for decision	NR	Information and/or advice<br>Shared decision making<br>Patient adherence

Focus/ Aim	Study (First author, date)	Medications of interest	Other medications included	Intervention name/s and aim	Outcomes measured	Setting/Context	Relevant part of care pathway
					making. Secondary outcomes: Choice (the actual choice implemented; if not reported, the participants preferred option), adherence to chosen option, health status and quality of life (generic and condition-specific), anxiety, depression, emotional distress, regret, confidence, costs, cost-effectiveness, consultation length, litigation rates		

For peer review only - http://bmjopen.bmj.com/site/about/guidelines.xhtml

Focus/ Aim	Study (First author, date)	Medications of interest	Other medications included	Intervention name/s and aim	Outcomes measured	Setting/Context	Relevant part of care pathway
Evaluating intervention: enhance adherence*	Posadzki 2016	Statins Antihypertensives	Asthma medication, prescription drugs taken without prescription or more than prescribed, illicit non-injection drugs, glaucoma medications, inhaled corticosteroids, insulin, methadone maintenance, cholinesterase inhibitor medications, oral hypoglycaemic medications, nicotine replacement therapy, bupropion, and varenicline, antiretroviral treatment, bone active medication, non-hormonal oral agents, opiates antidepressants	Automated telephone communication systems for preventing disease and managing long-term conditions	Health behaviour and clinical outcomes, changes in health-enhancing behaviour, risk-taking behaviour, physiological measures, blood biochemistry. Process outcomes: change in acceptability of service (e.g. consumer accessibility/usability of interventions to apply information/support supplied), satisfaction (e.g. patient/carer) the intervention), cost-effectiveness. Cognitive outcomes: changes in knowledge (i.e. accurate risk knowledge and perception), attitude and intention to change, self-efficacy. Patient-centred outcomes: quality of life, adverse outcomes, unintended AE attributable to intervention	Primary/health/ social care Secondary health/social care (not hospital) Hospital (inpatient) Hospital (outpatient) Patient home	Identifying health or prescribing concern Accessing care Choosing a medication Decision to start agreed treatment plan Patient adherence Maintaining treatment
Evaluating intervention: enhance adherence	Adler, 2017	Statins Antihypertensives	Antiplatelet	Mobile phone text messaging to enhance adherence to recommended medication in patients with established arterial occlusive events	Adherence to treatment, fatal/non-fatal cardiovascular events, combined CVD event, Surrogate outcomes according to the different interventions recommended for secondary prevention including: low-density lipoprotein-cholesterol, blood pressure, heart rate, urinary 11-dehydrothromboxane B2, AEs	Primary/health /social care Hospital (outpatient)	Patient adherence

For peer review only - http://bmjopen.bmj.com/site/about/guidelines.xhtml

Focus/ Aim	Study (First author, date)	Medications of interest	Other medications included	Intervention name/s and aim	Outcomes measured	Setting/Context	Relevant part of care pathway
Evaluating intervention: enhance adherence	Fletcher, 2015	Antihypertensives	NA	Self-monitoring of blood pressure: improve medication adherence, medication persistence, and lifestyle factors in people with hypertension	Primary outcomes: antihypertensive medication adherence and persistence, dietary outcomes, alcohol consumption and physical activity. Secondary outcomes: BP, BP control, and adherence to SMBP component of interventions	Primary/health/ social care<br>Hospital (outpatient)	Decision to start agreed treatment plan<br>Patient adherence<br>Progress review
Evaluating intervention: enhance adherence	Morrissey, 2017	Antihypertensives	NA	Interventions to enhance medication adherence and blood pressure control in hypertension	Change in BP, medication adherence	Primary/health/ social care<br>Hospital (inpatient)<br>Hospital (outpatient)<br>Care home<br>Patient home	Patient adherence
Evaluating intervention: enhance adherence	Nieuwlaat, 2014	Antidepressants<br>Statins<br>Antihypertensives	Medications to treat following conditions: HIV/AIDS, psychiatric disorders, chronic obstructive pulmonary disease, CVD/CV risk, diabetes, antibiotics, arthritis, complex chronic care, dyspepsia, glaucoma, oral anticoagulation, osteoporosis, tuberculosis, acne, cancer, hepatitis, iron supplementation during pregnancy, liver transplant, malaria, oral contraceptives,	Interventions to affect adherence with prescribed, self-administered medications	Medication adherence, clinical outcomes	Primary/health/ social care<br>Hospital (inpatient)<br>Hospital (outpatient)<br>Care home<br>Patient home	Information and/or advice<br>Patient adherence<br>Progress review

For peer review only - http://bmjopen.bmj.com/site/about/guidelines.xhtml

Focus/ Aim	Study (First author, date)	Medications of interest	Other medications included	Intervention name/s and aim	Outcomes measured	Setting/Context	Relevant part of care pathway
			antidepressants tonsillectomy/adenoidectomy, ulcerative colitis				
Evaluating intervention: enhance adherence	Palmer, 2018	Statins Antihypertensives	Antiplatelet drugs (low-dose aspirin, non-aspirin antiplatelet drugs)	Mobile phone-based interventions to improve adherence to medication prescribed for the primary prevention of cardiovascular disease in adults	Objective measures of adherence to treatment, combined CVD events, AE, indirect measures of adherence to treatment, health-related quality of life, cognitive outcomes (satisfaction with treatment, medication-taking self-efficacy, autonomy related to medication, attitudes (e.g. concerns about medicine AEs)), costs, process measures: extent of intervention received and acceptability of intervention	Primary/health/ social care Secondary health/social care (not hospital) Hospital (outpatient) Patient home	Progress review Patient adherence
Evaluating intervention: enhance adherence	van Driel, 2016	Statins	Any lipid-lowering medication	Interventions to improve adherence to lipid-lowering drugs: 1. Simplification of drug regimen; 2. Patient education and information; 3. Intensified patient care; 4. Complex behavioural approaches; 5. Decision support systems; 6. Administrative improvements; and 7. Pharmacy-led interventions	Indirect measures of adherence, subjective measures of adherence, direct measures of adherence, physiological indicators, health outcome indications (e.g. quality of life, morbidity, mortality), AEs, implications for costs (impact of intervention on economic outcomes, economic evaluation)	Primary/health /social care Secondary health/social care (not hospital)	Information and/or advice Patient adherence Progress review

For peer review only - http://bmjopen.bmj.com/site/about/guidelines.xhtml

Focus/ Aim	Study (First author, date)	Medications of interest	Other medications included	Intervention name/s and aim	Outcomes measured	Setting/Context	Relevant part of care pathway
Evaluating intervention: enhance adherence*	Sanders, 2011	Antihypertensives	NA	ACEIs to treat essential hypertension	Blood pressure control, mortality, morbidity, safety, specific adverse events, persistence/adherence, rate of use of a single antihypertensive medication for blood pressure control, lipid levels, rates of progression to type 2 diabetes, carbohydrate metabolism/diabetes control, left ventricular mass/function, kidney disease	Primary/health/ social care<br>Secondary health/social care (not hospital)<br>Hospital (outpatient)<br>Other<br>*Setting of studies not restricted*	Choosing a medication<br>Progress review<br>Patient adherence
Evaluating intervention: enhance adherence*	Mallat, 2016	Antihypertensives	NA	Free versus fixed drug combination. Aim: to increase adherence, increase overall BP-lowering efficacy	Mortality, morbidity (cardiovascular outcomes such as coronary events, stroke, progression of peripheral vascular disease and kidney disease), mean systolic BP, AE, adherence to treatment	NR	Choosing a medication<br>Patient adherence<br>Progress review

For peer review only - http://bmjopen.bmj.com/site/about/guidelines.xhtml

For peer review only

Focus/ Aim	Study (First author, date)	Medications of interest	Other medications included	Intervention name/s and aim	Outcomes measured	Setting/Context	Relevant part of care pathway
Evaluating intervention: enhance adherence*	de Cates, 2014	Statins<br>Antihypertensives	NA	A fixed-dose combination therapy, a combination of several active components into a single pill, including at least one statin and one antihypertensive agent. Aim: to optimise CVD risk and reduce CVD fatal and non-fatal events	Mortality, non-fatal CVD endpoints, AEs (overall rates of discontinuation, proportion of participants experiencing specific symptoms or results and rates of discontinuation by specific symptoms). Secondary outcomes: Systolic and diastolic blood pressure, total and LDL cholesterol, adherence, health-related QOL, costs of fixed-dose combination therapy	Primary/health/ social care	Choosing a medication<br>Progress Review<br>Patient adherence
Evaluating intervention: enhance adherence*	Maimaris, 2013	Antihypertensives	NA	National or regional health system arrangements on HT awareness, treatment, control, and antihypertensive medication adherence	HT awareness (persons with clinically measured HT diagnosed by a health care professional as hypertensive), HT treatment (use of at least one antihypertensive medication in an individual with known HT), antihypertensive medication adherence, HT control	Primary/health/ social care<br>Secondary health/social care (not hospital)<br>Hospital (inpatient)<br>Hospital (outpatient)	Identifying health or prescribing concern<br>Accessing care<br>Decision to start agreed treatment plan<br>Patient adherence<br>Other *health systems, infrastructure. System level interventions*

For peer review only - http://bmjopen.bmj.com/site/about/guidelines.xhtml

Focus/ Aim	Study (First author, date)	Medications of interest	Other medications included	Intervention name/s and aim	Outcomes measured	Setting/Context	Relevant part of care pathway
Evaluating intervention: optimise prescribing	Gillaizeau, 2013	Antidepressants	Insulin, aminoglycoside antibiotics, anticoagulants, theophylline, anti-rejection drugs, anaesthetic agents, gonadotropins	Computerized advice on drug dosage to improve prescribing practice computerized advice on drug dosage: optimize effects and minimize dangers	Proportion of participants/time: plasma drug concentrations within therapeutic range, studied physiological parameter within the target range. "Time to achieve therapeutic control, proportion of participants: with toxic drug levels, with clinical improvement, with adverse effects of drug therapy. Proportion of deaths, LOS, total cost/participant	Primary/health /social care<br>Secondary health/social care (not hospital)<br>Hospital (inpatient)<br>Hospital (outpatient)	Choosing a medication<br>Prescribing guidelines<br>Progress review
Evaluating intervention: optimise prescribing	Hukins, 2019	Statins<br>Hypnotics/Z-drugs	Anticholinergic medications, anxiolytics, oestrogens, NSAID, antipsychotics, antiplatelets, proton pump inhibitors, benzodiazepines	Any tool (dementia-specific or non-disease-specific) to identify potentially inappropriate prescribing	Prevalence of polypharmacy, prevalence of PIP, most common prescribed PIP medications	Primary/health/ social care<br>Secondary health/social care (not hospital)<br>Hospital (inpatient)<br>Hospital (outpatient)<br>Care home<br>Patient home	Identifying health or prescribing concern<br>Progress review<br>Decision to stop treatment: supported
Evaluating intervention: optimise prescribing*	Pantoja, 2019	Benzodiazepines<br>Hypnotics/Z-drugs<br>Statins<br>Antihypertensives	Anticonvulsants, antibiotic therapy, NSAID, nicotine gum, ezetimibe, thromboprophylaxis	Manual paper reminders to improve compliance with preventive guidelines and disease management guidelines	Patient-important endpoints: death, development of a pulmonary embolism , surrogate or intermediate endpoints: achievement of target blood pressure or serum cholesterol level, markers of disease/health status, adverse effects, resource use, changes in professional practice, patient outcomes	Primary/health /social care<br>Hospital (inpatient)<br>Hospital (outpatient)	Progress review<br>Identifying health or prescribing concern<br>Initial assessment<br>Prescribing guidelines

For peer review only - http://bmjopen.bmj.com/site/about/guidelines.xhtml

Focus/ Aim	Study (First author, date)	Medications of interest	Other medications included	Intervention name/s and aim	Outcomes measured	Setting/Context	Relevant part of care pathway
Evaluating intervention: optimise prescribing*	Legare, 2010	Statins	Not clear	Interventions for improving the adoption of shared decision making by healthcare professionals	Healthcare professionals' adoption of SDM	Primary/health/ social care<br>Secondary health/social care (not hospital)	Initial assessment<br>Information and/or advice<br>Choosing a medication<br>Shared decision making<br>Decision to start agreed treatment plan
Evaluating intervention: optimise prescribing	Jeffery, 2015	Statins<br>Antihypertensives	NA	Interventions to improve adherence to CVD guidelines and patient outcomes: education, audit and feedback, academic detailing, comprehensive interventions that included education, audit and feedback and an academic detailing component	Guideline adherence, clinical outcomes	Primary/health/ social care<br>Secondary health/social care (not hospital)<br>Hospital (inpatient)Hospital (outpatient)	Identifying health or prescribing concern<br>Initial assessment<br>Information and/or advice<br>Prescribing guidelines<br>Progress review
Evaluating intervention: optimise prescribing	Shanbhag, 2018	Antihypertensives	NA	Implementation interventions classified at the provider, organisational or health system levels. Aim: increasing physician adherence to the specified HF guideline recommendations	Patient-level outcomes. Primary outcomes: process indicators (assessing guideline-consistent activities undertaken by a provider, proportion of patients who were: prescribed a guideline-recommended pharmacological treatment, referred for implantable cardioverter defibrillator, CRT consideration, provided self-care education at discharge, LVEF. Secondary outcomes: HF	Hospital (inpatient)<br>Hospital (outpatient)	Prescribing guidelines<br>Decision to start agreed treatment plan<br>Other

For peer review only - http://bmjopen.bmj.com/site/about/guidelines.xhtml

Focus/ Aim	Study (First author, date)	Medications of interest	Other medications included	Intervention name/s and aim	Outcomes measured	Setting/Context	Relevant part of care pathway
					specific/all-cause clinical outcomes		

*A secondary aim or one of multiple aims of review. Green highlighted text=High Overall Quality AMSTAR-2, Turquoise highlighted text=Medium Overall Quality AMSTAR-2, Orange highlighted text=Low Overall Quality AMSTAR-2, Red highlighted text=Critically-low Overall Quality AMSTAR-2. ACEI= Angiotensin-Converting Enzyme Inhibitors, AE=Adverse Events, AIDS= Acquired Immunodeficiency Syndrome, BEZRA=Benzodiazepines/Zdrugs, BP=Blood Pressure, CBT=Cognitive Behavioural Therapy, CPM=Continuous Passive Motion, CRT= Cardiac Resynchronisation Therapy, CV=Cardiovascular, CVD=Cardiovascular Disease, DBI=Drug Burden Index, ED=Emergency Department, GDR=Gradual Dose Reduction, GP=General Practitioner, HF=Heart Failure, HIV= Human Immunodeficiency Virus, HT=Hypertension, LDL= Low-Density Lipoprotein, LOS=Hospital Length of Stay, NA=Not Applicable, NR=Not Reported, QOL=Quality Of Life, LVEF= Left Ventricular Ejection Fraction, MBCBT=Mindfulness Based Cognitive Behavioural Therapy, NSAID=Non-Steroidal Anti-Inflammatories, PIP=Potentially Inappropriately Prescribed, SAE=Serious Adverse Events, SDM=Shared Decision Making, SMBP=Self-Monitoring of Blood Pressure, TKA=Total Knee Arthroplasty

For peer review only

Table 5: Systmatic reviews scoring negatively on one CEESAT item which syntehsised studies evaluating the effectiveness of an intervention

Focus/ Aim	Study (First author, date)	Medications of interest	Other medications included	Intervention name/s and aim	Outcomes measured	Setting/Context	Relevant part of care pathway
**Evaluating intervention: deprescribing**	Page, 2016	Statins Antihypertensives	Clopidogrel, Cilostazol, pentoxifylline, diuretics, nitrates, Glucosamine, bisphosphonates, calcium, vitamin D, Calcitriol, conjugated equine estrogens Premarin combined with medroxyprogesterone acetate, Carbamazepine, Levodopa, lithium, anticholinestera, prednisolone, corticosteroids, d beta-2 receptor agonist, antipsychotics, benzodiazepines antidepressants	Deprescribing by a health care professional of one or more regular prescription medications:	Mortality, adverse drug withdrawal events, physical health, cognitive function and psychological health, quality of life	Hospital (inpatient) Care home Primary/health/ social care Hospital (outpatient) Patient home	Progress review Decision to stop treatment: supported

For peer review only - http://bmjopen.bmj.com/site/about/guidelines.xhtml

Focus/ Aim	Study (First author, date)	Medications of interest	Other medications included	Intervention name/s and aim	Outcomes measured	Setting/Context	Relevant part of care pathway
Evaluating intervention: enhance adherence	Park, 2017	Antihypertensives<br>Statins	Antidementia medication, pneumonia-related ambulatory antibiotics, antilpemics, insulin, biologics, oral diabetes drugs, antiasthmatics, pain reliever, phosphate binders, cinacalce, teriparatide, antidepressants	Medicare Part D: increase drug utilization and lower Medicare and Medicaid participants out- of-pocket prescription drug costs, thereby improving access	Out-of-pocket costs, drug utilization	Primary/health/social care<br>Hospital (inpatient)<br>Hospital (outpatient)	Choosing a medication<br>Patient adherence<br>Progress review
Evaluating intervention: enhance adherence	Conn, 2015[70]	Statins<br>Antihypertensives	Yes, in relation to following medical conditions: organ transplant, epilepsy, gastrointestinal, infections, chronic diseases, malaria, STD, neurological, HIV	Packaging interventions to improve adherence: blister packs, unit-packaging, unit-of-use systems, unit-of-dose packaging,<br>monitored dosage systems	Adherence, health outcomes	Primary/health/social care<br>Hospital (inpatient)<br>Patient home	Patient adherence

Focus/ Aim	Study (First author, date)	Medications of interest	Other medications included	Intervention name/s and aim	Outcomes measured	Setting/Context	Relevant part of care pathway
Evaluating intervention: enhance adherence	Conn, 2015[71]	Statins *Inferred from population disease characteristics* Antihypertensives *Inferred from population disease characteristics*	Yes, in relation to following medical conditions: diabetes, HIV, renal disease, asthma, lung disease. stroke, gastrointestinal diseases, acute illness, allergies, autoimmune disease, blood disorders, cancer, eye problems, gynaecological disorders, liver disease, malaria, nervous system problems, osteoarthritis, osteoporosis, rheumatoid arthritis, seizures, skin diseases, organ transplant, tuberculosis, and vascular disease	Health care provider targeted interventions to improve medication adherence: Research staff trained providers, integration of care across providers, increased providers, communication skills, monitor patients adherence behaviour, Provider- patient medication concordance, Increased provider time with patients, reduce the geographical distance between providers and patients, increase continuity of care, activating provider	Adherence behaviour	Primary/health/social care	Accessing care<br>Working with other health or social care services/ charities<br>Shared decision making<br>Decision to start agreed treatment plan<br>Patient adherence<br>Progress review

For peer review only - http://bmjopen.bmj.com/site/about/guidelines.xhtml

Focus/ Aim	Study (First author, date)	Medications of interest	Other medications included	Intervention name/s and aim	Outcomes measured	Setting/Context	Relevant part of care pathway
Evaluating intervention: enhance adherence	Conn, 2016	Statins<br>Antihypertensives<br>*All Inferred from participant sample characteristics*	Yes, in relation to following medical conditions: gallbladder disease, unspecified chronic illnesses, osteoarthritis, asthma, IBD, glaucoma, kidney transplant, anti-coagulant therapy, osteoarthritis stroke, diabetes, HIV, antidepressants	Medication Adherence Interventions: Intervention content could include: prompts/cues to administer medications, self-monitoring of medication administration, self-monitoring of disease symptoms, written instructions, rewards for increased adherence, increased communication between providers and patients, providing feedback to participants about their adherence, goal setting about adherence, habit assessment/modification, and problem solving about adherence challenges	Adherence	Primary/health/social care<br>Secondary health/social care (not hospital)<br>Patient home	Decision to start agreed treatment plan<br>Patient adherence<br>Progress review
Evaluating intervention: enhance adherence	De Simoni, 2013	Antihypertensives	NA	Interventions to improve adherence to antihypertensive medications: education, lifestyle, verbal information/advice on disease and secondary-prevention drug treatment, goal setting, supply of printed information/advice material, screening for depression, personalized instructions, and integrated care	Adherence and blood pressure control	Primary/health/social care<br>Hospital (outpatient)<br>Patient home	Information and/or advice<br>Decision to start agreed treatment plan<br>Patient adherence<br>Progress review

For peer review only - http://bmjopen.bmj.com/site/about/guidelines.xhtml

Focus/ Aim	Study (First author, date)	Medications of interest	Other medications included	Intervention name/s and aim	Outcomes measured	Setting/Context	Relevant part of care pathway
Evaluating intervention: enhance adherence	Kassavou, 2018	Statins Antihypertensives	Yes, NR	Automated telecommunication systems, including voice messaging and text messaging: promote adherence to cardio-metabolic medications	Adherence	Primary/health/social care Secondary health/social care (not hospital) Hospital (inpatient) Hospital (outpatient) Patient home	Patient adherence
Evaluating intervention: enhance adherence	Krack, 2019	Statins Antihypertensives	Calcium channel blockers, Clopidogrel, insulin oral antidiabetics, diabetes drugs, inhaled steroids, metformin, thiazides, Inhaled corticosteroid	Value-based health insurance designs including: VBID vs usual medication coverage VBID+education vs usual medication coverage, VBID+education vs usual medication coverage+education<br><br>Aim: increase patients understanding of importance of high-value medications, increase number of adherent patients without increasing total health care spending	Adherence	Primary/health/social care	Information and/or advice Patient adherence
Evaluating interventio n: enhance	Kronish, 2011	Antihypertensives	NA	Drug Class: improve adherence to antihypertensive medication	Adherence	NR	Choosing a medication Patient adherence

For peer review only - http://bmjopen.bmj.com/site/about/guidelines.xhtml

Focus/ Aim	Study (First author, date)	Medications of interest	Other medications included	Intervention name/s and aim	Outcomes measured	Setting/Context	Relevant part of care pathway
Evaluating Intervention: enhance adherence	Lawrence, 2015	Statins Antihypertensives	Antithrombotic medication	Multimodal behavioural interventions for secondary stroke prevention: addresses: 1) medication education and/or medication compliance education, 2) education or active information provision e.g. about stroke, stroke (lifestyle) risk factors, and 3) one or more of four specified lifestyle behaviours i.e. smoking, diet, physical inactivity, and alcohol consumption, and/or behaviours associated with amelioration of lifestyle risk factors i.e. medication compliance and management of perceived psychosocial stress	Primary outcomes of interest: physiological outcomes e.g. blood pressure, blood lipids, and lifestyle behaviour change. Secondary outcomes: psychosocial outcomes e.g. anxiety, learning e.g. knowledge of lifestyle risk factors for stroke, incidence of vascular events and mortality	Secondary health/social care (not hospital) Patient home	Information and/or advice Patient adherence Progress review
Evaluating intervention: enhance	Mann, 2014	Statins Antihypertensives	NA	Drug Insurance Cost Sharing Strategies: lower expenditures for prescription drug insurance plans	Adherence, clinical events (myocardial infarction, stroke, death), QOL, healthcare utilization, or cost	NR	Accessing care Decision to start agreed treatment plan Patient adherence

For peer review only - http://bmjopen.bmj.com/site/about/guidelines.xhtml

Focus/ Aim	Study (First author, date)	Medications of interest	Other medications included	Intervention name/s and aim	Outcomes measured	Setting/Context	Relevant part of care pathway
Evaluating intervention: enhance adherence	Milosavljevic, 2018	Antihypertensives	Yes, in relation to following conditions: Asthma, diabetes, dyslipidemia, COPD, 'older people's health' aldosterone-inhibiting diuretics (for heart failure), intravenous vitamin D analogs, calcimimetic, oral hypoglycemics, antipsychotics, other central nervous system drugs, beta blockers, analgesics, antidepressants	Community pharmacist interventions:<br>improve adherence, improve clinical outcomes.	Behavioural outcome: adherence, self-efficacy. Clinical outcome: clinical biomarkers (e.g. blood pressure, glycosylated haemoglobin, LDL), hospitalisation rates, mortality, emergency room visits, markers of disease progress. Economic outcome: cost effectiveness analysis, or other relevant analysis. Humanistic outcome: patient quality of life, knowledge, or satisfaction	Primary/health/social care Patient home	Information and/or advice Choosing a medication Decision to start agreed treatment plan Progress review Patient adherence

For peer review only - http://bmjopen.bmj.com/site/about/guidelines.xhtml

Focus/ Aim	Study (First author, date)	Medications of interest	Other medications included	Intervention name/s and aim	Outcomes measured	Setting/Context	Relevant part of care pathway
Evaluating intervention: enhance adherence	Rash, 2016	Statins	NA	Interventions targeting medication adherence:<br>Prescription cost coverage, simplification of drug regimen, reminders, education-based, multi-faceted interventions (including one or more of following: education, counselling, behavioural interventions, follow-up, medication review, risk-factor and adherence education, personalized health reports, bi-monthly mailings, collaborative care)	Patient adherence	Primary/health/social care<br>Secondary health/social care (not hospital)<br>Hospital (inpatient)<br>Hospital (outpatient)	Information and/or advice<br>Working with other health/social care services<br>Choosing a medication<br>Shared decision making<br>Patient adherence<br>Progress review
Evaluating interventio n: enhance	Reeves, 2020	Antihypertensives	NA	Pharmacist-led interventions to improve medication adherence and BP control	BP control, medication adherence	Primary/health/social care<br>Hospital (inpatient)<br>Hospital (outpatient)	Information and/or advice<br>Patient adherence<br>Progress review
Evaluating intervention: enhance adherence*	Cheema, 2014	Antihypertensives	NA	Interventions to improve management of BP: Pharmacological: education on drug treatment of BP, advice to patients to improve medication adherence, identifying drug adverse effects and drug prescribing issues, liaising with prescribers about concerns of drug treatment.<br>Nonpharmacological: education about hypertension. education about lifestyle (advice to patients on diet, weight management, alcohol consumption, smoking cessation)	Systolic and diastolic BP, adherence, identification and management of drug-related problems, cardiovascular risk factors, i.e. smoking, alcohol consumption	Primary/health/social care	Information and/or advice<br>Working with other health/social care services<br>Identifying health or prescribing concern<br>Patient adherence<br>Progress review

For peer review only - http://bmjopen.bmj.com/site/about/guidelines.xhtml

Focus/ Aim	Study (First author, date)	Medications of interest	Other medications included	Intervention name/s and aim	Outcomes measured	Setting/Context	Relevant part of care pathway
Evaluating intervention: optimise prescribing	Nguyen, 2018	Statins	Aspirin, antiplatelet agents	Organisational or professional interventions to enhance prescribing of guideline-recommended medications. Interventions included: Distribution of educational materials, educational outreach visits, audit and feedback and reminders, continuity of care, communication and case discussions between distant healthcare professionals, distribution of published/printed recommendations for clinical care, including clinical practice guidelines	Proportion of patients receiving guideline-recommended medications. Secondary outcomes: proportion of patients achieving target blood pressure and target LDL, C/cholesterol level, mortality rate	Primary/health/social care<br>Hospital (outpatient)	Identifying health or prescribing concern<br>Initial assessment<br>Information and/or advice<br>Choosing a medication<br>Prescribing guidelines
Evaluating intervention: optimise prescribing<br>Evaluating intervention: enhance	King, 2018	Statins<br>Antihypertensives	Diabetic medication, Digoxin, mixed medications, antidepressants	Longer-duration (2–4 months) versus shorter-duration (28-day)prescriptions to improve patient outcomes	Health outcomes, adverse events, medication adherence, medication wastage, professional administration time, pharmacists time and/or costs, patient experience, and patient out-of-pocket costs	Primary/health/social care<br>Secondary health/social care (not hospital)<br>Hospital (inpatient)	Initial assessment<br>Choosing a medication<br>Decision to start agreed treatment plan<br>Patient adherence<br>Progress review

*Secondary aim or outcome within the review. AE=Adverse Events, BI=Behavioural Interventions, BP=Blood Pressure, BZ=Benzodiazepine, CBT=Cognitive Behavioural Therapy, COPD=Coronary Obstructive Pulmonary Disease, DBT=Dialectical Behavioural Therapy, ED=Emergency Department, GP=General Practitioner, HIV= Human Immunodeficiency Virus, IBD=Irritable Bowel Disease, LDL= Low-Density Lipoprotein, LTOT=Long-Term Opioid Therapy, MDD=Major Depressive Disorder, MI=Motivational Interviewing, NA=Not Applicable, NR=Not Reported, QOL=Quality of Life, VBID=Value Based Insurance Design

For peer review only - http://bmjopen.bmj.com/site/about/guidelines.xhtml

Table 6: Systematic reviews scoring negatively on 2-4 CEESAT items which synthesised studies evaluating the effectiveness of an intervention

Focus/ Aim	Study (First author, date)	Medications of interest	Other medications included	Intervention name/s and aim	Outcomes measured	Setting/Context	Relevant part of care pathway
**Evaluating intervention: deprescribing*.**	Hart, 2020	Antihypertensives	Antipsychotics, Antihistamines, Antivertigo, Anti-Parkinson, Urinary spasmolytics, Vasodilators, Digoxin, Diuretics, Beta Blockers, Hypoglycemics, Beta blocker eye drops, Alpha adrenergic antagonists, Alpha blockers, Benzodiazepines Opiates Hypnotics/Z-drugs Antidepressants	Interventions to reduce falls or use of FRIDs: Face-to-face medication consultations with community pharmacy resident, fall-related assessment performed by research physician, geriatrician assessment of falls risk and systematic medication review, consideration of potential FRIDs for withdrawal+prescribing physician contact	Change in FRID use	Hospital (inpatient) Hospital (outpatient)	Initial assessment Choosing a medication Progress review Decision to stop treatment: supported Other *Medication review in acute care (e.g. after hip fracture)*
**Evaluating intervention: enhance adherence**	Al Alshaikh, 2016	Statins Antihypertensives	Aspirin, Dipyridamole, antithrombotics, antiplatelets, anticoagulants, diabetic medications	Multimodal Interventions to improve adherence: categories included educational+motivational, simplification of drug regimen, environmental cues or reminders+reducing concerns and misbeliefs regarding medications, combined interventions to enhance risk factor management after stroke, prescribed medications+educated about them	Medication adherence	Primary/health/social care Hospital (inpatient) Hospital (outpatient) Patient home	Information and/or advice Patient adherence

Focus/ Aim	Study (First author, date)	Medications of interest	Other medications included	Intervention name/s and aim	Outcomes measured	Setting/Context	Relevant part of care pathway
Evaluating intervention: enhance adherence	Baumgartner, 2020	Statins<br>Antihypertensives	Yes, in relation to following medical conditions:<br>HIV, diabetes, tuberculosis<br><br>Specific medications include:<br>metformin/sulfonylurea, various oral antidiabetic drugs, pioglitazone/ thiazolidinedione+ sulfonurea, FDC: rosiglitazone/glimepiride, glyburide/metformin, thiazolidinedione, Î±-blocker/antimuscarinic	Polypill to improve medication adherence	Adherence	Primary/health/social care<br>Hospital (inpatient)<br>Hospital (outpatient)	Choosing a medication<br>Decision to start agreed treatment plan<br>Patient adherence<br>Progress review
Evaluating intervention: enhance adherence	Bochkareva, 2019	Antihypertensives	NA	Medical Assistance Scheme to increase adherence to treatment: Examples include<br>special packaging of medications, amount of prescribed medications, interventions to improve communication with patients (more frequent visits, motivational interviewing, patient education, home BP monitoring, and provision of written instructions), duration and number of sessions/consultations	Adherence	Primary/health/social care<br>Secondary health/social care (not hospital)<br>Hospital (outpatient)	Information and/or advice<br>Patient adherence

Focus/ Aim	Study (First author, date)	Medications of interest	Other medications included	Intervention name/s and aim	Outcomes measured	Setting/Context	Relevant part of care pathway
Evaluating intervention: enhance adherence	Checchi, 2014	Antihypertensives Statins	Various HAARTs, luticasone propionate, travoprost, pilocarpine, antidiabetic medications, ipratropium bromide, lithium, Clopidogrel, bupropion-SR, antipsychotics, fluticasone, sulfonylureas, immuno-suppressants	Electronic medication packaging devices to promote medication adherence	Adherence, health outcomes, cost effectiveness results, and satisfaction	Primary/health/social care<br>Patient home	Patient adherence<br>Information and/or advice
Evaluating intervention: enhance adherence	Conn 2015[36]	Antihypertensives	NA	Interventions to increase adherence among adults with hypertension	Adherence	Primary/health/social care<br>NR	Information and/or advice<br>Patient adherence<br>Progress review<br>Referral to specialist care

For peer review only - http://bmjopen.bmj.com/site/about/guidelines.xhtml

Focus/ Aim	Study (First author, date)	Medications of interest	Other medications included	Intervention name/s and aim	Outcomes measured	Setting/Context	Relevant part of care pathway
Evaluating intervention: enhance adherence	Deichmann, 2016	Statins	Fibrates, Anion-exchange resins or bile acid sequestrants, Niacin or nicotinic acid, combined medication regimen	Interventions to increase adherence to self-administered lipid-lowering medication: Drug regimen simplification, patient education and information, intensified patient care with reminders via mail, telephone, and hand-held pill devices, complex behavioural approaches, group sessions, decision support systems, administrative improvements, pharmacy-led automated telephone intervention	Adherence to lipid-lowering medical therapy. Secondary outcomes: physiologic indicators (e.g., total cholesterol), health outcome indications (e.g., QOL, morbidity, mortality), adverse effects, implications for costs	Primary/health/social care<br>Patient home	Information and/or advice<br>Choosing a medication<br>Patient adherence<br>Progress review
Evaluating intervention: enhance adherence	Du, 2018	Antihypertensives	NA	Fixed-dose combination versus free-equivalent combination therapies on adherence to medication for hypertension	Medication adherence/persistence	NR	Choosing a medication<br>Patient adherence<br>Progress review
Evaluating intervention: enhance adherence	Gupta, 2010	Antihypertensives	NA	Fixed-Dose Combinations of Antihypertensive agents. Aim: to achieve better BP control by improving compliance	Compliance (or adherence), persistence, BP-lowering efficacy, adverse effects	NR	Choosing a medication<br>Patient adherence<br>Progress review

For peer review only - http://bmjopen.bmj.com/site/about/guidelines.xhtml

Focus/ Aim	Study (First author, date)	Medications of interest	Other medications included	Intervention name/s and aim	Outcomes measured	Setting/Context	Relevant part of care pathway
Evaluating intervention: enhance adherence	Guzman-Tordecilla, 2020	Antihypertensives	NA	Interventions to enhance adherence and control of arterial hypertension: community fund organization, reminders, educational material and calendar, sessions for the optimization of the use of medicines, advice with experienced pharmacists, group educational meetings, obtention of medical records, identification of problems with medications and intervention on them, follow-up with the pharmacist and education	Adherence, control of HBP	Primary/health/social care<br>Patient home	Information and/or advice<br>Working with other health or social care services/charities<br>Patient adherence<br>Progress review
Evaluating interventio n: enhance adherence	Kawalec, 2018	Antihypertensives	NA	Fixed-dose combination therapy. Aim: blood pressure lowering, increasing patients adherence	Medication adherence, blood pressure lowering, persistence, health outcomes and adverse events	Primary/health/social care<br>Patient home	Choosing a medication<br>Patient adherence<br>Progress review
-Evaluating intervention: enhance adherence	Leslie, 2016	Antihypertensives<br>Statins (assumed)	Oral diabetes medications	Interventions aimed to modify medication adherence behaviour to chronic medications administered orally to treat diabetes, hypertension, or hyperlipidaemia: behavioural counselling, education and Informational materials, medication packaging that increased convenience by combining pills in a blister pack	Adherence to medications	Primary/health/social care<br>Care home<br>Patient home	Information and/or advice<br>Patient adherence<br>Progress review

For peer review only - http://bmjopen.bmj.com/site/about/guidelines.xhtml

Focus/ Aim	Study (First author, date)	Medications of interest	Other medications included	Intervention name/s and aim	Outcomes measured	Setting/Context	Relevant part of care pathway
Evaluating intervention: enhance adherence	Mamudu, 2014	Statins<br>Antihypertensives	Diabetic medications, Aspirin	Coronary artery calcium screening: enable risk stratification, diagnosis of CAD and directs the treatment and management of CAD among asymptomatic individuals	Behavioural modification, risk perception, and medication adherence, lifestyle change	Primary/health/social care	Identifying health or prescribing concern<br>Initial assessment<br>Patient adherence
Evaluating intervention: enhance adherence	Tsioufis, 2020	Antihypertensives	NA	Single-pill combination therapy to treat hypertension. Aim: improve clinical outcomes and medication adherence	Adherence and/or persistent, BP, cardiovascular outcomes, mortality or morbidity	Primary/health/social care	Choosing a medication<br>Patient adherence<br>Progress review
Evaluating intervention: enhance adherence	Uhlig, 2013	Antihypertensives	NA	Interventions to enhance management of hypertension: self-measured blood pressure with or without additional support	Clinical outcomes (e.g. death and cardiovascular events), patient-reported outcomes (e.g. patient satisfaction or QOL), surrogate outcomes (e.g. measures of left ventricular hypertrophy), intermediate outcomes (e.g. BP; number, dose, or changes of antihypertensive medications; adherence to antihypertensive medication), health care utilization (including visits, calls, and e-mails	Primary/health/social care<br>Patient home	Patient adherence<br>Progress review

For peer review only - http://bmjopen.bmj.com/site/about/guidelines.xhtml

Focus/ Aim	Study (First author, date)	Medications of interest	Other medications included	Intervention name/s and aim	Outcomes measured	Setting/Context	Relevant part of care pathway
Evaluating intervention: enhance adherence	Xiong, 2018	Antihypertensives	Immunosuppressant's (n=1), lipid-lowering medication (n=1)	Mobile health technology to enhance medication adherence	Medication adherence	Primary/health/social care<br>Secondary health/social care (not hospital)<br>Hospital (outpatient)<br>Patient home	Patient adherence
Evaluating intervention : enhance adherence	Xu, 2018	Antihypertensives	NA	Medication education, self-monitoring of BP, reminders and regular follow-up visits to improve medication adherence	Adherence, BP, BP control	Primary/health/social care<br>Hospital (outpatient) *might be inpatient*<br>Patient home	Patient adherence<br>Information and/or advice<br>Progress review
Evaluating intervention: enhance adherence	Demonceau, 2013	Antihypertensives *Assumed*	Medications for following medications:: HIV, asthma, osteoporosis<br>Specific medications: sulfonylurea drugs, oral nitrates	Interventions to affect adherence to self-administered prescribed medications in short-term and in long-term therapy: treatment simplification, cognitive-educational interventions, behavioural counselling, social-psycho-affective interventions, electronically monitored adherence feedback, technical reminder systems, monitoring the disease being managed, rewards, integrating depression treatment with hypertension management or type 2 diabetes care, home-based physician-directed, nurse-guided drug therapy, drug regimen	Health outcomes' e.g. reductions in blood pressure, lower levels of glycosylated haemoglobin, medication adherence	Primary/health/social care<br>Hospital (inpatient)<br>Hospital (outpatient)<br>Patient home	Information and/or advice<br>Working with other health/social care services<br>Patient adherence<br>Referral to specialist care

For peer review only - http://bmjopen.bmj.com/site/about/guidelines.xhtml

Focus/ Aim	Study (First author, date)	Medications of interest	Other medications included	Intervention name/s and aim	Outcomes measured	Setting/Context	Relevant part of care pathway
Evaluating intervention: enhance adherence<br>Evaluating intervention: optimise prescribing	Laba, 2013	Statins<br>Antihypertensives	Yes<br>*Not all medicines reported, but all for CVD*<br>e.g. spironolactone, loop diuretics, digoxin	Strategies to improve patient adherence to cardiovascular medications:<br>education, persuasion, incentivisation, enablement, training, coercion, restriction, environmental restructuring, modelling, policies, communication/marketing, guidelines, fiscal, regulation, legislation, environmental/social planning, service provision	Adherence, Physiological: BP control, mean DBP/SBP, lipids, glucose/HbA1c, INR, echocardiogram, health outcomes, health service utilisation, mortality, QOL, other lifestyle, smoking/alcohol, nutrition, physical activity, stress, adverse events, cost comparison, cost effectiveness	Primary/health/social care<br>Patient home<br>Hospital (outpatient)	Information and/or advice<br>Shared decision making<br>Patient adherence<br>Progress review<br>Prescribing guidelines
Evaluating intervention: enhance adherence	Polinski, 2011	Statins<br>Antihypertensives	HEDIS, high risk drugs, anti-convulsants, antihistamines, propoxyphene, digoxin, NSAIDs, doxazosin, muscle relaxants/anti-spasmodics, others, warfarin, oral anti-diabetics, antiretroviral medications, warfarin, clopidogrel, antipsychotics, proton pump inhibitors, antibiotics, benzodiazepines antidepressants, hypnotics/Z-drugs (sedatives)	Medicare Part D cost-sharing provisions and drug coverage rules. Aim: improve access to essential medications through reduced cost-sharing with focus on under and over-use of specific drugs and classes	Reduced out-of-pocket costs, health	Primary/health/social care<br>Hospital (outpatient)	Accessing care<br>Choosing a medication<br>Decision to start agreed treatment plan<br>Patient adherence<br>Decision to stop treatment: unsupported

For peer review only - http://bmjopen.bmj.com/site/about/guidelines.xhtml

Focus/ Aim	Study (First author, date)	Medications of interest	Other medications included	Intervention name/s and aim	Outcomes measured	Setting/Context	Relevant part of care pathway
Evaluating intervention: optimise prescribing	Kang, 2016	Statins<br>Antihypertensives	Aspirin	Pharmacist-involved care (patient- targeted services) and healthcare professional-targeted services: PTS-counselling, providing educational materials, medical reminders, post-discharge follow- up services and supporting rehabilitation transition care. HTS-medication reconciliation, communication with health-care providers<br>Aim: improve CVD outcomes	All-cause mortality, all-cause hospitalization, cardiac-related hospitalization, key medication prescription rates, health-related QOL, medication adherence, medication utilization	Primary/health/social care<br>Hospital (inpatient)<br>Hospital (outpatient)<br>Patient home	Information and/or advice<br>Working alongside health/social care services<br>Choosing a medication<br>Patient adherence<br>Progress review
Evaluating intervention: optimise prescribing	Chhina, 2013	Antihypertensives	Propoxyphene, cerebral & peripheral vasodilators, cephalexin, amoxycillin with or without clavulinic acid, cephalexin, doxycycline, erythromycin, penicillin, rrimethoprim, cefaclor, roxithromycin, NSAIDS, metronidazole, omeprazole, inhaled steroids, antipsychotic, benzodiazepines antidepressants	Academic Detailing to modify drug prescription behaviour of Family Physicians in primary care settings	Prescription rate	Primary/health/social care	Choosing a medication<br>Progress review<br>Other<br>*Reducing repeat prescription*

Focus/ Aim	Study (First author, date)	Medications of interest	Other medications included	Intervention name/s and aim	Outcomes measured	Setting/Context	Relevant part of care pathway
Evaluating intervention: optimise prescribing* Evaluating intervention: enhance adherence*	Yuan, 2019	Antihypertensives	Yes, associated with following medical conditions: diabetes, chronic obstructive pulmonary disease, osteoporosis, obesity, antiphospholipid syndrome, drug related high risk of disease, outpatients taking multiple prescription drugs, bronchial asthma	Community Pharmacist interventions to improve outcomes for people with hypertension: medication review, patient education, adherence assessment, health/lifestyle advice, physical assessment (e.g., BP), monitoring, prescribing, or adjusting and administering therapy	Clinical outcomes of pharmaceutical services e.g. common end- points of systolic blood pressure, diastolic blood pressure and glycosylated haemoglobin after providing CPS	Primary/health/soci al care<br>Patient home<br>Hospital (inpatient)	Initial assessment<br>Information and/or advice<br>Decision to start agreed treatment plan<br>Patient adherence<br>Progress review

*Secondary aim or one of multiple aims; [a]Note that focus is on 'use' which includes initiating prescribing. Drugs of interest may be currently under- or over-used, [b]One aspect of broad aim, [c]One aspect of interventions. BP=Blood Pressure, CAD=Coronary Artery Disease, CPS=Community Pharmacist Services, CVD=Cardiovascular Disease, COX-2=Cyclooxygenase-2, DBP-Diastolic Blood Pressure, FDC=Fixed-Dose Combination, FRID=Fall Risk Increasing Drugs, HAART= Highly Active Antiretroviral Therapy, HBP=High Blood Pressure, HIV= Human Immunodeficiency Virus, HRT=Hormonal Replacement Therapy, HTS=Healthcare professional Targeted Services, INR=International Normalised Ratio, NA=Not Applicable, NR=Not Reported, NSAID=Non-Steroidal Anti-Inflammatories,PTS=Patient Targeted Services, SBP=Systolic Blood Pressure, SH=Sedative Hypnotics, SR=Sustained Release, QOL=Quality of Life,

For peer review only - http://bmjopen.bmj.com/site/about/guidelines.xhtml

Table 7: Systematic reviews synthesising qualitative evidence

Study (First author, date)	Medications of interest	Other medications included	Perspectives obtained	Phenomenon of Interest	Setting/Context	Relevant part of care pathway
Ingersgaard, 2020	Statins<br>Antihypertensives	Lipid-lowering agents, Î² blockers, angiotensin, renin system blockers, aspirin	Patient	Experiences/views of an intervention (adherence)<br>Reasons for adherence/non-adherence	Primary/health/social care<br>NR	Patient adherence
Rashid, 2014	Statins	Clopidogrel, antiplatelet medication, aspirin	Patient<br>Family member	Reasons for adherence/non-adherence	Primary/health/social care<br>Hospital (outpatient)<br>Secondary health/social care (not hospital)<br>Patient home	Patient adherence<br>Decision to stop treatment: unsupported

Study (First author, date)	Medications of interest	Other medications included	Perspectives obtained	Phenomenon of Interest	Setting/Context	Relevant part of care pathway
Kinnear, 2019	Statins	NA	Patient<br>Carer<br>Family member	Experiences/views of an intervention (adherence)<br>Reasons for adherence/non-adherence	Primary/health/social care<br>Secondary health/social care (not hospital)	Initial assessment<br>Information and/or advice<br>Choosing a medication<br>Shared decision making<br>Patient adherence<br>Progress review<br>Decision to stop treatment: unsupported
Reeve, 2013	Antihypertensives	Any long-term medication, methadone (substitution program), antiepileptic’s, HRT, PPIs, donepezil, benzodiazepines antidepressants	Patient<br>Carer	Experiences/views of an intervention (deprescribing)	NR	Decision to stop treatment: supported
Dhar, 2017	Antihypertensives	NA	Patient	Reasons for adherence/non-adherence	Primary/health/social care<br>Secondary health/social care (not hospital)<br>Hospital (outpatient)<br>Patient home	Patient adherence<br>Decision to stop treatment: unsupported

For peer review only - http://bmjopen.bmj.com/site/about/guidelines.xhtml

Study (First author, date)	Medications of interest	Other medications included	Perspectives obtained	Phenomenon of Interest	Setting/Context	Relevant part of care pathway
Marshall, 2012	Antihypertensives	NA	Patient	Reasons for adherence/non-adherence	Primary/health/social care<br>Hospital (outpatient)<br>Patient home	Choosing a medication<br>Decision to start agreed treatment plan<br>Patient adherence<br>Decision to stop treatment: supported<br>Decision to stop treatment: unsupported
Khatib, 2014	Antihypertensives	NA	Practitioner/Prescriber<br>Patient	Prescribing<br>Of an intervention (adherence)<br>Reasons for adherence/non-adherence	Primary/health/social care<br>Hospital (outpatient)<br>Patient home	Identifying health or prescribing concern<br>Accessing care<br>Initial assessment<br>Information and/or advice<br>Choosing a medication<br>Shared decision making<br>Prescribing guidelines<br>Decision to start agreed Patient adherence<br>Progress review

For peer review only - http://bmjopen.bmj.com/site/about/guidelines.xhtml

Study (First author, date)	Medications of interest	Other medications included	Perspectives obtained	Phenomenon of Interest	Setting/Context	Relevant part of care pathway
Oori, 2019	Antihypertensives	NA	Patient	Reasons for adherence/non-adherence	Care home<br>Primary/health/social care-<br>Secondary health/social care (not hospital)<br>Hospital (outpatient)<br>Patient home	Patient adherence

For peer review only

Study (First author, date)	Medications of interest	Other medications included	Perspectives obtained	Phenomenon of Interest	Setting/Context	Relevant part of care pathway
Rashid, 2018	Antihypertensives	Yes, relevant to the following conditions: asthma, renal transplant, proton pump inhibitors, schizophrenia, migraine, antiretroviral for HIV, osteoporosis, anxiety, malaria prophylaxis, bipolar, back pain, ADHD, dysphagia, IBD, RA, breast cancer, anticoagulants, borderline personality disorder, Specific drugs mentioned: clozapine, sumatriptan, SSRI, HAART, malaria prophylaxis, antibiotics, phosphate binding, insulin, clopidogrel, PMTCT, chemotherapy, Aripiprazole, Antidepressants	Practitioner/Prescriber<br>Patient<br>Carer<br>Family member	Experiences/views of an intervention (adherence)<br>Taking a medication	Primary/health/social care<br>Secondary health/social care (not hospital)<br>Hospital (inpatient)<br>Hospital (outpatient)	Accessing care<br>Initial assessment<br>Information and/or advice<br>Patient adherence<br>Progress review

* Data too thin for synthesis. Green highlighted text=High Overall Quality as appraised by AMSTAR-2, Turquoise highlighted text=Moderate Overall Quality as appraised by AMSTAR-2.
ADHD=Attention-Deficit Hyperactivity Disorder, BZ=Benzodiazepine, HAART= Highly Active Antiretroviral Therapy, HIV= Human Immunodeficiency Virus, IBD=Irritable Bowel Disease, NA=Not Applicable, NR=Not Reported, PMTCT=Prevention of Mother-to-Child Transmission, RA=Rheumatoid Arthritis, SSRI=Selective Serotonin Reuptake Inhibitor

For peer review only - http://bmjopen.bmj.com/site/about/guidelines.xhtml

APPENDIX E: QUALITY APPRAISAL OF INCLUDED STUDIES

Table 8: Scores on CEESAT items included studies - original search

First Author, Date	Search strategy: Is approach to searching clearly defined, systematic and transparent?	Is search comprehensive?	Does the review critically appraise each study?	During critical appraisal was an effort made to minimise subjectivity?	Total Score
Dills, 2018	Green	Green	Green	Green	0
Hukins, 2019	Green	Green	Green	Green	0
Pantoja, 2019	Green	Green	Green	Green	0
Posadzki, 2016	Green	Green	Green	Green	0
Stacey, 2017	Green	Green	Green	Green	0
Adler, 2017	Green	Green	Green	Green	0
de Cates, 2014	Green	Green	Green	Green	0
Fletcher, 2015	Green	Green	Green	Green	0
Jeffery, 2015	Green	Green	Green	Green	0
Kinnear, 2019	Green	Green	Green	Green	0
Legare, 2010	Green	Green	Green	Green	0
Maimaris, 2013	Green	Green	Green	Green	0
Mallat, 2016	Green	Green	Green	Green	0
Morrissey, 2017	Green	Green	Green	Green	0
Nieuwlaat, 2014	Green	Green	Green	Green	0
Palmer, 2018	Green	Green	Green	Green	0
Sanders, 2011	Green	Green	Green	Green	0
Shanbhag, 2018	Green	Green	Green	Green	0
van Driel, 2016	Green	Green	Green	Green	0
King, 2018	Green	Green	Amber	Green	1
Milosavljevic, 2018	Green	Green	Amber	Green	1
Page, 2016	Green	Green	Amber	Green	1
Park, 2017	Green	Green	Amber	Green	1
Cheema, 2014	Green	Green	Amber	Green	1

For peer review only - http://bmjopen.bmj.com/site/about/guidelines.xhtml

Conn, 2015[38]	Amber	Green	Green	Green	1
Conn, 2015[40]	Amber	Green	Green	Green	1
Conn, 2016	Amber	Green	Green	Green	1
de Simoni, 2013	Green	Amber	Green	Green	1
Ingersgaard, 2020	Green	Amber	Green	Green	1
Kassavou, 2018	Green	Green	Green	Red	1
Krack, 2019	Green	Green	Green	Red	1
Kronish, 2011	Green	Green	Amber	Green	1
Lawrence, 2015	Green	Green	Green	Red	1
Mann, 2014	Green	Green	Green	Red	1
Nguyen, 2018	Green	Amber	Green	Green	1
Rash, 2016	Green	Green	Green	Red	1
Reeve,s 2020	Amber	Green	Green	Green	1
Loganathan, 2011	Green	Amber	Amber	Green	2
Mamudu, 2014	Green	Green	Red	Red	2
Oori, 2019	Amber	Green	Amber	Green	2
Xu, 2018	Green	Amber	Amber	Green	2
Checchi, 2014	Green	Amber	Green	Red	2
Demonceau, 2013	Green	Amber	Amber	Green	2
Rashid, 2018	Green	Amber	Amber	Green	2
Reeve, 2013	Green	Green	Amber	Red	2
Baumgartner, 2020	Green	Amber	Green	Red	2
Bochkareva, 2019	Green	Green	Amber	Red	2
Deichmann, 2016	Amber	Green	Amber	Green	2
Khatib, 2014	Green	Amber	Green	Red	2
Laba, 2013	Green	Green	Amber	Red	2
Rashid, 2014	Green	Amber	Amber	Green	2
Tsioufis, 2020	Green	Amber	Green	Red	2
Uhlig, 2013	Green	Amber	Amber	Green	2
Yuan, 2019	Amber	Amber	Green	Green	2
Hart, 2020	Amber	Amber	Amber	Green	3
Polinski, 2011	Amber	Amber	Amber	Green	3
Al Alshaikh, 2016	Amber	Amber	Amber	Green	3
Conn, 2015	Amber	Green	Amber	Red	3
Dhar, 2017	Amber	Green	Amber	Red	3
Gupta, 2010	Amber	Amber	Amber	Green	3

For peer review only - http://bmjopen.bmj.com/site/about/guidelines.xhtml

Guzman-Tordecilla, 2020	Amber	Amber	Green	Red	3
Kang, 2016	Amber	Amber	Amber	Green	3
Marshall, 2012	Green	Amber	Amber	Red	3
Xiong, 2018	Green	Amber	Amber	Red	3
Du,2019	Amber	Amber	Amber	Red	4
Chhina, 2013.	Amber	Amber	Amber	Red	4
Kawalec, 2018	Amber	Amber	Amber	Red	4
Leslie, 2016[7]	Amber	Amber	Amber	Red	4

For peer review only

For peer review only - http://bmjopen.bmj.com/site/about/guidelines.xhtml

Table 9: Scores on AMSTAR-2 tool for reviews included in map with CEESAT score of 0

Study (First author, date)	1. PICO components	2. Protocol	3. Study design explanation	4. Comprehensive search strategy	5. Duplicate study selection	6. Duplicate data extraction	7. Details of excluded studies	8. Description of included studies	9b. RoB assessment (NRSIs)	10. Funding sources	9a. Risk of Bias (RoB) assessment (RCTs)	11a. RCTs Meta-analysis	11b. NRSIs Meta-analysis (MA)	12. MA: RoB in individual studies	13. Qualitative reviews: Is synthesis methodology /theoretical framework clearly described /justified?	14. Qualitative reviews: was description /rationale for sampling described?	15. Qualitative reviews: was process of data extraction clearly described?	16. Qualitative reviews: was process for coding data clearly described?	17. Qualitative reviews: was process of developing themes clearly described?	18. Qualitative reviews: Do authors reflect on how their theoretical /ideological perspectives may influence their findings?	19 Qualitative reviews: Was use of any software clearly described?	20. RoB: discussion of results	21. Heterogeneity	22. Publication bias	23. Reports conflicts of interest	Overall rating
Adler, 2017	Y	Y	N	Y	Y	Y	Y	Y	NA	Y	Y	N	NA	NA	NA	NA	NA	NA	NA	NA	NA	Y	Y	NA	Y	H
de Cates, 2014	Y	PY	N	Y	Y	Y	Y	Y	NA	N	Y	Y	NA	Y	NA	NA	NA	NA	NA	NA	NA	Y	Y	Y	Y	H
Dills 2018,	Y	N	N	Y	Y	Y	N	Y	NA	Y	Y	N	NA	NA	NA	NA	NA	NA	NA	NA	NA	Y	Y	NA	Y	L
Fletcher, 2015	Y	Y	Y	Y	Y	Y	N	PY	N	N	Y	Y	N	Y	NA	NA	NA	NA	NA	NA	NA	Y	Y	N	Y	CL
Hukins, 2019	Y	N	N	Y	Y	Y	N	Y	Y	N	NA	NA	N	NA	NA	NA	NA	NA	NA	NA	NA	Y	Y	NA	Y	L
Jeffery, 2015	Y	Y	N	Y	Y	Y	N	Y	Y	N	Y	Y	Y	Y	NA	NA	NA	NA	NA	NA	NA	Y	Y	N	Y	M
Kinnear, 2019	Y	Y	NA	Y	Y	Y	N	Y	Y	Y	NA	NA	N	NA	PY	NA	Y	Y	Y	N	Y	Y	Y	NA	Y	H
Legare, 2010	Y	Y	N	Y	Y	Y	Y	Y	NA	N	Y	N	NA	NA	NA	NA	NA	NA	NA	NA	NA	Y	Y	NA	Y	H
Maimaris, 2013	Y	Y	Y	Y	Y	Y	N	PY	PY	N	Y	N	N	NA	NA	NA	NA	NA	NA	NA	NA	Y	Y	NA	Y	M
Mallat, 2016	Y	Y	N	Y	Y	Y	N	Y	NA	Y	Y	Y	NA	Y	NA	NA	NA	NA	NA	NA	NA	Y	Y	Y	Y	H
Morrissey, 2017	Y	Y	N	Y	Y	Y	N	Y	NA	N	Y	N	NA	Y	NA	NA	NA	NA	NA	NA	NA	Y	Y	Y	Y	L

Study (First author, date)	1. PICO components	2. Protocol	3. Study design explanation	4. Comprehensive search strategy	5. Duplicate study selection	6. Duplicate data extraction	7. Details of excluded studies	8. Description of included studies	9b. RoB assessment (NRSIs)	10. Funding sources	9a. Risk of Bias (RoB) assessment (RCTs)	11a. RCTs Meta-analysis	11b. NRSIs Meta-analysis (MA)	12. MA: RoB in individual studies	13. Qualitative reviews: Is synthesis methodology /theoretical framework clearly described /justified?	14. Qualitative reviews: was description /rationale for sampling described?	15. Qualitative reviews: was process of data extraction clearly described?	16. Qualitative reviews: was process for coding data clearly described?	17. Qualitative reviews: was process of developing themes clearly described?	18. Qualitative reviews: Do authors reflect on how their theoretical /ideological perspectives may influence their findings?	19 Qualitative reviews: Was use of any software clearly described?	20. RoB: discussion of results	21. Heterogeneity	22. Publication bias	23. Reports conflicts of interest	Overall rating
Nieuwlaat, 2014	Y	Y	N	Y	Y	Y	Y	Y	NA	N	Y	N	NA	NA	NA	NA	NA	NA	NA	NA	NA	Y	Y	NA	Y	M
Palmer, 2018	Y	Y	Y	Y	Y	Y	Y	Y	NA	Y	Y	N	NA	NA	NA	NA	NA	NA	NA	NA	NA	Y	Y	NA	Y	H
Pantoja, 2019	Y	Y	N	Y	Y	Y	Y	Y	Y	Y	Y	N	N	NA	NA	NA	NA	NA	NA	NA	NA	Y	Y	NA	Y	H
Posadzki, 2016	Y	Y	Y	Y	Y	Y	Y	Y	Y	Y	Y	Y	Y	Y	NA	NA	NA	NA	NA	NA	NA	Y	Y	N	Y	H
Sanders, 2011	Y	N	N	Y	Y	Y	Y	Y	PY	Y	PY	Y	Y	N	NA	NA	NA	NA	NA	NA	NA	N	Y	N	Y	CL
Shanbhag, 2018	Y	Y	N	Y	Y	Y	N	PY	Y	N	Y	N	N	NA	NA	NA	NA	NA	NA	NA	NA	N	Y	NA	Y	L
Stacey, 2017	Y	Y	N	Y	Y	Y	Y	Y	NA	N	Y	Y	NA	Y	NA	NA	NA	NA	NA	NA	NA	Y	Y	Y	Y	M
van Driel, 2016	Y	Y	N	Y	Y	Y	Y	Y	NA	Y	Y	Y	NA	Y	NA	NA	NA	NA	NA	NA	NA	Y	Y	Y	Y	H

CL=Critically Low overall quality; H=High overall quality; Low overall quality; M=Moderate overall quality; N=No; NA=Not Applicable; NRSI=Non-Randomised Studies of healthcare Interventions; PICO=Population, Intervention, Comparator, Outcome; PY=Partial Yes; RCT=Randomised Controlled Trial; RoB=Risk of Bias; Y=Yes

For peer review only - http://bmjopen.bmj.com/site/about/guidelines.xhtml

For peer review only

For peer review only - http://bmjopen.bmj.com/site/about/guidelines.xhtml

PRISMA 2020 Checklist

Section and Topic	Item #	Checklist item	Location where item is reported
**TITLE**			
Title	1	Identify the report as a systematic review.	1
**ABSTRACT**			
Abstract	2	See the PRISMA 2020 for Abstracts checklist.	2
**INTRODUCTION**			
Rationale	3	Describe the rationale for the review in the context of existing knowledge.	4
Objectives	4	Provide an explicit statement of the objective(s) or question(s) the review addresses.	4
**METHODS**			
Eligibility criteria	5	Specify the inclusion and exclusion criteria for the review and how studies were grouped for the syntheses.	7-8
Information sources	6	Specify all databases, registers, websites, organisations, reference lists and other sources searched or consulted to identify studies. Specify the date when each source was last searched or consulted.	6
Search strategy	7	Present the full search strategies for all databases, registers and websites, including any filters and limits used.	Supplementary Materials
Selection process	8	Specify the methods used to decide whether a study met the inclusion criteria of the review, including how many reviewers screened each record and each report retrieved, whether they worked independently, and if applicable, details of automation tools used in the process.	6-7
Data collection process	9	Specify the methods used to collect data from reports, including how many reviewers collected data from each report, whether they worked independently, any processes for obtaining or confirming data from study investigators, and if applicable, details of automation tools used in the process.	7-8
Data items	10a	List and define all outcomes for which data were sought. Specify whether all results that were compatible with each outcome domain in each study were sought (e.g. for all measures, time points, analyses), and if not, the methods used to decide which results to collect.	8
	10b	List and define all other variables for which data were sought (e.g. participant and intervention characteristics, funding sources). Describe any assumptions made about any missing or unclear information.	8
Study risk of bias assessment	11	Specify the methods used to assess risk of bias in the included studies, including details of the tool(s) used, how many reviewers assessed each study and whether they worked independently, and if applicable, details of automation tools used in the process.	7-8
Effect measures	12	Specify for each outcome the effect measure(s) (e.g. risk ratio, mean difference) used in the synthesis or presentation of results.	8
Synthesis methods	13a	Describe the processes used to decide which studies were eligible for each synthesis (e.g. tabulating the study intervention characteristics and comparing against the planned groups for each synthesis (item #5)).	8-9
	13b	Describe any methods required to prepare the data for presentation or synthesis, such as handling of missing summary statistics, or data conversions.	na
	13c	Describe any methods used to tabulate or visually display results of individual studies and syntheses.	8
	13d	Describe any methods used to synthesize results and provide a rationale for the choice(s). If meta-analysis was performed, describe the model(s), method(s) to identify the presence and extent of statistical heterogeneity, and software package(s) used.	na
	13e	Describe any methods used to explore possible causes of heterogeneity among study results (e.g. subgroup analysis, meta-regression).	na
	13f	Describe any sensitivity analyses conducted to assess robustness of the synthesized results.	na
Reporting bias assessment	14	Describe any methods used to assess risk of bias due to missing results in a synthesis (arising from reporting biases).	na

For peer review only

PRISMA 2020 Checklist

Section and Topic	Item #	Checklist item	Location where item is reported
Certainty assessment	15	Describe any methods used to assess certainty (or confidence) in the body of evidence for an outcome.	na
**RESULTS**			
Study selection	16a	Describe the results of the search and selection process, from the number of records identified in the search to the number of studies included in the review, ideally using a flow diagram.	10
	16b	Cite studies that might appear to meet the inclusion criteria, but which were excluded, and explain why they were excluded.	Supplementary Materials
Study characteristics	17	Cite each included study and present its characteristics.	10-13
Risk of bias in studies	18	Present assessments of risk of bias for each included study.	10, Supplementary Materials
Results of individual studies	19	For all outcomes, present, for each study: (a) summary statistics for each group (where appropriate) and (b) an effect estimate and its precision (e.g. confidence/credible interval), ideally using structured tables or plots.	NA
Results of syntheses	20a	For each synthesis, briefly summarise the characteristics and risk of bias among contributing studies.	Supplementary Materials, 10-13
	20b	Present results of all statistical syntheses conducted. If meta-analysis was done, present for each the summary estimate and its precision (e.g. confidence/credible interval) and measures of statistical heterogeneity. If comparing groups, describe the direction of the effect.	na
	20c	Present results of all investigations of possible causes of heterogeneity among study results.	na
	20d	Present results of all sensitivity analyses conducted to assess the robustness of the synthesized results.	na
Reporting biases	21	Present assessments of risk of bias due to missing results (arising from reporting biases) for each synthesis assessed.	na
Certainty of evidence	22	Present assessments of certainty (or confidence) in the body of evidence for each outcome assessed.	na
**DISCUSSION**			
Discussion	23a	Provide a general interpretation of the results in the context of other evidence.	na
	23b	Discuss any limitations of the evidence included in the review.	14
	23c	Discuss any limitations of the review processes used.	14
	23d	Discuss implications of the results for practice, policy, and future research.	14-15
**OTHER INFORMATION**			
Registration and protocol	24a	Provide registration information for the review, including register name and registration number, or state that the review was not registered.	4
	24b	Indicate where the review protocol can be accessed, or state that a protocol was not prepared.	4
	24c	Describe and explain any amendments to information provided at registration or in the protocol.	8
Support	25	Describe sources of financial or non-financial support for the review, and the role of the funders or sponsors in the review.	1
Competing interests	26	Declare any competing interests of review authors.	1

For peer review only - http://bmjopen.bmj.com/site/about/guidelines.xhtml

PRISMA 2020 Checklist

Section and Topic	Item #	Checklist item	Location where item is reported
Availability of data, code and other materials	27	Report which of the following are publicly available and where they can be found: template data collection forms; data extracted from included studies; data used for all analyses; analytic code; any other materials used in the review.	1

From: Page MJ, McKenzie JE, Bossuyt PM, Boutron I, Hoffmann TC, Mulrow CD, et al. The PRISMA 2020 statement: an updated guideline for reporting systematic reviews. BMJ 2021;372:n71. doi: 10.1136/bmj.n71

For more information, visit: http://www.prisma-statement.org/

For peer review only

BMJ Open

What is the quantity, quality and type of systematic review evidence available to inform the optimal prescribing of statins and antihypertensives? A systematic umbrella review and evidence and gap map

Journal:	*BMJ Open*
Manuscript ID	bmjopen-2023-072502.R2
Article Type:	Original research
Date Submitted by the Author:	01-Dec-2023
Complete List of Authors:	Shaw, Liz; University of Exeter, Exeter Policy Research Programme Evidence Review Facility<br>Briscoe, Simon; University of Exeter, Exeter Policy Research Programme Evidence Review Facility<br>Nunns, Michael; University of Exeter, Exeter Policy Research Programme Evidence Review Facility<br>Lawal, Hassanat; University of Exeter, Exeter Policy Research Programme Evidence Review Facility<br>Melendez-Torres, G.J; University of Exeter, Exeter Policy Research Programme Evidence Review Facility<br>Turner, Malcolm; University of Exeter, Exeter Policy Research Programme Evidence Review Facility; University of Exeter, NIHR ARC South West Peninsula Patient and Public Engagement group<br>Garside, Ruth ; University of Exeter, Exeter Policy Research Programme Evidence Review Facility; University of Exeter, European Centre for Environment and Health<br>Thompson Coon, Jo; University of Exeter, Exeter Policy Research Programme Evidence Review Facility
**Primary Subject Heading**:	Cardiovascular medicine
Secondary Subject Heading:	Cardiovascular medicine, Evidence based practice, Global health, Pharmacology and therapeutics
Keywords:	Systematic Review, Hypertension < CARDIOLOGY, STROKE MEDICINE

SCHOLARONE™
Manuscripts

I, the Submitting Author has the right to grant and does grant on behalf of all authors of the Work (as defined in the below author licence), an exclusive licence and/or a non-exclusive licence for contributions from authors who are: i) UK Crown employees; ii) where BMJ has agreed a CC-BY licence shall apply, and/or iii) in accordance with the terms applicable for US Federal Government officers or employees acting as part of their official duties; on a worldwide, perpetual, irrevocable, royalty-free basis to BMJ Publishing Group Ltd ("BMJ") its licensees and where the relevant Journal is co-owned by BMJ to the co-owners of the Journal, to publish the Work in this journal and any other BMJ products and to exploit all rights, as set out in our licence.

The Submitting Author accepts and understands that any supply made under these terms is made by BMJ to the Submitting Author unless you are acting as an employee on behalf of your employer or a postgraduate student of an affiliated institution which is paying any applicable article publishing charge ("APC") for Open Access articles. Where the Submitting Author wishes to make the Work available on an Open Access basis (and intends to pay the relevant APC), the terms of reuse of such Open Access shall be governed by a Creative Commons licence – details of these licences and which Creative Commons licence will apply to this Work are set out in our licence referred to above.

Other than as permitted in any relevant BMJ Author's Self Archiving Policies, I confirm this Work has not been accepted for publication elsewhere, is not being considered for publication elsewhere and does not duplicate material already published. I confirm all authors consent to publication of this Work and authorise the granting of this licence.

For peer review only

What is the quantity, quality and type of systematic review evidence available to inform the optimal prescribing of statins and antihypertensives? A systematic umbrella review and evidence and gap map

Liz Shaw[1*], Simon Briscoe[1], Michael Nunns[1], Hassanat Lawal[1], G.J. Melendez-Torres[1], Malcolm Turner[1,2], Ruth Garside[1,3], Jo Thompson Coon[1],

[1]Exeter Policy Research Programme Evidence Review Facility, Faculty of Health and Life Sciences, St Lukes Campus, University of Exeter, Exeter, Devon, EX1 2LU, UK

[2] NIHR ARC South West Peninsula Patient and Public Engagement group, Faculty of Health and Life Sciences, St Lukes Campus, University of Exeter, Exeter, Devon, EX1 2LU

[3] European Centre for Environment and Health, Faculty of Health and Life Sciences, Knowledge Spa, Truro, TR1 3HD

*Corresponding author: E.H.Shaw@exeter.ac.uk, 01392 72 2396

Keywords: Statins, antihypertensives, mapping review

Word count (Excluding abstract, strengths/limitations, tables, figures, reference and appendices): 3254

Number of Figures: 3

Number of Tables: 2

For peer review only

For peer review only - http://bmjopen.bmj.com/site/about/guidelines.xhtml

ABSTRACT

Objectives: We aimed to map the systematic review evidence available to inform the optimal prescribing of statins and antihypertensive medication.

Design: Systematic umbrella review and evidence and gap map (EGM).

Data sources: Eight bibliographic databases (Cochrane Database of Systematic Reviews, CINAHL, EMBASE, Health Management Information Consortium, MEDLINE ALL, PsychInfo, Conference Proceedings Citation Index – Science and Science Citation Index) were searched from 2010 to 11th August 2020. Update searches conducted in MEDLINE ALL 2nd August 2022. We searched relevant websites and conducted backwards citation chasing.

Eligibility criteria for selecting studies: We sought systematic reviews of quantitative or qualitative research where adults 16 years+ were currently receiving, or being considered for, a prescription of statin or anti-hypertensive medication. Eligibility criteria were applied to the title and abstract and full text of each article independently by two reviewers.

Data extraction and synthesis: Quality appraisal was completed by one reviewer and checked by a second. Review characteristics were tabulated and incorporated into an EGM based on a patient care pathway. Patients with lived experience provided feedback on our research questions and evidence and gap map.

Results: Sixty-eight reviews were included within the EGM. The highest quantity of evidence focused on evaluating interventions to promote patient adherence to antihypertensive medication. Key gaps included a lack of reviews synthesising evidence on experiences of specific interventions to promote patient adherence or improve prescribing practice. The evidence was predominantly of low quality, limiting confidence in the findings from individual reviews.

Conclusions: This EGM provides an interactive, accessible format for policy developers, service commissioners and clinicians to view the systematic review evidence available relevant to optimising the prescribing of statin and antihypertensive medication. To address the paucity of high-quality research, future reviews should be conducted and reported according to existing guidelines and address the evidence gaps identified above.

For peer review only - http://bmjopen.bmj.com/site/about/guidelines.xhtml

STRENGTHS AND LIMITATIONS

- Our evidence and gap map (EGM) represents the results of a comprehensive, systematic search for systematic review evidence in this field.
- The evidence and gap map includes only systematic review evidence and thus there may be primary research relevant to the topic which may sit within the identified gaps.
- Our search strategy did not capture systematic reviews which may have met our eligibility criteria that were used to inform NICE guidelines relevant to the review topic. These reviews were not retrieved by our bibliographic database searches, thus highlighting issues regarding the lack of visibility of the reviews contributing to NICE guidelines.

For peer review only

For peer review only - http://bmjopen.bmj.com/site/about/guidelines.xhtml

INTRODUCTION

With an estimated 17.9 million deaths during 2019, cardiovascular disease (CVD) is the leading cause of death worldwide.[1] One study estimates the total costs of heart failure for 98.7% of the world's population to be $108 billion dollars.,[2] with individuals from black, Asian and other minority ethnic backgrounds, low socio-economic status or those living with diabetes or severe mental illness being more at risk of the disease.[3] Along with earlier detection of risk factors for CVD and promotion of lifestyle changes, high intensity statins are recommended over the use of low intensity statins as an effective treatment to prevent CVD.[4] Whilst a key target set by the World Health Organisation that a minimum 50% of eligible individuals will be in receipt of statins has been met in high-income countries,[5] the prescribing of this type of medication in the UK has not yet reached optimal levels.[4]

Hypertension is another risk factor in the development of CVD,[6] and can be controlled through use of antihypertensive medication, sometimes in combination with statins, to reduce the occurrence of CVD.[7] Whilst, the rate of hypertension remained largely unchanged between 1990 and 2019, the burden of illness shifted from high to middle- and low-income countries.[8] There are early signs that awareness and treatment of hypertension within some high-income countries, such as the UK, has decreased within the last five years.[9, 10] Patient non-adherence is also an issue of concern, with adherence decreasing with higher numbers of antihypertensive medications prescribed per patient.[11]

To achieve optimal prescribing of statins and antihypertensive medication, it is essential that the factors influencing both their prescription and patient adherence are fully understood. There is already a diverse range of existing systematic review evidence in this area which could be utilised by policy makers, commissioners and clinicians to design and deliver services or research to support the needs of those living with, or at risk of, CVD. To fully understand and make use of this existing evidence base, this paper focuses on the creation of an evidence and gap map to display the quantity, quality and type of quantitative and qualitative systematic review evidence available to inform the optimal prescribing of statins and antihypertensives.

We were interested in systematic reviews which synthesised the following:

- Evidence regarding the effectiveness or experiences of interventions intended to improve prescribing practices or patient adherence;
- Evidence on the effectiveness or experiences of interventions intended to improve implementation of interventions intended to improve prescribing practices or patient adherence;

For peer review only - http://bmjopen.bmj.com/site/about/guidelines.xhtml

- Evidence focusing on practitioner views or perceptions of making prescribing decisions;
- Guidelines intended to inform prescribing practise.

This paper is part of a broader systematic mapping review commissioned by NHS England and Improvement relating to the optimising of the prescribing of three different groups of medication: medications to treat cardiovascular disease, DCD (including opioids, benzodiazepines, gabapentinoids and non-benzodiazepine hypnotics) and antidepressants.[12]

For peer review only

For peer review only - http://bmjopen.bmj.com/site/about/guidelines.xhtml

METHODS

The methods used for the searching, screening, data extraction and quality appraisal processes of the evidence included in this evidence and gap map were in accordance with the University of York's Centre for Reviews and Dissemination guidelines.[13] We also consulted guidance for the development of evidence and gap maps.[14] Our methods were outlined in our pre-registered review protocol and are summarised below according to PRISMA reporting guidance.[15, 16]

Search strategy

The first stage of our systematic umbrella review involved conducting searches of bibliographic databases and websites for relevant literature. Our bibliographic database search strategy combined terms for optimising prescribing with terms for statins and antihypertensives and CVD. The search strategy was developed using MEDLINE (via Ovid) using a combination of free-text terms and controlled headings. Search terms were derived from pre-identified relevant papers and inspected by stakeholders with expertise on optimising prescribing. Searches were run on 11th August 2020 in the following bibliographic databases: Cochrane Database of Systematic Reviews (via Cochrane Library), CINAHL (via EBSCO), EMBASE (via Ovid), Health Management Information Consortium (via Ovid), MEDLINE ALL (via Ovid), PsychInfo (via Ovid), Conference Proceedings Citation Index – Science and Science Citation Index (both via Web of Science, Clarivate Analytics). We also searched systematic review database Epistemonikos and preprint server medRxiv. This selection of databases reflects our desire to comprehensively search for systematic review evidence within this field and the broad scope of an umbrella review. The databases selected provide coverage of a variety of health care disciplines relevant to our research question.

An historical date limit of 2010 was applied to all search results to prioritise the most recent systematic reviews, evaluating interventions and experiences most relevant to current medical settings, for inclusion in the EGM. We carried out backwards citation chasing for all reviews which met our inclusion criteria, searched topically relevant websites and pursued full texts for conference abstracts and review protocols identified through our searches. We also contacted review authors where we could not access full-texts. Update searches were conducted in MEDLINE ALL (via OVID) on 2nd August 2022 and 23rd June 2023. We limited our update search to MEDLINE since all the studies identified through our original search were indexed in this database. Full details of database search strategies are reproduced in Appendix A (which also includes search terms for drugs that cause dependency, which was a second topic that our client asked us to investigate in our mapping review. The findings associated with this second topic are available elsewhere).

For peer review only - http://bmjopen.bmj.com/site/about/guidelines.xhtml

Study selection

After running our search strategy, the second stage of our umbrella review involved two reviewers (LS, MN) screening a sample of 100 title and abstracts to ascertain the utility of our inclusion criteria for this review. The revised inclusion criteria (Table 1), were then applied independently by two reviewers to each citation retrieved via our search strategy (LS, MN), with disagreements resolved through discussion or referral to a third reviewer (SB). Eligibility of full texts were assessed using the same method. Screening decisions were recorded in Endnote X8 software (Clarivate Analytics, Philadephia, PA, USA) and the study selection process detailed within a PRISMA-style flowchart.

Table 1: Review inclusion criteria

PICO/PICo criteria	Inclusion criteria
Population	Mean age ≥ 16<br>Receiving/being considered for a prescription for statins and/or antihypertensives.
Intervention/Phenomenon of interest	For systematic reviews of quantitative evidence, interventions which aimed to improve one or more of the following:<br>- Adherence to prescribed medication of interest;<br>- Discontinuation of medications of interest;<br>- Prescriber adherence to clinical guidance for prescribing;<br>- Prescriber practices;<br>- Implementation of an intervention to enhance patient adherence or prescriber practices.<br><br>Interventions could be conducted at the level of system or that of the patient/prescriber.<br><br>For systematic reviews of qualitative evidence, review foci could include the views or experiences of patients, carers or clinicians for:<br>- Healthcare consultations to discuss initiation, reviewing or discontinuing a prescription;<br>- Interventions to improve adherence*/prescribing practice;<br>- Reasons for adherence or non-adherence to prescribed medication;<br>- Making prescribing decisions.
Comparator/Context	Any
Outcome	Relevant to the aims of our review and stated clearly in the study abstract.<br><br>For reviews of quantitative evidence, examples may include:<br>- Measures of patient adherence;<br>- Measures of prescriber adherence to prescribing guidelines;<br>- Measures of success of implementing interventions to promote adherence, prescribing or discontinuation<br><br>For reviews of qualitative evidence, please see phenomenon of interest section above.

For peer review only - http://bmjopen.bmj.com/site/about/guidelines.xhtml

.Study design	Systematic reviews which met the following criteria:<br>- Clearly stated research question;<br>- Indicate which sources were searched;<br>- Reproducible search strategy and search date;<br>- Clearly defined inclusion and exclusion criteria and study selection methods;<br>- Critically appraise and report the quality of included studies;<br>- Reproducible data synthesis strategy.<br><br>Encompasses: systematic reviews of quantitative and qualitative literature, systematic reviews of reviews, systematic reviews of guidelines, scoping and rapid reviews.
Date limit	Systematic reviews published before 2010 were excluded. This was to prioritise the most recent systematic reviews, evaluating interventions and experiences most relevant to current medical settings, for inclusion in the EGM

*For convenience, we use the term "patient adherence" throughout this report to encapsulate this continual decision-making process in which the patient can choose, and/or be enabled, to play an active role when taking their medication as prescribed.

Data extraction and quality appraisal

After title and abstract and full-text screening of the articles identified via our search strategy, two reviewers carried out a pilot of our data extraction form on five reviews. The finalised data extraction and quality appraisal form was applied to all included systematic reviews by one reviewer (MN, LS) and checked by a second (SB, LS, MN), supported by EPPI-Reviewer (v 4.11.5.2) software. Disagreements were resolved through discussion or referral to a third person.

We then conducted quality appraisal in two stages. Reviews were first appraised using four modified criteria from the Collaboration for Environmental Evidence Synthesis Assessment Tool (CEESAT):[17]

1. Search strategy: Is the approach to searching clearly defined, systematic and transparent?
2. Is the search comprehensive?
3. Does the review critically appraise each study?
4. During critical appraisal was an effort made to minimise subjectivity?

Only systematic reviews which met all four criteria were prioritised for full quality appraisal using the AMSTAR-2,[18] modified to incorporate reporting standards for qualitative evidence synthesis.[19] Reviews appraised using only the CEESAT criteria were given an overall quality rating of 'Critically-Low". This process of quality appraisal represents a deviation from our protocol and was necessary due to the high number of studies eligible for inclusion in the EGM. Thus, we decided to utilise the

For peer review only - http://bmjopen.bmj.com/site/about/guidelines.xhtml

process described above to priories the inclusion of the most robust evidence for inclusion in the EGM.

Full details of the data extracted from each review, including CEESAT and AMSTAR-2 criteria, are provided elsewhere.[12]

Data analysis and presentation

In the final stage of our umbrella review, details of the systematic reviews prioritised for inclusion in the evidence and gap map were summarised in a table and described narratively. We used EPPI-Mapper software to structure the evidence and gap map according to medications of interest and patient care pathway.[20]

The initial care pathway was based upon NICE guidelines for statins and antihypertensives, optimising prescribing and shared decision making.[5, 21-23] To ensure the pathways reflected both recommended practice and implementation within ‘real-life’ settings, we also drew upon a systematic review exploring patient experiences of medication taking and consulted with patients and carers with experience of seeking, being prescribed, taking and/or discontinuing statins and/or antihypertensives.[24] We also asked clinicians if the pathway we developed reflected their clinical experience working within the area. Finally, we produced a condensed version of the map which focused on pre-treatment/initiation, maintenance, discontinuation and development of guidelines.

The map presents the systematic review evidence according to the ‘Overall Quality’ rating provided by the AMSTAR-2. The colour and size of the bubble indicate the quality rating and number of systematic reviews for that medication type at that part of the care pathway respectively. Systematic reviews relevant to more than one type of medication or position on the care pathway are represented more than once within the map. Filters also allow the map user to alter the type of evidence shown in the map. Policy, clinical and patient stakeholders were asked to comment on the presentation and content of the final evidence and gap map to ensure findings were accessible to our intended audiences.

Patient and public involvement

We worked alongside a group of stakeholders with a variety of expertise in the topic area throughout the production of this evidence and gap map. Stakeholders included representatives from NHSE-I, NICE, clinicians and people with experience of taking one or more of the medications of interest. Key stages at which all stakeholders, including patients and members of the public, contributed during the project included; development of the protocol, including providing feedback on the appropriateness of review questions, supporting development of the bibliographic database

For peer review only - http://bmjopen.bmj.com/site/about/guidelines.xhtml

search strategies and list of relevant topic websites to be searched. Clinical stakeholders and patients/members of the public also provided insight on the list of included studies, including resolving queries regarding eligibility for inclusion, supported the development of the patient care-pathway and also provided feedback on the presentation of the evidence and gap map and gaps in systematic review evidence within the evidence and gap map and how findings should be disseminated.

For peer review only

For peer review only - http://bmjopen.bmj.com/site/about/guidelines.xhtml

RESULTS

Following deduplication, 1694 unique records were found through our bibliographic database and supplementary searches. 1516 of these were excluded during title and abstract screening, with a further 113 excluded after the reviewing of full-texts. Fourteen systematic reviews were identified through the search strategy intended to identify other medication groups as part of a wider piece of work (ref withheld for peer review). In total, 82 systematic reviews were eligible for inclusion in the review. These studies are presented within our evidence and gap map (access here, also shown in Figure 1). Figure 2 summarises the study selection process and reasons for exclusion at full-text, with a list of full reasons for exclusion provided in Appendix B.

[Insert Figure 1]

[Insert Figure 2]

Characteristics of evidence included within umbrella review

Medications of interest within the 82 reviews included in our umbrella review were: statins (n=10),[25-34] antihypertensives (n=38),[35-72] and statins and antihypertensives (n=34).[73-104] Nine of the reviews synthesised qualitative evidence,[27, 31, 42, 50, 55, 58, 60, 61, 83, 104] four of which also included quantitative evidence in a mixed evidence synthesis.[42, 50, 58, 61] Of the reviews synthesising quantitative evidence evaluating interventions, interventions focused on: enhancing patient adherence to medication as prescribed (n=57),[25, 30, 32-35, 37-41, 43-46, 48, 49, 51, 53, 54, 56, 57, 59, 62, 63, 65-71,, 73-81, 86, 87, 89, 90-95, 96,, 98-103, ,105] deprescribing a prescribed medication (n=6),[36, 46, 72, 80, 88, 93] or optimising the prescribing of medication to meet the needs of the patient (n=13).[26, 28, 29, 38, 52, 64, 71, 84, 85, 87, 90, 100, 106] Three of these reviews were judged by reviewers to have dual aims.[71, 87, 90] An overview of the characteristics of all reviews included in the evidence and gap map is included in Appendix C.

Characteristics of evidence included in evidence and gap map

Twenty-two of the 82 systematic reviews included in our umbrella review were fully appraised using the modified AMSTAR-2 tool. Ten were 'High' quality,[27, 28, 54, 72, 73, 81, 98, 100, 99, 103] five were of 'Moderate' quality,[32, 53, 65, 84, 95] four of 'Low' quality,[26, 57, 64, 82] and three 'Critically-Low' quality.[44, 603, 96] The remaining 60 reviews scored poorly on one or more of the CEESAT criteria during the first stage of quality appraisal, and were thus awarded a 'Critically-Low' overall quality rating. The full quality appraisal ratings for each review can be found in Appendices D and E.

For peer review only - http://bmjopen.bmj.com/site/about/guidelines.xhtml

Table 2 provides a summary of the systematic review evidence included in the evidence and gap map.

Table 2: Summary of included reviews

Topic/type of evaluation	Type of evidence	Number of reviews*	Appraised using AMSTAR-2 Y/N: N (Overall Quality rating)	Medication of interest
Prescriber, patient, and/or family/carer views of issues relating to prescribing and/or adherence to medications of interest	Qualitative	10*[1]	Y: 1 (High: 1)	Statins: 1
			N: 9	AH:6<br>Statins: 1<br>Statins+AH: 2
Evaluating intervention to optimise prescribing	Quantitative	15	Y: 6 (High4:, Moderate:1 , Low: 2)	AH: 2<br>Statins: 2<br>Statins+AH: 2
			N: 9	AH:3<br>Statins: 1<br>Statins+AH: 5
Evaluating intervention to deprescribe medication	Quantitative	5	Y: 1 (Critically low: 1)	Statins+AH: 1
			N: 4	AH:2<br>Statins+AH: 2
Evaluating intervention to optimise patient adherence to a medication	Quantitative	57	Y: 14 (High: 7, Moderate: 3, Low: 1, Critically low: 3)	AH: 8<br>Statins: 1<br>Statins+AH: 5
			N: 43	AH: 20<br>Statins: 4<br>Statins+AH: 19

*n>68 as some reviews were coded as having multiple aims; *[1]Four reviews also included quantitative evidence; AH=Antihypertensives; N=No; Y=Yes

Summary of evidence on the patient care pathway

Figure 3 presents a visual summary of the 68 reviews included in the evidence and gap map, presented according to the volume of evidence available which corresponds to the different aims of interest to this umbrella review. Further detail is summarised below.

[Insert Figure 3]

Summary of evidence and gaps

Figure 3 illustrates a large cluster of evidence relating to the evaluation of interventions to promote maintaining adherence to medication, particularly to treat hypertension. The next biggest cluster of evidence is relevant to the 'Pre-treatment' or 'Initiation' stage of the pathway with antihypertensive medication. However, overall the quality of the evidence at these segments of the care pathway was predominantly of critically-low quality.

For peer review only - http://bmjopen.bmj.com/site/about/guidelines.xhtml

The quantity of qualitative evidence available is low and, whilst the evidence examines the perceptions of patients, carers and/or clinicians of issues affecting medication prescription or adherence, none of the systematic reviews explore experiences of specific interventions intended to optimise prescribing in some way. Only one of the systematic reviews of qualitative evidence was of 'High' quality.

Pre-treatment/Initiation

Five reviews synthesised qualitative research.[27, 31, 50, 55, 104] One high quality review of qualitative evidence explored patient and family members views of the barriers and enablers to treatment adherence for heterozygous familial hypercholesterolaemia.[27] Three reviews of 'Critically Low' quality review synthesised qualitative evidence relevant to the prescribing of antihypertensives.[31, 50, 55] Two of these reviews included a range of different perspectives,[31, 50] whilst the other focused on patient perspectives.[55] One 'Critically Low' quality review explored practitioner and patient views of prescribing or adhering to statin and/or antihypertensive medication.[104]

Thirty-two systematic reviews synthesised quantitative evidence to promote medication adherence. Statins were the medication of interest for ten reviews, two of 'High' overall quality,[81, 103] and eight of 'Critically Low' overall quality.[33, 75, 87, 91, 93, 94, 101, 102] Antihypertensives were the medication of interest in twenty systematic reviews; three were of 'High' quality,[54, 81, 103] two of 'Moderate' overall quality,[53, 65] and fifteen were 'Critically Low' overall quality.[37, 43, 45, 49, 51, 56, 66, 71, 75, 87, 91, 94, 95, 101, 102] Ten reviews sat across both statin and antihypertensive care pathways.[74, 79, 80, 85, 88, 90, 91, 97, 98, 105]

Six systematic reviews synthesised quantitative evidence evaluating interventions to optimise prescribing, where the medication of interest was statins; 'High' quality (n=2),[28, 100] 'Moderate' quality (n=1),[84] and 'Critically Low' quality (n=3).[29, 85, 87] One 'Critically Low' quality review synthesised quantitative evidence evaluating interventions to reduce the prescribing of fall risk increasing drugs in older adults.[47] The medication of interest within one 'Critically Low' quality review was both statins and antihypertensives,[106] and antihypertensives alone in a high-quality review.[72]

Maintaining treatment

Two high quality,[27, 72] and seven 'Critically-low' quality,[31, 33, 50, 55, 80, 105, 106] reviews of qualitative evidence, included in the 'Pre-treatment/initiation' part of pathway, were also relevant here.

For peer review only - http://bmjopen.bmj.com/site/about/guidelines.xhtml

In addition, one ‘Critically-Low’ quality review explored reasons associated with adherence or non-adherence to statin medication,[61] and a further three ‘Critically-Low’ quality reviews combined quantitative and qualitative data to explore factors relating to adherence/nonadherence where antihypertensives and/or statins were the medication of interest.[16, 24, 52]

Twenty-five systematic reviews synthesised evidence evaluating interventions to improve adherence to statins. Five were of ‘High’ overall quality,[2, 13, 54, 58, 71] one of ‘Moderate’ overall quality,[51] and 19 of ‘Critically Low’ overall quality. In total, forty-nine systematic reviews synthesised quantitative evidence evaluating interventions to promote adherence to medications, which included antihypertensives Five were of ‘High’ overall quality,[54, 73, 81, 98, 103] three were ‘Moderate’ quality,[53, 65, 95] eight were of ‘Critically low’ quality,[34, 44, 46, 48, 57, 63, 67, 96,] and thirty-three 33 were of critically-low quality.[35, 37, 39-41, 43, 45, 46, 49, 51, 56, 62, 66, 68-71 74-76, 78, 79, 81, 86,87, 89-94, 101, 102]

Four ‘Critically Low’ quality synthesised quantitative evidence to optimise the prescribing of medications, including statins and antihypertensives.[85, 87, 90, 106] One ‘High’ quality review evaluated interventions to optimise adherence to both groups of medication.[98; 99] Two ‘Critically Low’ quality reviews, [38, 71] and one ‘High’ quality review synthesised evidence evaluating interventions to optimise the prescribing of antihypertensives alone.[72]

Discontinuing treatment

Three reviews, one of ‘Low’ quality,[82] five of ‘Critically Low’ quality,[36, 47, 88, 97, 105] synthesised quantitative evidence evaluating interventions promote deprescribing.

One systematic review of quantitative evidence evaluated impact of Medicare Part D on use of under and overuse of medication. Statins and antihypertensives were only two types of medication included in this review.[102]

One review of ‘Low’ quality focused on identifying potentially inappropriate prescribing in older adults with dementia.[26]

Two ‘Critically-Low’ quality reviews of qualitative evidence; one lay perspectives on hypertension and drug adherence,[55] one reported on patient and/or carer views barriers to and enablers of deprescribing where antihypertensives were the medication of interest.[61]

Two ‘Critically Low’ quality reviews synthesised quantitative evidence to optimise the prescribing of antihypertensives.[38, 52]

For peer review only - http://bmjopen.bmj.com/site/about/guidelines.xhtml

Prescribing guidelines

One ‘Critically low’ quality,[106] one ‘Moderate’ quality,[82] and one ‘High’ quality review, synthesised quantitative evidence evaluating interventions to improve adherence to CVD prescription guidelines.[72]

DISCUSSION

We aimed to provide a resource to enable policy makers, commissioners, clinicians and other decision makers to find and view existing systematic review evidence relevant to optimising the prescribing of statin and antihypertensive medication. The evidence and gap map displays systematic review evidence synthesising quantitative evidence evaluating interventions to improve or optimise medication adherence or prescribing and also qualitative evidence examining the perspectives of patients, carers and clinicians of issues which can affect optimal prescribing. This evidence is mapped onto a patient care pathway, according to the type of medication of interest, enabling map users to find and view the evidence most suited to their needs.

The map highlights a cluster of systematic review evidence evaluating interventions to promote adherence to medication, particularly antihypertensive, relevant to the “Pre-treatment” and “Maintaining treatment” sections of the patient care pathway. An identified gap within the pathway is that of qualitative evidence which seeks to explore the views and/or experiences of these types of intervention.

Strengths and limitations

This evidence and gap map will be a useful resource for decision makers who are seeking systematic review evidence to answer questions relating to the optimal prescribing of statins or antihypertensive medication. The maps functionality means that the key characteristics of the evidence are easily displayed and allow for map users to access a summary or link to full texts as required. The map also identifies both clusters of existing systematic review evidence and gaps, highlighting where further systematic review evidence may or may not be required. However, this map includes only systematic review evidence and thus there may be primary research relevant to the topic which may sit within the identified gaps. In addition, the majority of evidence in this map was of critically-low quality. These systematic reviews may be of more limited use to decision makers and mask gaps where further evidence syntheses or commissioning of primary research is required.

Our search strategy did not capture systematic reviews used to inform NICE guidelines relevant to the review topic. These reviews were not retrieved by our bibliographic database searches,

For peer review only

highlighting issues regarding the visibility of the reviews contributing to NICE guidelines. It was beyond our resources to search through individual guidelines to locate relevant reviews.

Implications of our findings

For research commissioners, the map highlights where further research may be useful, including the following specific areas:

- Further synthesis of existing systematic reviews of experiences of taking or adhering to statin and/or antihypertensive medication;
- Searching for primary studies where gaps in high quality systematic review evidence have been identified, for example, qualitative evidence regarding patient and/or prescriber experiences of specific interventions to increase medication adherence or practitioner experiences of prescribing statin medication.

The clusters of existing research may also be useful in informing government policy, however caution is required as many of the systematic reviews are of poor quality which may limit the confidence that can be placed in their findings.

For clinicians, the qualitative evidence within the map highlights factors which could influence patient's seeking out or accessing health services for CVD and their willingness or ability to take any medication which is prescribed to them. The map also includes systematic review evidence evaluating interventions to optimise medication adherence, which may also be useful to inform clinical practice. Patients may find the evidence and gap map a useful resource to enable them to review the research associated with the care they receive.

CONCLUSIONS

This evidence and gap map presents a snapshot of the systematic review evidence available to inform the optimal prescribing of statin and antihypertensive medication, displayed to indicate the quality and quantity of evidence available to inform decision making at key points in the patient care pathway. The evidence included within the map may support policy makers and service commissioners or aid in the commissioning of further research. The map may also help inform the prescribing decisions of clinicians. Whilst some caution is required when interpreting the content of the map given the limitations of the included evidence and methodology used within this mapping review as indicated above, this map provides a useful resource for a variety of stakeholders to access recent systematic review evidence in this topic area.

For peer review only - http://bmjopen.bmj.com/site/about/guidelines.xhtml

For peer review only

ACKNOWLEDGEMENTS

Sue Whiffin and Jenny Lowe for administrative support. Morwenna Rodgers for support developing the protocol and search strategy. Kirstin Liabo for support developing the protocol and facilitating involvement of patients and members of the public with the review. The PenARC Patient Engagement Group for their insight throughout the review process. Dr Phil Evans for his support developing the patient care pathways and feedback on the draft evidence and gap map. Melissa Bond from the EPPI centre for continued support in developing the evidence and gap map.

CONTRIBUTION OF AUTHORS

Liz Shaw: Lead author. Led scoping process and contributed to the development of the protocol and conduct of screening, data extraction, quality appraisal. Led development of the evidence-and-gap map, patient and public involvement and write up of paper. Supported liaison with government and clinical stakeholders.

Simon Briscoe: Information specialist and reviewer. Supported scoping and led to the development of the search strategy to inform the protocol. Contributed to screening, data extraction and quality appraisal and was responsible for data management throughout the project. Developed PRISMA diagram and contributed to all stakeholder involvement and write up of paper.

Michael Nunns: Reviewer. Supported development of the protocol and contributed to screening, data extraction, quality appraisal and write up of paper.

Hassanat Lawal: Reviewer. Supported screening, data extraction and quality appraisal and reviewed final paper.

G.J. Melendez-Torres: Supported conceptualisation of the project and development of the protocol. Supported development of the evidence and gap map and government stakeholder involvement. Read and reviewed final paper.

Malcolm Turner: Patient and Public Involvement representative and lead. Supported development of the protocol and provided feedback on main findings. Read and reviewed final paper.

Ruth Garside: Supported conceptualisation of the project and development of the protocol. Supported development of the evidence and gap map and government stakeholder involvement. Read and reviewed final paper.

Jo Thompson-Coon: Supported conceptualisation of the project and development of the protocol. Supported development of the evidence and gap map and led government stakeholder involvement. Read and reviewed final paper. Guarantor of work.

For peer review only

Conflicts of interest: None to declare.

Funding: This paper represents part of a larger piece of work commissioned by the National Institute of Health Research Policy Research Programme by the Exeter PRP Evidence Review Facility (NIHR200695) following a request to the Department of Health and Social Care (DHSC) Research and Development Committee by NHS England and Improvement (NHSE-I).

This paper is independent research funded by the National Institute for Health and Care Research Policy Research Programme (NIHR200605) and supported by the National Institute for Health and Care Research Applied Research Collaboration South West Peninsula. The views expressed in this publication are those of the author(s) and not necessarily those of the National Institute for Health and Care Research, the Department of Health and Social Care or NHS England and Improvement.
Data sharing: For access to documentation supporting this review, please contact L. Shaw.

Ethics approval: This review is secondary research and did not require ethical approval.

Patient consent statement: This review is secondary research and did not require collection of patient data.

For peer review only

REFERENCES

1 WHO 2021 https://www.who.int/news-room/fact-sheets/detail/cardiovascular-diseases-(cvds) Accessed 15.9.21

2 Ekinci G. Economic Impacts of Cardiovascular Diseases: An Econometric Evaluation in Turkey. Iran J Public Health. 2023 Jan;52(1):118-127. doi: 10.18502/ijph.v52i1.11673. PMID: 36824237; PMCID: PMC9941436

3 NHSE 2021 https://www.england.nhs.uk/ourwork/clinical-policy/cvd/ Accessed 15.9.21

4 National Institute for Health and Care Excellence. Cardiovascular disease: risk assessment and reduction, including lipid modification [CG181]. Last updated: 27 September 2016: NICE; 2014.

5 Marcus ME, Manne-Goehler J, Theilmann M, Farzadfar F, Moghaddam SS, Keykhaei M, Hajebi A, Tschida S, Lemp JM, Aryal KK, Dunn M, Houehanou C, Bahendeka S, Rohloff P, Atun R, Bärnighausen TW, Geldsetzer P, Ramirez-Zea M, Chopra V, Heisler M, Davies JI, Huffman MD, Vollmer S, Flood D. Use of statins for the prevention of cardiovascular disease in 41 low-income and middle-income countries: a cross-sectional study of nationally representative, individual-level data. Lancet Glob Health. 2022 Mar;10(3):e369-e379. doi: 10.1016/S2214-109X(21)00551-9.

6 Kjeldsen SE. Hypertension and cardiovascular risk: General aspects. Pharmacol Res 2018;129:95-9. https://doi.org/10.1016/j.phrs.2017.11.003

7 Gupta A, Mackay J, Whitehouse A, Godec T, Collier T, Pocock S, et al. Long-term mortality after blood pressure-lowering and lipid-lowering treatment in patients with hypertension in the Anglo-Scandinavian Cardiac Outcomes Trial (ASCOT) Legacy study: 16-year follow-up results of a randomised factorial trial. The Lancet 2018;392:1127-37. https://doi.org/https://doi.org/10.1016/S0140-6736(18)31776-8

8 NCD Risk Factor Collaboration (NCD-RisC). Worldwide trends in hypertension prevalence and progress in treatment and control from 1990 to 2019: a pooled analysis of 1201 population-representative studies with 104 million participants. Lancet. 2021 Sep 11;398(10304):957-980. doi: 10.1016/S0140-6736(21)01330-1. Epub 2021 Aug 24. Erratum in: Lancet. 2022 Feb 5;399(10324):520. PMID: 34450083; PMCID: PMC8446938

9 NCD Risk Factor Collaboration. Long-term and recent trends in hypertension awareness, treatment, and control in 12 high-income countries: an analysis of 123 nationally representative surveys. Lancet 2019;394:639-51. https://doi.org/10.1016/s0140-6736(19)31145-6

10 Suissa S, Brophy J, Azoulay L, Schuster T. Trends in Prescription of Antihypertensive Medications in UK Primary Care Practices, 1988-2018. Clinical Practice Research Datalink; 2019. URL: https://cprd.com/protocol/trends-prescription-antihypertensive-medications-uk-primary-care-practices-1988-2018 (accessed 1 March, 2021).

11 Gupta P, Patel P, Štrauch B, Lai FY, Akbarov A, Marešová V, et al. Risk Factors for Nonadherence to Antihypertensive Treatment. Hypertension 2017;69:1113-20. https://doi.org/10.1161/hypertensionaha.116.08729

12 Reference full report. Withheld for peer review.

For peer review only - http://bmjopen.bmj.com/site/about/guidelines.xhtml

13 Centre for Reviews and Dissemination. Systematic Reviews: CRD's guidance for undertaking reviews in health care: University of York; 2008.

14 White H, Albers B, Gaarder M, Kornør H, Littell J, Marshall Z, et al. Guidance for producing a Campbell evidence and gap map. Campbell Systematic Reviews 2020;16:e1125. https://doi.org/10.1002/cl2.1125

15 Review protocol. Reference withheld for peer review

16 Moher D, Liberati A, Tetzlaff J, Altman DG. Preferred reporting items for systematic reviews and meta-analyses: the PRISMA statement. BMJ 2009;339:b2535. https://doi.org/10.1136/bmj.b2535

17 Collaboration for Environmental Evidence. The Collaboration for Environmental Evidence Synthesis Appraisal Tool (CEESAT). Version 2.1. (Last updated 27 October 2020). CEE; 2018. URL: https://environmentalevidence.shinyapps.io/CEEDER/ (accessed 13 Jan, 2021).

18 Shea BJ, Reeves BC, Wells G, Thuku M, Hamel C, Moran J, et al. AMSTAR 2: a critical appraisal tool for systematic reviews that include randomised or non-randomised studies of healthcare interventions, or both. BMJ 2017;358:j4008. https://doi.org/10.1136/bmj.j4008

19 Tong A, Flemming K, McInnes E, Oliver S, Craig J. Enhancing transparency in reporting the synthesis of qualitative research: ENTREQ. BMC Med Res Methodol 2012;12:181. https://doi.org/10.1186/1471-2288-12-181

20 Digital Solution Foundry, EPPI-Centre. EPPI Mapper, Version 1.2.5: EPPI-Centre, UCL Social Research Institute, University College London; 2020.

21 National Institute for Health and Care Excellence. Hypertension in adults: diagnosis and management NICE guideline [NG136] London: NICE; 2019.

22 National Institute for Health and Care Excellence. Medicines optimisation: the safe and effective use of medicines to enable the best possible outcomes London: NICE; 2015.

23 National Institute for Health and Care Excellence. Shared decision-making about medicines. NICE Pathways; 2021. URL: https://pathways.nice.org.uk/pathways/medicines-optimisation#path=view%3A/pathways/medicines-optimisation/shared-decision-making-about-medicines.xml&content=view-index (accessed 3 March, 2021).

24 Pound P, Britten N, Morgan M, Yardley L, Pope C, Daker-White G, et al. Resisting medicines: a synthesis of qualitative studies of medicine taking. Soc Sci Med 2005;61:133-55. https://doi.org/10.1016/j.socscimed.2004.11.063

25 Deichmann RE, Morledge MD, Ulep R, Shaffer JP, Davies P, van Driel ML. A Metaanalysis of Interventions to Improve Adherence to Lipid-Lowering Medication. Ochsner J 2016;16:230-7.

26 Hukins D, Macleod U, Boland JW. Identifying potentially inappropriate prescribing in older people with dementia: a systematic review. Eur J Clin Pharmacol 2019;75:467-81. https://doi.org/10.1007/s00228-018-02612-x

27 Kinnear FJ, Wainwright E, Perry R, Lithander FE, Bayly G, Huntley A, et al. Enablers and barriers to treatment adherence in heterozygous familial hypercholesterolaemia: a qualitative evidence synthesis. BMJ Open 2019;9:e030290. https://doi.org/10.1136/bmjopen-2019-030290

For peer review only - http://bmjopen.bmj.com/site/about/guidelines.xhtml

28 Legare F, Ratte S, Stacey D, Kryworuchko J, Gravel K, Graham ID, et al. Interventions for improving the adoption of shared decision making by healthcare professionals. Cochrane Database Syst Rev 2010; 10.1002/14651858.CD006732.pub2:CD006732. https://doi.org/10.1002/14651858.CD006732.pub2

29 Nguyen T, Nguyen HQ, Widyakusuma NN, Nguyen TH, Pham TT, Taxis K. Enhancing prescribing of guideline-recommended medications for ischaemic heart diseases: a systematic review and meta-analysis of interventions targeted at healthcare professionals. BMJ Open 2018;8:e018271. https://doi.org/10.1136/bmjopen-2017-018271

30 Rash JA, Campbell DJ, Tonelli M, Campbell TS. A systematic review of interventions to improve adherence to statin medication: What do we know about what works? Prev Med 2016;90:155-69. https://doi.org/10.1016/j.ypmed.2016.07.006

31 Rashid MA, Edwards D, Walter FM, Mant J. Medication taking in coronary artery disease: a systematic review and qualitative synthesis. Ann Fam Med 2014;12:224-32. https://doi.org/10.1370/afm.1620

32 van Driel ML, Morledge MD, Ulep R, Shaffer JP, Davies P, Deichmann R. Interventions to improve adherence to lipid-lowering medication. Cochrane Database of Systematic Reviews 2016; 10.1002/14651858.CD004371.pub4. https://doi.org/10.1002/14651858.CD004371.pub4

33 Bond, Z.; Scanlon, T.; Judah, G. Systematic Review of RCTs Assessing the Effectiveness of mHealth Interventions to Improve Statin Medication Adherence: Using the Behaviour-Change Technique Taxonomy to Identify the Techniques That Improve Adherence. Healthcare 2021, 9, 1282. https://doi.org/ 10.3390/healthcare9101282

34 Reston JT, Buelt A, Donahue MP, Neubauer B, Vagichev E, McShea K. Interventions to Improve Statin Tolerance and Adherence in Patients at Risk for Cardiovascular Disease : A Systematic Review for the 2020 U.S. Department of Veterans Affairs and U.S. Department of Defense Guidelines for Management of Dyslipidemia. Ann Intern Med. 2020 Nov 17;173(10):806-812. doi: 10.7326/M20-4680

35 Bochkareva EV, Butina EK, Kim IV, Kontsevaya AV, Drapkina OM, Leon D, et al. Adherence to antihypertensive medication in Russia: a scoping review of studies on levels, determinants and intervention strategies published between 2000 and 2017. Arch Public Health 2019;77:43. https://doi.org/10.1186/s13690-019-0366-9

36 Chambergo-Michilot D, Runzer-Colmenares FM, Segura-Saldaña PA. Discontinuation of Antihypertensive Drug Use Compared to Continuation in COVID-19 Patients: A Systematic Review with Meta-analysis and Trial Sequential Analysis. High Blood Press Cardiovasc Prev. 2023 May;30(3):265-279. doi: 10.1007/s40292-023-00579-0.

37 Cheema E, Sutcliffe P, Singer DR. The impact of interventions by pharmacists in community pharmacies on control of hypertension: a systematic review and meta-analysis of randomized controlled trials. Br J Clin Pharmacol 2014;78:1238-47. https://doi.org/10.1111/bcp.12452

38 Chhina HK, Bhole VM, Goldsmith C, Hall W, Kaczorowski J, Lacaille D. Effectiveness of academic detailing to optimize medication prescribing behaviour of family physicians. J Pharm Pharm Sci 2013;16:511-29. https://doi.org/10.18433/j3kk6c

For peer review only - http://bmjopen.bmj.com/site/about/guidelines.xhtml

39 Conn VS, Ruppar TM, Chase JA, Enriquez M, Cooper PS. Interventions to Improve Medication Adherence in Hypertensive Patients: Systematic Review and Meta-analysis. Curr Hypertens Rep 2015;17:94. https://doi.org/10.1007/s11906-015-0606-5

40 De Simoni A, Hardeman W, Mant J, Farmer AJ, Kinmonth AL. Trials to improve blood pressure through adherence to antihypertensives in stroke/TIA: systematic review and meta-analysis. J Am Heart Assoc 2013;2:e000251. https://doi.org/10.1161/JAHA.113.000251

41 Demonceau J, Ruppar T, Kristanto P, Hughes DA, Fargher E, Kardas P, et al. Identification and assessment of adherence-enhancing interventions in studies assessing medication adherence through electronically compiled drug dosing histories: a systematic literature review and meta-analysis. Drugs 2013;73:545-62. https://doi.org/10.1007/s40265-013-0041-3

42 Dhar L, Dantas J, Ali M. A Systematic Review of Factors Influencing Medication Adherence to Hypertension Treatment in Developing Countries. Open Journal of Epidemiology 2017;07:211-50. https://doi.org/10.4236/ojepi.2017.73018

43 Du LP, Cheng ZW, Zhang YX, Li Y, Mei D. The impact of fixed-dose combination versus free-equivalent combination therapies on adherence for hypertension: a meta-analysis. J Clin Hypertens (Greenwich) 2018;20:902-7. https://doi.org/10.1111/jch.13272

44 Fletcher BR, Hartmann-Boyce J, Hinton L, McManus RJ. The Effect of Self-Monitoring of Blood Pressure on Medication Adherence and Lifestyle Factors: A Systematic Review and Meta-Analysis. Am J Hypertens 2015;28:1209-21. https://doi.org/10.1093/ajh/hpv008

45 Gupta AK, Arshad S, Poulter NR. Compliance, safety, and effectiveness of fixed-dose combinations of antihypertensive agents: a meta-analysis. Hypertension 2010;55:399-407. https://doi.org/10.1161/HYPERTENSIONAHA.109.139816

46 Guzman-Tordecilla DN, Bernal Garcia A, Rodriguez I. Interventions to increase the pharmacological adherence on arterial hypertension in Latin America: a systematic review. Int J Public Health 2020;65:55-64. https://doi.org/10.1007/s00038-019-01317-x

47 Hart LA, Phelan EA, Yi JY, Marcum ZA, Gray SL. Use of Fall Risk-Increasing Drugs Around a Fall-Related Injury in Older Adults: A Systematic Review. J Am Geriatr Soc 2020;68:1334-43. https://doi.org/10.1111/jgs.16369

48 Hong J, Tiu YC, Leung PYB, Wong MF, Ng WY, Cheung D, Mok HY, Lam WY, Li KY, Wong CK. Interventions that improve adherence to antihypertensive medications in coronary heart disease patients: a systematic review. Postgrad Med J. 2022 Mar;98(1157):219-227. doi: 10.1136/postgradmedj-2020-139116

49 Kawalec P, Holko P, Gawin M, Pilc A. Effectiveness of fixed-dose combination therapy in hypertension: systematic review and meta-analysis. Arch Med Sci 2018;14:1125-36. https://doi.org/10.5114/aoms.2018.77561

50 Khatib R, Schwalm JD, Yusuf S, Haynes RB, McKee M, Khan M, et al. Patient and healthcare provider barriers to hypertension awareness, treatment and follow up: a systematic review and meta-analysis of qualitative and quantitative studies. PLoS One 2014;9:e84238. https://doi.org/10.1371/journal.pone.0084238

For peer review only - http://bmjopen.bmj.com/site/about/guidelines.xhtml

51 Kronish IM, Woodward M, Sergie Z, Ogedegbe G, Falzon L, Mann DM. Meta-analysis: impact of drug class on adherence to antihypertensives. Circulation 2011;123:1611-21. https://doi.org/10.1161/CIRCULATIONAHA.110.983874

52 Loganathan M, Singh S, Franklin BD, Bottle A, Majeed A. Interventions to optimise prescribing in care homes: systematic review. Age Ageing 2011;40:150-62. https://doi.org/10.1093/ageing/afq161

53 Maimaris W, Paty J, Perel P, Legido-Quigley H, Balabanova D, Nieuwlaat R, et al. The influence of health systems on hypertension awareness, treatment, and control: a systematic literature review. PLoS Med 2013;10:e1001490. https://doi.org/10.1371/journal.pmed.1001490

54 Mallat SG, Tanios BY, Itani HS, Lotfi T, Akl EA. Free versus Fixed Combination Antihypertensive Therapy for Essential Arterial Hypertension: A Systematic Review and Meta-Analysis. PLoS One 2016;11:e0161285. https://doi.org/10.1371/journal.pone.0161285

55 Marshall IJ, Wolfe CD, McKevitt C. Lay perspectives on hypertension and drug adherence: systematic review of qualitative research. BMJ 2012;345:e3953. https://doi.org/10.1136/bmj.e3953

56 Milosavljevic A, Aspden T, Harrison J. Community pharmacist-led interventions and their impact on patients' medication adherence and other health outcomes: a systematic review. Int J Pharm Pract 2018;26:387-97. https://doi.org/10.1111/ijpp.12462

57 Morrissey EC, Durand H, Nieuwlaat R, Navarro T, Haynes RB, Walsh JC, et al. Effectiveness and content analysis of interventions to enhance medication adherence and blood pressure control in hypertension: A systematic review and meta-analysis. Psychol Health 2017;32:1195-232. https://doi.org/10.1080/08870446.2016.1273356

58 Oori MJ, Mohammadi F, Norouzi K, Fallahi-Khoshknab M, Ebadi A. Conceptual Model of Medication Adherence in Older Adults with High Blood Pressure-An Integrative Review of the Literature. Curr Hypertens Rev 2019;15:85-92. https://doi.org/10.2174/1573402114666181022152313

59 Parati G, Kjeldsen S, Coca A, Cushman WC, Wang J. Adherence to Single-Pill Versus Free-Equivalent Combination Therapy in Hypertension: A Systematic Review and Meta-Analysis. Hypertension. 2021 Feb;77(2):692-705. doi: 10.1161/HYPERTENSIONAHA.120.15781

60 Rashid MA, Llanwarne N, Heyns N, Walter F, Mant J. What are the implications for practice that arise from studies of medication taking? A systematic review of qualitative research. PLoS One 2018;13:e0195076. https://doi.org/10.1371/journal.pone.0195076

61 Reeve E, To J, Hendrix I, Shakib S, Roberts MS, Wiese MD. Patient barriers to and enablers of deprescribing: a systematic review. Drugs Aging 2013;30:793-807. https://doi.org/10.1007/s40266-013-0106-8

62 Reeves L, Robinson K, McClelland T, Adedoyin CA, Broeseker A, Adunlin G. Pharmacist Interventions in the Management of Blood Pressure Control and Adherence to Antihypertensive Medications: A Systematic Review of Randomized Controlled Trials. J Pharm Pract 2020; 10.1177/0897190020903573:897190020903573. https://doi.org/10.1177/0897190020903573

For peer review only - http://bmjopen.bmj.com/site/about/guidelines.xhtml

63 Sanders GD, Coeytaux R, Dolor RJ, Hasselblad V, Patel UD, Powers B, et al. AHRQ Comparative Effectiveness Reviews. In: Angiotensin-Converting Enzyme Inhibitors (ACEIs), Angiotensin II Receptor Antagonists (ARBs), and Direct Renin Inhibitors for Treating Essential Hypertension: An UpdateRockville (MD): Agency for Healthcare Research and Quality (US); 2011.

64 Shanbhag D, Graham ID, Harlos K, Haynes RB, Gabizon I, Connolly SJ, et al. Effectiveness of implementation interventions in improving physician adherence to guideline recommendations in heart failure: a systematic review. BMJ Open 2018;8:e017765. https://doi.org/10.1136/bmjopen-2017-017765

65 Stacey D, Legare F, Lewis K, Barry MJ, Bennett CL, Eden KB, et al. Decision aids for people facing health treatment or screening decisions. Cochrane Database Syst Rev 2017;4:CD001431. https://doi.org/10.1002/14651858.CD001431.pub5

66 Tsioufis K, Kreutz R, Sykara G, van Vugt J, Hassan T. Impact of single-pill combination therapy on adherence, blood pressure control, and clinical outcomes: a rapid evidence assessment of recent literature. Journal of Hypertension 2020;38:1016-28. https://doi.org/10.1097/Hjh.0000000000002381

67 Van Truong P, Wulan Apriliyasari R, Lin MY, Chiu HY, Tsai PS. Effects of self-management programs on blood pressure, self-efficacy, medication adherence and body mass index in older adults with hypertension: Meta-analysis of randomized controlled trials. Int J Nurs Pract. 2021 Apr;27(2):e12920. doi: 10.1111/ijn.12920

68 Uhlig K, Patel K, Ip S, Kitsios GD, Balk EM. Self-measured blood pressure monitoring in the management of hypertension: a systematic review and meta-analysis. Ann Intern Med 2013;159:185-94. https://doi.org/10.7326/0003-4819-159-3-201308060-00008

69 Xiong S, Berkhouse H, Schooler M, Pu W, Sun A, Gong E, et al. Effectiveness of mHealth Interventions in Improving Medication Adherence Among People with Hypertension: a Systematic Review. Curr Hypertens Rep 2018;20:86. https://doi.org/10.1007/s11906-018-0886-7

70 Xu R, Xie X, Li S, Chen X, Wang S, Hu C, et al. Interventions to improve medication adherence among Chinese patients with hypertension: a systematic review and meta-analysis of randomized controlled trails. Int J Pharm Pract 2018;26:291-301. https://doi.org/10.1111/ijpp.12452

71 Yuan C, Ding Y, Zhou K, Huang Y, Xi X. Clinical outcomes of community pharmacy services: A systematic review and meta-analysis. Health Soc Care Community 2019;27:e567-e87. https://doi.org/10.1111/hsc.12794

72 Zheng J, Mednick T, Heidenreich PA, Sandhu AT. Pharmacist- and Nurse-Led Medical Optimization in Heart Failure: A Systematic Review and Meta-Analysis. J Card Fail. 2023 Jul;29(7):1000-1013. doi: 10.1016/j.cardfail.2023.03.012.

73 Adler AJ, Martin N, Mariani J, Tajer CD, Owolabi OO, Free C, et al. Mobile phone text messaging to improve medication adherence in secondary prevention of cardiovascular disease. Cochrane Database Syst Rev 2017;4:CD011851. https://doi.org/10.1002/14651858.CD011851.pub2

For peer review only

For peer review only - http://bmjopen.bmj.com/site/about/guidelines.xhtml

74 Al AlShaikh S, Quinn T, Dunn W, Walters M, Dawson J. Multimodal Interventions to Enhance Adherence to Secondary Preventive Medication after Stroke: A Systematic Review and Meta-Analyses. Cardiovasc Ther 2016;34:85-93. https://doi.org/10.1111/1755-5922.12176

75 Baumgartner A, Drame K, Geutjens S, Airaksinen M. Does the Polypill Improve Patient Adherence Compared to Its Individual Formulations? A Systematic Review. Pharmaceutics 2020;12:190. https://doi.org/10.3390/pharmaceutics12020190

76 Checchi KD, Huybrechts KF, Avorn J, Kesselheim AS. Electronic medication packaging devices and medication adherence: a systematic review. JAMA 2014;312:1237-47. https://doi.org/10.1001/jama.2014.10059

77 Conn VS, Ruppar TM, Chan KC, Dunbar-Jacob J, Pepper GA, De Geest S. Packaging interventions to increase medication adherence: systematic review and meta-analysis. Curr Med Res Opin 2015;31:145-60. https://doi.org/10.1185/03007995.2014.978939

78 Conn VS, Ruppar TM, Enriquez M, Cooper PS, Chan KC. Healthcare provider targeted interventions to improve medication adherence: systematic review and meta-analysis. Int J Clin Pract 2015;69:889-99. https://doi.org/10.1111/ijcp.12632

79 Conn VS, Ruppar TM, Enriquez M, Cooper P. Medication adherence interventions that target subjects with adherence problems: Systematic review and meta-analysis. Res Social Adm Pharm 2016;12:218-46. https://doi.org/10.1016/j.sapharm.2015.06.001

80 Coronado-Vázquez V, Canet-Fajas C, Delgado-Marroquín MT, Magallón-Botaya R, Romero-Martín M, Gómez-Salgado J. Interventions to facilitate shared decision-making using decision aids with patients in Primary Health Care: A systematic review. Medicine (Baltimore). 2020 Aug 7;99(32):e21389. doi: 10.1097/MD.0000000000021389

81 de Cates AN, Farr MR, Wright N, Jarvis MC, Rees K, Ebrahim S, et al. Fixed-dose combination therapy for the prevention of cardiovascular disease. Cochrane Database Syst Rev 2014; 10.1002/14651858.CD009868.pub2:CD009868. https://doi.org/10.1002/14651858.CD009868.pub2

82 Dills H, Shah K, Messinger-Rapport B, Bradford K, Syed Q. Deprescribing Medications for Chronic Diseases Management in Primary Care Settings: A Systematic Review of Randomized Controlled Trials. J Am Med Dir Assoc 2018;19:923-35 e2.

83 Ingersgaard MV, Helms Andersen T, Norgaard O, Grabowski D, Olesen K. Reasons for Nonadherence to Statins - A Systematic Review of Reviews. Patient Prefer Adherence 2020;14:675-91. https://doi.org/10.2147/PPA.S245365

84 Jeffery RA, To MJ, Hayduk-Costa G, Cameron A, Taylor C, Van Zoost C, et al. Interventions to improve adherence to cardiovascular disease guidelines: a systematic review. BMC Fam Pract 2015;16:147. https://doi.org/10.1186/s12875-015-0341-7

85 Kang JE, Han NY, Oh JM, Jin HK, Kim HA, Son IJ, et al. Pharmacist-involved care for patients with heart failure and acute coronary syndrome: a systematic review with qualitative and quantitative meta-analysis. J Clin Pharm Ther 2016;41:145-57. https://doi.org/10.1111/jcpt.12367

86 Kassavou A, Sutton S. Automated telecommunication interventions to promote adherence to cardio-metabolic medications: meta-analysis of effectiveness and meta-regression of

For peer review only

behaviour change techniques. Health Psychol Rev 2018;12:25-42. https://doi.org/10.1080/17437199.2017.1365617

87 King S, Miani C, Exley J, Larkin J, Kirtley A, Payne RA. Impact of issuing longer- versus shorter-duration prescriptions: a systematic review. Br J Gen Pract 2018;68:e286-e92. https://doi.org/10.3399/bjgp18X695501

88 Kornholt J, Bülow C, Sørensen AMS, Pressel E, Petersen TS, Christensen MB. Scoping Review of Randomized Trials With Discontinuation of Medicines in Older Adults. J Am Med Dir Assoc. 2022 Dec;23(12):1926.e11-1926.e35. doi: 10.1016/j.jamda.2022.06.010.

89 Krack G. How to make value-based health insurance designs more effective? A systematic review and meta-analysis. Eur J Health Econ 2019;20:841-56. https://doi.org/10.1007/s10198-019-01046-1

90 Laba TL, Bleasel J, Brien JA, Cass A, Howard K, Peiris D, et al. Strategies to improve adherence to medications for cardiovascular diseases in socioeconomically disadvantaged populations: a systematic review. Int J Cardiol 2013;167:2430-40. https://doi.org/10.1016/j.ijcard.2013.01.049

91 Lawrence M, Pringle J, Kerr S, Booth J, Govan L, Roberts NJ. Multimodal secondary prevention behavioral interventions for TIA and stroke: a systematic review and meta-analysis. PLoS One 2015;10:e0120902. https://doi.org/10.1371/journal.pone.0120902

92 Leslie RS. Adherence to medications to treat diabetes, hypertension, and hypercholesterolemia among Medicare Part D beneficiaries [PhD]. San Diego: University of California; 2016.

93 Mamudu HM, Paul TK, Veeranki SP, Budoff M. The effects of coronary artery calcium screening on behavioral modification, risk perception, and medication adherence among asymptomatic adults: a systematic review. Atherosclerosis 2014;236:338-50. https://doi.org/10.1016/j.atherosclerosis.2014.07.022

94 Mann BS, Barnieh L, Tang K, Campbell DJ, Clement F, Hemmelgarn B, et al. Association between drug insurance cost sharing strategies and outcomes in patients with chronic diseases: a systematic review. PLoS One 2014;9:e89168. https://doi.org/10.1371/journal.pone.0089168

95 Nieuwlaat R, Wilczynski N, Navarro T, Hobson N, Jeffery R, Keepanasseril A, et al. Interventions for enhancing medication adherence. Cochrane Database Syst Rev 2014;2014:CD000011. https://doi.org/10.1002/14651858.CD000011.pub4

96 Nili M, Mohamed R, Kelly KM. A systematic review of interventions using health behavioral theories to improve medication adherence among patients with hypertension. Transl Behav Med. 2020 Oct 12;10(5):1177-1186. doi: 10.1093/tbm/ibaa020. PMID: 33044533

97 Page AT, Clifford RM, Potter K, Schwartz D, Etherton-Beer CD. The feasibility and effect of deprescribing in older adults on mortality and health: a systematic review and meta-analysis. Br J Clin Pharmacol 2016;82:583-623. https://doi.org/10.1111/bcp.12975

98 Palmer MJ, Barnard S, Perel P, Free C. Mobile phone-based interventions for improving adherence to medication prescribed for the primary prevention of cardiovascular disease in

For peer review only

adults. Cochrane Database Syst Rev 2018;6:CD012675. https://doi.org/10.1002/14651858.CD012675.pub2

99 Palmer MJ, Machiyama K, Woodd S, Gubijev A, Barnard S, Russell S, Perel P, Free C. Mobile phone-based interventions for improving adherence to medication prescribed for the primary prevention of cardiovascular disease in adults. Cochrane Database of Systematic Reviews 2021, Issue 3. Art. No.: CD012675. DOI: 10.1002/14651858.CD012675.pub3

100 Pantoja T, Grimshaw JM, Colomer N, Castanon C, Leniz Martelli J. Manually-generated reminders delivered on paper: effects on professional practice and patient outcomes. Cochrane Database Syst Rev 2019;12:CD001174. https://doi.org/10.1002/14651858.CD001174.pub4

101 Park YJ, Martin EG. Medicare Part D's Effects on Drug Utilization and Out-of-Pocket Costs: A Systematic Review. Health Serv Res 2017;52:1685-728. https://doi.org/10.1111/1475-6773.12534

102 Polinski JM, Donohue JM, Kilabuk E, Shrank WH. Medicare Part D's effect on the under- and overuse of medications: a systematic review. J Am Geriatr Soc 2011;59:1922-33. https://doi.org/10.1111/j.1532-5415.2011.03537.x

103 Posadzki P, Mastellos N, Ryan R, Gunn LH, Felix LM, Pappas Y, et al. Automated telephone communication systems for preventive healthcare and management of long-term conditions. Cochrane Database Syst Rev 2016;12:CD009921. https://doi.org/10.1002/14651858.CD009921.pub2

104 Qadi O, Lufumpa N, Adderley N, Bem D, Marshall T, Kokab F. Patients' and health professionals' attitudes and perceptions towards the initiation of preventive drugs for primary prevention of cardiovascular disease: a systematic review of qualitative studies. BJGP Open. 2020 Dec 15;4(5):bjgpopen20X101087. doi: 10.3399/bjgpopen20X101087

105 Vervloet M, Korevaar JC, Leemrijse CJ, Paget J, Zullig LL, van Dijk L. Interventions to Improve Adherence to Cardiovascular Medication: What About Gender Differences? A Systematic Literature Review. Patient Prefer Adherence. 2020 Oct 29;14:2055-2070. doi: 10.2147/PPA.S260562

106 Xie CX, Chen Q, Hincapié CA, Hofstetter L, Maher CG, Machado GC. Effectiveness of clinical dashboards as audit and feedback or clinical decision support tools on medication use and test ordering: a systematic review of randomized controlled trials. J Am Med Inform Assoc. 2022 Sep 12;29(10):1773-1785. doi: 10.1093/jamia/ocac094

For peer review only

For peer review only - http://bmjopen.bmj.com/site/about/guidelines.xhtml

For peer review only

For peer review only - http://bmjopen.bmj.com/site/about/guidelines.xhtml

FIGURES

Figure 1: Evidence and gap map

Figure 2: PRISMA diagram

Figure 3: Focus/aims of systematic review evidence included in the map

For peer review only

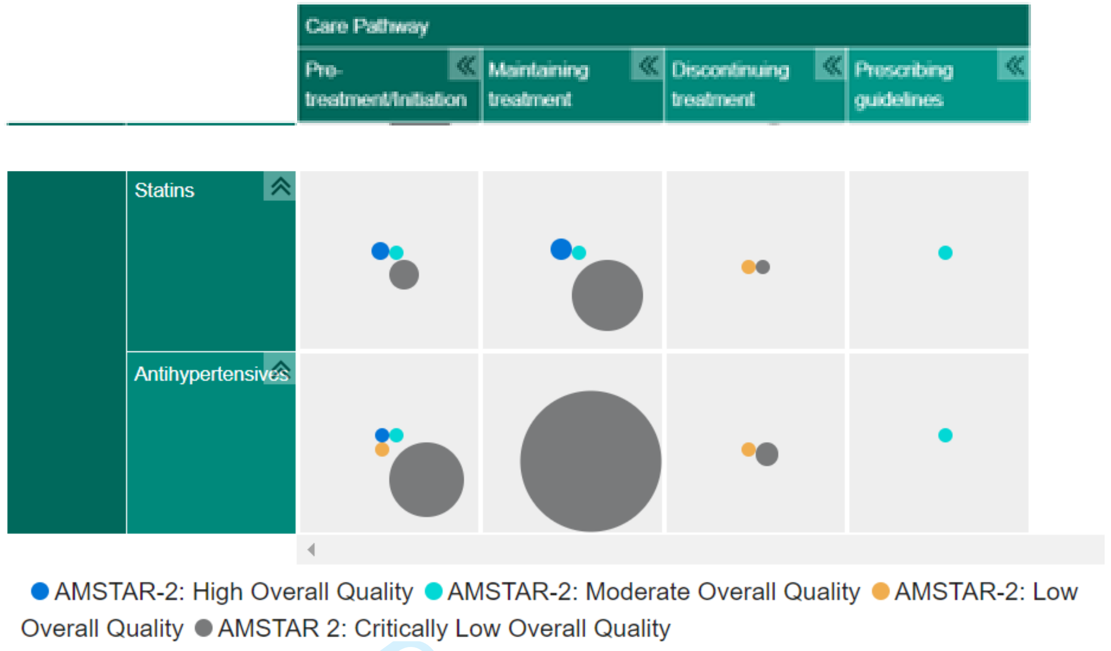

Figure 1: Evidence and gap map

peer review only

PRISMA 2020 flow diagram for new systematic reviews which included searches of databases, registers and other sources

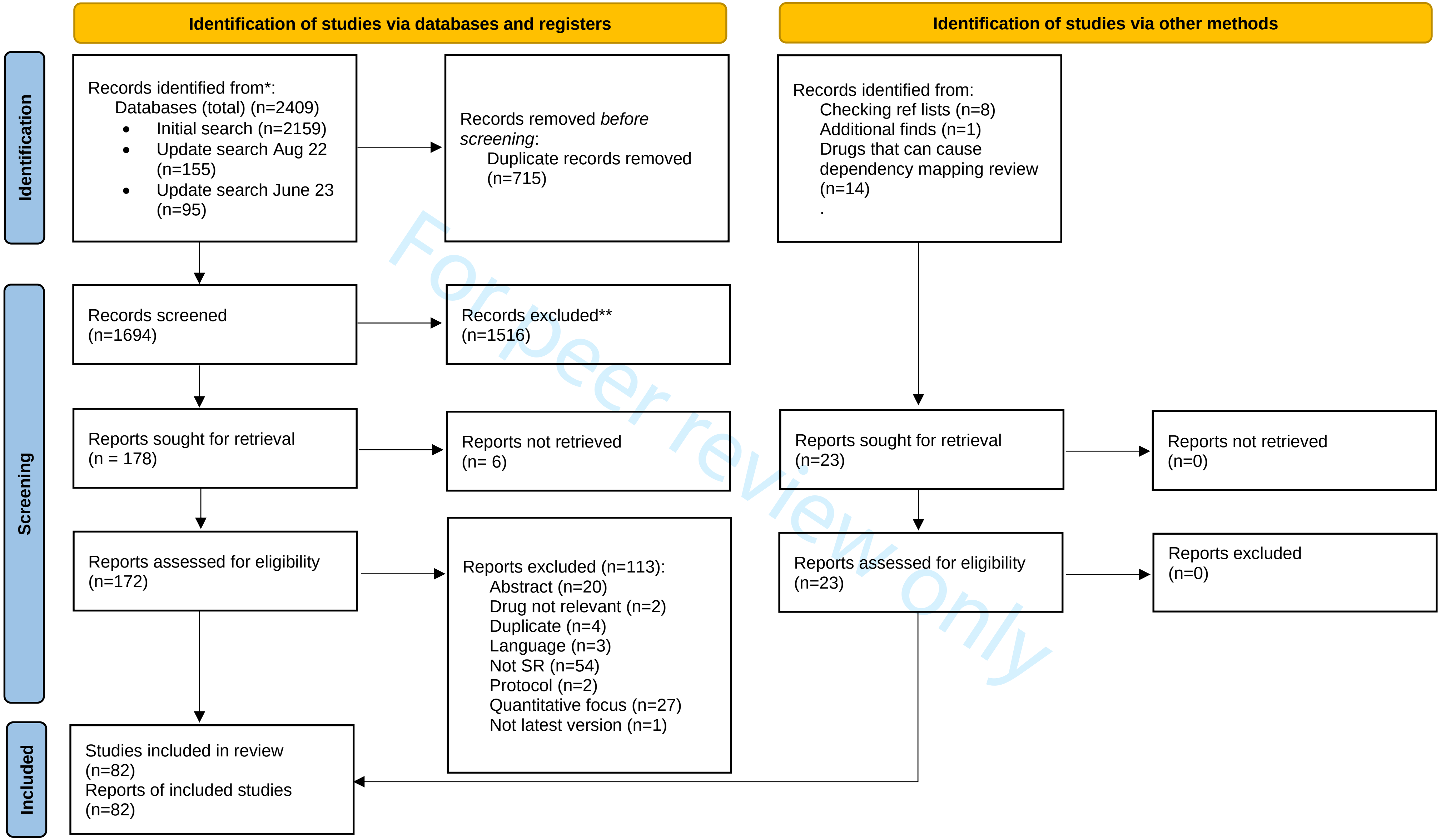

*Consider, if feasible to do so, reporting the number of records identified from each database or register searched (rather than the total number across all databases/registers).
**If automation tools were used, indicate how many records were excluded by a human and how many were excluded by automation tools.

From: Page MJ, McKenzie JE, Bossuyt PM, Boutron I, Hoffmann TC, Mulrow CD, et al. The PRISMA 2020 statement: an updated guideline for reporting systematic reviews. BMJ 2021;372:n71. doi: 10.1136/bmj.n71. For more information, visit: http://www.prisma-statement.org/

For peer review only

PRISMA 2020 flow diagram for new systematic reviews which included searches of databases, registers and other sources

For peer review only

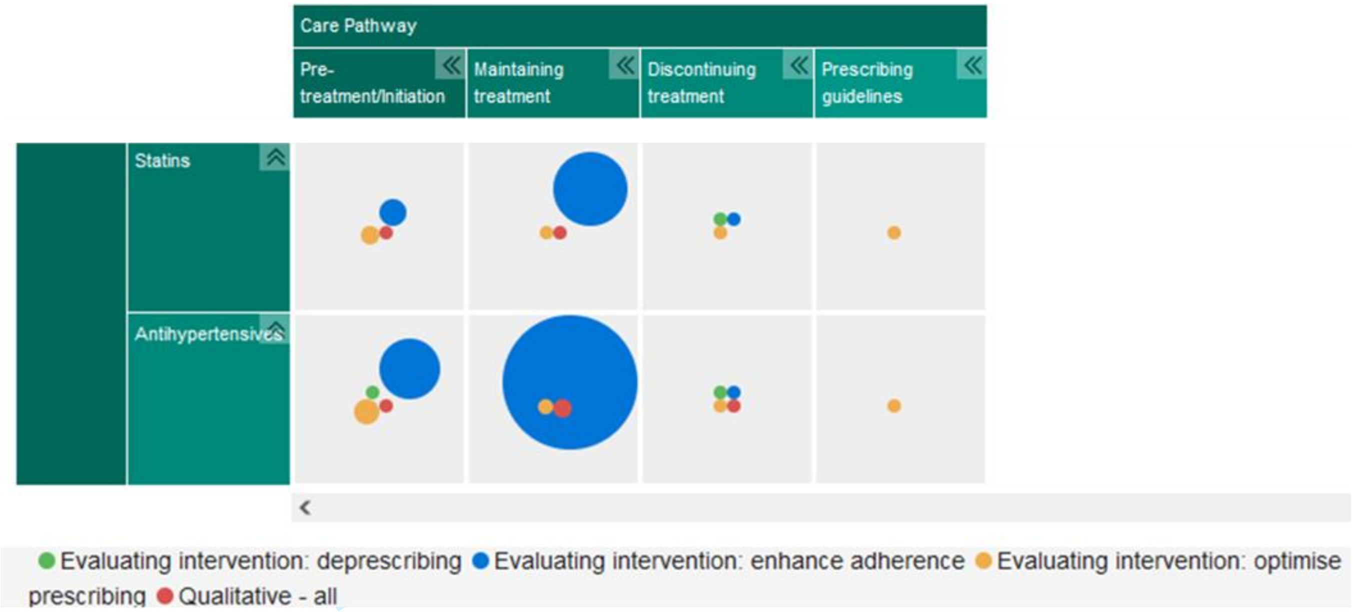

Figure 3: Focus/aims of systematic review evidence included in the map

For peer review only

APPENDIX A: SEARCH STRATEGY

Bibliographic database searches

Drugs that cause dependency

Database: Cochrane Database of Systematic Reviews
Host: Cochrane Library
Issue: Issue 8 of 12, August 2020
Date Searched: 11/8/2020
Searcher: SB
Hits: 111
Strategy:

#1 (((appropriate* or discontinu* or enhance* or inappropriate* or incorrect* or "in correct*" or optim* or safe or suboptim* or "sub optim*" or tapering or withdrawal) NEAR/4 (drug* or medicine* or medication* or prescri*))):ti,ab,kw

#2 MeSH descriptor: [Inappropriate Prescribing] this term only

#3 MeSH descriptor: [Drug Prescriptions] explode all trees

#4 (((drug* or guideline* or guidance or medicine* or medication* or patient* or prescri*) NEAR/4 (adhere* or compliance or concordance))):ti,ab,kw

#5 MeSH descriptor: [Patient Compliance] explode all trees

#6 ((shared or sharing or informed) near/2 (decision* or choice*)):ti,ab,kw

#7 ((decision near/2 (aid* or support*))):ti,ab,kw

#8 MeSH descriptor: [Decision Making] this term only

#9 MeSH descriptor: [Decision Support Techniques] this term only

#10 (((consumer* or patient*) near/3 (involv* or participat*))):ti,ab,kw

#11 MeSH descriptor: [Patient Participation] this term only

#12 ((patient NEXT cent* near/2 (approach* or care or decision* or intervention* or treatment*))):ti,ab,kw

#13 MeSH descriptor: [Patient-Centered Care] this term only

#14 ((behavi* near/2 chang*)):ti,ab,kw

#15 ((restriction near/2 (policy or policies))):ti,ab,kw

#16 {Wilson, #1-#15}

#17 (benzodiazepine*):ti,ab,kw

#18 ((alprazolam or flunitrazepam or chlordiazepoxide or clobazam or clonazepam or diazepam or lorazepam or midazolam or nitrazepam or oxazepam or prazepam or temazepam)):ti,ab,kw

#19 MeSH descriptor: [Benzodiazepines] explode all trees

For peer review only - http://bmjopen.bmj.com/site/about/guidelines.xhtml

#20 ((antidepres* or "anti depres*")):ti,ab,kw

#21 ((serotonin or norepinephrine or noradrenaline or neurotransmitter* or dopamin* or SSRI* or SNRI* or NARI* or SARI* or NDRI* or tricyclic* or tetracyclic*)):ti,ab,kw

#22 MeSH descriptor: [Antidepressive Agents] explode all trees

#23 ((opioid* or opiate*)):ti,ab,kw

#24 ((morphine or hydromorphone or levorphanol or meperidine or methadone or propoxyphene of codeine or pentazocine or hydrocodone or oxycodone or fentanyl or tramadol)):ti,ab,kw

#25 MeSH descriptor: [Analgesics, Opioid] explode all trees

#26 ("z drug*"):ti,ab,kw

#27 ((zopiclone or zolpidem or zaleplon or eszopiclone)):ti,ab,kw

#28 MeSH descriptor: [Hypnotics and Sedatives] explode all trees

#29 ((gabapentin* or mirogabalin or phenibut or pregabalin)):ti,ab,kw

#30 MeSH descriptor: [Gabapentin] this term only

#31 {Ahmed, #17-#30}

#32 #16 AND #31 in Cochrane Reviews [Drugs that cause dependency search results]

#33 (statin*):ti,ab,kw

#34 (("reductase inhibitors" or "HMGCR inhibitors")):ti,ab,kw

#35 MeSH descriptor: [Hydroxymethylglutaryl-CoA Reductase Inhibitors] explode all trees

#36 ((atorvastatin or Lipitor or Torvast)):ti,ab,kw

#37 ((fluvastatin or Lescol)):ti,ab,kw

#38 ((lovastatin or Mevacor or Altocor or Altoprev)):ti,ab,kw

#39 ((pitavastatin or Livalo or Pitava)):ti,ab,kw

#40 ((pravastatin or Pravachol or Selektine or Lipostat)):ti,ab,kw

#41 ((rosuvastatin or Crestor)):ti,ab,kw

#42 ((simvastatin or Zocor or Lipex)):ti,ab,kw

#43 (antihypertensive*):ti,ab,kw

#44 MeSH descriptor: [Antihypertensive Agents] explode all trees

#45 ((ACE NEXT inhibitor* or antagonist or "angiotensin II receptor" or beta NEXT blocker* or calcium NEXT channel NEXT blocker* or thiazide NEXT diuretic*)):ti,ab,kw

#46 ((acebutolol or adrenomedullin or alprenolol or amlodipine or atenolol or bendroflumethiazide or bepridil or betaxolol or bethanidine or bimatoprost or bisoprolol or bosentan or "bretylium tosylate" or brimonidine tartrate or bupranolol or captopril or carteolol or

For peer review only - http://bmjopen.bmj.com/site/about/guidelines.xhtml

carvedilol or celiprolol or chlorisondamine or chlorothiazide or chlorthalidone or cilazapril or clonidine or cromakalim or cyclopenthiazide or debrisoquin or diazoxide or dihydralazine or dihydroalprenolol or diltiazem or doxazosin or enalapril or enalaprilat or eplerenone or epoprostenol or felodipine or fenoldopam or fosinopril or guanabenz or guanethidine or guanfacine or hexamethonium or "hexamethonium compound*" or hydralazine or hydrochlorothiazide or hydroflumethiazide or indapamide or indoramin or irbesartan or isradipine or kallidin or ketanserin or labetalol or latanoprost or lisinopril or losartan or mecamylamine or methyldopa or metipranolol or metolazone or metoprolol or mibefradil or minoxidil or muzolimine or nadolol or nebivolol or nicardipine or nicorandil or nimodipine or nisoldipine or nitrendipine or nitroprusside or olmesartan medoxomil or oxprenolol or pargyline or pempidine or penbutolol or "pentolinium tartrate" or perindopril or phenoxybenzamine or phentolamine or pinacidil or pindolol or piperoxan or polythiazide or prazosin or propranolol or protoveratrines or quinapril or ramipril or reserpine or rilmenidine or telmisartan or teprotide or terlipressin or ticrynafen or timolol or todralazine or tolazoline or torsemide or travoprost or trichlormethiazide or trimethaphan or valsartan or "veratrum alkaloid*" or vincamine or xipamide)):ti,ab,kw

#47 ([103-#46])

#48 (cardiovascular):ti,ab,kw

#49 MeSH descriptor: [Cardiovascular Diseases] explode all trees

#50 (((cardiac or coronary or heart) near/3 (arrest* or attack* or disease* or failure*))):ti,ab,kw

#51 ((heart or myocard* or transient) near/3 (infarc* or ischaemi* or ischemi*)):ti,ab,kw

#52 (angina*):ti,ab,kw

#53 MeSH descriptor: [Angina Pectoris] this term only

#54 (stroke*):ti,ab,kw

#55 MeSH descriptor: [Stroke] explode all trees

#56 {Ford, #48-#55}

#57 #16 and #47 and #56 in Cochrane Reviews [Statins search results]

Notes: Date limited January 2010 – to date of search.

Database: CINAHL
Host: EBSCO
Issue: n/a
Date Searched: 11/8/2020
Searcher: SB
Hits: 372
Strategy

1. TI ((appropriate* or discontinu* or enhance* or inappropriate* or incorrect* or "in correct*" or optim* or safe or suboptim* or "sub optim*" or tapering or withdrawal) N3 (drug* or medicine* or medication* or prescri*)) OR AB ((appropriate* or discontinu* or enhance* or inappropriate* or incorrect* or "in correct*" or optim* or safe or suboptim* or

For peer review only - http://bmjopen.bmj.com/site/about/guidelines.xhtml

"sub optim*" or tapering or withdrawal) N3 (drug* or medicine* or medication* or prescri*))

2. (MH "Inappropriate Prescribing")
3. (MM "Prescriptions, Drug+")
4. TI ((drug* or guideline* or guidance or medicine* or medication* or patient* or prescri*) N3 (adhere* or compliance or concordance)) OR AB ((drug* or guideline* or guidance or medicine* or medication* or patient* or prescri*) N3 (adhere* or compliance or concordance))
5. (MM "Patient Compliance+")
6. TI ((shared or sharing or informed) N1 (decision* or choice*)) OR AB ((shared or sharing or informed) N1 (decision* or choice*))
7. TI (decision N1 (aid* or support*)) OR AB (decision N1 (aid* or support*))
8. (MM "Decision Making")
9. (MM "Decision Support Techniques")
10. TI ((consumer* or patient*) N2 (involv* or participat*)) OR AB ((consumer* or patient*) N2 (involv* or participat*))
11. (MM "Consumer Participation")
12. TI ("patient cent*" N1 (approach* or care or decision* or intervention* or treatment*)) OR AB ("patient cent*" N1 (approach* or care or decision* or intervention* or treatment*))
13. (MH "Patient Centered Care")
14. TI (behavi* N1 chang*) OR AB (behavi* N1 chang*)
15. TI (restriction N1 (policy or policies)) OR AB (restriction N1 (policy or policies))
16. S1 OR S2 OR S3 OR S4 OR S5 OR S6 OR S7 OR S8 OR S9 OR S10 OR S11 OR S12 OR S13 OR S14 OR S15
17. TI benzodiazepine* OR AB benzodiazepine*
18. TI (alprazolam or flunitrazepam or chlordiazepoxide or clobazam or clonazepam or diazepam or lorazepam or midazolam or nitrazepam or oxazepam or prazepam or temazepam) OR AB (alprazolam or flunitrazepam or chlordiazepoxide or clobazam or clonazepam or diazepam or lorazepam or midazolam or nitrazepam or oxazepam or prazepam or temazepam)
19. (MH "Antianxiety Agents, Benzodiazepine+")
20. TI (antidepres* or "anti depres*") OR AB (antidepres* or "anti depres*")
21. TI ((serotonin or norepinephrine or noradrenaline or neurotransmitter* or dopamin* or SSRI* or SNRI* or NARI* or SARI* or NDRI* or tricyclic* or tetracyclic*)) OR AB ((serotonin or norepinephrine or noradrenaline or neurotransmitter* or dopamin* or SSRI* or SNRI* or NARI* or SARI* or NDRI* or tricyclic* or tetracyclic*))

For peer review only - http://bmjopen.bmj.com/site/about/guidelines.xhtml

22. (MH "Antidepressive Agents+")
23. TI ((opioid* or opiate*)) OR AB ((opioid* or opiate*))
24. TI ((morphine or hydromorphone or levorphanol or meperidine or methadone or propoxyphene of codeine or pentazocine or hydrocodone or oxycodone or fentanyl or tramadol)) OR AB ((morphine or hydromorphone or levorphanol or meperidine or methadone or propoxyphene of codeine or pentazocine or hydrocodone or oxycodone or fentanyl or tramadol))
25. (MH "Analgesics, Opioid+")
26. TI "z drug*" OR AB "z drug*"
27. TI ((zopiclone or zolpidem or zaleplon or eszopiclone)) OR AB ((zopiclone or zolpidem or zaleplon or eszopiclone))
28. (MH "Hypnotics and Sedatives")
29. TI ((gabapentin* or mirogabalin or phenibut or pregabalin)) OR AB ((gabapentin* or mirogabalin or phenibut or pregabalin))
30. (MH "Gabapentin")
31. S17 OR S18 OR S19 OR S20 OR S21 OR S22 OR S23 OR S24 OR S25 OR S26 OR S27 OR S28 OR S29 OR S30
32. S16 AND S31
33. TI statin* OR AB statin*
34. TI ("HMG-CoA reductase inhibitor*" or "3-hydroxy-3-methyl-glutaryl-CoA reductase inhibitor*" or "3-hydroxy-3-methylglutaryl coenzyme A reductase inhibitor*" or "HMGCR inhibitor*" or "Hydroxymethylglutaryl-CoA Reductase Inhibitor*") OR AB ("HMG-CoA reductase inhibitor*" or "3-hydroxy-3-methyl-glutaryl-CoA reductase inhibitor*" or "3-hydroxy-3-methylglutaryl coenzyme A reductase inhibitor*" or "HMGCR inhibitor*" or "Hydroxymethylglutaryl-CoA Reductase Inhibitor*")
35. (MH "Statins+")
36. TI (atorvastatin or Lipitor or Torvast) OR AB (atorvastatin or Lipitor or Torvast)
37. TI (fluvastatin or Lescol) OR AB (fluvastatin or Lescol)
38. TI (lovastatin or Mevacor or Altocor or Altoprev) OR AB (lovastatin or Mevacor or Altocor or Altoprev)
39. TI (pitavastatin or Livalo or Pitava) OR AB (pitavastatin or Livalo or Pitava)
40. TI (pravastatin or Pravachol or Selektine or Lipostat) OR AB (pravastatin or Pravachol or Selektine or Lipostat)
41. TI (rosuvastatin or Crestor) OR AB (rosuvastatin or Crestor)
42. TI (simvastatin or Zocor or Lipex) OR AB (simvastatin or Zocor or Lipex)
43. TI antihypertensive* OR AB antihypertensive*

For peer review only

For peer review only - http://bmjopen.bmj.com/site/about/guidelines.xhtml

44. (MH "Antihypertensive Agents+")

45. TI ("ACE inhibitor*" or antagonist or "angiotensin II receptor" or "beta blocker*" or "calcium channel blocker*" or "thiazide diuretic*") OR AB ("ACE inhibitor*" or antagonist or "angiotensin II receptor" or "beta blocker*" or "calcium channel blocker*" or "thiazide diuretic*")

46. TI (acebutolol or adrenomedullin or alprenolol or amlodipine or atenolol or bendroflumethiazide or bepridil or betaxolol or bethanidine or bimatoprost or bisoprolol or bosentan or "bretylium tosylate" or brimonidine tartrate or bupranolol or captopril or carteolol or carvedilol or celiprolol or chlorisondamine or chlorothiazide or chlorthalidone or cilazapril or clonidine or cromakalim or cyclopenthiazide or debrisoquin or diazoxide or dihydralazine or dihydroalprenolol or diltiazem or doxazosin or enalapril or enalaprilat or eplerenone or epoprostenol or felodipine or fenoldopam or fosinopril or guanabenz or guanethidine or guanfacine or hexamethonium or "hexamethonium compound*" or hydralazine or hydrochlorothiazide or hydroflumethiazide or indapamide or indoramin or irbesartan or isradipine or kallidin or ketanserin or labetalol or latanoprost or lisinopril or losartan or mecamylamine or methyldopa or metipranolol or metolazone or metoprolol or mibefradil or minoxidil or muzolimine or nadolol or nebivolol or nicardipine or nicorandil or nimodipine or nisoldipine or nitrendipine or nitroprusside or olmesartan medoxomil or oxprenolol or pargyline or pempidine or penbutolol or "pentolinium tartrate" or perindopril or phenoxybenzamine or phentolamine or pinacidil or pindolol or piperoxan or polythiazide or prazosin or propranolol or protoveratrines or quinapril or ramipril or reserpine or rilmenidine or telmisartan or teprotide or terlipressin or ticrynafen or timolol or todralazine or tolazoline or torsemide or travoprost or trichlormethiazide or trimethaphan or valsartan or "veratrum alkaloid*" or vincamine or xipamide) OR AB (acebutolol or adrenomedullin or alprenolol or amlodipine or atenolol or bendroflumethiazide or bepridil or betaxolol or bethanidine or bimatoprost or bisoprolol or bosentan or "bretylium tosylate" or brimonidine tartrate or bupranolol or captopril or carteolol or carvedilol or celiprolol or chlorisondamine or chlorothiazide or chlorthalidone or cilazapril or clonidine or cromakalim or cyclopenthiazide or debrisoquin or diazoxide or dihydralazine or dihydroalprenolol or diltiazem or doxazosin or enalapril or enalaprilat or eplerenone or epoprostenol or felodipine or fenoldopam or fosinopril or guanabenz or guanethidine or guanfacine or hexamethonium or "hexamethonium compound*" or hydralazine or hydrochlorothiazide or hydroflumethiazide or indapamide or indoramin or irbesartan or isradipine or kallidin or ketanserin or labetalol or latanoprost or lisinopril or losartan or mecamylamine or methyldopa or metipranolol or metolazone or metoprolol or mibefradil or minoxidil or muzolimine or nadolol or nebivolol or nicardipine or nicorandil or nimodipine or nisoldipine or nitrendipine or nitroprusside or olmesartan medoxomil or oxprenolol or pargyline or pempidine or penbutolol or "pentolinium tartrate" or perindopril or phenoxybenzamine or phentolamine or pinacidil or pindolol or piperoxan or polythiazide or prazosin or propranolol or protoveratrines or quinapril or ramipril or reserpine or rilmenidine or telmisartan or teprotide or terlipressin or ticrynafen or timolol or todralazine or tolazoline or torsemide or travoprost or trichlormethiazide or trimethaphan or valsartan or "veratrum alkaloid*" or vincamine or xipamide)

47. S33 OR S34 OR S35 OR S36 OR S37 OR S38 OR S39 OR S40 OR S41 OR S42 OR S43 OR S44 OR S45 OR S46

48. TI cardiovascular OR AB cardiovascular

For peer review only - http://bmjopen.bmj.com/site/about/guidelines.xhtml

49. (MH "Cardiovascular Diseases+")
50. TI ((cardiac or coronary or heart) N2 (arrest* or attack* or disease* or failure*)) OR AB ((cardiac or coronary or heart) N2 (arrest* or attack* or disease* or failure*))
51. TI ((heart or myocard* or transient) N2 (infarc* or ischaemi* or ischemi*)) OR AB ((heart or myocard* or transient) N2 (infarc* or ischaemi* or ischemi*))
52. TI angina* OR AB angina*
53. (MH "Angina Pectoris")
54. TI stroke* OR AB stroke*
55. (MH "Stroke+")
56. S48 OR S49 OR S50 OR S51 OR S52 OR S53 OR S54 OR S55
57. S16 AND S47 AND S56
58. TI ((effectiveness or implementation or literature or map or mapping or qualitative or rapid or realist or systematic or scoping or "state of the art" or umbrella) N1 (assessment* or overview* or review* or synthes*)) OR AB ((effectiveness or implementation or literature or map or mapping or qualitative or rapid or realist or systematic or scoping or "state of the art" or umbrella) N1 (assessment* or overview* or review* or synthes*))
59. TI ("meta analy*" or metaanaly* or metasynthe* or "meta synthe*") OR AB ("meta analy*" or metaanaly* or metasynthe* or "meta synthe*")
60. TI ((systematic or evidence) N0 assess*) OR AB ((systematic or evidence) N0 assess*)
61. TI (qualitative N1 (evidence or synthes*)) OR AB (qualitative N1 (evidence or synthes*))
62. TI overarching N1 model OR AB overarching N1 model
63. TI "review* of reviews" OR AB "review* of reviews"
64. S58 OR S59 OR S60 OR S61 OR S62 OR S63
65. S32 AND S64 [Drugs that cause dependency search results]
66. S57 AND S64 [Statins search results]

Notes: Date limited 2010 to date of search.

Database: Epistemonikos
Host: https://www.epistemonikos.org/en/
Issue: n/a
Date Searched: 12/8/2020
Searcher: SB
Hits: 56
Strategy

1. title:(prescription* OR prescribing OR prescribe*) OR abstract:(prescription* OR prescribing OR prescribe*)

For peer review only - http://bmjopen.bmj.com/site/about/guidelines.xhtml

2. title:(optimi* OR inappropriat* OR enhance*) OR abstract:(optimi* OR inappropriat* OR enhance*)
3. title:(benzodiazepine*) OR abstract:(benzodiazepine*) OR title:(antidepres* OR "anti depres*") OR abstract:(antidepres* OR "anti depres*") OR title:(opioid* OR opiate*) OR abstract:(opioid* OR opiate*) OR title:("z drug*") OR abstract:("z drug*") OR title:(gabapentin*) OR abstract:(gabapentin*)
4. 1 AND 2 AND 3

Notes: Date limited 2010 to 2020.

Database: medRxiv
Host: 12/8/2020
Issue: n/a
Date Searched:
Searcher: SB
Hits: 87
Strategy:

optimi* and prescri* and benzodiazepine *	5 hits
optimi* and prescri* and antidepres*	5 hits
optimi* and prescri* and opioid*	11 hits
optimi* and prescri* and "z drug*	66 hits
optimi* and prescri* and gabapentin*	0 hits

Database: Embase
Host: Ovid
Issue: 1974 to 2020 August 10
Date Searched: 11/8/2020
Searcher: SB
Hits: 1776
Strategy:

1. ((appropriate* or discontinu* or enhance* or inappropriate* or incorrect* or "in correct*" or optim* or safe or suboptim* or "sub optim*" or tapering or withdrawal) adj4 (drug* or medicine* or medication* or prescri*)).tw.
2. exp *inappropriate prescribing/
3. *prescription/
4. ((drug* or guideline* or guidance or medicine* or medication* or patient* or prescri*) adj4 (adhere* or compliance or concordance)).tw.
5. exp *patient compliance/
6. medication compliance/

For peer review only

7. ((shared or sharing or informed) adj2 (decision* or choice*)).tw.
8. (decision adj2 (aid* or support*)).tw.
9. exp *decision making/
10. exp *decision support system/
11. ((consumer* or patient*) adj3 (involv* or participat*)).tw.
12. *patient participation/
13. ("patient cent*" adj2 (approach* or care or decision* or intervention* or treatment*)).tw.
14. *patient care/
15. (behavi* adj2 chang*).tw.
16. *behavior change/
17. (restriction adj2 (policy or policies)).tw.
18. or/1-17
19. benzodiazepine*.tw.
20. (alprazolam or flunitrazepam or chlordiazepoxide or clobazam or clonazepam or diazepam or lorazepam or midazolam or nitrazepam or oxazepam or prazepam or temazepam).tw.
21. exp benzodiazepine derivative/
22. (antidepres* or "anti depres*").tw.
23. (serotonin or norepinephrine or noradrenaline or neurotransmitter* or dopamin* or SSRI* or SNRI* or NARI* or SARI* or NDRI* or tricyclic* or tetracyclic*).tw.
24. exp antidepressant agent/
25. (opioid* or opiate*).tw.
26. (morphine or hydromorphone or levorphanol or meperidine or methadone or propoxyphene of codeine or pentazocine or hydrocodone or oxycodone or fentanyl or tramadol).tw.
27. exp opiate/
28. "z drug*".tw.
29. (zopiclone or zolpidem or zaleplon or eszopiclone).tw.
30. hypnotic agent/
31. sedative agent/
32. (gabapentin* or mirogabalin or phenibut or pregabalin).tw.
33. gabapentin/
34. or/19-33
35. 18 and 34

For peer review only - http://bmjopen.bmj.com/site/about/guidelines.xhtml

36. statin*.tw.

37. ("HMG-CoA reductase inhibitor*" or "3-hydroxy-3-methyl-glutaryl-CoA reductase inhibitor*" or "3-hydroxy-3-methylglutaryl coenzyme A reductase inhibitor*" or "HMGCR inhibitor*" or "Hydroxymethylglutaryl-CoA Reductase Inhibitor*").tw.

38. "statin (protein)"/

39. (atorvastatin or Lipitor or Torvast).tw.

40. (fluvastatin or Lescol).tw.

41. (lovastatin or Mevacor or Altocor or Altoprev).tw.

42. (pitavastatin or Livalo or Pitava).tw.

43. (pravastatin or Pravachol or Selektine or Lipostat).tw.

44. (rosuvastatin or Crestor).tw.

45. (simvastatin or Zocor or Lipex).tw.

46. antihypertensive*.tw.

47. exp antihypertensive agent/

48. ("ACE inhibitor*" or antagonist or "angiotensin II receptor" or "beta blocker*" or "calcium channel blocker*" or "thiazide diuretic*").tw.

49. (acebutolol or adrenomedullin or alprenolol or amlodipine or atenolol or bendroflumethiazide or bepridil or betaxolol or bethanidine or bimatoprost or bisoprolol or bosentan or "bretylium tosylate" or brimonidine tartrate or bupranolol or captopril or carteolol or carvedilol or celiprolol or chlorisondamine or chlorothiazide or chlorthalidone or cilazapril or clonidine or cromakalim or cyclopenthiazide or debrisoquin or diazoxide or dihydralazine or dihydroalprenolol or diltiazem or doxazosin or enalapril or enalaprilat or eplerenone or epoprostenol or felodipine or fenoldopam or fosinopril or guanabenz or guanethidine or guanfacine or hexamethonium or "hexamethonium compound*" or hydralazine or hydrochlorothiazide or hydroflumethiazide or indapamide or indoramin or irbesartan or isradipine or kallidin or ketanserin or labetalol or latanoprost or lisinopril or losartan or mecamylamine or methyldopa or metipranolol or metolazone or metoprolol or mibefradil or minoxidil or muzolimine or nadolol or nebivolol or nicardipine or nicorandil or nimodipine or nisoldipine or nitrendipine or nitroprusside or olmesartan medoxomil or oxprenolol or pargyline or pempidine or penbutolol or "pentolinium tartrate" or perindopril or phenoxybenzamine or phentolamine or pinacidil or pindolol or piperoxan or polythiazide or prazosin or propranolol or protoveratrines or quinapril or ramipril or reserpine or rilmenidine or telmisartan or teprotide or terlipressin or ticrynafen or timolol or todralazine or tolazoline or torsemide or travoprost or trichlormethiazide or trimethaphan or valsartan or "veratrum alkaloid*" or vincamine or xipamide).tw.

50. or/36-49

51. cardiovascular.tw.

52. exp cardiovascular disease/

For peer review only - http://bmjopen.bmj.com/site/about/guidelines.xhtml

53. ((cardiac or coronary or heart) adj3 (arrest* or attack* or disease* or failure*)).tw.
54. ((heart or myocard* or transient) adj3 (infarc* or ischaemi* or ischemi*)).tw.
55. angina*.tw.
56. Angina Pectoris/
57. stroke*.tw.
58. exp cerebrovascular accident/
59. or/51-58
60. 18 and 50 and 59
61. ((effectiveness or implementation or literature or map or mapping or qualitative or rapid or realist or systematic or scoping or "state of the art" or umbrella) adj2 (assessment* or overview* or review* or synthes*)).tw.
62. ("meta analy*" or metaanaly* or metasynthe* or "meta synthe*").tw.
63. ((systematic or evidence) adj1 assess*).tw.
64. (qualitative adj2 (evidence or synthes*)).tw.
65. (overarching adj2 model).tw.
66. "review* of reviews".tw.
67. or/61-66
68. 35 and 67 [Drugs that cause dependency search results]
69. 60 and 67 [Statins search results]

Notes: Date limited 2010 to date of search.

Database: Health Management Information Consortium
Host: Ovid
Issue: 1979 to July 2020
Date Searched: 11/8/2020
Searcher: SB
Hits: 10
Strategy: see MEDLINE search strategy

Database: MEDLINE
Host: Ovid
Issue: 1946 to August 10, 2020
Date Searched: 11/8/2020
Searcher: SB
Hits: 875
Strategy:

For peer review only - http://bmjopen.bmj.com/site/about/guidelines.xhtml

For peer review only

1. ((appropriate* or discontinu* or enhance* or inappropriate* or incorrect* or "in correct*" or optim* or safe or suboptim* or "sub optim*" or tapering or withdrawal) adj4 (drug* or medicine* or medication* or prescri*)).tw.
2. Inappropriate Prescribing/
3. exp *Drug Prescriptions/
4. ((drug* or guideline* or guidance or medicine* or medication* or patient* or prescri*) adj4 (adhere* or compliance or concordance)).tw.
5. exp *Patient Compliance/
6. ((shared or sharing or informed) adj2 (decision* or choice*)).tw.
7. (decision adj2 (aid* or support*)).tw.
8. *Decision Making/
9. *decision support techniques/
10. ((consumer* or patient*) adj3 (involv* or participat*)).tw.
11. *patient participation/
12. ("patient cent*" adj2 (approach* or care or decision* or intervention* or treatment*)).tw.
13. Patient-Centered Care/
14. (behavi* adj2 chang*).tw.
15. (restriction adj2 (policy or policies)).tw.
16. or/1-15
17. benzodiazepine*.tw.
18. (alprazolam or flunitrazepam or chlordiazepoxide or clobazam or clonazepam or diazepam or lorazepam or midazolam or nitrazepam or oxazepam or prazepam or temazepam).tw.
19. exp Benzodiazepines/
20. (antidepres* or "anti depres*").tw.
21. (serotonin or norepinephrine or noradrenaline or neurotransmitter* or dopamin* or SSRI* or SNRI* or NARI* or SARI* or NDRI* or tricyclic* or tetracyclic*).tw.
22. exp Antidepressive Agents/
23. (opioid* or opiate*).tw.
24. (morphine or hydromorphone or levorphanol or meperidine or methadone or propoxyphene of codeine or pentazocine or hydrocodone or oxycodone or fentanyl or tramadol).tw.
25. exp Analgesics, Opioid/
26. "z drug*".tw.
27. (zopiclone or zolpidem or zaleplon or eszopiclone).tw.

For peer review only

For peer review only - http://bmjopen.bmj.com/site/about/guidelines.xhtml

28. exp "hypnotics and sedatives"/
29. (gabapentin* or mirogabalin or phenibut or pregabalin).tw.
30. Gabapentin/
31. or/17-30
32. 16 and 31
33. statin*.tw.
34. ("HMG-CoA reductase inhibitor*" or "3-hydroxy-3-methyl-glutaryl-CoA reductase inhibitor*" or "3-hydroxy-3-methylglutaryl coenzyme A reductase inhibitor*" or "HMGCR inhibitor*" or "Hydroxymethylglutaryl-CoA Reductase Inhibitor*").tw.
35. exp Hydroxymethylglutaryl-CoA Reductase Inhibitors/
36. (atorvastatin or Lipitor or Torvast).tw.
37. (fluvastatin or Lescol).tw.
38. (lovastatin or Mevacor or Altocor or Altoprev).tw.
39. (pitavastatin or Livalo or Pitava).tw.
40. (pravastatin or Pravachol or Selektine or Lipostat).tw.
41. (rosuvastatin or Crestor).tw.
42. (simvastatin or Zocor or Lipex).tw.
43. antihypertensive*.tw.
44. exp Antihypertensive Agents/
45. ("ACE inhibitor*" or antagonist or "angiotensin II receptor" or "beta blocker*" or "calcium channel blocker*" or "thiazide diuretic*").tw.
46. (acebutolol or adrenomedullin or alprenolol or amlodipine or atenolol or bendroflumethiazide or bepridil or betaxolol or bethanidine or bimatoprost or bisoprolol or bosentan or "bretylium tosylate" or brimonidine tartrate or bupranolol or captopril or carteolol or carvedilol or celiprolol or chlorisondamine or chlorothiazide or chlorthalidone or cilazapril or clonidine or cromakalim or cyclopenthiazide or debrisoquin or diazoxide or dihydralazine or dihydroalprenolol or diltiazem or doxazosin or enalapril or enalaprilat or eplerenone or epoprostenol or felodipine or fenoldopam or fosinopril or guanabenz or guanethidine or guanfacine or hexamethonium or "hexamethonium compound*" or hydralazine or hydrochlorothiazide or hydroflumethiazide or indapamide or indoramin or irbesartan or isradipine or kallidin or ketanserin or labetalol or latanoprost or lisinopril or losartan or mecamylamine or methyldopa or metipranolol or metolazone or metoprolol or mibefradil or minoxidil or muzolimine or nadolol or nebivolol or nicardipine or nicorandil or nimodipine or nisoldipine or nitrendipine or nitroprusside or olmesartan medoxomil or oxprenolol or pargyline or pempidine or penbutolol or "pentolinium tartrate" or perindopril or phenoxybenzamine or phentolamine or pinacidil or pindolol or piperoxan or polythiazide or prazosin or propranolol or protoveratrines or quinapril or ramipril or reserpine or rilmenidine or telmisartan or teprotide or terlipressin or ticrynafen or timolol or todralazine

For peer review only - http://bmjopen.bmj.com/site/about/guidelines.xhtml

or tolazoline or torsemide or travoprost or trichlormethiazide or trimethaphan or valsartan or "veratrum alkaloid*" or vincamine or xipamide).tw.

47. or/33-46
48. cardiovascular.tw.
49. exp Cardiovascular Diseases/
50. ((cardiac or coronary or heart) adj3 (arrest* or attack* or disease* or failure*)).tw.
51. ((heart or myocard* or transient) adj3 (infarc* or ischaemi* or ischemi*)).tw.
52. angina*.tw.
53. Angina Pectoris/
54. stroke*.tw.
55. exp Stroke/
56. or/48-55
57. 16 and 47 and 56
58. ((effectiveness or implementation or literature or map or mapping or qualitative or rapid or realist or systematic or scoping or "state of the art" or umbrella) adj2 (assessment* or overview* or review* or synthes*)).tw.
59. ("meta analy*" or metaanaly* or metasynthe* or "meta synthe*").tw.
60. ((systematic or evidence) adj1 assess*).tw.
61. (qualitative adj2 (evidence or synthes*)).tw.
62. (overarching adj2 model).tw.
63. "review* of reviews".tw.
64. systematic review.pt.
65. meta-analysis.pt.
66. or/58-65
67. 32 and 66 [Drugs that cause dependency search results]
68. 57 and 66 [Statins search results]

Notes: Date limited 2010 to date of search.

Database: PsycInfo
Host: Ovid
Issue: 1806 to August Week 1 2020
Date Searched: 11/8/2020
Searcher: SB

For peer review only

For peer review only - http://bmjopen.bmj.com/site/about/guidelines.xhtml

Hits: 297
Strategy

1. ((appropriate* or discontinu* or enhance* or inappropriate* or incorrect* or "in correct*" or optim* or safe or suboptim* or "sub optim*" or tapering or withdrawal) adj4 (drug* or medicine* or medication* or prescri*)).tw.
2. prescription drug misuse/
3. exp "prescribing (drugs)"/
4. ((drug* or guideline* or guidance or medicine* or medication* or patient* or prescri*) adj4 (adhere* or compliance or concordance)).tw.
5. *treatment compliance/
6. ((shared or sharing or informed) adj2 (decision* or choice*)).tw.
7. (decision adj2 (aid* or support*)).tw.
8. Decision Making/
9. ((consumer* or patient*) adj3 (involv* or participat*)).tw.
10. patient participation/
11. ("patient cent*" adj2 (approach* or care or decision* or intervention* or treatment*)).tw.
12. (behavi* adj2 chang*).ti,ab.
13. exp behavior change/
14. (restriction adj2 (policy or policies)).tw.
15. or/1-14
16. benzodiazepine*.tw.
17. (alprazolam or flunitrazepam or chlordiazepoxide or clobazam or clonazepam or diazepam or lorazepam or midazolam or nitrazepam or oxazepam or prazepam or temazepam).tw.
18. exp Benzodiazepines/
19. (antidepres* or "anti depres*").tw.
20. (serotonin or norepinephrine or noradrenaline or neurotransmitter* or dopamin* or SSRI* or SNRI* or NARI* or SARI* or NDRI* or tricyclic* or tetracyclic*).tw.
21. exp antidepressant drugs/
22. (opioid* or opiate*).tw.
23. (morphine or hydromorphone or levorphanol or meperidine or methadone or propoxyphene of codeine or pentazocine or hydrocodone or oxycodone or fentanyl or tramadol).tw.
24. exp opiates/
25. "z drug*".tw.

For peer review only - http://bmjopen.bmj.com/site/about/guidelines.xhtml

For peer review only

26. (zopiclone or zolpidem or zaleplon or eszopiclone).tw.
27. exp hypnotic drugs/
28. exp sedatives/
29. (gabapentin* or mirogabalin or phenibut or pregabalin).tw.
30. Gabapentin/
31. or/16-30
32. 15 and 31
33. statin*.tw.
34. ("HMG-CoA reductase inhibitor*" or "3-hydroxy-3-methyl-glutaryl-CoA reductase inhibitor*" or "3-hydroxy-3-methylglutaryl coenzyme A reductase inhibitor*" or "HMGCR inhibitor*" or "Hydroxymethylglutaryl-CoA Reductase Inhibitor*").tw.
35. statins/
36. (atorvastatin or Lipitor or Torvast).tw.
37. (fluvastatin or Lescol).tw.
38. (lovastatin or Mevacor or Altocor or Altoprev).tw.
39. (pitavastatin or Livalo or Pitava).tw.
40. (pravastatin or Pravachol or Selektine or Lipostat).tw.
41. (rosuvastatin or Crestor).tw.
42. (simvastatin or Zocor or Lipex).tw.
43. antihypertensive*.tw.
44. exp antihypertensive drugs/
45. ("ACE inhibitor*" or antagonist or "angiotensin II receptor" or "beta blocker*" or "calcium channel blocker*" or "thiazide diuretic*").tw.
46. (acebutolol or adrenomedullin or alprenolol or amlodipine or atenolol or bendroflumethiazide or bepridil or betaxolol or bethanidine or bimatoprost or bisoprolol or bosentan or "bretylium tosylate" or brimonidine tartrate or bupranolol or captopril or carteolol or carvedilol or celiprolol or chlorisondamine or chlorothiazide or chlorthalidone or cilazapril or clonidine or cromakalim or cyclopenthiazide or debrisoquin or diazoxide or dihydralazine or dihydroalprenolol or diltiazem or doxazosin or enalapril or enalaprilat or eplerenone or epoprostenol or felodipine or fenoldopam or fosinopril or guanabenz or guanethidine or guanfacine or hexamethonium or "hexamethonium compound*" or hydralazine or hydrochlorothiazide or hydroflumethiazide or indapamide or indoramin or irbesartan or isradipine or kallidin or ketanserin or labetalol or latanoprost or lisinopril or losartan or mecamylamine or methyldopa or metipranolol or metolazone or metoprolol or mibefradil or minoxidil or muzolimine or nadolol or nebivolol or nicardipine or nicorandil or nimodipine or nisoldipine or nitrendipine or nitroprusside or olmesartan medoxomil or oxprenolol or pargyline or pempidine or penbutolol or "pentolinium tartrate" or perindopril

For peer review only - http://bmjopen.bmj.com/site/about/guidelines.xhtml

or phenoxybenzamine or phentolamine or pinacidil or pindolol or piperoxan or polythiazide or prazosin or propranolol or protoveratrines or quinapril or ramipril or reserpine or rilmenidine or telmisartan or teprotide or terlipressin or ticrynafen or timolol or todralazine or tolazoline or torsemide or travoprost or trichlormethiazide or trimethaphan or valsartan or "veratrum alkaloid*" or vincamine or xipamide).tw.

47. or/33-46
48. cardiovascular.tw.
49. exp cardiovascular disorders/
50. ((cardiac or coronary or heart) adj3 (arrest* or attack* or disease* or failure*)).tw.
51. ((heart or myocard* or transient) adj3 (infarc* or ischaemi* or ischemi*)).tw.
52. angina*.tw.
53. Angina Pectoris/
54. stroke*.tw.
55. exp cerebral ischemia/
56. or/48-55
57. 15 and 47 and 56
58. ((effectiveness or implementation or literature or map or mapping or qualitative or rapid or realist or systematic or scoping or "state of the art" or umbrella) adj2 (assessment* or overview* or review* or synthes*)).tw.
59. ("meta analy*" or metaanaly* or metasynthe* or "meta synthe*").tw.
60. ((systematic or evidence) adj1 assess*).tw.
61. (qualitative adj2 (evidence or synthes*)).tw.
62. (overarching adj2 model).tw.
63. "review* of reviews".tw.
64. systematic review.pt.
65. meta-analysis.pt.
66. or/58-65
67. 32 and 66 [Drugs that cause dependency search results]
68. 57 and 66 [Statins search results]

Notes: Date limited 2010 to date of search.

Database: Conference Proceedings Citation Index – Science (CPCI-S); Science Citation Index (SCI)
Host: Web of Science via Clarivate Analytics
Issue: n/a
Date Searched: 11/8/2020

For peer review only - http://bmjopen.bmj.com/site/about/guidelines.xhtml

For peer review only

Searcher: SB
Hits: 1030
Strategy:

1. TOPIC =((appropriate* or discontinu* or enhance* or inappropriate* or incorrect* or "in correct*" or optim* or safe or suboptim* or "sub optim*" or tapering or withdrawal) near/3 (drug* or medicine* or medication* or prescri*))
2. TOPIC =((drug* or guideline* or guidance or medicine* or medication* or patient* or prescri*) near/3 (adhere* or compliance or concordance))
3. TOPIC =((shared or sharing or informed) near/1 (decision* or choice*))
4. TOPIC =((consumer* or patient*) near/2 (involv* or participat*))
5. TOPIC =("patient cent*" near/1 (approach* or care or decision* or intervention* or treatment*))
6. TOPIC =(decision near/1 (aid* or support*))
7. TOPIC =(behavi* near/1 chang*)
8. TOPIC =(restriction near/1 (policy or policies))
9. #8 OR #7 OR #6 OR #5 OR #4 OR #3 OR #2 OR #1
10. TOPIC: (benzodiazepine*)
11. TOPIC: ((alprazolam or flunitrazepam or chlordiazepoxide or clobazam or clonazepam or diazepam or lorazepam or midazolam or nitrazepam or oxazepam or prazepam or temazepam))
12. TOPIC: ((antidepres* or "anti depres*"))
13. TOPIC: ((serotonin or norepinephrine or noradrenaline or neurotransmitter* or dopamin* or SSRI* or SNRI* or NARI* or SARI* or NDRI* or tricyclic* or tetracyclic*))
14. TOPIC: ((opioid* or opiate*))
15. TOPIC: ((morphine or hydromorphone or levorphanol or meperidine or methadone or propoxyphene of codeine or pentazocine or hydrocodone or oxycodone or fentanyl or tramadol))
16. TOPIC: ("z drug*")
17. TOPIC: ((zopiclone or zolpidem or zaleplon or eszopiclone))
18. TOPIC: ((gabapentin* or mirogabalin or phenibut or pregabalin))
19. #18 OR #17 OR #16 OR #15 OR #14 OR #13 OR #12 OR #11 OR #10
20. #19 AND #9
21. TOPIC: (statin*)
22. TOPIC: ("HMG-CoA reductase inhibitor*" or "3-hydroxy-3-methyl-glutaryl-CoA reductase inhibitor*" or "3-hydroxy-3-methylglutaryl coenzyme A reductase inhibitor*" or "HMGCR inhibitor*" or "Hydroxymethylglutaryl-CoA Reductase Inhibitor*")

For peer review only - http://bmjopen.bmj.com/site/about/guidelines.xhtml

23. TOPIC: (atorvastatin or Lipitor or Torvast)
24. TOPIC: (fluvastatin or Lescol)
25. TOPIC: (lovastatin or Mevacor or Altocor or Altoprev)
26. TOPIC: (pitavastatin or Livalo or Pitava)
27. TOPIC: (pravastatin or Pravachol or Selektine or Lipostat)
28. TOPIC: (rosuvastatin or Crestor)
29. TOPIC: (simvastatin or Zocor or Lipex)
30. TOPIC: (antihypertensive*)
31. TOPIC: ("ACE inhibitor*" or antagonist or "angiotensin II receptor" or "beta blocker*" or "calcium channel blocker*" or "thiazide diuretic*")
32. TOPIC: (acebutolol or adrenomedullin or alprenolol or amlodipine or atenolol or bendroflumethiazide or bepridil or betaxolol or bethanidine or bimatoprost or bisoprolol or bosentan or "bretylium tosylate" or brimonidine tartrate or bupranolol or captopril or carteolol or carvedilol or celiprolol or chlorisondamine or chlorothiazide or chlorthalidone or cilazapril or clonidine or cromakalim or cyclopenthiazide or debrisoquin or diazoxide or dihydralazine or dihydroalprenolol or diltiazem or doxazosin or enalapril or enalaprilat or eplerenone or epoprostenol or felodipine or fenoldopam or fosinopril or guanabenz or guanethidine or guanfacine or hexamethonium or "hexamethonium compound*" or hydralazine or hydrochlorothiazide or hydroflumethiazide or indapamide or indoramin or irbesartan or isradipine or kallidin or ketanserin or labetalol or latanoprost or lisinopril or losartan or mecamylamine or methyldopa or metipranolol or metolazone or metoprolol or mibefradil or minoxidil or muzolimine or nadolol or nebivolol or nicardipine or nicorandil or nimodipine or nisoldipine or nitrendipine or nitroprusside or olmesartan medoxomil or oxprenolol or pargyline or pempidine or penbutolol or "pentolinium tartrate" or perindopril or phenoxybenzamine or phentolamine or pinacidil or pindolol or piperoxan or polythiazide or prazosin or propranolol or protoveratrines or quinapril or ramipril or reserpine or rilmenidine or telmisartan or teprotide or terlipressin or ticrynafen or timolol or todralazine or tolazoline or torsemide or travoprost or trichlormethiazide or trimethaphan or valsartan or "veratrum alkaloid*" or vincamine or xipamide)
33. #32 OR #31 OR #30 OR #29 OR #28 OR #27 OR #26 OR #25 OR #24 OR #23 OR #22 OR #21
34. TOPIC =((cardiac or coronary or heart) near/2 (arrest* or attack* or disease* or failure*))
35. TOPIC: (cardiovascular)
36. TS=((heart or myocard* or transient) near/2 (infarc* or ischaemi* or ischemi*))
37. TOPIC =angina*
38. TOPIC =stroke*
39. #38 OR #37 OR #36 OR #35 OR #34

For peer review only

For peer review only - http://bmjopen.bmj.com/site/about/guidelines.xhtml

40. TOPIC =((effectiveness or implementation or literature or map or mapping or qualitative or rapid or realist or systematic or scoping or "state of the art" or umbrella) near/1 (assessment* or overview* or review* or synthes*))
41. TOPIC =("meta analy*" or metaanaly* or metasynthe* or "meta synthe*")
42. TOPIC =((systematic or evidence) near/0 assess*)
43. TOPIC =(qualitative near/1 (evidence or synthes*))
44. TOPIC =(overarching near/1 model)
45. TOPIC ="review* of reviews"
46. #45 OR #44 OR #43 OR #42 OR #41 OR #40
47. #46 AND #20 [Drugs that cause dependency search results]
48. #46 AND #39 AND #33 AND #9 [Statins search results]

Table 1: Drugs that can cause dependency and antidepressant search results

Database	Hits
**Cochrane Database of Systematic Reviews**	111
**CINAHL**	372
**Epistemonikos**	56
**medRxiv**	87
**Embase**	1776
**HMIC**	10
**MEDLINE**	875
**PsycInfo**	297
**Web of Science (CPCI-S/SCI)**	1030
**TOTAL RECORDS**	**4614**
**DUPLICATE RECORDS**	**1898**
**UNIQUE RECORDS**	**2716**

Statins

Database: Cochrane Database of Systematic Reviews
Host: Cochrane Library
Issue: Issue 8 of 12, August 2020
Date Searched: 10/8/2020
Searcher: SB
Hits: 33
Strategy: see line 57 of Cochrane Database search above.
Notes: date limited 2010 to date of search.

Database: CINAHL
Host: EBSCO
Issue: n/a
Date Searched: 10/8/2020
Searcher: SB

For peer review only - http://bmjopen.bmj.com/site/about/guidelines.xhtml

Hits: 173
Strategy: see line 66 of CINAHL search above.
Notes: Date limited 2010 to date of search.

Database: Epistemonikos
Host: https://www.epistemonikos.org/en/
Issue: n/a
Date Searched: 10/8/2020
Searcher: SB
Hits: 16
Strategy:

1. title:(prescription* OR prescribing OR prescribe*) OR abstract:(prescription* OR prescribing OR prescribe*)
2. title:(optimi* OR inappropriat* OR enhance*) OR abstract:(optimi* OR inappropriat* OR enhance*)
3. title:(statin*) OR abstract:(statin*) OR title:(antihypertensive*) OR abstract:(antihypertensive*)
4. 1 AND 2 AND 3 [Statins search results]

Notes: Date limited 2010 to 2020 and limited to systematic reviews publication type.

Database: medRxiv
Host: https://www.medrxiv.org/
Issue: n/a
Date Searched: 10/8/2020
Searcher: SB
Hits: 8
Strategy:

optimi* and prescri* and statin*	7 hits
optimi* and prescri* and antihypertensive*	1 hit

Notes: Date limited 2010 to date of search.

Database: Embase
Host: Ovid
Issue: 1974 to 2020 August 07
Date Searched: 10/8/2020
Searcher: SB
Hits: 903
Strategy: see line 69 of Embase search above.
Notes: Date limited 2010 to date of search.

Database: HMIC
Host: Ovid
Issue: 1979 to May 2020
Date Searched: 10/8/2020

For peer review only - http://bmjopen.bmj.com/site/about/guidelines.xhtml

Searcher: SB
Hits: 0
Strategy: see line 68 of MEDLINE search strategy above.
Notes: Date limited 2010 to date of search.

Database: MEDLINE ALL
Host: Ovid
Issue: 1946 to August 07, 2020
Date Searched: 10/8/2020
Searcher: SB
Hits: 371
Strategy: see line 68 of MEDLINE search strategy above.
Notes: Date limited 2010 to date of search.

Database: PsycInfo
Host: Ovid
Issue: 1806 to August Week 1 2020
Date Searched: 10/8/2020
Searcher: SB
Hits: 17
Strategy: see line 68 of PsycInfo search strategy above.
Notes: Date limited 2010 to date of search.

Database: Conference Proceedings Citation Index – Science (CPCI-S); Science Citation Index (SCI)
Host: Web of Science via Clarivate Analytics
Issue: n/a
Date Searched: 10/8/2020
Searcher: SB
Hits: 638
Strategy: see line 48 of CPCI-S/SCI search strategy above.

Table 2: Statin search results

Database	Hits
**Cochrane Database of Systematic Reviews**	33
**CINAHL**	173
**Epistemonikos**	16
**medRxiv**	8
**EMBASE**	903
**HMIC**	0
**MEDLINE**	371
**PsycInfo**	17
**Web of Science (CPCI-S/SCI)**	638
**TOTAL RECORDS**	**2159**
**DUPLICATE RECORDS**	**714**
**UNIQUE RECORDS**	**1445**

Website searches

For peer review only - http://bmjopen.bmj.com/site/about/guidelines.xhtml

DCDs

Website: Royal Pharmaceutical Society Library Catalogue
URL: https://rpsgb.koha-ptfs.co.uk/
Date Searched: 21/10/2020
Searcher: SB
Hits: 73 hits
Strategy:

Advanced search setting.

Keyword: opioid* OR

Keyword: benzodiazepine* OR

Keyword: antidepressant* OR

Keyword: gabapentinoid*

Website: Royal College of Physicians Library Catalogue
URL: https://rcp.soutron.net/Portal/Default/en-GB/Search/SimpleSearch
Date Searched: 21/10/2020
Searcher: SB
Hits: 6
Strategy:

All fields: opioid OR opioids OR benzodiazepine OR benzodiazepines OR antidepressant OR antidepressants OR gabapentinoid OR gabapentinoids

Website: Royal College of Psychiatrists Library Catalogue
URL: https://rcpsych.soutron.net/Portal/Default/en-GB/Search/SimpleSearch
Date Searched: 21/10/2020
Searcher: SB
Hits: 20
Strategy:

opioid* OR benzodiazepine* OR antidepressant* OR gabapentinoid*

Website: Mind
URL: https://www.mind.org.uk/
Date Searched: 21/10/2020
Searcher: SB
Hits: 0
Strategy: Browsed website for relevant studies

Website: Mental Health UK
URL: https://mentalhealth-uk.org/

For peer review only - http://bmjopen.bmj.com/site/about/guidelines.xhtml

Date Searched: 21/10/2020
Searcher: SB
Hits: 0
Strategy: Browsed website for relevant studies

Website: Priory Group
URL: https://www.priorygroup.com/
Date Searched: 21/10/2020
Searcher: SB
Hits: 0
Strategy: Browsed website for relevant studies

Statins

Website: Royal Pharmaceutical Society Library Catalogue
URL: https://rpsgb.koha-ptfs.co.uk/
Date Searched: 21/10/2020
Searcher: SB
Hits: 13
Strategy:

Advanced search setting.

Keyword:	statin*	OR
Keyword:	"ace inhibitor*"	OR
Keyword:	antihypertensive*	

Website: Royal College of Physicians Library Catalogue
URL: https://rcp.soutron.net/Portal/Default/en-GB/Search/SimpleSearch
Date Searched: 21/10/2020
Searcher: SB
Hits: 6
Strategy:

All fields: statin OR statins OR "ace inhibitor" OR "ace inhibitors" OR antihypertensive OR antihypertensives

Website: British Cardiovascular Society
URL: https://www.britishcardiovascularsociety.org/
Date Searched: 21/10/2020
Searcher: SB
Hits: 0
Strategy: Browsed website for relevant studies.

For peer review only - http://bmjopen.bmj.com/site/about/guidelines.xhtml

Website: European Society of Cardiology
URL: https://www.escardio.org/
Date Searched: 21/10/2020
Searcher: SB
Hits: 0
Strategy: Browsed website for relevant studies

Website: British and Irish Hypertension Society
URL: https://bihsoc.org/
Date Searched: 21/10/2020
Searcher: SB
Hits: 0
Strategy: Browsed website for relevant studies

Website: British Heart Foundation
URL: https://www.bhf.org.uk/
Date Searched: 21/10/2020
Searcher: SB
Hits: 31 (sum total of each keyword search)
Strategy: Searched for keywords "statin", "ace inhibitor" or "antihypertensive" (one term per search) and viewed the publications and research tabs.

Website: Heart Research UK
URL: https://heartresearch.org.uk/
Date Searched: 21/10/2020
Searcher: SB
Hits: 0
Strategy: Browsed website for relevant studies

For peer review only

For peer review only - http://bmjopen.bmj.com/site/about/guidelines.xhtml

APPENDIX B: REASONS FOR EXCLUSION AT FULL TEXT

**Reference**	**Reason for exclusion**
Abstracts from Professional Poster Presentations at AMCP's 2010 Educational Conference. *Journal of Managed Care Pharmacy* 2010;**16**.	Abstract proceedings
The Canadian Association for Population Therapeutics. November 17th - 19th, 2013 Toronto, Ontario. "Healthcare Cost, Quality, and Policy: Driving Stakeholder Innovation in Process and Practice". *Journal of Population Therapeutics and Clinical Pharmacology* 2014;**21**:e116.	Abstract proceedings
Transcatheter Cardiovascular Therapeutics Asia Pacific, 19th Cardiovascular Summit: TCTAP 2014. *Journal of the American College of Cardiology* 2014;**63**.	Abstract proceedings
Abstracts of Papers Presented at the Health Services Research and Pharmacy Practice Conference, HSRPP 2016. *International Journal of Pharmacy Practice* 2016;**24**.	Abstract proceedings
12th Annual Scientific Meeting of Indonesian Society of Hypertension, INASH. *Journal of Hypertension* 2018;**36**.	Abstract proceedings
Selected abstracts from the EGPRN meeting in Tampere, Finland, 9–12 May 2019. *European Journal of General Practice* 2019;**25**:164-75.	Abstract proceedings
46th ESAO Congress 3-7 September 2019 Hannover, Germany Abstracts. *International Journal of Artificial Organs* 2019;**42**:386-474.	Abstract proceedings
AbuDagga A, Resnick HE, Alwan M. Impact of blood pressure telemonitoring on hypertension outcomes: a literature review. *Telemed J E Health* 2010;**16**:830-8. https://doi.org/10.1089/tmj.2010.0015	Not SR
Akeroyd JM, Chan WJ, Kamal AK, Palaniappan L, Virani SS. Adherence to cardiovascular medications in the South Asian population: A systematic review of current evidence and future directions. *World J Cardiol* 2015;**7**:938-47. https://doi.org/10.4330/wjc.v7.i12.938	Not SR
Al AlShaikh S, Quinn T, Dunn W, Dawson J. Interventions to Enhance Adherence to Secondary Preventative Medication After Stroke a Systematic Review and Meta-Analysis. *Stroke* 2016;**47**.	Abstract
Alford DP. Chronic back pain with possible prescription opioid misuse. *JAMA* 2013;**309**:919-25. https://doi.org/10.1001/jama.2013.522	Not SR
AlGhurair SA, Hughes CA, Simpson SH, Guirguis LM. A systematic review of patient self-reported barriers of adherence to antihypertensive medications using the world health organization multidimensional adherence model. *J Clin Hypertens (Greenwich)* 2012;**14**:877-86. https://doi.org/10.1111/j.1751-7176.2012.00699.x	Not SR

For peer review only - http://bmjopen.bmj.com/site/about/guidelines.xhtml

Alkhouli M, Noseworthy PA, Rihal CS, Holmes DR, Jr. Stroke Prevention in Nonvalvular Atrial Fibrillation: A Stakeholder Perspective. *J Am Coll Cardiol* 2018;**71**:2790-801. https://doi.org/10.1016/j.jacc.2018.04.013	Not SR
Almeida JT, Esteves AL, Martins F, Palma I. [Approach to Patients with Statin Intolerance: Evidence-Based Review]. *Acta Med Port* 2020;**33**:49-57. https://doi.org/10.20344/amp.10376	Non-English language (Duplicated excluded from update search)
AlShaikh SA, Quinn T, Dunn W, Dawson J. Interventions to enhance adherence to secondary preventative medication after stroke a systematic review and meta-analysis. *Stroke* 2016;**47**.	Duplicate
Anderson SL, Marrs JC. A Review of the Role of the Pharmacist in Heart Failure Transition of Care. *Adv Ther* 2018;**35**:311-23. https://doi.org/10.1007/s12325-018-0671-7	Not SR
Anonymous. Review of: Fuller RH et al. Improving adherence to medicines in people with heart disease: a systematic review. Heart. *Drug Ther Bull* 2018;**56**:114. https://doi.org/10.1136/dtb.2018.10.000021	Not SR
Armstrong SO, Little RA. Cost effectiveness of interventions to improve adherence to statin therapy in ASCVD patients in the United States. *Patient Prefer Adherence* 2019;**13**:1375-89. https://doi.org/10.2147/PPA.S213258	Not SR
Banerjee A, Khandelwal S, Nambiar L, Saxena M, Peck V, Moniruzzaman M, *et al.* Health system barriers and facilitators to medication adherence for the secondary prevention of cardiovascular disease: a systematic review. *Open Heart* 2016;**3**:e000438. https://doi.org/10.1136/openhrt-2016-000438	Quantitative focus
Basaraba JE, Picard M, George-Phillips K, Mysak T. Pharmacists as Care Providers for Stroke Patients: A Systematic Review. *Can J Neurol Sci* 2018;**45**:49-55. https://doi.org/10.1017/cjn.2017.233	Not SR
Bramlage P, Sims H, Minguet J, Ferrero C. The polypill: An effective approach to increasing adherence and reducing cardiovascular event risk. *Eur J Prev Cardiol* 2017;**24**:297-310. https://doi.org/10.1177/2047487316674817	Not SR
Brito JP, Montori VM. Reinitiation of statins after statin-associated musculoskeletal symptoms: a patient-centered approach. *Circ Cardiovasc Qual Outcomes* 2013;**6**:243-7. https://doi.org/10.1161/CIRCOUTCOMES.111.000039	Not SR
Buck HG, McGhee S, Polo RL, Zambroski C. Hypercholesterolemia Management in Older Adults: A Scoping Review of Recent Evidence. *J Gerontol Nurs* 2019;**45**:31-42. https://doi.org/10.3928/00989134-20190211-04	Quantitative focus

For peer review only - http://bmjopen.bmj.com/site/about/guidelines.xhtml

Calleja Rivero JL, Zerpa F, Rivera L. Fixed-dose versus separate drug combinations for antihypertensive treatment: literature review. *Medwave* 2016;**16**:e6549. https://doi.org/10.5867/medwave.2016.08.6549	Not SR
Campos K, Sheth S, Coulter SA. Hypertension Treatment ACCORDing to SPRINT. *Tex Heart Inst J* 2016;**43**:324-7. https://doi.org/10.14503/THIJ-16-5908	Not SR
Chan DK, O'Rourke F, Shen Q, Mak JC, Hung WT. Meta-analysis of the cardiovascular benefits of intensive lipid lowering with statins. *Acta Neurol Scand* 2011;**124**:188-95. https://doi.org/10.1111/j.1600-0404.2010.01450.x	Quantitative focus
Chapman RH, Ferrufino CP, Kowal SL, Classi P, Roberts CS. The cost and effectiveness of adherence-improving interventions for antihypertensive and lipid-lowering drugs*. *Int J Clin Pract* 2010;**64**:169-81. https://doi.org/10.1111/j.1742-1241.2009.02196.x	Not SR
Chee YJ, Chan HH, Tan NC. Understanding patients' perspective of statin therapy: can we design a better approach to the management of dyslipidaemia? A literature review. *Singapore Med J* 2014;**55**:416-21. https://doi.org/10.11622/smedj.2014099	Not SR
Choi H-I, Han KH. The ideal management of blood cholesterol: a review of the updated 2013 ACC/AHA cholesterol guideline for reducing cardiovascular risk. *Journal of the Korean Medical Association* 2014;**57**:857-65. https://doi.org/10.5124/jkma.2014.57.10.857	Non-English language
Clarkesmith DE, Pattison HM, Khaing PH, Lane DA. Educational and behavioural interventions for anticoagulant therapy in patients with atrial fibrillation. *Cochrane Database of Systematic Reviews* 2017; 10.1002/14651858.CD008600.pub3. https://doi.org/10.1002/14651858.CD008600.pub3	Drug not relevant
Coleman CI, Roberts MS, Sobieraj DM, Lee S, Alam T, Kaur R. Effect of dosing frequency on chronic cardiovascular disease medication adherence. *Curr Med Res Opin* 2012;**28**:669-80. https://doi.org/10.1185/03007995.2012.677419	Not SR
Conn VS, Ruppar TM, Chase JD. Blood pressure outcomes of medication adherence interventions: systematic review and meta-analysis. *J Behav Med* 2016;**39**:1065-75. https://doi.org/10.1007/s10865-016-9730-1	Quantitative focus
Cooke CE, Xing S, Gale SE, Peters S. Initial non-adherence to antihypertensive medications in the United States: a systematic literature review. Journal of human hypertension 2022;36:3-13. https://doi.org/https://dx.doi.org/10.1038/s41371-021-00549-w	Not SR
Cooke CE, Xing S, Gale SE, Peters S. Initial non-adherence to antihypertensive medications in the United States: a systematic literature review. J Hum Hypertens. 2022 Jan;36(1):3-13. doi: 10.1038/s41371-021-00549-w. Epub 2021 May 14. PMID: 33990698.	Duplicate

For peer review only - http://bmjopen.bmj.com/site/about/guidelines.xhtml

Crisafulli S, Luxi N, Coppini R, Capuano A, Scavone C, Zinzi A, et al. Anti-hypertensive drugs deprescribing: an updated systematic review of clinical trials. BMC family practice 2021;22:208. https://doi.org/https://dx.doi.org/10.1186/s12875-021-01557-y	Not SR
Cutrona SL, Choudhry NK, Fischer MA, Servi A, Liberman JN, Brennan TA*, et al.* Modes of delivery for interventions to improve cardiovascular medication adherence. *Am J Manag Care* 2010;**16**:929-42.	Not SR
de Barra M, Scott CL, Scott NW, Johnston M, de Bruin M, Nkansah N*, et al.* Pharmacist services for non-hospitalised patients. *Cochrane Database Syst Rev* 2018;**9**:CD013102. https://doi.org/10.1002/14651858.CD013102	Quantitative focus
Dobremez V, Ruel JH, Hugon A, Gay S, Berlioz J, Pineau-Blondel E. Contraindicated drugs in myasthenia gravis patients: Elaboration of a tool to optimize drug therapy. *International Journal of Clinical Pharmacy* 2015;**37**:236-7. https://doi.org/10.1007/s11096-014-0039-2	Abstract
Dunn BL, Teusink AC, Taber DJ, Hemstreet BA, Uber LA, Weimert NA. Management of hypertension in renal transplant patients: a comprehensive review of nonpharmacologic and pharmacologic treatment strategies. *Ann Pharmacother* 2010;**44**:1259-70. https://doi.org/10.1345/aph.1P004	Not SR
Elley CR, Gupta AK, Webster R, Selak V, Jun M, Patel A*, et al.* The efficacy and tolerability of 'polypills': meta-analysis of randomised controlled trials. *PLoS One* 2012;**7**:e52145. https://doi.org/10.1371/journal.pone.0052145	Quantitative focus
Elnaem MH, Rosley NFF, Alhifany AA, Elrggal ME, Cheema E. Impact of Pharmacist-Led Interventions on Medication Adherence and Clinical Outcomes in Patients with Hypertension and Hyperlipidemia: A Scoping Review of Published Literature. *J Multidiscip Healthc* 2020;**13**:635-45. https://doi.org/10.2147/JMDH.S257273	Not SR
Engel J, Damen NL, van der Wulp I, de Bruijne MC, Wagner C. Adherence to Cardiac Practice Guidelines in the Management of Non-ST-Elevation Acute Coronary Syndromes: A Systematic Literature Review. *Curr Cardiol Rev* 2017;**13**:3-27. https://doi.org/10.2174/1573403x12666160504100025	Quantitative focus
Gale N, Marshall T, Bramley G. Starting and staying on preventive medication for cardiovascular disease. *Curr Opin Cardiol* 2012;**27**:533-41. https://doi.org/10.1097/HCO.0b013e328356dae5	Not SR
Gandapur Y, Kianoush S, Kelli HM, Misra S, Urrea B, Blaha MJ*, et al.* The role of mHealth for improving medication adherence in patients with cardiovascular disease: a systematic review. *Eur Heart J Qual Care Clin Outcomes* 2016;**2**:237-44. https://doi.org/10.1093/ehjqcco/qcw018	Not SR

For peer review only

Gellad WF, Grenard JL, Marcum ZA. A systematic review of barriers to medication adherence in the elderly: looking beyond cost and regimen complexity. *Am J Geriatr Pharmacother* 2011;**9**:11-23. https://doi.org/10.1016/j.amjopharm.2011.02.004	Quantitative focus
Georgiopoulos G, Kollia Z, Katsi V, Oikonomou D, Tsioufis C, Tousoulis D. Nurse's Contribution to Alleviate Non-adherence to Hypertension Treatment. *Curr Hypertens Rep* 2018;**20**:65. https://doi.org/10.1007/s11906-018-0862-2	Not SR
Glynn L, Fahey T. Cardiovascular medication: improving adherence. *BMJ Clin Evid* 2011;**2011**.	Not SR
Golubev S. Compliance measurement-guided medication management programs in hypertension: A health technology assessment. *Journal of Hypertension* 2010;**28**:e269.	Quantitative focus
Golubev S, Fedoseyeva I, Johri M. Compliance measurement-guided medication management programs in hypertension: A systematic review. *Basic and Clinical Pharmacology and Toxicology* 2011;**109**:109-10. http://dx.doi.org/10.1111/j.1742-7843.2011.00722.x	Abstract
Gwadry-Sridhar FH, Manias E, Lal L, Salas M, Hughes DA, Ratzki-Leewing A, *et al.* Impact of interventions on medication adherence and blood pressure control in patients with essential hypertension: a systematic review by the ISPOR medication adherence and persistence special interest group. *Value Health* 2013;**16**:863-71. https://doi.org/10.1016/j.jval.2013.03.1631	Not SR
Hong J, Chak Tiu Y, Leung PYB, Wong MF, Ng WY, Cheung D, Yan Mok H, Lam WY, Li KY, Wong CKH, Interventions that improve adherence to antihypertensive medications in coronary heart disease patients: a systematic review, *Postgraduate Medical Journal*, Volume 98, Issue 1157, March 2022, Pages 219–227	Duplicate
Hopper I, Samuel R, Tonkin A, Krum H. The effects of discontinuing medications in stable systolic heart failure-systematic review and meta-analysis. *Heart Lung and Circulation* 2012;**21**:S95. http://dx.doi.org/10.1016/j.hlc.2012.05.241	Abstract
Imbeault B, Vallee M. Single-Pill Combinations in the Treatment of Hypertension in Adults: Beyond Convenience. *Can J Diabetes* 2018;**42**:205-8. https://doi.org/10.1016/j.jcjd.2018.01.011	Not SR
Iwelunmor J, Airhihenbuwa CO, Cooper R, Tayo B, Plange-Rhule J, Adanu R, *et al.* Prevalence, determinants and systems-thinking approaches to optimal hypertension control in West Africa. *Global Health* 2014;**10**:42. https://doi.org/10.1186/1744-8603-10-42	Not SR
Jamshidnezhad A, Kabootarizadeh L, Hoseini SM. The Effects of Smartphone Applications on Patients Self-care with Hypertension: A Systematic Review Study. *Acta Inform Med* 2019;**27**:263-7. https://doi.org/10.5455/aim.2019.27.263-267	Not SR

For peer review only - http://bmjopen.bmj.com/site/about/guidelines.xhtml

Jones LK, Gionfriddo MR, Tilberry S, Frisbie L, Gregor C, Gidding S*, et al.* Implementation strategies to improve statin utilization in individuals with hypercholesterolemia: A systematic review. *JACCP Journal of the American College of Clinical Pharmacy* 2020;**3**:365. http://dx.doi.org/10.1002/jac5.1204	Abstract
Jornten-Karlsson M, Pintat S, Molloy-Bland M, Berg S, Ahlqvist M. Patient-Centered Interventions to Improve Adherence to Statins: A Narrative Synthesis of Systematically Identified Studies. *Drugs* 2016;**76**:1447-65. https://doi.org/10.1007/s40265-016-0640-x	Not SR
Ju A, Hanson CS, Banks E, Korda R, Craig JC, Usherwood T*, et al.* Patient beliefs and attitudes to taking statins: systematic review of qualitative studies. *Br J Gen Pract* 2018;**68**:e408-e19. https://doi.org/10.3399/bjgp18X696365	Not SR
Ko DT, Jackevicius CA. Stopping beta-Blockers After Myocardial Infarction: Not So Fast! *Circ Cardiovasc Qual Outcomes* 2018;**11**:e004678. https://doi.org/10.1161/CIRCOUTCOMES.118.004678	Not SR
Krack G. EXPLANATIONS FOR THE DIFFERENCES BETWEEN EFFECTS ON ADHERENCE OF VALUE-BASED HEALTH INSURANCE DESIGNS: A SYSTEMATIC REVIEW AND META-ANALYSIS. *Value in Health* 2018;**21**:S155. http://dx.doi.org/10.1016/j.jval.2018.09.924	Abstract
Krueger K, Griese-Mammen N, Schubert I, Kieble M, Botermann L, Laufs U*, et al.* In search of a standard when analyzing medication adherence in patients with heart failure using claims data: a systematic review. *Heart Fail Rev* 2018;**23**:63-71. https://doi.org/10.1007/s10741-017-9656-x	Quantitative focus
Kwon A, Kim GH. Single-pill Combination Therapy of Azilsartan Medoxomil/Chlorthalidone for Treatment of Hypertension: A Systematic Review. *Clin Ther* 2020;**42**:1390-403. https://doi.org/10.1016/j.clinthera.2020.05.015	Not SR (Duplicate excluded in update search)
Lane D, Patel P, Khunti K, Gupta P. Objective measures of non-adherence in cardiometabolic diseases: a review focused on urine biochemical screening. *Patient Prefer Adherence* 2019;**13**:537-47. https://doi.org/10.2147/PPA.S162215	Not SR
Lee JL, Maciejewski M, Raju S, Shrank WH, Choudhry NK. Value-based insurance design: quality improvement but no cost savings. *Health Aff (Millwood)* 2013;**32**:1251-7. https://doi.org/10.1377/hlthaff.2012.0902	Not SR
Lennon O, Blake C, Booth J, Pollock A, Lawrence M. Interventions for behaviour change and self-management in stroke secondary prevention: protocol for an overview of reviews. *Syst Rev* 2018;**7**:231. https://doi.org/10.1186/s13643-018-0888-1	Protocol

For peer review only - http://bmjopen.bmj.com/site/about/guidelines.xhtml

Liu HL, Chen XY, Li JR, Su SW, Ding T, Shi CX*, et al.* Efficacy and Safety of Pulmonary Arterial Hypertension-specific Therapy in Pulmonary Arterial Hypertension: A Meta-analysis of Randomized Controlled Trials. *Chest* 2016;**150**:353-66. https://doi.org/10.1016/j.chest.2016.03.031	Quantitative focus
Logue J, Al-Ghibiwi H, Alamri AA, Preiss D. Systematic review of studies exploring reasons for statin non-adherence and of randomised controlled trials of interventions to improve adherence. *Atherosclerosis* 2015;**241**:e52.	Abstract
Madhavan MV, Gersh BJ, Alexander KP, Granger CB, Stone GW. Coronary Artery Disease in Patients >/=80 Years of Age. *J Am Coll Cardiol* 2018;**71**:2015-40. https://doi.org/10.1016/j.jacc.2017.12.068	Not SR
Marcum ZA, Handler SM, Boyce R, Gellad W, Hanlon JT. Medication misadventures in the elderly: a year in review. *Am J Geriatr Pharmacother* 2010;**8**:77-83. https://doi.org/10.1016/j.amjopharm.2010.02.002	Not SR
Matthes J, Albus C. Improving adherence with medication: a selective literature review based on the example of hypertension treatment. *Dtsch Arztebl Int* 2014;**111**:41-7. https://doi.org/10.3238/arztebl.2014.0041	Not SR
Mendes Soares M, Oliveira LeÆo e Silva L, Alberto Dias C, Maria Rodrigues S, Jorge Machado C. ADESAO DO IDOSO AO TRATAMENTO DA HIPERTENSAO ARTERIAL SISTEMICA: REVISAO INTEGRATIVA. *Cogitare Enfermagem* 2012;**17**:114-50.	Non-English language
Meza-Contreras A, Wenczenovicz C, Ruiz-Arellanos K, Vesely EAK, Mogollon R, Montori VM. Statin intolerance management: a systematic review. Endocrine. 2023 Mar;79(3):430-436. doi: 10.1007/s12020-022-03263-w. Epub 2022 Dec 2. PMID: 36459335.	Not SR
Molloy GJ, O'Carroll RE, Witham MD, McMurdo ME. Interventions to enhance adherence to medications in patients with heart failure: a systematic review. *Circ Heart Fail* 2012;**5**:126-33. https://doi.org/10.1161/CIRCHEARTFAILURE.111.964569	Not SR
Morgado M, Castanheira L, Verde I, Castelo-Branco M. Pharmacist interventions to enhance patient adherence to antihypertensive medication: A systematic review. *Journal of Clinical Hypertension* 2010;**12**:A112-A3. http://dx.doi.org/10.1111/j.1751-7176.2010.00282.x	Abstract
Morgado MP, Morgado SR, Mendes LC, Pereira LJ, Castelo-Branco M. Pharmacist interventions to enhance blood pressure control and adherence to antihypertensive therapy: Review and meta-analysis. *Am J Health Syst Pharm* 2011;**68**:241-53. https://doi.org/10.2146/ajhp090656	Not SR

For peer review only

For peer review only - http://bmjopen.bmj.com/site/about/guidelines.xhtml

Morrissey EC, Durand H, Nieuwlaat R, Navarro T, Haynes RB, Walsh JC*, et al.* Effectiveness and content analysis of interventions to enhance medication adherence in hypertension: a systematic review and meta-analysis protocol. *Syst Rev* 2016;**5**:96. https://doi.org/10.1186/s13643-016-0278-5	Protocol
Na. Selected Abstracts from the National Congress of the Italian Society of Hypertension (SIIA 2010). *High Blood Pressure & Cardiovascular Prevention* 2010;**17**:131-90. https://doi.org/10.2165/11311900-000000000-00000	Quantitative focus
Naderi SH, Bestwick JP, Wald DS. Adherence to drugs that prevent cardiovascular disease: meta-analysis on 376,162 patients. *Am J Med* 2012;**125**:882-7 e1. https://doi.org/10.1016/j.amjmed.2011.12.013	Not SR
Nguyen E, Sobieraj DM. The impact of appointment-based medication synchronization on medication taking behaviour and health outcomes: A systematic review. *J Clin Pharm Ther* 2017;**42**:404-13. https://doi.org/10.1111/jcpt.12554	Not SR
Nguyen T, Gurwitz J. Paper Abstract. *Journal of the American Geriatrics Society* 2016;**64**:S1-S311. https://doi.org/10.1111/jgs.14231	Quantitative focus
Ni Z, Dardas L, Wu B, Shaw R. Cardioprotective medication adherence among patients with coronary heart disease in China: a systematic review. *Heart Asia* 2019;**11**:e011173. https://doi.org/10.1136/heartasia-2018-011173	Not SR
Nielsen JO, Shrestha AD, Neupane D, Kallestrup P. Non-adherence to anti-hypertensive medication in low- and middle-income countries: a systematic review and meta-analysis of 92443 subjects. *J Hum Hypertens* 2017;**31**:14-21. https://doi.org/10.1038/jhh.2016.31	Quantitative focus
Nieuwlaat R, Connolly SJ, MacKay JA, Weise-Kelly L, Navarro T, Wilczynski NL, *et al.* Computerized clinical decision support systems for therapeutic drug monitoring and dosing: A decision maker-researcher partnership systematic review. *Circulation: Cardiovascular Quality and Outcomes* 2011;**4**.	Drug not relevant
Njie GJ, Mukhtar Q, Finnie RKC, Dhakal-Acharya S, Thota AB, Hopkins DP*, et al.* Effectiveness of reducing out-of-pocket costs for medications to treat hypertension and hyperlipidemia: A community guide systematic review. *Journal of the American Society of Hypertension* 2014;**8**:e124-e5. http://dx.doi.org/10.1016/j.jash.2014.03.285	Abstract
Ofori-Asenso R, Zoungas S, Liew D. Reinitiation of Statin Therapy After Discontinuation: A Meta-analysis. *Mayo Clin Proc* 2018;**93**:666-8. https://doi.org/10.1016/j.mayocp.2018.01.011	Quantitative focus
Oosterom-Calo R, van Ballegooijen AJ, Terwee CB, te Velde SJ, Brouwer IA, Jaarsma T*, et al.* Determinants of adherence to heart failure medication: a systematic literature review. *Heart Fail Rev* 2013;**18**:409-27. https://doi.org/10.1007/s10741-012-9321-3	Quantitative focus

For peer review only - http://bmjopen.bmj.com/site/about/guidelines.xhtml

Osborn D, Burton A, Walters K, Atkins L, Barnes T, Blackburn R*, et al. Primary care management of cardiovascular risk for people with severe mental illnesses: the Primrose research programme including cluster RCT*. Southampton (UK): NIHR Journals Library; 2019 Apr. http://doi.org/ 10.3310/pgfar07020	Quantitative focus
Pena RCF, Hofmann Bowman MA, Ahmad M, Pham J, Kline-Rogers E, Case MJ, Lee J, Eagle K; Aortic Dissection Collaborative. An assessment of the current medical management of thoracic aortic disease: A patient-centered scoping literature review. Semin Vasc Surg. 2022 Mar;35(1):16-34. doi: 10.1053/j.semvascsurg.2022.02.007. Epub 2022 Feb 18. PMID: 35501038.	Not SR
Perera R, McFadden E, McLellan J, Lung T, Clarke P, Perez T*, et al.* Optimal strategies for monitoring lipid levels in patients at risk or with cardiovascular disease: a systematic review with statistical and cost-effectiveness modelling. *Health Technol Assess* 2015;**19**:1-401, vii-viii. https://doi.org/10.3310/hta191000	Quantitative focus
Phan K, Gomez YH, Elbaz L, Daskalopoulou SS. Statin treatment non-adherence and discontinuation: clinical implications and potential solutions. *Curr Pharm Des* 2014;**20**:6314-24. https://doi.org/10.2174/1381612820666140620162629	Quantitative focus
Pinho S, Cruz M, Ferreira F, Ramalho A, Sampaio R. Improving medication adherence in hypertensive patients: A scoping review. Preventive medicine 2021;146:106467. https://doi.org/https://dx.doi.org/10.1016/j.ypmed.2021.106467	Not SR
Polinski JM, Donohue JM, Kilabuk E, Shrank WH. Medicare Part D's effect on the under- and overuse of medications: a systematic review. *J Am Geriatr Soc* 2011;**59**:1922-33. https://doi.org/10.1111/j.1532-5415.2011.03537.x	Quantitative focus
Powers BJ, Coeytaux RR, Dolor RJ, Hasselblad V, Patel UD, Yancy WS, Jr.*, et al.* Updated report on comparative effectiveness of ACE inhibitors, ARBs, and direct renin inhibitors for patients with essential hypertension: much more data, little new information. *J Gen Intern Med* 2012;**27**:716-29. https://doi.org/10.1007/s11606-011-1938-8	Quantitative focus
Rabien A, Price M. Development of a clinical decision support tool for the primary prevention of cardiovascular disease. *Canadian Family Physician* 2015;**61**:S2.	Abstract
Rahmawati R, Bajorek BV. Self-medication among people living with hypertension: a review. *Fam Pract* 2017;**34**:147-53. https://doi.org/10.1093/fampra/cmw137	Not SR
Reeve E, Jordan V, Thompson W, Sawan M, Todd A, Gammie TM*, et al.* Withdrawal of antihypertensive drugs in older people. *Cochrane Database Syst Rev* 2020;**6**:CD012572. https://doi.org/10.1002/14651858.CD012572.pub2	Quantitative focus

For peer review only - http://bmjopen.bmj.com/site/about/guidelines.xhtml

Ruppar TM, Dunbar-Jacob JM, Mehr DR, Lewis L, Conn VS. Medication adherence interventions among hypertensive black adults: a systematic review and meta-analysis. *J Hypertens* 2017;**35**:1145-54. https://doi.org/10.1097/HJH.0000000000001260	Full text not retrieved
Schedlbauer A, Davies P, Fahey T. Interventions to improve adherence to lipid lowering medication. *Cochrane Database Syst Rev* 2010; 10.1002/14651858.CD004371.pub3:CD004371. https://doi.org/10.1002/14651858.CD004371.pub3	Not latest version
Scranton RE, Goldstein I, Stecher VJ. Erectile dysfunction diagnosis and treatment as a means to improve medication adherence and optimize comorbidity management. *J Sex Med* 2013;**10**:551-61. https://doi.org/10.1111/j.1743-6109.2012.02998.x	Not SR
Seedat Y. Fixed drug combination in the treatment of hypertension and hyperlipidemia in the developing world. *Circulation* 2010;**122**:e94. http://dx.doi.org/10.1161/CIRCULATIONAHA.110.192773	Abstract
Selak V, Webster R, Stepien S, Bullen C, Patel A, Thom S*, et al.* Reaching cardiovascular prevention guideline targets with a polypill-based approach: a meta-analysis of randomised clinical trials. *Heart* 2019;**105**:42-8. https://doi.org/10.1136/heartjnl-2018-313108	Not SR
Shahaj O, Denneny D, Schwappach A, Pearce G, Epiphaniou E, Parke HL*, et al.* Supporting self-management for people with hypertension: a meta-review of quantitative and qualitative systematic reviews. *J Hypertens* 2019;**37**:264-79. https://doi.org/10.1097/HJH.0000000000001867	Full text not retrieved
Shaman AM, Smyth B, Arnott C, Palmer SC, Mihailidou AS, Jardine MJ*, et al.* Comparative Efficacy and Safety of BP-Lowering Pharmacotherapy in Patients Undergoing Maintenance Dialysis: A Network Meta-Analysis of Randomized, Controlled Trials. *Clin J Am Soc Nephrol* 2020;**15**:1129-38. https://doi.org/10.2215/CJN.12201019	Full text not retrieved
Sherrill B, Halpern M, Khan S, Zhang J, Panjabi S. Single-pill vs free-equivalent combination therapies for hypertension: a meta-analysis of health care costs and adherence. *J Clin Hypertens (Greenwich)* 2011;**13**:898-909. https://doi.org/10.1111/j.1751-7176.2011.00550.x	Not SR
Sommerauer C, Kaushik N, Woodham A, Renom-Guiteras A, Martinez YV, Reeves D*, et al.* Thiazides in the management of hypertension in older adults - a systematic review. *BMC Geriatr* 2017;**17**:228. https://doi.org/10.1186/s12877-017-0576-3	Quantitative focus

For peer review only

For peer review only - http://bmjopen.bmj.com/site/about/guidelines.xhtml

Sparrow RT, Khan AM, Ferreira-Legere LE, Ko DT, Jackevicius CA, Goodman SG, *et al.* Effectiveness of Interventions Aimed at Increasing Statin-Prescribing Rates in Primary Cardiovascular Disease Prevention: A Systematic Review of Randomized Clinical Trials. *JAMA Cardiol* 2019;**4**:1160-9. https://doi.org/10.1001/jamacardio.2019.3066	Full text not retrieved
Stergiou GS, Bliziotis IA. Home blood pressure monitoring in the diagnosis and treatment of hypertension: a systematic review. *Am J Hypertens* 2011;**24**:123-34. https://doi.org/10.1038/ajh.2010.194	Not SR
Sua YS, Jiang Y, Thompson DR, Wang W. Effectiveness of mobile phone-based self-management interventions for medication adherence and change in blood pressure in patients with coronary heart disease: A systematic review and meta-analysis. European journal of cardiovascular nursing 2020;19:192-200. https://doi.org/https://dx.doi.org/10.1177/1474515119895678	Medication NR
Thio SL, Nam J, van Driel ML, Dirven T, Blom JW. Effects of discontinuation of chronic medication in primary care: a systematic review of deprescribing trials. *Br J Gen Pract* 2018;**68**:e663-e72. https://doi.org/10.3399/bjgp18X699041	Quantitative focus
Thomopoulos C, Parati G, Zanchetti A. Effects of blood pressure lowering treatment in hypertension: 8. Outcome reductions vs. discontinuations because of adverse drug events - meta-analyses of randomized trials. *J Hypertens* 2016;**34**:1451-63. https://doi.org/10.1097/HJH.0000000000000972	Full text not retrieved
Thomopoulos C, Parati G, Zanchetti A. Effects of blood-pressure-lowering treatment in hypertension: 9. Discontinuations for adverse events attributed to different classes of antihypertensive drugs: meta-analyses of randomized trials. *J Hypertens* 2016;**34**:1921-32. https://doi.org/10.1097/HJH.0000000000001052	Full text not retrieved
Vassy JL, Chun S, Advani S, Ludin SA, Smith JG, Alligood EC. Impact of SLCO1B1 Pharmacogenetic Testing on Patient and Healthcare Outcomes: A Systematic Review. *Clin Pharmacol Ther* 2019;**106**:360-73. https://doi.org/10.1002/cpt.1223	Not SR
Vogele A, Johansson T, Renom-Guiteras A, Reeves D, Rieckert A, Schlender L, *et al.* Effectiveness and safety of beta blockers in the management of hypertension in older adults: a systematic review to help reduce inappropriate prescribing. *BMC Geriatr* 2017;**17**:224. https://doi.org/10.1186/s12877-017-0575-4	Quantitative focus
Webster R, Patel A, Selak V, Billot L, Bots ML, Brown A, *et al.* Effectiveness of fixed dose combination medication ('polypills') compared with usual care in patients with cardiovascular disease or at high risk: A prospective, individual patient data meta-analysis of 3140 patients in six countries. *Int J Cardiol* 2016;**205**:147-56. https://doi.org/10.1016/j.ijcard.2015.12.015	Not SR

For peer review only

For peer review only - http://bmjopen.bmj.com/site/about/guidelines.xhtml

Weeda ER, Coleman CI, McHorney CA, Crivera C, Schein JR, Sobieraj DM. Impact of once- or twice-daily dosing frequency on adherence to chronic cardiovascular disease medications: A meta-regression analysis. *Int J Cardiol* 2016;**216**:104-9. https://doi.org/10.1016/j.ijcard.2016.04.082	Not SR
Weernink MG, Vaanholt MC, Groothuis-Oudshoorn CG, Von Birgelen C, M.J IJ, Van Til JA. Patients' priorities for oral anticoagulation therapy in non-valvular atrial fibrillation. *Value in Health* 2017;**20**:A621. http://dx.doi.org/10.1016/j.jval.2017.08.1355	Abstract
Weisser B, Predel HG, Gillessen A, Hacke C, Vor dem Esche J, Rippin G*, et al.* Single Pill Regimen Leads to Better Adherence and Clinical Outcome in Daily Practice in Patients Suffering from Hypertension and/or Dyslipidemia: Results of a Meta-Analysis. *High Blood Press Cardiovasc Prev* 2020;**27**:157-64. https://doi.org/10.1007/s40292-020-00370-5	Not SR (Duplicate excluded in update search)
Wettermark B, Qvarnstrom M, Hasselstrom J, Kahan T. Controversies on patient persistence in antihypertensive drug treatment: A systematic review. *JRSM Cardiovascular Disease* 2013; http://dx.doi.org/10.1177/2048004013506452:6-7. http://dx.doi.org/10.1177/2048004013506452	Abstract
Xie CX, Chen Q, Hincapié CA, Hofstetter L, Maher CG, Machado GC, Effectiveness of clinical dashboards as audit and feedback or clinical decision support tools on medication use and test ordering: a systematic review of randomized controlled trials, *Journal of the American Medical Informatics Association*, Volume 29, Issue 10, October 2022, Pages 1773–1785, https://doi.org/10.1093/jamia/ocac094	Duplicte
Yan YL, Qiu B, Hu LJ, Jing XD, Liu YJ, Deng SB*, et al.* Efficacy and safety evaluation of intensive statin therapy in older patients with coronry heart disease: a systematic review and meta-analysis. *Eur J Clin Pharmacol* 2013;**69**:2001-9. https://doi.org/10.1007/s00228-013-1570-0	Quantitative focus
Zhao P, Xu P, Wan C, Wang Z. Evening versus morning dosing regimen drug therapy for hypertension. *Cochrane Database Syst Rev* 2011; 10.1002/14651858.CD004184.pub2:CD004184. https://doi.org/10.1002/14651858.CD004184.pub2	Quantitative focus
Zharkova T, Kyomen H. Management Strategies to Prevent Stroke in Elderly with Major Neurocognitive Disorder and Psychosis Treated with Antipsychotic Medication. *The American Journal of Geriatric Psychiatry* 2019;**27**:S135-S6. https://doi.org/10.1016/j.jagp.2019.01.042	Not SR

For peer review only - http://bmjopen.bmj.com/site/about/guidelines.xhtml

APPENDIX D: CHARACTERISTICS OF INCLUDED REVIEWS

Key for interpreting tables

Green highlighted text:	High overall quality as appraised by AMSTAR-2
Turquoise highlighted text:	Medium overall quality as appraised by AMSTAR-2
Orange highlighted text:	Low overall quality as appraised by AMSTAR-2
Red highlighted text:	Critically-low quality as appraised by the AMSTAR-2
CEESAT Set 1:	Prioritised for full quality appraisal using AMSTAR-2
CEESAT Set 2:	Scored poorly on 1 out of 4 items on CEESAT
CEESAT Set 3:	Scored poorly on 2-4 items on CEESAT

Reviews were judged to be of 'High' relevance if review focused on evaluating intervention/gathering experiences regarding medication of interest within health/social care services relevant to UK service setting, 'Medium' relevance: if review aim/inclusion criteria somewhat relevant to aim of systematic mapping review, but also includes other medications not of interest, or quantity of relevant information is limited, 'Low' relevance: if quantity of relevant information low and/or intervention being evaluated not relevant to UK health/social care setting

For peer review only - http://bmjopen.bmj.com/site/about/guidelines.xhtml

Table 3: Review overview

Focus/Aim	Study (First author, date)	Type of Review (design of studies included), synthesis methods	Eligible age of participants within studies included in the review	Medications of interest	Medical condition for which medication of interest being taken	Relevance of systematic review to aim of evidence-gap map	CEESAT set	Other comments
Evaluating intervention: enhance adherence	Adler, 2017	SR (Quantitative: RCTs), Narrative	NR<br>*Mean age where reported in included studies: 53.6 years to 64 years*	Statins<br>Antihypertensives	Secondary prevention CVD	Medium	1	
Evaluating intervention: enhance adherence	Al Alshaikh, 2016	SR(Quantitative: RCTs), MA+Narrative	18 years or over	Statins<br>Antihypertensives	Secondary prevention CVD	Medium	3	
Evaluating intervention: enhance adherence	Baumgartner, 2020	SR (Quantitative: RCTs, retrospective/prospective cohort, non-randomized, economic models, systematic reviews, meta-analysis), Narrative	NR<br>*Where reported, participants in included studies 18 years of age or over. Some studies specifically included adults 65 years or older*	Statins<br>Antihypertensives	HBP<br>Primary prevention CVD<br>Secondary prevention CVD	Medium	3	
Evaluating intervention: enhance adherence	Bochkareva, 2019	Scoping review (Quantitative: RCTs, non-RCTs, (cross-sectional, cohort studies), registers), Narrative Matrix/map of studies	18 years or over	Antihypertensives	HBP	Medium	3	Focus on Russia. Boad focus of scoping review, of which interventions to increase adherence are one part
Evaluating intervention: enhance adherence	Checchi, 2014	SR (Quantitative: RCTs, nonRCT, controlled, prospective/retrospective, observational, cohort), Narrative	NR<br>*Adults*	Antihypertensives<br>Statins	HBP<br>Primary prevention CVD	Medium	3	

For peer review only - http://bmjopen.bmj.com/site/about/guidelines.xhtml

Focus/Aim	Study (First author, date)	Type of Review (design of studies included), synthesis methods	Eligible age of participants within studies included in the review	Medications of interest	Medical condition for which medication of interest being taken	Relevance of systematic review to aim of evidence-gap map	CEESAT set	Other comments
Evaluating intervention: enhance adherence Secondary aim	Cheema, 2014	SR (Quantitative: RCTs, systematic reviews of RCTs), Meta-analysis+Narrative	18 years or over	Antihypertensives	HBP	High	2	
Evaluating intervention: optimise prescribing	Chhina, 2013	SR (Quantitative: RCT, observational studies with a control group), Narrative	NR<br>*1 included study focused on children, 3 focused on elderly*	Antihypertensives	HBP	Medium	3	One included study focused on children
Evaluating intervention: enhance adherence	Conn, 2015[70]	SR (Quantitative: NR), Meta-analysis	NR<br>*Median value for mean age accross included studies was 54.4 years*	Statins<br>Antihypertensives	HBP<br>Secondary prevention CVD	Medium	2	Medications of interest inferred from population disease characteristics
Evaluating intervention: enhance adherence	Conn, 2015[71]	SR (Quantitative: treatment-versus-control comparisons, pre-experimental studies), Exploratory analyses, Meta-analysis	NR<br>*Median mean participant age accross included studies was 60.70 years*	Statins<br>Antihypertensives	HBP<br>Primary prevention CVD<br>Secondary prevention CVD<br>Inferred from population disease characteristics	Medium	2	Medications of interest inferred from population disease characteristics
Evaluating intervention: enhance adherence	Conn, 2015[36]	SR (Quantitative: varied research methods were included), Meta-analysis	Other<br>*Adults*	Antihypertensives	HBP	High	3	

For peer review only - http://bmjopen.bmj.com/site/about/guidelines.xhtml

Focus/Aim	Study (First author, date)	Type of Review (design of studies included), synthesis methods	Eligible age of participants within studies included in the review	Medications of interest	Medical condition for which medication of interest being taken	Relevance of systematic review to aim of evidence-gap map	CEESAT set	Other comments
Evaluating intervention: enhance adherence	Bond, 2021	SR (Quantitative: RCTs), Narrative	Any age	Statins	Primary and Secondary prevention CVC	High	3	
Evaluating intervention: deprescribing	Chambergo-Michilot, 2023	SR (Quantitative: observational studies, trials, RCT, cohort), Meta-analysis	NR: *In majority of studies, mean/median age was higher than 60 years*	Antihypertensives	HBP	High	3	
Evaluating intervention: enhance adherence	Conn, 2016	SR (Quantitative: RCTs, non-RCTs, Pre-experimental before and after), Narrative+Meta-analysis	NR<br>*Adults. Median mean age accross included studies 53 years*	Statins<br>Antihypertensives	Primary prevention CVD<br>Depression<br>HBP<br>Secondary prevention CVD	Medium	2	Medications of interest inferred from population disease characteristic
Evaluating intervention: enhance adherence/optimise prescribing	Coronado-Vazquez, 2020	SR (Quantitative: RCT, CT), Narrative	NR *mean age of the participants in the reviewed studies ranged from 8 years[32] to 73 years*	Statins<br>Antihypertensives	Primary and secondary prevention CVD	Low	2	
Evaluating intervention: enhance adherence secondary aim	de Cates, 2014	SR (Quantitative: RCTs), Meta-analysis	18 years or over	Statins<br>Antihypertensives	Primary prevention CVD<br>Secondary prevention CVD	Medium	1	Adherence only secondary outcome
Evaluating intervention: enhance adherence	De Simoni, 2013	Quantitative (RCTs), Narrative+Meta-analysis	Other<br>*Adults*	Antihypertensives	HBP<br>Secondary prevention CVD	High	2	

For peer review only - http://bmjopen.bmj.com/site/about/guidelines.xhtml

Focus/Aim	Study (First author, date)	Type of Review (design of studies included), synthesis methods	Eligible age of participants within studies included in the review	Medications of interest	Medical condition for which medication of interest being taken	Relevance of systematic review to aim of evidence-gap map	CEESAT set	Other comments
Evaluating intervention: enhance adherence	Deichmann. 2016	SR (Quantitative: RCTs, parallel group or crossover, individual or cluster randomization), Meta-analysis+Narrative	18 years or over	Statins	Primary prevention CVD Secondary prevention CVD	Medium	3	
Evaluating intervention: enhance adherence	Demonceau, 2013	SR (Quantitative: RCT, cross-over and cluster-randomized), Meta-analysis+meta-regression	Other *Average age expressed in years was 47.5 [range (3.0; 73.7)] in the usual care group and 46.8 [range (3.4; 76.2)] in the intervention group* *Adults and children*	Antihypertensives *Assumed*	HBP NR	Medium	3	Included methadone clinic patients (n=1). Included children
Patient views: adherence to medication of interest Only these of interest	Dhar, 2017	SR (Mixed: only quali studies met focus of review. Quantitative, descriptive-co relational study, cross-sectional, focus group interviews, survey, interviews), Narrative	18 years or over	Antihypertensives	HBP	Low	3	Focus on undeveloped countries. Only 5 included qualitative studies

For peer review only - http://bmjopen.bmj.com/site/about/guidelines.xhtml

Focus/Aim	Study (First author, date)	Type of Review (design of studies included), synthesis methods	Eligible age of participants within studies included in the review	Medications of interest	Medical condition for which medication of interest being taken	Relevance of systematic review to aim of evidence-gap map	CEESAT set	Other comments
Evaluating intervention: deprescribing	Dills, 2018	SR (Quantitative: Dills-2019-Deprescribing Medications for Chronic.pdf: Page 1: "Randomized Controlled Trials" Narrative	18 years or over	Statins Antihypertensives	HBP Secondary prevention CVD Other Illnesses associated with use of: alendronate, PPI, H2 antagonist	Medium	1	Palliative care studies where life expectancy is > 6 months included
Evaluating intervention: enhance adherence	Du, 2018	SR (Quantitative: parallel comparison studies, retrospective cohort, prospective), Meta-analysis	NR *Mean age of included studies (where reported) ranged from 53-76 years*	Antihypertensives	HBP	High	3	
Evaluating intervention: enhance adherence	Fletcher, 2015	SR (Quantitative: Randomized and quasi-randomized trials), Narrative+Meta-analysis	NR *Where reported, participants mean age ranged from 38 years to 77 accross included studies*	Antihypertensives	HBP	High	1	
Evaluating intervention: enhance adherence	Gupta, 2010	SR (Quantitative: RCT, clinical trial and prospective or retrospective cohort), Narrative+Meta-analysis	Other *18 to 79 years*	Antihypertensives	HBP	High	3	
Evaluating intervention: enhance adherence	Guzman-Tordecilla, 2020	SR (Quantitative: Guzman-Tordecilla-2020-InterventionsToIncreaseThePhar.pdf: Page 5: "Quasi-experimental" "Experimental" Narrative	17 years or over	Antihypertensives	HBP	Low	3	Studies conductedi n Latin America

For peer review only - http://bmjopen.bmj.com/site/about/guidelines.xhtml

Focus/Aim	Study (First author, date)	Type of Review (design of studies included), synthesis methods	Eligible age of participants within studies included in the review	Medications of interest	Medical condition for which medication of interest being taken	Relevance of systematic review to aim of evidence-gap map	CEESAT set	Other comments
Evaluating intervention: deprescribing Secondary aim, not actually explicit. Look at interventions to reduce Fall Risk-Inducing Drugs (FRID) in an attempt to understand whether this reduced falls.	Hart, 2020	SR (Quantitative: observational or intervention (eg, RCTs and quasi-experimental design)), Narrative	Other *60 or over*	Antihypertensives	NR	Medium	3	Lots of other drugs. Also approach reduction of drugs of interest in a round-about way, rather than directly wanting to reduce them
Evaluating intervention: enhance adherence	Hong, 2022	SR (Quantitative: RCT, cross-sectional), Narrative	NR *Only adults were recruited*	Antihypertensives	HBP Secondary prevention CVD	High	3	
Evaluating intervention: optimise prescribing	Hukins, 2019	SR (Quantitative: observational, non-randomised evaluation), Narrative	Other *People with a diagnosis of/suspected dementia*	Statins	NR	Medium	1	

Focus/Aim	Study (First author, date)	Type of Review (design of studies included), synthesis methods	Eligible age of participants within studies included in the review	Medications of interest	Medical condition for which medication of interest being taken	Relevance of systematic review to aim of evidence-gap map	CEESAT set	Other comments
Patient views: adherence to medication of interest<br>Patient views: intervention to enhance adherence	Ingersgaard, 2020	Review of SR (Other, all types of systematic reviews), Narrative	NR	Statins<br>Antihypertensives	Primary prevention CVD<br>Secondary prevention CVD	Medium	2	Review of reviews, little relevant qualitative evidence
Evaluating intervention: optimise prescribing	Jeffery, 2015	SR (Quantitative: RCTs, cluster and nexted), Meta-analysis+Narrative	NA<br>*Targeted prescribers*	Statins<br>Antihypertensives	Primary prevention CVD<br>Secondary prevention CVD	High	1	
Evaluating intervention: optimise prescribing	Kang, 2016	SR (Quantitative: RCTs, non-RCT, prospective observational studies), Narrative+Meta-analysis	18 years or over	Statins<br>Antihypertensives	Secondary prevention CVD	High	3	
Evaluating intervention: enhance adherence	Kassavou, 2018	SR (Quantitative: RCTs, randomisation at individual patient or cluster level), Meta-analysis+meta-regression	18 years or over	Statins<br>Antihypertensives	HBP<br>Primary prevention CVD<br>Secondary prevention CVD<br>Other Cardio-metabolic conditions, high cholesterol	High	2	
Evaluating intervention: enhance adherence	Kawalec, 2018	SR (Quantitative: No restrictions were applied as to the study design. SRs, cohort, non-RCT, prospective observational study), Narrative+Meta-analysis	NR<br>*Where reported, all included studies included participants of 18 years or older*	Antihypertensives	HBP	High	3	

For peer review only - http://bmjopen.bmj.com/site/about/guidelines.xhtml

Focus/Aim	Study (First author, date)	Type of Review (design of studies included), synthesis methods	Eligible age of participants within studies included in the review	Medications of interest	Medical condition for which medication of interest being taken	Relevance of systematic review to aim of evidence-gap map	CEESAT set	Other comments
Practitioner views: prescribing medication of interest<br>Patient views: adherence to medication of interest	Khatib, 2014	SR (Mixed: Qualitative and quantitative observational studies. Included RCT, cohort, case-control), Framework/Content analysis, Narrative	Other<br>*Any age*	Antihypertensives	HBP	High	2	Methods of qualitative synthesis unclear
Evaluating intervention: optimise prescribing<br>Think this is most appropriate overall, but could justify selecting more than one?<br>Evaluating intervention: enhance adherence	King, 2018	SR (Quantitative: RCT, observational), Narrative	NR	Statins<br>Antihypertensives	HBP<br>Primary prevention CVD	Medium	2	
Patient views: adherence to medication of interest<br>Family/carer views: adherence to medication of interest	Kinnear, 2019	SR (Qualitative: face to face/telephone/online interviews, focus groups) Thematic	NR<br>*264 individuals with FH and 13 family members were involved, aged 8-69 years*	Statins	Primary prevention CVD<br>Secondary prevention CVD	Medium	1	N=7 papers reported findings from samples including individuals under 18 years. N=4 papers reported parental views of having children with FH
Evaluating intervention: deprescribing	Kornholt, 2022	Scoping review (Quantitative: RCT), Narrative/descriptive statistics	65 years or over	Statins<br>Antihypertensives	HBP<br>Primary and Secondary CVD	Medium	2	

For peer review only - http://bmjopen.bmj.com/site/about/guidelines.xhtml

Focus/Aim	Study (First author, date)	Type of Review (design of studies included), synthesis methods	Eligible age of participants within studies included in the review	Medications of interest	Medical condition for which medication of interest being taken	Relevance of systematic review to aim of evidence-gap map	CEESAT set	Other comments
Evaluating intervention: enhance adherence	Krack, 2019	SR (Quantitative: RCTs, non-RCTs), Narrative+Meta-analysis	NR	Statins Antihypertensives	HBP Primary prevention CVD Secondary prevention CVD	Low	2	
Evaluating intervention: enhance adherence	Kronish, 2011	SR (Quantitative: observational cohorts), Meta-analysis	18 years or over	Antihypertensives	HBP	High	2	
Evaluating intervention: enhance adherence Evaluating intervention: optimise prescribing Because of interventions directed at HCPs	Laba, 2013	SR (Quantitative: Randomised/quasi-randomised controlled trials), Narrative	Other *Adults*	Statins Antihypertensives	HBP Primary prevention CVD *Possibly secondary prevention, unclear*	Medium	3	
Evaluating intervention: enhance adherence	Lawrence, 2015	SR (Quantitative: RCTs), Meta-analysis	18 years or over	Statins Antihypertensives	Secondary prevention CVD *Prevention of secondary stroke and/or TIA*	Medium	2	Only 3 studies reporting adherence outcomes for each of statins and antihypertensives
Evaluating intervention: optimise prescribing Secondary aim	Legare, 2010	SR (Quantitative: RCTs, well designed quasi-experimental studies (controlled clinical trials, CBA, ITS), Narrative	NR	Statins	NR	Low	1	Possibly only one study (of 5) about statins, the others are not relevant

For peer review only - http://bmjopen.bmj.com/site/about/guidelines.xhtml

Focus/Aim	Study (First author, date)	Type of Review (design of studies included), synthesis methods	Eligible age of participants within studies included in the review	Medications of interest	Medical condition for which medication of interest being taken	Relevance of systematic review to aim of evidence-gap map	CEESAT set	Other comments
Evaluating intervention: enhance adherence	Leslie, 2016	SR (Quantitative: RCTs), Narrative	65 years or over *or consisted of Medicare beneficiaries*	Antihypertensives Statins	HBP Primary prevention CVD	Medium	3	Focus on elderly. Medications of interest assumed from population characteristics
Evaluating intervention: optimise prescribing	Loganathan, 2011	SR (Quantitative: RCTs, non-RCTs), Narrative	65 years or over	Antihypertensives	NR	Medium	3	Only a couple of included studies with medications of interest
Evaluating intervention: enhance adherence Secondary aim	Maimaris, 2013	SR (Quantitative: RCTs, controlled trials, cohort, cross-sectional, case-control, cross-sectional, ecological), Narrative	NR	Antihypertensives	HBP	Medium	1	Limited information relating to adherence of hypertensives. Uncertain as to relevance of findings to UK health and social care setting
Evaluating intervention: enhance adherence Seconardy aim	Mallat, 2016	SR (Quantitative: RCTs), Meta-analysis	18 years or over	Antihypertensives	HBP	High	1	
Evaluating intervention: enhance adherence	Mamudu, 2014	SR (Quantitative: RCTs, non-RCTs), Narrative	NR	Statins Antihypertensives	Primary prevention CVD	Medium	3	
Evaluating intervention: enhance adherence	Mann, 2014	SR (Quantitative: RCTs, ITS, CBA), Narrative	Other *Adult patients*	Statins Antihypertensives	HBP Primary prevention CVD Secondary prevention CVD	Low	2	One included study included participants aged 14 and above. Intervention not relevant to UK setting

For peer review only - http://bmjopen.bmj.com/site/about/guidelines.xhtml

Focus/Aim	Study (First author, date)	Type of Review (design of studies included), synthesis methods	Eligible age of participants within studies included in the review	Medications of interest	Medical condition for which medication of interest being taken	Relevance of systematic review to aim of evidence-gap map	CEESAT set	Other comments
Patient views: adherence to medication of interest<br>Qualitative: other<br>Understanding and experiences of hypertension and drug adherence	Marshall, 2012	SR (Qualitative: Interviews, focus groups), Narrative/Thematic	NR<br>*Where reported, age of population within included studies ranged from 18-92 years*	Antihypertensives	HBP	High	3	
Evaluating intervention: enhance adherence	Milosavljevic, 2018	SR (Quantitative: RCTs, quasi-controlled trials, cluster-controlled trials, BA, retrospective/prospective cohort, observational, single group pre/post comparisons, statistical predictions), Narrative	NR	Antihypertensives	HBP<br>Depression	Medium	2	
Evaluating intervention: enhance adherence	Morrissey, 2017	SR (Quantitative: RCTs), Narrative Meta-analysis	NR<br>*Where reported, mean age ranged from 51 to 76 accross included studies*	Antihypertensives	HBP	High	1	
Evaluating intervention: optimise prescribing	Nguyen, 2018	SR (Quantitative: RCTs), Meta-analysis	NR	Statins	Secondary prevention CVD<br>*Ischaemic heart disease*	Medium	2	
Evaluating intervention: enhance adherence	Nieuwlaat, 2014	SR (Quantitative: RCTs), Narrative	NR<br>*Included studies characteristics table indicates both adult and child populations included*	Statins<br>Antihypertensives	HBP<br>Primary prevention CVD<br>Secondary prevention CVD	Medium	1	

For peer review only - http://bmjopen.bmj.com/site/about/guidelines.xhtml

Focus/Aim	Study (First author, date)	Type of Review (design of studies included), synthesis methods	Eligible age of participants within studies included in the review	Medications of interest	Medical condition for which medication of interest being taken	Relevance of systematic review to aim of evidence-gap map	CEESAT set	Other comments
Evaluating intervention: enhance adherence	Nili, 2020	SR (Quantitative: RCT), Narrative	Adults	Antihypertensives	HBP	High	1	
Qualitative: other Factors which predict medication adherence. Not a qualitative review but didn't know what else to select What factors are associated with/influence or predict ad- herence of anti-hypertensive medication in older adults with HBP?, How are their causal relationship and their influence on MA in older adults with HBP based on ecological ap-proach?	Oori, 2019	Integrative review (Mixed: quantitative and qualitative designs, including: cross-sectional and descriptive, interventional, cohort and two descriptive qualitative), Content analysis	Older adults	Antihypertensives	HBP	Low	3	A qualitative synthesis of quant/qual data. Included because of qualitative aspect, but only 'descriptive qualitative' studies were included

For peer review only - http://bmjopen.bmj.com/site/about/guidelines.xhtml

Focus/Aim	Study (First author, date)	Type of Review (design of studies included), synthesis methods	Eligible age of participants within studies included in the review	Medications of interest	Medical condition for which medication of interest being taken	Relevance of systematic review to aim of evidence-gap map	CEESAT set	Other comments
Evaluating intervention: deprescribing	Page, 2016	SR (Quantitative: RCTs, quasi-randomized controlled studies, non-RCTs, prospective and retrospective cohort, case-control, single arm, BA), Meta-analysis	65 years or over	Statins Antihypertensives	HBP Primary prevention CVD Secondary prevention CVD	Medium	2	
Evaluating intervention: enhance adherence	Palmer, 2018	SR (Quantitative: RCTs of parallel group design that randomised by participant or by cluster), Narrative	18 years or over	Statins Antihypertensives	Primary prevention CVD	Medium	1	
Evaluating intervention: enhance adherence	Palmer, 2020 (update 2018)	SR (Quantitative: RCT), Meta-analysis+Narrative	18 years or over	Statins Antihypertensives	Primary prevention CVD	Medium	1	
Evaluating intervention: optimise prescribing One of a variety of aims	Pantoja, 2019	SR (Quantitative: RCTs, non-RCTs), Narrative	NR	Statins Antihypertensives	HBP Primary prevention CVD *Unclear if statin for primary or secondary prevention*	Medium	1	Some included studies included child participants. Low amount of information about prescribing drugs of interest. intervention is more of a general aid

For peer review only - http://bmjopen.bmj.com/site/about/guidelines.xhtml

Focus/Aim	Study (First author, date)	Type of Review (design of studies included), synthesis methods	Eligible age of participants within studies included in the review	Medications of interest	Medical condition for which medication of interest being taken	Relevance of systematic review to aim of evidence-gap map	CEESAT set	Other comments
Evaluating intervention: enhance adherence	Parati, 2021	SR (Quantitative: Retrospective data- base analysis, prospective, open- label, non-randomized, 2 phased, International, randomized, open-label, controlled, 12-wk phase III trial, multi-center, observational study, retrospective database analysis, retrospective cohort study, non-RCT, observational, randomized, prospective, multi-center trial, interventional parallel group, interventional study), Narrative+Meta-analysis	18 years or over	Antihypertensives	HBP	High	2	
Evaluating intervention: enhance adherence	Park, 2017	SR (Quantitative: Original research, comparison group, pre/post comparisons, statistical predictions), Narrative	NR	Antihypertensives Statins	HBP Primary prevention CVD NR	Low	3	Not relevant to UK, medications not of interest included
Evaluating intervention: enhance adherence Note that focus is on 'use' which includes initiating prescribing. Drugs of interest may be currently under- or -ver-used	Polinski, 2011	SR (Quantitative: Polinski-2011-Medicare Part D's Effect on the.pdf: Page 3: "Articles were included if they reported original results regarding Part D drug utilization for specific drugs or drug classes, whether drawn from self-report surveys or prescription drug claims" Narrative	NR	Statins Antihypertensives	HBP Primary prevention CVD NR *Not clearly reported*	Low	3	Low relevance to UK. Other medications not of interest included

For peer review only

Focus/Aim	Study (First author, date)	Type of Review (design of studies included), synthesis methods	Eligible age of participants within studies included in the review	Medications of interest	Medical condition for which medication of interest being taken	Relevance of systematic review to aim of evidence-gap map	CEESAT set	Other comments
Evaluating intervention: enhance adherence Seconary aim: interventions to affect behaviour change that leads to prevention of chronic disease - e.g. taking antihypertensives...	Posadzki, 2016	SR (Quantitative: RCTs, cluster RCTs, quasi-RCTs, ITS, CBA), Narrative+Meta-analysis	NR	Statins Antihypertensives	HBP Primary prevention CVD	Medium	1	Included children. Interventions target general positive behaviour change in terms of health management. However there are specific studies reporting adherence-type interventions. Several non-relevant drugs and outcomes within the review.
Prescriber, patient, and/or family/carer views of issues relating to prescribing and/or adherence to medications of interest	Qadi, 2020	SR(Qualitative) Thematic	NR *Excludes studies initiating medication in older age group*	Statins Antihypertensives	Primary prevention CVD	High	2	
Evaluating intervention: enhance adherence	Rash, 2016	SR (Quantitative: RCTs), Narrative	NR	Statins	Primary prevention CVD Secondary prevention CVD	High	2	
Patient views: adherence to medication of interest	Rashid, 2014	SR (Qualitative: Focus groups, interviews, interviews and questionnaires), Meta-ethnography	Other *Adults*	Statins	Secondary prevention CVD	Medium	3	

For peer review only - http://bmjopen.bmj.com/site/about/guidelines.xhtml

Focus/Aim	Study (First author, date)	Type of Review (design of studies included), synthesis methods	Eligible age of participants within studies included in the review	Medications of interest	Medical condition for which medication of interest being taken	Relevance of systematic review to aim of evidence-gap map	CEESAT set	Other comments
Qualitative: other Rashid 2018.pdf: Page 1: "explored medication-taking experiences in patients, citizens, carers, relatives and clinicians."	Rashid, 2018	SR (Qualitative: All types of qualitative studies, including those linked to observational, experimental, mixed methods papers. Interviews, focus groups or a combination), Thematic	Other	Antihypertensives	HBP	Medium	3	Includes children and adults. Other medications not of interest included
Patient views: intervention to encourage deprescribing Family/carer views: intervention to encourage deprescribing	Reeve, 2013	SR (Mixed: No limits placed on the type of methods used, qualitative, quantitative or mixed), Narrative/Content analysis/Thematic	Other *No limits were placed on the age of the participants*	Antihypertensives	HBP Anxiety NR	Medium	3	Methods of synthesis unclear
Evaluating intervention: enhance adherence	Reeves, 2020	SR (Quantitative: RCTs), Narrative	18 years or over *Adult*	Antihypertensives	HBP	High	2	
Evaluating intervention: enhance adherence	Reston, 2020	SR (Quantitative: SR, RCT, observational), Narrative	18 years or over	Statins	Primary prevention CVD	High	2	
Evaluating intervention: enhance adherence One of multiple aims	Sanders, 2011	SR (Quantitative: comparative clinical studies of any design, including RCTs, non-RCTs, retrospective/prospective cohort, and case-control), Narrative+Meta-analysis	18 years or over	Antihypertensives	HBP *Essential hypertension*	Medium	1	Adherence low on the list of outcomes

For peer review only - http://bmjopen.bmj.com/site/about/guidelines.xhtml

Focus/Aim	Study (First author, date)	Type of Review (design of studies included), synthesis methods	Eligible age of participants within studies included in the review	Medications of interest	Medical condition for which medication of interest being taken	Relevance of systematic review to aim of evidence-gap map	CEESAT set	Other comments
Evaluating intervention: optimise prescribing	Shanbhag, 2018	SR (Quantitative: RCTs, cohort (with comparisons), controlled and uncontrolled BA, ITS), Narrative	NR	Antihypertensives	HBP *Associated with heart failure*	High	1	
Evaluating intervention: enhance adherence	Stacey, 2017	SR (Quantitative: RCTs), Meta-analysis	18 years or over	Antihypertensives	HBP Primary prevention CVD Seconday prevention CVD	Medium	1	Study focus: RE: making decisions about screening or treatment options for themselves, a child, or an incapacitated significant other
Evaluating intervention: enhance adherence	Tsioufis, 2020	Rapid review (Quantitative: RCTs, observational studies), Narrative	Other *Adults. Where reported, mean age of populations in included studies ranged from 47 to 71.5 years*	Antihypertensives	HBP	High	3	
Evaluating intervention: enhance adherence	Uhlig, 2013	SR (Quantitative: prospective comparative), Narrative+Meta-analysis	Other *Adults*	Antihypertensives	HBP	High	3	
Evaluating intervention: enhance adherence	van Driel, 2016	SR (Quantitative: RCTs of parallel-group or cross-over design, that used individual or cluster randomisation), Narrative+Meta-analysis	18 years or over	Statins	Primary prevention CVD Seconday prevention CVD	High	1	
Evaluating intervention: enhance adherence	Van Truong, 2021	SR (Quantitative: RCT), Meta-analysis	Other: 60 years or above	Antihypertensives (assumed)	HBP	Medium	2	

For peer review only - http://bmjopen.bmj.com/site/about/guidelines.xhtml

Focus/Aim	Study (First author, date)	Type of Review (design of studies included), synthesis methods	Eligible age of participants within studies included in the review	Medications of interest	Medical condition for which medication of interest being taken	Relevance of systematic review to aim of evidence-gap map	CEESAT set	Other comments
Evaluating intervention: enhance adherence	Verveloet, 2020	SR (Quant: RCT, controlled CT, CT), Narrative	NR	Statins Antihypertensives	HBP	High	2	
Evaluating intervention: enhance adherence and prescription	Xie,2022	SR (Quantitative: RCT), Narrative	NR	Statin Antihypertensives	HBP, Other: atherosclerotic CVD	Low	2	
Evaluating intervention: enhance adherence	Xiong, 2018	SR (Quantitative: RCTs, non-RCTs, before-and-after studies without a control group), Narrative	NR *All studies involved adults aged from 40 to 69*	Antihypertensives	HBP	High	3	
Evaluating intervention: enhance adherence	Xu, 2018	SR (Quantitative: RCTs), Meta-analysis	18 years or over	Antihypertensives	HBP	Medium	3	Focus on Chinese patients
Evaluating intervention: optimise prescribing Secondary aim Evaluating intervention: enhance adherence Secondary aim	Yuan, 2019	SR (Quantitative: RCTs, high quality cohort studies), Narrative+Meta-analysis	NR	Antihypertensives	HBP Primary prevention CVD Seconday prevention CVD *Assumed from content of paper*	Medium	3	

For peer review only - http://bmjopen.bmj.com/site/about/guidelines.xhtml

Focus/Aim	Study (First author, date)	Type of Review (design of studies included), synthesis methods	Eligible age of participants within studies included in the review	Medications of interest	Medical condition for which medication of interest being taken	Relevance of systematic review to aim of evidence-gap map	CEESAT set	Other comments
Evaluate intervention: optimise prescribing. Guidelines	Zheng, 2023	SR (Quantitative: RCT, non-randomized observational), meta-analysis	Adults	Antihypertensives	HBP	High	1	

Green highlighted text=High overall quality as appraised by AMSTAR-2, Turquoise highlighted text=Medium overall quality as appraised by AMSTAR-2, Orange highlighted text=Low overall quality as appraised by AMSTAR-2, Red highlighted text=Critically-low quality as appraised by the AMSTAR-2. *CEESAT Set: 1=prioritised for full quality appraisal using AMSTAR-2, CEESAT Set 2=Scored poorly on 1 out of 4 items on CEESAT, CEESAT Set 3=Scored poorly on 2-4 items on CEESAT. BA=Before and After, BPD=Borderline Personality Disorder, CBA=Controlled Before and After, CPG=Clinical Practice Guidelines, CT=Controlled-Trials, CVD=Cardiovascular Disease, FH=Familial Hypercholesterolemia, HPP=High Blood Pressure, ITS=Interrupted Time Series, MA=Meta-analysis, NR=Not Reported, PPI=Proton Pump Inhibitors, PTSD=Post-Traumatic Stress Disorder, RCT=Randomized Controlled Trial, RQ=Research Question, SR=Systematic Review

For peer review only - http://bmjopen.bmj.com/site/about/guidelines.xhtml

Table 4: Systematic reviews appraised using AMSTAR-2 which synthesised studies evaluating the effectiveness of an intervention

Focus/ Aim	Study (First author, date)	Medications of interest	Other medications included	Intervention name/s and aim	Outcomes measured	Setting/Context	Relevant part of care pathway
Evaluating intervention: deprescribing	Dills, 2018	Statins Antihypertensives	Diuretics, salmeterol, insulin, sulfonylurea, antipsychotics, anxiolytic, CV drugs, levodopa, nitrate, benzodiazepines Z-drugs Antidepressants	Deprescription: reduce medication burden, and enhance control of chronic medical and mental health conditions commonly managed by primary care physicians, compared with standard care in the non-terminally ill adult population	Primary outcome: successful deprescription, Secondary outcome: AE related to drug or underlying chronic condition as a result of deprescription	Primary/health /social care Hospital (inpatient) Hospital (outpatient) Care home Patient home	Decision to stop treatment: supported
Evaluating intervention: enhance adherence	Stacey, 2017	Antihypertensives	Hepatitis B vaccination, schizophrenia medication, immunotherapy for multiple sclerosis, vaccines (measles, mumps, rubella), radioactive iodine treatment for thyroid cancer, antibiotic use for upper respiratory infections, chemotherapy for cancer, medications for diabetes, hormone therapy, "breast cancer prevention medication, bisphosphonates, thyroid cancer radioactive iodine treatment	Decision aids for people facing health treatment or screening decisions.<br>Aim: help people make specific and deliberated choices among options, by making the decision explicit/by providing information on the options and outcomes relevant to person's health status as well as implicit methods to clarify values	Attributes of choice made: does patient decision aid improve match between chosen option and features that matter most to informed patient (demonstrated by knowledge, accurate risk perceptions, values-choice congruence).<br><br>Attributes of decision-making process: does patient decision aid help patients to recognize decision needs to be made, feel informed about options and their features, be clear about option features that matter most, discuss values with their clinician, and become involved in decision making? Other decision-making process variables: Decisional conflict, Patient-clinician communication, participation in decision making, proportion undecided, satisfaction with the choice/process of decision making/preparation for decision	NR	Information and/or advice Shared decision making Patient adherence

Focus/ Aim	Study (First author, date)	Medications of interest	Other medications included	Intervention name/s and aim	Outcomes measured	Setting/Context	Relevant part of care pathway
					making. Secondary outcomes: Choice (the actual choice implemented; if not reported, the participants preferred option), adherence to chosen option, health status and quality of life (generic and condition-specific), anxiety, depression, emotional distress, regret, confidence, costs, cost-effectiveness, consultation length, litigation rates		

For peer review only - http://bmjopen.bmj.com/site/about/guidelines.xhtml

Focus/ Aim	Study (First author, date)	Medications of interest	Other medications included	Intervention name/s and aim	Outcomes measured	Setting/Context	Relevant part of care pathway
Evaluating intervention: enhance adherence*	Posadzki 2016	Statins<br>Antihypertensives	Asthma medication, prescription drugs taken without prescription or more than prescribed, illicit non-injection drugs, glaucoma medications, inhaled corticosteroids, insulin, methadone maintenance, cholinesterase inhibitor medications, oral hypoglycaemic medications, nicotine replacement therapy, bupropion, and varenicline, antiretroviral treatment, bone active medication, non-hormonal oral agents, opiates antidepressants	Automated telephone communication systems for preventing disease and managing long-term conditions	Health behaviour and clinical outcomes, changes in health-enhancing behaviour, risk-taking behaviour, physiological measures, blood biochemistry. Process outcomes: change in acceptability of service (e.g. consumer accessibility/usability of interventions to apply information/support supplied), satisfaction (e.g. patient/carer) the intervention), cost-effectiveness. Cognitive outcomes: changes in knowledge (i.e. accurate risk knowledge and perception), attitude and intention to change, self-efficacy. Patient-centred outcomes: quality of life, adverse outcomes, unintended AE attributable to intervention	Primary/health/ social care<br>Secondary health/social care (not hospital)<br>Hospital (inpatient)<br>Hospital (outpatient)<br>Patient home	Identifying health or prescribing concern<br>Accessing care<br>Choosing a medication<br>Decision to start agreed treatment plan<br>Patient adherence<br>Maintaining treatment
Evaluating intervention: enhance adherence	Adler, 2017	Statins<br>Antihypertensives	Antiplatelet	Mobile phone text messaging to enhance adherence to recommended medication in patients with established arterial occlusive events	Adherence to treatment, fatal/non-fatal cardiovascular events, combined CVD event, Surrogate outcomes according to the different interventions recommended for secondary prevention including: low-density lipoprotein-cholesterol, blood pressure, heart rate, urinary 11-dehydrothromboxane B2, AEs	Primary/health /social care<br>Hospital (outpatient)	Patient adherence

For peer review only - http://bmjopen.bmj.com/site/about/guidelines.xhtml

Focus/ Aim	Study (First author, date)	Medications of interest	Other medications included	Intervention name/s and aim	Outcomes measured	Setting/Context	Relevant part of care pathway
Evaluating intervention: enhance adherence	Fletcher, 2015	Antihypertensives	NA	Self-monitoring of blood pressure: improve medication adherence, medication persistence, and lifestyle factors in people with hypertension	Primary outcomes: antihypertensive medication adherence and persistence, dietary outcomes, alcohol consumption and physical activity. Secondary outcomes: BP, BP control, and adherence to SMBP component of interventions	Primary/health/ social care<br>Hospital (outpatient)	Decision to start agreed treatment plan<br>Patient adherence<br>Progress review
Evaluating intervention: enhance adherence	Morrissey, 2017	Antihypertensives	NA	Interventions to enhance medication adherence and blood pressure control in hypertension	Change in BP, medication adherence	Primary/health/ social care<br>Hospital (inpatient)<br>Hospital (outpatient)<br>Care home<br>Patient home	Patient adherence
Evaluating intervention: enhance adherence	Nieuwlaat, 2014	Antidepressants<br>Statins<br>Antihypertensives	Medications to treat following conditions: HIV/AIDS, psychiatric disorders, chronic obstructive pulmonary disease, CVD/CV risk, diabetes, antibiotics, arthritis, complex chronic care, dyspepsia, glaucoma, oral anticoagulation, osteoporosis, tuberculosis, acne, cancer, hepatitis, iron supplementation during pregnancy, liver transplant, malaria, oral contraceptives,	Interventions to affect adherence with prescribed, self-administered medications	Medication adherence, clinical outcomes	Primary/health/ social care<br>Hospital (inpatient)<br>Hospital (outpatient)<br>Care home<br>Patient home	Information and/or advice<br>Patient adherence<br>Progress review

For peer review only - http://bmjopen.bmj.com/site/about/guidelines.xhtml

For peer review only

Focus/ Aim	Study (First author, date)	Medications of interest	Other medications included	Intervention name/s and aim	Outcomes measured	Setting/Context	Relevant part of care pathway
			antidepressants tonsillectomy/adenoidectomy, ulcerative colitis				
Evaluating intervention: enhance adherence	Palmer, 2018	Statins Antihypertensives	Antiplatelet drugs (low-dose aspirin, non-aspirin antiplatelet drugs)	Mobile phone-based interventions to improve adherence to medication prescribed for the primary prevention of cardiovascular disease in adults	Objective measures of adherence to treatment, combined CVD events, AE, indirect measures of adherence to treatment, health-related quality of life, cognitive outcomes (satisfaction with treatment, medication-taking self-efficacy, autonomy related to medication, attitudes (e.g. concerns about medicine AEs)), costs, process measures: extent of intervention received and acceptability of intervention	Primary/health/ social care Secondary health/social care (not hospital) Hospital (outpatient) Patient home	Progress review Patient adherence
Evaluating intervention: enhance adherence	Palmer, 2020	See above	See above	See above	See above	See above	See above

For peer review only - http://bmjopen.bmj.com/site/about/guidelines.xhtml

Focus/ Aim	Study (First author, date)	Medications of interest	Other medications included	Intervention name/s and aim	Outcomes measured	Setting/Context	Relevant part of care pathway
Evaluating intervention: enhance adherence	van Driel, 2016	Statins	Any lipid-lowering medication	Interventions to improve adherence to lipid-lowering drugs: 1. Simplification of drug regimen; 2. Patient education and information; 3. Intensified patient care; 4. Complex behavioural approaches; 5. Decision support systems; 6. Administrative improvements; and 7. Pharmacy-led interventions	Indirect measures of adherence, subjective measures of adherence, direct measures of adherence, physiological indicators, health outcome indications (e.g. quality of life, morbidity, mortality), AEs, implications for costs (impact of intervention on economic outcomes, economic evaluation)	Primary/health /social care<br>Secondary health/social care (not hospital)	Information and/or advice<br>Patient adherence<br>Progress review
Evaluating intervention: enhance adherence*	Sanders, 2011	Antihypertensives	NA	ACEIs to treat essential hypertension	Blood pressure control, mortality, morbidity, safety, specific adverse events, persistence/adherence, rate of use of a single antihypertensive medication for blood pressure control, lipid levels, rates of progression to type 2 diabetes, carbohydrate metabolism/diabetes control, left ventricular mass/function, kidney disease	Primary/health/ social care<br>Secondary health/social care (not hospital)<br>Hospital (outpatient)<br>Other<br>*Setting of studies not restricted*	Choosing a medication<br>Progress review<br>Patient adherence
Evaluating intervention: enhance adherence*	Nili, 2020	Antihypertensives	NA	Describe randomized clinical trial (RCT) interventions using health behavioral models/theories to improve medication adherence among adults with hypertension	Adherence, MMAS, discontinuation rate, MAES, MMAS, TAQPH, Self-report of number of missed pills, re view of prescription renewal patterns Steinberg's Hypertension Self-Efficacy Scale	Primary/health/soci al care<br>Hospital (outpatient)<br>Care home<br>Patient home	Information and/or advice<br>Patient adherence<br>Progress review<br>Maintaining treatmenT

For peer review only

For peer review only - http://bmjopen.bmj.com/site/about/guidelines.xhtml

Focus/ Aim	Study (First author, date)	Medications of interest	Other medications included	Intervention name/s and aim	Outcomes measured	Setting/Context	Relevant part of care pathway
Evaluating intervention: enhance adherence*	Mallat, 2016	Antihypertensives	NA	Free versus fixed drug combination. Aim: to increase adherence, increase overall BP-lowering efficacy	Mortality, morbidity (cardiovascular outcomes such as coronary events, stroke, progression of peripheral vascular disease and kidney disease), mean systolic BP, AE, adherence to treatment	NR	Choosing a medication Patient adherence Progress review
Evaluating intervention: enhance adherence*	de Cates, 2014	Statins Antihypertensives	NA	A fixed-dose combination therapy, a combination of several active components into a single pill, including at least one statin and one antihypertensive agent. Aim: to optimise CVD risk and reduce CVD fatal and non-fatal events	Mortality, non-fatal CVD endpoints, AEs (overall rates of discontinuation, proportion of participants experiencing specific symptoms or results and rates of discontinuation by specific symptoms). Secondary outcomes: Systolic and diastolic blood pressure, total and LDL cholesterol, adherence, health-related QOL, costs of fixed-dose combination therapy	Primary/health/ social care	Choosing a medication Progress Review Patient adherence

For peer review only - http://bmjopen.bmj.com/site/about/guidelines.xhtml

Focus/ Aim	Study (First author, date)	Medications of interest	Other medications included	Intervention name/s and aim	Outcomes measured	Setting/Context	Relevant part of care pathway
Evaluating intervention: enhance adherence*	Maimaris, 2013	Antihypertensives	NA	National or regional health system arrangements on HT awareness, treatment, control, and antihypertensive medication adherence	HT awareness (persons with clinically measured HT diagnosed by a health care professional as hypertensive), HT treatment (use of at least one antihypertensive medication in an individual with known HT), antihypertensive medication adherence, HT control	Primary/health/ social care<br>Secondary health/social care (not hospital)<br>Hospital (inpatient)<br>Hospital (outpatient)	Identifying health or prescribing concern<br>Accessing care<br>Decision to start agreed treatment plan<br>Patient adherence<br>Other *health systems, infrastructure. System level interventions*
Evaluating intervention: optimise prescribing	Hukins, 2019	Statins	Anticholinergic medications, anxiolytics, oestrogens, NSAID, antipsychotics, antiplatelets, proton pump inhibitors, benzodiazepines Hypnotics/Z-drugs	Any tool (dementia-specific or non-disease-specific) to identify potentially inappropriate prescribing	Prevalence of polypharmacy, prevalence of PIP, most common prescribed PIP medications	Primary/health/ social care<br>Secondary health/social care (not hospital)<br>Hospital (inpatient)<br>Hospital (outpatient)<br>Care home<br>Patient home	Identifying health or prescribing concern<br>Progress review<br>Decision to stop treatment: supported
Evaluating intervention: optimise prescribing*	Pantoja, 2019	Benzodiazepines<br>Hypnotics/Z-drugs<br>Statins<br>Antihypertensives	Anticonvulsants, antibiotic therapy, NSAID, nicotine gum, ezetimibe, thromboprophylaxis	Manual paper reminders to improve compliance with preventive guidelines and disease management guidelines	Patient-important endpoints: death, development of a pulmonary embolism , surrogate or intermediate endpoints: achievement of target blood pressure or serum cholesterol level, markers of disease/health status, adverse effects, resource use, changes in professional practice, patient outcomes	Primary/health /social care<br>Hospital (inpatient)<br>Hospital (outpatient)	Progress review<br>Identifying health or prescribing concern<br>Initial assessment<br>Prescribing guidelines

For peer review

Focus/ Aim	Study (First author, date)	Medications of interest	Other medications included	Intervention name/s and aim	Outcomes measured	Setting/Context	Relevant part of care pathway
Evaluating intervention: optimise prescribing*	Legare, 2010	Statins	Not clear	Interventions for improving the adoption of shared decision making by healthcare professionals	Healthcare professionals' adoption of SDM	Primary/health/ social care<br>Secondary health/social care (not hospital)	Initial assessment<br>Information and/or advice<br>Choosing a medication<br>Shared decision making<br>Decision to start agreed treatment plan
Evaluating intervention: optimise prescribing	Jeffery, 2015	Statins<br>Antihypertensives	NA	Interventions to improve adherence to CVD guidelines and patient outcomes: education, audit and feedback, academic detailing, comprehensive interventions that included education, audit and feedback and an academic detailing component	Guideline adherence, clinical outcomes	Primary/health/ social care<br>Secondary health/social care (not hospital)<br>Hospital (inpatient)Hospital (outpatient)	Identifying health or prescribing concern<br>Initial assessment<br>Information and/or advice<br>Prescribing guidelines<br>Progress review
Evaluating intervention: optimise prescribing	Shanbhag, 2018	Antihypertensives	NA	Implementation interventions classified at the provider, organisational or health system levels. Aim: increasing physician adherence to the specified HF guideline recommendations	Patient-level outcomes. Primary outcomes: process indicators (assessing guideline-consistent activities undertaken by a provider, proportion of patients who were: prescribed a guideline-recommended pharmacological treatment, referred for implantable cardioverter defibrillator, CRT consideration, provided self-care education at discharge, LVEF. Secondary outcomes: HF	Hospital (inpatient)<br>Hospital (outpatient)	Prescribing guidelines<br>Decision to start agreed treatment plan<br>Other

For peer review only - http://bmjopen.bmj.com/site/about/guidelines.xhtml

Focus/ Aim	Study (First author, date)	Medications of interest	Other medications included	Intervention name/s and aim	Outcomes measured	Setting/Context	Relevant part of care pathway
Evaluating intervention: optimise prescribing					specific/all-cause clinical outcomes		
	Zheng, 2020	Antihypertensives	NA	Nonphysician provider-led interventions: GDMT initiation and target dosage optimization	Primary outcomes: proportion of patients newly initiated into GDMT and the proportion of patients up titrated to target dosages of GDMT, stratified by therapeutic class. Secondary outcomes included clinical endpoints: all-cause mortality and hospitalizations due to HF. GDMT was defined as angiotensin-converting enzyme inhibitor/angiotensin II receptor blocker/angiotensin receptor-neprilysin inhibitor (ACEI/ARB/ARNI or RASI), BB, MRA, and SGLT2I. Outcomes related to GDMT management were collected only for therapeutic classes detailed in study-specific titration protocol	Secondary health/social care (not hospital)<br>Hospital (inpatient)<br>Hospital (outpatient)	Pre-treatment/Initiation<br>Maintaining treatment<br>Prescribing guidelines

*A secondary aim or one of multiple aims of review. Green highlighted text=High Overall Quality AMSTAR-2, Turquoise highlighted text=Medium Overall Quality AMSTAR-2, Orange highlighted text=Low Overall Quality AMSTAR-2, Red highlighted text=Critically-low Overall Quality AMSTAR-2. ACEI= Angiotensin-Converting Enzyme Inhibitors, AE=Adverse Events, AIDS= Acquired Immunodeficiency Syndrome, BEZRA=Benzodiazepines/Zdrugs, BP=Blood Pressure, CBT=Cognitive Behavioural Therapy, CPM=Continuous Passive Motion, CRT= Cardiac Resynchronisation Therapy, CV=Cardiovascular, CVD=Cardiovascular Disease, DBI=Drug Burden Index, ED=Emergency Department, GDR=Gradual Dose Reduction, GP=General Practitioner, HF=Heart Failure, HIV= Human Immunodeficiency Virus, HT=Hypertension, LDL= Low-Density Lipoprotein, LOS=Hospital Length of Stay, NA=Not Applicable, NR=Not Reported, QOL=Quality Of Life, LVEF= Left Ventricular Ejection Fraction, MBCBT=Mindfulness Based Cognitive Behavioural Therapy, NSAID=Non-Steroidal Anti-Inflammatories, PIP=Potentially Inappropriately Prescribed, SAE=Serious Adverse Events, SDM=Shared Decision Making, SMBP=Self-Monitoring of Blood Pressure, TKA=Total Knee Arthroplasty

For peer review only

Table 5: Systematic reviews scoring negatively on one CEESAT item which synthesised studies evaluating the effectiveness of an intervention

Focus/ Aim	Study (First author, date)	Medications of interest	Other medications included	Intervention name/s and aim	Outcomes measured	Setting/Context	Relevant part of care pathway
Evaluating intervention: deprescribing	Kornhold, 2022	Statins Antihypertensives	Treatment for: dementia, Parkinson's disease, orthostatic hypotension, dyskinesia, osteoporosis, Pain, LUTS, COPD, Insomnia (Lorazepam), Depression (Sertraline)	Discontinuation	Dropout rate (proportion of patients who did not complete the entire study per protocol in the discontinuation and continuation groups); Disease recurrence (proportion of patients with disease appearance for preventive treatments (eg, fractures after discontinuation of bisphosphonate treatment) or clinical worsening of the treated disease only for primarily symptomatic treatments (eg, pain after opioid treatment withdrawal) in the discontinuation and continuation group), Adverse drug withdrawal events	Primary/health/social care<br>Hospital (inpatient)<br>Care home	Decision to stop treatment: supported<br>Decision/ability to adhere to deprescribing plan

For peer review only

For peer review only - http://bmjopen.bmj.com/site/about/guidelines.xhtml

Focus/ Aim	Study (First author, date)	Medications of interest	Other medications included	Intervention name/s and aim	Outcomes measured	Setting/Context	Relevant part of care pathway
Evaluating intervention: deprescribing	Page, 2016	Statins Antihypertensives	Clopidogrel, Cilostazol, pentoxifylline, diuretics, nitrates, Glucosamine, bisphosphonates, calcium, vitamin D, Calcitriol, conjugated equine estrogens Premarin combined with medroxyprogeste rone acetate, Carbamazepine, Levodopa, lithium, anticholinestera, prednisolone, corticosteroids, d beta-2 receptor agonist, antipsychotics, benzodiazepines antidepressants	Deprescribing by a health care professional of one or more regular prescription medications:	Mortality, adverse drug withdrawal events, physical health, cognitive function and psychological health, quality of life	Hospital (inpatient) Care home Primary/health/ social care Hospital (outpatient) Patient home	Progress review Decision to stop treatment: supported

Focus/ Aim	Study (First author, date)	Medications of interest	Other medications included	Intervention name/s and aim	Outcomes measured	Setting/Context	Relevant part of care pathway
Evaluating intervention: enhance adherence	Park, 2017	Antihypertensives Statins	Antidementia medication, pneumonia-related ambulatory antibiotics, antilpemics, insulin, biologics, oral diabetes drugs, antiasthmatics, pain reliever, phosphate binders, cinacalce, teriparatide, antidepressants	Medicare Part D: increase drug utilization and lower Medicare and Medicaid participants out- of-pocket prescription drug costs, thereby improving access	Out-of-pocket costs, drug utilization	Primary/health/social care Hospital (inpatient) Hospital (outpatient)	Choosing a medication Patient adherence Progress review
Evaluating intervention: enhance adherence	Parati, 2021	Antihypertensives	Na	Single Pill Combination Therapy: determine whether SPC therapy leads to improved adherence, patient persistence, and better BP control compared with FEC therapy in patients with hypertension	adherence, BP	NR	Choosing a medication Patient adherence Maintaining treatmen
Evaluating intervention: enhance adherence	Conn, 2015[70]	Statins Antihypertensives	Yes, in relation to following medical conditions: organ transplant, epilepsy, gastrointestinal, infections, chronic diseases, malaria, STD, neurological, HIV	Packaging interventions to improve adherence: blister packs, unit-packaging, unit-of-use systems, unit-of-dose packaging, monitored dosage systems	Adherence, health outcomes	Primary/health/social care Hospital (inpatient) Patient home	Patient adherence

Focus/ Aim	Study (First author, date)	Medications of interest	Other medications included	Intervention name/s and aim	Outcomes measured	Setting/Context	Relevant part of care pathway
**Evaluating intervention: enhance adherence**	Conn, 2015[71]	Statins *Inferred from population disease characteristics* Antihypertensives *Inferred from population disease characteristics*	Yes, in relation to following medical conditions: diabetes, HIV, renal disease, asthma, lung disease. stroke, gastrointestinal diseases, acute illness, allergies, autoimmune disease, blood disorders, cancer, eye problems, gynaecological disorders, liver disease, malaria, nervous system problems, osteoarthritis, osteoporosis, rheumatoid arthritis, seizures, skin diseases, organ transplant, tuberculosis, and vascular disease	Health care provider targeted interventions to improve medication adherence: Research staff trained providers, integration of care across providers, increased providers, communication skills, monitor patients adherence behaviour, Provider- patient medication concordance, Increased provider time with patients, reduce the geographical distance between providers and patients, increase continuity of care, activating provider	Adherence behaviour	Primary/health/social care	Accessing care Working with other health or social care services/ charities Shared decision making Decision to start agreed treatment plan Patient adherence Progress review

Focus/ Aim	Study (First author, date)	Medications of interest	Other medications included	Intervention name/s and aim	Outcomes measured	Setting/Context	Relevant part of care pathway
Evaluating intervention: enhance adherence	Conn, 2016	Statins<br>Antihypertensives<br>*All Inferred from participant sample characteristics*	Yes, in relation to following medical conditions: gallbladder disease, unspecified chronic illnesses, osteoarthritis, asthma, IBD, glaucoma, kidney transplant, anti-coagulant therapy, osteoarthritis stroke, diabetes, HIV, antidepressants	Medication Adherence Interventions: Intervention content could include: prompts/cues to administer medications, self-monitoring of medication administration, self-monitoring of disease symptoms, written instructions, rewards for increased adherence, increased communication between providers and patients, providing feedback to participants about their adherence, goal setting about adherence, habit assessment/modification, and problem solving about adherence challenges	Adherence	Primary/health/social care<br>Secondary health/social care (not hospital)<br>Patient home	Decision to start agreed treatment plan<br>Patient adherence<br>Progress review
Evaluating intervention: enhance adherence	Coronado-Vazquez, 2020	Statins<br>Antihypertensives	Antidepressants, medications to treat cancer screening and type 2 diabetes	Shared decision making: to balance the patients' right of autonomy with the practitioners' responsibility to protect patients' safety.[2] SDM is a process within the physician–patient relationship applicable to any clinical action, whether diagnostic, therapeutic, or preventive in nature	Adherence to the treatment, knowledge and awareness of the illness, satisfaction of both professionals and patients with the intervention, and decisional conflict	Primary health/social care	Information and/or advice<br>Shared decision making<br>Patient adherence

Focus/ Aim	Study (First author, date)	Medications of interest	Other medications included	Intervention name/s and aim	Outcomes measured	Setting/Context	Relevant part of care pathway
Evaluating intervention: enhance adherence	De Simoni, 2013	Antihypertensives	NA	Interventions to improve adherence to antihypertensive medications: education, lifestyle, verbal information/advice on disease and secondary-prevention drug treatment, goal setting, supply of printed information/advice material, screening for depression, personalized instructions, and integrated care	Adherence and blood pressure control	Primary/health/social care<br>Hospital (outpatient)<br>Patient home	Information and/or advice<br>Decision to start agreed treatment plan<br>Patient adherence<br>Progress review
Evaluating intervention: enhance adherence	Kassavou, 2018	Statins<br>Antihypertensives	Yes, NR	Automated telecommunication systems, including voice messaging and text messaging: promote adherence to cardio-metabolic medications	Adherence	Primary/health/social care<br>Secondary health/social care (not hospital)<br>Hospital (inpatient)<br>Hospital (outpatient)<br>Patient home	Patient adherence
Evaluating intervention: enhance adherence	Krack, 2019	Statins<br>Antihypertensives	Calcium channel blockers, Clopidogrel, insulin oral antidiabetics, diabetes drugs, inhaled steroids, metformin, thiazides, Inhaled corticosteroid	Value-based health insurance designs including: VBID vs usual medication coverage VBID+education vs usual medication coverage, VBID+education vs usual medication coverage+education<br><br>Aim: increase patients understanding of importance of high-value medications, increase number of adherent patients without increasing total health care spending	Adherence	Primary/health/social care	Information and/or advice<br>Patient adherence

For peer review only - http://bmjopen.bmj.com/site/about/guidelines.xhtml

Focus/ Aim	Study (First author, date)	Medications of interest	Other medications included	Intervention name/s and aim	Outcomes measured	Setting/Context	Relevant part of care pathway
Evaluating interventio n: enhance adherence	Kronish, 2011	Antihypertensives	NA	Drug Class: improve adherence to antihypertensive medication	Adherence	NR	Choosing a medication Patient adherence
Evaluating Intervention: enhance adherence	Lawrence, 2015	Statins Antihypertensives	Antithrombotic medication	Multimodal behavioural interventions for secondary stroke prevention: addresses: 1) medication education and/or medication compliance education, 2) education or active information provision e.g. about stroke, stroke (lifestyle) risk factors, and 3) one or more of four specified lifestyle behaviours i.e. smoking, diet, physical inactivity, and alcohol consumption, and/or behaviours associated with amelioration of lifestyle risk factors i.e. medication compliance and management of perceived psychosocial stress	Primary outcomes of interest: physiological outcomes e.g. blood pressure, blood lipids, and lifestyle behaviour change. Secondary outcomes: psychosocial outcomes e.g. anxiety, learning e.g. knowledge of lifestyle risk factors for stroke, incidence of vascular events and mortality	Secondary health/social care (not hospital) Patient home	Information and/or advice Patient adherence Progress review
Evaluating intervention: enhance adherence	Mann, 2014	Statins Antihypertensives	NA	Drug Insurance Cost Sharing Strategies: lower expenditures for prescription drug insurance plans	Adherence, clinical events (myocardial infarction, stroke, death), QOL, healthcare utilization, or cost	NR	Accessing care Decision to start agreed treatment plan Patient adherence

Focus/ Aim	Study (First author, date)	Medications of interest	Other medications included	Intervention name/s and aim	Outcomes measured	Setting/Context	Relevant part of care pathway
Evaluating intervention: enhance adherence	Milosavljevic, 2018	Antihypertensives	Yes, in relation to following conditions: Asthma, diabetes, dyslipidemia, COPD, 'older people's health' aldosterone-inhibiting diuretics (for heart failure), intravenous vitamin D analogs, calcimimetic, oral hypoglycemics, antipsychotics, other central nervous system drugs, beta blockers, analgesics, antidepressants	Community pharmacist interventions: improve adherence, improve clinical outcomes.	Behavioural outcome: adherence, self-efficacy. Clinical outcome: clinical biomarkers (e.g. blood pressure, glycosylated haemoglobin, LDL), hospitalisation rates, mortality, emergency room visits, markers of disease progress. Economic outcome: cost effectiveness analysis, or other relevant analysis. Humanistic outcome: patient quality of life, knowledge, or satisfaction	Primary/health/social care Patient home	Information and/or advice Choosing a medication Decision to start agreed treatment plan Progress review Patient adherence

For peer review only - http://bmjopen.bmj.com/site/about/guidelines.xhtml

Focus/ Aim	Study (First author, date)	Medications of interest	Other medications included	Intervention name/s and aim	Outcomes measured	Setting/Context	Relevant part of care pathway
Evaluating intervention: enhance adherence	Rash, 2016	Statins	NA	Interventions targeting medication adherence:<br>Prescription cost coverage, simplification of drug regimen, reminders, education-based, multi-faceted interventions (including one or more of following: education, counselling, behavioural interventions, follow-up, medication review, risk-factor and adherence education, personalized health reports, bi-monthly mailings, collaborative care)	Patient adherence	Primary/health/social care<br>Secondary health/social care (not hospital)<br>Hospital (inpatient)<br>Hospital (outpatient)	Information and/or advice<br>Working with other health/social care services<br>Choosing a medication<br>Shared decision making<br>Patient adherence<br>Progress review
Evaluating intervention: enhance adherence	Reeves, 2020	Antihypertensives	NA	Pharmacist-led interventions to improve medication adherence and BP control	BP control, medication adherence	Primary/health/social care<br>Hospital (inpatient)<br>Hospital (outpatient)	Information and/or advice<br>Patient adherence<br>Progress review
Evaluating intervention: enhance adherence	Vervloet, 2020	Statin<br>Antihypertensives	Anticoagulants	Mix: improve adherence	Adherence	NR<br>Primary care	Information and/or advice<br>Patient adherence<br>Progress review<br>Maintaining treatment
Evaluating intervention: enhance adherence and prescribing	Xie, 2022	Statin<br>Antihypertensive	Antibiotics	Clinical dashboards: change clinician or patient behaviour	1) medication use, including the rate of medication prescribed/administered and medication intake adherence; (2) test ordering, such as the rate of imaging referrals and the count of routine laboratory test orders	Primary health/social care	Initial assessment<br>Prescribing guidelines<br>Patient adherence<br>Maintaining treatment

For peer review only - http://bmjopen.bmj.com/site/about/guidelines.xhtml

Focus/ Aim	Study (First author, date)	Medications of interest	Other medications included	Intervention name/s and aim	Outcomes measured	Setting/Context	Relevant part of care pathway
Evaluating intervention: enhance adherence*	Cheema, 2014	Antihypertensives	NA	Interventions to improve management of BP: Pharmacological: education on drug treatment of BP, advice to patients to improve medication adherence, identifying drug adverse effects and drug prescribing issues, liaising with prescribers about concerns of drug treatment. Nonpharmacological: education about hypertension. education about lifestyle (advice to patients on diet, weight management, alcohol consumption, smoking cessation)	Systolic and diastolic BP, adherence, identification and management of drug-related problems, cardiovascular risk factors, i.e. smoking, alcohol consumption	Primary/health/social care	Information and/or advice<br>Working with other health/social care services<br>Identifying health or prescribing concern<br>Patient adherence<br>Progress review
Evaluating intervention: optimise prescribing	Nguyen, 2018	Statins	Aspirin, antiplatelet agents	Organisational or professional interventions to enhance prescribing of guideline-recommended medications. Interventions included: Distribution of educational materials, educational outreach visits, audit and feedback and reminders, continuity of care, communication and case discussions between distant healthcare professionals, distribution of published/printed recommendations for clinical care, including clinical practice guidelines	Proportion of patients receiving guideline-recommended medications. Secondary outcomes: proportion of patients achieving target blood pressure and target LDL C/cholesterol level, mortality rate	Primary/health/social care<br>Hospital (outpatient)	Identifying health or prescribing concern<br>Initial assessment<br>Information and/or advice<br>Choosing a medication<br>Prescribing guidelines

Focus/ Aim	Study (First author, date)	Medications of interest	Other medications included	Intervention name/s and aim	Outcomes measured	Setting/Context	Relevant part of care pathway
Evaluating intervention: optimise prescribing<br>Evaluating intervention: enhance adherence	King, 2018	Statins<br>Antihypertensives	Diabetic medication, Digoxin, mixed medications, antidepressants	Longer-duration (2–4 months) versus shorter-duration (28-day)prescriptions to improve patient outcomes	Health outcomes, adverse events, medication adherence, medication wastage, professional administration time, pharmacists time and/or costs, patient experience, and patient out-of-pocket costs	Primary/health/social care<br>Secondary health/social care (not hospital)<br>Hospital (inpatient)	Initial assessment<br>Choosing a medication<br>Decision to start agreed treatment plan<br>Patient adherence<br>Progress review

*Secondary aim or outcome within the review. AE=Adverse Events, BI=Behavioural Interventions, BP=Blood Pressure, BZ=Benzodiazepine, CBT=Cognitive Behavioural Therapy, COPD=Coronary Obstructive Pulmonary Disease, DBT=Dialectical Behavioural Therapy, ED=Emergency Department, GP=General Practitioner, HIV= Human Immunodeficiency Virus, IBD=Irritable Bowel Disease, LDL= Low-Density Lipoprotein, LTOT=Long-Term Opioid Therapy, MDD=Major Depressive Disorder, MI=Motivational Interviewing, NA=Not Applicable, NR=Not Reported, QOL=Quality of Life, VBID=Value Based Insurance Design

For peer review only - http://bmjopen.bmj.com/site/about/guidelines.xhtml

Table 6: Systematic reviews scoring negatively on 2-4 CEESAT items which synthesised studies evaluating the effectiveness of an intervention

Focus/ Aim	Study (First author, date)	Medications of interest	Other medications included	Intervention name/s and aim	Outcomes measured	Setting/Context	Relevant part of care pathway
Evaluating intervention: deprescribing*.	Hart, 2020	Antihypertensives	Antipsychotics, Antihistamines, Antivertigo, Anti-Parkinson, Urinary spasmolytics, Vasodilators, Digoxin, Diuretics, Beta Blockers, Hypoglycemics, Beta blocker eye drops, Alpha adrenergic antagonists, Alpha blockers, Benzodiazepines Opiates Hypnotics/Z-drugs Antidepressants	Interventions to reduce falls or use of FRIDs: Face-to-face medication consultations with community pharmacy resident, fall-related assessment performed by research physician, geriatrician assessment of falls risk and systematic medication review, consideration of potential FRIDs for withdrawal+prescribing physician contact	Change in FRID use	Hospital (inpatient) Hospital (outpatient)	Initial assessment Choosing a medication Progress review Decision to stop treatment: supported Other *Medication review in acute care (e.g. after hip fracture)*
Evaluating interventions: deprescribing	Chambergo-Michilot, 2023	Antihypertensives	No	NR	Discontinuation	Hospital (inpatient)	Discontinuing treatment

For peer review only - http://bmjopen.bmj.com/site/about/guidelines.xhtml

Focus/ Aim	Study (First author, date)	Medications of interest	Other medications included	Intervention name/s and aim	Outcomes measured	Setting/Context	Relevant part of care pathway
Evaluating intervention: enhance adherence	Al Alshaikh, 2016	Statins Antihypertensives	Aspirin, Dipyridamole, antithrombotics, antiplatelets, anticoagulants, diabetic medications	Multimodal Interventions to improve adherence: categories included educational+motivational, simplification of drug regimen, environmental cues or reminders+reducing concerns and misbeliefs regarding medications, combined interventions to enhance risk factor management after stroke, prescribed medications+educated about them	Medication adherence	Primary/health/social care<br>Hospital (inpatient)<br>Hospital (outpatient)<br>Patient home	Information and/or advice<br>Patient adherence
Evaluating intervention: enhance adherence	Baumgartner, 2020	Statins Antihypertensives	Yes, in relation to following medical conditions:<br>HIV, diabetes, tuberculosis<br><br>Specific medications include:<br>metformin/sulfonylurea, various oral antidiabetic drugs, pioglitazone/ thiazolidinedione+ sulfonurea, FDC: rosiglitazone/glimepiride, glyburide/metformin, thiazolidinedione, Î±-blocker/antimuscarinic	Polypill to improve medication adherence	Adherence	Primary/health/social care<br>Hospital (inpatient)<br>Hospital (outpatient)	Choosing a medication<br>Decision to start agreed treatment plan<br>Patient adherence<br>Progress review

Focus/ Aim	Study (First author, date)	Medications of interest	Other medications included	Intervention name/s and aim	Outcomes measured	Setting/Context	Relevant part of care pathway
Evaluating intervention: enhance adherence	Bochkareva, 2019	Antihypertensives	NA	Medical Assistance Scheme to increase adherence to treatment: Examples include special packaging of medications, amount of prescribed medications, interventions to improve communication with patients (more frequent visits, motivational interviewing, patient education, home BP monitoring, and provision of written instructions), duration and number of sessions/consultations	Adherence	Primary/health/social care<br>Secondary health/social care (not hospital)<br>Hospital (outpatient)	Information and/or advice<br>Patient adherence
Evaluating intervention: enhance adherence	Bond, 2021	Statins	No	Mobile health: improve adherence	Adherence	Hospital (outpatient)<br>*Assumed*<br>Patient home	Information and/or advice<br>Patient adherenc

For peer review only - http://bmjopen.bmj.com/site/about/guidelines.xhtml

Focus/ Aim	Study (First author, date)	Medications of interest	Other medications included	Intervention name/s and aim	Outcomes measured	Setting/Context	Relevant part of care pathway
Evaluating intervention: enhance adherence	Checchi, 2014	Antihypertensives Statins	Various HAARTs, luticasone propionate, travoprost, pilocarpine, antidiabetic medications, ipratropium bromide, lithium, Clopidogrel, bupropion-SR, antipsychotics, fluticasone, sulfonylureas, immuno-suppressants	Electronic medication packaging devices to promote medication adherence	Adherence, health outcomes, cost effectiveness results, and satisfaction	Primary/health/social care<br>Patient home	Patient adherence<br>Information and/or advice
Evaluating intervention: enhance adherence	Conn 2015[36]	Antihypertensives	NA	Interventions to increase adherence among adults with hypertension	Adherence	Primary/health/social care<br>NR	Information and/or advice<br>Patient adherence<br>Progress review<br>Referral to specialist care

For peer review only - http://bmjopen.bmj.com/site/about/guidelines.xhtml

Focus/ Aim	Study (First author, date)	Medications of interest	Other medications included	Intervention name/s and aim	Outcomes measured	Setting/Context	Relevant part of care pathway
Evaluating intervention: enhance adherence	Deichmann, 2016	Statins	Fibrates, Anion-exchange resins or bile acid sequestrants, Niacin or nicotinic acid, combined medication regimen	Interventions to increase adherence to self-administered lipid-lowering medication: Drug regimen simplification, patient education and information, intensified patient care with reminders via mail, telephone, and hand-held pill devices, complex behavioural approaches, group sessions, decision support systems, administrative improvements, pharmacy-led automated telephone intervention	Adherence to lipid-lowering medical therapy. Secondary outcomes: physiologic indicators (e.g., total cholesterol), health outcome indications (e.g., QOL, morbidity, mortality), adverse effects, implications for costs	Primary/health/social care<br>Patient home	Information and/or advice<br>Choosing a medication<br>Patient adherence<br>Progress review
Evaluating intervention: enhance adherence	Du, 2018	Antihypertensives	NA	Fixed-dose combination versus free-equivalent combination therapies on adherence to medication for hypertension	Medication adherence/persistence	NR	Choosing a medication<br>Patient adherence<br>Progress review
Evaluating intervention: enhance adherence	Gupta, 2010	Antihypertensives	NA	Fixed-Dose Combinations of Antihypertensive agents. Aim: to achieve better BP control by improving compliance	Compliance (or adherence), persistence, BP-lowering efficacy, adverse effects	NR	Choosing a medication<br>Patient adherence<br>Progress review

For peer review only - http://bmjopen.bmj.com/site/about/guidelines.xhtml

Focus/ Aim	Study (First author, date)	Medications of interest	Other medications included	Intervention name/s and aim	Outcomes measured	Setting/Context	Relevant part of care pathway
Evaluating intervention: enhance adherence	Guzman-Tordecilla, 2020	Antihypertensives	NA	Interventions to enhance adherence and control of arterial hypertension: community fund organization, reminders, educational material and calendar, sessions for the optimization of the use of medicines, advice with experienced pharmacists, group educational meetings, obtention of medical records, identification of problems with medications and intervention on them, follow-up with the pharmacist and education	Adherence, control of HBP	Primary/health/social care<br>Patient home	Information and/or advice<br>Working with other health or social care services/charities<br>Patient adherence<br>Progress review
Evaluating intervention: enhance adherence	Hong, 2022	Antihypertensives	No	Mix: influence decisions of the patients with CHD on their adherence to antihypertensive medications	Adherence	Primary/health/ social care<br>Hospital (inpatient)<br>Hospital (outpatient)	

For peer review only - http://bmjopen.bmj.com/site/about/guidelines.xhtml

Focus/ Aim	Study (First author, date)	Medications of interest	Other medications included	Intervention name/s and aim	Outcomes measured	Setting/Context	Relevant part of care pathway
Evaluating interventio n: enhance adherence	Kawalec, 2018	Antihypertensives	NA	Fixed-dose combination therapy. Aim: blood pressure lowering, increasing patients adherence	Medication adherence, blood pressure lowering, persistence, health outcomes and adverse events	Primary/health/soci al care<br>Patient home	Choosing a medication<br>Patient adherence<br>Progress review
-Evaluating intervention: enhance adherence	Leslie, 2016	Antihypertensives<br>Statins (assumed)	Oral diabetes medications	Interventions aimed to modify medication adherence behaviour to chronic medications administered orally to treat diabetes, hypertension, or hyperlipidaemia: behavioural counselling, education and Informational materials, medication packaging that increased convenience by combining pills in a blister pack	Adherence to medications	Primary/health/soci al care<br>Care home<br>Patient home	Information and/or advice<br>Patient adherence<br>Progress review
Evaluating intervention: enhance adherence	Mamudu, 2014	Statins<br>Antihypertensives	Diabetic medications, Aspirin	Coronary artery calcium screening: enable risk stratification, diagnosis of CAD and directs the treatment and management of CAD among asymptomatic individuals	Behavioural modification, risk perception, and medication adherence, lifestyle change	Primary/health/soci al care	Identifying health or prescribing concern<br>Initial assessment<br>Patient adherence
Evaluating interventio n: enhance adherence	Tsioufis, 2020	Antihypertensives	NA	Single-pill combination therapy to treat hypertension. Aim: improve clinical outcomes and medication adherence	Adherence and/or persistent, BP, cardiovascular outcomes, mortality or morbidity	Primary/health/soci al care	Choosing a medication<br>Patient adherence<br>Progress review

For peer review only - http://bmjopen.bmj.com/site/about/guidelines.xhtml

Focus/ Aim	Study (First author, date)	Medications of interest	Other medications included	Intervention name/s and aim	Outcomes measured	Setting/Context	Relevant part of care pathway
Evaluating intervention: enhance adherence	Uhlig, 2013	Antihypertensives	NA	Interventions to enhance management of hypertension: self-measured blood pressure with or without additional support	Clinical outcomes (e.g. death and cardiovascular events), patient-reported outcomes (e.g. patient satisfaction or QOL), surrogate outcomes (e.g. measures of left ventricular hypertrophy), intermediate outcomes (e.g. BP; number, dose, or changes of antihypertensive medications; adherence to antihypertensive medication), health care utilization (including visits, calls, and e-mails	Primary/health/social care<br>Patient home	Patient adherence<br>Progress review
Evaluating intervention: enhance adherence	Xiong, 2018	Antihypertensives	Immunosuppressant's (n=1), lipid-lowering medication (n=1)	Mobile health technology to enhance medication adherence	Medication adherence	Primary/health/social care<br>Secondary health/social care (not hospital)<br>Hospital (outpatient)<br>Patient home	Patient adherence
Evaluating intervention: enhance adherence	Xu, 2018	Antihypertensives	NA	Medication education, self-monitoring of BP, reminders and regular follow-up visits to improve medication adherence	Adherence, BP, BP control	Primary/health/social care<br>Hospital (outpatient) *might be inpatient*<br>Patient home	Patient adherence<br>Information and/or advice<br>Progress review

For peer review only - http://bmjopen.bmj.com/site/about/guidelines.xhtml

Focus/ Aim	Study (First author, date)	Medications of interest	Other medications included	Intervention name/s and aim	Outcomes measured	Setting/Context	Relevant part of care pathway
Evaluating intervention: enhance adherence	Demonceau, 2013	Antihypertensives *Assumed*	Medications for following medications:: HIV, asthma, osteoporosis Specific medications: sulfonylurea drugs, oral nitrates	Interventions to affect adherence to self-administered prescribed medications in short-term and in long-term therapy: treatment simplification, cognitive-educational interventions, behavioural counselling, social-psycho-affective interventions, electronically monitored adherence feedback, technical reminder systems, monitoring the disease being managed, rewards, integrating depression treatment with hypertension management or type 2 diabetes care, home-based physician-directed, nurse-guided drug therapy, drug regimen	Health outcomes' e.g. reductions in blood pressure, lower levels of glycosylated haemoglobin, medication adherence	Primary/health/social care<br>Hospital (inpatient)<br>Hospital (outpatient)<br>Patient home	Information and/or advice<br>Working with other health/social care services<br>Patient adherence<br>Referral to specialist care
Evaluating intervention: enhance adherence<br>Evaluating intervention: optimise prescribing	Laba, 2013	Statins<br>Antihypertensives	Yes<br>*Not all medicines reported, but all for CVD*<br>e.g. spironolactone, loop diuretics, digoxin	Strategies to improve patient adherence to cardiovascular medications:<br>education, persuasion, incentivisation, enablement, training, coercion, restriction, environmental restructuring, modelling, policies, communication/marketing, guidelines, fiscal, regulation, legislation, environmental/social planning, service provision	Adherence, Physiological: BP control, mean DBP/SBP, lipids, glucose/HbA1c, INR, echocardiogram, health outcomes, health service utilisation, mortality, QOL, other lifestyle, smoking/alcohol, nutrition, physical activity, stress, adverse events, cost comparison, cost effectiveness	Primary/health/social care<br>Patient home<br>Hospital (outpatient)	Information and/or advice<br>Shared decision making<br>Patient adherence<br>Progress review<br>Prescribing guidelines

For peer review only - http://bmjopen.bmj.com/site/about/guidelines.xhtml

Focus/ Aim	Study (First author, date)	Medications of interest	Other medications included	Intervention name/s and aim	Outcomes measured	Setting/Context	Relevant part of care pathway
Evaluating intervention: enhance adherence	Polinski, 2011	Statins<br>Antihypertensives	HEDIS, high risk□ drugs, anti-convulsants, antihistamines, propoxyphene, digoxin, NSAIDs, doxazosin, muscle relaxants/anti-spasmodics, others, warfarin, oral anti-diabetics, antiretroviral medications, warfarin, clopidogrel, antipsychotics, proton pump inhibitors, antibiotics, benzodiazepines antidepressants, hypnotics/Z-drugs (sedatives)	Medicare Part D cost-sharing provisions and drug coverage rules. Aim: improve access to essential medications through reduced cost-sharing with focus on under and over-use of specific drugs and classes	Reduced out-of-pocket costs, health	Primary/health/social care<br>Hospital (outpatient)	Accessing care<br>Choosing a medication<br>Decision to start agreed treatment plan<br>Patient adherence<br>Decision to stop treatment: unsupported

For peer review only - http://bmjopen.bmj.com/site/about/guidelines.xhtml

Focus/ Aim	Study (First author, date)	Medications of interest	Other medications included	Intervention name/s and aim	Outcomes measured	Setting/Context	Relevant part of care pathway
Evaluating intervention: optimise prescribing	Kang, 2016	Statins<br>Antihypertensives	Aspirin	Pharmacist-involved care (patient- targeted services) and healthcare professional-targeted services: PTS-counselling, providing educational materials, medical reminders, post-discharge follow- up services and supporting rehabilitation transition care. HTS-medication reconciliation, communication with health-care providers<br>Aim: improve CVD outcomes	All-cause mortality, all-cause hospitalization, cardiac-related hospitalization, key medication prescription rates, health-related QOL, medication adherence, medication utilization	Primary/health/social care<br>Hospital (inpatient)<br>Hospital (outpatient)<br>Patient home	Information and/or advice<br>Working alongside health/social care services<br>Choosing a medication<br>Patient adherence<br>Progress review
Evaluating intervention: optimise prescribing	Chhina, 2013	Antihypertensives	Propoxyphene, cerebral & peripheral vasodilators, cephalexin, amoxycillin with or without clavulinic acid, cephalexin, doxycycline, erythromycin, penicillin, rrimethoprim, cefaclor, roxithromycin, NSAIDS, metronidazole, omeprazole, inhaled steroids, antipsychotic, benzodiazepines antidepressants	Academic Detailing to modify drug prescription behaviour of Family Physicians in primary care settings	Prescription rate	Primary/health/social care	Choosing a medication<br>Progress review<br>Other<br>*Reducing repeat prescription*

For peer review only - http://bmjopen.bmj.com/site/about/guidelines.xhtml

Focus/ Aim	Study (First author, date)	Medications of interest	Other medications included	Intervention name/s and aim	Outcomes measured	Setting/Context	Relevant part of care pathway
Evaluating intervention: optimise prescribing*<br>Evaluating intervention: enhance adherence*	Yuan, 2019	Antihypertensives	Yes, associated with following medical conditions: diabetes, chronic obstructive pulmonary disease, osteoporosis, obesity, antiphospholipid syndrome, drug related high risk of disease, outpatients taking multiple prescription drugs, bronchial asthma	Community Pharmacist interventions to improve outcomes for people with hypertension: medication review, patient education, adherence assessment, health/lifestyle advice, physical assessment (e.g., BP), monitoring, prescribing, or adjusting and administering therapy	Clinical outcomes of pharmaceutical services e.g. common end- points of systolic blood pressure, diastolic blood pressure and glycosylated haemoglobin after providing CPS	Primary/health/social care<br>Patient home<br>Hospital (inpatient)	Initial assessment<br>Information and/or advice<br>Decision to start agreed treatment plan<br>Patient adherence<br>Progress review

*Secondary aim or one of multiple aims; [a]Note that focus is on 'use' which includes initiating prescribing. Drugs of interest may be currently under- or over-used, [b]One aspect of broad aim, [c]One aspect of interventions. BP=Blood Pressure, CAD=Coronary Artery Disease, CPS=Community Pharmacist Services, CVD=Cardiovascular Disease, COX-2=Cyclooxygenase-2, DBP-Diastolic Blood Pressure, FDC=Fixed-Dose Combination, FRID=Fall Risk Increasing Drugs, HAART= Highly Active Antiretroviral Therapy, HBP=High Blood Pressure, HIV= Human Immunodeficiency Virus, HRT=Hormonal Replacement Therapy, HTS=Healthcare professional Targeted Services, INR=International Normalised Ratio, NA=Not Applicable, NR=Not Reported, NSAID=Non-Steroidal Anti-Inflammatories,PTS=Patient Targeted Services, SBP=Systolic Blood Pressure, SH=Sedative Hypnotics, SR=Sustained Release, QOL=Quality of Life,

For peer review only - http://bmjopen.bmj.com/site/about/guidelines.xhtml

Table 7: Systematic reviews synthesising qualitative evidence

Study (First author, date)	Medications of interest	Other medications included	Perspectives obtained	Phenomenon of Interest	Setting/Context	Relevant part of care pathway
Ingersgaard, 2020	Statins<br>Antihypertensives	Lipid-lowering agents, Î² blockers, angiotensin, renin system blockers, aspirin	Patient	Experiences/views of an intervention (adherence)<br>Reasons for adherence/non-adherence	Primary/health/social care<br>NR	Patient adherence
Rashid, 2014	Statins	Clopidogrel, antiplatelet medication, aspirin	Patient<br>Family member	Reasons for adherence/non-adherence	Primary/health/social care<br>Hospital (outpatient)<br>Secondary health/social care (not hospital)<br>Patient home	Patient adherence<br>Decision to stop treatment: unsupported

For peer review only - http://bmjopen.bmj.com/site/about/guidelines.xhtml

Study (First author, date)	Medications of interest	Other medications included	Perspectives obtained	Phenomenon of Interest	Setting/Context	Relevant part of care pathway
Kinnear, 2019	Statins	NA	Patient<br>Carer<br>Family member	Experiences/views of an intervention (adherence)<br>Reasons for adherence/non-adherence	Primary/health/social care<br>Secondary health/social care (not hospital)	Initial assessment<br>Information and/or advice<br>Choosing a medication<br>Shared decision making<br>Patient adherence<br>Progress review<br>Decision to stop treatment: unsupported
Reeve, 2013	Antihypertensives	Any long-term medication, methadone (substitution program), antiepileptic's, HRT, PPIs, donepezil, benzodiazepines antidepressants	Patient<br>Carer	Experiences/views of an intervention (deprescribing)	NR	Decision to stop treatment: supported
Dhar, 2017	Antihypertensives	NA	Patient	Reasons for adherence/non-adherence	Primary/health/social care<br>Secondary health/social care (not hospital)<br>Hospital (outpatient)<br>Patient home	Patient adherence<br>Decision to stop treatment: unsupported

For peer review only - http://bmjopen.bmj.com/site/about/guidelines.xhtml

Study (First author, date)	Medications of interest	Other medications included	Perspectives obtained	Phenomenon of Interest	Setting/Context	Relevant part of care pathway
Marshall, 2012	Antihypertensives	NA	Patient	Reasons for adherence/non-adherence	Primary/health/social care<br>Hospital (outpatient)<br>Patient home	Choosing a medication<br>Decision to start agreed treatment plan<br>Patient adherence<br>Decision to stop treatment: supported<br>Decision to stop treatment: unsupported
Khatib, 2014	Antihypertensives	NA	Practitioner/Prescriber<br>Patient	Prescribing<br>Of an intervention (adherence)<br>Reasons for adherence/non-adherence	Primary/health/social care<br>Hospital (outpatient)<br>Patient home	Identifying health or prescribing concern<br>Accessing care<br>Initial assessment<br>Information and/or advice<br>Choosing a medication<br>Shared decision making<br>Prescribing guidelines<br>Decision to start agreed Patient adherence<br>Progress review

For peer review only - http://bmjopen.bmj.com/site/about/guidelines.xhtml

Study (First author, date)	Medications of interest	Other medications included	Perspectives obtained	Phenomenon of Interest	Setting/Context	Relevant part of care pathway
Oori, 2019	Antihypertensives	NA	Patient	Reasons for adherence/non-adherence	Care home<br>Primary/health/social care-<br>Secondary health/social care (not hospital)<br>Hospital (outpatient)<br>Patient home	Patient adherence

For peer review only - http://bmjopen.bmj.com/site/about/guidelines.xhtml

Study (First author, date)	Medications of interest	Other medications included	Perspectives obtained	Phenomenon of Interest	Setting/Context	Relevant part of care pathway
Rashid, 2018	Antihypertensives	Yes, relevant to the following conditions: asthma, renal transplant, proton pump inhibitors, schizophrenia, migraine, antiretroviral for HIV, osteoporosis, anxiety, malaria prophylaxis, bipolar, back pain, ADHD, dysphagia, IBD, RA, breast cancer, anticoagulants, borderline personality disorder, Specific drugs mentioned: clozapine, sumatriptan, SSRI, HAART, malaria prophylaxis, antibiotics, phosphate binding, insulin, clopidogrel, PMTCT, chemotherapy, Aripiprazole, Antidepressants	Practitioner/Prescriber<br>Patient<br>Carer<br>Family member	Experiences/views of an intervention (adherence)<br>Taking a medication	Primary/health/social care<br>Secondary health/social care (not hospital)<br>Hospital (inpatient)<br>Hospital (outpatient)	Accessing care<br>Initial assessment<br>Information and/or advice<br>Patient adherence<br>Progress review

For peer review only - http://bmjopen.bmj.com/site/about/guidelines.xhtml

Study (First author, date)	Medications of interest	Other medications included	Perspectives obtained	Phenomenon of Interest	Setting/Context	Relevant part of care pathway
Qadi, 2020	Statins<br>Antihypertensives	No	Practitioner/Prescriber<br>Patient	Prescribing medication<br>Taking medication	Primary health/social care	Initial assessment<br>Decision to start agreed treatment plan

* Data too thin for synthesis. Green highlighted text=High Overall Quality as appraised by AMSTAR-2, Turquoise highlighted text=Moderate Overall Quality as appraised by AMSTAR-2. ADHD=Attention-Deficit Hyperactivity Disorder, BZ=Benzodiazepine, HAART= Highly Active Antiretroviral Therapy, HIV= Human Immunodeficiency Virus, IBD=Irritable Bowel Disease, NA=Not Applicable, NR=Not Reported, PMTCT=Prevention of Mother-to-Child Transmission, RA=Rheumatoid Arthritis, SSRI=Selective Serotonin Reuptake Inhibitor

For peer review only - http://bmjopen.bmj.com/site/about/guidelines.xhtml

APPENDIX E: QUALITY APPRAISAL OF INCLUDED STUDIES

Table 8: Scores on CEESAT items included studies

First Author, Date	Search strategy: Is approach to searching clearly defined, systematic and transparent?	Is search comprehensive?	Does the review critically appraise each study?	During critical appraisal was an effort made to minimise subjectivity?	Total Score
Dills, 2018	Green	Green	Green	Green	0
Hukins, 2019	Green	Green	Green	Green	0
Pantoja, 2019	Green	Green	Green	Green	0
Posadzki, 2016	Green	Green	Green	Green	0
Stacey, 2017	Green	Green	Green	Green	0
Adler, 2017	Green	Green	Green	Green	0
de Cates, 2014	Green	Green	Green	Green	0
Fletcher, 2015	Green	Green	Green	Green	0
Jeffery, 2015	Green	Green	Green	Green	0
Kinnear, 2019	Green	Green	Green	Green	0
Legare, 2010	Green	Green	Green	Green	0
Maimaris, 2013	Green	Green	Green	Green	0
Mallat, 2016	Green	Green	Green	Green	0
Morrissey, 2017	Green	Green	Green	Green	0
Nieuwlaat, 2014	Green	Green	Green	Green	0
Nili, 2020	Green	Green	Green	Green	0
Palmer, 2018;2020	Green	Green	Green	Green	0
Sanders, 2011	Green	Green	Green	Green	0
Shanbhag, 2018	Green	Green	Green	Green	0
van Driel, 2016	Green	Green	Green	Green	0
Zheng, 2023	Green	Green	Green	Green	0
King, 2018	Green	Green	Amber	Green	1
Milosavljevic, 2018	Green	Green	Amber	Green	1
Page, 2016	Green	Green	Amber	Green	1
Park, 2017	Green	Green	Amber	Green	1

For peer review only - http://bmjopen.bmj.com/site/about/guidelines.xhtml

Cheema, 2014	Green	Green	Amber	Green	1
Conn, 2015[38]	Amber	Green	Green	Green	1
Conn, 2015[40]	Amber	Green	Green	Green	1
Conn, 2016	Amber	Green	Green	Green	1
Coronado- Vázquez, 2020	Green	Green	Amber	Green	1
de Simoni, 2013	Green	Amber	Green	Green	1
Ingersgaard, 2020	Green	Amber	Green	Green	1
Kornholt 2022	Green	Amber	Green	Green	1
Kassavou, 2018	Green	Green	Green	Red	1
Krack, 2019	Green	Green	Green	Red	1
Kronish, 2011	Green	Green	Amber	Green	1
Lawrence, 2015	Green	Green	Green	Red	1
Mann, 2014	Green	Green	Green	Red	1
Nguyen, 2018	Green	Amber	Green	Green	1
Parati, 2021	Green	Green	Green	Amber	1
Qadi, 2020	Green	Green	Amber	Green	1
Rash, 2016	Green	Green	Green	Red	1
Reeve,s 2020	Amber	Green	Green	Green	1
Reston, 2020	Green	Green	Amber	Green	1
Van Truong	Green	Amber	Green	Green	1
Verveloet, 2020	Green	Amber	Green	Green	2
Xie, 2022	Green	Green	Green	Amber	1
Loganathan, 2011	Green	Amber	Amber	Green	2
Bond, 2021	Green	Green	Amber	Ambeo	2
Mamudu, 2014	Green	Green	Red	Red	2
Oori, 2019	Amber	Green	Amber	Green	2
Xu, 2018	Green	Amber	Amber	Green	2
Checchi, 2014	Green	Amber	Green	Red	2
Demonceau, 2013	Green	Amber	Amber	Green	2
Rashid, 2018	Green	Amber	Amber	Green	2
Reeve, 2013	Green	Green	Amber	Red	2
Baumgartner, 2020	Green	Amber	Green	Red	2
Bochkareva, 2019	Green	Green	Amber	Red	2
Deichmann, 2016	Amber	Green	Amber	Green	2

Hong 2020	Green	Amber	Green	Amber	2
Khatib, 2014	Green	Amber	Green	Red	2
Laba, 2013	Green	Green	Amber	Red	2
Rashid, 2014	Green	Amber	Amber	Green	2
Tsioufis, 2020	Green	Amber	Green	Red	2
Uhlig, 2013	Green	Amber	Amber	Green	2
Yuan, 2019	Amber	Amber	Green	Green	2
Hart, 2020	Amber	Amber	Amber	Green	3
Polinski, 2011	Amber	Amber	Amber	Green	3
Al Alshaikh, 2016	Amber	Amber	Amber	Green	3
Conn, 2015	Amber	Green	Amber	Red	3
Dhar, 2017	Amber	Green	Amber	Red	3
Gupta, 2010	Amber	Amber	Amber	Green	3
Guzman-Tordecilla, 2020	Amber	Amber	Green	Red	3
Kang, 2016	Amber	Amber	Amber	Green	3
Marshall, 2012	Green	Amber	Amber	Red	3
Xiong, 2018	Green	Amber	Amber	Red	3
Du,2019	Amber	Amber	Amber	Red	4
Chhina, 2013.	Amber	Amber	Amber	Red	4
Kawalec, 2018	Amber	Amber	Amber	Red	4
Leslie, 2016[7]	Amber	Amber	Amber	Red	4

For peer review only - http://bmjopen.bmj.com/site/about/guidelines.xhtml

Table 9: Scores on AMSTAR-2 tool for reviews included in map with CEESAT score of 0

Study (First author, date)	1. PICO components	2. Protocol	3. Study design explanation	4. Comprehensive search strategy	5. Duplicate study selection	6. Duplicate data extraction	7. Details of excluded studies	8. Description of included studies	9b. RoB assessment (NRSIs)	10. Funding sources	9a. Risk of Bias (RoB) assessment (RCTs)	11a. RCTs Meta-analysis	11b. NRSIs Meta-analysis (MA)	12. MA: RoB in individual studies	13. Qualitative reviews: Is synthesis methodology /theoretical framework clearly described /justified?	14. Qualitative reviews: was description /rationale for sampling described?	15. Qualitative reviews: was process of data extraction clearly described?	16. Qualitative reviews: was process for coding data clearly described?	17. Qualitative reviews: was process of developing themes clearly described?	18. Qualitative reviews: Do authors reflect on how their theoretical /ideological perspectives may influence their findings?	19 Qualitative reviews: Was use of any software clearly described?	20. RoB: discussion of results	21. Heterogeneity	22. Publication bias	23. Reports conflicts of interest	Overall rating
Adler, 2017	Y	Y	N	Y	Y	Y	Y	Y	NA	Y	Y	N	NA	NA	NA	NA	NA	NA	NA	NA	NA	Y	Y	NA	Y	H
de Cates, 2014	Y	PY	N	Y	Y	Y	Y	Y	NA	N	Y	Y	NA	Y	NA	NA	NA	NA	NA	NA	NA	Y	Y	Y	Y	H
Dills 2018,	Y	N	N	Y	Y	Y	N	Y	NA	Y	Y	N	NA	NA	NA	NA	NA	NA	NA	NA	NA	Y	Y	NA	Y	L
Fletcher, 2015	Y	Y	Y	Y	Y	Y	N	PY	N	N	Y	Y	N	Y	NA	NA	NA	NA	NA	NA	NA	Y	Y	N	Y	CL
Hukins, 2019	Y	N	N	Y	Y	Y	N	Y	Y	N	NA	NA	N	NA	NA	NA	NA	NA	NA	NA	NA	Y	Y	NA	Y	L
Jeffery, 2015	Y	Y	N	Y	Y	Y	N	Y	Y	N	Y	Y	Y	Y	NA	NA	NA	NA	NA	NA	NA	Y	Y	N	Y	M
Kinnear, 2019	Y	Y	NA	Y	Y	Y	N	Y	Y	Y	NA	NA	N	NA	PY	NA	Y	Y	Y	N	Y	Y	Y	NA	Y	H
Legare, 2010	Y	Y	N	Y	Y	Y	Y	Y	NA	N	Y	N	NA	NA	NA	NA	NA	NA	NA	NA	NA	Y	Y	NA	Y	H
Maimaris, 2013	Y	Y	Y	Y	Y	Y	N	PY	PY	N	Y	N	N	NA	NA	NA	NA	NA	NA	NA	NA	Y	Y	NA	Y	M
Mallat, 2016	Y	Y	N	Y	Y	Y	N	Y	NA	Y	Y	Y	NA	Y	NA	NA	NA	NA	NA	NA	NA	Y	Y	Y	Y	H
Morrissey, 2017	Y	Y	N	Y	Y	Y	N	Y	NA	N	Y	N	NA	Y	NA	NA	NA	NA	NA	NA	NA	Y	Y	Y	Y	L

For peer review only - http://bmjopen.bmj.com/site/about/guidelines.xhtml

Study (First author, date)	1. PICO components	2. Protocol	3. Study design explanation	4. Comprehensive search strategy	5. Duplicate study selection	6. Duplicate data extraction	7. Details of excluded studies	8. Description of included studies	9b. RoB assessment (NRSIs)	10. Funding sources	9a. Risk of Bias (RoB) assessment (RCTs)	11a. RCTs Meta-analysis	11b. NRSIs Meta-analysis (MA)	12. MA: RoB in individual studies	13. Qualitative reviews: Is synthesis methodology /theoretical framework clearly described /justified?	14. Qualitative reviews: was description /rationale for sampling described?	15. Qualitative reviews: was process of data extraction clearly described?	16. Qualitative reviews: was process for coding data clearly described?	17. Qualitative reviews: was process of developing themes clearly described?	18. Qualitative reviews: Do authors reflect on how their theoretical /ideological perspectives may influence their findings?	19 Qualitative reviews: Was use of any software clearly described?	20. RoB: discussion of results	21. Heterogeneity	22. Publication bias	23. Reports conflicts of interest	Overall rating
Nieuwlaat, 2014	Y	Y	N	Y	Y	Y	Y	Y	NA	N	Y	N	NA	NA	NA	NA	NA	NA	NA	NA	NA	Y	Y	NA	Y	M
Nili, 2020	Y	PY	N	PY	Y	N	N	PY	NA	Y	Y	NA	NA	NA	NA	NA	NA	NA	NA	NA	NA	N	N	NA	Y	CL
Palmer, 2018	Y	Y	Y	Y	Y	Y	Y	Y	NA	Y	Y	N	NA	NA	NA	NA	NA	NA	NA	NA	NA	Y	Y	NA	Y	H
Palmer	Y	Y	Y	Y	Y	Y	Y	Y	Y	Y	Y	Y	NA	Y	NA	NA	NA	NA	NA	NA	NA	Y	Y	Y	Y	H
Pantoja, 2019	Y	Y	N	Y	Y	Y	Y	Y	Y	Y	Y	N	N	NA	NA	NA	NA	NA	NA	NA	NA	Y	Y	NA	Y	H
Posadzki, 2016	Y	Y	Y	Y	Y	Y	Y	Y	Y	Y	Y	Y	Y	Y	NA	NA	NA	NA	NA	NA	NA	Y	Y	N	Y	H
Sanders, 2011	Y	N	N	Y	Y	Y	Y	Y	PY	Y	PY	Y	Y	N	NA	NA	NA	NA	NA	NA	NA	N	Y	N	Y	CL
Shanbhag, 2018	Y	Y	N	Y	Y	Y	N	PY	Y	N	Y	N	N	NA	NA	NA	NA	NA	NA	NA	NA	N	Y	NA	Y	L
Stacey, 2017	Y	Y	N	Y	Y	Y	Y	Y	NA	N	Y	Y	NA	Y	NA	NA	NA	NA	NA	NA	NA	Y	Y	Y	Y	M
van Driel, 2016	Y	Y	N	Y	Y	Y	Y	Y	NA	Y	Y	Y	NA	Y	NA	NA	NA	NA	NA	NA	NA	Y	Y	Y	Y	H
Zheng, 2023	Y	Y	N	Y	Y	Y	Y	Y	Y	N	Y	Y	NA	Y	NA	NA	NA	NA	NA	NA	NA	Y	Y	Y	N	H

CL=Critically Low overall quality; H=High overall quality; Low overall quality; M=Moderate overall quality; N=No; NA=Not Applicable; NRSI=Non-Randomised Studies of healthcare Interventions; PICO=Population, Intervention, Comparator, Outcome; PY=Partial Yes; RCT=Randomised Controlled Trial; RoB=Risk of Bias; Y=Yes

For peer review only - http://bmjopen.bmj.com/site/about/guidelines.xhtml

For peer review only

For peer review only - http://bmjopen.bmj.com/site/about/guidelines.xhtml

PRISMA 2020 Checklist

Section and Topic	Item #	Checklist item	Location where item is reported
**TITLE**			
Title	1	Identify the report as a systematic review.	1
**ABSTRACT**			
Abstract	2	See the PRISMA 2020 for Abstracts checklist.	2
**INTRODUCTION**			
Rationale	3	Describe the rationale for the review in the context of existing knowledge.	4
Objectives	4	Provide an explicit statement of the objective(s) or question(s) the review addresses.	4
**METHODS**			
Eligibility criteria	5	Specify the inclusion and exclusion criteria for the review and how studies were grouped for the syntheses.	7-8
Information sources	6	Specify all databases, registers, websites, organisations, reference lists and other sources searched or consulted to identify studies. Specify the date when each source was last searched or consulted.	6
Search strategy	7	Present the full search strategies for all databases, registers and websites, including any filters and limits used.	Supplementary Materials
Selection process	8	Specify the methods used to decide whether a study met the inclusion criteria of the review, including how many reviewers screened each record and each report retrieved, whether they worked independently, and if applicable, details of automation tools used in the process.	6-7
Data collection process	9	Specify the methods used to collect data from reports, including how many reviewers collected data from each report, whether they worked independently, any processes for obtaining or confirming data from study investigators, and if applicable, details of automation tools used in the process.	7-8
Data items	10a	List and define all outcomes for which data were sought. Specify whether all results that were compatible with each outcome domain in each study were sought (e.g. for all measures, time points, analyses), and if not, the methods used to decide which results to collect.	8
	10b	List and define all other variables for which data were sought (e.g. participant and intervention characteristics, funding sources). Describe any assumptions made about any missing or unclear information.	8
Study risk of bias assessment	11	Specify the methods used to assess risk of bias in the included studies, including details of the tool(s) used, how many reviewers assessed each study and whether they worked independently, and if applicable, details of automation tools used in the process.	7-8
Effect measures	12	Specify for each outcome the effect measure(s) (e.g. risk ratio, mean difference) used in the synthesis or presentation of results.	8
Synthesis methods	13a	Describe the processes used to decide which studies were eligible for each synthesis (e.g. tabulating the study intervention characteristics and comparing against the planned groups for each synthesis (item #5)).	8-9
	13b	Describe any methods required to prepare the data for presentation or synthesis, such as handling of missing summary statistics, or data conversions.	na
	13c	Describe any methods used to tabulate or visually display results of individual studies and syntheses.	8
	13d	Describe any methods used to synthesize results and provide a rationale for the choice(s). If meta-analysis was performed, describe the model(s), method(s) to identify the presence and extent of statistical heterogeneity, and software package(s) used.	na
	13e	Describe any methods used to explore possible causes of heterogeneity among study results (e.g. subgroup analysis, meta-regression).	na
	13f	Describe any sensitivity analyses conducted to assess robustness of the synthesized results.	na
Reporting bias assessment	14	Describe any methods used to assess risk of bias due to missing results in a synthesis (arising from reporting biases).	na

For peer review only

PRISMA 2020 Checklist

Section and Topic	Item #	Checklist item	Location where item is reported
Certainty assessment	15	Describe any methods used to assess certainty (or confidence) in the body of evidence for an outcome.	na
**RESULTS**			
Study selection	16a	Describe the results of the search and selection process, from the number of records identified in the search to the number of studies included in the review, ideally using a flow diagram.	10
	16b	Cite studies that might appear to meet the inclusion criteria, but which were excluded, and explain why they were excluded.	Supplementary Materials
Study characteristics	17	Cite each included study and present its characteristics.	10-13
Risk of bias in studies	18	Present assessments of risk of bias for each included study.	10, Supplementary Materials
Results of individual studies	19	For all outcomes, present, for each study: (a) summary statistics for each group (where appropriate) and (b) an effect estimate and its precision (e.g. confidence/credible interval), ideally using structured tables or plots.	NA
Results of syntheses	20a	For each synthesis, briefly summarise the characteristics and risk of bias among contributing studies.	Supplementary Materials, 10-13
	20b	Present results of all statistical syntheses conducted. If meta-analysis was done, present for each the summary estimate and its precision (e.g. confidence/credible interval) and measures of statistical heterogeneity. If comparing groups, describe the direction of the effect.	na
	20c	Present results of all investigations of possible causes of heterogeneity among study results.	na
	20d	Present results of all sensitivity analyses conducted to assess the robustness of the synthesized results.	na
Reporting biases	21	Present assessments of risk of bias due to missing results (arising from reporting biases) for each synthesis assessed.	na
Certainty of evidence	22	Present assessments of certainty (or confidence) in the body of evidence for each outcome assessed.	na
**DISCUSSION**			
Discussion	23a	Provide a general interpretation of the results in the context of other evidence.	na
	23b	Discuss any limitations of the evidence included in the review.	14
	23c	Discuss any limitations of the review processes used.	14
	23d	Discuss implications of the results for practice, policy, and future research.	14-15
**OTHER INFORMATION**			
Registration and protocol	24a	Provide registration information for the review, including register name and registration number, or state that the review was not registered.	4
	24b	Indicate where the review protocol can be accessed, or state that a protocol was not prepared.	4
	24c	Describe and explain any amendments to information provided at registration or in the protocol.	8
Support	25	Describe sources of financial or non-financial support for the review, and the role of the funders or sponsors in the review.	1
Competing interests	26	Declare any competing interests of review authors.	1

For peer review only - http://bmjopen.bmj.com/site/about/guidelines.xhtml

PRISMA 2020 Checklist

Section and Topic	Item #	Checklist item	Location where item is reported
Availability of data, code and other materials	27	Report which of the following are publicly available and where they can be found: template data collection forms; data extracted from included studies; data used for all analyses; analytic code; any other materials used in the review.	1

From: Page MJ, McKenzie JE, Bossuyt PM, Boutron I, Hoffmann TC, Mulrow CD, et al. The PRISMA 2020 statement: an updated guideline for reporting systematic reviews. BMJ 2021;372:n71. doi: 10.1136/bmj.n71

For more information, visit: http://www.prisma-statement.org/

For peer review only
